#  Diretriz Brasileira de Ergometria em População Adulta – 2024 

**DOI:** 10.36660/abc.20240110

**Published:** 2024-02-23

**Authors:** Tales de Carvalho, Odilon Gariglio Alvarenga de Freitas, William Azem Chalela, Carlos Alberto Cordeiro Hossri, Mauricio Milani, Susimeire Buglia, Dalton Bertolim Precoma, Andréa Maria Gomes Marinho Falcão, Luiz Eduardo Mastrocola, Iran Castro, Pedro Ferreira de Albuquerque, Ricardo Quental Coutinho, Fabio Sandoli de Brito, Josmar de Castro Alves, Salvador Manoel Serra, Mauro Augusto dos Santos, Clea Simone Sabino de Souza Colombo, Ricardo Stein, Artur Haddad Herdy, Anderson Donelli da Silveira, Claudia Lucia Barros de Castro, Miguel Morita Fernandes da Silva, Romeu Sergio Meneghello, Luiz Eduardo Fonteles Ritt, Felipe Lopes Malafaia, Leonardo Filipe Benedeti Marinucci, José Luiz Barros Pena, Antônio Eduardo Monteiro de Almeida, Marcelo Luiz Campos Vieira, Arnaldo Laffitte Stier

**Affiliations:** 1 Clínica de Prevenção e Reabilitação Cardiosport Florianópolis SC Brasil Clínica de Prevenção e Reabilitação Cardiosport Florianópolis SC Brasil Clínica de Prevenção e Reabilitação Cardiosport , Florianópolis , SC – Brasil; 2 Universidade do Estado de Santa Catarina Florianópolis SC Brasil Universidade do Estado de Santa Catarina Florianópolis SC Brasil Universidade do Estado de Santa Catarina , Florianópolis , SC – Brasil; 3 Minascor Centro Médico Belo Horizonte MG Brasil Minascor Centro Médico Belo Horizonte MG Brasil Minascor Centro Médico , Belo Horizonte , MG – Brasil; 4 Instituto do Coração do Hospital das Clínicas Faculdade de Medicina Universidade de São Paulo São Paulo SP Brasil Instituto do Coração do Hospital das Clínicas Faculdade de Medicina Universidade de São Paulo São Paulo SP Brasil Instituto do Coração do Hospital das Clínicas da Faculdade de Medicina da Universidade de São Paulo (InCor-HCFMUSP), São Paulo , SP – Brasil; 5 Hospital do Coração São Paulo SP Brasil Hospital do Coração São Paulo SP Brasil Hospital do Coração (HCOr), São Paulo , SP – Brasil; 6 Instituto Dante Pazzanese de Cardiologia São Paulo SP Brasil Instituto Dante Pazzanese de Cardiologia São Paulo SP Brasil Instituto Dante Pazzanese de Cardiologia , São Paulo , SP – Brasil; 7 Universidade de Brasília Brasília DF Brasil Universidade de Brasília Brasília DF Brasil Universidade de Brasília (UnB), Brasília , DF , Brasil; 8 Hasselt University Hasselt Bélgica Hasselt University Hasselt Bélgica Hasselt University , Hasselt – Bélgica; 9 Jessa Ziekenhuis Hasselt Bélgica Jessa Ziekenhuis Hasselt Bélgica Jessa Ziekenhuis , Hasselt – Bélgica; 10 Sociedade Hospitalar Angelina Caron Campina Grande do Sul PR Brasil Sociedade Hospitalar Angelina Caron Campina Grande do Sul PR Brasil Sociedade Hospitalar Angelina Caron , Campina Grande do Sul , PR – Brasil; 11 Instituto de Cardiologia do Rio Grande do Sul Porto Alegre RS Brasil Instituto de Cardiologia do Rio Grande do Sul Porto Alegre RS Brasil Instituto de Cardiologia do Rio Grande do Sul , Porto Alegre , RS – Brasil; 12 Hospital Arthur Ramos Maceió AL Brasil Hospital Arthur Ramos Maceió AL Brasil Hospital Arthur Ramos , Maceió , AL – Brasil; 13 Hospital do Coração Alagoano Maceió AL Brasil Hospital do Coração Alagoano Maceió AL Brasil Hospital do Coração Alagoano , Maceió , AL – Brasil; 14 Universidade de Pernambuco Recife PE Brasil Universidade de Pernambuco Recife PE Brasil Universidade de Pernambuco (UPE), Recife , PE – Brasil; 15 Hospital Sírio Libanês São Paulo SP Brasil Hospital Sírio Libanês São Paulo SP Brasil Hospital Sírio Libanês , São Paulo , SP – Brasil; 16 Procardio Clínica Cardiológica Natal RN Brasil Procardio Clínica Cardiológica Natal RN Brasil Procardio Clínica Cardiológica , Natal , RN – Brasil; 17 Instituto Estadual de Cardiologia Aloysio de Castro Rio de Janeiro RJ Brasil Instituto Estadual de Cardiologia Aloysio de Castro Rio de Janeiro RJ Brasil Instituto Estadual de Cardiologia Aloysio de Castro (IECAC), Rio de Janeiro , RJ – Brasil; 18 Instituto Nacional de Cardiologia do Rio de Janeiro Rio de Janeiro RJ Brasil Instituto Nacional de Cardiologia do Rio de Janeiro Rio de Janeiro RJ Brasil Instituto Nacional de Cardiologia do Rio de Janeiro , Rio de Janeiro , RJ – Brasil; 19 Linkcare Saúde Rio de Janeiro RJ Brasil Linkcare Saúde Rio de Janeiro RJ Brasil Linkcare Saúde , Rio de Janeiro , RJ – Brasil; 20 Faculdade São Leopoldo Mandic Valinhos SP Brasil Faculdade São Leopoldo Mandic Valinhos SP Brasil Faculdade São Leopoldo Mandic , Valinhos , SP – Brasil; 21 Universidade Federal do Rio Grande do Sul Porto Alegre RS Brasil Universidade Federal do Rio Grande do Sul Porto Alegre RS Brasil Universidade Federal do Rio Grande do Sul (UFRGS), Porto Alegre , RS – Brasil; 22 Hospital de Clínicas de Porto Alegre Porto Alegre RS Brasil Hospital de Clínicas de Porto Alegre Porto Alegre RS Brasil Hospital de Clínicas de Porto Alegre , Porto Alegre , RS – Brasil; 23 Hospital Moinhos de Vento Porto Alegre RS Brasil Hospital Moinhos de Vento Porto Alegre RS Brasil Hospital Moinhos de Vento , Porto Alegre , RS – Brasil; 24 Universidade Federal do Rio de Janeiro Rio de Janeiro RJ Brasil Universidade Federal do Rio de Janeiro Rio de Janeiro RJ Brasil Universidade Federal do Rio de Janeiro (UFRJ), Rio de Janeiro , RJ – Brasil; 25 CLINIMEX – Clínica de Medicina de Exercício Rio de Janeiro RJ Brasil CLINIMEX – Clínica de Medicina de Exercício Rio de Janeiro RJ Brasil CLINIMEX – Clínica de Medicina de Exercício , Rio de Janeiro , RJ – Brasil; 26 Universidade Federal do Paraná Curitiba PR Brasil Universidade Federal do Paraná Curitiba PR Brasil Universidade Federal do Paraná (UFPR), Curitiba , PR – Brasil; 27 Escola Bahiana de Medicina e Saúde Pública Salvador BA Brasil Escola Bahiana de Medicina e Saúde Pública Salvador BA Brasil Escola Bahiana de Medicina e Saúde Pública , Salvador , BA – Brasil; 28 Instituto D’Or de Pesquisa e Ensino Salvador BA Brasil Instituto D’Or de Pesquisa e Ensino Salvador BA Brasil Instituto D’Or de Pesquisa e Ensino , Salvador , BA – Brasil; 29 Hospital Cárdio Pulmonar Salvador BA Brasil Hospital Cárdio Pulmonar Salvador BA Brasil Hospital Cárdio Pulmonar , Salvador , BA – Brasil; 30 Hospital Samaritano Paulista São Paulo SP Brasil Hospital Samaritano Paulista São Paulo SP Brasil Hospital Samaritano Paulista , São Paulo , SP – Brasil; 31 UnitedHealth Group Brasil São Paulo SP Brasil UnitedHealth Group Brasil São Paulo SP Brasil UnitedHealth Group Brasil , São Paulo , SP – Brasil; 32 Faculdade Ciências Médicas de Minas Gerais Belo Horizonte MG Brasil Faculdade Ciências Médicas de Minas Gerais Belo Horizonte MG Brasil Faculdade Ciências Médicas de Minas Gerais , Belo Horizonte , MG – Brasil; 33 Hospital Felício Rocho Belo Horizonte MG Brasil Hospital Felício Rocho Belo Horizonte MG Brasil Hospital Felício Rocho , Belo Horizonte , MG – Brasil; 34 Cardiológica Métodos Gráficos João Pessoa PB Brasil Cardiológica Métodos Gráficos João Pessoa PB Brasil Cardiológica Métodos Gráficos , João Pessoa , PB – Brasil; 35 Hospital Israelita Albert Einstein São Paulo SP Brasil Hospital Israelita Albert Einstein São Paulo SP Brasil Hospital Israelita Albert Einstein , São Paulo , SP – Brasil; 36 Secretaria Municipal de Saúde Curitiba Curitiba PR Brasil Secretaria Municipal de Saúde Curitiba Curitiba PR Brasil Secretaria Municipal de Saúde Curitiba , Curitiba , PR – Brasil

Classes de Recomendação:

**Classe I:** Condições para as quais há evidências conclusivas e, na sua falta, consenso geral de que o procedimento é seguro e útil/eficaz. 

**Classe II:** Condições para as quais há evidências conflitantes e/ou divergência de opinião sobre segurança e utilidade/eficácia do procedimento. 

**Classe IIa:** Peso ou evidência/opinião a favor do procedimento. A maioria aprova. 

**Classe IIb:** Segurança e utilidade/eficácia menos estabelecidas, havendo opiniões divergentes. 

**Classe III:** Condições para as quais há evidências e/ou consenso de que o procedimento não é útil/eficaz e, em alguns casos, pode ser prejudicial. 

Níveis de Evidência

**Nível A:** Dados obtidos a partir de múltiplos estudos randomizados de bom porte, concordantes e/ou de metanálise robusta de estudos randomizados. 

**Nível B:** Dados obtidos a partir de metanálise menos robusta, a partir de um único estudo randomizado e/ou de estudos observacionais. 

**Nível C:** Dados obtidos de opiniões consensuais de especialistas. 

**Table:** 

Diretriz Brasileira de Ergometria em População Adulta – 2024	
O relatório abaixo lista as declarações de interesse conforme relatadas à SBC pelos especialistas durante o período de desenvolvimento deste posicionamento, 2022/2023.	
**Especialista**	**Tipo de relacionamento com a indústria**
Anderson Donelli da Silveira	Nada a ser declarado
Andréa Maria Gomes Marinho Falcão	Nada a ser declarado
Antonio Eduardo Monteiro de Almeida	Nada a ser declarado
Arnaldo Laffitte Stier Junior	Declaração financeira A - Pagamento de qualquer espécie e desde que economicamente apreciáveis, feitos a (i) você, (ii) ao seu cônjuge/ companheiro ou a qualquer outro membro que resida com você, (iii) a qualquer pessoa jurídica em que qualquer destes seja controlador, sócio, acionista ou participante, de forma direta ou indireta, recebimento por palestras, aulas, atuação como *proctor* de treinamentos, remunerações, honorários pagos por participações em conselhos consultivos, de investigadores, ou outros comitês, etc. Provenientes da indústria farmacêutica, de órteses, próteses, equipamentos e implantes, brasileiras ou estrangeiras: - Libbs: educação continuada, Ebatz, Vatis. Outros relacionamentos Participação societária de qualquer natureza e qualquer valor economicamente apreciável de empresas na área de saúde, de ensino ou em empresas concorrentes ou fornecedoras da SBC: - Quanta Diagnóstico Curitiba.
Artur Haddad Herdy	Nada a ser declarado
Carlos Alberto Cordeiro Hossri	Nada a ser declarado
Claudia Lucia Barros de Castro	Nada a ser declarado
Clea Simone Sabino de Souza Colombo	Nada a ser declarado
Dalton Bertolim Precoma	Declaração financeira A - Pagamento de qualquer espécie e desde que economicamente apreciáveis, feitos a (i) você, (ii) ao seu cônjuge/ companheiro ou a qualquer outro membro que resida com você, (iii) a qualquer pessoa jurídica em que qualquer destes seja controlador, sócio, acionista ou participante, de forma direta ou indireta, recebimento por palestras, aulas, atuação como *proctor* de treinamentos, remunerações, honorários pagos por participações em conselhos consultivos, de investigadores, ou outros comitês, etc. Provenientes da indústria farmacêutica, de órteses, próteses, equipamentos e implantes, brasileiras ou estrangeiras: - Novonordisk: Ozempic; Daiichi-Sankyo: Lixiana; Servier: Vastarel; Astrazeneca: Forxiga. B - Financiamento de pesquisas sob sua responsabilidade direta/pessoal (direcionado ao departamento ou instituição) provenientes da indústria farmacêutica, de órteses, próteses, equipamentos e implantes, brasileiras ou estrangeiras: - Bayer: anticoagulante; Janssen: anticoagulante; Novonordisk: cardiometabolismo; Astrazeneca: insuficiência cardíaca, hipercalcemia, disfunção diastólica; Daiichi-Sankyo: anticoagulante; Cardiol: COVID e miocardite; Servier: coronariopatia crônica. Outros relacionamentos Financiamento de atividades de educação médica continuada, incluindo viagens, hospedagens e inscrições para congressos e cursos, provenientes da indústria farmacêutica, de órteses, próteses, equipamentos e implantes, brasileiras ou estrangeiras: - Novonordisk: cardiometabolismo; Daiichi-Sankyo: anticoagulante; Servier: coronariopatia crônica; Torrent: dislipidemia. Participação societária de qualquer natureza e qualquer valor economicamente apreciável de empresas na área de saúde, de ensino ou em empresas concorrentes ou fornecedoras da SBC: - Área da Saúde: medicina nuclear.
Fabio Sandoli de Brito	Nada a ser declarado
Felipe Lopes Malafaia	Nada a ser declarado
Iran Castro	Nada a ser declarado
José Luiz Barros Pena	Nada a ser declarado
Josmar de Castro Alves	Nada a ser declarado
Leonardo Filipe Benedeti Marinucci	Nada a ser declarado
Luiz Eduardo Fonteles Ritt	Declaração financeira A - Pagamento de qualquer espécie e desde que economicamente apreciáveis, feitos a (i) você, (ii) ao seu cônjuge/ companheiro ou a qualquer outro membro que resida com você, (iii) a qualquer pessoa jurídica em que qualquer destes seja controlador, sócio, acionista ou participante, de forma direta ou indireta, recebimento por palestras, aulas, atuação como *proctor* de treinamentos, remunerações, honorários pagos por participações em conselhos consultivos, de investigadores, ou outros comitês, etc. Provenientes da indústria farmacêutica, de órteses, próteses, equipamentos e implantes, brasileiras ou estrangeiras: - Boeringher Lilly: Jardiance; Novonordis: pesquisador em estudos; Astrazeneca; Novartis; Bayer; Bristol; Pfizer. B - Financiamento de pesquisas sob sua responsabilidade direta/pessoal (direcionado ao departamento ou instituição) provenientes da indústria farmacêutica, de órteses, próteses, equipamentos e implantes, brasileiras ou estrangeiras: - MDI Medical. Outros relacionamentos Financiamento de atividades de educação médica continuada, incluindo viagens, hospedagens e inscrições para congressos e cursos, provenientes da indústria farmacêutica, de órteses, próteses, equipamentos e implantes, brasileiras ou estrangeiras: - Novo Nordisk: Ozempic.
Luiz Eduardo Mastrocola	Nada a ser declarado
Marcelo Luiz Campos Vieira	Nada a ser declarado
Mauricio Milani	Nada a ser declarado
Mauro Augusto dos Santos	Nada a ser declarado
Miguel Morita Fernandes da Silva	Declaração financeira A - Pagamento de qualquer espécie e desde que economicamente apreciáveis, feitos a (i) você, (ii) ao seu cônjuge/ companheiro ou a qualquer outro membro que resida com você, (iii) a qualquer pessoa jurídica em que qualquer destes seja controlador, sócio, acionista ou participante, de forma direta ou indireta, recebimento por palestras, aulas, atuação como *proctor* de treinamentos, remunerações, honorários pagos por participações em conselhos consultivos, de investigadores, ou outros comitês, etc. Provenientes da indústria farmacêutica, de órteses, próteses, equipamentos e implantes, brasileiras ou estrangeiras: - Novartis: Entresto; Bayer: Firialta; Astrazeneca: Forxiga; Boehringer: Jardiance. B - Financiamento de pesquisas sob sua responsabilidade direta/pessoal (direcionado ao departamento ou instituição) provenientes da indústria farmacêutica, de órteses, próteses, equipamentos e implantes, brasileiras ou estrangeiras: - Novartis; Bayer.
Odilon Gariglio Alvarenga de Freitas	Nada a ser declarado
Pedro Ferreira de Albuquerque	Declaração financeira A - Pagamento de qualquer espécie e desde que economicamente apreciáveis, feitos a (i) você, (ii) ao seu cônjuge/ companheiro ou a qualquer outro membro que resida com você, (iii) a qualquer pessoa jurídica em que qualquer destes seja controlador, sócio, acionista ou participante, de forma direta ou indireta, recebimento por palestras, aulas, atuação como *proctor* de treinamentos, remunerações, honorários pagos por participações em conselhos consultivos, de investigadores, ou outros comitês, etc. Provenientes da indústria farmacêutica, de órteses, próteses, equipamentos e implantes, brasileiras ou estrangeiras: - MMedicine Cursos: aula de Ergometria. Outros relacionamentos Vínculo empregatício com a indústria farmacêutica, de órteses, próteses, equipamentos e implantes, brasileiras ou estrangeiras, assim como se tem relação vínculo empregatício com operadoras de planos de saúde ou em auditorias médicas (incluindo meio período) durante o ano para o qual você está declarando: - Sócio Cooperado da Unimed Maceió Alagoas.
Ricardo Quental Coutinho	Nada a ser declarado
Ricardo Stein	Outros relacionamentos Financiamento de atividades de educação médica continuada, incluindo viagens, hospedagens e inscrições para congressos e cursos, provenientes da indústria farmacêutica, de órteses, próteses, equipamentos e implantes, brasileiras ou estrangeiras: - Life Genomics
Salvador Manoel Serra	Nada a ser declarado
Susimeire Buglia	Nada a ser declarado
Tales de Carvalho	Nada a ser declarado
William Azem Chalela	Nada a ser declarado

## Sumário

 Parte 1 – Indicações, Aspectos Legais e Formação em Ergometria 08 

1. Introdução 08

 2. Indicações e Contraindicações do TE e TCPE, Inclusive Associados a Imagens 08 

2.1. Indicações Gerais do TE 08

 2.2. Indicações do TE em Situações Clínicas Específicas 09 

**2.2.1. Indicações do TE na Doença Arterial Coronariana** 09 

**2.2.2. Indicações do TE em Assintomáticos** 09 

**2.2.3. Indicações do TE em Atletas** 09 

**2.2.4. Indicações do TE na Hipertensão Arterial Sistêmica** 09 

**2.2.5. Indicações do TE em Valvopatias** 09 

**2.2.6. Indicações na Insuficiência Cardíaca e nas Cardiomiopatias** 11 

**2.2.7. Indicações do TE no Contexto de Arritmias e Distúrbios de Condução** 11 

**2.2.8. Indicações do TE em Outras Condições Clínicas** 12 

2.3. Contraindicações Relativas e Absolutas 12

**2.3.1. Contraindicações Relativas do TE/TCPE** 12 

**2.3.2. Contraindicações Absolutas do TE/TCPE** 14 

2.4. Indicações do TCPE 14

**2.4.1. Indicações Gerais do TCPE** 14 

**2.4.2. Indicações do TCPE em Situações Clínicas Específicas** 14 

 2.5. Indicações do TE/TCPE Associados a Métodos de Imagem 14 

**2.5.1. Cintilografia de Perfusão Miocárdica** 14 

**2.5.2. Indicações da Ecocardiografia sob Estresse** 15 

 3. Aspectos Legais e Condições Imprescindíveis para Realização do TE, TCPE e Quando Associados a Exames Cardiológicos de Imagem 16 

3.1. Aspectos Legais da Prática do TE e TCPE 16

 3.2. Condições Imprescindíveis à Realização do TE e TCPE 16 

3.3. Termo de Consentimento para o TE e TCPE 20

 3.4. Termo de Consentimento ao TE Associado a Métodos de Imagem 21 

 4. Aspectos Referentes à Formação na Área de Atuação de Ergometria 21 

Parte 2 – Teste Ergométrico 23

1. Metodologia do TE 23

1.1. Condições Básicas para a Realização do TE 23

**1.1.1. Equipe** 23 

**1.1.2. Área Física** 23 

**1.1.3. Equipamentos** 23 

**1.1.4. Material para Emergência Médica** 24 

**1.1.5. Medicamentos para Emergência Médica** 24 

**1.1.6. Orientações ao Paciente na Marcação do TE** 24 

 1.2. Procedimentos Básicos para a Realização do TE 24 

**1.2.1. Fase Pré-teste** 24 

**1.2.2. Avaliação Inicial** 25 

**1.2.3. Exame Físico Sumário e Específico** 25 

**1.2.4. Sistema de Monitorização e Registro Eletrocardiográfico** 25 

**1.2.4.1. Sistemas de Três Derivações** 25 

**1.2.4.2. Sistema de 12 Derivações** 25 

**1.2.4.3. Sistema de 13 ou Mais Derivações** 26 

**1.2.4.4. Preparo da Pele para Monitorização Eletrocardiográfica** 26 

**1.2.4.5. Registros Eletrocardiográficos** 26 

**1.2.5. Monitorização dos Dados Hemodinâmicos** 27 

**1.2.5.1. Monitorização da Frequência Cardíaca** 27 

**1.2.5.2. Monitorização da Pressão Arterial Sistêmica** 27 

**1.2.6. Monitoração de Sinais e Sintomas** 27 

**1.2.7. Profilaxia de Complicações no TE** 28 

1.3. Ergômetros 28

**1.3.1. Cicloergômetro** 28 

**1.3.2. Esteira Ergométrica** 28 

**1.3.3. Cicloergômetro de Braço** 28 

**1.3.4. Outros Ergômetros** 28 

1.4. Escolha do Protocolo 28

**1.4.1. Protocolos para Bicicleta Ergométrica** 28 

**1.4.2. Protocolos para Esteira Ergométrica** 29 

**1.4.2.1. Protocolos Escalonados** 29 

**1.4.2.1.1. Protocolo de Bruce** 29 

**1.4.2.1.2. Protocolo de Bruce Modificado** 29 

**1.4.2.1.3. Protocolo de Ellestad** 29 

**1.4.2.1.4. Protocolo de Naughton** 29 

**1.4.2.2. Protocolo em Rampa** 29 

**1.4.3. Protocolo para Ergômetro de Braços** 30 

**1.4.4. Interrupção/Término do Exame** 30 

2. Acurácia, Probabilidade e Escores Pré-teste 30

2.1. Probabilidade Pré-teste de DAC 30

 2.2. Sensibilidade, Especificidade e Valor Preditivo 30 

2.3. Escores e Fatores de Risco DCV Pré-teste 31

 3. Respostas Clínicas e Hemodinâmicas ao Esforço na População Adulta 32 

3.1. Respostas Clínicas 32

**3.1.1. Tolerância ao Esforço** 32 

**3.1.2. Aptidão Cardiorrespiratória/Classificação Funcional** 32 

**3.1.3. Sintomas** 33 

**3.1.4. Ectoscopia/Ausculta** 34 

3.2. Respostas Hemodinâmicas 35

**3.2.1. Frequência Cardíaca** 35 

**3.2.1.1. Frequência Cardíaca de Repouso** 35 

**3.2.1.2. Resposta Cronotrópica** 35 

**3.2.2. Resposta da Pressão Arterial** 36 

**3.2.3. Duplo-Produto** 39 

4. Respostas Eletrocardiográficas 41

4.1. Onda P 42

**4.1.1. Respostas Normais** 42 

**4.1.2. Respostas Anormais** 42 

4.2. Intervalo PR/Segmento PR 43

**4.2.1. Respostas Normais** 43 

**4.2.2. Respostas Anormais** 43 

4.3. Onda Q 43

**4.3.1. Respostas Normais** 43 

**4.3.2. Respostas Anormais** 43 

4.4. Onda R 43

**4.4.1. Respostas Normais** 43 

**4.4.2. Respostas Anormais** 43 

4.5. Onda S 44

**4.5.1. Respostas Normais** 44 

**4.5.2. Respostas Anormais** 44 

4.6. Duração QRS 44

 4. **6.1. Respostas Normais** 44 

**4.6.2. Respostas Anormais** 44 

4.7. Fragmentação de QRS em Alta Frequência 44

**4.7.1. Respostas Normais** 44 

**4.7.2. Respostas Anormais** 45 

4.8. Onda T 45

**4.8.1. Respostas Normais** 45 

**4.8.2. Respostas Anormais** 45 

4.9. Onda U 45

**4.9.1. Respostas Normais** 45 

**4.9.2. Respostas Anormais** 45 

4.10. Repolarização Precoce 46

4.11. Supradesnivelamento do Segmento ST 47

4.12. Ponto J e Infradesnivelamento Ascendente 47

 4.13. Infradesnivelamento do Segmento ST: Ascendente Lento, Horizontal e Descendente 48 

**4.13.1. Sinal de Corcunda do Segmento ST** 49 

4.14. Normalização de Alterações do Segmento ST 49

 4.15. Inclinação ( *Slope* ) ST/FC, Índice ST/FC, Loop ST/FC e Histerese ST/FC 49 

**4.15.1. Inclinação (** ***Slope*** **) ST/FC** 49 

**4.15.2. Índice ST/FC** 49 

**4.15.3.** ***Loop*** **ST/FC** 50 

**4.15.4. Histerese ST/FC** 50 

 4.16. Intervalo QT/QTc/Histerese QT/Dispersão QT 50 

 4.17. Distúrbios da Condução Atrioventricular, Intraventricular e da Formação do Impulso 51 

**4.17.1. Distúrbios da Condução Atrioventricular** 51 

**4.17.1.1. Bloqueio Atrioventricular (BAV) de Primeiro Grau** 51 

**4.17.1.2. Bloqueio Atrioventricular de Segundo Grau Tipo I (Mobitz I)** 52 

**4.17.1.3. Bloqueio Atrioventricular de Segundo Grau Tipo II (Mobitz II)** 52 

**4.17.1.4. Bloqueio Atrioventricular Tipo 2:1/Bloqueio Atrioventricular Avançado ou de Alto Grau/Bloqueio Atrioventricular de Terceiro Grau ou Total** 52 

**4.17.2. Distúrbios da Condução Intraventricular** 53 

**4.17.2.1. Bloqueio de Ramo Esquerdo** 53 

**4.17.2.1.1. Bloqueio do Ramo Esquerdo Preexistente** 53 

**4.17.2.1.2. Bloqueio do Ramo Esquerdo Esforço-induzido** 53 

**4.17.2.2. Bloqueios Divisionais do Ramo Esquerdo** 54 

**4.17.2.3. Bloqueio de Ramo Direito** 54 

**4.17.2.3.1. Bloqueio de Ramo Direito Preexistente** 54 

**4.17.2.3.2. Bloqueio de Ramo Direito Esforço-induzido** 54 

**4.17.3. Distúrbios da Formação do Impulso** 55 

**4.17.3.1. Arritmias Ventriculares** 55 

**4.17.3.2. Arritmias Supraventriculares** 57 

**4.17.3.3. Fibrilação Atrial/Flutter Atrial** 57 

**4.17.3.4. Bradiarritmias/Incompetência Cronotrópica Crônica** 58 

**4.17.3.5. Taquicardia Sinusal Inapropriada** 59 

4.18. Avaliação Metabólica Indireta 59

**4.18.1. VO**_**2**_ **/METs** 59 

**4.18.2. Déficit Funcional Aeróbico (FAI)** 59 

**4.18.3. Déficit Aeróbio Miocárdico (MAI)** 60 

 4.19. Escores de Risco Pós-teste e Variáveis Prognósticas do TE 60 

**4.19.1. Escore de Duke** 60 

**4.19.2. Escore de Athenas/Escore QRS** 61 

**4.19.3. Escore de Raxwal e Morise** 61 

5. Critérios de Interrupção do Esforço 62

6. Elaboração do Laudo do TE 62

6.1. Dados Gerais 62

6.2. Dados Observados, Mensurados e Registrados 62

6.3. Relatório Descritivo 63

6.4. Conclusão 64

6.5. Registros Eletrocardiográficos 64

 7. Exames Realizados Simultaneamente e Adicionalmente ao TE 65 

7.1. Índice Tornozelo-braquial 65

**7.1.1. Realização do Exame ITB** 65 

**7.1.1.1. ITB de Repouso** 65 

**7.1.1.2. ITB Pós-esforço** 66 

**7.1.2. Preparação do Paciente e Técnica de Exame** 66 

7.2. Oximetria Não Invasiva 67

**7.2.1. Equipamentos** 67 

**7.2.2. Procedimentos da Oximetria Não Invasiva** 68 

**7.2.3. Interpretação dos Dados** 68 

7.3. Biomarcadores e Exames Laboratoriais 69

**7.3.1. Lactato Sanguíneo** 70 

**7.3.2. Gasometria Arterial** 70 

 8. Particularidades na Realização e Interpretação do TE em Condições Clínicas Específicas 71 

 8.1. Dextrocardia/ *Situs Inversus* 71 

8.2. Doença de Chagas/Cardiomiopatia Chagásica 72

8.3. Doença Arterial Periférica 73

8.4. Doença de Parkinson 74

8.5. Doenças Valvares 75

**8.5.1. Estenose Aórtica** 75 

**8.5.2. Regurgitação Aórtica** 76 

**8.5.3. Estenose Mitral** 76 

**8.5.4. Regurgitação Mitral** 77 

**8.5.5. Prolapso da Válvula Mitral** 77 

8.6. TE Pós-revascularização Miocárdica 78

**8.6.1. TE após Intervenção Coronária Percutânea** 78 

**8.6.2. TE após Cirurgia de Revascularização Miocárdica** 79 

Parte 3 – Teste Cardiopulmonar de Exercício 79

1. Introdução 79

2. Fisiologia do Exercício Aplicada ao TCPE 79

 3. Ventilação Pulmonar, Gases no Ar Expirado e Variáveis Derivadas 80 

3.1. Ventilação Pulmonar 80

**3.1.1. Espirometria Basal** 80 

**3.1.2. Ergoespirometria** 81 

**3.1.3. Reserva Ventilatória** 81 

3.2. Consumo de Oxigênio 82

3.3. Produção de Gás Carbônico 82

3.4. Limiares Ventilatórios 82

**3.4.1. Primeiro Limiar Ventilatório** 82 

**3.4.2. Segundo Limiar Ventilatório** 83 

3.5. Quociente Respiratório 84

 3.6. Equivalentes Ventilatórios de Oxigênio e Gás Carbônico 84 

 3.7. Pressões Parciais Expiratórias do Oxigênio e Dióxido de Carbono 84 

3.8. Pulso de Oxigênio 85

 3.9. Relação Delta VO _2_ e Delta Carga de Trabalho (ΔVO _2_ /ΔWR) 85 

3.10. Ponto Ótimo Cardiorrespiratório 85

 3.11. Inclinação da Eficiência da Captação do Oxigênio (OUES) 86 

3.12. Ventilação Oscilatória ao Esforço 86

 3.13. Tempo de Recuperação do Consumo de Oxigênio 87 

 3.14. Potência Circulatória e Potência Ventilatória 87 

 3.15. Valores de Referência de Variáveis do TCPE 88 

4. Equipamentos e Metodologia 88

4.1. Ergômetros 88

4.2. Transdutores de Fluxo ou Volume de Ar 88

4.3. Analisadores de Gás 88

4.4. Medições das Trocas Gasosas 88

 4.5. Procedimentos de Calibração, Controle de Qualidade e Higienização 88 

4.6. Protocolos 90

 4.7. *Software* para Análise dos Dados 90 

4.8. Recomendações Prévias aos Pacientes 90

 5. Realização do TCPE em Algumas Situações Específicas 90 

5.1. Insuficiência Cardíaca 90

5.2. Doença Arterial Coronariana 90

5.3. Miocardiopatia Hipertrófica 91

5.4. Valvopatias 91

5.5. Pneumopatias 91

**5.5.1. Doença Pulmonar Obstrutiva Crônica** 91 

**5.5.2. Doença Vascular Pulmonar** 92 

5.6. Diagnóstico Diferencial da Dispneia 93

5.7. Atletas e Exercitantes 94

5.8. Reabilitação Cardiorrespiratória 94

6. Interpretação e Elaboração do Laudo do TCPE 94

 Parte 4 – Teste Ergométrico Associado aos Métodos de Imagem em Cardiologia 94 

 1. Estresses Cardiovasculares Associado aos Métodos de Imagem em Cardiologia 94 

1.1. Cintilografia Perfusional Miocárdica 94

**1.1.1. Metodologia do Estresse Físico – Teste Ergométrico** 94 

**1.1.1.1. Contraindicações à Realização do Estresse Físico na CPM** 95 

**1.1.1.2. Orientações para Marcação do Estresse Físico na CPM** 95 

**1.1.1.3. Realização do Estresse Físico na CPM** 95 

**1.1.1.4. Interpretação do TE na CPM** 96 

**1.1.2. Metodologia das Provas Farmacológicas** 96 

**1.1.2.1. Fármacos que Promovem Vasodilatação** 96 

**1.1.2.1.1. Dipiridamol** 96 

**1.1.2.1.2. Adenosina** 96 

**1.1.2.2. Fármacos que Promovem a Elevação do Consumo de Oxigênio Miocárdico** 97 

**1.1.3. Metodologia do Estresse Combinado** 97 

**1.1.4. Novos Fármacos** 97 

1.2. Ecocardiografia sob Estresse 97

**1.2.1. Metodologia** 98 

**1.2.1.1. Metodologia do Estresse Físico** 98 

**1.2.1.2. Metodologia do Estresse Farmacológico** 98 

**1.2.1.2.1. Dobutamina** 98 

**1.2.1.2.2. Vasodilatadores** 99 

**1.2.1.3. Agentes de Realce Ultrassonográfico** 99 

Referências 100

##  Parte 1 – Indicações, Aspectos Legais e Formação em Ergometria 

## 1. Introdução

 O Teste Ergométrico ou Teste de Exercício (TE) é um exame médico, complementar e rotineiro na prática clínica/cardiológica, no qual o indivíduo é submetido a um esforço físico programado e individualizado, com a finalidade de avaliar as respostas clínica, hemodinâmica, autonômica, eletrocardiográfica, metabólica indireta e eventualmente enzimática. ^1^ ^,^ ^2^ Recebe a denominação de Teste Cardiopulmonar de Exercício (TCPE) quando, ao realizar o TE, são feitas a avaliação dos parâmetros ventilatórios e a análise dos gases expirados. ^3^ A denominação Ergometria contempla o TE e o TCPE. 

Em linhas gerais, o TE e TCPE:

 – Contribuem para o diagnóstico e prognóstico de doenças cardiovasculares, fornecem orientações para a definição das intervenções terapêuticas, auxiliam na adoção de providências relacionadas à prevenção e à prática esportiva, são utilizados nas avaliações periciais médicas e fornecem subsídios para o acompanhamento evolutivo de pacientes. ^1^ ^,^ ^3^ ^-^ ^5^

 – Apresentam alta reprodutibilidade, excelência reconhecida em termos de custo-benefício e custo-efetividade, são passíveis de realização em todas as regiões do Brasil. ^1^ ^,^ ^6^

 – São reconhecidos e legalmente registrados como Área de Atuação em Ergometria pela Comissão Mista de Especialidades médicas. ^7^

 – Têm grande importância como estressor cardiovascular associado aos métodos de imagem em cardiologia, especialmente visando ao diagnóstico e prognóstico da doença cardiovascular isquêmica. ^8^ ^,^ ^9^

 Esta diretriz consolida e atualiza, em um único documento, todas as informações e recomendações presentes nas diretrizes anteriores da SBC sobre o TE e TCPE, abordando novos aspectos não considerados nos documentos anteriores, destacando-se como importantes novidades as informações relacionadas aos exames em população adulta e as necessárias adequações do exame em cenários de síndromes respiratórias agudas. ^1^ ^,^ ^2^ Esta diretriz será relevante fonte de consultas para os cardiologistas em geral e, de forma especial, para os médicos em formação e atuantes na área de Ergometria. 

##  2. Indicações e Contraindicações do TE e TCPE, Inclusive Associados a Imagens 

### 2.1. Indicações Gerais do TE

 O TE está amplamente disponível no Brasil, a um custo acessível e com reconhecida utilidade na prática clínica. ^10^ ^,^ ^11^ É uma importante ferramenta de diagnóstico, para estratificação de risco e determinação de prognóstico em pacientes com doença cardíaca conhecida ou suspeita. Permite avaliar a repercussão das doenças cardiovasculares e a eficácia de terapêuticas implementadas. 

 Indicações e objetivos gerais do TE: ^1^ ^,^ ^6^ ^,^ ^12^ ^-^ ^18^

1) Avaliar sintomas esforço-induzidos.

2) Determinar capacidade funcional.

3) Avaliar o comportamento da pressão arterial.

4) Avaliar o comportamento da frequência cardíaca.

5) Detectar isquemia miocárdica.

 6) Reconhecer as arritmias cardíacas quanto ao tipo, densidade e complexidade. 

 7) Avaliar o comportamento das canalopatias ao esforço. 

 8) Diagnosticar e estabelecer o prognóstico em determinadas doenças cardiovasculares. 

 9) Avaliação de indicação de intervenções terapêuticas. 

 10) Avaliar os resultados de intervenções terapêuticas. 

11) Avaliação pré-operatória.

 12) Avaliar a aptidão cardiorrespiratória e o condicionamento físico. 

 13) Contribuir para prescrição de exercícios físicos, inclusive na reabilitação cardiopulmonar. 

 14) Fornecer subsídios para exames admissionais, periódicos e perícia médica. 

 O TE pode ser realizado em situações clínicas e doenças nas quais se deseje verificar as condições citadas, respeitando as contraindicações relativas e absolutas. 

###  2.2. Indicações do TE em Situações Clínicas Específicas 

 Em determinadas situações clínicas específicas, o TE teve sua efetividade estudada e testada, permitindo a determinação do grau e o nível de recomendação de suas indicações, a serem apresentadas nas próximas sessões. ^6^ ^,^ ^12^ ^-^ ^14^ ^,^ ^17^ ^,^ ^19^

####  2.2.1. Indicações do TE na Doença Arterial Coronariana 

 A doença arterial coronariana permanece como uma das principais doenças por sua morbidade e mortalidade, estimando-se que a prevalência de angina entre 65 a 84 anos seja de 12% a 14% nos homens e 10% a 12% nas mulheres. No Brasil, cerca de 30% das mortes são de causa cardiovascular. ^20^

 TE está indicado na investigação de dor precordial de provável origem cardíaca devido a sua relevância, ampla disponibilidade e custo-efetividade, sendo referendado como a escolha ideal pelo *Choosing Wisely* . ^21^

 A prevalência de DAC assintomática e isquemia silenciosa varia amplamente dependendo da população estudada. Assintomáticos diabéticos apresentam risco relativo (RR) de 2,0 para DAC e a prevalência de TE positivo é de aproximadamente 23% nesses pacientes. ^22^ ^,^ ^23^

 O diagnóstico de isquemia miocárdica silenciosa permite realizar intervenções visando à redução de risco de eventos futuros, inclusive morte. ^24^

 O TE é recomendado para estratificação de risco dos pacientes com DAC estável, definição de prognóstico, eficácia de intervenções e investigação de mudança no quadro clínico. ^25^ ^-^ ^27^

 Mesmo com um TE não isquêmico, os pacientes com suspeita de DAC podem se beneficiar da estratificação de risco aprimorada pelo TE, por meio de variáveis de prognóstico, tais como sintomas esforço-induzidos, capacidade funcional, resposta pressórica e cronotrópica, função autonômica e resposta musculoesquelética. ^28^

 O TE é fundamental em pacientes com DAC para a prescrição inicial de exercícios e subsequentes ajustes na programação de reabilitação cardiovascular ( Tabela 1 ). ^17^ ^,^ ^29^ ^,^ ^30^

**Tabela 1:** – Indicações do TE na doença arterial coronariana sintomática e assintomática

Indicação	GR	NE
Pacientes com probabilidade pré-teste intermediária para DAC incluindo aqueles com bloqueio de ramo direito ou infradesnivelamento do segmento ST <1 mm no ECG de repouso ^14^ ^,^ ^31^	I	A
Diagnóstico diferencial de dor torácica em paciente de baixo risco, estável clínica e hemodinamicamente (após 9 a 12 horas), sem sinais de isquemia eletrocardiográfica e/ou disfunção ventricular e com marcadores sorológicos de necrose normais, na unidade de dor torácica ^32^ ^,^ ^33^	I	A
Prescrição de exercício e avaliação seriada em programa de reabilitação ^29^ ^,^ ^30^	I	A
Sintomas atípicos e anormalidades no ECG de repouso (interpretável) para liberação de atividade física de alta intensidade ^17^ ^,^ ^34^	I	A
Síndromes coronarianas agudas após, no mínimo, 72 horas de completa estabilização clínica e hemodinâmica para estratificação de risco e definição terapêutica ^33^ ^,^ ^35^	I	B
Pós IAM, não complicado, antes da alta hospitalar, para estratificação de risco e adequação terapêutica ^36^ ^,^ ^37^	I	B
Avaliação prognóstica na DAC estável* ^38^ ^,^ ^39^	I	B
Investigação de DAC em pacientes sintomáticos, diabéticos e com ECG interpretável ^40^ ^-^ ^42^	I	B
Suspeita de angina vasoespástica ^43^ ^,^ ^44^	IIa	B
Estratificação de risco e definição terapêutica em pacientes de alto risco para DAC ^14^ ^,^ ^45^	IIa	B
Avaliação de assintomáticos com três ou mais fatores de risco clássicos ^46^ ^,^ ^47^	IIa	B
Decisão terapêutica em lesões coronarianas intermediárias detectadas na cineangiocoronariografia ^14^ ^,^ ^26^	IIa	B
Avaliação da eficácia terapêutica farmacológica na DAC ^27^ ^,^ ^48^	IIa	B
Investigação de alterações de repolarização ventricular (desde que infradesnivelamento <1 mm) no ECG de repouso ^6^ ^,^ ^14^	IIa	B
Pacientes sintomáticos após revascularização miocárdica (cirurgia ou intervenção coronária percutânea) ^49^ ^,^ ^50^	IIa	B
Avaliação de assintomáticos após revascularização miocárdica (cirurgia ou intervenção coronária percutânea) para estratificação de risco, ajuste terapêutico, liberação/prescrição de exercícios físicos, inclusive reabilitação ^14^ ^,^ ^49^	IIa	B
Pré-operatório de paciente com risco intermediário ou alto de complicações** ^51^ ^,^ ^52^	IIa	C
Investigação de DAC em pacientes com critérios eletrocardiográficos para sobrecarga ventricular esquerda com depressão do segmento ST <1 mm ^53^ ^,^ ^54^	IIb	B
Avaliação funcional nos casos em que outro método tenha avaliado anatomia coronariana ^6^ ^,^ ^14^	IIb	B
Perícia médica e/ou avaliação pela medicina do trabalho ^55^ ^,^ ^56^	IIb	B
Baixa probabilidade de DAC para estratificação de risco cardiovascular ^24^	IIb	C
Portador assintomático de lesão de TCE ou equivalente conhecido para acompanhamento evolutivo e ajuste/decisões terapêuticas ^6^ ^,^ ^14^	IIb	C
Síndromes coronarianas agudas não estabilizadas clínica ou hemodinamicamente ou ainda com alterações eletrocardiográficas persistentes ou marcadores de necrose não normalizados ^14^ ^,^ ^33^	III	B
Pesquisa de DAC em pacientes com BRE, WPW, ritmo de MP, depressão do segmento ST ≥1 mm no ECG de repouso e terapêutica com digitálicos ^6^ ^,^ ^14^	III	B
Presença de lesão de TCE ou equivalente conhecido sintomático ^6^ ^,^ ^14^	III	B
GR: grau de recomendação; NE: nível de evidência; ECG: eletrocardiograma; IAM: infarto agudo do miocárdio; DAC: doença arterial coronariana; HAS: hipertensão arterial sistêmica; MP: marca-passo; BRE: bloqueio de ramo esquerdo; TCE: tronco de coronária esquerda; WPW: síndrome de Wolff-Parkinson-White. *Avaliação prognóstica/evolutiva da DAC poderá ser necessária anualmente, de acordo com a condição clínica. **Ver classificação do risco intrínseco da cirurgia de complicações cardíacas da 3ª Diretriz de Avaliação Cardiovascular Perioperatória da Sociedade Brasileira de Cardiologia. ^*51,52*^		

#### 2.2.2. Indicações do TE em Assintomáticos

 O TE apresenta papel relevante na avaliação de pacientes assintomáticos por permitir determinar o prognóstico e o risco de futuras anormalidades através de suas variáveis (FC, pressão arterial, eletrocardiograma etc.). ^57^ ^,^ ^58^

 A aptidão cardiorrespiratória (capacidade funcional) determinada no TE é considerada um marcador fundamental de saúde e definidor de metas terapêuticas e preventivas. Em pacientes assintomáticos com comorbidades, auxilia na prescrição de exercícios de modo a promover a saúde e o bem-estar. O TE é viável e seguro mesmo em pacientes com idade avançada e comorbidades significativas. ^59^ ^,^ ^60^

 O TE também aprimora a estratificação de risco de um indivíduo assintomático quanto a estar fisicamente apto para desempenho de suas atividades físicas laborais, sem colocar em risco indevido a si mesmo ou a terceiros ( Tabela 2 ). ^61^

**Tabela 2:** – Indicações do TE em pacientes assintomáticos

Indicação	GR	NE
Avaliação de indivíduos com história familiar de DAC precoce (em mulheres <65 anos e em homens <55 anos) – realizar pelo menos um TE até os 40 anos ^45^ ^,^ ^62^	I	B
Rastreamento de indivíduos com história de morte súbita em familiares de primeiro grau ^55^ ^,^ ^63^	IIa	B
Avaliação de sedentários diabéticos para diagnóstico de sintoma moderado ou intenso esforço-induzido e/ou prescrição de exercício ^41^ ^,^ ^64^ ^,^ ^65^	IIa	B
Indivíduos classificados como de alto risco pelo escore de Framingham ^1^ ^,^ ^62^	IIa	B
Avaliação de indivíduo com ocupação de alto risco e/ou responsável pela vida de outros, tais como pilotos, motoristas profissionais, militares, policiais, bombeiros etc. ^14^ ^,^ ^66^	IIa	B
Avaliação pré-participativa para atividades de lazer e esporte recreacional em indivíduos ≥60 anos ^17^ ^,^ ^34^	IIa	C
Pré-operatório de paciente com história familiar de DAC precoce em cirurgia não cardíaca de médio e grande porte ^52^ ^,^ ^67^	IIa	C
Considerar na avaliação pré-participação para atividades de lazer e esporte recreacional em indivíduos de 35 a 59 anos ^17^ ^,^ ^34^	IIb	B
Paciente <35 anos, sem fator de risco cardiovascular, para início de programa de atividade física de intensidade leve ou moderada ^34^	III	C
* GR: grau de recomendação; NE: nível de evidência; DAC: doença arterial coronariana; TE: teste ergométrico. *		

#### 2.2.3. Indicações do TE em Atletas

 A atividade física (AF) é definida como qualquer movimento corporal produzido pelo sistema musculoesquelético. O exercício ou treinamento físico é um programa de atividade física estruturada, repetitiva, com objetivo de recuperar, manter ou melhorar um ou mais componentes da aptidão física (cardiorrespiratório, morfológico, muscular, metabólico ou motor). O atleta é indivíduo de qualquer idade, amador ou profissional, que pratique regularmente exercícios físicos, com maior ênfase no desempenho e, eventualmente, participe de competições esportivas. ^13^ ^,^ ^34^

 O TE fornece dados importantes para a cardiologia, medicina esportiva e preventiva quanto à saúde dos atletas de elite, atletas olímpicos, atletas profissionais, atletas competitivos, federados e/ou pertencentes a clubes esportivos, atletas masters e atletas recreativos (atividade de prazer e de lazer). É utilizado na avaliação pré-participação e permite detectar doenças pulmonares e cardiovasculares latentes (p. ex., asma esforço-induzida, hipertensão, isquemia, arritmias etc.), monitorar intervenções e realizar avaliação prognóstica ( Tabela 3 ). ^13^ ^,^ ^17^ ^,^ ^34^ ^,^ ^68^

**Tabela 3:** – Indicações do TE em atletas

Indicação	GR	NE
Realizar em indivíduos ≥60 anos ao iniciar atividade de alta intensidade, esportiva e competições esportivas ^13^ ^,^ ^17^ ^,^ ^69^	I	B
Rastreamento de indivíduos com história de morte súbita em familiares de primeiro grau ^13^ ^,^ ^17^ ^,^ ^70^	I	B
Indivíduo ≥35 anos com alto risco (escore clínico) em avaliação pré-participação para exercícios de alta intensidade e competições esportivas ^13^ ^,^ ^17^ ^,^ ^34^	IIa	A
Indivíduos de 35-59 anos, considerar no início do programa de exercício de alta intensidade e competições esportivas ^13^ ^,^ ^17^ ^,^ ^34^	IIa	B
História familiar de DAC precoce (em mulheres <65 anos e em homens <55 anos) – realizar pelo menos um TE até os 35 anos ^13^ ^,^ ^17^ ^,^ ^34^	IIa	B
Atleta diabético para diagnóstico de sinais e sintomas esforço-induzidos, estratificação de risco e prognóstico ^17^ ^,^ ^40^ ^,^ ^41^ ^,^ ^64^	IIa	B
No ajuste de carga de treinamento físico de atletas	III	C
Atleta em síndrome de excesso de treinamento sintomática	III	C
* GR: grau de recomendação; NE: nível de evidência; DAC: doença arterial coronariana. *		

####  2.2.4. Indicações do TE na Hipertensão Arterial Sistêmica 

 O comportamento da pressão arterial sistólica (PAS) durante o TE é considerado marcador de risco para desenvolvimento de hipertensão, morte por doença cardiovascular e risco de acidente vascular cerebral. ^71^ ^-^ ^73^ Dados recentes sugerem que a resposta da PA ao exercício de intensidade submáxima tem maior significado clínico e prognóstico do que a PA alcançada no exercício de intensidade máxima. O desempenho físico no TE influi na interpretação da resposta da PA ao exercício. Tanto a hipotensão quanto a PA exagerada servem como marcador prognóstico e indicador de necessidade de investigação de DCV subjacentes ( Tabela 4 ). ^74^ ^,^ ^75^

**Tabela 4:** – Indicações do TE na hipertensão arterial sistêmica

Indicação	GR	NE
Avaliação de hipertensos sintomáticos com ECG normal para investigação de DAC ^27^ ^,^ ^77^ ^,^ ^78^	I	B
Comportamento da PA em pacientes com síndrome metabólica ou diabéticos ^79^ ^,^ ^80^	IIa	B
Em hipertensos, para avaliação de aptidão cardiorrespiratória, estratificação de risco e liberação para prática esportiva ^71^ ^,^ ^76^ ^,^ ^81^ ^,^ ^82^	IIa	B
Ajustes da terapêutica farmacológica anti-hipertensiva ^83^ ^-^ ^85^	IIa	B
Avaliação do comportamento da PA em pacientes sob investigação de hipertensão ^86^ ^,^ ^87^	IIa	B
Avaliação do comportamento pressórico em hipertensos com DAC para estratificação de risco, ajuste terapêutico e liberação para exercícios físicos ^78^ ^,^ ^88^	IIa	B
Avaliação de idosos hipertensos para programa de atividade física ^29^ ^,^ ^34^ ^,^ ^89^	IIa	C
Suspeita de hipotensão arterial esforço-induzida em hipertensos tratados ^90^ ^,^ ^91^	IIa	B
Comportamento da PA em indivíduos com história familiar de HAS ^71^	IIb	B
* GR: grau de recomendação; NE: nível de evidência; DAC: doença arterial coronariana; HAS: hipertensão arterial sistêmica; PA: pressão arterial; ECG: eletrocardiograma. *		

 Em atletas submetidos ao TE, a resposta da PA indexada à carga de esforço foi superior à PASpico como preditora de mortalidade em homens saudáveis, sendo útil na triagem pré-participação. A resposta hipertensiva ao TE esteve associada ao desenvolvimento de hipertensão em atletas jovens. ^76^

#### 2.2.5. Indicações do TE em Valvopatias

 Na doença valvar, o TE deve ser realizado rotineiramente para esclarecimento de sintomas duvidosos, avaliação de indicadores que contribuam na decisão sobre intervenção e para liberação e prescrição de exercícios ( Tabela 5 ). ^92^ ^-^ ^94^ O TE é útil para desmascarar os pacientes “pseudoassintomáticos” e permite o acompanhamento seriado de assintomáticos. ^94^ As intervenções, cirúrgica ou transcateter, são indicadas em pacientes sintomáticos ou com sintomas esforço-induzidos. ^94^

**Tabela 5:** – Indicações do TE em valvopatias

Indicação	GR	NE
Em valvopatia leve e moderada, para confirmação de ausência de sintomas, esclarecimento de sintomas, avaliação da capacidade funcional e prescrição de exercícios físicos ^93^ ^,^ ^94^ ^,^ ^96^ ^,^ ^97^	I	B
Na insuficiência mitral, para esclarecimento de sintomas, avaliação da capacidade funcional, indicação de intervenção e prognóstico ^98^ ^-^ ^100^	IIa	A
EAo para esclarecimento de sintomas, indicação de intervenção e prognóstico ^93^ ^,^ ^94^ ^,^ ^101^ ^,^ ^102^	IIa	A
EAo moderada e grave, em paciente assintomático, para avaliação de marcadores de mau prognóstico e indicação de intervenção ^93^ ^,^ ^94^ ^,^ ^96^ ^,^ ^101^ ^,^ ^103^	IIa	A
No seguimento de IAo para esclarecimento de sintomas, avaliação de capacidade funcional e prognóstico ^104^ ^,^ ^105^	IIa	B
EM assintomática ou presença de sintomas atípicos ou sintomas discordantes com o grau de estenose ^14^ ^,^ ^106^ ^,^ ^107^	IIa	B
No seguimento de EAo grave assintomática pelo menos a cada 6 meses para detecção precoce de sintomas, avaliação funcional e indicação de intervenção ^93^ ^,^ ^108^ ^,^ ^109^	IIa	B
EAo grave, assintomática, com FEVE normal em planejamento familiar para gestação ^110^ ^,^ ^111^	IIa	B
Avaliação pós-intervenção valvar para esclarecimento de sintomas, avaliação da capacidade funcional, prognóstico e prescrição de exercício (incluindo reabilitação cardiovascular) ^93^ ^,^ ^112^	IIa	B
Cirurgia não cardíaca para determinação do risco cirúrgico e capacidade funcional ^52^ ^,^ ^67^ ^,^ ^113^	IIb	B
Nas estenoses ou insuficiências aórtica e mitral, assintomáticas, para determinar a capacidade funcional e prescrição de exercícios ^17^ ^,^ ^29^ ^,^ ^114^	IIb	B
Investigação de DAC em pacientes com valvopatia grave ^115^	III	B
EAo ou mitral grave sintomática ^93^	III	C
* GR: grau de recomendação; NE: nível de evidência; EAo: estenose aórtica; IAo: insuficiência aórtica; DAC: doença arterial coronariana; FEVE: fração de ejeção do ventrículo esquerdo. *		

 A prática de exercícios físicos requer avaliação de sintomatologia, capacidade funcional, características da lesão valvar e sua repercussão na função cardíaca. Indivíduos assintomáticos com lesões de gravidade moderada podem se exercitar intensamente se o TE revelar boa capacidade funcional e ausências de isquemia miocárdica, distúrbios hemodinâmicos e arritmias. ^95^

####  2.2.6. Indicações na Insuficiência Cardíaca e nas Cardiomiopatias 

 Na insuficiência cardíaca (IC) e nas cardiomiopatias, o TE é utilizado no esclarecimento de sintomas, avaliação da tolerância ao esforço/classe funcional, avaliação prognóstica, ajustes terapêuticos e prescrição de programas de exercício ( Tabela 6 ). ^29^ ^,^ ^116^

**Tabela 6:** – Indicações do TE na insuficiência cardíaca e nas cardiomiopatias

Indicação	GR	NE
Na IC e nas cardiomiopatias compensadas, para prescrição e adequação de programa de exercícios (incluindo programas de reabilitação cardiovascular)* ^6^ ^,^ ^13^ ^,^ ^17^ ^,^ ^29^ ^,^ ^119^	IIa	B
Na cardiomiopatia hipertrófica e na IC compensada, em protocolo atenuado, para esclarecimento de sintomas, avaliação da capacidade funcional e marcadores prognósticos (sintomas, arritmia ventricular e resposta pressórica) ^17^ ^,^ ^29^ ^,^ ^120^ ^,^ ^121^	IIa	B
Na cardiomiopatia hipertrófica, de forma seriada, para ajustes de programa de exercícios e atividade esportiva recreacional ^6^ ^,^ ^13^ ^,^ ^17^ ^,^ ^29^ ^,^ ^121^	IIa	B
Pacientes recuperados e assintomáticos, após 3 a 6 meses de quadro agudo de miocardite, para liberação e prescrição de prática de exercícios ^122^ ^,^ ^123^	IIa	B
Prescrição e adequação de programa de exercícios (incluindo reabilitação cardiovascular) em pacientes após transplante cardíaco* ^13^ ^,^ ^29^ ^,^ ^124^ ^,^ ^125^	IIa	C
Na cardiomiopatia hipertrófica ou na IC compensada, de forma seriada, para avaliação do comportamento pressórico e de intervenções terapêuticas ^14^ ^,^ ^18^ ^,^ ^119^ ^,^ ^121^	IIa	C
Reavaliação periódica após miocardite, nos primeiros 2 anos, para identificar progressão silenciosa da doença e estratificação de risco ^115^ ^,^ ^123^ ^,^ ^126^ ^,^ ^127^	IIa	C
Seleção para transplante cardíaco pelo TE (com base nos valores de VO _2_ estimados e não medidos)** ^6^ ^,^ ^115^	III	B
Miocardite, pericardite aguda ou IC descompensada ^6^ ^,^ ^115^	III	C
Diagnóstico de insuficiência cardíaca ^6^ ^,^ ^115^	III	C
GR: grau de recomendação; NE: nível de evidência; IC: insuficiência cardíaca; VO _*2*_ : consumo de O _*2*_ . *Na indisponibilidade do TCPE. **As variáveis obtidas pelo TCPE são fundamentais para a indicação do transplante cardíaco, permitindo detectar com maior precisão os mecanismos responsáveis pela limitação ao esforço.		

 A intolerância ao exercício é uma manifestação típica de pacientes afetados por IC, sendo a classificação funcional e a resposta da FC no TE variáveis importantes de prognóstico. ^117^ ^,^ ^118^

####  2.2.7. Indicações do TE no Contexto de Arritmias e Distúrbios de Condução 

 As arritmias esforço-induzidas são frequentemente causadas por diversas doenças cardiovasculares passíveis de avaliação pelo TE. Podem ser totalmente assintomáticas ou cursar com sintomas que variam de palpitações até síncope. O TE permite investigar a sintomatologia, diagnosticar e quantificar (densidade) as arritmias e estratificar o risco de morte súbita cardíaca (MSC). Apresenta, também, papel relevante nos distúrbios de condução atrioventricular e intraventricular na investigação de suas causas, repercussões e decisões terapêuticas ( Tabela 7 ). ^63^ ^,^ ^128^ ^-^ ^132^

**Tabela 7:** – Indicações do TE no contexto de arritmias e distúrbios de condução

Indicação	GR	NE
Palpitação, síncope, pré-sincope, equivalente sincopal, mal-estar indefinido ou palidez relacionada ao esforço físico e/ou recuperação ^6^ ^,^ ^14^ ^,^ ^133^ ^,^ ^134^	I	B
Arritmia assintomática detectada em exame clínico/complementar, para avaliação do comportamento ao esforço e determinação de prognóstico ^14^ ^,^ ^39^ ^,^ ^133^ ^,^ ^135^ ^,^ ^136^	I	B
No BAVT congênito, para avaliação da resposta ventricular e indicação de marca-passo ^131^ ^,^ ^132^ ^,^ ^137^ ^,^ ^138^	I	B
Em pacientes com taquicardia ventricular catecolaminérgica, para avaliação de terapêutica farmacológica e indicação de cardiodesfibrilador implantável ^132^ ^,^ ^138^ ^,^ ^139^	I	B
Na doença do nó sinusal, para avaliação da resposta cronotrópica* ^14^ ^,^ ^133^ ^,^ ^134^	I	B
Na síndrome do QT longo (sintomática e assintomática), para confirmação diagnóstica, estratificação de risco, avaliação de potencial arritmogênico e de terapêutica ^140^ ^,^ ^141^	I	B
Suspeita diagnóstica de taquicardia ventricular paroxística catecolaminérgica ^115^ ^,^ ^132^ ^,^ ^138^ ^,^ ^139^	I	C
No BAVT congênito, para avaliação da resposta atrial e consequente escolha do tipo de marca-passo ^131^ ^,^ ^132^ ^,^ ^134^ ^,^ ^138^	I	C
Eficácia da terapêutica farmacológica e/ou pós-ablação ^131^ ^-^ ^134^ ^,^ ^142^	IIa	B
Indicação de implante de marca-passo ^14^ ^,^ ^131^ ^,^ ^132^ ^,^ ^138^ ^,^ ^143^	IIa	B
Recuperado de parada cardiorrespiratória, estável clinicamente, para liberação e prescrição de exercício físico (recreacional e/ou reabilitação cardiovascular) ^13^ ^,^ ^144^ ^-^ ^146^	IIa	B
Na síndrome de Brugada (sintomática e assintomática), para confirmação diagnóstica, estratificação de risco e avaliação de potencial arritmogênico e de terapêutica** ^147^ ^,^ ^148^	IIa	B
Suspeita de incompetência cronotrópica ^6^ ^,^ ^14^ ^,^ ^149^ ^-^ ^151^	IIa	B
Marca-passo com biossensor, para avaliação do comportamento da frequência cardíaca ^131^ ^,^ ^132^ ^,^ ^134^ ^,^ ^137^ ^,^ ^138^ ^,^ ^152^	IIa	B
Avaliação de marca-passo, ressincronizador e/ou cardiodesfibrilador implantável, para ajustes de programação ^131^ ^,^ ^132^ ^,^ ^134^ ^,^ ^137^ ^,^ ^138^ ^,^ ^152^	IIa	C
Rastreamento dos familiares de pacientes com síndrome do QT longo ^13^ ^,^ ^140^ ^,^ ^153^ ^,^ ^154^	IIa	B
Na arritmia conhecida, controlada, para liberação e prescrição de exercício físico (recreacional e/ou reabilitação cardiovascular) ^6^ ^,^ ^13^ ^,^ ^14^ ^,^ ^133^ ^,^ ^134^	IIa	C
Em assintomático com cardiomiopatia arritmogênica praticante de exercícios físicos, anualmente ^17^ ^,^ ^155^ ^-^ ^157^	IIa	C
Na fibrilação atrial persistente (crônica) para avaliação terapêutica, controle da resposta ventricular, estratificação de risco e liberação para exercícios (inclusive reabilitação) ^119^ ^,^ ^158^ ^,^ ^159^	IIa	C
Avaliação do comportamento de via anômala (pré-excitação) e do potencial arritmogênico ^6^ ^,^ ^13^ ^,^ ^14^ ^,^ ^133^ ^,^ ^134^	IIb	B
Displasia arritmogênica do ventrículo direito para estratificação de risco e liberação de exercícios físicos ^14^ ^,^ ^134^ ^,^ ^135^	IIb	B
Em portadores de desfibrilador cardíaco implantável para avaliação funcional, prognóstica, da eficácia terapêutica e liberação para programa de exercícios ^131^ ^,^ ^132^ ^,^ ^160^ ^,^ ^161^	IIb	B
Rastreamento dos familiares de pacientes com Síndrome de Brugada** ^13^ ^,^ ^134^ ^,^ ^147^ ^,^ ^162^	IIb	C
Portador de marca-passo de frequência fixa ^115^ ^,^ ^133^	III	B
BAVT adquirido com baixa resposta da frequência ventricular ^115^ ^,^ ^133^	III	B
Arritmia não controlada, sintomática ou com comprometimento hemodinâmico ^115^ ^,^ ^133^	III	C
GR: grau de recomendação; NE: nível de evidência; BAVT: bloqueio atrioventricular total. *Contraindicação absoluta na presença de bloqueio sinoatrial total. **Utilizando derivações precordiais altas e recuperação passiva.		

####  2.2.8. Indicações do TE em Outras Condições Clínicas 

 A Tabela 8 apresenta outras condições clínicas para as quais o TE é recomendado, visando a avaliação funcional, prescrição de exercícios físicos e ajustes terapêuticos. 

**Tabela 8:** – Indicações do TE em outras condições clínicas reconhecidas

Indicação	GR	NE
Assintomáticos com artéria coronária de origem anômala, para estratificação de risco, definição da conduta terapêutica e para liberação de exercícios físicos/atividades esportivas ^13^ ^,^ ^17^ ^,^ ^34^	IIa	B
3 meses após correção cirúrgica de artéria coronária de origem anômala, se assintomático, para liberação de exercícios físicos/atividade esportiva ^13^ ^,^ ^17^ ^,^ ^29^ ^,^ ^163^	IIa	C
Ponte miocárdica para estratificação de risco, decisão terapêutica e liberação para exercícios físicos ^13^ ^,^ ^17^ ^,^ ^29^ ^,^ ^163^ ^,^ ^164^	IIa	C
Cardiomiopatia não compactada de VE, assintomática, FEVE ≥40%, em programa de exercícios de baixa a moderada intensidade ^17^ ^,^ ^127^ ^,^ ^165^	IIa	C
Na doença de Parkinson, para avaliação da tolerância ao esforço e liberação/prescrição de exercícios ^19^ ^,^ ^166^ ^,^ ^167^	IIa	B
Na anemia falciforme, para avaliar capacidade funcional, estratificação de risco e liberação/prescrição de exercícios ^168^ ^-^ ^170^	IIa	C
Paciente em tratamento oncológico, para liberação e prescrição de exercícios (inclusive reabilitação) ^171^ ^,^ ^172^	IIa	C
Avaliação de risco e prognóstico após efeitos colaterais do tratamento de câncer ^173^ ^,^ ^174^	IIb	C
Na doença arterial periférica, para avaliar claudicação, quantificar a isquemia, estratificar o risco e decisão terapêutica ^175^ ^,^ ^176^	I	B
Na doença arterial periférica, para avaliar a capacidade funcional, prescrever e adequar programa de exercícios físicos ^176^ ^,^ ^177^	I	B
Prescrição e adequação de programa de exercícios (incluindo reabilitação cardiovascular) em pacientes em hemodiálise e pós-transplante renal ^178^ ^,^ ^179^	IIb	C
Aneurisma de aorta ou em outras artérias, assintomático, sem critérios para intervenção, para ajustes terapêuticos (p. ex., otimização tratamento anti-hipertensivo) e liberação/prescrição de exercícios (incluindo reabilitação) ^180^	IIa	C
Recuperados de acidente vascular cerebral ou ataque isquêmico transitório, estável clinicamente, para ajustes terapêuticos (p. ex., otimização de tratamento anti-hipertensivo) e liberação/prescrição de exercícios ^181^	IIb	C
No adulto com cardiopatia congênita, estável clinicamente (classe funcional I e II), para prescrição e adequação de programa de exercícios ^17^ ^,^ ^182^ ^,^ ^183^	IIb	B
* GR: grau de recomendação; NE: nível de evidência; VE: ventrículo esquerdo; FEVE: fração de ejeção do ventrículo esquerdo. *		

2.3. Contraindicações Relativas e Absolutas

 O TE geralmente é bem tolerado e seguro quando adequadamente indicado e realizado. Entretanto, situações clínicas específicas podem aumentar os riscos para eventuais complicações, exigindo intervenções médicas imediatas ( Tabela 9 ). O risco de morte cardíaca súbita encontra-se em torno de 1 em 10.000 TE. ^6^ ^,^ ^184^ ^,^ ^185^

**Tabela 9:** – Principais eventos e complicações durante o TE

Evento	Frequência	Comentários
**Morte súbita cardíaca **	1 para 10.000 exames	Dependente do quadro clínico e comorbidades. ^6^ ^,^ ^184^ ^,^ ^185^
**Taquicardia ventricular esforço-induzida**	0,05-2,3%	Risco aumentado de ocorrência se arritmias ventriculares prévias. Risco aumentado de morte por DCV e por todas as causas. ^135^ ^,^ ^186^ ^,^ ^187^ Ocorrência frequente na suspeita de TV polimórfica catecolaminérgica, TV da via de saída do ventrículo direito e TV fascicular. ^63^ ^,^ ^130^ ^,^ ^188^
**Taquicardia supraventricular paroxística**	3,4-15%	Risco aumentado de desenvolver FA. ^189^ Quando reentrante, geralmente exige terapia medicamentosa para interrupção. ^129^ ^,^ ^142^ ^,^ ^190^
**Extrassistolia ventricular esforço-induzida**	2-20%	Quando frequente, risco aumentado de mortalidade (por todas as causas e por DCV) e eventos cardiovasculares. ^191^ ^-^ ^195^ Mais comum em pacientes com DAC: 7% a 20%. ^196^
**Extrassistolia supraventricular esforço-induzida**	4-25%	Ocorrem em até 10% dos pacientes aparentemente saudáveis e em até 25% dos com DAC. Não está associada a mortalidade cardíaca ou IAM. ^189^ ^,^ ^197^ ^,^ ^198^ Em idosos, associa-se a maior risco de FA/FluA. ^199^ ^,^ ^200^
**Fibrilação/flutter atrial esforço-induzidos**	<1%	Costumam causar repercussão hemodinâmica se a resposta ventricular for exacerbada. ^197^ ^,^ ^201^
**Bloqueio intermitente do ramo esquerdo**	0,4-0,5%	DAC e IC são as causas mais prevalentes. Maior risco de mortalidade por todas as causas e eventos cardiovasculares. ^202^ ^,^ ^203^
**Bloqueio intermitente do ramo direito**	0,25%	Geralmente associado à DAC. ^202^ ^-^ ^204^
**Bradiarritmia/BAVT esforço-induzidos**	<0,1%	Na disfunção do nó sinusal, podem ocorrer sintomas de IC e angina. ^133^ Na bradicardia sinusal esforço-induzida, pode ocorrer síncope devido ao reflexo de Bezold-Jarisch. ^205^ ^,^ ^206^ BAVT esforço-induzido pode estar associado à isquemia transitória ou doença degenerativa grave do sistema de condução. ^207^ ^,^ ^208^
**Síndrome coronariana aguda**	0,1-0,5%	Requer interrupção imediata do esforço. ^6^ ^,^ ^209^ ^,^ ^210^
* DCV: doença cardiovascular; BAVT: bloqueio atrioventricular avançado/total; DAC: doença arterial coronariana; IC: insuficiência cardíaca; TV: taquicardia ventricular; IAM: infarto agudo do miocárdio; FA: fibrilação atrial; FluA: flutter atrial. *		

#### 2.3.1. Contraindicações Relativas do TE/TCPE

 São situações clínicas de alto risco para a realização do TE/TCPE que exijam a adoção de eventuais condutas preventivas e terapêuticas ( Tabela 10 ). Tais medidas incluem a realização do TE exclusivamente em ambiente hospitalar e cuidados especiais: adequação de protocolos e carga de esforço a ser atingida no TE, rigorosa observação de sintomas, medições mais frequentes da pressão arterial, presença de pessoal e equipamento para reprogramação de marca-passo/CDI. 

**Tabela 10:** – Contraindicações relativas e eventuais condutas no TE/TCPE 1,6,12-17

Ambiente hospitalar + Cuidados especiais	Cuidados especiais
Dor torácica aguda: realizar exclusivamente em hospital, idealmente em unidade de dor torácica, seguindo rigorosamente o protocolo	Cardiomiopatia hipertrófica não obstrutiva
Estenoses valvares graves em assintomáticos*	Marca-passo unicameral, ventricular, sem resposta de frequência (modo estimulação VVI)
Insuficiências valvares graves*	Insuficiência cardíaca compensada avançada (classe III da NYHA)
IAM não complicado (a partir do 5º dia e estável clinicamente)	AVC ou ataque isquêmico transitório recente (menos que 2 meses) ^9^
Angina instável após 72 horas de estabilização*	Aneurisma de aorta ou em outras artérias, sem critérios para intervenção
Doença conhecida do tronco da coronária esquerda ou equivalente, em assintomático*	FA ou FluA assintomáticos detectados na avaliação pré-teste, com paciente informando desconhecimento da arritmia**
Suspeita de arritmias complexas (taquiarritmias e bradiarritmias), QT longo e síndrome de Brugada	FA (persistente ou crônica) ou FluA crônico com FC elevada em repouso**
Síncope por provável etiologia arritmogênica ou suspeita de bloqueio atrioventricular de alto grau ou total esforço-induzido	Gravidez***
Insuficiência renal dialítica	
Cardiodesfibrilador implantado (CDI)	
Cardiopatias congênitas complexas acianóticas	
Hipertensão pulmonar importante ou sintomática*	
Cardiomiopatia hipertrófica obstrutiva com gradiente de repouso grave*	
Anemia grave (hemoglobina <8,0g/dL)** ^211^ ^,^ ^212^	
FA: fibrilação atrial; FLuA: flutter atrial; FC: frequência cardíaca. *Situação em que o risco/benefício do exame deverá ser criteriosamente avaliado. **Situação em que o risco/benefício do exame deverá ser criteriosamente avaliado e provavelmente resultará na decisão de adiar ou cancelar o exame. ***Em nível submáximo de esforço, grávidas em situações específicas (p. ex., valvopatias e cardiopatias congênitas), após exclusão das contraindicações clínicas e obstétricas absolutas. Não é recomendado como exame de rotina. ^*213,214*^	

#### 2.3.2. Contraindicações Absolutas do TE/TCPE

 São consideradas contraindicações absolutas, não devendo realizar o TE e o TCPE, a presença das situações constantes no Quadro 1 . ^1^ ^,^ ^6^ ^,^ ^12^ ^-^ ^17^

**Quadro 1:** – Contraindicações absolutas do TE e TCPE

Contraindicações absolutas do TE e TCPE
– Embolia pulmonar aguda ou infarto pulmonar
– Enfermidade aguda, febril ou grave
– Deficiência mental ou física que leva à incapacidade de se exercitar adequadamente
– Intoxicação medicamentosa
– Distúrbios hidroeletrolíticos e metabólicos não corrigidos
– Bloqueio atrioventricular de risco para eventos/complicações*
– Pressão arterial sistólica persistente em repouso ≥180 mmHg ou pressão arterial diastólica >110 mmHg**
– Crise/urgência hipertensiva**
– Hipertireoidismo descontrolado
– Deslocamento recente de retina, em fase de recuperação***
– Cardiopatias congênitas cianóticas descompensadas
– Infarto agudo do miocárdio antes de 5 dias ou com complicações
– Angina instável
– Arritmias cardíacas não controladas
– Estenose aórtica grave sintomática
– Insuficiência cardíaca descompensada
– Miocardite aguda ou pericardite
– Dissecção aguda da aorta
– Aneurisma de aorta ou em outras artérias com indicação de intervenção
– Doença pulmonar descompensada
– Diabetes *mellitus* descompensado****
*Considera-se como de risco para eventos/complicações: bloqueio atrioventricular de segundo grau tipo II; bloqueio AV 2:1; bloqueio avançado/alto grau; bloqueio atrioventricular de terceiro grau/total (exceto bloqueio atrioventricular total congênito). **Crise hipertensiva: elevação aguda da pressão arterial (PA) sistólica ≥180 mmHg e/ou PA diastólica ≥120 mmHg, que pode resultar ou não em lesões de órgãos-alvo (LOA), que é dividida em urgência hipertensiva (elevação da PA sem LOA e sem risco de morte iminente; isso permite a redução da PA em 24 a 48 horas) e emergência hipertensiva (elevação da PA com LOA aguda ou em progressão e risco imediato de morte; requer redução rápida e gradual da PA em minutos a horas, com medicamentos intravenosos). ^*215*^ ***Para retorno à atividade física, principalmente em carga moderada/alta, são necessárias a avaliação e a liberação por parte do oftalmologista. ^*216,217*^ ****Caso o paciente com diabetes tipo II tenha feito o automonitoramento da glicemia no pré-teste ou no dia do exame, suspender exercícios físicos se glicemia >300 mg/dL (16,7 mmol/L). Caso o paciente com diabetes tipo I tenha feito o automonitoramento da glicemia, suspender exercícios se glicemia >350 mg/dL; se entre 251-350 mg/dL, sugere-se avaliação prévia de presença de cetonas, pois caso em moderada a grande quantidade, deve-se também suspender o exame. ^*64,218*^

## 2.4. Indicações do TCPE

### 2.4.1. Indicações Gerais do TCPE

 As indicações gerais para a realização do TCPE são as mesmas relacionadas ao TE, principalmente quando há necessidade de adicionar as variáveis ventilatórias e metabólicas ( Quadro 2 ). 

**Quadro 2:** – Indicações gerais do TCPE 3,5,219-223

Indicações gerais do TCPE
** 1) Doenças e situações em que a adição da determinação direta dos parâmetros ventilatórios e a análise de gases no ar espirado contribuem para avaliação diagnóstica, estratificação de risco e estabelecimento de condutas preventivas e terapêuticas **
** 2) Determinação das causas limitantes do desempenho cardiorrespiratório e mecanismos fisiopatológicos envolvidos **
** 3) Diagnóstico diferencial de dispneia (asma esforço-induzida, IC, DPOC etc.) **
** 4) Doenças cardiovasculares visando ao diagnóstico, prognóstico e ajustes terapêuticos (DAC, CC, IC etc.) **
**5) Seleção de candidatos ao transplante cardíaco**
** 6) Nas doenças pulmonares (DPOC, asma, enfisema, intersticiais etc.) visando ao diagnóstico, prognóstico e ajustes terapêuticos **
** 7) Resposta terapêutica na hipertensão pulmonar e fibrose cística **
**8) Outras situações:**
– Pré-operatório de cirurgia não cardíaca em pneumopata
– Avaliação após transplante de pulmão, coração e coração-pulmão
– Seleção de modalidade esportiva em atleta competitivo
– Teste seriado para ajustes de intensidade de cargas de treinamento em atletas competitivos de atividades predominantemente aeróbicas
– Avaliação pericial/medicina do trabalho
– Avaliação e prescrição de exercícios para reabilitação cardiovascular, pulmonar e metabólica
* DPOC: doença pulmonar obstrutiva crônica; CC: cardiopatia congênita; DAC: doença arterial coronariana; IC: insuficiência cardíaca. *

###  2.4.2. Indicações do TCPE em Situações Clínicas Específicas 

 Situações clínicas com evidências científicas que possibilitam determinar o grau de recomendação do TCPE são apresentadas na Tabela 11 . 

**Tabela 11:** – Indicações específicas do TCPE

Indicação	GR	NE
Intolerância ao esforço e diagnóstico diferencial de dispneia ^14^ ^,^ ^224^ ^,^ ^225^	I	A
Paciente pós-síndrome respiratória aguda (incluindo COVID-19), para investigação de dispneia, fadiga crônica e/ou intolerância ao esforço ^226^ ^-^ ^228^	I	B
Paciente pós-síndrome respiratória aguda (incluindo COVID-19), para determinação da aptidão cardiorrespiratória e liberação/prescrição de exercícios físicos (incluindo reabilitação) ^13^ ^,^ ^226^ ^,^ ^227^	I	B
Avaliação de broncoespasmo esforço-induzido (associado à prova espirométrica pré e pós-esforço) ^219^ ^,^ ^229^ ^-^ ^231^	IIa	B
Na IC estável, para determinação da aptidão cardiorrespiratória, estratificação de risco, ajustes terapêuticos e liberação/prescrição de exercícios físicos (incluindo reabilitação) ^14^ ^,^ ^232^ ^,^ ^233^	I	A
Na IC, para indicação de implante de dispositivo de suporte ventricular ou transplante cardíaco ^3^ ^,^ ^14^ ^,^ ^224^ ^,^ ^232^ ^,^ ^234^ ^,^ ^235^	I	B
Paciente com DAC, para determinação da aptidão cardiorrespiratória, estratificação de risco, ajustes terapêuticos e liberação/prescrição de exercícios físicos (incluindo reabilitação) ^14^ ^,^ ^29^ ^,^ ^236^	I	B
Na suspeita de DAC, para investigação diagnóstica, estratificação de risco e decisão terapêutica ^237^ ^,^ ^238^	IIa	B
Estenose aórtica grave assintomática, para orientar a decisão terapêutica ^14^ ^,^ ^93^ ^,^ ^239^ ^,^ ^240^	IIa	B
Na doença valvar estável, para determinação da aptidão cardiorrespiratória, ajustes terapêuticos e liberação/prescrição de exercícios físicos (incluindo reabilitação) ^13^ ^,^ ^17^ ^,^ ^29^ ^,^ ^163^	IIa	B
Doença valvar com quadro clínico não correspondente aos achados ecocardiográficos (exceto na estenose aórtica) ^14^ ^,^ ^92^ ^-^ ^94^ ^,^ ^240^	IIa	C
Adulto com CC, para avaliação de sintomas, decisões terapêuticas, estratificação de risco e liberação/prescrição de exercícios físicos (incluindo reabilitação) ^17^ ^,^ ^30^ ^,^ ^241^ ^,^ ^242^	I	B
Avaliação pré-participação de atleta com CC ^17^ ^,^ ^241^ ^-^ ^243^	IIa	B
Atletas competitivos após revascularização miocárdica ou correção de doença valvar, para estratificação de risco e liberação para retorno ao esporte ^17^ ^,^ ^34^	IIa	B
Na cardiomiopatia hipertrófica, para avaliar aptidão cardiorrespiratória, estratificação de risco e liberação/prescrição de exercícios físicos (incluindo reabilitação) ^14^ ^,^ ^121^ ^,^ ^244^ ^,^ ^245^	IIa	B
Hipertensão pulmonar, para diagnóstico e avaliação seriada (em intervalos de 6 a 12 meses) ^14^ ^,^ ^246^	I	B
Hipertensão pulmonar para investigação de piora de sintomas e estratificação de risco ^14^ ^,^ ^246^	IIa	B
Pós-embolia pulmonar aguda (após 3 meses), sintomática com discordância entre ventilação/perfusão na cintilografia pulmonar (V/Q *scan* ), para diagnóstico e seguimento da hipertensão pulmonar ^247^	I	B
Paciente em tratamento oncológico para estratificação de risco e liberação / prescrição de exercícios (inclusive reabilitação) ^248^ ^,^ ^249^	I	B
Pré-operatório de cirurgia não cardíaca em pacientes com baixa capacidade funcional (<4 METs) e/ou alto risco cardiovascular ^14^ ^,^ ^250^	IIa	B
* GR: grau de recomendação; NE: nível de evidência; CC: cardiopatia congênita; DAC: doença arterial coronariana; IC: insuficiência cardíaca. *		

##  2.5. Indicações do TE/TCPE Associados a Métodos de Imagem 

### 2.5.1. Cintilografia de Perfusão Miocárdica

 A cintilografia de perfusão miocárdica (CPM) apresenta indicações nas diversas apresentações clínicas das doenças isquêmicas cardíacas e contribui para definição de sua gravidade. ^9^ Outras indicações são a avaliação de revascularização em pacientes com viabilidade miocárdica e no pré-operatório em situações específicas ( Tabela 12 ). ^26^ ^,^ ^31^ ^,^ ^128^ ^,^ ^251^ ^-^ ^253^

**Tabela 12:** – Escolha de estresse cardiovascular na cintilografia de perfusão do miocárdio 26,31,128,251-253

Indicação	GR	NE
Estresse físico (TE) desde não haja limitação ou contraindicações ao esforço	I	A
Prova farmacológica (dipiridamol ou adenosina) nos casos de BRE, síndrome WPW e marca-passo	I	A
Prova farmacológica (dipiridamol, adenosina, dobutamina) na contraindicação de realização do estresse físico (TE)	I	A
Prova farmacológica (dipiridamol, adenosina, dobutamina) quando existir limitação para o estresse físico (TE)	IIa	A
Protocolo combinado: esforço físico de baixa carga de trabalho após a prova farmacológica (dipiridamol ou adenosina)	IIa	A
* GR: grau de recomendação; NE: nível de evidência; BRE: bloqueio de ramo esquerdo; WPW: síndrome de Wolff-Parkinson-White; TE: teste ergométrico. *		

 Pacientes sintomáticos com risco intermediário para cardiopatia isquêmica são os que mais se beneficiam da CPM para avaliação diagnóstica e prognóstica. Deve ser realizada, preferencialmente, em associação com o esforço físico (estresse físico) desde que o paciente tenha capacidade funcional acima de 5 METs e habilidade para execução do esforço no ergômetro disponível. 

 Os pacientes com bloqueio de ramo esquerdo (BRE), síndrome de Wolff-Parkinson-White (WPW) e marca-passo devem realizar a CPM com prova farmacológica (dipiridamol ou adenosina). ^9^

 Entre as indicações para a realização de CPM, destacam-se pacientes com baixa capacidade funcional ou condições que impedem a interpretação quanto à presença de isquemia no TE e TCPE. A CPM apresenta melhores resultados na estratificação de risco da DAC em pacientes de alta probabilidade pré-teste de DAC. A CPM deverá ser realizada sob estresse farmacológico nos indivíduos com probabilidade pré-teste intermediária e ECG de repouso que impossibilita interpretar isquemia ou naqueles que não conseguem realizar esforço físico ( Tabela 13 ). ^9^

**Tabela 13:** – Critérios para indicação da cintilografia de perfusão do miocárdio em pacientes sintomáticos 9

Indicação	GR	NE	Escore
Alta probabilidade pré-teste de DAC, independentemente do ECG de repouso interpretável e preencha os critérios para estresse físico*	I	A	8
Probabilidade pré-teste intermediária de DAC, com ECG de repouso não interpretável ou não preencha os critérios para estresse físico*	I	A	9
Probabilidade pré-teste intermediária de DAC, com ECG de repouso interpretável e preencha os critérios para estresse físico*	IIa	B	7
Baixa probabilidade pré-teste de DAC, com ECG de repouso não interpretável ou indivíduo que não preencha os critérios para estresse físico*	IIa	B	7
Baixa probabilidade pré-teste de DAC, com ECG de repouso interpretável e preencha os critérios para estresse físico*	III	C	3
GR: grau de recomendação; NE: nível de evidência; DAC: doença arterial coronariana; ECG: eletrocardiograma de 12 derivações; SCA: síndrome coronariana aguda. * Estresse físico: tenha capacidade funcional para realização de atividades físicas diárias acima de 5 METs e habilidade para execução do esforço no ergômetro disponível.			

 Seguindo as recomendações da Diretriz Brasileira de Cardiologia Nuclear, adotamos o escore internacional de indicação: indicação apropriada, se o escore for de 7 a 9; possivelmente apropriada, se o escore for de 4 a 6; raramente apropriada, com escore de 1 a 3. ^9^

 Pacientes assintomáticos sem história de cardiopatia isquêmica e sem TE/TCPE alterado geralmente não se beneficiam da realização da CPM. Os assintomáticos com TE alterado podem se beneficiar da CPM, principalmente se risco intermediário ou alto ( Tabela 14 ). ^9^ ^,^ ^254^

**Tabela 14:** – Critérios de indicação da cintilografia de perfusão do miocárdio para pacientes assintomáticos e/ou com exames cardiológicos prévios 9,254

Assintomáticos – detecção de DAC/estratificação de risco	GR	NE	Escore
Baixo risco (critérios ATP III)	III	A	1
Risco intermediário (critérios ATP III) – ECG não interpretável	IIa	B	5
Risco intermediário (critérios ATP III) – ECG interpretável	IIb	C	3
Alto risco (critérios ATP III)	I	A	7
Alto risco e escore de cálcio (Agatston) entre 100 e 400	IIa	B	7
Escore de cálcio (Agatston) > 400	IIa	B	7
Escore de Duke de risco elevado (<-11)	I	A	8
Escore de Duke de risco intermediário (entre -11 e +5)	IIa	B	7
Escore de Duke de baixo risco (>+5)	III	B	2
* GR: grau de recomendação; NE: nível de evidência; Agatston: escore que define a presença e quantidade de cálcio nas artérias coronárias, caracterizando aterosclerose; ATP III: painel de tratamento em adultos, do programa de detecção, avaliação e tratamento de colesterol elevado em adultos; DAC: doença arterial coronariana. *			

 Em pacientes assintomáticos após intervenção coronária percutânea (ICP) e/ou revascularização cirúrgica do miocárdio, a CPM apresenta relação custo-benefício favorável em seguimentos superiores a 2 e 5 anos, respectivamente. Tais pacientes, caso apresentem sintomas anginosos ou manifestações equivalentes, beneficiam-se da CPM a qualquer momento ( Tabela 15 ). ^9^ ^,^ ^31^ ^,^ ^251^ ^,^ ^252^ ^,^ ^254^

**Tabela 15:** – Critérios de indicação da cintilografia de perfusão do miocárdio após procedimentos de revascularização (CRM ou ICP) 9,31,251,252,254

Revascularização percutânea ou cirúrgica prévia	GR	NE	Escore
Sintomáticos a qualquer momento	I	B	8
Assintomático, CRVM ≥5 anos	IIa	B	7
Assintomático, CRVM <5 anos	IIb	B	5
Assintomático, revascularização percutânea ≥2 anos	IIa	B	6
Assintomático, revascularização percutânea <2 anos	III	C	3
* GR: grau de recomendação; NE: nível de evidência; ICP: intervenção coronária percutânea; CRVM: cirurgia de revascularização miocárdica. *			

 Pacientes com DAC estabelecida e piora dos sintomas (ou com manifestações equivalentes), podem se beneficiar do exame a qualquer momento, com o objetivo principal da quantificação da carga isquêmica (extensão e intensidade dos defeitos) e suporte à decisão terapêutica (GR-NE: I-C). ^252^

 Nos quadros de dor torácica aguda com suspeita de SCA, ECG normal (sem alterações isquêmicas ou necrose) ou ECG não interpretável (BRE, WPW e ritmo de marca-passo) e marcadores de necrose miocárdica (MNM) normais, a CPM em repouso apresenta elevado valor preditivo negativo, permitindo a liberação do paciente da sala de emergência ( Tabela 16 ). ^1^ ^,^ ^9^ ^,^ ^33^ ^,^ ^254^ ^-^ ^258^

**Tabela 16:** – Critérios de indicação da cintilografia de perfusão do miocárdio em pacientes com dor torácica aguda ou pós-síndrome coronariana aguda 1,9,33,254-258

Dor torácica aguda (imagem em repouso)	GR	NE	Escore
SCA possível – ECG normal ou ECG não interpretável*; escore TIMI de baixo risco; MNM limítrofes, minimamente e elevados ou normais	IIa	A	8
SCA possível – ECG normal ou ECG não interpretável*; escore TIMI de alto risco; MNM limítrofes, minimamente elevados ou normais	IIa	A	7/8
SCA possível – ECG normal ou ECG não interpretável*; MNM iniciais negativos. Dor torácica recente (até 2 horas) ou em evolução	IIa	B	7
** Pós-SCA (infarto com ou sem supradesnível do segmento ST) **	**GR**	**NE**	**Escore**
Paciente estável pós-IAM com supradesnível do segmento ST para avaliação de isquemia / viabilidade e cateterismo cardíaco não realizado	IIa	B	8
Paciente estável pós-IAM sem supradesnível do segmento ST para avaliação de isquemia / viabilidade e cateterismo cardíaco não realizado.	IIa	B	9
* GR: grau de recomendação; NE: nível de evidência; BRE: bloqueio do ramo esquerdo; DCA: doença arterial coronariana; ECG: eletrocardiograma de 12 derivações; IAM: infarto agudo do miocárdio; MP: marca-passo; SCA: síndrome coronariana aguda. ECG normal: sem alterações isquêmicas ou de necrose. ECG não interpretável: BRE antigo, ritmo de marca-passo, síndrome de WPW e sobrecarga ventricular esquerda importante; MNM: marcadores de necrose miocárdica. *			

 A pesquisa de viabilidade miocárdica através da CPM auxilia a seleção de pacientes com disfunção ventricular esquerda acentuada, elegíveis para revascularização miocárdica ( Tabela 17 ). ^1^ ^,^ ^9^ ^,^ ^31^ ^,^ ^128^ ^,^ ^258^ ^,^ ^259^

**Tabela 17:** – Critérios de indicação da cintilografia de perfusão do miocárdio para avaliação de viabilidade miocárdica 1,9,31,128,258,259

Avaliação de viabilidade miocárdica	GR	NE	Escore
Disfunção ventricular esquerda acentuada, elegível para revascularização	I	A	9
*GR: grau de recomendação; NE: nível de evidência.*			

 As indicações de CPM referentes a investigação de insuficiência cardíaca, arritmias, síncope, pacientes com escore de cálcio elevado (≥400), diabéticos, insuficiência renal crônica ou com história familiar de cardiopatia isquêmica, avaliação de risco pré-operatório em cirurgia não cardíaca e cirurgia vascular estão contempladas na Atualização da Diretriz de Cardiologia Nuclear. ^9^ ^,^ ^253^

### 2.5.2. Indicações da Ecocardiografia sob Estresse

 O ecocardiograma sob estresse (EcoE) é o método de imagem não invasivo utilizado para diagnóstico, estratificação de risco, prognóstico e avaliação da viabilidade miocárdica na doença arterial coronariana (DAC), valvopatias e cardiomiopatias. ^260^

 Na investigação de isquemia, oferece boa acurácia em pacientes de moderado a alto risco, com leve predomínio da especificidade frente a outros métodos não invasivos de imagem, como a CPM. ^260^ ^-^ ^262^ Entretanto, o método não deve ser considerado como substituto do TE, e está indicado nos pacientes com limitações ou contraindicações à realização do TE. ^262^

 As modalidades de estresse aplicáveis são: físico (em esteira, bicicleta ergométrica ou cicloergômetro de maca); farmacológico com dobutamina (sensibilizada com atropina) ou com vasodilatador (adenosina ou dipiridamol; uso mais raro). Tanto o estresse físico quanto o farmacológico com dobutamina apresentam desempenho diagnóstico similar em relação à isquemia. Entretanto, o estresse físico (GR-NE: I-A) permite melhor interpretação da repercussão funcional, avaliação da aptidão cardiorrespiratória e da disfunção ventricular, além de definição de prognóstico e terapêuticas nas cardiopatias isquêmicas, valvopatias ou cardiomiopatias ( Tabela 18 ). ^8^ ^,^ ^260^ ^,^ ^263^

**Tabela 18:** – Vantagens, desvantagens e contraindicações das diferentes modalidades de estresse 8,260,264

	Cicloergômetro de maca	Bicicleta ergométrica	Esteira	Dobutamina
Aumenta a demanda miocárdica de oxigênio	Sim	Sim	Sim	Sim
Avaliação durante o período de estresse	Sim	Sim	Não	Sim
Permite imagens no estresse máximo	Sim	Sim	Não*	Sim
Avaliação adequada da gravidade das DCV	Sim	Sim	Sim	Sim
Avaliação diagnóstica de isquemia	Sim	Sim	Sim	Sim
Aptidão cardiorrespiratória	Sim	Sim	Sim – melhor	Não
Repercussão funcional	Sim	Sim	Sim	Não
Risco de complicações	Muito baixo	Baixo	Baixo	Baixo
Definição de prognóstico	Sim	Sim	Sim	Limitada
Disponibilidade da modalidade de estresse	Moderada	Baixa	Alta	Alta
Contraindicações	1) Síndrome coronariana aguda instável ou complicada** 2) Arritmias cardíacas graves (TV e BAVT)** 3) Hipertensão moderada/grave (PAS >180 mmHg)** 4) Alteração EcoB que possa tornar o estresse inseguro** 5) Contraindicações absolutas ao TE (Quadro 1)			Mesmas (1 a 4) e 5) Obstrução significativa de via de saída VE
DCV: doenças cardiovasculares; TV: taquicardia ventricular; BAVT: bloqueio atrioventricular total; PAS: pressão arterial sistólica de repouso; VE: ventrículo esquerdo. EcoB: ecocardiograma basal. *Aquisição de imagens feita imediatamente após o esforço, o mais rápido possível. **Contraindicações comuns ao estresse físico e dobutamina.				

 O EcoE pode ser recomendado para estratificação de risco de pacientes com síndrome coronariana aguda em unidades de dor torácica ( Tabela 19 ), e na investigação da DAC estável ( Tabela 20 ). As principais indicações do EcoE em outras DCV não isquêmicas são apresentadas na Tabela 21 . 

**Tabela 19:** – Indicações do ecocardiograma sob estresse na síndrome coronariana aguda em unidade de dor torácica e internação hospitalar 8,260,261,265

Indicação	GR	NE
Pacientes com angina instável de baixo risco controlada clinicamente* antes de decidir a estratégia invasiva	IIa	A
Para avaliar o significado funcional de obstrução coronariana moderada na angiografia, desde que o resultado interfira na conduta	IIa	C
Estratificação de risco após infarto do miocárdio não complicado	IIa	A
Investigação de pacientes com suspeita de doença microvascular,** para estabelecer se há alteração segmentar simultânea à angina e alterações eletrocardiográficas	IIa	C
Parâmetros de *strain* e *strain rate* derivados do *speckle tracking* como ferramenta adjunta ao *wall motion score index* , para diagnóstico e/ou prognóstico de doença coronariana aguda ^266^	IIa	B
Angina instável de alto risco ou na fase aguda do infarto do miocárdio	III	C
GR: grau de recomendação; NE: nível de evidência. *Ausência de recorrência da angina, sem sinais de insuficiência cardíaca, sem alterações no eletrocardiograma inicial/seriado e troponina normal. **Dor anginosa típica com alteração ao eletrocardiograma ou prova funcional, na vigência de cinecoronariografia normal.		

**Tabela 20:** – Indicações do ecocardiograma sob estresse em pacientes com suspeita ou doença coronariana conhecida 8,260,265

Indicação	GR	NE
Investigação de doença coronariana em pacientes com probabilidade pré-teste baixa ou intermediária, incapazes de realizar teste ergométrico e/ou com eletrocardiograma não interpretável	I	B
Investigação após teste ergométrico sem definição diagnóstica	I	B
Investigação após tomografia coronária com escore de cálcio (Agatston) >400 ^254^	I	B
Investigação após angiografia coronária com lesões intermediárias	I	B
Avaliação de viabilidade miocárdica em paciente com disfunção ventricular e elegível à revascularização	I	B
Avaliação pré-operatória de cirurgia não cardíaca de paciente com risco intermediário e alto segundo escores de risco*	I	B
Na avaliação pré-operatória de cirurgia não cardíaca de risco intermediário, em paciente com um ou mais fatores de risco e/ou em baixa classe funcional (<4 METs)	IIa	B
Sintomáticos após revascularização	IIa	B
Assintomáticos após revascularização incompleta	IIa	C
Ecocardiograma com contraste (microbolhas) adjunto às modalidades de estresse, na investigação de isquemia e viabilidade miocárdica	IIa	B
Pré-operatório de cirurgia não cardíaca de risco intermediário e classe funcional ≥4 METs	III	B
Substituição inicial ou rotineira de teste ergométrico em paciente com condições físicas e ECG interpretável	III	C
GR: grau de recomendação; NE: nível de evidência. MET: equivalente metabólico (do inglês, metabolic equivalent of task); DAC: doença arterial coronariana crônica; TE: teste ergométrico; FEVE: fração de ejeção do ventrículo esquerdo; ECG: eletrocardiograma; CF: classe funcional. *Índices de risco com desfechos cardiovasculares: Índice de Risco Cardíaco Revisado (RCRI, Revised Cardiac Risk Index – de Lee); o índice desenvolvido pelo American College of Physicians (ACP); Estudo Multicêntrico de Avaliação Perioperatória (EMAPO). ^*52,267-269*^		

**Tabela 21:** – Indicações do ecocardiograma sob estresse em pacientes com DCV não isquêmicas

Indicação	GR	NE
**Estenose mitral:** na discordância entre sintomas e área/gradiente valvar (área mitral >1,5 cm ^2^ ) ^8^ ^,^ ^93^ ^,^ ^265^ ^,^ ^270^	I	C
**Estenose mitral:** em assintomáticos com área <1cm ^2^ ^8^ ^,^ ^93^ ^,^ ^265^ ^,^ ^270^	IIa	C
**Estenose mitral:** em assintomáticos com área entre 1 e 1,5cm ^2^ em programação de gravidez ou cirurgia de maior porte ^8^ ^,^ ^93^ ^,^ ^265^ ^,^ ^270^	IIb	C
**Insuficiência mitral:** para avaliar discrepância entre a gravidade da doença valvar e sintomas ^8^ ^,^ ^93^ ^,^ ^265^ ^,^ ^270^	IIa	B
**Insuficiência mitral:** quando grave e assintomática, para avaliação de tolerância ao esforço e alterações hemodinâmicas ^8^ ^,^ ^93^ ^,^ ^265^ ^,^ ^270^	IIa	B
**Insuficiência mitral:** para avaliar reserva ventricular esquerda ^8^ ^,^ ^93^ ^,^ ^265^ ^,^ ^270^	IIb	B
**Estenose aórtica:** quando moderada ou acentuada (estágios B e C1), assintomática, para avaliar sintomas esforço-induzidos, respostas da pressão arterial sistêmica ou pulmonar, comportamento dos gradientes e função ventricular esquerda ^8^ ^,^ ^93^ ^,^ ^265^	IIa	B
**Estenose aórtica:** em assintomáticos ou sintomas leves / duvidosos, com baixo fluxo / gradiente e FEVE preservada, na diferenciação de estenose verdadeira de pseudoestenose ^8^ ^,^ ^93^ ^,^ ^265^	IIb	B
**Estenose aórtica:** ecocardiograma de esforço ou com dobutamina em EAo grave sintomática ^8^ ^,^ ^93^ ^,^ ^265^	III	C
**Insuficiência aórtica:** quando grave, assintomática ou com sintomas duvidosos, para avaliar sintomas esforço-induzidos e capacidade funcional ^8^ ^,^ ^93^ ^,^ ^265^	IIa	B
**Insuficiência aórtica:** quando moderada, para esclarecimento de sintomas e exclusão de outras causas ^8^ ^,^ ^93^ ^,^ ^265^	IIa	B
**Insuficiência aórtica:** ecocardiograma sob estresse (exercício ou com dobutamina), para quantificar IAo na discordância entre a gravidade da lesão e sintomas ^8^ ^,^ ^93^ ^,^ ^265^	III	C
**Prótese aórtica ou mitral:** avaliação de sintomas, confirmação de estenose hemodinamicamente significativa e/ou incompatibilidade paciente-prótese, quando o gradiente transprotético em repouso for leve a moderado (posição aórtica, entre 20-40 mmHg; posição mitral, entre 5-10 mmHg) ^265^ ^,^ ^271^	IIa	B
**Cardiomiopatia hipertrófica:** em sintomáticos, com gradiente intraventricular de repouso ou provocável por manobra de Valsalva <50 mmHg, para avaliação do grau de obstrução dinâmica e refluxo mitral durante o esforço ^8^ ^,^ ^121^ ^,^ ^272^	I	B
**Cardiomiopatia hipertrófica:** em assintomáticos, sem obstrução dinâmica ao repouso, quando a detecção de gradiente na VSVE é relevante, para orientação de mudança de estilo de vida, mudança profissional e tomada de decisão terapêutica ^8^ ^,^ ^121^ ^,^ ^272^	IIb	C
**Insuficiência cardíaca:** para identificar a causa da dispneia, orientar e monitorar a resposta ao tratamento, deterioração clínica, estratificação de risco e reserva contrátil ^265^ ^,^ ^273^ ^,^ ^274^	IIa	B
**Atletas:** na suspeita ou sintomas (tontura ou síncope) de obstrução dinâmica com desenvolvimento de gradiente de pressão sistólica intraventricular ^265^ ^,^ ^275^ ^,^ ^276^	IIb	B
* GR: grau de recomendação; NE: nível de evidência; IM: insuficiência mitral; EAo: estenose aórtica; IAo: insuficiência aórtica; FEVE: fração de ejeção do ventrículo esquerdo; VSVE: via de saída do ventrículo esquerdo. *		

##  3. Aspectos Legais e Condições Imprescindíveis para Realização do TE, TCPE e Quando Associados a Exames Cardiológicos de Imagem 

### 3.1. Aspectos Legais da Prática do TE e TCPE

 O TE e TCPE são métodos não invasivos, com baixo risco de complicações em populações não selecionadas, fácil acessibilidade e reprodutibilidade. ^6^ ^,^ ^10^ ^,^ ^11^ ^,^ ^277^ Por se tratar de ato médico, são regidos pelo Código de Ética Médica e, assim, o médico deve conhecer as possíveis implicações éticas e jurídicas devidamente abordadas no próprio Código de Ética Médica do Conselho Federal de Medicina (CFM), Código Civil Brasileiro, Código de Proteção ao Consumidor e demais leis vigentes ( Anexo 1 ). 

###  3.2. Condições Imprescindíveis à Realização do TE e TCPE 

 Diante das particularidades dos métodos e determinações legais estabelecidas, tornam-se imprescindíveis as seguintes condições: 

 1) Teste ergométrico e o teste cardiopulmonar de exercício são atos médicos, de exclusiva competência do médico habilitado, cuja presença física é obrigatória em todas as etapas do exame. Não há possibilidade de realização de TE e TCPE utilizando qualquer modalidade de telemedicina, laudo à distância ou mais de um exame simultâneo, por um único médico – mesmo que presencialmente. Essa conduta se deve à necessidade de realização de procedimentos e diagnósticos durante todo o exame, de atribuição única e exclusiva dos médicos, bem como atendimento de eventuais complicações / emergências. 

 2) O médico habilitado executante do exame deve estar inscrito no Conselho Regional de Medicina e apto ao exercício profissional. A recomendação do Departamento de Ergometria, Exercício, Cardiologia Nuclear e Reabilitação Cardiovascular da Sociedade Brasileira de Cardiologia (DERC/SBC) é de que o médico possua Título de Especialista em Cardiologia e Título de Atuação em Ergometria da AMB, ambos devidamente registrados no CFM. 

 3) O TE e TCPE somente devem ser realizados com a solicitação formal médica. 

 4) É obrigatória a obtenção prévia de termo de consentimento livre e esclarecido assinado pelo paciente ou seu representante legal, principalmente no caso de menores de 18 anos de idade. 

 5) Em se tratando de menores de idade ou legalmente incapazes, recomenda-se que o seu representante legal permaneça na sala de exame. 

 6) O serviço de ergometria deve dispor de todos os equipamentos preconizados para realização do exame, bem como equipamentos / medicamentos para o atendimento de emergências, conforme consta nesta diretriz. ^278^ ^-^ ^280^

 7) O médico executante deverá realizar anamnese sumária, exame físico direcionado e registro eletrocardiográfico no pré-teste. Registrar medicamentos em uso, morbidades e fatores de risco. 

 8) Avaliar a presença de contraindicações relativas e absolutas para a realização do exame. 

 9) Na escolha do protocolo de esforço do TE e do TCPE, deverão ser consideradas as condições clínicas do paciente, solicitação médica, disponibilidade de ergômetros e experiência do médico executante. 

 10) Somente liberar o paciente após a estabilização clínica / hemodinâmica. 

 11) Na eventualidade de eventos adversos de natureza grave ou fatal decorrentes do exame, o médico executante assumirá o suporte ao paciente até contato efetivo com o médico assistente e/ou eventual encaminhamento ao serviço de emergência. Sugere-se, em casos de evento fatal, a comunicação e solicitação de parecer da comissão de ética e do Conselho Regional de Medicina. 

 12) Orientar o paciente a retornar ao médico solicitante para as devidas condutas. Caso seja arguido pelo paciente ou seu representante legal sobre o resultado do exame, o médico executante deverá prestar as informações pertinentes. 

 13) A remuneração pelo exame realizado deve contemplar honorários médicos justos e todos os custos operacionais. 

 14) O médico executante deverá seguir as recomendações das autoridades públicas e sanitárias e das entidades médicas referentes às eventuais endemias, epidemias e pandemias, assim como as normas dos núcleos de segurança do paciente. ^281^

 15) A realização de TE e/ou TCPE envolve a obtenção e o tratamento de dados sensíveis dos pacientes, devendo os serviços de ergometria respeitarem a Lei Geral de Proteção de Dados (LGPD) e legislações do CFM. ^282^ ^-^ ^284^

### 3.3. Termo de Consentimento para o TE e TCPE

 O modelo e o processo de obtenção de termo de consentimento livre e esclarecido (TCLE) para a realização de TE e TCPE devem observar os critérios norteadores do Código de Ética Médica e Recomendação do CFM Nº 1/2016. ^285^

###  3.4. Termo de Consentimento ao TE Associado a Métodos de Imagem 

 O TCLE para a realização do TE no ecocardiograma de estresse físico, cintilografia de perfusão do miocárdio e tomografia por emissão de pósitrons deve também observar as determinações dos departamentos e sociedades de especialidades envolvidos, em observância aos critérios norteadores do Código de Ética Médica e Recomendação do CFM Nº 1/2016. ^285^

##  4. Aspectos Referentes à Formação na Área de Atuação de Ergometria 

 A área de atuação em ergometria é homologada pela Comissão Mista de Especialidades (CME) composta pelo CFM, AMB e pela Comissão Nacional de Residência Médica (CNRM). ^7^ A formação na área de atuação de ergometria busca fornecer aprimoramento profissional aos cardiologistas e, consequentemente, melhora da qualidade dos serviços de diagnóstico cardiológico e do atendimento dos pacientes submetidos ao TE e TCPE. 

 A formação deverá observar as determinações legais estabelecidas pelas entidades médicas e as seguintes recomendações do DERC/SBC: 

 1) Ser feita em instituição com serviço de ergometria atuante, legalmente constituído, com inscrição nos órgãos públicos, documentação sanitária e registros regulares e atualizados. A instituição formadora poderá ser submetida a processo de cadastramento, avaliação e de credenciamento por parte do DERC/SBC. 

 2) É considerado quesito mínimo para constituição de uma instituição formadora a realização rotineira de TE. Adicionalmente, é necessária a realização de TCPE e de ambos os métodos (TE/TCPE) associados a outros métodos de imagem, visando cumprir o programa prático. A instituição formadora poderá estabelecer convênio oficial com outra instituição para realização do treinamento prático em TCPE e/ou TE/TCPE associados aos métodos de imagem. 

 3) A forma de seleção de participantes para o programa de formação será de livre escolha da instituição, podendo ser por entrevista e/ou prova teórica e/ou prova prática. Recomenda-se a divulgação da seleção através de edital público contendo os pré-requisitos, forma de inscrição no processo, critérios de seleção, cronograma e divulgação do resultado. A instituição deverá garantir um processo justo, equânime e transparente. 

 4) Como pré-requisito obrigatório à formação em ergometria, o candidato deverá ter concluído Residência Médica em Cardiologia ou ser detentor do Título de Especialista em Cardiologia da AMB/CFM. 

 5) O programa de formação visa que o cardiologista adquira experiência de modo a ser responsável pela realização, interpretação e organização de serviços de TE e TCPE. O programa será teórico-prático com duração de 12 meses (1 ano) e carga horária mínima de 960 horas (48 semanas com 20 horas semanais de formação e adicionalmente 30 dias reservados para férias). 

 6) O programa teórico corresponderá a, no mínimo, 10% e, no máximo, 20% da carga horária total, dedicado exclusivamente às atividades teóricas: aulas, seminários, reuniões científicas, congressos, discussões de artigos de revistas (clube de revistas ou de atualização), sessões de discussão clínica/cardiológica e reuniões interpretativas de exames de complementares (não invasivos e invasivos). 

 7) O programa teórico da instituição deve incluir, no mínimo, todos os tópicos e assuntos abordados nesta diretriz. Sugere-se treinamento em técnicas básicas de pesquisa em exames complementares, noções de metodologia científica, estatística básica, ética e técnicas de comunicação com paciente. 

 8) O treinamento prático deverá ser sob supervisão direta e presencial de preceptor detentor de Título de Especialista em Cardiologia e Título da Área de Atuação em Ergometria. Os programas devem ter uma proporção mínima de um preceptor para, no máximo, dois participantes. 

 9) O treinamento prático divide-se em: treinamento inicial sob supervisão direta do preceptor, correspondendo a pelo menos 25% do período do total de horas do programa prático; treinamento sob supervisão indireta, após aprovação no programa sob supervisão direta, correspondendo a pelo menos mais 55% do período total. Recomenda-se que o número de exames do treinamento prático seja na proporção mínima de 70% de TE, 15% de TCPE, de 15% de TE/TCPE associados aos métodos de imagem. 

 10) Nos programas de formação nos quais o ano adicional em ergometria é reconhecido pelo Ministério da Educação (MEC), mantêm-se todos os pré-requisitos. As atividades devem compreender uma carga horária de 2.880 horas, sendo distribuídas da seguinte maneira: 10% a 20% (288h a 576h) em atividades de cunho teórico e de 80% a 90% (2.304 a 2.592h) em atividades práticas. Nas atividades práticas, recomenda-se que parte dos exames de TE e TCPE seja realizada em associação a outros métodos de imagem, e sugere-se que outra parte em avaliações para programas de reabilitação cardiovascular e de cardiologia do esporte. 

 11) É recomendado o treinamento regular em atendimento de urgência para uma abordagem otimizada dos pacientes com complicações durante os exames. Esse treinamento correspondendo à realização de curso de *Advanced Cardiovascular Life Support* (ACLS) ou de Treinamento de Emergências Cardiovasculares Avançado (TECA-A). 

 12) A instituição formadora poderá fornecer treinamento e/ou formação em outros métodos de diagnóstico e exames cardiológicos independentemente ou simultaneamente à formação em ergometria. Entretanto, caso ocorra essa situação, não poderá haver interferência no programa de formação em ergometria, bem como não será computada como atividade ou carga horária da formação teórica e/ou prática. 

 13) A instituição deverá elaborar avaliação dos participantes, com critérios próprios, durante e/ou ao final do programa de formação. Recomendando-se manter transparência nas avaliações definindo previamente os critérios objetivos que serão exigidos, e incluir uma autoavaliação com escala de atitudes. Caso não haja aprovação, sugere-se que a instituição forneça opções de treinamento adicional para sanar pendências, seguido de nova avaliação. A instituição deverá fornecer certificado oficial ao participante aprovado, bem como declaração de cumprimento de todos os requisitos aqui apresentados. 

 14) Após conclusão da formação, a SBC recomenda realizar a prova para obtenção do Título da Área de Atuação em Ergometria da AMB/Sociedade Brasileira de Cardiologia e subsequente registro no CFM. ^286^

 15) É recomendável que, após o término da formação, haja participação periódica em eventos científicos / programas de atualização em TE e TCPE, em âmbito nacional e/ou internacional, para revalidação e aperfeiçoamento constante da habilitação adquirida durante a formação. 

## Parte 2 – Teste Ergométrico

## 1. Metodologia do TE

 A realização do TE necessita, obrigatoriamente, de obediência às condições metodológicas do exame, visando à segurança do paciente e à obtenção de resultados válidos e reprodutíveis. 

### 1.1. Condições Básicas para a Realização do TE

#### 1.1.1. Equipe

 O TE é realizado por médico habilitado, com experiência no método, obrigatoriamente presente na sala do exame, executando um único exame por vez, que deverá emitir o respectivo laudo. O médico executante poderá ser auxiliado por profissionais da área de saúde (auxiliar de enfermagem ou técnica de enfermagem ou enfermeira) especificamente treinados para auxiliar o exame e participar de eventuais atendimentos de emergência. ^286^ ^,^ ^287^

 A instituição e/ou o médico executante deverão orientar e treinar adequadamente outros possíveis profissionais envolvidos no TE quanto a marcação do exame, higienização de equipamentos, limpeza da sala de exame e transporte de pacientes. 

#### 1.1.2. Área Física

 Ambiente planejado, adequadamente iluminado e ventilado, com dimensões suficientes para acomodação de todos os equipamentos do TE (incluindo maca, cadeira para paciente assentar e carro de emergência), permitindo circulação de pelo menos três pessoas (no mínimo, de 7 m ^2^ ), com temperatura ambiente mantida entre 18 e 22°C, sendo desejável umidade relativa do ar em pelo menos 40%. ^288^ ^-^ ^290^

#### 1.1.3. Equipamentos

 Equipamentos básicos recomendados: ergômetro; sistema de ergometria com monitor para observação do ECG; impressora (ou acesso para servidor de impressão); esfigmomanômetro calibrado e estetoscópio; termômetro de parede; oxímetro digital; cadeiras destinadas ao paciente e médico; maca ou cama; carro de emergência (se sala única); cilindro de oxigênio (junto ao carro de emergência) ou ponto de oxigênio em cada sala de TE; aspirador portátil (junto ao carro de emergência) ou ponto de aspiração em cada sala de TE; lixeiras (lixo comum e hospitalar). ^4^ ^,^ ^13^

 Antes de cada exame, recomenda-se: ^4^ ^,^ ^13^

 – Limpeza do cabo do aparelho de ECG do TE/TCPE com um tecido embebido em álcool a 70%. 

 – Limpeza e desinfecção da barra de apoio do ergômetro, selim do cicloergômetro, estetoscópio etc. Utilizar produtos de higienização segundo as rotinas institucionais. 

 – Preferencialmente, utilizar materiais descartáveis e descarte de maneira adequada e em local apropriado. 

#### 1.1.4. Material para Emergência Médica

 O serviço deverá manter disponível carro de emergência, para suporte básico e avançado de vida, no local de realização do TE e/ou TCPE. Recomenda-se adotar a padronização do carro de emergência da Diretriz de Ressuscitação Cardiopulmonar e Cuidados Cardiovasculares de Emergência da Sociedade Brasileira de Cardiologia (consulte Quadro 17.2 da referida Diretriz: Padronização do carro de emergência na unidade de terapia intensiva e pronto-socorro). ^279^

#### 1.1.5. Medicamentos para Emergência Médica

 Recomenda-se adotar as medicações para suporte básico e avançado de vida conforme padronização da Diretriz de Ressuscitação Cardiopulmonar e Cuidados Cardiovasculares de Emergência da Sociedade Brasileira de Cardiologia (consulte Quadro 17.2 da referida Diretriz: Padronização do carro de emergência na unidade de terapia intensiva e pronto-socorro). ^279^

#### 1.1.6. Orientações ao Paciente na Marcação do TE

 Recomendações a serem feitas ao paciente: ^4^ ^,^ ^13^

 1) Evitar fumar nas 3 horas antes do exame. ^291^

 2) No dia anterior ao exame e, no dia do exame, não realizar esforços físicos exaustivos e que não sejam habituais. 

 3) Evitar jejum ou alimentação excessiva antes do exame; fazer uma refeição leve 2 horas antes. Não ingerir bebidas alcoólicas e/ou bebidas energéticas (ricas em cafeína) na véspera e no dia do exame. 

 4) Comparecer de bermuda ou calça comprida confortável, calçado com solado de borracha e sem salto (de preferência tênis). Para mulheres, aconselha-se utilizar sutiã ou top. 

5) Trazer o pedido médico do exame.

6) Sugere-se trazer TE realizados anteriormente.

 7) A suspensão ou manutenção de uso de medicações: a critério do médico assistente do paciente. 

 Em exames para o diagnóstico de DAC, alguns medicamentos devem ser suspensos por interferir no resultado TE ( Tabela 22 ). Essa suspensão não deve ser feita em exames para prescrição de exercícios e avaliação de resposta terapêutica. As suspensões de medicações devem ser feitas com parcimônia e levar em consideração os riscos de descompensação clínica em prol de benefícios adicionais das informações do exame. Algumas medicações podem influir (positiva ou negativamente) na duração do exercício, na carga de esforço atingida, no limiar de isquemia e angina, na ocorrência de dor anginosa, na depressão de segmento ST, no comportamento da FC e PA, no tempo para normalização do infradesnivelamento etc. ^6^ ^,^ ^13^

**Tabela 22:** – Tempo recomendado de suspensão de medicações em TE para o diagnóstico de DAC 4,6,13,134

Medicação	Suspensão prévia por
**Amiodarona**	30 dias
Betabloqueadores*	4 (cardiosseletivos) e 7 dias (outros)
**Bloqueador de canal de cálcio**	4 dias
**Outros antiarrítmicos**	3 a 5 dias
**Digoxina**	7 dias
**Inibidores da ECA:**	
• Captopril, enalapril	1 dia
• Outros	3 dias
**BRAs**	3 dias
Diuréticos de alça**	3 dias
**Nitratos**	1 dia
**Trimetazidina**	2 dias
**Metildopa e clonidina**	1 dia
**Minoxidil**	2 dias
ECA: enzima conversora de angiotensina; BRAs: bloqueadores de receptores da angiotensina. *Sugere-se realizar a retirada gradual dos betabloqueadores e anti-hipertensivos buscando evitar o fenômeno de rebote. **Considerar com parcimônia a suspensão em caso de insuficiência cardíaca.	

##  1.2. Procedimentos Básicos para a Realização do TE 292 

### 1.2.1. Fase Pré-teste

 O médico executante deverá constatar se as recomendações para realização do TE foram adequadamente cumpridas pelo paciente e esclarecer possíveis dúvidas quanto ao exame e para obtenção do TCLE. Caso haja recusa pelo paciente em assinar o TCLE, o médico executante não poderá realizar o exame. 

### 1.2.2. Avaliação Inicial

 Recomenda-se ao médico executante do TE avaliar: pedido médico, motivo do exame, medicações em uso, sintomas do paciente, realização de anamnese e exame físico dirigidos aos sistemas cardiovascular e respiratório, tendo em vista, inclusive, a identificação de eventuais contraindicações relativas e absolutas para realização do exame ( Tabela 23 ). Deve-se avaliar as atividades cotidianas realizadas pelo paciente, de modo a identificar eventuais limitações e permitir a adequada escolha do protocolo e ergômetro. ^6^ ^,^ ^13^ ^,^ ^293^

**Tabela 23:** – Recomendações quanto à anamnese e ao exame físico dirigido 13,115,293

Anamnese	Exame físico
Sintomas atuais	Ectoscopia geral (anemia, faces sindrômicas, palidez cutânea)
História familiar e fatores de risco	Frequência cardíaca/ pressão arterial
Antecedentes patológicos	Ausculta cardíaca e pulmonar
Medicações em uso	Oximetria*
Tolerância ao esforço físico	Pulsos periféricos e índice tornozelo-braquial**
Realizou TE anteriormente? Teve alguma anormalidade?	Exame adicional direcionado à sintomatologia***
Aplicação de escore clínico pré-teste	
*Exame adicional ao TE recomendado na ICC, valvopatias, cardiomiopatias e pós-COVID. **Exame adicional ao TE para investigação de doença arterial periférica e claudicação. ***Exemplos ausculta de carótida em idosos com suspeita de síncope, medição da PA nos quatros membros inferiores em caso de coarctação de aorta etc.	

 A hiperventilação no pré-teste não é recomendada, e pode causar desconforto torácico, broncoespasmo e alterações eletrocardiográficas que podem interferir na acurácia do TE. ^294^

### 1.2.3. Exame Físico Sumário e Específico

 O exame físico deve ser realizado de forma dirigida de acordo com a anamnese previamente realizada. São obrigatórias a realização de auscultas cardíaca e pulmonar e a medição da pressão arterial e FC de repouso. 

 Ressalta-se a importância das auscultas na avaliação de doenças valvares, na IC, doenças pulmonares, investigação de dispneia e no pós-COVID. Nesses pacientes, recomenda-se avaliar a saturação através de oxímetro digital, pois uma saturação inadequada (SpO _2_ ≤92% em ar ambiente), a princípio, contraindica a realização do TE. Uma dessaturação parcial (SpO _2_ >92% e <95% em ar ambiente) exige atenção especial com monitorização da oximetria digital durante todo o TE (ver seção Exames Realizados Simultaneamente e Adicionalmente ao TE). ^122^ ^,^ ^226^ ^,^ ^228^

###  1.2.4. Sistema de Monitorização e Registro Eletrocardiográfico 

 A monitorização contínua do eletrocardiograma e a realização de registros são obrigatórias em todas as etapas do TE (repouso, esforço e recuperação). Sugere-se utilização de eletrodo para monitorização de eletrocardiograma de longa duração, hipoalergênico e extra-aderente. 

 Recomenda-se a utilização de sistema computadorizado de ergometria para a monitorização do ECG e *software* que permita a obtenção de dados e adequado registro e interpretação do exame. O sistema deverá receber manutenção preventiva conforme legislação vigente, e sugere-se atualização rotineira do sistema. ^13^ ^,^ ^115^

 Recomenda-se a realização de ECG convencional de 12 derivações de forma adicional, precedendo o TE/TCPE. O ECG convencional é um exame complementar não invasivo que permite avaliar a condição cardíaca do indivíduo, podendo contribuir para eventual contraindicação do TE/TCPE prestes a ser realizado. O ECG convencional difere dos registros de ECG no TE por utilizar posicionamento periférico de eletrodos (braços e pernas), em decúbito dorsal, com utilização de filtros de sinal para repouso. O ECG convencional de 12 derivações é um procedimento médico previsto na Classificação Brasileira Hierarquizada de Procedimentos Médicos (Código: 4.01.01.01-0). ^295^

### 1.2.4.1. Sistemas de Três Derivações

 É composto de uma combinação de duas derivações bipolares (CM5 = obrigatório; aVF ou D2M = D2 modificada) e uma unipolar (normalmente V2). Não é mais recomendado o uso no TE tendo em vista a superioridade dos sistemas com mais derivações. ^296^

### 1.2.4.2. Sistema de 12 Derivações

 No TE, recomenda-se utilizar o posicionamento de 12 derivações clássicas de Mason-Likar (ou sua versão modificada preservando CM5) ou o posicionamento para 13 derivações. ^6^ ^,^ ^297^ ^,^ ^298^

**Figura 1:**
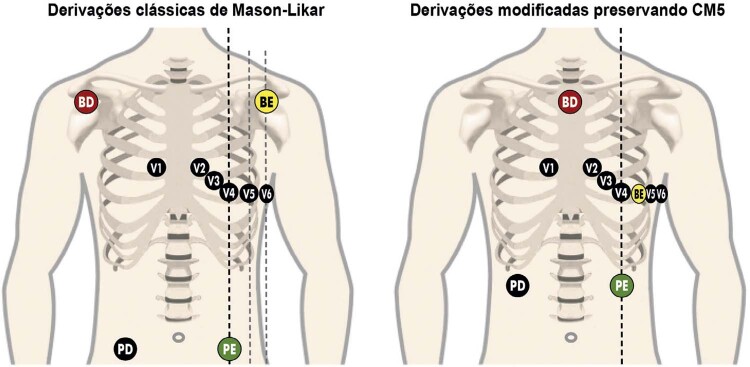
– Posicionamento dos eletrodos nas derivações clássicas de Mason-Likar e derivações modificadas preservando CM. BD: braço direito; BE: braço esquerdo; PD: perna direita; PE: perna esquerda.

 Posicionamento dos eletrodos para obtenção das derivações clássicas de Mason-Likar ( Figura 1 ): 

 1) Eletrodo do braço direito é posicionado próximo à raiz do ombro direito, na linha do 2º espaço intercostal direito. 

 2) Eletrodo do braço esquerdo é posicionado próximo à raiz do ombro esquerdo, na linha do 2º espaço intercostal esquerdo. 

 3) Eletrodo da perna direita é posicionado na porção mais alta da crista ilíaca direita (preferencialmente, logo abaixo do umbigo, na linha hemiclavicular direita). Esse eletrodo tem importância para a referência de impedância elétrica, qualidade técnica dos traçados, e sua posição não interfere diretamente no triângulo de Einthoven. 

 4) Eletrodo da perna esquerda é posicionado na porção mais alta da crista ilíaca esquerda (preferencialmente logo abaixo do umbigo, na linha hemiclavicular esquerda). 

 5) Eletrodos precordiais são posicionados nos pontos de V1 a V6 do ECG clássico: 

 – V1: no 4º espaço intercostal, na linha paraesternal direita. 

 – V2: no 4º espaço intercostal, na linha paraesternal esquerda. 

– V3: entre os eletrodos V2 e V4.

 – V4: no 5º espaço intercostal, na linha hemiclavicular esquerda. 

 – V5: no 5º espaço intercostal, entre V4 e V6, na linha axilar anterior. 

 – V6: no 5º espaço intercostal, na linha axilar média. 

 Observação: Os eletrodos V4, V5 e V6 devem ser colocados no mesmo nível, ao longo de uma linha horizontal que não segue necessariamente o espaço intercostal. 

 Posicionamento dos eletrodos para obtenção das 12 derivações modificadas preservando CM5 ( Figura 1 ): 

 1) Eletrodo do braço direito é posicionado junto à fúrcula esternal (manúbrio). 

 2) Eletrodo do braço esquerdo é posicionado no ponto do V5 do ECG clássico (5º espaço intercostal, na linha axilar anterior). 

 3) Eletrodo da perna direita é posicionado logo abaixo do rebordo costal direito, na linha hemiclavicular direita (ou na porção mais alta da crista ilíaca direita). 

 4) Eletrodo da perna esquerda posicionado logo abaixo do rebordo costal esquerdo, na linha hemiclavicular esquerda (ou na porção mais alta da crista ilíaca esquerda). 

5) Eletrodos precordiais nos pontos:

– V1, V2, V3, V4 e V6 do ECG clássico.

 – V5: é deslocado para o lado esquerdo, posicionado imediatamente antes do V6 clássico. 

**1.2.4.3. Sistema de 13 ou Mais Derivações**

**Figura 2:**
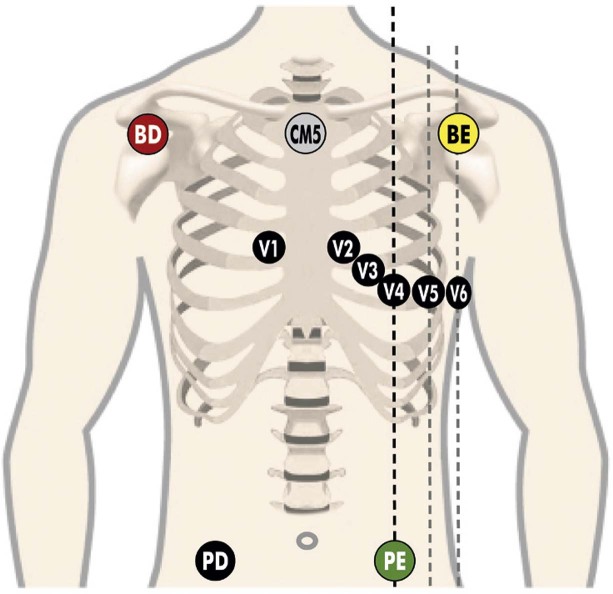
– Posicionamento dos eletrodos no sistema de 13 derivações com CM5. BD: braço direito; BE: braço esquerdo; PD: perna direita; PE: perna esquerda; CM5: eletrodo adicional.

 Este sistema é o principal adotado nos equipamentos de ergometria com tecnologia nacional, em que se acrescenta a derivação CM5 as 12 derivações clássicas de Mason-Likar ( Figura 2 ). Essa derivação é obtida com o acréscimo de um eletrodo posicionado junto à fúrcula esternal (manúbrio). ^152^ ^,^ ^299^

 CM5 é considerada a derivação individual de maior sensibilidade para detecção de isquemia miocárdica, entretanto, com discreta piora da especificidade. Permite monitorar a região anterolateral do ventrículo esquerdo. ^152^

 O sistema de 16 derivações com acréscimo de derivações precordiais direitas (V1r, V2r, V3r) não se estabeleceu na prática clínica, apesar de existirem estudos demonstrando melhora significativa de sensibilidade e especificidade do TE, sobretudo para lesões nas coronárias direita e circunflexa. ^134^

**1.2.4.4. Preparo da Pele para Monitorização Eletrocardiográfica**

 O preparo da pele é fundamental para garantir boa qualidade do traçado eletrocardiográfico. Recomenda-se a limpeza da pele no local de fixação dos eletrodos com gaze embebida em álcool (de 70% a 99%). Em idosos e crianças, deve-se ter maior cuidado com a abrasão devido a maior sensibilidade da pele e propensão a lesões. Nos homens com excesso de pelos nas regiões de fixação dos eletrodos, recomenda-se a realização de tricotomia com lâmina descartável. ^299^

**1.2.4.5. Registros Eletrocardiográficos**

 Os registros eletrocardiográficos devem ser feitos imediatamente após as aferições da pressão arterial e frequência cardíaca no: repouso (posição supina e ortostática); final de cada estágio do esforço; no pico do esforço; fase de recuperação (tempos de um, dois, quatro e seis minutos, pelo menos, ou até normalização de quaisquer alterações eletrocardiográficas). Registros adicionais devem ser realizados na ocorrência de alterações de ritmo, bloqueios átrio e intraventriculares e do segmento ST. 

### 1.2.5. Monitorização dos Dados Hemodinâmicos

**1.2.5.1. Monitorização da Frequência Cardíaca**

 O comportamento da FC reflete a resposta do sistema autonômico ao esforço, fornecendo informações diagnósticas e prognósticas. ^6^ ^,^ ^14^ No TE, conceitua-se como: 

 – Frequência cardíaca máxima (FCmax) de um indivíduo aquela atingida em nível de exaustão ao esforço. 

 – Frequência cardíaca pico (FCpico) é a maior FC observada no pico do esforço, mesmo que não esteja associada à exaustão física. 

 Pode-se estimar a FCmax que um indivíduo alcançará no TE por meio de equações de regressão, ajustadas à idade e/ou associada ao sexo. Não há consenso sobre a melhor equação de estimativa da FCmax. As equações mais utilizadas são: 

**Equações de predição da FCmax para ambos os sexos:**

**FCmax = 220 – idade** (Karvonen et al., 1957) ^300^

**FCmax = 208 – (0,7 × idade)** (Tanaka et al., 2001) ^301^

**Equações de predição da FCmax específicas para homens e mulheres:**

$\text{ FCmax }=192-(0,7\times \text{ idade) }(\text{ Calvert et. al, }1977-\text{ para mulheres }{)}^{302}$ $\text{ FCmax }=201-(0,6\times \text{ idade })(\text{ Calvert et. al., }1977-\text{ para homens }{)}^{302}$

 A FCmax é influenciada pelas condições individuais, o tipo de ergômetro, estado emocional, estado metabólico, capacidade física, uso de fármacos, de dispositivos implantáveis, dentre outros (p. ex., temperatura, umidade relativa do ar etc.). ^301^ ^,^ ^303^ ^,^ ^304^

 Recomenda-se a monitorização contínua da FC durante todo o TE e seu registro associado aos dos traçados do ECG. 

**1.2.5.2. Monitorização da Pressão Arterial Sistêmica**

 A medida da pressão arterial deve ser executada por profissionais devidamente treinados e experientes, e pode ser: ^13^ ^,^ ^134^

 – Manual, com utilização do esfigmomanômetro aneroide. 

 – Semiautomática, com equipamento sem sincronização ao ECG. 

 – Automática, com equipamento com dupla checagem (auscultatória sincronizada ao ECG). 

 A medida automática é limitada em velocidades elevadas de esforço, devido ao maior movimento do corpo e instabilidade do braço. 

 Todas as formas de medição devem utilizar manguito de velcro do tamanho adequado à circunferência do braço e proteção contra excesso de suor (papel toalha ou malha). O estetoscópio e/ou sensor dos equipamentos automáticos devem ser posicionados sobre a artéria braquial. 

 Recomenda-se a medição da PA em ambos os braços no pré-teste e subsequentes medições (durante o teste e recuperação) no braço com maior nível pressórico (normalmente membro superior esquerdo). Em caso de PA elevada no pré-teste, obter medidas repetidas e acuradas em ambos os braços. ^215^

 Contraindica-se a medição da pressão no membro superior com fístula arteriovenosa, esvaziamento ganglionar, trombose, linfedema e/ou retirada de artéria radial para enxerto. ^134^ ^,^ ^305^

 Recomenda-se a medição da PA, pelo menos, no pré-esforço, ao final de cada estágio de protocolo escalonado ou a cada 2 minutos em protocolo de rampa, quando o paciente atingir 5 METs (ver seção 3.2.2. Resposta da Pressão Arterial), no pico do esforço e na recuperação (1, 2, 4 e 6 minutos). Caso haja necessidade, as medições devem ser mantidas por tempo maior na recuperação. Reavaliar sempre que houver discrepâncias ou dúvidas em relação às medições. 

### 1.2.6. Monitoração de Sinais e Sintomas

 Os sinais e sintomas apresentados no repouso, durante o esforço e recuperação devem ser monitorados e descritos no laudo do exame, de forma objetiva, incluindo o motivo de interrupção. Detalhar os sintomas que estejam diretamente relacionados à solicitação do exame. 

 Monitorar a ocorrência de dor torácica, precordial ou retroesternal, acompanhada ou não por dispneia, caracterizando possível angina desencadeada ao esforço. ^306^ ^,^ ^307^

 A dispneia aos esforços pode estar relacionada à cardiopatia, pneumopatia e asma induzida pelo esforço. ^225^ ^,^ ^307^ ^-^ ^309^ Quanto à ectoscopia, salienta-se a necessidade de observação de coloração da pele (palidez, cianose) e sudorese, assim como padrão respiratório e de marcha. ^175^ ^,^ ^310^

### 1.2.7. Profilaxia de Complicações no TE

 Como medidas de profilaxia, recomendamos: ^13^ ^,^ ^152^

 – Respeitar os critérios de realização do TE em nível hospitalar com devida retaguarda. 

 – Escolher o ergômetro e protocolo adequados para o paciente. 

 – Permitir, quando necessário, o apoio na barra da esteira por aumentar a segurança do paciente e favorecer obtenção de melhor traçado eletrocardiográfico. 

 – Observar o comportamento e a postura do paciente sobre o ergômetro. 

 – Respeitar o limite de tolerância ao esforço referido pelo paciente e os critérios de interrupção. 

 – Dispor de material adequado para atendimento de possíveis emergências e intercorrências. 

## 1.3. Ergômetros

 Os ergômetros utilizados no TE/TCPE são desenvolvidos especificamente para este fim, e devem ter registro na Anvisa. Os principais tipos de ergômetro são: cicloergômetro (bicicleta ergométrica; rolo estacionário); esteira ergométrica; cicloergômetro de braço (ergômetro de braço); dentre outros. No Brasil, os mais utilizados são a esteira ergométrica e a bicicleta ergométrica. 

 A escolha do ergômetro deve levar em consideração: indicação do TE (p. ex., síncope e taquicardia ventricular catecolaminérgica – preferir ciclo ergômetro); atividade física desenvolvida habitualmente; exames seriados idealmente com a manutenção do ergômetro; disponibilidade do equipamento; limitações físicas do paciente. ^4^ ^,^ ^6^

### 1.3.1. Cicloergômetro

 A bicicleta ergométrica deve ser preferida em ciclistas, nas limitações neurológicas, visuais ou de equilíbrio e investigações de síncope esforço-induzida (prevenção de quedas). Facilita a medição da pressão arterial e ausculta cardiopulmonar durante o esforço. 

 O incremento da carga de esforço na bicicleta é feito por meio de frenagem mecânica ou eletromagnética. É considerado ideal manter a velocidade de pedalar em 60 rpm para adequada estimativa do VO _2_ por fórmula, mas considerando limitações individuais (exemplo: idade, morbidades, etc.) aceita-se uma variação entre 40 e 70 rpm. 

 Como principais limitações: em idosos e pessoas não habituadas ao ciclismo, dificuldade de coordenação e manutenção da velocidade constante; em indivíduos não habituados ao ciclismo, são encontrados maior valor de PAS e menores valores de FC e VO _2_ (de 5% a 25% menores em relação à esteira). ^1^ ^,^ ^292^

### 1.3.2. Esteira Ergométrica

 É mais adequada na população em geral por permitir melhor adaptação ao ergômetro. Possibilita atingir maiores valores de FC e VO _2_ em comparação ao cicloergômetro. Permite aumentar a carga de esforço por meio da inclinação, de maneira isolada ou em conjunto com o aumento da velocidade. ^1^

 Como principais limitações: maior dificuldade de medida da PA em grandes velocidades; possibilidade de desencadear síndrome vertiginosa; dificuldade de adaptação em pacientes com escalafobia (medo de escadas rolantes) ou medo de esteira rolante; exames cuja indicação envolvam potenciais situações de queda (p. ex., síncope). ^134^

### 1.3.3. Cicloergômetro de Braço

 É recomendado em indivíduos incapacitados de realizar exercícios com os membros inferiores e em praticantes de atividades esportivas predominantemente de membros superiores. ^311^ A medida da pressão arterial deve ser realizada na coxa ou em um dos braços, enquanto o outro se mantém ativo. A massa muscular envolvida no esforço é menor. Quando comparado ao TE em esteira, geralmente atinge menores valores de VO _2 _ e PAS. ^312^

### 1.3.4. Outros Ergômetros

 Existem outras opções, como esteira adaptada para cadeiras de rodas, remo ergômetro, piscina adaptada com corrente de água etc. Cada ergômetro requer protocolo próprio e individualizado, e fórmulas específicas para estimativa do VO _2_ . Devem ser consideradas possíveis variações das respostas hemodinâmicas relacionadas ao ergômetro. ^134^

## 1.4. Escolha do Protocolo

 Os protocolos podem ser divididos quanto ao tipo do esforço: 

1) Incrementais (aumento gradativo de carga):

 – Escalonado (em degraus): com aumento de cargas em etapas (estágio) em tempo predeterminado (a cada 1 ou mais minutos por estágio). Normalmente envolve grandes incrementos de carga ao final de cada estágio. 

 – Rampa: com incrementos pequenos de carga, frequentes (tendendo a linear) e em curtos intervalos de tempo (incrementos em segundos, não podendo atingir 1 minuto). 

 2) Sem incremento (carga fixa): não realiza aumento de cargas durante todo o exame. Quando realizado em esteira ergométrica, mantém velocidade e inclinação fixas. É realizado com cargas de esforço predeterminadas exclusivamente em situações clínicas específicas (p. ex., na determinação da claudicação inicial e absoluta de membros inferiores; no TE associado ao exame de índice tornozelo-braquial pós-esforço). ^1^ ^,^ ^4^

 A escolha do protocolo deve ser individualizada, levar em conta a indicação do TE, o condicionamento físico, eventuais limitações físicas e visar a um tempo de esforço ideal de 10 minutos (com variações entre 8 e 12 minutos). ^1^ ^,^ ^4^

### 1.4.1. Protocolos para Bicicleta Ergométrica

 Existem vários protocolos para bicicleta ergométrica, sendo os principais apresentados na Tabela 24 . A carga de trabalho realizada na bicicleta normalmente é expressa em watts (W). 

**Tabela 24:** – Protocolos para bicicleta ergométrica

Protocolo	Indicado para	Carga inicial	Incremento de carga
Balke	Jovens e adultos	25W a 50W*	25W/2 minutos
Astrand	Adultos	25W	25W/3 minutos
Jones	Sedentários e idosos	25W	15W/1 minuto
Mellorowicz	Bem condicionados ou atletas	50W	50W/2 minutos
Rampa	Todas as populações, sendo o ideal para atletas**	10W a 50W***	5 a 50W/1 min. Subdividir o aumento em valores iguais e incremento em intervalos regulares (<60 segundos)****
*Nos indivíduos jovens e sadios, recomenda-se iniciar com 50W; nos limitados, com carga livre; e nos demais, com 25W. **Ajustável à expectativa de desempenho físico e atividades diárias do indivíduo. ***Nos atletas, recomenda-se iniciar com pelo menos 50W; nos limitados, com 10W; e nos demais, com 25W. ****Exemplo: protocolo de rampa com incremento de carga de 15W/1 minuto = aumentar a carga em 5W a cada 20 segundos.			

 O protocolo mais utilizado é o de Balke. A estimativa do VO _2_ máximo (VO _2_ max) é calculada pela fórmula: VO _2_ max= (12 × carga em watts) + 300/peso em kg. 

 O protocolo de rampa tem sido mais utilizado em atletas e no TCPE. O VO _2_ max também pode ser calculado por fórmulas, sendo a mais utilizada a do ACMS, ^313^ em que: 

${VO}_{2}\max(mL/kg/min)=10,8\times W+7/\text{ peso }(kg)$

Nessa equação, o VO^2^max relativo ao peso corporal foi estimado por:

${VO}_{2}\max(mL/kg/min)=(W\times 11,4+260+\text{ peso }\times 3,5)/\text{ peso }$

## 1.4.2. Protocolos para Esteira Ergométrica

### 1.4.2.1. Protocolos Escalonados

**1.4.2.1.1. Protocolo de Bruce ^4^ ^,^ ^13^ ^,^ ^134^**

 É o protocolo escalonado mais utilizado. É recomendado para adultos e também para idosos sem limitação física e com algum grau de condicionamento físico. O cálculo da estimativa do VO _2_ max pode ser feito pelas fórmulas: ^13^ ^,^ ^134^ ^,^ ^293^

${VO}_{2}\max=(2,9\times$

 Envolve incrementos abruptos e, em indivíduos sedentários, a mudança de estágio pode provocar desequilíbrios e dificuldades de adaptação. Em atletas, geralmente, os incrementos de carga são pequenos, tornando o exame demasiadamente prolongado. 

**1.4.2.1.2. Protocolo de Bruce Modificado ^13^ ^,^ ^134^**

 Trata-se de uma adaptação do protocolo de Bruce objetivando atender a pacientes adultos e idosos com baixa capacidade física. A adaptação mais conhecida é a sugerida por Shefield ( Tabela 25 ), em que os dois primeiros estágios são de baixa carga e, a partir do terceiro estágio, que corresponde ao primeiro estágio do protocolo de Bruce, segue-se o protocolo original (com incrementos de grandes cargas ao final de cada estágio). 

**Tabela 25:** – Principais protocolos escalonados para esteira rolante e suas características 6,13,134,293

	Bruce				Bruce modificado/Sheffield				Ellestad				Naugthon			
				
**Estágio**	**Min.**	**mph/km/h**	**E%**	**MET**	**Min.**	**mph/km/h**	**E%**	**MET**	**Min.**	**mph/km/h**	**E%**	**MET**	**Min.**	**mph/km/h**	**E%**	**MET**
01	3	1,7/2,7	10	4,6	3	1,7/2,7	0	1,7	3	1,7/2,7	10	4,6	2	1,0/1,6	0	1,5
02	6	2,5/4,0	12	7,1	6	1,7/2,7	5	2,9	5	3,0/4,8	10	7,4	4	2,0/3,2	0	2,0
03	9	3,4/5,5	14	9,6	9	1,7/2,7	10	4,1	7	4,0/6,4	10	9,6	6	2,0/3,2	3,5	3,0
04	12	4,2/6,7	16	12,0	12	2,5/4,0	12	6,7	10	5,0/8,0	10	12,0	8	2,0/3,2	7,0	4,0
05	15	5,0/8,0	18	14,5	15	3,4/5,5	14	10,0	12	6,0/9,7	15	14,5	10	2,0/3,2	10,5	5,0
06	18	5,5/8,8	20	16,8	18	4,2/6,7	16	13,5	14	7,0/9,6	15	17,0	12	2,0/3,2	14,0	6,0
07	21	6,0/9,7	22	19,3	21	5,0/8,0	18	17,5	16	8,0/11,2	15	19,0	14	2,0/3,2	17,5	7,0
08	24	6,5/10,5	24	22,4	24	5,5/8,8	20	20,0	18	9,0/12,8	15	21,5	16	2,0/3,2	21,0	8,0
* Min.: minutos; mph: milhas por hora; km/h: quilômetros por hora; E%: elevação/inclinação da esteira (em %); MET: metabolic equivalent of task (equivalente metabólico da tarefa). *																

**1.4.2.1.3. Protocolo de Ellestad ^13^ ^,^ ^134^**

 Emprega aumentos expressivos de velocidade com inclinação fixa até o 4º estágio. Indicado preferencialmente para jovens, adultos fisicamente ativos e idosos que tenham o hábito de correr. 

 As limitações desse protocolo consistem em: precocemente, iniciam-se altas velocidades, dificultando adaptação (de quem não está acostumado a correr), e isso pode dificultar as medições pressóricas. 

**1.4.2.1.4. Protocolo de Naughton ^13^ ^,^ ^134^ ^,^ ^293^**

 Envolve pequenos incrementos de carga equivalentes a 1 MET por estágio. Indicado preferencialmente para idosos sedentários e indivíduos com limitações físicas, baixa capacidade física, insuficiência cardíaca compensada, infarto agudo do miocárdio recente e doença arterial periférica de membros inferiores. 

 Esse protocolo não deve ser utilizado em pacientes ativos, por prolongar demasiadamente o exame, dificultando atingir o esforço máximo. 

**1.4.2.2. Protocolo em Rampa ^4^ ^,^ ^6^ ^,^ ^13^**

 O protocolo em rampa pode ser totalmente individualizado quanto à velocidade e inclinação (iniciais e finais) e duração do exame. Permite a melhor avaliação da aptidão cardiorrespiratória (capacidade aeróbica). 

 Para definição do limite máximo do esforço suportado pelo paciente, sugere-se utilizar: escala de atividade física diária; questionário de estimava da capacidade funcional máxima (escala de atividade de Duke ou o questionário *Veterans Specific Activity Questionaire* [VSAQ]). Caso ocorra a sub ou a superestimativa desse limite, pode-se ajustar durante o exame a carga a ser atingida, de modo a manter a meta de duração de 8 a 12 minutos. ^13^ ^,^ ^134^ ^,^ ^314^

 A taxa de incremento de carga depende das velocidades e inclinações iniciais e finais programadas. Em indivíduos sedentários ou com limitações, sugere-se iniciar o TE em baixa velocidade (1,6 a 2,7 km/h) e pouca inclinação (de 0% a 5%). Em indivíduos ativos, sugere-se iniciar o TE em velocidade de 2,7 a 4,0 km/h (de 0% a 5%). ^13^ ^,^ ^315^ ^,^ ^316^

 Para o cálculo do VO _2_ , são sugeridas as fórmulas de Foster: ^317^

${VO}_{2}=0,694\times {VO}_{2}\text{ do }ACSM+3,33$ ${VO}_{2}=0,869\times {VO}_{2}\text{ do ACSM - 0,07 }$

### 1.4.3. Protocolo para Ergômetro de Braços

 Habitualmente, o incremento da carga deve ser a metade do utilizado nos protocolos para os membros inferiores. Balady et al. desenvolveram protocolo com carga inicial de 10W, acréscimo de 10W a cada 2 minutos e manutenção de velocidade constante entre 75 e 80rpm. ^318^

 Mitropoulos et al. desenvolveram protocolo com carga inicial de 30W para homens e 20W para mulheres, acréscimo em rampa linear (de 10W/min para homens e 6W/min para mulheres) e um ritmo de 70rpm. ^319^

### 1.4.4. Interrupção/Término do Exame

 A interrupção parcial do esforço durante o TE/TCPE pode ser feita em casos excepcionais com desaceleração ou mesmo parada do ergômetro brevemente: diante da necessidade de confirmação de níveis pressóricos; ajustes de posicionamento de eletrodos; verificação de ausculta cardiopulmonar; vertigem (para tentativa de readaptação) etc. 

 Após a fase de esforço, sugere-se realizar recuperação ativa por pelo menos 1 a 3 minutos: 

 – No cicloergômetro, realizar desaceleração gradual da carga. 

 – Na esteira ergométrica, realizar inicialmente desaceleração lenta e gradual (da velocidade e inclinação), seguida de manutenção de caminhada a uma velocidade de 1,5 MPH ou 2,4km/h e 2,5% de inclinação. ^13^ ^,^ ^152^

 Independentemente do ergômetro e protocolo, após interromper completamente o esforço, deve-se manter o paciente sentado até o retorno próximo à sua condição basal (pelo menos até o sexto minuto). Em casos de sintomas e/ou incapacidade de realização de recuperação ativa, sugere-se realizar interrupção rápida da velocidade e colocação do paciente em decúbito dorsal. 

## 2. Acurácia, Probabilidade e Escores Pré-teste

 A acurácia diagnóstica do TE/TCPE varia de acordo com a doença investigada e sua prevalência, características clínicas do paciente, idade e sexo. Com base nessas informações, podemos selecionar os pacientes que mais se beneficiarão do TE/TCPE para diagnóstico, evitando investigações e intervenções desnecessárias. ^277^

### 2.1. Probabilidade Pré-teste de DAC

 A probabilidade da existência de DAC varia com idade, sexo, características dos sintomas, fatores de risco e estilo de vida. Para definir a probabilidade, recomenda-se utilizar uma das tabelas de estimativa de risco baseadas na classificação de dor precordial por sexo e idade (Tabelas 26 a 28). Ressalta-se que a frequência de DAC aumenta com a idade. 

 O TE é mais útil para o diagnóstico de DAC em pacientes com probabilidade pré-teste intermediária (definida como de 10% a 90% na Tabela 26 , ou entre 25% a 75% na Tabela 28 ), pois o resultado terá maior impacto na decisão clínica. ^6^ ^,^ ^320^

**Tabela 26:** – Diamond-Forrester and CASS – Estimativa de risco para probabilidade pré-teste de DAC obstrutiva de acordo com classificação de angina 321

Idade (anos)	Sem angina		Angina atípica		Angina típica	
	Homens	Mulheres	Homens	Mulheres	Homens	Mulheres
30 a 39	4	2	34	12	76	26
40 a 49	13	3	51	22	87	55
50 a 59	20	7	65	31	93	73
60 a 69	27	14	72	51	94	86

**Tabela 28:** – *European database* – Estimativa de risco para probabilidade pré-teste de DAC obstrutiva em pacientes sintomáticos 45

Idade (anos)	Sem angina		Angina atípica		Angina típica	
	Homens	Mulheres	Homens	Mulheres	Homens	Mulheres
30 a 39	18	5	29	10	59	28
40 a 49	25	8	38	14	69	37
50 a 59	34	12	49	20	77	47
60 a 69	44	17	59	28	84	58
70 a 79	54	24	69	37	89	68
>80	65	32	78	47	93	76
* Probabilidade de DAC estimada para pacientes com idade 35, 45, 55, 65, 75 e 85 anos. *						

###  2.2. Sensibilidade, Especificidade e Valor Preditivo 

**Figura 3:**
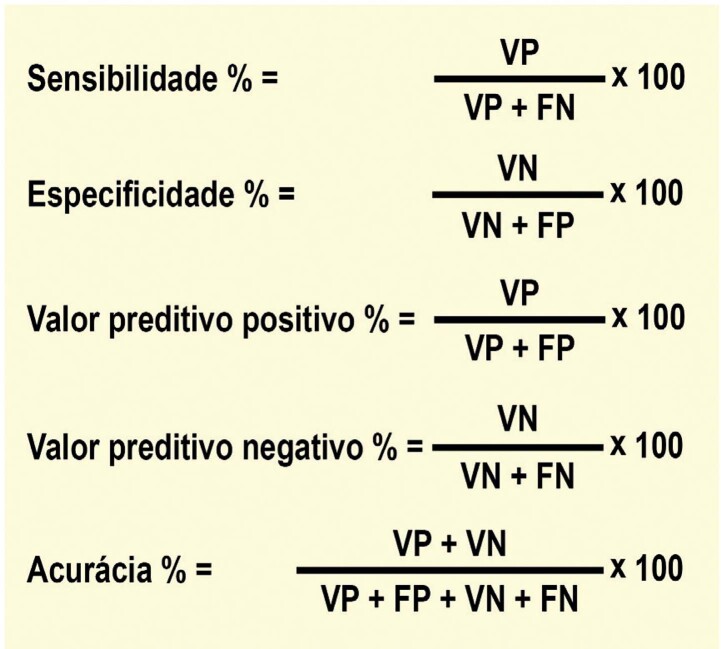
– Definição de sensibilidade, especificidade, valores preditivos e acurácia. VP: verdadeiro positivo – quando o exame se apresenta positivo e o paciente tem a doença investigada; FP: falso-positivo – quando o exame se apresenta positivo em paciente sem doença; VN: verdadeiro negativo – quando o exame se apresenta negativo e o paciente não tem doença; FN: falso-negativo – quando o exame se apresenta negativo, mas o paciente tem a doença investigada.

 O TE pode ser avaliado sob características de desempenho dos testes diagnósticos baseados em cálculos operacionais de sensibilidade, especificidade, valor preditivo (positivo e negativo) e acurácia ( Figura 3 ). 

 Em relação ao TE na investigação de DAC, a maioria dos estudos realizados demonstra sensibilidade entre 61% e 73% (média de 69%) e especificidade entre 69% e 81% (média de 75%). Ressalta-se que as variações nesses valores estão associadas às diferenças nas metodologias e populações estudadas. ^6^ ^,^ ^323^

 O valor preditivo positivo do TE é sempre maior em homens, em todas as faixas etárias, devido à alta prevalência de DAC. ^324^

 As mulheres, em geral, apresentam menor prevalência de DAC e, portanto, maior presença de testes falso-positivos em relação aos homens. ^325^ Nas mulheres, a idade tem maior influência no VPP, pois ele é mais baixo nas mais jovens (35 a 50 anos = 36%) em comparação às idosas (>65 anos = 68%). ^326^

 No estudo de coorte comparando TE em ambos os sexos, nas mulheres, a utilização da FC máxima, a duração do esforço e o tempo de recuperação de infradesnivelamento do ST permitiram aumento significativo do VPP (aumentando de 47,8 para 61,5%) e VPN (88%) do TE. ^327^

### 2.3. Escores e Fatores de Risco DCV Pré-teste

 A utilização de escores de risco para doenças cardiovasculares pré-teste visa propiciar uma abordagem individualizada, previsão de possíveis complicações (principalmente em pacientes de risco intermediário e elevado) e, consequentemente, uma análise mais contextualizada dos achados do exame. Essa avaliação é recomendada em adultos e idosos (faixa etária típica de 40-75 anos), devendo ser baseada em um dos escores: *European Society of Cardiology Systematic Coronary Risk Evaluation* (SCORE2), algoritmos do *American College of Cardiology/American Heart Association Atherosclerotic Cardiovascular Disease* (ASCVD) ou Escore de Risco Global de Framingham ( *Framingham Risk Score* ). ^328^ ^-^ ^330^

 Nos adultos jovens (<40 anos), sugere-se a avaliação e os registros dos fatores de risco clássicos: diabetes, tabagismo, dislipidemia, estresse, sedentarismo, obesidade, HAS, história familiar. ^331^

##  3. Respostas Clínicas e Hemodinâmicas ao Esforço na População Adulta 

### 3.1. Respostas Clínicas

#### 3.1.1. Tolerância ao Esforço

 A tolerância ao esforço é reconhecida como o melhor marcador atual de expectativa de vida, independentemente de idade, sexo, etnia e condições associadas. Durante um TE, pode ser quantificada de forma objetiva pela potência desenvolvida em watts, pela duração do exercício ou pelo equivalente metabólico (em METs). ^332^

**Figura 4:**
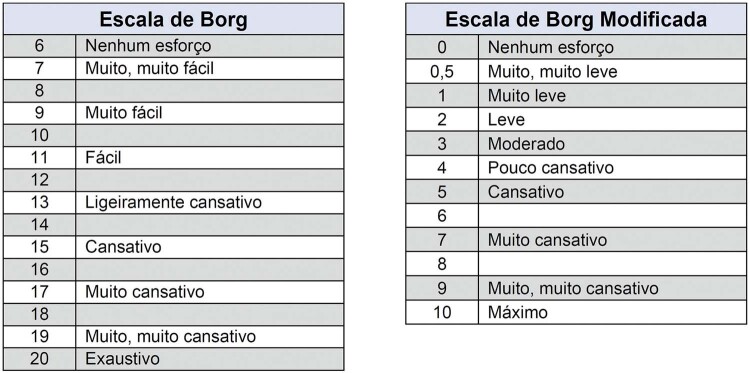
– Escalas de esforço percebido de Borg e Borg modificada.

 A tolerância ao esforço pode ser quantificada subjetivamente por meio da escala de esforço percebido de Borg ou de Borg modificada ( Figura 4 ) visando medir o nível de intensidade da atividade física, o grau de cansaço, a gravidade de dispneia aos esforços e também a fadiga dos membros inferiores. ^333^ ^,^ ^334^

 Independentemente da presença de DCV, a baixa tolerância ao esforço se relacionada a taxas mais elevadas de mortalidade e aumento da incidência de IC e DAC. ^332^

 A intolerância ao exercício (IE) é definida como a capacidade prejudicada de realizar atividade física na presença de sintomas, como dispneia e/ou fadiga. ^335^

 Tanto na ICFEr quanto na insuficiência cardíaca com fração de ejeção preservada (ICFEp), a IE associa-se a pior qualidade de vida, hospitalizações mais frequentes e aumento da mortalidade por todas as causas. ^116^ ^,^ ^336^ Na insuficiência cardíaca diastólica crônica, um dos principais mecanismos de IE é a incompetência cronotrópica que pode ser adequadamente avaliada ao TE. ^337^

 No diabetes *mellitus* tipo 2 (DM2), a IE também está associada à incompetência cronotrópica, sendo ambas associadas a risco elevado de DCV e morte prematura. ^338^

**Figura 5:**
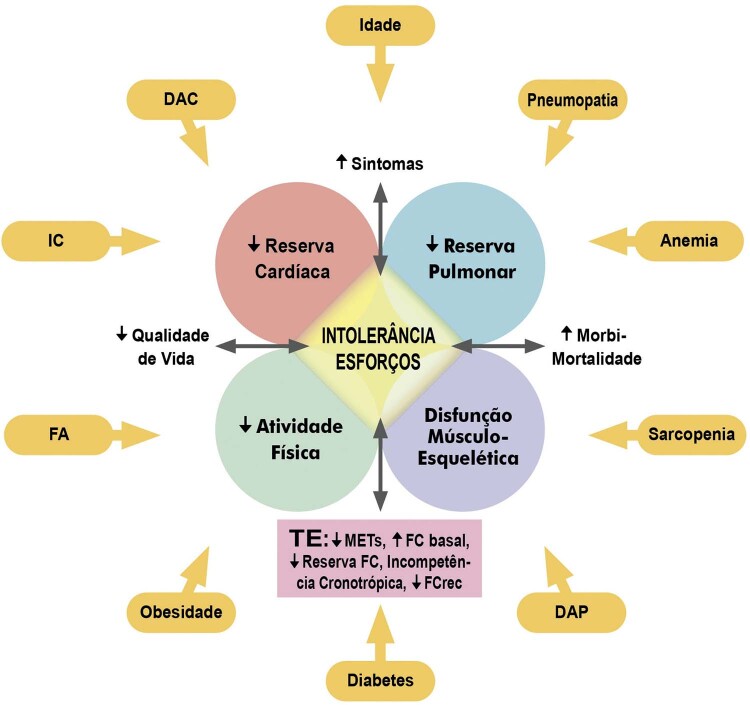
– Mecanismos e fatores associados ao surgimento e progressão da intolerância ao esforço e papel do TE. DAC: doença arterial coronariana; IC: insuficiência cardíaca; FA: fibrilação atrial; DAP: doença arterial periférica; TE: teste ergométrico; FC: frequência cardíaca; ↓ METs: baixa aptidão cardiorrespiratória; ↑ FC basal: frequência cardíaca basal elevada; ↓ Reserva FC: redução da reserva cronotrópica; ↓ FCrec: recuperação lenta da FC pós-esforço.

 A Figura 5 apresenta os principais mecanismos e fatores que contribuem para o surgimento e a progressão da IE. 

 TE/TCPE são métodos imprescindíveis para diagnóstico da IE e monitoração de programas de treinamento físico, visando documentar a melhora da tolerância ao exercício, do desempenho cardiopulmonar e consequente redução da morbimortalidade. ^339^ ^,^ ^340^ O programa de treinamento físico parece ser uma das únicas intervenções potencialmente eficazes e viáveis para a melhora da IE. ^338^ ^,^ ^340^

####  3.1.2. Aptidão Cardiorrespiratória/Classificação Funcional 

 A aptidão cardiorrespiratória (ACR)/classificação funcional no TE é uma estratificação de desempenho físico baseada no consumo de oxigênio (VO _2_ ). 

 O consumo máximo de oxigênio (VO _2_ max) expressa a maior quantidade de oxigênio extraído do ar inspirado durante realização de exercício dinâmico que envolva grande massa muscular. Nos TE que não houver as características de um esforço máximo, o VO _2_ obtido deve ser denominado VO _2_ pico. ^13^ ^,^ ^134^ ^,^ ^293^

 O TE é considerado de esforço máximo quando: ^4^

– Ocorrerem sinais ou sintomas de exaustão física.

– Incapacidade de prosseguir o esforço.

– Escala de Borg (≥18).

 – FC não se eleva mesmo com aumentos da intensidade do esforço. 

 – Atingir a FCmax prevista (sempre levar em consideração os itens anteriores) ou exceder a FCmax prevista (≥110%). 

 No TE, o VO _2_ é estimado (medida indireta) por meio de fórmulas, enquanto, no TCPE, é mensurado diretamente. A medida indireta costuma superestimar os valores de VO _2_ devido às limitações dos estudos que geraram as fórmulas. ^341^ ^,^ ^342^

 Existem várias fórmulas (clássicas e novas) para o cálculo de VO _2_ pico previsto baseadas no tipo de ergômetro, por sexo, protocolo, se mãos apoiadas nas barras etc. ( Anexo 2 ). ^293^ ^,^ ^343^ ^-^ ^348^

 O VO _2_ é mais frequentemente expresso em mL/kg/min (também aceitável mL.kg ^-^ ^1^ .min ^-^ ^1^ ). Também pode ser expresso por meio do equivalente metabólico (MET), e cada MET corresponde a 3,5 mL/kg/min de VO _2_ . ^6^


$$MET=\frac{{VO}_{2}mL/kg/min}{3,5mL/kg/min}$$

Na população em geral, o VO _2_ mensurado no cicloergômetro costuma ser menor do que na esteira. O VO _2_ max apresenta declínio progressivo a partir dos 30 anos, sendo, em média, de 8% a 10% menor por década. Aos 60 anos, a média de VO _2_ max em homens é aproximadamente dois terços daquela de 20 anos. Quando comparadas aos homens, as mulheres costumam apresentar menor VO _2_ max devido a valores menores de hemoglobina, volume sanguíneo, volume sistólico e massa muscular. ^13^ ^,^ ^134^

**Figura 6:**
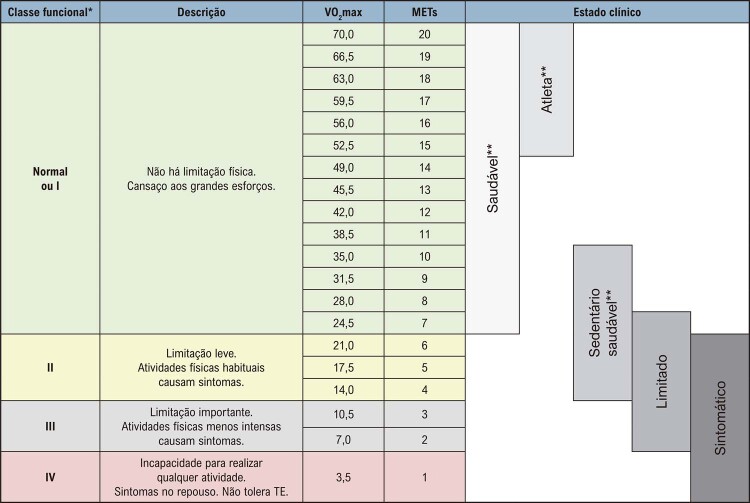
– Valores de VO 2 /METs em relação à classe funcional e estado clínico. 6,232 VO 2 max: consumo máximo de oxigênio; TE: teste ergométrico; MET: metabolic equivalent of task (equivalente metabólico da tarefa). *New York Heart Association. **Dependendo da idade e do nível de atividade física.

 A classificação funcional ( *New York Heart Association* ) é utilizada no diagnóstico e no prognóstico, contribuindo para condutas terapêuticas e prescrição de exercícios, estando relacionada ao VO _2_ /METs alcançados no TE ( Figura 6 ). ^6^ ^,^ ^322^

#### 3.1.3. Sintomas

 Os sintomas durante o TE devem ser descritos minuciosamente e correlacionados com sinais clínicos, resposta hemodinâmica e achados do ECG. Os sintomas que levarem à interrupção da fase de exercício devem ser mencionados com o respectivo grau de percepção do esforço (pela escala de Borg ou Borg modificada) e o desempenho atingido (carga de esforço/METs). 

**Figura 7:**
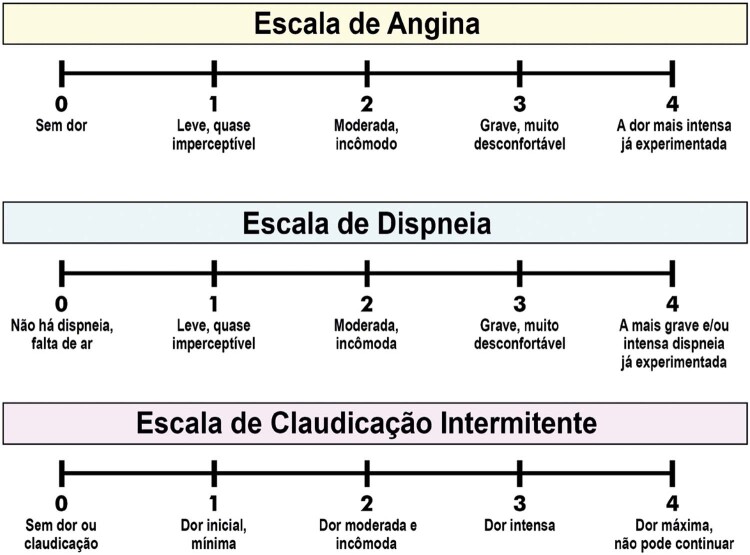
– Escalas para quantificação de angina, dispneia e claudicação intermitente.

 Sugere-se a utilização de escalas para quantificar os sintomas de angina, dispneia e claudicação intermitente, principalmente durante o TCPE. As escalas devem estar visíveis e serem claramente explicadas antes do início do exame ( Figura 7 ). ^13^ ^,^ ^292^ ^,^ ^349^ ^,^ ^350^

 No caso de ocorrência de dor torácica, recomenda-se descrever seu caráter, localização, irradiação, fatores de agravamento e alívio, duração, demais sintomas concomitantes e o momento do TE em que ela iniciou e terminou. Recomenda-se que se descreva também se a dor foi limitante ou não, a FC, a PAS e duplo-produto iniciais e respectivos achados do ECG. 

 A ocorrência de angina típica, progressiva com o incremento das cargas, por si só, é considerada como compatível com resposta isquêmica. ^6^

 Estudo com 10.870 pacientes submetido a TE limitado por sintomas, a angina *pectoris* típica foi associada a risco aumentado de mortalidade (RR: 2,7; IC 95%: 1,4-5,1; p<0,002) em comparação com dor torácica não anginosa. ^351^

 A ocorrência da dispneia no TE está associada a um aumento significativo na mortalidade por todas as causas, mas não apresenta maiores taxas de isquemia em comparação com os pacientes com dor torácica. ^352^

 Na suspeita de DAP e avaliação de claudicação intermitente, recomenda-se utilizar a escala de dor de claudicação intermitente para quantificar a dor e sua gravidade. Idealmente, o TE deve levar o paciente à dor máxima tolerada e/ou até o momento em que o paciente não puder mais andar (claudicação absoluta). ^353^

 A escala de claudicação intermitente varia de 0 a 4 ( Figura 7 ) e permite identificar a progressão da claudicação durante o TE. Recomenda-se registrar a respectiva carga de esforço, o tempo de exercício e o momento de alívio da dor. ^175^

 Todos os outros sintomas (p. ex., vertigem, tontura, lipotimia, cefaleia) devem ser relatados quanto ao momento de ocorrência, intensidade, duração, relação com PA e FC, bem como outras informações que possibilitem a adequada interpretação. 

#### 3.1.4. Ectoscopia/Ausculta

 A ocorrência de sudorese e rubor facial é esperada durante esforço físico intenso. A presença de palidez cutânea, acompanhada de sudorese excessiva, cianose ou taquipneia, denota condição patológica. A ocorrência em baixas cargas de esforço, associadas a alterações hemodinâmicas ou modificações eletrocardiográficas (infradesnivelamento ou supradesnivelamento de ST e arritmias complexas), indica maior gravidade. ^1^

 É necessário comparar a ausculta antes e após o esforço, de modo a correlacioná-la aos sinais clínicos observados. A presença de sibilos, roncos ou estertores crepitantes antes do esforço podem contraindicar a realização do TE e, após o esforço, pode significar asma esforço-induzida ou disfunção ventricular. ^354^ ^,^ ^355^

 Na ausculta de novos sopros e/ou agravamento de sopros preexistentes (no esforço ou recuperação), deve-se descrever sua relação com o ciclo cardíaco, localização, qualidade ou tom, intensidade e presença de cliques de ejeção. ^356^ ^,^ ^357^

 O esforço costuma aumentar a intensidade dos sopros regurgitantes originados das câmaras cardíacas esquerdas (regurgitação mitral e aórtica). ^92^ ^,^ ^94^

 O surgimento de B3 ao esforço em homens acima dos 40 anos, na presença de dispneia ou fadiga excessiva, pode estar associado à disfunção ventricular. ^358^ Em adultos e idosos, o surgimento de B3 logo após o término do exercício frequentemente está associado a disfunção ventricular, infarto prévio e bloqueio de ramo esquerdo. ^359^

 Adultos e atletas jovens costumam apresentar B3 na ausculta basal. Caso surja durante o exercício, é considerada uma adaptação fisiológica, sem correlação com cardiopatia estrutural. ^360^

## 3.2. Respostas Hemodinâmicas

 Diante do incremento do esforço físico no TE, conceitua-se como: ^13^ ^,^ ^134^ ^,^ ^293^

 – Reserva dromotrópica: aumento da velocidade da condução dos estímulos elétricos cardíacos. 

 – Reserva inotrópica: capacidade de aumento da função ventricular (eficiência do enchimento e do esvaziamento dos ventrículos) sendo avaliada pelo comportamento da PA sistólica. 

 – Resistência arterial periférica: capacidade de adaptação dos vasos periféricos (vasodilatação/vasoconstrição), sendo avaliada pelo comportamento da PA diastólica. 

 – Reserva coronária: capacidade da rede coronária adequar-se ao maior fluxo de sangue devido ao aumento da atividade metabólica do miocárdio. 

 – Reserva cardíaca: capacidade do coração em elevar seu débito para compensar a maior demanda metabólica da musculatura em exercício, sendo influenciada por todos os parâmetros anteriores. 

### 3.2.1. Frequência Cardíaca

 O comportamento da FC permite avaliar a resposta cronotrópica, a reserva cronotrópica, o DP, a regulação autonômica, sendo importante parâmetro diagnóstico e prognóstico. 

**3.2.1.1. Frequência Cardíaca de Repouso**

 A faixa de normalidade da FC de repouso (FCR) é de 50 bpm a 99 bpm e verificada a partir do ECG em repouso (sentado ou em decúbito dorsal). A FCR ao TE deve ser valorizada para a interpretação do comportamento da FC, regulação autonômica e definição de prognóstico. ^361^ ^,^ ^362^

 É preditora de DAC, IC, FA, AVC e está associada a risco aumentado de eventos cardiovasculares, morte cardíaca e morte por todas as causas. ^363^ ^-^ ^365^

 Tem sido demonstrado que, no TE, a FCR ≥80 bpm na população geral e a FCR ≥75 bpm em diabéticos com DAC estável estão relacionadas a aumento de mortalidade por todas as causas. ^366^

 Em coorte de 56.634 indivíduos (49% mulheres) sem DAC conhecida ou FA, FCR ≥90 bpm apresentou aumento significativo de mortalidade por todas as causas, sendo que em homens foi independente da aptidão física. ^367^

**3.2.1.2. Resposta Cronotrópica**

 A resposta cronotrópica normal ao exercício consiste no aumento da FC devido à diminuição do tônus vagal, seguido 

 por aumento do tônus simpático e consequentes adaptações do fluxo sanguíneo vascular sistêmico. ^368^

 O incremento da FC acompanha o aumento das cargas de esforço e costuma apresentar correlação linear somente com o VO _2_ entre 50 e 90% do VO _2_ max. Em adultos saudáveis, a FC normalmente aumenta a uma taxa de ≈10 bpm por MET. ^134^

**Figura 8:**
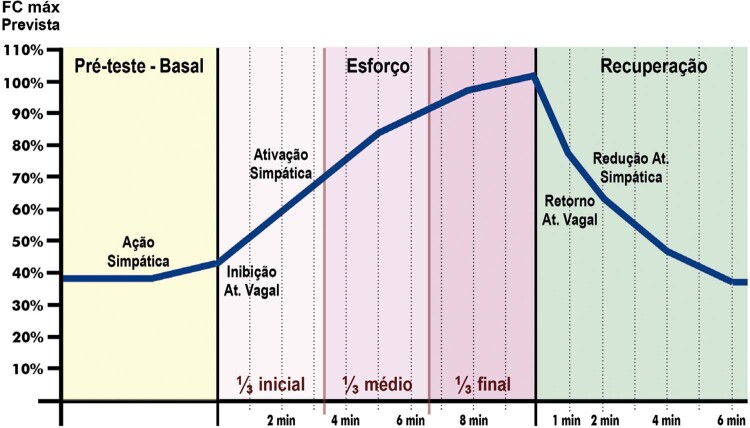
– Comportamento da frequência cardíaca e ajustes autonômicos durante o TE em adultos. FCmax: frequência cardíaca máxima; At.: atividade; min: minuto.

 A retirada vagal é responsável pelo aumento inicial dos 10 a 30 batimentos, sendo o restante geralmente mediado pela atividade simpática. A FC é responsável pela maior parte do aumento do débito cardíaco durante o esforço, particularmente em cargas mais elevadas ( Figura 8 ). ^134^ ^,^ ^299^

 A recuperação da FC (FCrec) durante os primeiros 30 a 60 segundos após o esforço envolve a reativação do sistema parassimpático e progressiva inibição da atividade simpática. A FCrec também pode ser influenciada pelo grau de retorno venoso e resposta dos barorreceptores atriais. O retardo na FCrec é um importante marcador de disfunção autonômica e de risco de mortalidade cardiovascular (CV) e por todas as causas e eventos cardiovasculares maiores. ^369^

 A frequência cardíaca máxima estimada (FCME) não deve ser usada como único critério de interrupção do esforço ou para avaliar a eficácia do TE. De modo geral, existe uma margem de erro (grande dispersão da média) nas fórmulas, variando em ±10 a 15 bpm. O sexo feminino costuma apresentar valores menores de FCME. ^301^ ^,^ ^370^

 A avaliação da resposta cronotrópica através da FCME é limitada na vigência de medicações que interferem na modulação autonômica (antiarrítmicos e betabloqueadores), na FA e flutter atrial, marca-passo com sensores, CDI, BAVT congênito etc. Os principais fatores que interferem na FC máxima atingida no TE encontram-se na Tabela 29 . 

**Tabela 29:** – Fatores que afetam a FC máxima em resposta ao exercício dinâmico

Fatores que afetam a FC máxima	

Idade	Doença cardiovascular
Gênero	Medicamentos
Peso corporal	Marca-passo/CDI
Repouso prolongado	Arritmias/fibrilação e * flutter* atrial
Tipo de exercício	Anemia
Intensidade do esforço atingido	Hipo e hipertireoidismo
*CDI: cardiodesfibrilador implantável.*	

 A Tabela 30 apresenta as variáveis referentes ao comportamento da FC ao TE, enquanto a Tabela 31 apresenta suas definições, critérios e interpretações. 

**Tabela 30:** – Variáveis referentes ao comportamento da FC ao TE

Índice	Cálculo	Valores Normalidade
** FC repouso ^**371**^**	Verificada no ECG repouso (sentado ou decúbito dorsal)	50 bpm a 99 bpm
** FC máxima estimada para idade ^**300-302**^**	– Equações predição da FCmax para ambos os sexos:** FCmax = 220 - idade FCmax = 208 - (0,7 × idade) – Equações predição da FCmax específicas:** FCmax para mulheres = 192 - (0,7 × idade) FCmax para homens = 201 - (0,6 × idade)	≥85% da FCmax estimada
** Reserva cronotrópica medida ^**372,373**^**	RFC = FCmax atingida - FC repouso	Avaliação seriada
**Reserva cronotrópica prevista para a idade**	RFC idade = FCmax estimada para idade – FC repouso	Avaliação seriada
**Índice cronotrópico (%)**	ICr = (FCmax atingida - FC repouso) × 100 (FCmax estimada - FC repouso)	≥80%
FC 1º minuto recuperação ativa*	FCmax atingida - FC recuperação 1 **º** minuto	>12 bpm
**FC 1º minuto recuperação passiva (deitado)**	FCmax atingida - FC recuperação 1 **º** minuto	>18 bpm
**FC 2º minuto recuperação passiva (sentado)**	FCmax atingida - FC recuperação 2 **º** minuto	≥22 bpm
FC: frequência cardíaca; FCmax: frequência cardíaca máxima; ICr: índice cronotrópico; RFC: reserva cronotrópica; *Recuperação ativa em esteira: caminhada na velocidade de 1,5 MPH ou 2,4 km/h e 2,5% de inclinação. **Idade em anos.		

**Tabela 31:** – Definições, critérios e interpretações do comportamento da FC ao TE

Termo	Critérios*	Interpretações
**Comportamento da FC no ECG basal**		
**Comportamento normal da FC**	ECG de repouso (sentado ou em decúbito dorsal) com FC de 50 bpm a 99 bpm.	Adulto em ritmo sinusal.
**Bradicardia sinusal em repouso**	ECG de repouso (sentado ou em decúbito dorsal) com FC <50 bpm.	Comum em atletas e jovens vagotônicos, assintomáticos. Caso secundária à utilização de betabloqueador ou antiarrítmico, referir essa interferência no laudo. Em pacientes que não utilizam medicações inotrópicas negativas, avaliar possibilidade de doença do nó sinusal ou outras causas secundárias. Afastar BAV de segundo grau e BAV avançado.
**Taquicardia sinusal em repouso**	ECG de repouso (sentado ou em decúbito dorsal) com FC ≥100 bpm.	Usualmente encontrada em pacientes obesos, com elevado grau de ansiedade, no hipertireoidismo, na anemia e após ingestão excessiva de cafeína ou álcool.
**Comportamento da FC ao esforço**		
**Resposta cronotrópica normal**	Atingir ≥85% da FCmax estimada entre 8 e 12 minutos de esforço.	Quando em ritmo sinusal.
**Resposta cronotrópica acentuada **	1) Elevação exacerbada da FC, desproporcional à carga de trabalho, atingindo FC ≥85% da FCmax prevista ou ≥50% de aumento da FC de repouso aos 3,5 METs de esforço, ou 2) Aumento ≥17 bpm no primeiro minuto do esforço em indivíduos normais, ou 3) Aumento ≥15 bpm no primeiro minuto do esforço em coronariopatas.	Usualmente encontrada em sedentários, pacientes com elevado grau de ansiedade, na distonia neurovegetativa, no hipertireoidismo, nas condições que reduzem o volume vascular ou a resistência periférica, na anemia, nas alterações metabólicas, em TE precoces após infarto e/ou cirurgia de revascularização etc. ^374^
**Queda da FC intraesforço**	Queda da FC com a progressão do esforço (>10 bpm).	Apesar de rara, apresenta alta correlação com doença isquêmica. ^152^
**Reserva cronotrópica **	Este parâmetro deve ser avaliado em TE seriados. Quanto maior a reserva, melhor o estado funcional, o tônus vagal/modulação autonômica e a saúde cardiovascular.	Redução da RFC é fator de risco para mortalidade por DCV. ^375^ Cada incremento de 1 batimento/min na RFC reduz a incidência de MSC em 1-2% e a incidência de diabetes tipo 2 em 2-3%. ^373^ ^,^ ^376^
** Resposta cronotrópica deprimida ou incompetência cronotrópica **	1) A FC atingida no esforço < que dois desvios padrões da FC máxima prevista, ou 2) Não atingir 85% da FC prevista para idade, ou 3) Índice cronotrópico inferior a 0,80.	Relacionada à diminuição da atividade vagal e associada a risco de mortalidade cardiovascular e por todas as causas. ^149^ ^,^ ^151^ ^,^ ^377^ ^,^ ^378^
**Platô da FC intraesforço**	Manutenção da FC mesmo com a progressão do esforço.	Pode ocorrer em mulheres e crianças, sem significado clínico. Pode ocorrer em pacientes com DAC. ^134^ ^,^ ^152^
**Comportamento da FC na recuperação**		
**Comportamento normal da FC na recuperação**	1) FC 1 **º** minuto recuperação ativa (>12 bpm), ou 2) FC 1 **º** minuto recuperação passiva (deitado; >18 bpm), ou 3) FC 2 **º** minuto recuperação passiva (sentado; ≥22 bpm).	Quando em ritmo sinusal.
**Recuperação lenta da FC (pós-esforço)**	1) FC 1 **º** minuto recuperação ativa (≤12 bpm), ou 2) FC 1 **º** minuto recuperação passiva (deitado; ≤18 bpm), ou 3) FC 2 **º** minuto recuperação passiva (sentado; ≤21 bpm).	A recuperação lenta da FC está associada à mortalidade cardiovascular e por todas as causas. ^38^
**Queda súbita e acentuada da FC na recuperação**	Queda abrupta da FC em qualquer momento da recuperação.	Achado comum em indivíduos bem condicionados fisicamente, incluindo atletas, sendo considerada normal desde que assintomática. ^13^
FC: frequência cardíaca; FCmax: frequência cardíaca máxima; MSC: morte súbita cardíaca; RFC: reserva cronotrópica; DAC: doença arterial coronariana; DCV: doença cardiovascular; BAV: bloqueio atrioventricular. *Descrever o uso de medicamentos que possam afetar o comportamento da FC.		

Particularidades da resposta cronotrópica:

 – Coorte em 458 homens (idade 56±8,5; seguimento médio: 6 anos) com DAC demonstrou que o aumento da FC durante o primeiro minuto do esforço no TE ≥12 bpm foi fortemente relacionada à morte cardíaca (RR: 15,6; IC 95%: 2,0-118,7; p<0,001) e IAM não fatal (RR: 5,0; IC 95%: 2,7-9,1; p<0,0001). ^379^

 – Em seguimento de 306 pacientes com estenose aórtica assintomática (idade 65±12 anos, 33% mulheres; seguimento médio de 25 meses), nos casos graves, a elevação rápida e precoce da FC (definida como atingir 85% FC máxima ou aumento ≥50% da FC aos 3,5 METs) associou-se à necessidade de troca valvar (RR: 3,32; IC 95%: 2,03-5,45; p<0,001). ^374^

### 3.2.2. Resposta da Pressão Arterial

 A avaliação da resposta da pressão arterial (RPA) ao esforço é um importante instrumento diagnóstico, prognóstico e de estratificação de risco cardiovascular. Basicamente, depende do débito cardíaco, da resistência vascular periférica e do uso de medicações. ^6^ ^,^ ^380^

**Figura 9:**
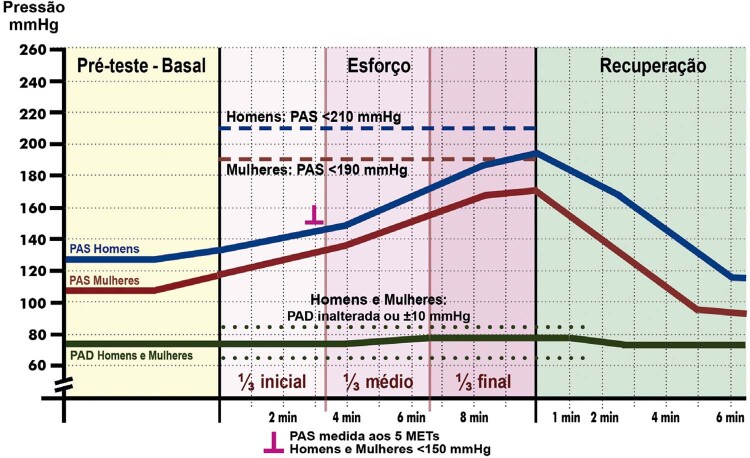
– Comportamento da pressão arterial sistólica e diastólica em adultos de ambos os sexos durante o TE. PAS: pressão arterial sistólica; PAD: pressão arterial diastólica; min: minuto.

 Em adultos saudáveis, espera-se que o aumento da pressão arterial sistólica (PAS) seja proporcional ao aumento de carga de exercício dinâmico (correspondendo à resposta inotrópica). Com a manutenção da carga de esforço, a PAS se estabiliza após 2 a 3 minutos. ^380^ ^,^ ^381^ O aumento médio da PAS geralmente é de 10 mmHg/MET. Em indivíduos que atingem carga de esforço >10 METs, deve-se considerar que a elevação da PAS seja de 6,2 mmHg/MET. ^6^ ^,^ ^382^ Após o esforço, a PAS tende a diminuir gradativamente devido à resposta vagal e rápida redução do débito cardíaco. Em geral, retorna ao padrão de repouso até os 6 minutos de recuperação, podendo ocorrer níveis inferiores aos do pré-exercício por várias horas ( Figura 9 ). ^383^

 Durante o esforço, a pressão arterial diastólica (PAD) permanece inalterada ou pode apresentar pequena oscilação (±10 mmHg) devido à queda da resistência arterial periférica. Ocasionalmente, em indivíduos saudáveis, os sons da PAD no esforço podem ser ouvidos até 0 mmHg, sem significar alteração patológica. Nesses casos, recomenda-se utilizar o som da fase IV de Korotkoff (abafamento abrupto de sons que se tornam suaves) para definir o nível de PAD. No início da recuperação, a PAD pode apresentar pequena elevação ou manter-se inalterada. Geralmente, até os 6 minutos da recuperação, tende a retornar aos valores de repouso. ^384^

 Recomenda-se não realizar o TE caso seja constatada hipertensão em repouso com PAS ≥180 e/ou PAD >110 mmHg. Nas medições com esfigmomanômetro, realizar a aproximação dos valores para 5 mmHg. Em medições com equipamentos automáticos considerar os números absolutos. ^4^ ^,^ ^6^ ^,^ ^13^

 A resposta da PAS considerada hipertensiva/exagerada ao esforço é definida como um valor máximo ≥210 mmHg para homens e ≥190 mmHg para mulheres, independentemente de protocolo e/ou ergômetro. ^85^ ^,^ ^385^ ^-^ ^387^ Em normotensos no basal, essa resposta da PAS está associada a risco maior de desenvolvimento futuro de hipertensão e eventos cardiovasculares ( Tabela 32 ). ^388^ ^,^ ^389^

**Tabela 32:** – Comportamento da pressão arterial ao TE em adultos

Termo	Critérios	Interpretação
Resposta normal da PA ao esforço e recuperação*	1) Repouso PA normal: PAS <140 mmHg e PAD <90 mmHg e 2) Esforço: PAS <210 mmHg para homens e <190 mmHg para mulheres; PAD inalterada ou oscilação de até ±10 mmHg e 3) Recuperação: queda da PAS gradativa até retorno aos padrões de repouso ou pouco inferior ao mesmo. PAD pode apresentar pequena elevação no início ou manter-se inalterada; aos 6 minutos, tende a retornar aos valores de repouso.	Normotensão em repouso, esforço e recuperação. Na ausência de outras alterações do TE, bom prognóstico e baixo risco. ^6^ ^,^ ^215^
** Hipertensão pré-teste com resposta normal da PA ao esforço **	1) PA repouso elevada: PAS ≥140 mmHg e/ou PAD ≥90 mmHg e 2) Esforço: PAS <210 mmHg para homens e <190 mmHg para mulheres; PAD inalterada ou oscilação de até ±10 mmHg e 3) Recuperação: normal.	Normalmente devido à ansiedade e sem associação ao desenvolvimento futuro de HA. ^6^ ^,^ ^215^
**Resposta hipertensiva/exagerada ao esforço**	1) A PA de repouso poderá estar normal ou elevada (PAS ≥140 mmHg e/ou PAD ≥90 mmHg) e 2) Esforço: PAS ≥210 mmHg para homens e ≥190 mmHg para mulheres; elevação de PAD ≥15 mmHg ou PAD >90 mmHg (homens e mulheres).** ^85^ ^,^ ^387^ Observação: descrever as respostas da PAS e da PAD.	Representa manutenção/agravamento de HA ao esforço. Em pacientes normotensos no repouso, a RHE está associada ao risco de HAS futura. ^71^ ^,^ ^387^ ^-^ ^389^ ^,^ ^406^ A RHE sistólica está associada a aumento do risco de HVE, IAM, FA, AVC e morte CV. ^74^ ^,^ ^405^ ^,^ ^407^ ^,^ ^408^ A RHE diastólica está associada ao aumento do risco de DAC e de HA. ^73^ ^,^ ^396^
**Hipotensão/queda da PA intraesforço**	1) Queda na PAS abaixo do valor de repouso sem queda da PAD (normalmente associada à isquemia). ^409^ 2) Queda na PAS e queda da PAD abaixo do valor de repouso (normalmente não está associada à isquemia, e sim a déficit inotrópico do VE [p. ex., valvopatia]). ^409^ 3) PAS com aumento inicial no exercício seguido por uma queda da PAS ≥20 mmHg.*** ^115^ ^,^ ^386^ ^,^ ^410^	A hipotensão esforço-induzida é marcador de eventos adversos no TE, de mau prognóstico e útil para definição de intervenções. ^403^ ^,^ ^410^ A hipotensão sistólica está associada a disfunção ventricular esquerda e débito cardíaco reduzido, sendo um marcador de cardiopatia grave. ^405^ ^,^ ^409^ Aproximadamente um terço dos pacientes adultos CMH apresentam hipotensão sistólica intraesforço, causada por queda inadequada na resistência vascular sistêmica e baixa reserva de débito cardíaco. Essa hipotensão é definida como uma queda da PAS >20 mmHg. ^410^
Resposta pressórica deprimida* ^**4**^	1) Reserva pressórica sistólica (diferença entre a PAS máxima de esforço e PAS de repouso) <35 mmHg na ausência de queda acentuada na PAD, ou 2) Aumento máximo da PAS <140 mmHg, ou 3) Comportamento da PAS em platô (manutenção da PA por 2 ou mais estágios escalonados ou mais de 3 minutos consecutivos em protocolo de rampa) com reserva pressórica sistólica <35 mmHg. ^411^	Frequentemente associada a DAC grave e pior prognóstico. ^403^ Está associada a risco aumentado de eventos cardiovasculares e mortalidade por todas as causas. ^412^ Na CMH considerar resposta pressórica deprimida como uma falha em aumentar a PAS em pelo menos 20 mmHg do repouso até o pico do esforço. ^410^
Resposta normal da PA na recuperação*	1) PAS apresenta redução progressiva. No início da recuperação, a PAD pode apresentar pequena elevação ou manter-se inalterada. Aos 6 minutos, a PAS e a PAD tendem a retornar aos valores de repouso. 2) Relação da PAS do 3º minuto da recuperação / PASpico ≤0,9. 3) PA no quinto minuto da recuperação: PAS <160 mmHg e PAD <90 mmHg. 4) Ausência de hipotensão na recuperação.	Na ausência de outras alterações do TE, bom prognóstico e baixo risco. ^383^ ^,^ ^384^ ^,^ ^413^ ^,^ ^414^
**Resposta paradoxal da PA na recuperação**	Relação entre a PAS no 3º minuto da recuperação com a PAS do 1 **º** minuto da recuperação ≥1. ^415^ ^-^ ^417^	Preditora de DAC, IAM, AVC e mortalidade CV. ^387^ ^,^ ^418^ ^,^ ^419^
Recuperação lenta da PA sistólica na recuperação* ^**5**^	Independe do comportamento da PA de repouso e no esforço: – PAS do 3 **º** minuto da recuperação/PASpico >0,9. ^420^ – PA no 5 **º** minuto da recuperação: PAS ≥160 e PAD ≥90 mmHg. ^421^	Tem boa correlação com hipertensão arterial futura e DAC. ^420^
Hipotensão na recuperação* ^**5**^	Na recuperação, apresentar sensação de desmaio, tontura, náusea, pré-síncope e síncope associada a: – PAS da recuperação com queda >50% da PASmax do esforço ou – PAS da recuperação <90 mmHg. ^422^	A hipotensão arterial pós-esforço geralmente ocorre em indivíduos aparentemente sadios. A despeito de aumentar a incidência de arritmias, não tem associação com morbimortalidade CV, sendo mais frequente em indivíduos jovens exercitados até a exaustão. ^422^
**Resposta hipertensiva diastólica na recuperação**	PAD no 5 **º** minuto da recuperação ≥90 mmHg.	Preditora de HA futura, DAC e AVC. ^73^ ^,^ ^421^
PA: pressão arterial; TE: teste ergométrico; PAS: pressão arterial sistólica; PAD: pressão arterial diastólica; HVE: hipertrofia ventricular esquerda; VE: ventrículo esquerdo; HA: hipertensão arterial; RHE: resposta hipertensiva/exagerada; DAC: doença arterial coronariana; FA: fibrilação atrial; AVC: acidente vascular cerebral; CV: cardiovascular; IC: insuficiência cardíaca; IAM: infarto agudo do miocárdio; PASmax: PAS medida no esforço máximo; PASpico: PAS no pico do esforço mesmo que não esteja associada à exaustão física (esforço máximo). *Se em vigência de medicação, descrever se a resposta do TE é em vigência de uso ou não de drogas com efeito anti-hipertensivo. **O aumento médio da PAS geralmente é de 10 mmHg/MET. Em indivíduos que atingem carga de esforço >10 METs, deve-se considerar que a elevação da PAS seja de 6,2 mmHg/MET. ***Ocasionalmente, indivíduos sem doença cardíaca clinicamente significativa apresentarão hipotensão induzida pelo exercício relacionada a desidratação, dose inadequada da terapia anti-hipertensiva ou exercício extenuante prolongado. Caso assintomática, confirmar a queda da PA em pelo menos mais uma mensuração. * ^*4 *^ PAS com valor fixo durante a progressão do exercício, em atletas, crianças e adolescentes, e mulheres em fase estrogênica pode ser observada na ausência de doença. * ^*5 *^ Tanto para recuperação ativa quanto passiva.		

 A PAS máxima no esforço apresenta valores gradativamente maiores com o avanço da idade. Nas mulheres, a PAS máxima é geralmente menor do que nos homens, tendendo a torna-se semelhante nos idosos. Mulheres jovens podem apresentar resposta em platô da PAS ou mesmo ligeira queda no pico do esforço, sem significado clínico específico. ^6^ ^,^ ^87^ ^,^ ^390^

 A resposta da PAD é considerada anormal quando ocorrer elevação ≥15 mmHg e/ou atingir valor >90 mmHg, partindo-se de valores de PAD normal em repouso. ^391^ Há fortes evidências científicas, nacionais e internacionais, validando esses critérios de normalidade do comportamento da PAS e PAD durante o TE para o diagnóstico de HAS, avaliação de eficácia terapêutica, associação com DCV e estratificação de risco. ^391^ ^-^ ^393^

 A resposta hipertensiva ao esforço (RHE) é mais comum em idosos e em hipertensos, mesmo quando a PA está bem controlada em repouso. ^394^ Em hipertensos, a RHE está relacionada com maior risco de IC futura, hipertrofia de VE, disfunção endotelial, disfunção diastólica e eventos cardiovasculares. ^392^ ^,^ ^395^ ^,^ ^396^

 Na ausência de HAS ou outra doença cardiovascular, a ocorrência de RHE não é um fenômeno benigno, sendo os seus principais responsáveis a disfunção endotelial e o aumento da rigidez das grandes artérias. A RHE geralmente está associada a anormalidades funcionais e estruturais do VE, principalmente quando acompanhada de aumento da PA central. ^72^ ^,^ ^305^ ^,^ ^387^ ^,^ ^397^

 Paradoxalmente, estudos em pacientes com DAC (suspeita ou diagnosticada) demonstraram que a ocorrência de RHE foi associada a lesões coronarianas menos graves e menor mortalidade na comparação com os que apresentaram resposta normal da PA. ^398^ ^,^ ^399^

 Sugere-se a medição da PA ao atingir 5 METs de carga de esforço. A PAS ≥150 mmHg é limiar discriminatório de HA, associada a HA sistólica em MAPA de 24 horas e hipertrofia do VE ao ecocardiograma. ^400^ ^,^ ^401^

 Em coorte prospectiva com 6.578 participantes assintomáticos do *Lipid Research Clinics Prevalence Study* (idade média: 46 anos, 45% mulheres e seguimento médio 20 anos), nos normotensos ou pré-hipertensos no repouso, a PA no segundo estágio de Bruce >180/90 mmHg associou-se ao risco de morte por DCV (PAS RR: 1,96; IC 95%: 1,40 a 2,74; p<0,001/PAD RR: 1,48; IC 95%: 1,06 a 2,06; p=0,02). ^75^

 A hipertensão pré-teste com resposta normal da PA ao esforço é comum em pacientes ansiosos, e geralmente não está associada ao desenvolvimento de HA futura. ^215^ ^,^ ^402^

 Consideram-se como critérios de interrupção do esforço a elevação de PAS >250 mmHg e PAD ≥120 mmHg nos normotensos ou PAD ≥140 mmHg nos hipertensos. ^4^ ^,^ ^6^ ^,^ ^13^

**Figura 10:**
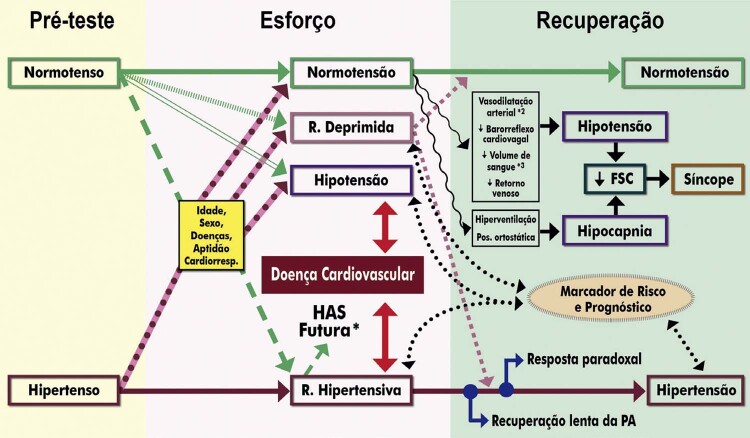
– Principais comportamentos da pressão arterial durante o TE e suas repercussões. HAS: hipertensão arterial sistêmica; FSC: fluxo sanguíneo cerebral; R. Hipertensiva: resposta hipertensiva; Pos. ortostática: posição ortostática na esteira ergométrica; Cariorresp.: cardiorrespiratória; PA: pressão arterial, *Normotensão no pré-teste com resposta hipertensiva no esforço corresponde a risco de hipertensão arterial sistémica no futuro. * ^2^ Associada a diminuição da atividade adrenérgica e vasodilatação histamina mediada. * ^3^ Associado a hipertermia no esforço e desidratação prévia (inclusive secundária a diuréticos).

 A ocorrência de hipotensão/queda da PA intraesforço, cuja incidência varia de menos de 2% a 6%, requer intervenção 

 imediata com a suspensão do esforço por motivos de segurança (risco agudo de evento cardiovascular) e colocação do paciente em decúbito dorsal. As causas mais frequentes são: DAC multiarterial grave com disfunção do VE; cardiomiopatias; obstrução da via de saída do VE; tônus vagal aumentado; hipovolemia; arritmias. Seu VPP é maior em homens do que em mulheres ( Figura 10 ). ^403^

 A hipotensão e a síncope na recuperação podem ter causas diversas, variando de respostas decorrentes de exaustão física em indivíduo aparentemente saudável até situação de anormalidade: obstrução da via de saída do VE (p. ex., EAo e cardiomiopatia hipertrófica obstrutiva); disautonomia; regulação negativa do barorreflexo vagal; Figura 10 ). ^74^ ^,^ ^386^ ^,^ ^404^ ^,^ ^405^

 A Tabela 32 apresenta a definição de termos, critérios de normalidade, interpretação e implicações quanto ao comportamento da PA no TE. 

### 3.2.3. Duplo-Produto

 O duplo-produto (DP) expressa o consumo de oxigênio miocárdico (relação linear com a captação de oxigênio pelo miocárdio e o fluxo sanguíneo coronariano). É calculado por meio da multiplicação da frequência cardíaca pela pressão arterial sistólica, a qualquer momento do TE. ^6^ ^,^ ^134^ ^,^ ^423^


DP=FC×PAS(bpm⋅mmHg)

O DP é importante na avaliação dos limiares de angina, alterações eletrocardiográficas (segmento ST e arritmias), eficiência cardiovascular, progressão da aptidão cardiorrespiratória, terapêutica medicamentosa e intervencionistas, sendo marcador de prognóstico 

 independente da presença de DAC e, portanto, recomenda-se sua utilização de forma seriada. O DP máximo (DPmax), geralmente obtido no pico do TE, se inferior a 25.000 bpm.mmHg, está associado a pior prognóstico. ^115^ ^,^ ^134^ ^,^ ^423^ ^,^ ^424^

 Fatores limitantes da avaliação do DP: uso de betabloqueadores e antiarrítmicos; hipertensão descontrolada; fibrilação e flutter atrial com resposta ventricular não controlada. Não deve ser calculado durante taquiarritmias. ^134^ ^,^ ^293^ ^,^ ^299^

##  4. Respostas Eletrocardiográficas 371 

 Para adequada análise, descrição e interpretação das respostas eletrocardiográficas, recomenda-se: 

 – Verificação do posicionamento e correta fixação dos eletrodos para minimizar erros e artefatos. ^425^ ^,^ ^426^

 – Utilizar a normatização para emissão de laudos eletrocardiográficos da Diretriz da Sociedade Brasileira de Cardiologia sobre a Análise e Emissão de Laudos Eletrocardiográficos – 2022. ^295^

 – Utilização de sistemas de medições automatizados para intervalos, durações e amplitudes das ondas e segmentos do eletrocardiograma (ECG). ^427^

 – Considerar os efeitos dos filtros de ECG que estiverem sendo utilizados (alta, média, baixa frequência) para a estabilização da linha de base, redução de artefatos musculares e de rede elétrica. Para os filtros de alta frequência de, no mínimo, 150 Hz para os grupos de adultos e adolescentes. Filtros com frequências mais baixas podem interferir na captação das espículas de marca-passos. ^295^ ^,^ ^428^ ^,^ ^429^

 – Revisão dos valores das medidas automatizadas de modo a afastar erros por possíveis interferências, artefatos ou anormalidades do traçado subjacente. ^427^ ^,^ ^430^

 – Descrição detalhada e contextualizada do registro eletrocardiográfico. 

### 4.1. Onda P

#### 4.1.1. Respostas Normais

 Em adultos, as ondas P no ECG de repouso, em presença de ritmo sinusal, apresentam-se positivas nas derivações D1, D2 e aVF, eixo vetorial médio de +60° (variando entre 0° e +90°), amplitude máxima de 250 mV (2,5 mm) e duração ≤110 ms. ^295^

**Figura 11:**
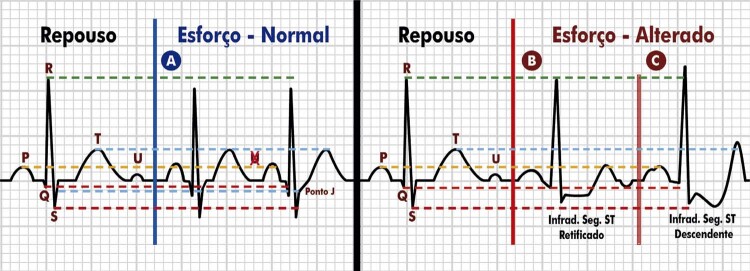
– Representação dos principais comportamentos das ondas P, Q, R, S, T, U, intervalo PR, segmentos PR e ST e ponto J durante a fase de esforço do TE. A. Esforço normal: onda P apresenta redução da duração e aumento de amplitude tornando-se apiculada; PRi apresenta redução com o aumento da frequência cardíaca (FC); segmento PR mantém-se inalterado (na linha de base); onda Q apresenta aumento do seu tamanho; onda R apresenta redução de tamanho concomitantemente ao aumento de onda S; ponto J mantém o mesmo nível do basal ou ocorre pequeno infradesnível; segmento ST mantém-se na linha de base ou pode apresentar infradesnivelamento ascendente rápido; onda T mantém seu tamanho e morfologia com componente inicial rápido e componente final lento (pode apresentar pequena diminuição e redução da duração); onda U tende a manter-se positiva no início do esforço e, com aumento da FC, tende a desaparecer. B. Esforço alterado: onda P apresenta aumento da duração e redução de amplitude; PRi mantém-se inalterado mesmo com o aumento da FC; infradesnivelamento do segmento PR >0,5 mm; manutenção da amplitude da onda Q; onda R inalterada mesmo com progressão do esforço e redução da onda S; infradesnivelamento do ponto J associado a infradesnivelamento do segmento ST com morfologia retificada e ponto Y ≥1,0 mm; redução da amplitude da onda T tendendo a simétrica; negativação da onda U. C. Esforço alterado: onda P apresenta aumento da duração tornando-se entalhada; aumento do PRi com o aumento da FC; desaparecimento de onda Q; aumento da amplitude da onda R com redução concomitante de onda S; infradesnivelamento do ponto J associado a infradesnivelamento do segmento ST descendente (ponto Y ≥1,0 mm); aumento da amplitude de onda T, apiculada e simétrica.

 Durante o exercício, normalmente observa-se ( Figura 11 ): ^431^

 – Aumento gradual e linear da amplitude da onda P com a elevação da FC (em derivações inferiores do plano frontal). Esse aumento é, em média, de 100 mV (1 mm). 

– Manutenção do eixo vetorial da onda P.

 – Normalmente não há alteração na duração da onda P ou raramente um aumento mínimo (≤20 ms). 

Comportamento normal da onda P na recuperação:

 – No primeiro minuto da recuperação, pode ocorrer aumento adicional da amplitude onda P (atingindo valor superior ao do esforço) mesmo com a redução da FC. Após, ocorre redução progressiva da amplitude e retorno ao padrão basal após o sexto minuto. ^432^

 – A duração da onda P mantém-se inalterada ou raramente apresenta aumento mínimo (≤20 ms) até o terceiro minuto. ^433^

#### 4.1.2. Respostas Anormais

 As principais respostas anormais da onda P são: ^431^ ^,^ ^434^ ^,^ ^435^

 1) Manutenção da amplitude da onda P de repouso, principalmente quanto ao componente negativo (mudanças <0,25 mm), aumenta a sensibilidade (69%) e especificidade (78%) para o diagnóstico de DAC. 

 2) Aumento da duração da onda P está associada à sobrecarga de pressão atrial esquerda durante isquemia esforço-induzida. 

 3) Mudança na morfologia da onda P com aumento da amplitude do componente negativo terminal na derivação V1, em 50% do tempo máximo de exercício, foi a alteração mais preditiva de DAC verificada pela cintilografia de perfusão. 

 4) A dispersão da duração de onda P combinada a infradesnivelamento do segmento ST aumentou a sensibilidade do TE para 79% e VPP para 91%. ^436^

## 4.2. Intervalo PR/Segmento PR

### 4.2.1. Respostas Normais

 No ECG de repouso, o segmento PR (PRs) começa com o final da onda P, termina com o início do complexo QRS e é normalmente isoelétrico. Serve como ponte temporal entre a ativação atrial e a ativação ventricular, bem como a recuperação atrial, que costuma ser de muito baixa amplitude e dificilmente detectada, por ocorrer dentro do QRS. Quando utilizada amplificação suficiente ou em presença de BAV de primeiro grau, é possível visualizar a onda de repolarização atrial (onda Ta). O início da onda P até o final da onda Ta equivale ao intervalo QT atrial. 

 O intervalo PR (PRi – início da P até o início do complexo QRS) normalmente mede 120 a 200 ms, sendo melhor determinado em DII. 

 O PRs serve como ponto de demarcação da linha de base que será utilizada para a avaliação das alterações do segmento ST e de amplitude de ondas. A linha que une as junções PQ (final do segmento PR e início do complexo QRS) é considerada como “linha de base”, considerando-se pelo menos quatro complexos sucessivos, no mesmo nível horizontal e sem artefatos. Os sistemas computadorizados utilizam algoritmos automatizados, considerando o final do PRs como a linha de base isoelétrica. ^1^ ^,^ ^295^

 No esforço, o PRs encurta e inclina-se discretamente para baixo nas derivações inferiores, enquanto o PRi sofre encurtamento diretamente proporcional à elevação da FC. ^437^

 Na fase inicial de recuperação, o PRi e o PRs são dependentes do grau de condicionamento físico dos pacientes, em que, em homens sedentários, o PRi médio é ≈110 ms e, em atletas, ≈280 ms. ^438^

### 4.2.2. Respostas Anormais

As principais alterações de PRs durante o TE são:

 – Mesmo em indivíduos normais, pode ocorrer maior inclinação decrescente do PRs atribuída à repolarização atrial exagerada (onda Ta negativa) que, caso persista, no período inicial de repolarização ventricular, causará infradesnivelamento do ponto J e segmento ST (ascendente, falso-positivo, se nas derivações inferiores). ^152^ ^,^ ^439^

 – Infarto atrial esforço-induzido correspondendo a infradesnivelamento do PRs >0,5 mm, normalmente acompanhado de supradesnivelamento do segmento ST (infarto ventricular), e a arritmias atriais. ^295^ ^,^ ^440^

 – Prolongamentos patológicos do PRi serão abordados na seção referente aos bloqueios atrioventriculares. 

## 4.3. Onda Q

### 4.3.1. Respostas Normais

 No ECG de repouso, as ondas Q são consideradas normais quando a sua duração é ≤30 ms e a amplitude é <0,2 mV (em adolescentes, pode atingir até 0,4 mV) o que corresponde a <25% da onda R seguinte. Na derivação D3, a duração da onda Q pode exceder 40 ms, mas raramente atinge 50 ms. A presença de onda Q em V1 é sempre patológica. ^295^ ^,^ ^371^

 No exercício, a onda Q normalmente aumenta significativamente sua amplitude, particularmente em CM5 e derivações laterais. ^441^

### 4.3.2. Respostas Anormais

 No ECG de repouso, a onda Q é considerada anormal quando, na ausência de bloqueio de ramo e/ou na síndrome de pré-excitação, sua duração é ≥40 ms e/ou amplitude maior que um terço da onda R adjacente (em duas ou mais derivações de uma mesma parede ventricular). 

 As principais alterações de onda Q durante o TE são: 

 – Redução de amplitude/desaparecimento da onda Q durante o esforço ou recuperação, podendo indicar isquemia septal e que, quando associada a infradesnivelamento do segmento ST (ISTs), aumenta o VPP para DAC. ^441^ ^,^ ^442^

 – Aumento da amplitude da onda Q associada a ISTs reduz o VPP, aumentando a possibilidade de falsos-positivos. ^443^

 – Aumento da duração da onda Q induzido pelo exercício (de 10±13 ms), em pacientes com DAC uniarterial e IAM recente, está associado à isquemia na cintilografia com tálio. ^444^

 – Ondas Q transitórias podem ser observadas na hipoglicemia, hipercalemia e asma. 

## 4.4. Onda R

### 4.4.1. Respostas Normais

 No ECG de repouso, a duração da onda R dependerá da derivação observada e duração/padrão do complexo QRS. A amplitude de R é variável, aumenta progressivamente nas derivações precordiais, sendo geralmente <27 mm em V5 e V6. ^445^

Durante o exercício, normalmente ocorre:

 – Redução da amplitude média das ondas R perto do esforço máximo (redução de 2,6±1,1 mm). ^446^ Diminuição acentuada é observada nas derivações laterais (V5 e V6) no exercício máximo e primeiro minuto de recuperação. 

 – À medida que a onda R diminui em amplitude, observa-se aumento da onda S. ^115^

### 4.4.2. Respostas Anormais

 No ECG de repouso, pacientes com infarto do miocárdio de parede anterior apresentam onda R com amplitude diminuída ou ausente. ^447^ ^,^ ^448^

 As principais alterações anormais da onda R durante o TE são: 

 – Aumento na amplitude da onda R decorrentes de alterações de dimensões do VE e isquemia miocárdica. ^449^ A presença e extensão de isquemia reversível em paciente com DAC correlaciona-se diretamente com o aumento da amplitude da onda R e ISTs. ^450^ ^,^ ^451^

 – A amplitude de R aumenta significativamente nas derivações precordiais durante episódios de isquemia transmural. ^452^

 – Ondas R de baixa amplitude (<10 mm) podem cursar com ISTs de pequena magnitude em relação à real isquemia, interferindo na acurácia do TE para diagnóstico de DAC. ^453^ ^,^ ^454^

## 4.5. Onda S

### 4.5.1. Respostas Normais

 No ECG de repouso, a onda S é comumente observada em DI, DII, DIII, aVF, V1 e V2 (em que sua amplitude é maior que a onda R), sendo frequentemente ausente em V5 e V6. Normalmente, a amplitude de S é <0,3 mV (30 mm). ^445^

 Durante o TE, a onda S, na ausência de distúrbio de condução intraventricular, normalmente: 

 – Tende a aumentar nas derivações ínferolateral (principalmente em aVF e V5; máximo 0,3-0,4 mV) com concomitante redução da onda R. ^115^ ^,^ ^455^

 – No primeiro minuto da recuperação, a amplitude de S permanece a mesma ou apresenta pequena redução. Retorna à amplitude pré-teste entre 3 e 5 minutos de recuperação supina. ^455^

 – A duração média de S apresenta redução progressiva com o incremento do esforço, independentemente de distúrbio de condução intraventricular. ^456^

### 4.5.2. Respostas Anormais

 As principais anormalidades referentes à onda S são: 

 – Diminuição da onda S em pacientes com DAC que se associa à isquemia subendocárdica mesmo na ausência de alterações do segmento ST, aumentando a sensibilidade do TE. ^457^

 – Em pacientes sem distúrbio da condução intraventricular, a duração inalterada geralmente associa-se à obstrução significativa em artéria coronária direita (CD) ou artéria circunflexa (CX). ^456^ ^,^ ^458^

 – Aumento significativo da duração (≈12,5±6 ms) é observado em pacientes com obstrução crítica em DA, no bloqueio divisional anterossuperior esquerdo e no bloqueio de ramo direito. ^456^

## 4.6. Duração QRS

### 4.6.1. Respostas Normais

 A duração dos complexos QRS geralmente diminui proporcionalmente com o aumento da FC no exercício (redução ≈3,0 a 4,9 ms). Excepcionalmente, em pacientes normais, não ocorre alteração da duração. ^459^

 O cálculo da variação da duração dos complexos QRS (ΔDQRS) corresponde à diferença da duração medida em aVF e V5 imediatamente na suspensão do esforço (início da recuperação) em relação ao repouso. ^459^ ΔDQRS normal é ≤3 ms. ^460^


△DQRS= duração QRS início recuperação - duração QRS repouso*

*Medidas em aVF e V5 (ms).

### 4.6.2. Respostas Anormais

Significado de alterações de ΔDQRS:

 – É considerado positivo para isquemia ΔDQRS >3 ms. ^460^ Em portadores de DAC, a variação está diretamente relacionada ao número de artérias com obstrução significativa (4,8 ms = uma artéria, 7,8 ms = duas e 13,3 = três artérias; p<0,001) e anormalidades de contração segmentar pela ventriculografia radioisotópica (6,7 ms = uma região do VE, 13,5 ms = duas e 21 ms = três regiões; p<0,0001). ^461^

 – ΔDQRS >3 ms na avaliação de DAC melhorou a precisão diagnóstica do TE, independentemente de alterações segmento ST, em comparação à cintilografia de perfusão (sensibilidade 93%, especificidade 71% e VPP 86%). ^459^

 – ΔDQRS >3 ms em mulheres é mais sensível e específico do que as alterações ST-T para a detecção de isquemia. ^462^ ^,^ ^463^ Estudo anterior mostrou que ΔDQRS alterado em mulheres aumentou sensibilidade (91%), especificidade (89%) e VPP (88%) do TE e, nas mais jovens (27-50 anos), a sensibilidade foi de 80% e especificidade de 83%. ^462^

 – ΔDQRS ≥15 ms em pacientes com cardiopatia isquêmica previu a ocorrência de arritmias ventriculares graves (taquicardia ou fibrilação ventricular) com VPP 73%. Após a revascularização cirúrgica, esses pacientes não apresentaram mais ΔDQRS alterado, e as arritmias foram suprimidas. ^464^

## 4.7. Fragmentação de QRS em Alta Frequência

### 4.7.1. Respostas Normais

 Fragmentação de QRS em alta frequência ou QRS em alta frequência (AFQRS) é uma técnica especial de filtragem do ECG (geralmente entre 150 e 250 Hz) que permite analisar os componentes de alta frequência dos complexos QRS. ^465^

 A técnica envolve combinar complexos QRS de uma mesma derivação (avaliando as 12 derivações) ou complexos de quatro derivações (V3, V4, V5 e V6) para formar um complexo precordial médio. Nesses complexos, calcula-se a raiz quadrada média normalizada (RQMN) e/ou a amplitude máxima normalizada (AMN). Esses parâmetros são geralmente avaliados no ECG de repouso, no pico do esforço e na recuperação. ^466^

 Em pessoas saudáveis, a AFQRS é considerada normal quando os valores médios da RQMN são >1 μV nas três etapas do TE (repouso, pico do esforço e recuperação) e se observa aumento da RQMN com o esforço e imediatamente após o esforço (comparado com valor de repouso). ^466^ ^,^ ^467^

### 4.7.2. Respostas Anormais

 A AFRQS é considerada anormal para isquemia, independentemente de alterações do segmento ST, quando: ocorre redução absoluta ≥1μV ou uma redução relativa ≥50% (entre os valores máximos e mínimos de RQMN) em ≥3 derivações, nos pacientes que atingiram esforço máximo. No esforço submáximo (≤85% da FC máxima prevista), a redução relativa deve ser ajustada linearmente, entre 40% e 50%, de acordo com a razão entre a FC máxima alcançada e a FC prevista. A AFRQS apresenta maior sensibilidade, especificidade e VPP quando comparada às alterações de segmento ST. ^468^ ^-^ ^470^

 AFRQS alterada é geralmente encontrada em pacientes pós IAM com cicatriz miocárdica, na isquemia ou por atraso de condução por ativação não homogêneo dos ventrículos. É um preditor de mortalidade e eventos cardíacos em pacientes com DAC. ^467^ ^,^ ^468^

 AFRQS alterada demonstrou valor diagnóstico incremental quando associada às alterações do segmento ST (VPP 80,4% *vs.* 74,9% p<0,0001). Análise conjunta da AFRQS e alterações do segmento ST identificou 92,3% dos indivíduos com isquemia significativa. ^471^

 Análise conjunta de AFRQS com ISTs demonstrou VPN de 99% para doença isquêmica grave. Na regressão multivariada, a AFRQS alterada foi fator independente de risco para eventos cardíacos maiores em 2 anos (RR: 2,8; IC 95%: 1,7-4,4; p<0,001). ^472^

## 4.8. Onda T

### 4.8.1. Respostas Normais

 No ECG de repouso, a onda T é arredondada, assimétrica, com porção inicial ascendente mais lenta e porção final descendente mais rápida, habitualmente com polaridade semelhante à do QRS e positiva em quase todas as derivações (sempre negativa em aVR). A duração varia de 100 a 300 ms, e sua amplitude é de, no máximo, 5 mm nas derivações periféricas e <15 mm nas precordiais, equivalendo a cerca de 10% a 30% da amplitude total do QRS que a antecede. ^473^ ^,^ ^474^

 Na fase inicial do esforço, ocorre diminuição geral na amplitude da onda T seguida de aumento em cargas de exercício mais altas (retornando ao nível basal), com aumento adicional na fase inicial da recuperação. ^455^

### 4.8.2. Respostas Anormais

 Na interpretação das alterações de onda T, deve-se considerar as doenças preexistentes (em especial HAS, valvopatias e IRC), fatores de risco e a probabilidade pré-teste de DAC. 

 No ECG de repouso, as principais alterações das ondas T que podem estar associadas à isquemia: 

 – Positivas, simétricas, pontiagudas e acompanhadas por onda U (positiva ou negativa). ^475^

 – Negativas, simétricas e pontiagudas em derivações com QRS predominantemente positivo (exceto DIII, aVR e V1). ^115^

 – Padrão bifásico nas derivações torácicas anteriores (V1 a V3), geralmente associado à isquemia miocárdica em quadro de angina instável. 

 – Achatadas e geralmente inespecíficas, podendo estar associadas à isquemia miocárdica. 

Comportamento anormal das ondas T ao esforço:

 – Aumento na amplitude (>2,5 mV), simétrica, nas derivações V2 a V4 em pacientes com dor torácica, sendo associada à isquemia grave. ^115^

 – Pseudonormalização de onda T correspondendo à presença de onda T invertida ≥1 mm em qualquer derivação do repouso e que se torna positiva com o pico do esforço, sendo geralmente associada a defeitos reversíveis e fixos na cintilografia de perfusão miocárdica. ^476^

 – Pseudonormalização de onda T associada à inversão da onda U nas derivações torácicas anteriores é altamente indicativa de estenose crítica da DA. ^477^

 – Pseudonormalização de onda T em derivações relacionadas a IAM prévio em baixa carga de esforço se mostrou índice sensível e específico para presença de viabilidade residual. A sensibilidade e a acurácia foram maiores para infartos anteriores. ^478^ ^,^ ^479^

 – Em TE submáximo realizado em fase precoce de angina instável estabilizada ou IM sem onda Q, a pseudonormalização de onda T, independentemente da ocorrência de ISTs, foi preditora de sobrevida em seguimento de 6 meses. ^480^

 – Pseudonormalização de onda T em pacientes com baixa prevalência de DAC é um achado não diagnóstico. ^152^

## 4.9. Onda U

### 4.9.1. Respostas Normais

 No ECG de repouso, a onda U é uma deflexão de baixa amplitude e frequência, ocorre após a onda T (geralmente com a mesma polaridade), amplitude proporcional à da onda T (≈5% a 25% da amplitude; média: 0,33 mm), sendo observada melhor em V2 e V3 (≤2 mm), duração de 221±73 ms (<50% da onda T precedente) e mais comumente identificada em FC <95 bpm. É observada em até 50% dos indivíduos normais. No esforço, normalmente mantém duração, amplitude e eixo, sendo difícil identificá-la em FC >120 bpm devido à aproximação das ondas T e P. ^481^ ^-^ ^483^

### 4.9.2. Respostas Anormais

 Onda U negativa no ECG de repouso é considerada anormal e frequentemente associada a regurgitação mitraI e/ou aórtica, HAS e cardiopatia isquêmica. ^484^ ^,^ ^485^ Amplitude aumentada pode estar associada a hipopotassemia e uso de medicamentos (digitálicos, amiodarona e quinidina). ^483^ ^,^ ^484^ ^,^ ^486^

 As principais alterações referentes à onda U durante o TE são: 

 – No basal (positiva), com aumento da amplitude ao esforço (≥0,5 mV) em derivações precordiais, está associada à isquemia miocárdica ínferoposterior, sendo marcador de obstrução significativa em CX ou CD. ^487^ ^,^ ^488^

 – Aparecimento de onda U positiva no esforço e/ou nos primeiros 3 minutos da recuperação está frequentemente associado à obstrução coronariana em artéria CX e/ou CD. ^489^

 – Aparecimento de onda U negativa de amplitude ≥0,5 mV, persistindo por pelo menos 1 minuto durante e/ou após esforço, está associado à DAC grave em DA. ^487^ ^,^ ^490^

 – Aparecimento de onda U negativa é marcador de circulação colateral bem desenvolvida em pacientes com DAC grave ou angina estável. ^487^ Em derivações precordiais, é marcador de miocárdio viável pós-IAM anterior. ^491^

 – Inversão de onda U esforço-induzida transitória com amplitude ≥0,5 mV, em derivações de parede anterior (V2 a V5), está associada a episódios de isquemia aguda e DAC grave em DA. ^492^ ^,^ ^493^

## 4.10. Repolarização Precoce

 O padrão de repolarização precoce (PRP) pode ser observado em 1% até 13% da população geral. ^494^ ^,^ ^495^ Em indivíduos de meia-idade, associou-se a maior risco de morte súbita cardíaca (MSC). ^496^ ^,^ ^497^

 Os critérios para diagnóstico de repolarização precoce no ECG de repouso são: ^498^ ^,^ ^499^

**Figura 12:**
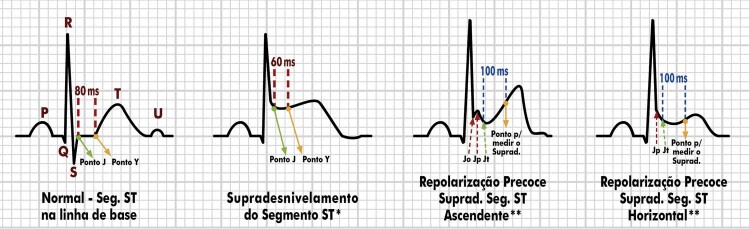
– Padrões de supradesnivelamento do segmento ST incluindo a repolarização precoce. Seg. ST: segmento ST; Suprad. Seg. ST: supradesnivelamento do segmento ST; ms: milissegundos. *Supradesnivelamento do segmento ST esforço-induzido (≥1,0 mm medido a 60 ms após o ponto J). **No padrão de repolarização precoce, o supradesnivelamento do segmento ST deve ser medido a 100 ms após o ponto Jt, e também utilizado para avaliar o padrão de supradesnivelamento (ascendente, horizontal ou descendente).

1) Duração dos complexos QRS <120 ms.

 2) Presença de entalhe ou ligadura QRS final na inclinação descendente de uma onda R proeminente. Se houver um entalhe, ele deve ficar totalmente acima da linha de base. O início de uma ligadura (Jo) também deve estar acima da linha de base ( Figura 12 ). 

 3) O ponto Jp (pico do entalhe do ponto J) deve ter ≥0,1 mV em 2 ou mais derivações contíguas do ECG de 12 derivações, exceto de V1 a V3. ^500^

 O supradesnivelamento do segmento ST (SSTs) deve ser medido a 100 ms após o ponto Jt (final do entalhe do ponto J). Além da magnitude do supradesnivelamento, deve ser descrito o padrão: 

 – “Repolarização precoce com segmento ST ascendente”, quando o segmento ST estiver inclinado para cima e seguido por uma onda T vertical. 

 – “Repolarização precoce com segmento ST horizontal ou descendente”, quando o segmento ST for horizontal ou descendente (inclinado para baixo). 

Comportamento e significado do PRP no TE:

 – Comum em indivíduos jovens, geralmente apresenta redução progressiva com o esforço, podendo ocorrer seu desaparecimento em cargas moderadas. PRP com SSTs ascendente rápido, em derivações anterolateral, tem sido encontrado em atletas. ^501^

 – Em pacientes sintomáticos (devido a morte súbita cardíaca abortada, arritmia ventricular sustentada e/ou síncope inexplicada), observou-se PRP persistente ao esforço. ^502^

 – O retorno do PRP na recuperação é progressivo, lento e, em aproximadamente 30% dos pacientes, ocorre no 5º minuto. ^503^ ^,^ ^504^

 Marcadores de alto risco verificáveis no TE em pacientes com PRP: ^55^ ^,^ ^494^

– Ocorrência de TV polimórfica esforço-induzida.

 – ISTs horizontal e/ou descendente em derivações inferiores ou ínferolaterais, associadas ao risco de FV idiopática e aumento em três vezes do risco de morte súbita arrítmica. ^500^

## 4.11. Supradesnivelamento do Segmento ST

 Supradesnivelamento do segmento ST induzido pelo esforço (SSTE) é definido como uma elevação do segmento ST ≥1,0 mm (≥0,10 mV) em 60 ms após o ponto J, ocorrendo em 2 ou mais derivações, independentemente de presença de onda Q ( Figura 12 ). O SSTE pode estar acompanhado de infradesnivelamento recíproco do segmento ST (“imagem em espelho”). ^505^ ^,^ ^506^

 SSTE geralmente está associado a isquemia miocárdica grave (geralmente transmural), espasmo da artéria coronária, angina Prinzmetal, aneurisma ventricular esquerdo, isquemia peri-infarto e anormalidades da movimentação da parede VE. ^506^ ^,^ ^507^

 As derivações nas quais ocorrem SSTE têm correlação com segmentos anatomovasculares do VE. ^506^ ^,^ ^508^ ^,^ ^509^ Sugere-se a utilização da descrição topográfica de manifestações isquêmicas (Meyers): ^295^

– Parede anterosseptal: derivações V1, V2, V3.

– Parede anterior: derivações V1, V2, V3 e V4.

 – Parede anterior localizada: derivações V3, V4 ou V3-V5. 

 – Parede anterolateral: derivações V4 a V5, V6, D1 e aVL. 

– Parede anterior extensa: V1 a V6, D1 e aVL.

– Parede lateral: derivações V5 e V6.

– Parede lateral alta: D1 e aVL.

– Parede inferior: D2, D3 e aVF.

 Os termos “parede posterior” e “dorsal” não deverão mais ser utilizados, em vista das evidências atuais de que as derivações V7 a V9 referem-se à parede lateral. 

 Trinta por cento dos pacientes com IAM prévio de parede anterior e 15% de parede inferior apresentam SSTE nas derivações envolvidas e seu significado varia com os achados adicionais do TE. ^510^ ^,^ ^511^ A associação do SSTE em derivações com ondas Q anormais pode representar isquemia residual da área peri-infarto (viabilidade miocárdica), discinesia ventricular ou movimento acinético segmentar do VE. ^511^ ^,^ ^512^ Entretanto, não permite quantificar o tecido viável justificando investigação adicional por método de imagem para indicar intervenção terapêutica. ^512^ ^,^ ^513^

Particularidades do SSTE:

 – SSTE ≥0,2 mV (2 mm) em derivações sem ondas Q é critério de interrupção do esforço. 

 – SSTE é mais comumente associado à obstrução proximal grave do que ao espasmo coronariano em artérias não obstruídas. ^514^ ^,^ ^515^

 – SSTE, com ou sem ISTs, foi preditivo da presença, extensão e localização da isquemia miocárdica avaliada por cintilografia de perfusão miocárdica. O SSTE em V1 com ISTs em aVR e V4-V6 associou-se à estenose no tronco da artéria coronária esquerda (TCE) ou em artéria descendente anterior (DA) proximal. ^509^

 – SSTE em aVR apresentou sensibilidade de 100% na detecção de estenose de TCE (especificidade 33,5%) e de 94,3% para a DA (especificidade 26,6%). A associação de SSTE em aVR e V1 reduziu a sensibilidade (74,4% e 65,9%) e aumentou a especificidade (68,5% e 64,4%). ^508^ ^,^ ^516^

 – SSTE em aVR esteve associado a eventos cardíacos adversos maiores (seguimento de 2 anos) em 33% dos pacientes. ^517^

 – Em pacientes pós-IAM uniarterial com angioplastia transluminal coronária percutânea (ATCP) – sem isquemia residual –, o SSTE pode estar associado a microcirculação coronariana prejudicada e menor viabilidade miocárdica. ^518^

## 4.12. Ponto J e Infradesnivelamento Ascendente

 O ponto J (junção do fim QRS e início do segmento ST) costuma apresentar infradesnivelamento ao esforço, em derivações de parede lateral, retornando gradualmente aos valores pré-exercício na recuperação. O infradesnivelamento do ponto J é mais comum em pacientes idosos e geralmente não se associa a DAC. ^519^

**Figura 13:**
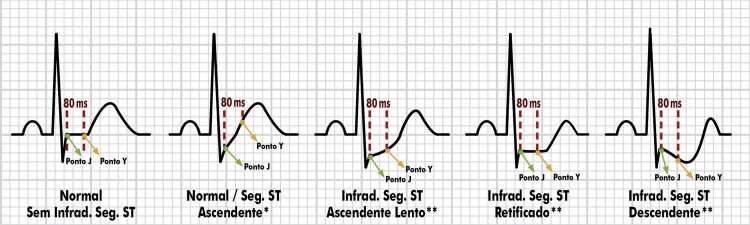
– Comportamento do segmento ST e tipos de infradesnivelamento. *Ascendente, horizontal ou descente: dependendo da FC no momento de medida, o ponto Y estará em 60 ou 80 ms do ponto J. **Ascendente lento: aferido no ponto Y em 80 ms do ponto J.

 O infradesnivelamento ascendente do segmento ST – depressão do ponto J seguido de infradesnivel STs rapidamente ascendente e sem depressão no ponto Y (medido em 60 ou 80 ms do ponto J) – é observado em 10% a 20% dos indivíduos normais. Não é considerado critério de diagnóstico de DAC ( Figura 13 ). ^519^

##  4.13. Infradesnivelamento do Segmento ST: Ascendente Lento, Horizontal e Descendente 

 O infradesnivelamento de ST esforço-induzido (ISTE) é a manifestação eletrocardiográfica mais frequente da isquemia miocárdica (geralmente subendocárdica). A acurácia diagnóstica do ISTE dependerá de idade, sexo, características clínicas do paciente, doenças cardiovasculares preexistentes, prevalência de DAC, intensidade do esforço e FC alcançadas. ^277^ ^,^ ^453^ ^,^ ^520^ ^,^ ^521^

 Consideram-se anormais e sugestivas de isquemia esforço-induzida as seguintes alterações do segmento ST na fase de esforço e/ou recuperação ( Figura 13 ): 

 1) Infradesnivelamento com morfologia horizontal (retificado) ou descendente, ≥1 mm (aferido no ponto Y). Dependendo da FC no momento de medida, o ponto Y estará em 60 ou 80 ms do ponto J (exemplo: geralmente, em crianças/jovens 60 ms e nos adultos/idosos 80 ms). ^6^ ^,^ ^522^ ^,^ ^523^

 2) Infradesnivelamento com morfologia ascendente lenta (utilizar escores de risco para DCV pré-teste – ver na Parte 2, Seção 2.3): 

 – ≥1,5 mm, em indivíduos de risco moderado ou alto de doença coronariana. 

 – ≥2 mm em indivíduos de baixo risco de doença coronariana (aferido no ponto Y em 80 ms do ponto J). ^524^ ^-^ ^527^

 ISTE ascendente lento apresenta menores sensibilidade, especificidade, baixo VPP e mais resultados falsos-positivos quando comparado ao horizontal/descendente. 

 Particularidades quanto a quantificação e interpretação do ISTE: 

 – ISTE <1,0 mm não preenche critérios para isquemia miocárdica (não isquêmico). 

 – Quando houver infradesnivelamento do segmento ST em repouso, na posição ortostática, considerar apenas a depressão adicional de ST durante o esforço. 

 – A existência de ISTs no repouso ≥1,0 mm reduz a associação de ISTE adicional com DAC obstrutiva. ^528^ No entanto, em pacientes sob investigação de dor torácica, a presença de IST no repouso não interferiu na acurácia diagnóstica e sensibilidade do TE. ^529^

 – Fatores que influenciam a magnitude de ISTE e gravidade da DAC: probabilidade pré-teste; aptidão cardiorrespiratória; momento do aparecimento; carga de esforço associada; duração e número de derivações com infradesnivelamento; momento de normalização na recuperação. Quanto menor a carga de esforço e DP em que ocorre o ISTE, pior é o prognóstico e maior a probabilidade de DAC multiarterial. ^522^ ^,^ ^523^ ^,^ ^530^ ^,^ ^531^

 – Na repolarização precoce em repouso, caso ocorra ISTE, considerar apenas as alterações abaixo da linha de base. 

 – Na vigência de bloqueio de ramo direito, não valorizar as alterações secundárias de ISTs nas derivações V1, V2 e V3 quanto à isquemia; a análise e a interpretação de ISTE em outras derivações seguem o padrão convencional descrito nesta seção. 

 – ISTE associado a angina ou correspondente anginoso esforço-induzido aumenta a sensibilidade do TE, estando associado a DAC grave e pior prognóstico. ^532^

 – ISTE ≥3 mm (0,3 mV), adicional aos valores de repouso, na presença de DAC suspeita ou conhecida, é critério de interrupção do esforço. ^6^ ^,^ ^209^

 – ISTE descendente geralmente está associado à isquemia mais grave quando comparado ao ISTE horizontal. ^533^ ^,^ ^534^

 – A normalização precoce de ISTE anormal no primeiro minuto da recuperação foi associado a menor carga isquêmica e maior probabilidade de “falso-positivo” para DAC. ^535^ ^,^ ^536^ Entretanto, a persistência do ISTE por período >3 minutos foi associado à DAC grave. A recorrência de ISTE após seu desaparecimento no início da recuperação também indica DAC grave. ^4^ ^,^ ^299^

 – ISTE observado exclusivamente na recuperação apresenta as mesmas acurácias diagnóstica e prognóstica ao constatado no esforço. ^537^ ^-^ ^539^ Entretanto, a ocorrência de ISTE exclusivamente na recuperação tardia (após terceiro minuto) aumenta a probabilidade de resultado falso-positivo para DAC. ^538^

 Quanto ao infradesnivelamento do segmento ST na estratificação de risco: 

 – ISTE em assintomáticos, de ambos os sexos, geralmente está associado a maior probabilidade de eventos coronarianos futuros (angina, IAM ou morte cardíaca). ^184^ ^,^ ^540^ ^,^ ^541^

 – Em seguimento de 19 anos, o ISTE associado à baixa capacidade física (<8 METs) apresentou RR 4,8 vezes maior (IC 95%: 2,9-7,9; p=0,013) de morte súbita cardíaca. ^541^

 – Em seguimento de 3,4 anos de pacientes com IST de repouso ≥1,0 mm, somente o ISTE adicional ≥2,0 mm foi significativo na avaliação prognóstica de IAM e morte súbita cardíaca. ^542^

 – Coorte prospectiva de 11.605 pacientes (52,9% masculinos, seguimento médio 6,7 anos), a ocorrência de ISTE (horizontal ou descendente) sem angina típica foi associada a RR de 3,9 (2,7-5,7) de SCA em 1 ano, e nos com angina típica, RR de 20,8 (13,9-31,3). ^543^

 – Estudo prospectivo em 366 mulheres na peri/pós-menopausa (54,4±5,5 anos), com Escore de Framingham de baixo a intermediário risco, seguidas por 5 anos, o ISTE horizontal/descendente ≥1 mm foi fator de risco independente para eventos CV (RR: 10,3; IC 95%: 1,9-61,4; p=0,007). ^544^

 A Tabela 33 apresenta as principais situações e condições que interferem na avaliação das alterações do segmento ST quanto à presença de isquemia miocárdica e DAC. 

**Tabela 33:** – Condições que interferem na interpretação das alterações da repolarização no TE para o diagnóstico de DAC 4,6

Invalidam a interpretação	Interpretação possível com menor acurácia
Síndrome de Wolff-Parkinson-White	Onda de repolarização atrial exagerada*
Variantes de síndrome de pré-excitação	Prolapso da valva mitral
Bloqueio de ramo esquerdo	Cardiomiopatias, valvopatias, pericardite
Marca-passo artificial estimulando o ventrículo	Distúrbios metabólicos, hipocalemia
Infradesnivelamento do segmento ST ≥1 mm no ECG de repouso	Fármacos anti-isquêmicos, antiarrítmicos e betabloqueadores
Terapêutica com digitálicos	Hipertrofia VE no ECG de repouso
ECG com qualidade técnica insatisfatória	
*Onda Ta negativa que, no período inicial de repolarização ventricular, pode causar infradesnível do ponto J e segmento ST (ascendente, falso-positivo).	

 O TE é considerado não diagnóstico para DAC quando não se atinge 85% da FC máxima prevista na ausência de alterações do segmento ST e/ou angina (ou equivalente anginoso). ^4^ ^,^ ^6^

### 4.13.1. Sinal de Corcunda do Segmento ST

 O “sinal de corcunda do segmento ST” (SCSST – do inglês “ *hump sign* ”), também denominado infradesnivelamento com convexidade superior, tem sua origem atribuída às ondas de repolarização atrial exageradas com infradesnível ascendente do STs seguida de onda com morfologia de uma corcunda após o ponto J. ^545^ ^,^ ^546^ Em indivíduos sem cardiopatia e assintomáticos, tem sido associado à hipertensão de repouso e à resposta hipertensiva exagerada ao esforço, sendo provavelmente resultado “falso-positivo” para DAC obstrutiva e com bom prognóstico. ^547^

Particularidades do SCSST:

 – Foi encontrado associado à disfunção diastólica do VE. ^546^

 – Na cardiomiopatia hipertrófica (seguimento de 5,3 anos), foi considerado fator de risco para morte súbita cardíaca. ^548^

 – Em coorte com 81 pacientes com cardiomiopatia hipertrófica (média 42 anos; 30% mulheres; seguimento 5,3 anos), ocorreu em 52% dos pacientes com taxa de mortalidade CV de 19%. ^548^

 – Em coorte com 237 pacientes não consecutivos (59% homens, média de 41 anos), dos quais 130 com “sinal de corcunda” observou-se forte correlação com a disfunção diastólica do VE (em 88% dos pacientes). ^546^

 O SCSST carece de estudos adicionais para melhor definição de sua aplicabilidade clínica. 

## 4.14. Normalização de Alterações do Segmento ST

 Alterações de repolarização presentes em repouso (inversão da onda T e ISTs) podem, durante o esforço, apresentar redução progressiva até a sua normalização (“pseudonormalização do segmento ST”) durante episódios anginosos e na DAC crônica. ^549^ ^,^ ^550^ Pode estar relacionada aos efeitos de cancelamento de vetores direcionados opostamente às áreas de isquemia. É infrequente e só deve ser considerada quando associada a dor ou equivalente anginoso. ^549^ ^,^ ^551^

##  4.15. Inclinação ( *Slope* ) ST/FC, Índice ST/FC, Loop do ST/FC e Histerese ST/FC 

 A magnitude do ISTs está associada aos aumentos de cargas de esforço, da FC e da demanda miocárdica de oxigênio, principalmente em presença de DAC. A correlação do ISTs ao comportamento da FC melhora a precisão do TE (VPP) no diagnóstico e estratificação de risco da DAC. ^423^ ^,^ ^552^

###  4.15.1. Inclinação ( *Slope* ) ST/FC 

 A inclinação ( *slope* ) ST/FC é calculada por meio de regressão linear correlacionando a magnitude de ISTs (mais frequentemente a 60 ms do ponto J), em cada derivação individualmente (incluindo CM5; excluindo aVR, aVL e V1), com a FC respectiva em cada estágio do TE. ^553^ O uso prático da inclinação ST/FC requer um protocolo de esforço com pequenos incrementos necessários para o adequado cálculo por regressão. ^554^

 A inclinação ST/FC >2,4 µV/bpm é considerada anormal e valores >6 µV/bpm são sugestivos de DAC grave (DAC triarterial ou TCE). ^553^

Particularidades da inclinação ST/FC:

 – Demonstrou sensibilidade de 78%, especificidade de 93% e acurácia de 89% na identificação de DAC triarterial ou lesão de TCE. ^555^

 – No pico do esforço, em pacientes com suspeita de DAC, quando ≥6,0 µV/bpm identificou DAC triarterial com sensibilidade de 78%, especificidade 97%, VPP de 93% e acurácia de 90%. ^556^

### 4.15.2. Índice ST/FC

 O índice ST/FC representa a variação do infradesnivelamento do segmento ST (ΔIST) pela variação da FC (ΔFC) em posição ortostática ou sentado, não requer cálculo de regressão e nem protocolo de incremento suave. ^557^ O índice ST/FC é definido como anormal quando >1,6 µV/bpm. ^553^ ^,^ ^557^ ^,^ ^558^


$$\text{ Índice ST/FC }(\mu V/bpm)=\frac{\Delta IST=(\text{ maior IST ao esforço }-\text{ IST repouso em milivolts })}{\Delta FC=(FC\text{ máxima }-FC\text{ de repouso })}\times 100$$

ΔIST

ST;ΔFC

Particularidades do índice ST/FC:

 – O valor de 4,7 µV/bpm (±4,7) esteve associado à isquemia grave na cintilografia de perfusão miocárdica (pontuação ≥11; p<0,0001) com sensibilidade de 77% e especificidade de 82%. ^559^

 – Aprimorou a predição de risco de eventos coronarianos em homens assintomáticos de alto risco e também em homens e mulheres assintomáticos de baixo risco. ^557^

###  4.15.3. *Loop* ST/FC 

 O *loop* ST/FC é um gráfico contínuo refletindo o comportamento do desnível do segmento ST (supra ou infradesnivelamento) em relação à variação da FC na derivação V2 durante o esforço e a cada minuto da recuperação. De acordo com o sentido da rotação do *loop* ST/FC, os pacientes podem ser divididos em: rotação horária com o desnível mudando mais rapidamente na recuperação; rotação anti-horária com desnível sendo mais prolongado durante a recuperação. ^560^

 Particularidades do *loop* ST/FC: 

 – Em pacientes pós IAM, o *loop* ST/FC com rotação anti-horária apresentou sensibilidade, especificidade e acurácia, respectivamente, de 88%, 73% e 77%, estando fortemente relacionada à isquemia miocárdica na área peri-infarto. ^561^

 – A frequência de amostragem <2 amostras/min pode prejudicar o desempenho no diagnóstico de DAC. ^562^

### 4.15.4. Histerese ST/FC

 A histerese ST/FC mensura as diferenças entre as áreas de ISTs das fases de recuperação e esforço em relação às FC correspondentes. As amplitudes do segmento ST são medidas em microvolts, a 60 ms do ponto J. ^563^ ^,^ ^564^ Pode proporcionar maior precisão diagnóstica e prognóstica comparada à análise isolada do ISTs e do índice ST/FC. ^563^ ^,^ ^565^ ^,^ ^566^

Particularidades da histerese ST/FC:

 – Demonstrou 89% de acurácia na detecção de DAC. ^563^

 – Valor de -15 µV permitiu a melhor discriminação diagnóstica de DAC. Nesse estudo, a inclinação de ST/FC de 2,4 µV/bpm e o índice ST/FC de 1,6 µV/bpm também auxiliaram no diagnóstico de DAC. ^567^

## 4.16. Intervalo QT/QTc/Histerese QT/Dispersão QT

 O intervalo QT (QTi) representa a atividade elétrica ventricular, guarda relação direta com a FC (aumento da FC acarreta encurtamento do QTi), sendo influenciado por alterações neuro-humorais inclusive no esforço. ^568^ ^,^ ^569^

 Devido à variação do QTi com a FC, recomenda-se corrigir o QTi pela FC (QTc): 


ST;ΔFC

*QT medido em milissegundos e distância entre RR em segundos.

**Figura 14:**
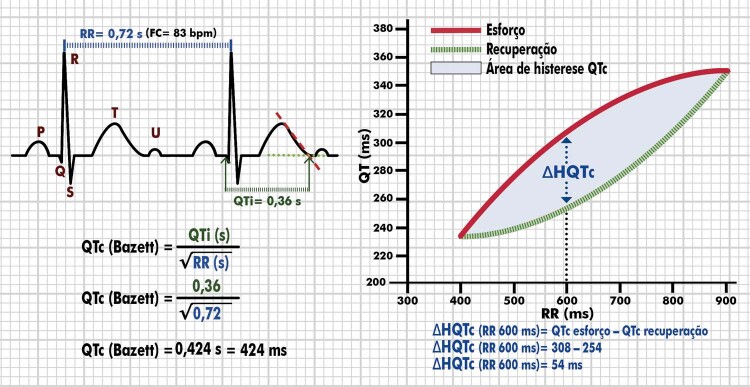
– Exemplo de avaliação do intervalo QT, QTc e cálculo da histerese do QTc.

 No ECG basal, o QTc geralmente é corrigido pela fórmula de Bazzet, para FC entre 60 e 90 bpm ( Figura 14 ). Quando FC <60 bpm ou >90 bpm, deve-se utilizar fórmulas como as de Fridericia, Framingham e Hodges. Uma limitação técnica é a dificuldade de aferição exata do QTi em todas as fases do esforço, especialmente com FC mais elevada. ^6^ ^,^ ^570^ ^,^ ^571^

 No ECG basal, o QTi é prolongado quando >500 ms, e o QTc é normal até ≤450 ms para homens e ≤470 ms para mulheres. ^295^ ^,^ ^371^ ^,^ ^572^

 Normalmente, o QTi diminui desde o início do esforço. Um aumento da FC para 160 bpm reduz o QTi em 25% a 40%. ^573^ Entretanto, em alguns indivíduos (comumente mulheres), pode haver prolongamento paradoxal do QTi nos primeiros minutos do esforço. 

 O QTc aumenta no início do esforço, seguido de diminuição progressiva com elevação da FC. Na recuperação, com a queda da FC o QTc retorna ao padrão basal. ^574^ ^,^ ^575^

 A dispersão do QTi (DQTi) é a diferença entre o maior e o menor valor de QTi medidos nas 12 derivações do ECG em determinada fase do TE (esforço ou recuperação). 

 A histerese do QTc (HQTc) – histerese QT/RR – geralmente é estimada por meio de um dos métodos ( Figura 14 ): ^576^ ^,^ ^577^

 – Variação da HQTc (∆HQTc) estimada pela diferença (em ms) entre o QTi medido em um RR predeterminado (geralmente de 600 ms) medidos no esforço e recuperação. ^578^

 – Área das curvas de HQTc (AHQTc) estimada pela quantificação da diferença das áreas (em ms) das curvas QT/RR ajustadas separadamente para aumento e queda da FC, durante o esforço e recuperação. ^579^

 Mulheres saudáveis apresentam maior variação do QTc durante o esforço e recuperação do que os homens, resultando em uma maior HQTc. ^580^

Respostas anormais:

 – Ausência de diminuição QTc no pico do esforço tem sido associada à isquemia esforço-induzida. Entretanto, não pode ser utilizada como critério único para seu diagnóstico. ^581^

 – Pacientes com DAC estável e TE normal (sem isquemia induzível) apresentam maior risco arrítmico quando o QTc no pico do esforço aumentou significativamente em relação ao basal (de 381 ms para 447 ms; p<0,001). ^582^

 – Em pacientes com alterações isquêmicas do segmento ST, o QTc (fórmula de Bazett; OR: 1,051) e o DQTi (OR: 1,117) medidos no segundo minuto da recuperação foram preditores independentes para DAC crítica. Na recuperação, o QTc ≥404 ms ou o DQTi ≥37 ms aumentou a sensibilidade para 90%. ^583^

 – O prolongamento do QTc esforço-induzido (QTc>440 ms – fórmula de Bazett) possibilitou diferenciar pacientes após IAM com alto risco de morte súbita cardíaca. ^584^

 – Histerese do intervalo QTc (método AHQTc) durante o esforço e a recuperação ≥375 foi preditor independente de isquemia miocárdica (OR: 1,61; IC 95%: 1,22-2,12; p=0,0008). ^585^

 – Histerese do intervalo QTc (método AHQTc) de 11 ms mostrou sensibilidade de 77,9%, especificidade de 85,2%, VPP de 87%, VPN de 75,4% e acurácia superior à do Escore de Duke para detecção de DAC. ^586^

 – Em estudo com 273 pacientes sem IAM (idade 56±9 anos, ambos sexos) o DQTi imediatamente após o esforço (≥60 ms; OR: 2,60; p<0,01) foi preditor de DAC significativa, independentemente do sexo ou da presença de ISTs. ^587^

 – Medidas do QTc no pico do esforço e recuperação (3-4 minutos) contribuem na identificação da SQTL1, sendo recomendadas para sobreviventes de morte súbita cardíaca. ^70^ ^,^ ^153^ ^,^ ^588^ ^,^ ^589^

 – Pacientes com SQTL1 apresentaram aumento máximo do QTc no pico do esforço (DP: ±21 a ±90 ms). Prolongamento do QTc >30 ms no terceiro minuto de recuperação associou-se a 75% de testes genéticos positivos para SQTL1. ^140^

 – Pacientes com SQTL1 apresentaram prolongamento progressivo ou persistente do QTc com o incremento da FC ao esforço. Na SQTL2, ocorreu prolongamento máximo do QTc em FC submáximas (50% da FC máxima prevista), sendo o QTc no pico do esforço significativamente menor do que na SQTL1 (335±45 ms *vs* . 366±33 ms; p=0,01). ^590^

 – A ocorrência isolada de QTc >480 ms no 4º minuto de recuperação confere 1 ponto no escore de risco da síndrome do QT longo (escore de Schwartz). ^591^ Algoritmo de triagem combinando o QTc no repouso e aos 4 minutos de recuperação mostrou sensibilidade de 94% e especificidade de 90% para detectar portadores de SQTL. ^592^

 Recomenda-se que os valores do QTi e QTc sejam sempre verificados no ECG basal e, quando normais os seus valores, não precisam ser registrados no laudo do TE. Quando anormais ou em TE para investigação do comportamento do intervalo QT e em pacientes recuperados de morte súbita, o laudo deve incluir os valores de QTc do ECG basal, a fórmula utilizada para correção, o maior valor de QTc observado no esforço, o QTc no pico do esforço e o comportamento do QTc na recuperação (registrar obrigatoriamente o QTc do 4º minuto). 

##  4.17. Distúrbios da Condução Atrioventricular, Intraventricular e da Formação do Impulso 

### 4.17.1. Distúrbios da Condução Atrioventricular

**4.17.1.1. Bloqueio Atrioventricular (BAV) de Primeiro Grau**

 Definido como prolongamento do intervalo PR (PRi) >200 ms (para FC entre 50 e 90 bpm) no ECG basal de adultos. A prevalência varia com a idade: em adultos jovens saudáveis (20 a 30 anos), é de 0,65% a 2%; na população geral, ≈4%; acima dos 60 anos, entre 3% a 5%. Geralmente é assintomático e, em acompanhamento por médio e longo prazo, associou-se a risco ligeiramente aumentado de DAC, IC e FA. ^593^ ^-^ ^596^ Nos pacientes de alto risco CV, associou-se fortemente a AVC isquêmico, IAM e morte CV. ^593^ ^,^ ^595^ ^,^ ^597^

 Na maioria dos casos, o prolongamento PRi observado no ECG basal sofre normalização com o esforço devido à modulação autonômica. Em saudáveis, o PRi pode atingir até 100 ms com o aumento da FC. ^295^ ^,^ ^598^ ^,^ ^599^

 O BAV de primeiro grau (esforço-induzido ou persistente) é definido como um PRi medido maior que o previsto para a FC. Equações de previsão do PRi médio pela FC: 

**– PRi médio para FC de 90 a 140 bpm = (-0,287xFC) + 182,9 ^600^**

**– PRi médio para FC de 60 a 160 bpm = (-0,351xFC) + 176,7 ^598^**

 Pacientes com bloqueio AV de primeiro grau acentuado (PRi ≥300 ms) no ECG basal podem apresentar durante o TE quadro semelhante ao da síndrome do marca-passo. Tais pacientes são mais propensos a se tornarem sintomáticos em exercícios leves ou moderados devido à falha de adaptação do PRi ao esforço. O PRi não diminui adequadamente com o aumento da FC, causando aproximação excessiva da sístole atrial à sístole ventricular precedente. Alguns desses pacientes sintomáticos, principalmente os com função de VE normal, podem se beneficiar do implante de marca-passo com estimulação de dupla câmara (GR-NE: IIa-B). ^601^ ^,^ ^602^

 O BAV de primeiro grau pode ocorrer no final do esforço ou na recuperação, particularmente na doença oculta do nó AV. Pode estar associado a medicamentos (digital, betabloqueadores, alguns bloqueadores dos canais de cálcio etc.) ou a condições que prolongam o tempo de condução AV (miocardite, doença de Chagas etc.). ^439^

 O FINCAVAS ( *Finnish Cardiovascular Study* ), com 1.979 pacientes submetidos a TE (seguimento médio 47 meses), demonstrou que o BAV de primeiro grau no 2º minuto da recuperação associou-se ao risco de mortalidade CV (RR contínua: 1,29, p=0,006; RR dicotomizada: 2,41, p=0,045). ^603^

**4.17.1.2. Bloqueio Atrioventricular de Segundo Grau Tipo I (Mobitz I)**

 Caracteriza-se por alentecimento gradativo da condução AV (fenômeno de Wenckebach) com aumento progressivo do PRi até que a condução AV seja bloqueada. A frequência de bloqueio pode ser variável e ocorrer repetição de ciclos. ^604^

 A presença de BAV Mobitz I no ECG basal em assintomáticos saudáveis não contraindica o TE e geralmente ocorre a normalização da condução AV. Em cardiopatas assintomáticos, deve-se avaliar o benefício de realização do exame. ^605^ ^-^ ^607^

 A manutenção BAV Mobitz I ou seu aparecimento durante o esforço são considerados critérios de interrupção do esforço quando apresentar: sintomas de baixo débito cardíaco ou angina; aumento do número de batimentos bloqueados; redução da FC com a progressão do esforço. ^6^ ^,^ ^608^ ^-^ ^610^

 O BAV Mobitz I com complexos QRS de duração ≥120 ms está associado a bloqueio infranodal AV em 30% a 40% dos pacientes, tendo o mesmo significado prognóstico observado no bloqueio AV de segundo grau tipo II, pois ambos indicam doença grave do sistema His-Purkinje. ^131^

**4.17.1.3. Bloqueio Atrioventricular de Segundo Grau Tipo II (Mobitz II)**

 No BAV de segundo grau tipo II (BAV Mobitz II), ocorre claudicação súbita da condução AV, sendo a localização do bloqueio na região intra/infra-His-Purkinje. ^611^

 A presença de BAV Mobitz II no ECG basal é critério de contraindicação do TE por estar associado à doença grave no sistema de condução cardíaco e outras cardiopatias. ^606^

 O BAV Mobitz II esforço-induzido é critério de interrupção do esforço por interferir na manutenção do débito cardíaco. ^612^ ^,^ ^613^ Geralmente está associado à DAC ou à estenose da valva aórtica e pode evoluir para BAVT. ^614^ ^,^ ^615^

**4.17.1.4. Bloqueio Atrioventricular Tipo 2:1 / Bloqueio Atrioventricular Avançado ou de Alto Grau / Bloqueio Atrioventricular de Terceiro Grau ou Total**

 No BAV tipo 2:1, para cada dois batimentos de origem atrial, um é conduzido e despolariza o ventrículo, e o outro é bloqueado com manutenção de intervalos PP constantes (excluindo o diagnóstico de extrassístoles atriais bloqueadas). BAV tipo 2:1 esforço-induzido é incomum e associa-se à queda do débito cardíaco com possibilidade de dispneia e síncope. A maioria dos pacientes tem histórico de sintomas, e o TE pode ser indicado na investigação e distinção de bloqueio nodal AV e infranodal. O BAV tipo 2:1 pode ser precedido por período de BAV Mobitz tipo I ou tipo II. O BAV tipo 2:1 desencadeado pelo aumento da FC (inclusive aos esforços) geralmente está associado à doença do sistema His-Purkinje. ^612^ ^,^ ^616^ ^-^ ^618^

 No BAV avançado ou de alto grau, existe condução AV em menos da metade dos batimentos atriais, sendo o bloqueio em proporção 3:1, 4:1 ou maior. A presença de condução AV é notada pelo intervalo PR constante em cada batimento que gera um QRS. A maior parte dos BAVs avançados esforço-induzidos localiza-se na região intra/infra-His. ^619^ ^,^ ^620^

 No BAV de terceiro grau ou total (BAVT), não existe correlação entre a atividade elétrica atrial e a ventricular, ocorrendo ondas P bloqueadas que não despolarizaram os ventrículos. Um foco abaixo da região de bloqueio assume o ritmo ventricular. A frequência do ritmo atrial geralmente é maior que a do ritmo de escape. BAVT de origem supra-hissiana apresentam QRS do escape ventricular semelhante ao do ECG basal, enquanto nos de origem infra-hissiana os complexos QRS são largos. ^621^

 BAVT adquirido é uma contraindicação ao TE porque o aumento da atividade simpática sem o correspondente aumento efetivo da FC pode resultar em arritmias ventriculares complexas e complicações graves. 

 O BAVT esforço-induzido é incomum e pode estar associado à isquemia transitória ou doença degenerativa grave do sistema de condução. Caso ocorra, há indicação absoluta de interrupção do esforço. ^207^ ^,^ ^208^ ^,^ ^622^

 O BAVT congênito tem prevalência de 1 por 15.000 a 20.000 nascidos vivos (60% mulheres). ^623^ O TE pode ser empregado para ajudar a documentar sintomatologia, avaliar aumento da resposta do escape ventricular, determinar eventual ocorrência de ectopias e a repercussão hemodinâmica do BAVT. BAVT localizado no sistema His-Purkinje apresentou pior prognóstico. A ocorrência de ectopia ventricular esforço-induzida associou-se ao aumento de risco de morte súbita. ^624^

 O TE pode ser realizado em indivíduos com BAVT congênito se não houver coexistência de doenças (congênitas ou não) que reduzam a segurança do TE. 

Particularidades do TE no BAVT congênito:

 – Muitos pacientes podem apresentar capacidade funcional normal. Não devem ser utilizadas equações de predição de VO _2_ max previsto e FCmax prevista. A ectopia esforço-induzida é frequente (50% a 70% dos pacientes). ^625^ ^,^ ^626^

 – A evolução natural do BAVT congênito consiste no declínio progressivo das frequências ventriculares ao longo da vida. No ECG de repouso, entre 6 e 10 anos, observa-se FC média de 50 bpm; entre 16 e 20 anos, de 45 bpm; e acima de 40 anos, de 38 bpm. Adultos com BAVT congênito apresentaram 8% de morte súbita cardíaca como primeiro sintoma do BAVT. Fadiga, dispneia, tontura e ectopias ventriculares esforço-induzidas foram responsáveis por 26,5% dos implantes de marca-passo. ^627^

### 4.17.2. Distúrbios da Condução Intraventricular

 Os distúrbios de condução intraventricular (bloqueios) podem ser preexistentes ao TE, desenvolver-se ou desaparecer com o esforço, sendo variadas a sua repercussão clínica e associação a cardiopatias. 

 Os critérios eletrocardiográficos de diagnóstico dos distúrbios da condução intraventricular encontram-se na Diretriz da Sociedade Brasileira de Cardiologia sobre a Análise e Emissão de Laudos Eletrocardiográficos – 2022. ^295^

**4.17.2.1. Bloqueio de Ramo Esquerdo**

 O bloqueio de ramo esquerdo (BRE) é raro em pacientes <50 anos e quase nunca ocorre em <35 anos, sugerindo tratar-se de um distúrbio adquirido, secundário a DCV. Estudos populacionais demonstraram que a prevalência aumenta de maneira constante a partir dos 50 anos (<1%) atingindo 6% aos 80 anos. ^295^ ^,^ ^628^ ^,^ ^629^

**4.17.2.1.1. Bloqueio do Ramo Esquerdo Preexistente**

 O BRE no ECG basal é uma limitação para a análise do ISTs, pois geralmente não está associado à isquemia miocárdica, reduzindo a especificidade e a acurácia do TE. ^628^ ^,^ ^630^ ^-^ ^632^ Em indivíduos normais e saudáveis com BRE, o ISTs no TE pode atingir até 10 mm. O TE pode ser realizado para investigação de sintomas, e a análise de todas as outras variáveis do exame não se encontra prejudicada. ^12^ ^,^ ^628^

 Na vigência de BRE, sugere-se aplicar o escore de diagnóstico de IAM (Sgarbossa) de modo a identificar possível evento adverso agudo durante o TE, em que: ^633^ ^,^ ^634^

 – Supradesnivelamento do segmento ST (SSST) ≥1 mm concordante com o QRS (em derivação com QRS predominantemente positivo) – pontuação = 5. 

– ISTs ≥1 mm em V1, V2 ou V3 – pontuação = 3.

 – SSST ≥5 mm discordante com o QRS (em derivação com QRS predominantemente negativo) – pontuação = 2. 

 Interpretação da pontuação do Escore de Sgarbossa em relação ao TE: 

– ≥3 = IAM na presença de BRE.

 – <3 e >0 = não descarta IAM. Em pacientes de alto risco ou com sintomatologia, realizar avaliação adicional. 

– 0 = comportamento esperado do BRE durante o TE.

 Para diagnosticar a complicação de IAM em presença de BRE durante o TE, também pode-se utilizar: escore de Sgarbossa modificado; algoritmo de Barcelona; critérios de Smith. ^635^ ^-^ ^638^

 Particularidades do TE em pacientes com BRE preexistente: 

 – Acarreta importantes efeitos hemodinâmicos: ativação miocárdica assíncrona; comprometimento da função sistólica e diastólica; redução da fração de ejeção. ^639^ ^-^ ^641^

 – Na miocarcardiopatia dilatada com BRE, observa-se um VO _2_ pico significativamente menor do que VO _2_ max previsto (≈33% inferior; p<0,001). O BRE e a duração do QRS foram preditores de baixa tolerância ao esforço. ^642^

 – O desaparecimento do BRE durante o esforço é situação muito rara geralmente associada a bloqueio de ramo temporário (persiste por dias ou meses) ou transitório (dura apenas segundos ou horas). É considerada observação fortuita e sua relação com o esforço é incerta. Tais bloqueios podem estar associados à cardiopatia ou a condições como doença hipertensiva, febre reumática, embolia pulmonar, hipercalemia e tireotoxicose. ^643^ ^,^ ^644^

**4.17.2.1.2. Bloqueio do Ramo Esquerdo Esforço-induzido**

 O BRE esforço-induzido (BRE-EI) ocorre em aproximadamente 0,4-0,5% dos pacientes submetidos a TE. ^202^ ^,^ ^203^ ^,^ ^645^ Seu mecanismo permanece incerto, podendo estar associado a doenças valvares, miocardiopatias, cardiopatias congênitas, defeito primário do sistema de condução, DAC ou mesmo em pacientes sem doenças detectadas. Estudo longitudinal demonstrou que a DAC e a IC foram as causas mais prevalentes. ^203^

 O BRE-EI transitório pode causar dissincronia ventricular esquerda e alterações secundárias no padrão de enchimento reversíveis. ^646^ ^,^ ^647^ Além disso, pacientes com BRE-EI apresentam risco aumentado de desenvolver BRE permanente, disfunção ventricular e raramente BAVT com necessidade de implante de marca-passo. ^203^

 As principais formas de manifestações clínica do BRE-EI são: 

 1) Assintomático, tanto no aparecimento quanto no momento de desaparecimento. 

 2) Dor torácica de início abrupto (normalmente localizada e sem irradiação), intensidade variada (de desconforto a dor severa), simultânea ao aparecimento e melhora concomitante ao desaparecimento e com ECG normal antes e após o BRE. Uma minoria dos pacientes pode apresentar melhora da dor antes do desaparecimento do BRE. 

 3) Dor precordial típica antes do aparecimento do BRE, que não melhora com seu desaparecimento, geralmente está associada a DAC (principalmente com ISTs precedendo o BRE). ^643^ ^,^ ^648^

 A síndrome de BRE doloroso é constituída por dor torácica associada ao BRE-EI (descrita no item 2, anteriormente), em paciente com função ventricular normal, sem outras possíveis causas e com relação da amplitude das ondas S/T <1,8 (derivações precordiais e parede inferior). O TE tem papel fundamental para o seu diagnóstico. ^648^ ^-^ ^652^

 Particularidades do BRE-EI e das alterações associadas: 

 – No BRE intermitente ou após o seu desaparecimento, comumente ocorrem inversões profundas e simétricas da onda T (em V1 a V4) nos batimentos normalmente conduzidos. São consequência do próprio BRE constituindo fenômeno elétrico secundário à ativação anormal e não devem ser interpretadas como isquemia miocárdica. ^653^ ^-^ ^657^

 – Durante o período com BRE, a acurácia relacionada às alterações do segmento ST quanto a isquemia miocárdica encontra-se prejudicada. ^631^

 – A ocorrência de ISTs precedendo o início do BRE deve ser valorizada, e sua interpretação não está prejudicada, pois caso preencha critérios de isquemia miocárdica, geralmente está associada a DAC. ^648^

 – Relatos e séries de casos demonstraram que o início do BRE em FC ≤125 bpm tem sido fortemente correlacionado com a presença de DAC obstrutiva. ^649^ ^,^ ^658^ Quando o BRE-EI ocorreu em FC >125/min, geralmente observou-se cineangiocoronariografia (CAT) normal e um melhor prognóstico. ^202^ ^,^ ^659^

 – Coorte com 25 pacientes com BRE-EI (entre 16.500 TE) identificou um VPP de 72% para DAC e, quando em FC <120 bpm, associou-se à estenose proximal de DA. ^660^

 – A síndrome do BRE doloroso e o BRE-EI em FC >125 bpm geralmente não estão associados à DAC obstrutiva. ^648^ ^-^ ^652^

 – Em estudo com 9.318 pacientes (seguimento médio 6,9 anos), 20 pacientes apresentaram BRE-EI. Os pacientes com DAC (60%) apresentaram pior prognóstico (risco aumentado de morte e IAM). O risco de desenvolvimento de BRE permanente e BAVT foi semelhante nos pacientes com e sem DAC. ^648^

**4.17.2.2. Bloqueios Divisionais do Ramo Esquerdo**

 Os bloqueios divisionais do ramo esquerdo (BDRE) no ECG basal não impossibilitam a análise de alterações da repolarização ventricular esforço-induzidas quanto à isquemia, entretanto podem diminuir a acurácia do exame. ^661^ ^,^ ^662^

 Os BDRE esforço-induzidos (BDRE-EI) são raros. O mecanismo mais aceito para sua ocorrência é a condução lenta induzida por isquemia em fibras no feixe esquerdo e suas subdivisões, ou nas fibras miocárdicas de Purkinje. ^663^

Particularidades BDRE-EI:

 – Apresentam alta correlação não só com a existência de DAC, mas com a extensão e a gravidade da doença. ^661^ ^,^ ^664^ ^-^ ^667^

 – O posteroinferior esquerdo (BDPI) esforço-induzido tem sido relacionado à DAC em coronária direita ou multiarterial. ^668^

 – O anterossuperior esquerdo (BDAS) e anteromedial esquerdo (BDAM) esforço-induzidos estão associados à DAC em tronco de coronária esquerda ou DA. ^669^

 – No BDAS esforço-induzido, inúmeros relatos de caso documentaram a reversibilidade desse bloqueio após o tratamento intervencionista da obstrução coronariana. ^664^ ^,^ ^665^ ^,^ ^670^

**4.17.2.3. Bloqueio de Ramo Direito**

**4.17.2.3.1. Bloqueio de Ramo Direito Preexistente**

 O bloqueio de ramo direito (BRD) ocorre em 0,2% a 3% da população geral. A prevalência aumenta com a idade, sendo maior em homens (≈14,3% em homens >80 anos). ^671^

 O BRD isolado é geralmente benigno, exceto em determinadas cardiopatias (p. ex., miocardiopatia, DAC ou IC), quando se associa a maior mortalidade CV. BRD isolado é comum em indivíduos aparentemente saudáveis. ^672^ O principal diagnóstico diferencial do ECG com BRD é a síndrome de Brugada. ^673^ ^-^ ^675^

 O BRD no ECG basal invalida a interpretação de alterações do ISTs ao esforço apenas nas derivações de V1 a V3. Geralmente está presente no repouso e aumenta no esforço, sem relação com DAC. Nas demais derivações, a quantificação e a interpretação de ISTE permitem o diagnóstico de isquemia esforço-induzida. ^645^ ^,^ ^676^ ^,^ ^677^

Particularidades do BRD preexistente:

 – Estudo com 3.609 pacientes submetidos ao TE identificou 163 (4,5%) com BRD, dos quais 133 foram seguidos (36% com IAM). O TE apresentou sensibilidade de 27%, especificidade de 87% e acurácia de 62% para DAC. Durante seguimento, observou mortalidade anual de 10%. ^678^

 – Estudo com 23.026 pacientes sem diagnóstico de DCV, 220 (0,96%) com BRD nos quais observou-se maior mortalidade por todas as causas (RR: 1,5; IC 95%: 1,1-2,0; p=0,0058) e por DCV (RR: 1,7, IC 95%: 1,1-2,8; p=0,0178). Os pacientes com BRD apresentaram menor tolerância ao esforço, recuperação mais lenta da FC e maior ocorrência de dispneia. ^673^

 – Coorte com 7.073 adultos submetidos ao TE associado à cintilografia de perfusão miocárdica (seguimento médio 6,7 anos) demonstrou que a mortalidade em 190 pacientes com BRD foi maior que nos sem bloqueio (24% *vs.* 11%, respectivamente; RR: 1,5; IC 95%: 1,1-2,1; p=0,007), mesmo após ajuste para aptidão cardiorrespiratória, defeitos de perfusão nuclear e outros fatores de risco. O bloqueio divisional do ramo direito não foi associado à mortalidade. ^679^

 – Existem poucos relatos de casos na literatura de desaparecimento do BRD preexistente durante o esforço, e o mecanismo envolvido não é claro. ^680^ ^-^ ^682^

**4.17.2.3.2. Bloqueio de Ramo Direito Esforço-induzido**

 O BRD esforço-induzido (BRD-EI) ocorre em aproximadamente 0,25% dos pacientes submetidos ao TE, sendo menos frequente que o BRE-EI. Geralmente está associado à DAC. ^152^ ^,^ ^202^ ^-^ ^204^

Particularidades do BRD-EI:

 – A análise das alterações de isquemia durante o período com BRD é semelhante à descrita para pacientes com BRD preexistente. 

 – Duas coortes avaliando pacientes com BRD-EI identificaram alta prevalência de DAC nesses pacientes, sejam lesões uni ou multiarteriais. ^645^ ^,^ ^660^

 – Em coorte com 8.047 pacientes (seguimento médio de 8,8 anos), os 23 pacientes com BRD-EI apresentaram maior prevalência de DAC e IC e maior risco de morte. ^204^

 – Em coorte com 3.974 homens (idade média de 57,5 anos; seguimento médio 5,9 anos), observou-se 1,9% de BRD-EI, que foi associado a maior risco de morte por todas as causas (p<0,001). ^306^

**4.17.3. Distúrbios da Formação do Impulso**

 É comum a ocorrência de anormalidades do ritmo cardíaco (ARC) durante o TE em pacientes com e sem DCV. Frequentemente, as arritmias são isoladas, transitórias, episódicas, assintomáticas e geralmente não representam risco de eventos CV. Costumam apresentar grande variabilidade espontânea e circadiana, o que dificulta sua reprodutibilidade. ^299^

 Em presença de ARC, sugere-se a utilização do registro em tempo total do ECG (registro contínuo) para diagnóstico, quantificação e documentação das arritmias. 

**Figura 15:**
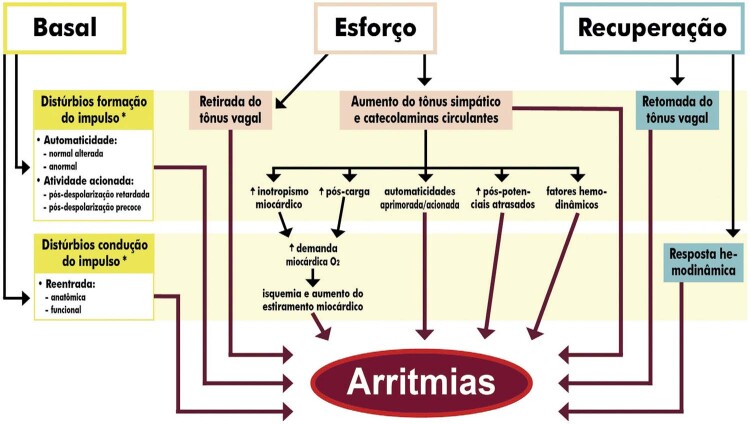
– Principais mecanismos e fatores envolvidos na arritmogênese durante o TE. *Permanecem como mecanismos durante todas as etapas do TE. ↑ = aumento. O 2 : oxigênio.

 Em repouso, os principais mecanismos envolvidos na arritmogênese são: reentrada, automaticidade aprimorada/acionada e pós-potencial atrasado. Outros fatores: anormalidades eletrolíticas, alterações no pH, hipóxia, fatores hemodinâmicos (pré e pós-carga, distensão parede VE etc.), modulação autonômica, catecolaminas circulantes, interações medicamentosas e isquemia miocárdica. O exercício pode ser gatilho devido à retirada simultânea da atividade vagal, aumento da atividade simpática, alterações da automaticidade cardíaca e aumento do consumo de oxigênio miocárdico. Na recuperação, ocorrem a retomada súbita do tônus vagal e alterações hemodinâmicas que também podem precipitar arritmias ( Figura 15 ). ^63^ ^,^ ^133^ ^,^ ^134^ ^,^ ^142^

 No pré-teste, recomenda-se pesquisar possíveis fatores precipitantes e agravantes das arritmias, tais como esforço físico, ingestão excessiva de cafeína e de álcool, tabagismo, uso de drogas recreativas e hipertireoidismo. Caso ocorra ARC durante o TE, recomenda-se correlacionar com os dados do pré-teste e possíveis fatores desencadeantes relacionados ao esforço (p. ex., isquemia). ^63^ ^,^ ^133^ ^,^ ^134^ ^,^ ^683^

**4.17.3.1. Arritmias Ventriculares**

 O TE é útil para fins de investigação de sintomas sugestivos de arritmia, diagnóstico, comportamento (potencialização e supressão) e prognóstico em pacientes selecionados. ^684^

 A extrassístole ventricular (EV) apresenta-se como batimento ectópico ventricular, prematuro em relação ao intervalo RR anterior, geralmente com pausa pós-extrassistólica. Caso não ocorra modificação na duração do intervalo RR, a EV é chamada de EV interpolada. ^63^ ^,^ ^188^ ^,^ ^371^ São arritmias comuns na prática clínica que aumentam com a idade e DCV. A prevalência em repouso na população aparentemente saudável é de 1% a 4% e, no TE, de 5% a 34%. Em pacientes com miocardiopatia, a incidência no TE pode atingir ≈90%. ^684^ ^,^ ^685^

A EV pode ser classificada conforme:

 – Morfologia: monomórfica e polimórfica (mais de uma morfologia). 

 – Inter-relação com os batimentos sinusais e outras EVs: 

• Isolada: batimento ectópico ventricular único.

 • Pareada (pares ventriculares): dois batimentos ectópicos ventriculares, de mesma morfologia ou diferentes, com intervalo de acoplamento fixo ou variável. 

 • Em salva: são três ectopias ventriculares seguidas, em série, equivalentes à TVNS. 

 • Bigeminada: são EVs que alternam com ritmo sinusal na proporção de 1 EV para 1 batimento normal, de forma repetitiva por períodos curtos ou prolongados. 

 • Trigeminada: são EVs que alternam repetitivamente com o ritmo sinusal na proporção de 1 EV para 2 batimentos normais. 

 • Quadrigeminada: são EVs que alternam repetitivamente com o ritmo sinusal na proporção de 1 EV para 3 batimentos normais. 

 – Quanto à frequência. A classificação de Lown e Wolf permite quantificar a arritmia, sendo útil para definição de gravidade (as classes I e II necessitam de monitorização do ECG pelo método Holter): 

0: Ausência EV.

I: <30 EVs/h.

II: ≥30 EVs/h.

III: EVs polimórficas.

IVa: EVs pareadas (pares ventriculares).

 IVb: taquicardia ventricular (3 ou mais EVs consecutivas). 

 V: intervalo de acoplamento curto (fenômeno R sobre T). 

 Na população geral, as EVs são definidas como frequentes quando se observa pelo menos 1 EV em ECG basal de 12 derivações ou ≥30 EVs/h (Classe II de Lown e Wolf – em Holter), estando associadas a aumento do risco CV e mortalidade. ^63^ ^,^ ^191^

 A classificação quanto a morfologia, duração do QRS e inter-relação com os batimentos sinusais e outras EVs pode ser utilizada tanto no ECG basal quanto no TE. 

 A taquicardia ventricular (TV) corresponde a pelo menos três batimentos ventriculares sucessivos com FC >100 bpm. Classifica-se de acordo com: 

 – Morfologia dos batimentos: TV monomórfica (TVM) com morfologia uniforme e TV polimórfica (TVP) com três ou mais morfologias. 

 – Duração: não sustentada (TVNS) se <30 segundos e TV sustentada (TVS) se ≥30 segundos. 

 – Sintomas e repercussão hemodinâmica: ausentes ou presentes. 

 Na presença de taquicardias de QRS largo (>120 ms), pode ser necessária a utilização de algoritmos (Brugada ou Vereckei) para o diagnóstico diferencial de taquicardia supraventricular com aberrância de condução. ^686^

 A TV tipo *torsades de pointes* apresenta QRS largo, polimórfico, “girando” em torno da linha de base, precedida por ciclos longo/curto, relacionada com intervalo QTc longo (congênito ou secundário a fármacos e distúrbios eletrolíticos) e geralmente autolimitada. 

 Na TV bidirecional, ocorre uma TV com morfologia de bloqueio de ramo direito (raramente BRE) associada a bloqueio alternado das divisões anterossuperior e posteroinferior do ramo esquerdo. O aspecto bidirecional se deve à observação de um batimento ventricular com QRS positivo seguido de outro com QRS negativo, sucessivamente. Geralmente está associada a miocardiopatia avançada grave, quadros de intoxicação digitálica e taquicardia catecolaminérgica familiar. 

 A arritmia ventricular esforço-induzida (AV-EI) é definida como quaisquer complexos ventriculares prematuros ou taquicardia ventricular durante o esforço e a recuperação. Quando frequente, é marcador de mau prognóstico. ^687^

Principais definições de AV-EI frequente:

 1) Ocorrência de ≥7 EVs/minuto, bigeminismo ou trigeminismo ventricular, pares ou salvas, taquicardia ventricular, flutter ventricular, *torsades de pointes* ou fibrilação ventricular. ^192^

 2) Quando em qualquer período de 30 segundos no TE, as arritmias ventriculares corresponderem a mais de 10% dos batimentos ou ocorrer TV (com ≥3 EVs consecutivas). ^688^ ^,^ ^689^

 Quando ocorrer AV-EI, recomenda-se informar na conclusão sua forma de apresentação e se é frequente ou não. 

 Sintomas associados a AV-EI consistem em queixa falha do batimento (palpitação), sensação de aceleração, desconforto torácico, fadiga e tontura. Os sopros de ejeção existentes podem ser exacerbados em virtude do maior volume sistólico e da força contrátil após pausas compensatórias. Nas TVs, a sintomatologia costuma ser mais frequente e intensa. Quando sustentada, a repercussão hemodinâmica é comum e associa-se a queixas de desconforto ou pressão precordial, dor típica, dispneia, palpitações, diaforese, tontura, náusea, pré-síncope e síncope. ^690^

 Particularidades das arritmias ventriculares ao TE: 

 – AV-EI frequente em qualquer das fases do TE está associada a maior risco de morte por todas as causas e CV, principalmente quando ocorrer na recuperação, sendo ainda maior caso ocorra no esforço e recuperação. ^191^ ^-^ ^194^ ^,^ ^196^ ^,^ ^689^ ^,^ ^691^

 – São de pior prognóstico: EVs polimórficas; EVs com morfologia de via de saída VE; EVs com densidade crescente ao esforço; EVs com intervalo de acoplamento curto. ^684^ ^,^ ^692^

 – Em estudo com 302 pacientes (idade média 54 anos, 152 homens), 22% apresentaram AV-EI frequentes associadas a maior ocorrência de anormalidades perfusionais e ISTs, principalmente em homens (67% *vs.* 38%; p<0,05). ^693^

 – Na população aparentemente saudável (incluindo atletas), as EVs isoladas em repouso costumam apresentar redução com o esforço, sendo geralmente de bom prognóstico. ^694^ ^-^ ^696^

 – Em atletas com TV-EI (com ≥3 batimentos a ≥120 bpm, sustentada ou não), recomenda-se investigação adicional para investigação diagnóstica e de risco. ^136^ ^,^ ^694^ ^,^ ^695^

 – EVs e AV-EI nas condições arrítmicas familiares (p. ex., síndrome do QT longo) e sensíveis às catecolaminas (p. ex., TV da via de saída do ventrículo direito). ^63^ ^,^ ^130^ ^,^ ^188^

**4.17.3.2. Arritmias Supraventriculares**

 As arritmias supraventriculares (AS) são relativamente comuns, muitas vezes repetitivas, ocasionalmente persistentes e raramente fatais. Os principais fatores precipitantes de AS são: idade (mais comum em idosos), sexo (mais comum no sexo feminino) e comorbidades associadas (p. ex., hipertensão, valvopatia e miocardiopatias). ^697^ ^-^ ^700^

 A extrassístole supraventricular isolada (ESV) é um batimento ectópico atrial ou juncional precoce que é seguido de despolarização ventricular com morfologia e duração semelhante aos batimentos sinusais precedentes. A taquicardia atrial (TA) constitui ritmo atrial originado em região diversa do nó sinusal, caracterizada pela presença de onda P distinta da sinusal com frequência atrial >100 bpm. É comum a ocorrência de condução AV variável. A taquicardia atrial multifocal (TAMF) apresenta as mesmas características do ritmo atrial multifocal, com frequência atrial >100 bpm. 

 A taquicardia por reentrada nodal (TRN) comum utiliza o nó AV como parte fundamental do seu circuito e pelo mecanismo de reentrada nodal. Em geral, a onda de ativação atrial está dentro do QRS e não é observada. Nos casos de TRN com QRS alargado, faz-se necessário o diagnóstico diferencial com taquicardias de origem ventricular. Na TRN atípica (incomum), o sentido de ativação é inverso (atrial retrógrada) com intervalo RP maior que o PR. 

 A taquicardia por reentrada atrioventricular (TRAV) ortodrômica utiliza o sistema de condução normal no sentido anterógrado e uma via acessória no sentido retrógrado. O QRS é estreito e a onda P retrógrada com morfologia diversa dependendo da localização da via acessória e intervalo RP >80 ms. 

 As taquicardias supraventriculares podem ser classificadas baseadas no RP: 

 – RP’ curto (habitualmente até 120-140 ms): associadas a TRN forma comum e taquicardia por reentrada via feixe anômalo. 

 – RP’ longo: associadas a TRN forma incomum, TA e taquicardia de Coumel (reentrada por feixe anômalo de condução retrógrada única). 

 As AS podem se apresentar com complexo QRS alargado nas seguintes situações: 

 – Aberrância de condução, em que um estímulo supraventricular (normal ou extrassistólico) que encontra dificuldade de propagação regional no sistema de condução, gerando um QRS com morfologia de bloqueio de ramo. 

 – Taquicardia supraventricular com aberrância de condução, que é a denominação genérica para as taquicardias supraventriculares de RP’ curto ou longo. 

 As arritmias supraventriculares (ESV, fibrilação atrial, flutter atrial, TPSV) são comumente induzidas pelo esforço e encontradas em até 10% dos TE de pacientes aparentemente saudáveis e em até 25% daqueles com DAC conhecida ou suspeita. ^189^ ^,^ ^197^

 As ESVs isoladas são frequentes no TE com incidência de 4% a 18%. Normalmente são assintomáticas, inclusive quando esforço-induzidas. As ESVs não apresentam correlação com isquemia miocárdica ou risco de mortalidade CV/IAM. ^189^ ^,^ ^197^ ^,^ ^198^

 ESV isoladas, arritmias sinusais com períodos de bradicardia sinusal e ritmo de marca-passo atrial mutável são relativamente comuns no início do esforço e da recuperação, tanto em pacientes aparentemente saudáveis quanto em cardiopatas. ESVs no ECG basal tendem a se tornar progressivamente menos frequentes com o esforço, sendo geralmente benignas. ^6^ ^,^ ^142^ ^,^ ^699^ Pacientes idosos com ESVs frequentes ao esforço (>5/estágio) apresentaram maior risco de FA/FluA (RR: 15,23; IC 95%: 4,59-50,56; p<0,001). ^199^ ^,^ ^200^

 A TRAV é mais comum na meia-idade e idosos, enquanto, em adolescentes, observa-se prevalência semelhante de TRAV e TRN. Ambas são raras no TE, apresentam início e término súbitos, FC entre 150 e 250 bpm e geralmente necessitam de adenosina para sua interrupção. ^129^ ^,^ ^190^

 A incidência de pré-excitação manifesta ou padrão WPW na população geral é de 0,1% a 0,3%, sendo a ocorrência de TPSV-EI muito rara. ^129^ ^,^ ^701^ ^,^ ^702^

 A incidência de TPSV-EI varia de 3,4% a 15% em pacientes com AS paroxísticas. ^185^ ^,^ ^197^ É mais comum em homens idosos, geralmente assintomática, não sustentada, ocorre perto do pico do esforço, não está associada a isquemia esforço-induzida e mortalidade CV. ^189^ Entretanto, a TPSV-EI geralmente associa-se a palpitação, desconforto torácico, mal-estar, tontura, pré-síncope e dispneia. Mais raramente, pode ocorrer intolerância súbita ao esforço, hipotensão, síncope, sinais de IC e choque. ^129^ ^,^ ^703^ ^-^ ^705^

 Particularidades das arritmias supraventriculares ao TE: 

 – Indivíduos com TPSV-EI (salva de 3 ou mais batimentos) apresentaram maior risco de FA no seguimento médio de 5,7 anos (RR: 7,6; p<0,001). ^189^

 – Durante episódios de TPSV, pode ocorrer ISTs que geralmente não estão associados à isquemia miocárdica. ^706^ ^,^ ^707^

 – As AS-EI estão mais frequentemente relacionadas à idade avançada, DPOC, ingestão recente de álcool ou ingestão excessiva de cafeína. ^6^

**4.17.3.3. Fibrilação Atrial/Flutter Atrial**

 Fibrilação atrial (FA) é uma atividade elétrica atrial desorganizada (ondas “f”) com frequência atrial entre 450 e 700 bpm e resposta ventricular variável. ^371^ Em relação ao repouso, considerar ritmo de FC com: 

– Baixa resposta ventricular, quando a FC ≤50 bpm.

 – Controle adequado da FC, quando FC entre 51 e 89 bpm. 

 – Controle leniente (ou inadequado), quando a FC estiver entre 90 e 110 bpm. 

 – Resposta ventricular elevada, quando a FC >110 bpm. ^295^

 Critérios de classificação da FA: ^119^

 – Paroxística: um episódio de FA que se converte espontaneamente ao ritmo sinusal normal em até 7 dias. 

 – Persistente: paciente requer cardioversão elétrica ou química/medicamentosa para restaurar o ritmo sinusal. 

 – Permanente ou crônica: presente por >6 meses ou quando o paciente e o médico decidem não tentar mais restaurar o ritmo sinusal. 

 Flutter atrial (FluA) é uma atividade elétrica atrial organizada (ondas “F”) dividido em: ^133^ ^,^ ^371^

 – Tipo I (comum ou típico) com ativação no sentido anti-horário, frequência atrial entre 240 e 340 bpm e ondas “F” com aspecto em dentes de serrote (negativas nas derivações inferiores e positivas em V1). 

 – Tipo II (atípico ou incomum) com ativação no sentido horário, frequência atrial entre 340 e 430 bpm, graus variados de bloqueio AV e ondas “F” alargadas e positivas nas derivações inferiores. 

 Na FA e no FluA, a determinação da FC deve ser feita a partir de um traçado de ECG de 6s. Geralmente o esforço não provoca aumento na frequência das ondas atriais e o aumento da frequência ventricular depende da condução atrioventricular. Na FA (persistente e crônica) e no FluA crônico, o TE é útil na avaliação de sintomas e reposta da FC, para ajustes terapêuticos e prescrição de exercício/reabilitação. ^708^ ^-^ ^712^

 Preferencialmente, os pacientes devem manter as medicações para o controle de ritmo e/ou frequência ventricular e anticoagulantes para realização do TE. ^133^

 Nos pacientes com FA permanente não tratados, a FC no TE costuma variar de 90 a 170 bpm. Uma FA com FC <60 bpm no ECG de repouso pode estar associada a doença do nó AV, síndrome do nódulo sinusal e uso de drogas que afetam a FC (betabloqueadores e antiarrítmicos). ^133^

 Os pacientes com FA podem ser exercitados com segurança até o esforço máximo, limitado por sintomas, na ausência de outras indicações formais de interrupção. Considera-se adequado o controle medicamentoso da FC quando a resposta cronotrópica for semelhante à dos pacientes em ritmo sinusal. ^708^ ^-^ ^711^ Aumentos acentuados da FC, atingindo ou superando a FC submáxima já no primeiro estágio do esforço, bem como FCpico >110% da FC máxima prevista (para a idade), necessitam de adequação da terapia farmacológica, por serem preditores de IC e reduzirem a capacidade física. ^159^ ^,^ ^713^ ^-^ ^716^ Nos pacientes com IC e FA permanente, o ΔFC demonstrou estar relacionado ao desempenho no esforço e morbimortalidade. ^158^ ^,^ ^717^ ^,^ ^718^

 Na vigência de FA, a avaliação de isquemia fica prejudicada, pois as alterações do segmento ST podem ser decorrentes da própria arritmia (baixo VPP). Entretanto, ausência de ISTE confere elevado VPN para isquemia. ^713^ ^,^ ^719^

 A FA permanente, independentemente da doença de base (IC, hipertensão, cardiopatia isquêmica, miocardiopatia ou doença valvar), relacionou-se a VO _2_ max abaixo do previsto para a idade. ^715^ ^,^ ^717^

 A conversão para o ritmo sinusal reduziu a FCpico (≈40 bpm) e melhorou a aptidão cardiorrespiratória (≈15%). ^720^

 No FluA crônico, geralmente ocorre modificação do padrão de condução AV com redução do grau de bloqueio e aumento da FC ao esforço. Os sintomas associados ao FluA (fadiga, dispneia, mal-estar) estão relacionados à FC elevada. Embora o FluA com condução AV 1:1 seja raro, é importante reconhecê-lo, pois pode precipitar rápido comprometimento hemodinâmico e síncope. No início da recuperação, geralmente observa-se BAV de segundo grau tipo 1 transitório, seguido de retorno ao padrão basal do ECG. Não é possível avaliar isquemia durante o FluA. ^721^ ^-^ ^723^

 FA e FluA esforço-induzidos (FA-EI e FluA-EI) são raros e ocorrem em <1% dos indivíduos. Durante o TE, pode ocorrer a modificação de FA-EI para FluA-EI e vice-versa, pois uma arritmia pode ser mecanismo desencadeador da outra. ^724^ Ocorrem em indivíduos aparentemente saudáveis, na doença cardíaca reumática, hipertireoidismo, síndrome de Wolff-Parkinson-White e cardiomiopatia. Ambas costumam causar repercussão hemodinâmica quando a resposta ventricular ao esforço for elevada. Na FA-EI, a avaliação de isquemia fica prejudicada pelo baixo VPP e inviabilizada no FluA-EI. ^201^ ^,^ ^725^

**4.17.3.4. Bradiarritmias/Incompetência Cronotrópica Crônica**

 Um grupo heterogêneo de indivíduos apresenta FC de repouso inadequadamente baixa, e a maioria é assintomática e desconhece a anormalidade. É comum em atletas de alto rendimento associada ao tônus vagal aumentado. Pode ser secundária ao uso de medicamentos (antiarrítmicos e betabloqueadores), doença intrínseca do tecido nodal (geralmente por isquemia) alterações degenerativas, miocardiopatia atrial e síndrome do nó sinusal. ^131^ ^,^ ^726^ ^,^ ^727^

 Nessa situação, o TE permite analisar a resposta cronotrópica ao estímulo simpático, correlacionar eventuais sintomas relacionados à bradicardia, podendo ser determinante no diagnóstico e terapêutica, incluindo indicação de implante de marca-passo definitivo. ^131^ ^,^ ^368^

 Nos pacientes com bradicardia sinusal não patológica submetidos ao TE, observa-se uma resposta cronotrópica normal, caracterizando-os como indivíduos vagotônicos. Entretanto, nos pacientes com bradicardia sinusal e ocorrência de uma resposta cronotrópica deprimida, geralmente observa-se disfunção do nó sinusal (DisNS). Alguns pacientes com DisNS podem atingir uma FCpico apropriada durante o esforço, mas podem ter uma aceleração muito lenta da FC nos estágios iniciais do protocolo ou apresentarem uma rápida desaceleração da FC no estágio inicial de recuperação. A DisNS pode desencadear sintomas de ICC e angina ao esforço. ^133^

 Nos pacientes com bradicardia sinusal não patológica submetidos ao TE, observa-se uma resposta cronotrópica normal, caracterizando-os como indivíduos vagotônicos. 

 Pacientes com disfunção do nó sinusal (DisNS) submetidos ao TE podem: apresentar bradicardia sinusal no repouso com ocorrência de resposta cronotrópica deprimida ao esforço; raramente apresentar aceleração muito lenta da FC nos estágios iniciais do protocolo, podendo atingir até a FCmax prevista; muito raramente, apresentarem isoladamente rápida desaceleração da FC no início da recuperação não associada ao bom condicionamento físico. A DisNS pode desencadear sintomas de ICC e angina ao esforço. ^133^

 Alguns pacientes com bradicardia sinusal esforço-induzida também podem apresentar síncope associada a uma queda profunda da PA devido ao reflexo de Bezold-Jarisch. ^205^ ^,^ ^206^

**4.17.3.5. Taquicardia Sinusal Inapropriada**

 A síndrome da taquicardia sinusal inapropriada (SindTSI) é um distúrbio crônico caracterizado por: ^133^ ^,^ ^728^

 – Aumento da FC sinusal desproporcional à demanda fisiológica: FC diurna em repouso >100 bpm; FC média no Holter de 24 horas >90 bpm; resposta exagerada da FC ao mínimo esforço físico ou estresse emocional. 

 – Ausência de outras causas que justifiquem a taquicardia sinusal. 

 – No ECG de repouso, durante a taquicardia sinusal, a onda P apresenta eixo e morfologia semelhantes ao do ritmo sinusal. 

 – Ocorrência de sintomas associados à taquicardia, tais como palpitações, fadiga, dispneia, intolerância ao exercício e ansiedade. 

 A maioria dos pacientes com SindTSI consiste em mulheres com ≈38±12 anos. Em uma população de meia-idade, a prevalência da SindTSI (sintomática ou assintomática) é de até 1,2%. Entretanto, na pós-COVID e COVID crônica, a prevalência pode atingir até 20% (idade 40,1±10 anos, 85% mulheres, 83% COVID-19 leve), com ausência de doença cardíaca estrutural, estado pró-inflamatório, lesão de miócitos ou hipóxia. ^729^ ^,^ ^730^

 O curso natural e o prognóstico da SindTSI são geralmente benignos e raramente ocorre cardiomiopatia induzida por taquicardia. Sua associação com condições psiquiátricas não é infrequente. O principal diagnóstico diferencial é com a síndrome de taquicardia postural que é um distúrbio do sistema nervoso autônomo associado à posição ortostática, em que se observa aumento da FC >30 bpm ou FC >120 bpm nos primeiros 10 minutos nessa posição. ^731^ ^,^ ^732^

 O TE é útil na avaliação da SindTSI, pois geralmente mostra aumento precoce e excessivo da FC em resposta a uma carga de esforço mínima (FC >130 bpm em 90 segundos de esforço no protocolo de Bruce) e/ou FCmax alcançada rapidamente. Essa resposta da FC é diferenciada da encontrada nos sedentários pela ocorrência de sintomas. ^133^ ^,^ ^733^ ^,^ ^734^

## 4.18. Avaliação Metabólica Indireta

###  4.18.1. VO 2 /METs 

 A estimativa da capacidade aeróbica deve, preferencialmente, ser descrita utilizando a quantidade de esforço realizado em METs ou o seu respectivo VO _2_ max estimado. Deve-se relatar o valor real alcançado e o respectivo valor percentual previsto. Recomenda-se não expressá-la em número de minutos de esforço ou estágio alcançado, pois essas formas dificultam a interpretação clínica e podem ter grande variação entre os protocolos. ^735^ ^,^ ^736^

 O cálculo do VO _2_ e sua conversão para METs, seus valores previstos por idade e sexo e sua significância prognóstica foram descritos anteriormente nesta diretriz. 

 O TE é indicado para determinar a tolerância ao esforço em indivíduos aparentemente saudáveis, com DCV (cardiopatia isquêmica, IC, miocardiopatias, valvopatias, arritmias, cardiopatias congênitas, DAP etc.) e comorbidades (p. ex., diabetes e DPOC). 

 Em pacientes adultos com DCV, considera-se como critério de intolerância ao esforço/baixo desempenho físico não atingir: 

 – Carga de trabalho de 5 METs (capacidade aeróbica estimada). ^737^

 – 15,0 mL/kg/min (capacidade aeróbica medida diretamente pelo TCPE). 

 Particularidades do VO _2_ /METs: 

 – Considerava-se um marcador de alto risco (mau prognóstico) em adultos atingir no TE <5 METs em mulheres e <7 METs em homens, mas atualmente é preciso avaliar cada indivíduo em relação a sua idade, condicionamento físico e doenças associadas. ^735^ ^,^ ^738^

 – Vários estudos têm demonstrado que pacientes com ≥10 METs ao TE, em especial os idosos, independentemente de isquemia esforço-induzida, apresentaram baixas taxas de eventos cardíacos adversos e mortalidade. ^257^ ^,^ ^739^ ^-^ ^742^

 – A cada 1 MET de aumento na carga de trabalho, ocorreu uma redução de 18% de eventos cardíacos em homens ≥65 anos e de 14% nos <65 anos. ^742^

### 4.18.2. Déficit Funcional Aeróbico (FAI)

 O déficit funcional aeróbico (do inglês, *functional aerobic impairment* [FAI]) é a diferença percentual entre o VO _2_ max atingido (estimado ou medido) e o VO _2_ previsto (para idade, sexo e grau de atividade) por meio de equações de regressão. 

 O FAI é uma medida que expressa a porcentagem de déficit, ou seja, porcentagem de comprometimento da capacidade funcional aeróbica: ^743^

$FAI(\%)=\frac{{VO}_{2}\text{ máximo previsto }-{VO}_{2}\text{ máximo atingido }}{{VO}_{2}\text{ máximo previsto }}\times 100$

Legenda (idade em anos):

– VO^2^pico: estimado pelo TE ou medido no TCPE.

– Cálculo do VO^2^ máximo previsto (mL/kg/min):

1) Para sedentários

• Feminino: 42,3 – (0,356 × idade)

• Masculino: 57,8 – (0,445 × idade)

2) Para ativos

• Feminino: 42,9 – (0,312 × idade)

• Masculino: 69,7 – (0,612 × idade)

 Desse modo, os valores do FAI podem ser interpretados ( Tabela 34 ): 

**Tabela 34:** – Classificação da capacidade funcional baseado no FAI 744

Classificação	% do FAI
Superou o VO _2_ previsto (ideal)	Valor negativo*
Sem comprometimento significativo	0-26%
Comprometimento leve	27-40%
Comprometimento moderado	41-54%
Comprometimento acentuado	55-68%
Comprometimento extremo	>68%
*Quanto maior o valor negativo, melhor a aptidão cardiorrespiratória.	

 – Porcentagem positiva >26%: quanto maior o valor do FAI, maior será o comprometimento da capacidade funcional do paciente. 

 – De 0% a 26%: não há comprometimento significativo, tendo o paciente atingido o previsto para idade e sexo. 

 – Porcentagem negativa: o paciente superou o VO _2_ previsto. Essa situação é comumente observada em pessoas aparentemente saudáveis e ativas, e em especial em atletas. 

Suas principais aplicabilidades são:

 – Em TEs seriados, quantifica a evolução da capacidade aeróbica (piora ou melhora). 

 – Em atletas, para quantificar a progressão de melhoria da capacidade funcional. 

 – Estimar a aptidão cardiorrespiratória em relação à idade. O FAI com valor positivo representa que o paciente tem uma capacidade aquém da esperada para a sua idade, e vice-versa. ^745^

 – Parâmetro objetivo da evolução da aptidão cardiorrespiratória de pacientes em programas de treinamento físico e reabilitação. ^29^ ^,^ ^746^ ^,^ ^747^

### 4.18.3. Déficit Aeróbio Miocárdico (MAI)

 O déficit aeróbio miocárdico (do inglês, *myocardial aerobic impairment* [MAI]) também chamado de déficit ventricular esquerdo (do inglês, *left ventricular impairment* [LVI]) é uma medida que expressa a porcentagem de comprometimento da capacidade miocárdica, do ventrículo esquerdo, em responder às demandas de uma atividade física baseando-se nas respostas da FC e da PAS. A fórmula de cálculo do MAI é: ^748^


$$MAI(\%)=\frac{\text{ DP máximo previsto }-\text{ DP máximo atingido }}{\text{ DP máximo previsto }}\times 100$$

Legenda:

– DP (duplo-produto) máximo atingido = FCpico × PAS máxima.

– DP máximo previsto em sedentários:

• Feminino: [354 – (0,48 × idade)] × 100

• Masculino: [438 – (1,59 × idade)] × 100

– DP previsto para homens ativos:

DP máximo previsto = [364 – (0,58 × idade)] × 100

Observação: idade em anos. A interpretação do MAI é prejudicada quando ocorrer queda da FC intraesforço e/ou hipotensão/queda da PAS intraesforço (vide Tabelas 31 e 32). 

Particularidades do MAI:

 – Utilizado em TE seriados para acompanhamento evolutivo após intervenções em pacientes com DAC e valvopatia. ^749^ ^,^ ^750^

 – Coorte de 104 pacientes submetidos a ATCP e 38 com CRVM bem-sucedidas, submetidos ao TE (pré e pós-intervenção; acompanhamento de 2 anos), demonstrou que o MAI melhorou significativamente em ambos os grupos (ATCP de 20,2±17,8% para 9,9±15,8%; CRVM de 31,9±21,7% para 9,9±19,3%). ^751^

 4.19. Escores de Risco Pós-teste e Variáveis Prognósticas do TE 

 A aplicação de escores pós-teste reduz vícios de interpretação, melhora a acurácia diagnóstica, permite a estimativa prognóstica, fornece estratégias custo-efetivas de manejo de DCV e auxilia médicos não especialistas na interpretação dos resultados. ^752^ ^-^ ^755^

 Escores são desenvolvidos, validados e aplicados em populações específicas. A escolha, cuja justificativa sugere-se constar no relatório do exame, deve ser feita de acordo com as características de cada paciente. 

### 4.19.1. Escore de Duke

 O escore de Duke (ED) é um dos escores mais utilizados para avaliação diagnóstica de DAC grave e probabilidade de morbimortalidade. ^756^ ^,^ ^757^ Está indicado em pacientes sintomáticos, de ambos os sexos, com suspeita de DAC e idade entre 45 e 75 anos. ^758^ ^,^ ^759^ A aplicação do ED apresenta limitações significativas em assintomáticos, pacientes de baixo risco pré-teste, pós CRVM e após IAM recente. ^760^ ^,^ ^761^

 O ED incorpora apenas 3 variáveis do TE para o seu cálculo: a magnitude do desnível do segmento ST, o tempo de tolerância e a ocorrência de angina ao esforço. A equação de cálculo é: ^762^


ED= tempo de esforço −(5× desnível ST )−(4× angina )

Legenda:

1) Tempo de esforço: minutos de esforço no protocolo de Bruce. Quando utilizado outro protocolo, realizar a conversão dos METs atingidos para o correspondente no protocolo de Bruce para determinar o tempo em minutos.

2) Desnível ST: considerar a depressão ou elevação do segmento ST, medida em milímetros.

3) Angina: valor igual a zero, se ausência de angina; 1 ponto, se ocorreu angina durante o esforço; 2 pontos, se a angina tiver sido limitante.

 A pontuação final do ED varia de ≥+15 pontos a ≤-25 pontos, permitindo classificar o risco: 

– Alto: escore ≤-11 pontos, indica sobrevida em 5 anos de ≈67% e mortalidade anual ≥5%. 

– Intermediário: varia de -10 a +4 pontos, indica uma sobrevida em 5 anos de ≈90%. 

– Baixo: ≥+5 pontos, indica uma sobrevida em 5 anos de ≈97% e mortalidade anual ≤1%. ^756^ ^,^ ^757^

Particularidades do ED:

 – No estudo original de elaboração do ED, realizado em homens, 74% classificados como de alto risco tinham DAC oclusiva triarterial ou lesão de tronco de coronária esquerda à cineangiocoronariografia. ^762^

 – Estudo em 976 mulheres e 2.249 homens submetidos ao TE e cineangiocoronariografia demonstrou que mulheres e homens diferiram quanto ao ED (1,6 *vs* . -0,3; p<0,0001), prevalência de DAC (32% *vs.* 72%; p<0,001) e mortalidade em 2 anos (1,9% *vs* . 4,9%; p<0,0001). O ED teve desempenho melhor para excluir DAC em mulheres, principalmente quando classificadas como de baixo risco. ^763^

 – Em estudo prospectivo com 603 pacientes (seguimento por 2 anos), os pacientes com CPM normal e ED de risco baixo a intermediário tiveram menos IAM não fatal, enquanto os de alto risco apresentaram alto risco de IAM e morte CV. ^764^

 – 6.251 pacientes do estudo GISSI-2, realizaram TE 1 mês após IAM e foram estratificados pelo ED. Em acompanhamento por 6 meses, as taxas de mortalidade nos grupos de risco do ED foram: baixo risco = 0,6%; risco intermediário = 1,8% (RR: 2,50; IC 95%:1,47-12,59; p=0,0001); alto risco = 3,4% (RR: 5,13; IC 95%: 3,61-15,55; p=0,0001). ^765^

### 4.19.2. Escore de Athenas/Escore QRS

 O Escore de Athenas (EAthenas) é um escore pós-teste para avaliação diagnóstica de DAC multiarterial e que pode ser utilizado em ambos os sexos. Não deve ser aplicado nos portadores de bloqueios de ramo (direito ou esquerdo), na sobrecarga ventricular esquerda, na pré-excitação ventricular e no bigeminismo ventricular. ^766^ ^-^ ^768^

 As alterações na amplitude das ondas Q, R e S induzidas pelo esforço são úteis para o diagnóstico de DAC. ^769^ Em 1990, essas alterações foram reunidas em um escore denominado “Athenas QRS score”, atualmente também denominado Escore QRS (EQRS). ^770^

 É calculado por meio da média da amplitude das ondas Q, R e S em três complexos QRS consecutivos, nas derivações aVF e V5 no repouso e imediatamente após o esforço. Os complexos QS devem ser tratados como onda Q ou onda S. O Escore QRS é calculado pela fórmula: ^767^ ^,^ ^769^ ^,^ ^770^


 Escore QRS (em milimetros) =(ΔR−ΔQ−ΔS)aVF+(ΔR−ΔQ−ΔS)V5

Escore QRS (em milímetros) = (ΔR – ΔQ – ΔS)aVF + (ΔR – ΔQ – ΔS)V5

ΔR = média de R em repouso – média de R no pico do esforço

ΔQ = média de Q em repouso – média de Q no pico do esforço

ΔS = média de S em repouso – média de S no pico do esforço

 O valor considerado normal do EQRS, em pacientes sem DAC, é >+5 mm. 

No TE, o EQRS é considerado anormal quando:

 – O valor do EQRS ≤+5 mm prevê a existência DAC obstrutiva, independentemente de alterações do segmento ST, com sensibilidade variando de 75% a 86% e especificidade de 73% a 79%. Os valores do EQRS estão relacionados com a gravidade da DAC; quanto menor for o escore, maior é a probabilidade de estenose coronariana multiarterial significativa. ^770^ ^,^ ^771^

 – Em mulheres EQRS <+5 mm associado a resposta eletrocardiográfica isquêmica aumentou a sensibilidade de 59% para 80%, a especificidade de 40% para 94%, a acurácia de 50% para 87% e reduziu os falsos-positivos de 60% para 6%. ^772^

 – EQRS <+5 mm tem maior capacidade diagnóstica de reestenose quando comparado ao ISTs em pacientes 6 meses após ATCP em vaso único (sensibilidade = 80%, especificidade = 89% e VPP = 77%) e de isquemia após 1 ano de CRVM (sensibilidade = 75%, especificidade = 86% e VPP = 62%). ^766^ ^,^ ^773^

 – EQRS ≤-3 mm prevê estenose coronariana multiarterial após 1 mês de IAM. ^774^

 – EQRS <-4 mm em pacientes com DAC foi preditor independente de mortalidade cardíaca (RR: 11,7; IC 95%: 2,5-55,4; p=0,002). ^768^

### 4.19.3. Escore de Raxwal e Morise

 O escore de Raxwal e Morise é um escore pós-teste para avaliar a probabilidade de DAC em ambos os sexos, sintomáticos ou assintomáticos. Seu cálculo envolve uma somatória de pontos, conforme mostra a Tabela 35 . ^775^ ^,^ ^776^

**Tabela 35:** – Escore de Raxwal e Morise

Variáveis	Homens		Mulheres	
	Dados	Pontuação	Dados	Pontuação
**FC máxima**	<100 bpm	30	<100 bpm	20
	100 a 129 bpm	24	100 a 129 bpm	16
	130 a 159 bpm	18	130 a 159 bpm	12
	160 a 189 bpm	12	160 a 189 bpm	08
	190 a 220 bpm	06	190 a 220 bpm	04
**Infradesnível ST**	1-2 mm	15	1-2 mm	06
	>2 mm	25	>2 mm	10
**Angina intraesforço**	Presente	03	Presente	09
	Limitante	05	Limitante	15
**Idade**	>55 anos	20	>65 anos	25
	40-55 anos	12	50-65 anos	15
**Relato de angina pré-TE**	Definida/típica	05	Definida/típica	10
	Provável/atípica	03	Provável/atípica	06
	Não cardíaca	01	Não cardíaca	02
**Diabetes**	Presente	05	Presente	10
**Dislipidemia**	Presente	05	Não avaliar	--
**Tabagismo**	Não avaliar	--	Presente	10
**Estado estrogênico**	Não se aplica	--	Positivo	- 05
			Negativo	+ 05
* Estados estrogênico: negativo = mulheres pós-menopausadas, ooforectomizadas ou sem terapia de reposição hormonal; positivo = mulheres pré-menopausa ou em terapia de reposição hormonal. *				

 Classificação de probabilidade de DAC pelo escore de Raxwal e Morise: 

– Baixa probabilidade: de 0 a 39 pontos.

 – Probabilidade intermediária: entre 40 e 60 pontos. 

– Alta probabilidade: >60 pontos.

 Estudo com 4.640 pacientes (idade média: 50 anos, 53% homens), sem DAC conhecida e submetidos ao TE para avaliar dor torácica, demonstrou que o escore de Raxwal e Morise e o escore de Duke estratificaram adequadamente os pacientes quanto ao risco (baixo, intermediário e alto; p<0,00001). Nesse estudo, o escore de Raxwal e Morise apresentou melhor valor prognóstico para mortalidade por todas as causas. ^776^

## 5. Critérios de Interrupção do Esforço

 Em termos gerais, a interrupção do esforço é baseada na sintomatologia, nos dados do exame físico, variáveis cardiovasculares, respiratórias e eletrocardiográficas, eventuais falhas na monitorização eletrocardiográfica e outras condições consideradas de risco para intercorrências graves ( Tabela 36 ). 

**Tabela 36:** – Critérios de interrupção do esforço 6,209,210,762,777

Parâmetro	Critérios
**Sintomatologia**	– Exaustão física (Escala de Borg ≥18) – Dor e fadiga da musculatura de membros inferiores – Claudicação de membros inferiores (limitante), ataxia – Vertigem persistente e limitante, náusea, pré-síncope, síncope – Desconforto ou dor torácica crescente com incremento das cargas do esforço (limitante), angina típica (moderada a forte intensidade) – Dispneia precoce e desproporcional à intensidade do esforço
** Exame físico/variáveis cardiovasculares e respiratórias **	– Palidez cutânea e de mucosas, sudorese profusa e desproporcional, má perfusão periférica – Taquipneia desproporcional ao esforço, broncoespasmo, estertores crepitantes em bases pulmonares; aumento de estertores crepitantes em idosos* – Surgimento de sopro cardíaco e/ou de B3 ou B4 – PAS com aumento inicial no esforço seguido por uma queda da PAS ≥20 mmHg. Caso assintomática, confirmar a queda em pelo menos mais uma mensuração – Elevação acentuada da PAS >250 mmHg – Elevação da PAD ≥120 mmHg nos normotensos; elevação da PAD ≥140 mmHg nos hipertensos – Oximetria digital normal no basal seguida de dessaturação (SpO _2_ ≤92%)
**Eletrocardiográficas**	– Modificações do segmento ST: infradesnivelamento ≥0,3 mV (3,0 mm) adicionais aos valores de repouso; supradesnivelamento ≥0,2 mV (2,0 mm) em derivação sem evidência de infarto prévio – Taquiarritmia supraventricular não sustentada sintomática ou com repercussão hemodinâmica – Taquiarritmia supraventricular não sustentada, assintomática ou sem repercussão hemodinâmica: individualizar o número de batimentos e repetições, considerando a indicação do TE e doenças de base – Taquiarritmia supraventricular sustentada (≥30 segundos) – Fibrilação atrial ou flutter atrial esforço-induzidos – Taquicardia ventricular não sustentada (≥3 batimentos/<30 segundos)** ou um episódio de TVNS polimórfica – Taquicardia ventricular sustentada (≥30 segundos) – Fibrilação ventricular – BAV de 2 **º** e 3 **º** graus esforço-induzidos – Bloqueio de ramo esquerdo esforço-induzido (sintomático ou com repercussão hemodinâmica)*** – Desenvolvimento de bloqueio de ramo quando não puder distinguir de taquicardia ventricular – Portadores de CDI: interromper 10 batimentos abaixo da FC de acionamento do desfibrilador – Queda persistente da FC cardíaca com o incremento de carga
**Outras**	– A pedido do paciente, independentemente da ocorrência de anormalidades – Falência dos sistemas de monitorização e/ou registro eletrocardiográfico – Inadaptação e/ou falta de coordenação ao ergômetro
PAS: pressão arterial sistólica; PAD: pressão arterial diastólica; BAV: bloqueio atrioventricular; CDI: cardiodesfibrilador implantável; FC: frequência cardíaca; TVNS: taquicardia ventricular não sustentada. *Em idosos assintomáticos, é comum a presença de crepitações pulmonares no exame basal, sendo a idade o único preditor independente. **Em caso de episódio único de TVNS, individualizar o número de batimentos para a interrupção, levando em consideração a indicação do TE, patologia de base, achados clínicos durante o TE e local de realização (hospitalar ou não). ***Nos casos assintomáticos ou sem repercussão, individualizar a critério do médico executante.	

## 6. Elaboração do Laudo do TE

 Imediatamente após o término da recuperação, o médico executante do TE deve analisar e interpretar todos os dados pré-teste, sintomatologia, achados e alterações do exame físico, variáveis eletrocardiográficas, medições e registros realizados, intercorrências, escores e informações prognósticas pertinentes. A partir dessas informações, deve-se redigir o laudo do exame seguindo os requisitos mínimos apresentados nesta seção e composto por: 

1) Descrição dos dados gerais do exame.

2) Dados observados, mensurados e registrados.

3) Relatório descritivo do TE.

4) Conclusões.

5) Registros eletrocardiográficos.

### 6.1. Dados Gerais

 O laudo do TE deverá apresentar, inicialmente, a descrição das informações gerais inerentes ao paciente (dados gerais), sobre sua condição de saúde, indicações do exame e ergômetro/protocolo: 

 1) Identificação do paciente: nome, sexo, peso, altura, IMC, registro do paciente no sistema de ergometria (número ou código atribuído ao exame). 

 2) Condições de saúde do paciente: medicamentos em uso corrente (relatar se houve cumprimento de solicitação de suspensão de medicação feita pelo médico solicitante); fatores de risco para DCV; escore de risco pré-teste. 

3) Indicação do exame e/ou CID.

 4) Ergômetro, protocolo de esforço e sistema de registro do ECG (12 ou 13 derivações e posição dos eletrodos). Nos protocolos escalonados, sugere-se detalhar todas as variáveis adotadas: duração (mínima, média e máxima); carga (inicial e final) e/ou a velocidade (inicial e final) e inclinação (inicial e final). 

 5) Observações adicionais facultativas consideradas relevantes, tais como resultados de exames prévios, modelo de marca-passo/CDI etc. 

### 6.2. Dados Observados, Mensurados e Registrados

 Após a seção de dados gerais, o laudo deve apresentar os dados de todas as fases do TE (repouso, esforço e recuperação): 

 1) Tabelas contendo as informações de medições da PAS e PAD (com respectivas FC e sintomas associados) e registros eletrocardiográficos (com FC, METs, PA e DP do momento do registro). 

 2) Sugere-se utilizar gráficos para avaliação do comportamento das medições da PA (sistólica e diastólica), da FC e do segmento ST. 

 3) Tabela contendo informações sobre sintomas apresentados e os momentos de início e de melhora (se possível, com a respectivas PA, FC e DP). 

 4) Dados obtidos sobre: FC de repouso; FC máxima prevista; FC máxima atingida ou pico; FAI; duplo-produto; valor (numérico ou interpretativo) da escala de percepção de esforço (Borg ou Borg Modificada); VO _2_ /METs (previsto, atingindo e porcentagem); classificação funcional. 

 5) Sugere-se a utilização de escore de risco pós-teste com respectiva legenda de interpretação do resultado obtido. 

 6) Apresentar outras medições, dados, tabelas e gráficos julgados pertinentes: comportamento de variáveis eletrocardiográficas, tais como QTi, QTc, escore de QRS etc.; escala utilizada para a quantificação de angina, dispneia e claudicação intermitente; comportamento de marca-passo e/ou CDI. 

 7) Exames adicionais ao TE passíveis de agregar valor diagnóstico e prognóstico: oximetria; índice tornozelo-braquial; medições de dosagens sanguíneas. 

### 6.3. Relatório Descritivo

 Deve ser elaborado relatório descritivo, ordenado e interpretativo, apresentando resumidamente o comportamento dos dados e variáveis obtidos no TE em todas as suas fases. As informações necessárias para a adequada descrição e interpretação do TE constam nesta diretriz. 

O relatório descritivo deve conter:

 1) Dados relevantes da avaliação pré-teste do paciente referentes à anamnese dirigida e exame físico (sumário e específico). 

 2) Comentário breve sobre a adaptação do paciente ao ergômetro e protocolo. 

3) Momento e motivo(s) da interrupção do esforço.

 4) Descrição sumária das respostas clínicas (sintomas, sinais clínicos, exame físico, tolerância ao esforço) e suas possíveis interpretações. Caso ocorra evento adverso, realizar relato detalhado. 

 5) Descrição das respostas hemodinâmicas (comportamento da FC, PA, DP e outros parâmetros julgados pertinentes) e interpretação dos resultados. 

 6) Descrição das respostas eletrocardiográficas. Quando anormais, incluir as respectivas interpretações: 

 – Ondas e intervalos: onda P, PRi, onda Q, onda R, onda S, complexo QRS, onda T, onda U, QTi, QTc. 

 – Segmento ST: infradesnivelamento, supradesnivelamento, pseudonormalização ou ausência de mudanças. Descrever quando iniciou, maior magnitude atingida, se há sintomas associados, momento de normalização e se preencheu critérios de isquemia. 

 – Condução atrioventricular normal ou distúrbio da condução AV (preexistente ou esforço-induzido). 

 – Condução intraventricular ou distúrbios da condução IV (preexistentes ou esforço-induzidos). 

 – Arritmias: momento de ocorrência, sintomas associados, repercussão hemodinâmica, comportamento (mudanças na densidade e complexidade em todas as etapas) e eventual momento de desparecimento. 

 7) Descrição interpretativa da avaliação metabólica indireta: VO _2_ /METs, FAI, classificação funcional etc. 

 8) Comentários pertinentes sobre dados dos escores de risco pré-teste. Caso utilizado o escore de risco pós-teste (facultativo) e variáveis prognósticas do TE (caso aplicável), também apresentar os comentários pertinentes. 

 9) Particularidades dos resultados obtidos quanto às seguintes condições: 

 – Clínica específica do paciente (doenças preexistentes, limitações físicas e psicológicas). 

– Efeito e interferência de medicações em uso.

 – Limitações/interferências na interpretação das variáveis do TE. 

 No relatório do TE, não é recomendada a liberação de prescrição de exercícios, baseada no desempenho físico atingido. A prescrição de exercícios é de responsabilidade do médico assistente. 

Sugere-se:

 – Inclusão de um glossário de termos e abreviaturas encontrados no laudo do TE, de modo a permitir a correta compreensão. 

 – Quando pertinente, interpretação e comparação com TE realizados anteriormente. 

 – Utilização de sistema de ergometria que facilite a elaboração do relatório descritivo: apresentar ordenadamente todas as possíveis informações a serem relatadas; utilizar frases pré-programadas visando à padronização dos laudos; permitir edição e descrições individualizadas. 

 – Incorporar relatório complementar de exames adicionais realizados no momento do TE (p. ex., medidas bioquímicas laboratoriais). 

### 6.4. Conclusão

 A conclusão deve apresentar, de maneira concisa e clara, informações relacionadas à indicação do exame e eventuais parâmetros anormais relevantes ao diagnóstico e prognóstico do paciente: 

1) Comportamento clínico.

 2) Resposta pressórica, baseada nos conceitos da Tabela 32 . 

 3) Comportamento cronotrópico, baseado nos conceitos da Tabela 31 . 

4) Arritmias.

 5) Resposta eletrocardiográfica incluindo a repolarização ventricular. 

6) Classificação funcional.

 7) Referir eventuais limitações/interferências na interpretação das variáveis do TE. 

 Não são recomendadas a utilização das expressões: “teste positivo”, “teste negativo” ou “teste inconclusivo”. Essas expressões são vagas, geralmente restritas a uma única variável (segmento ST), desprezando a interpretação dos múltiplos parâmetros/variáveis necessários para adequados diagnóstico e prognóstico proporcionados pelo TE. 

### 6.5. Registros Eletrocardiográficos

 O laudo do TE necessita conter registros que demonstrem a evolução eletrocardiográfica de todas as fases (repouso, esforço e recuperação) e representem as informações pertinentes do relatório descritivo. Sugere-se que os registros sejam realizados em 12 ou 13 derivações e que se faça registro de ritmo contínuo (geralmente DII) para documentação de arritmias. Cada registro de ECG deverá conter a etapa do TE, a velocidade e a amplitude do traçado, a FC e, caso disponível, a PA medida. 

 É facultativo o registro de complexos médios calculados automaticamente. Evitar quando em presença de artefatos, grande oscilação da linha de base e de arritmias ventriculares por causarem interferência no cálculo. 

 Sugere-se incorporar tabela com os valores automáticos das medições das ondas, intervalos e segmentos do ECG, desde que relevantes e coerentes com o diagnóstico do TE. 

##  7. Exames Realizados Simultaneamente e Adicionalmente ao TE 

### 7.1. Índice Tornozelo-braquial

 O índice tornozelo-braquial (ITB) é um exame não invasivo, de diagnóstico e acompanhamento da doença arterial periférica (DAP) dos membros inferiores. É um forte marcador de aterosclerose, comprometimento funcional, risco cardiovascular e de mortalidade. ^778^ ^,^ ^779^ Pode identificar pacientes sob risco de complicações nas extremidades dos membros inferiores, passíveis de abordagem preventiva otimizada. ^780^

O ITB pode ser realizado:

 – Em repouso, durante avaliação clínica especializada. É recomendado em pacientes com suspeita clínica ou exame físico sugestivo de DAP (Recomendação I; Nível de evidência B): dor em membros inferiores aos esforços; claudicação intermitente; abolição do pulso e/ou sopro arterial nos membros inferiores; feridas nos membros inferiores que não cicatrizam. É recomendado em pacientes em risco de DAP: DAC ou obstrução arterial aterosclerótica em outras partes do corpo (p. ex., carótidas; subclávia); doença renal crônica; IC; homens e mulheres assintomáticos >65 anos ou <65 anos com alto risco para DCV e também se >50 anos com história familiar de DAP. ^780^ ^-^ ^785^

 – Pós-esforço, associado adicionalmente ao TE em esteira ergométrica (ITB pós-esforço), é útil para estabelecer o diagnóstico de DAP nos pacientes sintomáticos quando o ITB em repouso é normal ou limítrofe. Permite quantificar objetivamente as limitações funcionais atribuíveis aos sintomas, bem como realizar estratificação aprimorada de risco. As indicações encontram-se na Tabela 37 . ^780^ ^,^ ^781^ ^,^ ^786^ ^-^ ^788^

**Tabela 37:** – Indicações de realização de ITB pós-esforço associado ao TE em esteira ergométrica

Indicação	GR	NE
Pacientes com sintomas esforço-induzidos em membros inferiores* e ITB em repouso normal ou limítrofe (>0,90 e ≤1,40) para diagnóstico e estratificação de risco ^781,788-791^	I	B
Pacientes com DAP e ITB em repouso anormal (≤0,90) para avaliação do estado funcional e estratificação prognóstica ^781,787,792,793^	IIa	B
Pacientes diabéticos assintomáticos com ITB em repouso anormal (≤0,90) para avaliação do estado funcional e estratificação prognóstica ^785^	IIb	B
Em associação com a medida da pressão transcutânea de oxigênio no esforço para investigação de estenoses arteriais de membros inferiores ^794,795^	IIb	B
GR: grau de recomendação; NE: nível de evidência. *Sintomas não relacionados às articulações.		

#### 7.1.1. Realização do Exame ITB

 O exame ITB associado ao TE compreende duas fases: em repouso e pós-esforço em esteira ergométrica. 

**7.1.1.1. ITB de Repouso**

 O ITB de repouso é medido com o paciente em decúbito dorsal com a cabeça e calcanhares apoiados, em ambiente com temperatura confortável (19-22°C) após um descanso de 5 a 10 minutos. São realizadas medições da pressão arterial sistólica nos quatro membros utilizando ultrassom Doppler de onda contínua (5-10 MHz): ^780^ ^,^ ^781^ ^,^ ^791^

 – Utilizar manguito adequado para a circunferência e largura dos membros (deve contornar pelo menos 40% da circunferência). 

 – O manguito deve ser colocado logo acima do tornozelo (evitando áreas com feridas), com sua borda inferior situada 2 cm acima do ponto superior do maléolo medial. 

 – Gel ecocardiográfico deve ser aplicado sobre o transdutor do Doppler, colocando-o na área do pulso, em ângulo de 45° a 60°. Mover o transdutor até localizar o som mais claro do fluxo arterial. O manguito deve ser inflado progressivamente até 20 mmHg acima do nível de desaparecimento do som. Desinflar lentamente para detectação do reaparecimento do som com registro da PAS correspondente. 

 – Medir e registrar a PAS nas artérias braquiais de ambos os braços e nas artérias tibial posterior e anterior de ambos os tornozelos (ou pediosa dorsal do mesmo membro que o pulso estiver ausente em uma das tibiais). ^796^

 – Para o cálculo do ITB, utilizar a pressão mais alta encontrada nas medições das artérias de cada perna. 

 O ITB de cada perna é calculado dividindo a PAS mais alta do tornozelo pela PAS do braço do mesmo lado. ^780^ Os valores do ITB devem ser registrados com 2 casas decimais. ^781^


$$\text{ ITB de repouso }=\frac{\text{ Maior PAS medida em uma das artérias do tornozelo }}{\text{ Maior PAS braquial medida nos membros superiores }}$$

ITB: índice tornozelo-braquial; PAS: pressão arterial sistólica medida com ultrassom Doppler. Numerador: maior pressão arterial sistólica medida em uma das artérias (tibial posterior e anterior; ou pediosa dorsal do mesmo membro que o pulso estiver ausente em uma das tibiais). Denominador: maior pressão braquial medida nos dois membros superiores.

Observação: registrar qual artéria do tornozelo apresentou a maior PAS, pois a medição para cálculo do ITB pós-esforço deverá ser feita na mesma artéria.

 Os aparelhos automáticos de medição de pressão arterial não são, na maioria das vezes, válidos para medição da PAS do tornozelo e geralmente superestimam os valores da pressão quando baixa. 

 O resultado do ITB de repouso deverá ser interpretado de acordo com a finalidade do exame: 

 – Para diagnóstico de DAP, interprete cada perna separadamente (um ITB de repouso por perna). 

 – Na estratificação de risco CV, considerar o ITB mais baixo obtido nas duas pernas. 

 O ITB de repouso é considerado: ^780^ ^,^ ^781^

 – Anormal (baixo): ≤0,90. Em sintomáticos, é critério diagnóstico de DAP com estenose >50%, associada ao risco aumentado de eventos coronarianos, mortalidade CV e mortalidade por total em 10 anos. ^781^ ^,^ ^797^

 – Limítrofe baixo: 0,91-0,99. Não consegue descartar DAP. Se houver sintomas suspeitos de isquemia esforço-induzida de membros inferiores, recomenda-se a realização do ITB pós-esforço. ^791^

– Normal: 1,00-1,40.

 – Alto: >1,40. Representa enrijecimento arterial (calcificação média-intimal) impossibilitando a compressão das artérias. É mais prevalente em idosos (principalmente nos diabéticos ou com DRC), apresenta menor sensibilidade para o diagnóstico de DAP, mas está associado a maior risco de eventos CV e mortalidade. ^778^

 O ITB de repouso apresenta desempenho razoavelmente bom para diagnosticar DAP com sensibilidade de 61% a 73% e especificidade de 83% a 96%. ^790^ ^,^ ^798^ A sensibilidade é menor em pacientes com diabetes e/ou doença renal crônica (DRC) em estágio avançado devido à frequente ocorrência de calcificação arterial. ^785^ ^,^ ^791^

**7.1.1.2. ITB Pós-esforço**

 Em pacientes saudáveis, a realização de esforço em esteira ergométrica acarreta aumento progressivo da PAS na circulação central e membros superiores. Entretanto, devido à vasodilatação fisiológica na musculatura dos membros inferiores, ocorre diminuição da PAS ao nível do tornozelo e, consequentemente, leve diminuição do ITB pós-esforço, em média 5% do valor do ITB de repouso. Em pacientes com DAP, ocorre diminuição >20%. ^791^ ^,^ ^799^

 Em 1 a 2 minutos de recuperação, a PAS dos membros inferiores aumenta rapidamente, retornando aos valores pré-esforço. Retorno a pelo menos 90% do valor do ITB de repouso nos primeiros 3 minutos da recuperação descarta DAP (especificidade de 94%). ^791^ ^,^ ^799^

 Na DAP oclusiva (tipicamente dos vasos proximais), a pressão do tornozelo diminui mais acentuadamente com o esforço, e o tempo para retorno ao valor de repouso é prolongado, sendo proporcional à gravidade da DAP. ^791^

#### 7.1.2. Preparação do Paciente e Técnica de Exame

 Adicionalmente às orientações feitas ao paciente para o TE, recomenda-se que: 

 1) Use roupas que permitam a fácil exposição dos braços e pernas, aplicação dos manguitos necessários e medição da PA. 

 2) Não utilizar calção/bermuda com pernas apertadas e de tecidos com compressão (bermuda com elastano; bermuda *fitness* ; bermuda *training* ; short de corrida *run dry* etc.). 

 É feita uma medição basal da PAS nos braços e tornozelos em repouso. 

 Adequação do TE: ^797^ ^,^ ^800^

 1) O exame deverá ser feito em esteira ergométrica. 

 2) Protocolo de esforço sem incremento (carga fixa), mantendo caminhada em velocidade de 3,2 km/h (2 mph), com inclinação de 10% a 12,5%, com duração máxima de 5 minutos (para não prejudicar a avaliação do tempo de recuperação do ITB). ^801^ Para paciente com limitação importante, ajustar a velocidade e/ou inclinação (valores menores). 

 3) O esforço pode ser interrompido antes caso haja dor em membro inferior e/ou claudicação absoluta (impeditiva). 

4) Não realizar recuperação ativa.

 5) Todos os parâmetros clínicos, hemodinâmicos, eletrocardiográficos, aptidão cardiorrespiratória e escores deverão ser avaliados conforme preconizado nesta diretriz. 

 6) Registrar todos os sintomas ocorridos. Sugere-se utilizar escala de percepção subjetiva de esforço (Borg ou Borg modificada) e escala de claudicação intermitente. 

 7) Após a interrupção do esforço, realizar recuperação passiva com o paciente em decúbito dorsal. A PAS deve ser imediatamente medida nos 2 braços e 2 tornozelos (nas artérias que apresentavam a maior PAS medida para o cálculo do ITB em repouso). Repetir as medições da PAS e cálculo do ITB bilateralmente (por pelo menos 3 vezes) e/ou até o retorno da PAS dos membros inferiores ao valor basal. Registrar todos os valores de PAS obtidos com respectivos momentos de medição e sintomas associados. O cálculo do ITB pós-esforço é feito conforme a equação: 


$$\text{ ITB pós-esforço }=\frac{\text{ PAS medida na mesma artéria do ITB em repouso }}{\text{ Maior PAS braquial medida nos membros superiores }}$$

ITB: índice tornozelo-braquial; PAS: pressão arterial sistólica medida com ultrassom Doppler.

Observação: artéria do tornozelo com maior PAS utilizada para cálculo do ITB de repouso. Medir nos 2 tornozelos e calcular o ITB pós-esforço para os 2 membros. Denominador: maior pressão braquial medida nos dois membros superiores.

 Recomenda-se o cálculo da variação percentual do ITB em cada membro (individualmente) através da equação: 


$$\text{ Variação percentual do ITB }(\%)=\frac{\text{ ITB pós-esforço }-\text{ ITB de repouso }}{\text{ ITB de repouso }}\times 100$$

Os critérios para diagnóstico de DAP ainda não foram totalmente padronizados. Recomenda-se considerar como exame alterado, compatível com diagnóstico de DAP: ^780^ ^,^ ^781^ ^,^ ^791^ ^,^ ^799^

 – ITB pós-esforço <0,90 (sensibilidade 70% a 88% e acurácia de 72% para DAP) ^788^ ^,^ ^791^ ^,^ ^795^ ^,^ ^802^ ou 

 – Queda >20% nos valores de ITB pós-esforço em relação ao repouso em um mesmo membro (sensibilidade de 67% e estratificação de risco de mortalidade e eventos CV) ^800^ ^,^ ^802^ ^,^ ^803^ ou 

 – Queda de PAS no tornozelo >30 mmHg em relação ao padrão da PAS basal. 

 Uma limitação do ITB pós-esforço é a presença de ITB de repouso anormalmente alto devido à artéria do tornozelo incompressível (com PAS >250 mmHg) impossibilitando a adequada detecção da doença pelo ITB pós-esforço. 

Particularidades do ITB pós-esforço:

 – Estudo demonstrou que, em indivíduos com ITB de repouso normal, a adição do ITB pós-esforço identificou 25% a mais de lesões com estenoses significativas (>75%). ^802^

 – Estudo em 619 pacientes (idade média 64,2 anos; 64% homens) com suspeita de DAP e ITB de repouso normal e pós-esforço <0,90 apresentou sensibilidade de 81,7%, especificidade de 94,7%, VPP de 84,8% e VPN de 94,4%. A variação percentual do ITB >20% mostrou 70,4% sensibilidade, 83,4% especificidade, 47,2% VPP e 93% de VPN. ^787^

## 7.2. Oximetria Não Invasiva

 A oximetria não invasiva (oximetria de pulso) é um exame que visa monitorar continuamente a oxigenação tecidual. Contribui na detecção de hipóxia e de instabilidade hemodinâmica, tanto no repouso quanto no esforço, as quais poderiam passar despercebidas por avaliação clínica isolada. A oximetria não invasiva é um procedimento médico previsto na Classificação Brasileira Hierarquizada de Procedimentos Médicos (Código: 4.14.01.51-4). ^804^

 O oxímetro de pulso convencional monitora de forma contínua, indireta e transcutânea a saturação de oxigênio da hemoglobina no sangue arterial (SpO _2_ ) baseando-se na metodologia espectrofotométrica. A determinação da SpO _2_ tem como base a média de uma série de medições comparadas a uma curva de calibração interna do equipamento (medições SpO _2_ <70% não são confiáveis). ^805^ ^,^ ^806^

 O oxímetro de pulso em vários comprimentos de onda permite medir o monóxido de carbono (CO) ligado à hemoglobina (CO-oximetria), determinar a carboxi-hemoglobina (CO _2_ Hb) e a meta-hemoglobina (MetHb), definindo melhor a eventual causa de hipóxia/hipoxemia. ^807^

**Figura 16:**
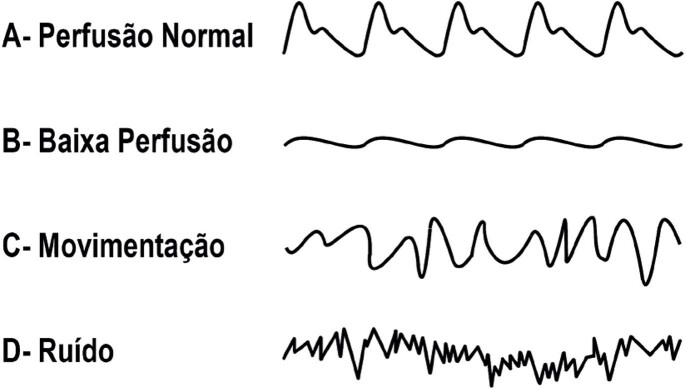
–Comportamento da onda pletismográfica normal (A), na baixa perfusão (B) e na presença de artefatos (movimentação e ruído [C e D]).

 O oxímetro também registra a forma de onda associada (onda pletismográfica), correspondendo aos sinais pulsáteis associados aos batimentos cardíacos. A onda pletismográfica é importante na avaliação da qualidade do sinal e interpretação dos dados de saturação ( Figura 16 ): ^808^

 – Quando normal, a onda apresenta entalhe dicrótico evidente, associado ao batimento cardíaco. 

 – Durante baixa perfusão, a onda apresenta-se na forma senoidal típica com baixa amplitude e sem entalhe (mas mantendo associação aos batimentos). 

 – A presença de arritmias pode tornar a ocorrência das ondas irregulares e, no caso de EVs, a onda pode apresentar-se com amplitude diminuída. 

 – A presença de artefatos (de ruído ou movimentação) é percebida como uma onda de forma irregular, com múltiplos entalhes e sem uma relação com os batimentos. 

 A onda pletismográfica tem semelhança com o traçado da pressão arterial invasiva, mas não é um análogo direto da pressão arterial ou do débito cardíaco. 

 A oximetria de pulso associado ao TE (OxPTE) deve ser realizada durante todas as fases do TE (repouso, esforço e recuperação), assim como já é feito no TCPE. Pode ser realizada tanto na população pediátrica quanto em adultos. 

 A OxPTE é um exame complementar que aumenta a segurança do TE e permite avaliação mais precisa de sintomas de dispneia e fadiga em populações específicas. A indicação de realização da OxPTE associada ao TE não modifica as suas indicações e graus de recomendação. ^809^

 Como indicações gerais da OxPTE, temos: pacientes com queixa de dispneia aos esforços; doenças pulmonares; cardiomiopatias; CC; IC; valvopatias; pós-COVID. ^228^ ^,^ ^810^ As principais indicações específicas encontram-se na Tabela 38 . 

**Tabela 38:** – Indicações de realização de oximetria de pulso associada ao TE 219,811

Indicação	GR	NE
Crianças e adolescentes com cardiopatia congênita (corrigida e não corrigida), cardiomiopatias, insuficiência cardíaca, valvopatias ^812,813^	I	B
Pacientes pediátricos e adultos em investigação de queixa ou diagnóstico diferencial de dispneia*	IIa	B
Pacientes adultos com valvopatia ou cardiomiopatia assintomática	IIb	B
Pacientes pediátricos e adultos após infecção respiratória com potencial capacidade de comprometimento da função pulmonar (p. ex., pós-COVID) ^228^	IIb	B
Pacientes pediátricos e adultos após cirurgia cardíaca (p. ex., troca valvar, revascularização miocárdica)	IIb	B
Pacientes adultos com insuficiência cardíaca ou cardiopatia congênita ^814^	IIb	B
GR: grau de recomendação; NE: nível de evidência.*Nos quais o TCPE não tenha indicação formal de realização ou quando o TCPE não estiver disponível.		

### 7.2.1. Equipamentos

 Recomendamos a utilização de oxímetro de pulso com medida de absorção de luz em vários comprimentos de onda, determinação da saturação de O _2_ e CO-oximetria, registro da FR e de ondas pletismográficas. Preferir equipamento que tenha calibração/validação para medições durante esforço físico. O oxímetro poderá ser de mesa ou integrado ao computador/sistema do TE. Deverá, preferencialmente, utilizar *software* de comunicação com o computador, permitindo exportar os dados necessários à elaboração de relatório. ^804^

 Os sensores do oxímetro devem ser de tamanho adequado ao paciente (modelo pediátrico e adulto), com diferentes formas de fixação (p. ex., de dedo; lóbulo da orelha). O cabo do sensor deve ser flexível, de tamanho adequado para movimentação do paciente e com capacidade para suportar tensões. ^814^ Recomenda-se acompanhar durante o TE (pelo computador ou visor do oxímetro) o comportamento da SpO _2_ , FR e ondas plestimográficas. Não se recomenda a utilização de oxímetro de dedo portátil (utilizado em consultas clínicas). 

###  7.2.2. Procedimentos da Oximetria Não Invasiva 804 

 – Explicar e orientar o paciente sobre todo o procedimento necessário à realização da oximetria. 

 – Certificar-se de que a área onde será feita a aferição esteja limpa. 

 – Quando for utilizado sensor de lóbulo da orelha, é necessário remover todas as joias da orelha que possam interferir no encaixe adequado do sensor. 

 – No caso de sensor de dedo (preferencialmente no dedo indicador), é necessário remover o esmalte das unhas e joias da mão. Não colocar o sensor no membro em que for monitorizar a PA. 

 – Certificar-se de que o sensor está adequadamente adaptado (sem estar muito solto ou muito apertado). 

 – Ligar o oxímetro e aguardar o tempo de autocalibração para aferição. 

 – Aguardar que o oxímetro detecte o pulso, a FR e calcule a saturação de oxigênio. Caso ocorram artefatos e inconstância no sinal, reposicionar o sensor e/ou trocar para outro local do corpo (p. ex., do dedo para o lóbulo da orelha). 

 Durante a realização do esforço do TE: ^815^

 – Quando for utilizado o sensor de dedo, desencorajar o paciente de segurar firmemente o guidão/barra do ergômetro para evitar ruídos. 

 – Caso ocorra registro de artefatos frequentes (ruído e/ou movimento), ajustar o sensor e, se necessário, reposicioná-lo. 

 – Em valores muito alterados em medições sequenciais (sem artefatos como causa), avaliar a presença de sintomas, FR e achados de ausculta pulmonar, de modo a confirmar os achados. ^816^

### 7.2.3. Interpretação dos Dados

 A FR deverá ser interpretada de acordo com a faixa etária do paciente e carga de esforço desenvolvida. As ondas pletismográficas deverão ser interpretadas durante a realização da monitorização, conforme descrito anteriormente nesta seção. 

 Quanto à avaliação da SpO _2_ basal (em repouso), em ar ambiente, considerar: ≥97% – função pulmonar normal; 96-91% – função pulmonar anormal (leve a moderada); ≤90% – hipoxemia/hipóxia. 

 Valores de SpO _2_ basal <94% são geralmente encontrados em pacientes tabagistas, com doenças pulmonares crônicas, com CC e IC. No caso de hipóxia, verificar a pertinência da suspensão do TE, de acordo com sintomatologia do paciente e quadro clínico. ^219^

 Em adultos, assintomáticos, aparentemente saudáveis, espera-se uma oscilação entre 1% e 4% na saturação de oxigênio durante o esforço. Na recuperação, espera-se desaparecimento dessa oscilação nos primeiros minutos. 

 Queda de ≥5% de SpO _2_ foi associada às variáveis de repouso (homens, idosos, tabagistas), além de menor FC máxima e VO _2_ . ^816^

 Tanto nos pacientes pediátricos quanto adultos, hipotensão grave, baixo débito cardíaco, vasoconstrição e hipotermia reduzem o volume pulsátil de sangue nas extremidades do corpo, causando queda da saturação. ^817^ ^-^ ^819^

 Na população pediátrica é considerado critério de interrupção do esforço uma queda progressiva da saturação de oxigênio (<90%) ou queda de 10 pontos na saturação de repouso, associada a sintomas. ^820^

 Existem situações/condições em que a oximetria de pulso pode apresentar medições não confiáveis ( Tabela 39 ). ^817^

**Tabela 39:** – Causas e mecanismos de medições de SpO 2 não confiáveis 817-819

1. Causas de oscilações intermitentes ou incapacidade de medição da SpO _**2**_
• Má perfusão periférica (p. ex., hipovolemia, vasoconstrição etc.)
• Tremores (doença de Parkinson) e edema
** 2. Causas de SpO _**2**_ falsamente normal ou elevada **
• Envenenamento por monóxido de carbono
• Crises vaso-oclusivas de anemia falciforme
** 3. Causas de SpO _**2**_ falsamente baixa **
• Pulsações venosas (fístula arteriovenosa)
• Movimento excessivo
• Formas hereditárias de hemoglobina anormal
• Anemia grave (com hipoxemia concomitante)
• Esmalte
** 4. Causas de SpO _**2**_ falsamente baixa ou alta **
• Metemoglobinemia
• Sulfemoglobinemia
• Sepse e choque séptico
** 5. Causas de CO _**2**_ Hb falsamente baixo, conforme medido na CO-oximetria **
• Hiperbilirrubinemia grave
• Hemoglobina fetal (HbF)

## 7.3. Biomarcadores e Exames Laboratoriais

 A realização de exames laboratoriais e dosagem de biomarcadores adicionalmente ao TE/TCPE apresenta indicações específicas e, quando concomitantes, recomenda-se que sejam feitos em nível hospitalar. A dosagem do lactato sanguíneo e gasometria arterial são os exames mais frequentemente realizados. 

 Em pacientes com suspeita de DAC estável, a troponina T cardíaca ultrassensível (TroTUS) tem demonstrado utilidade clínica por seu valor preditivo isolado, bem como quando realizada complementarmente ao TE para diagnóstico de DAC. ^821^ ^-^ ^824^ Uma TropTUS em repouso >6,0 ng/L foi preditiva para DAC (RR: 2,55; IC 95%: 1,40-4,65; p=0,002) e, quando elevada durante o TE, aumentou a acurácia para 0,71, sendo marcador de risco para eventos cardíacos maiores. ^822^ ^,^ ^825^

**Figura 17:**
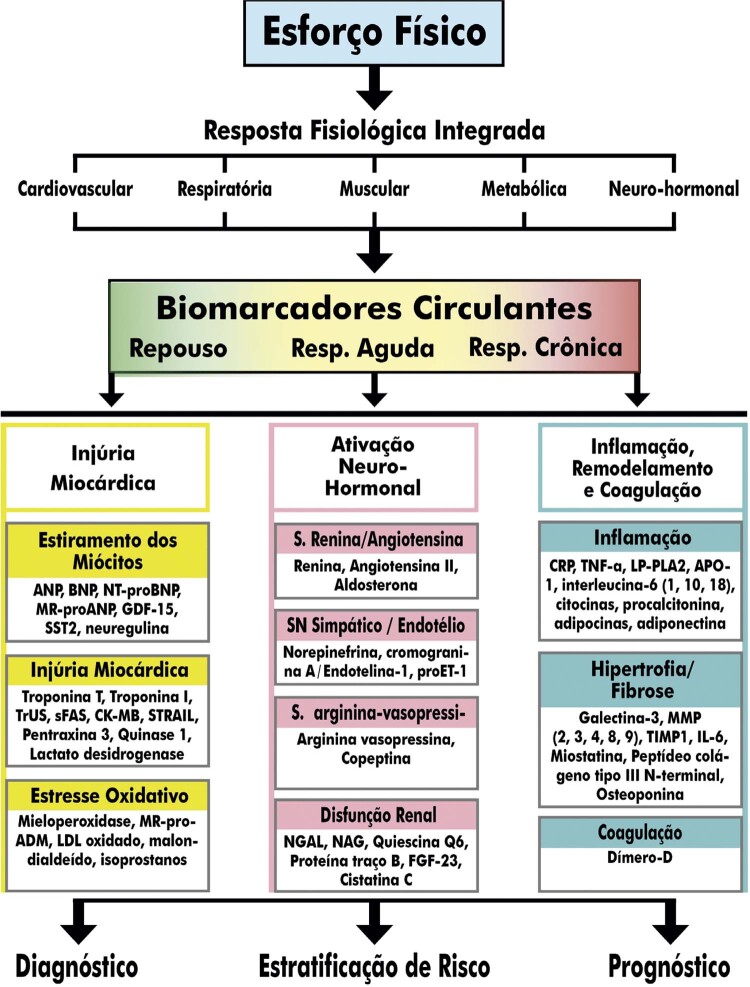
– Biomarcadores estudados na perspectiva de serem adicionados ao TE/TCPE. S.: sistema; SN: sistema nervoso; ANP: peptídio natriurético atrial; BNP: peptídio natriurético tipo B; NT-proBNP: peptídio natriurético tipo B – fragmento N-terminal inativo; GDF-15: fator-15 de diferenciação e crescimento; SST2: proteína receptora da interleucina-1; TrUS: troponina ultrassensível; CK-Mb: creatinoquinase fração MB; STRAIL: ligante indutor de apoptose relacionado ao fator de necrose tumoral; ADM: adrenomedulina; MR-pro-ADM: pró-adrenomedulina midregional; LDL oxidada: lipoproteína de baixa densidade oxidada; proET-1: peptídos precursores da endotelina 1; NGAL: lipocalina associada com gelatinase de neutrófilos humanos; NAG: N-acetil-Β-D-glucosaminidase; FGF-23: fator de crescimento de fibroblastos 23; CRP: proteína C-reativa; TNF-α: fator de necrose tumoral α; Lp-PLA2: fosfolipase A2 associada à lipoproteína (fator ativador de plaquetas); APO-1: apolipoproteína A-1; TIMP-1: inibidor tecidual de metaloproteinase 1; MMP: metaloproteinase; IL-6: interleucina-6.

 A Figura 17 apresenta outros biomarcadores que vêm sendo estudados nesta última década. Observa-se aumento contínuo das evidências científicas sobre a utilidade, a reprodutibilidade e a aplicabilidade dos biomarcadores, com perspectiva de serem incorporados à prática clínica. ^821^ ^,^ ^826^ ^-^ ^830^

Os exames laboratoriais podem ser feitos:

 – Fora do TE, podendo ser associados a exames laboratoriais de triagem/rotina. 

 – No pré-teste, para avaliação do estado basal do paciente. 

 – No esforço, necessitando de acesso venoso ou arterial puncionado no pré-teste e adequadamente preparado para coletas. Utilizado para avaliação do efeito agudo do esforço sobre o marcador estudado. 

 – Na recuperação, visando avaliar o efeito tardio do esforço ou retorno à condição de estado basal. 

 – Em 2 ou mais fases do TE (pré-teste e esforço; pré-teste e recuperação; esforço e recuperação; nas três fases). 

 Alguns desses biomarcadores são amplamente utilizados na prática clínica, outros ainda não apresentam evidências científicas que sustentem seu uso clínico. 

### 7.3.1. Lactato Sanguíneo

 A dosagem dos níveis séricos de lactato (NSL) é um dos melhores indicadores disponíveis para avaliar o metabolismo celular tanto em pacientes com morbidades quanto em atletas. Adicionado ao TE, os NSL podem otimizar a avaliação de doenças como: DAC, DPOC, insuficiência renal crônica e esclerose múltipla. Contribui para prescrição otimizada de exercícios, particularmente no contexto do esporte competitivo e programas de reabilitação ( Tabela 40 ). ^831^ ^,^ ^832^

**Tabela 40:** – Indicações de realização de dosagem de lactato e gasometria arterial adicionalmente ao TE/TCPE

Indicação	GR	NE
**Dosagem de lactato**		
Teste seriado em atletas competitivos de atividades predominantemente aeróbicas para ajustes de intensidade de cargas de treinamento ^840,841,855-857^	IIa	B
Liberação/prescrição de ajustes na prescrição de exercícios na reabilitação ^831,832,855,858^	IIb	A
Investigação de síndrome de excesso de treinamento ^859,860^	IIb	B
Avaliação do metabolismo celular em pacientes com morbidades (p. ex., DAC; DPOC; insuficiência renal crônica; esclerose múltipla) e estratificação de risco* ^831,832,855,858^	IIb	B
**Gasometria arterial**		
Investigação de dispneia/hipoxemia e suspeita de dessaturação ^861,862^	IIb	B
Suspeita de incompatibilidade da relação ventilação/perfusão (p. ex., insuficiência cardíaca, DPOC)* ^850,851,853,863^	IIb	B
Na DPOC, visando à determinação do volume de espaço morto fisiológico, hiperinsuflação dinâmica e suas inter-relações, para diagnóstico e prognóstico* ^854,864^	IIb	B
GR: grau de recomendação; NE: nível de evidência; IC: insuficiência cardíaca; DPOC: doença pulmonar obstrutiva crônica; DAC: doença arterial coronariana. *Normalmente associado ao TCPE.		

 Em pacientes com morbidades, apesar da complexidade das vias bioquímicas relacionadas à cinética do lactato sanguíneo, o NSL tem se mostrado melhor preditor prognóstico que as variáveis derivadas da oxigenação tecidual e o consumo de oxigênio. Nesses pacientes, parte do aumento do NSL pode estar associada à hipoxemia. ^833^

 A concentração normal de lactato no sangue em repouso é <2 mmol/L (18 mg/dL). Pacientes submetidos a TE com carga de esforço progressiva e incremental até a exaustão voluntária apresentam NLS significativamente elevado (≈8-10 mmol/L). O esforço é considerado máximo quando o NSL em adultos for >9 mmol/L (indivíduos normais) ou >5 mmol/L (com morbidades). ^834^ ^,^ ^835^ Entretanto, é na recuperação (entre 3 e 8 minutos) que são observados os níveis mais altos de lactato (≈15-25 mmol/L). ^836^

 Na medicina esportiva, é onde mais se tem indicado a realização da dosagem de NSL (podendo ser associada ao TCPE), para avaliação seriada do desempenho, ajustes de cargas de treinamento e determinação do limiar de lactato. Sugere-se: 

 – Utilizar protocolo em bicicleta ergométrica com carga inicial de 40W e incrementos de carga de 40W/4 min. ^835^

 – Quando disponível, adotar protocolo e ergômetro buscando simular a modalidade esportiva praticada, inclusive por permitir manutenção de esforço por período mais prolongado. ^837^ ^-^ ^839^

 – As tabelas de referência do NSL por percentil de acordo com a intensidade de esforço alcançado e/ou modalidade esportiva praticada necessitam de validação na população brasileira. 

 Em atletas competitivos, pode-se realizar análises adicionais NSL, tais como: comportamento da curva de NSL, padrão de recuperação, concentração máxima de lactato em estado estável, teste de lactato mínimo, entre outros. ^840^ ^,^ ^841^

### 7.3.2. Gasometria Arterial

 A gasometria arterial (GA), ou análise de gases no sangue arterial, é um exame complementar invasivo que tem por objetivo revelar valores de potencial de hidrogênio (pH) sanguíneo, da pressão parcial de gás carbônico (PaCO _2_ ou pCO _2_ ) e oxigênio (PaO _2_ ), íon-bicarbonato (HCO3) e saturação da oxi-hemoglobina, avaliando principalmente o equilíbrio acidobásico orgânico. 

 A GA basal (em repouso) permite: avaliar a adequação de ventilação, equilíbrio acidobásico e oxigenação; avaliar a resposta do paciente à terapia e/ou avaliação diagnóstica; monitorar a gravidade e progressão de doenças cardiorrespiratórias e metabólicas. Como indicações gerais da GA associada ao TE/TCPE, temos: ^842^ ^,^ ^843^

– Avaliação mais precisa de sintomas de dispneia.

– Diagnóstico diferencial de hipoxemia.

 – Doenças em que o acréscimo da GA auxiliará no diagnóstico, na segurança e na precisão da avaliação. Exemplos: doenças pulmonares em estágio avançado (DPOC e enfisema); pneumopata em associação à espirometria de repouso. 

 – Nos atletas de alto rendimento (principalmente *masters* ). 

 A indicação de realização da GA adicionalmente ao TE/TCPE não modifica suas indicações e graus de recomendação ( Tabela 40 ). 

 As coletas de amostras sanguíneas para a GA podem ser feitas por meio de: ^844^

 – Cateter arterial (geralmente na artéria radial) em membro superior, no qual não está sendo medida a PA. A técnica de coleta segue os procedimentos padrões da coleta da GA seguida de heparinização do cateter, de modo a permitir novas coletas. 

 – Sangue arterializado de lobo de orelha não é recomendado, por apresentar baixa correlação com os valores de PaO _2_ e PaCO _2_ . ^845^ ^,^ ^846^

 Critérios mínimos para considerar que a carga de esforço atingida foi máxima, baseado na gasometria: ^847^ ^,^ ^848^

– pH: <7,25.

 – Excesso de base: <9 mmol/L (indivíduos normais), <5 mmol/L (com morbidades). ^134^ ^,^ ^849^

 A gasometria arterial é necessária para o cálculo da relação espaço morto em relação ao volume corrente (VD/VT) corrigida pelo espaço morto mecânico durante o TCPE. Essa correção produz resultados quantitativa e qualitativamente diferentes, o que pode ter um impacto importante na interpretação da incompatibilidade V/Q. ^850^ ^-^ ^852^

Particularidades da GA adicionalmente ao TE/TCPE:

 – Na IC, pode ocorrer aumento progressivo da PaCO _2_ com o esforço devido à área pulmonar mal ventilada. Na IC grave, o aumento de VD/VT e PaCO _2_ na ausência de hipoxemia associa-se a grande incompatibilidade da relação ventilação/perfusão. ^850^ ^,^ ^853^

 – Na ICFEP, pode ocorrer incompatibilidade V/Q e piora na eficiência das trocas gasosas, refletidas por um aumento do VD/VT. ^850^ ^,^ ^851^

 – Em tabagistas, a relação VD/VT elevada (com aumentos compensatórios na ventilação minuto) associa-se a maior grau de dispneia e intolerância ao esforço. ^854^

##  8. Particularidades na Realização e Interpretação do TE em Condições Clínicas Específicas 

###  8.1. Dextrocardia/ *Situs Inversus*

 Dextrocardia (DxC) é uma anomalia congênita na qual o coração se posiciona no lado direito do tórax e apresenta inversão de suas câmaras, similar a uma “imagem em espelho” ( *situs inversus* ). Sua prevalência varia de 1/6.000 a 1/35.000 nascimentos. Quando associada à inversão dos órgãos abdominais, é denominada *situs inversus totalis* . ^865^ ^,^ ^866^

 Geralmente é assintomática, mas também pode estar associada a malformações cardíacas congênitas, sendo as principais: *no situs solitus* , discordância atrioventricular com a obstrução da via de saída do ventrículo direito; no *situs inversus* , dupla via de saída do ventrículo direito com obstrução do trato de saída. ^867^

 DxC não deve ser confundida com dextroposição do coração, situação na qual não há inversão das câmaras cardíacas e o coração está deslocado para o hemitórax direito (devido, por exemplo, a pneumectomia ou grande derrame pleural esquerdo). Nesse caso, não há indicação de modificação da forma de monitorização eletrocardiográfica para o TE. ^868^

 A DxC geralmente é diagnosticada na infância e o paciente costuma informá-la no pré-teste. O seu reconhecimento no exame físico (ausculta de bulhas cardíacas e ictus em hemitórax direito) e/ou alterações no ECG de repouso contribui para evitar eventuais erros diagnósticos. 

 Deve-se suspeitar de DxC se, no ECG de repouso, houver onda P, QRS e onda T negativas na derivação D1. Nessa situação, deve-se afastar a troca de eletrodos dos membros superiores e verificar a progressão da amplitude das ondas R nas derivações precordiais (na dextrocardia, não aumentam de V1 a V6). ^869^

 Em pacientes sabidamente portadores de DxC, recomenda-se: 

 1) Documentar o ECG de repouso com os eletrodos nas posições clássicas, considerando que: 

 1.1) No *situs inversus* , observa-se: ^870^ ^,^ ^871^

 – Onda P invertida em DI e AVL e uma onda P positiva em AVR devido ao nó sinusal localizado à esquerda (espelhado). 

 – Ativação ventricular invertida com complexo QRS e onda T negativos em DI. As derivações precordiais direitas espelham as derivações precordiais esquerdas de um coração normal. 

 – Ondas Q presentes nas derivações precordiais direitas devido à despolarização septal direita-esquerda pelo posicionamento em espelho do coração. 

 1.2) No *situs solitus* , observa-se: ^870^ ^,^ ^872^

 – Despolarização atrial progredindo normalmente, independentemente da localização cardíaca (hemitórax direito ou esquerdo). 

 – Despolarização ventricular no sentido anti-horário e normalmente com ondas Q nas derivações DI, AVL e precordiais esquerdas (devido à despolarização septal apropriada). 

 1.3) No *situs* ambíguo e isomerismo à direita, as ondas P podem ter diferentes origens, pois representam a atividade de marca-passos atriais bilaterais. No entanto, o eixo da onda P ainda pode ser normal se o nó sinusal direito atuar como marca-passo dominante. Devido à ausência de um nó sinusal funcional, os pacientes com isomerismo esquerdo apresentam marca-passo atrial ectópico com onda P geralmente anormal. Com a idade, esse marca-passo ectópico sofre desaceleração progressiva da FC e a maioria dos pacientes necessita de implante de marca-passo permanente. 

 2) No TE, ajustar a monitorização eletrocardiográfica para cada tipo de DxC: 

 2.1) No *situs inversus* , tanto os eletrodos dos membros quanto das derivações precordiais devem ser invertidos: 

 – Na posição do V2 (no 4º espaço intercostal, na linha paraesternal esquerda) colocar o eletrodo V1 (V1R). 

 – Na posição do V1 (no 4º espaço intercostal, na linha paraesternal direita) colocar o eletrodo V2 (V2R). 

– V3R: entre os eletrodos V2R e V4R.

 – V4R: no 5º espaço intercostal direito, na linha hemiclavicular direita. 

 – V5R: no 5º espaço intercostal direito, na linha axilar anterior. 

 – V6R: no 5º espaço intercostal direito, na linha axilar média. 

 – Inversão dos eletrodos dos braços e pernas (direita para esquerda e vice-versa). 

 – Nos sistemas de 13 derivações, a derivação CM5 (manúbrio) é mantida no mesmo local. 

 – É obrigatório acrescentar no laudo a realização da inversão das posições dos eletrodos e adicionar uma letra R ou letra D (abreviação de direita) depois da denominação das derivações. 

 2.2) No *situs solitus* com DxC, os eletrodos das derivações precordiais devem ser invertidos (V1 a V6 posicionados em hemitórax direito, semelhante ao *situs inversus* ), enquanto os eletrodos dos membros devem permanecer inalterados. 

 2.3) No situs ambíguo, não se recomenda modificar a posição dos eletrodos do ECG convencional. ^872^

 O TE realizado com os eletrodos nessas posições recomendadas permite a adequada avaliação de todas as variáveis eletrocardiográficas e suas respectivas interpretações. 

 A prevalência de arritmias em pacientes com DxC é significativamente maior do que na população geral (RR: 2,60; IC 95%: 1,67-4,06; p<0,001), sendo a mais comum a fibrilação atrial/flutter atrial (RR: 3,06; IC 95%: 1,02-9,18; p=0,046). ^873^

 Atentar para a possibilidade de existência de malformações cardíacas associadas à DxC e suas eventuais interferências nas respostas hemodinâmicas, funcionais e no ECG do TE. ^870^ ^,^ ^874^ ^,^ ^875^

### 8.2. Doença de Chagas/Cardiomiopatia Chagásica

 A doença de Chagas (DCh) continua sendo um grave problema de saúde pública mundial requerendo medidas adequadas para o seu diagnóstico, tratamento e seguimento. Na América Latina, estima-se que ≈6 milhões de pessoas estejam infectadas, das quais 30% a 40% podem cursar com a forma cardíaca com mortalidade ≈24,5 por 1.000 pacientes/ano. ^876^ ^,^ ^877^

 A DCh apresenta amplo espectro de apresentação clínica: forma indeterminada, clinicamente inaparente; cardiomiopatia chagásica (CCh), com comprometimento cardíaco, alterações no ECG e sorologia positiva para Trypanosoma cruzi; e CCh dilatada, com IC e disfunção ventricular. As arritmias cardíacas (síndrome arrítmica) podem ser a única manifestação da CCh, frequentemente ocorrendo em combinação com IC e/ou eventos tromboembólicos. ^878^ ^,^ ^879^

 O TE é útil em todas formas de apresentação da DCh, inclusive devido à existência frequente de comorbidades. Indicações gerais do TE na DCh: ^879^ ^,^ ^880^

 – Avaliação da capacidade funcional, resposta cronotrópica e comportamento pressórico. ^881^ ^-^ ^883^

 – Avaliação de sintomas, inclusive dor torácica. ^884^

 – Avaliação de comorbidades (p. ex., HAS, DAC etc.). 

 – Detecção e avaliação do comportamento de arritmia (suspeita ou conhecida) e distúrbios da condução atrioventricular. ^885^

 – Estratificação de risco para morte súbita (inclusive como parte dos critérios do escore de Rassi). ^878^ ^,^ ^886^ ^,^ ^887^

 – Avaliação seriada, inclusive em programa de reabilitação para prescrição de exercícios. ^888^

 – Perícia médica e/ou avaliação pela medicina do trabalho. ^889^

Particularidades da realização do TE na DCh:

 – No ECG de repouso, nenhum achado eletrocardiográfico isolado é patognomônico, sendo comuns alterações múltiplas, inclusive decorrentes de comorbidades. ^890^

 – Alterações do ECG de repouso potencialmente relacionadas ao mau prognóstico: ondas Q patológicas (simulando IAM prévio), baixa voltagem dos complexos QRS e alterações primárias da onda T. ^886^ ^,^ ^891^

 – Capacidade funcional relativamente preservada na forma indeterminada e na CCh crônica mesmo em presença de alterações do ECG e função ventricular deprimida. ^892^ ^,^ ^893^ Comprometimento funcional é preditor de declínio da função cardiovascular, inclusive na forma indeterminada e em estágios iniciais da CCh. Na CCh dilatada, verificou-se que o VO _2_ pico foi preditor independente de morte. ^883^ ^,^ ^885^ ^,^ ^894^

 – Recomenda-se adequar o protocolo de esforço à classe funcional, inclusive com eventual utilização de protocolos atenuados. 

 – Resposta cronotrópica diminuída e/ou incompetência cronotrópica são consideradas manifestações de disfunção autonômica secundária à DCh. Essa disautonomia geralmente associa-se com a densidade e letalidade das arritmias ventriculares. ^895^ ^,^ ^896^

 – Pacientes com DCh na forma indeterminada e na forma digestiva isolada podem apresentar incompetência cronotrópica ou arritmias ventriculares esforço-induzidas. ^881^ ^,^ ^897^

 – Alterações do segmento ST esforço-induzidas podem ser secundárias a aneurisma apical do VE, principalmente na síndrome de dor torácica da DCh. No entanto, essas anormalidades podem ser inerentes à DCh (aguda ou crônica). ^898^

 – Foi demonstrado que a ocorrência de dor precordial associada a alterações isquêmicas de ST (em ECG possível de interpretação) tem VPP de 100% para DAC obstrutiva. ^884^

 – Alterações eletrocardiográficas basais associadas aos distúrbios de condução intraventricular geralmente impossibilitam a adequada avaliação de isquemia pelo segmento ST. ^884^

 – Alterações do ritmo cardíaco no ECG basal são preditoras de gravidade/classe funcional da DCh, enquanto as esforço-induzidas se correlacionam com mortalidade CV. ^899^ ^,^ ^900^

 – Ocorrência de TVNS, em especial nos pacientes com disfunção ventricular esquerda, é preditora de morte súbita cardíaca. ^877^ ^,^ ^901^ ^,^ ^902^

 – Ocorrência de arritmia ventricular esforço-induzida ou aumento de sua densidade em relação ao ECG basal (em mais de 10%) estão associados ao risco de morte CV. ^877^ ^,^ ^903^

 – A morte súbita na CCh, geralmente precipitada pelo esforço, pode ser causada por taquicardia ou fibrilação ventricular, assistolia e BAVT. ^904^

 – Na prescrição de exercício e avaliação seriada em programa de reabilitação, o TE deve ser realizado mantendo as medicações usuais, principalmente para pacientes com drogas cronotrópicas negativas, como betabloqueadores, digitálicos ou antiarrítmicos, para mimetizar a condição em que estarão durante as sessões de treinamento físico. Sugere-se que associe ao TE, a monitorização da saturação de oxigênio e variáveis cardiometabólicas (TCPE) visando melhor quantificação de possíveis limitações. ^905^

 – Na prescrição de exercício/avaliação seriada em programa de reabilitação, o TE deve ser realizado mantendo as medicações de uso corrente (inclusive drogas cronotrópicas negativas) preservando a condição do paciente nas sessões de treinamento físico. ^888^

### 8.3. Doença Arterial Periférica

 A doença arterial periférica (DAP) é caracterizada por lesões ateroscleróticas das artérias dos membros inferiores que causam redução do fluxo sanguíneo, claudicação intermitente (CI), dor muscular isquêmica esforço-induzida e alívio da dor com o repouso (geralmente dentro de 10 minutos). ^175^ ^,^ ^780^ Podem ocorrer sintomas atípicos como cãibras e limitações aos esforços sem sintomas claramente associados aos membros inferiores. ^906^ Estima-se que, no mundo, a DAP afete mais de 200 milhões de pessoas, sendo sua apresentação e progressão dos sintomas influenciadas por sexo, idade e etnia do paciente. A DAP frequentemente ocorre em pacientes tabagistas e diabéticos. ^907^

 A CI ocorre entre 7,5% e 33% dos pacientes, diminuindo progressivamente a capacidade de caminhada, a capacidade funcional e a qualidade de vida. O ITB em repouso é o principal exame para o diagnóstico e estabelecimento da gravidade da DAP, independentemente dos sintomas. ^780^ ^,^ ^908^

 Pacientes com suspeita ou diagnóstico confirmado de DAP apresentam risco aumentado de doença arterial obstrutiva em outras partes do corpo, em especial na artéria subclávia, coronárias e artéria renal. Pacientes com DAP e DAC apresentam pior evolução da doença arterial periférica, aumento de mortalidade CV e por todas as causas. ^175^ ^,^ ^176^ ^,^ ^909^

 Em linhas gerais, o TE é indicado na DAP para avaliar sintomas de claudicação/dor em membros inferiores, quantificar possível isquemia, avaliar a capacidade funcional, estratificação de risco, prescrição de programa de exercícios físicos e ajustes terapêuticos. ^175^ ^-^ ^177^ ^,^ ^781^ ^,^ ^801^

Particularidades da realização do TE na DAP:

 1) Respeitar as orientações pré-teste principalmente quanto à suspensão do tabagismo e evitar esforços físicos antes do TE. 

 2) No pré-teste, é recomendada a realização do exame vascular, incluindo palpação dos pulsos dos membros inferiores, ausculta de sopros femorais e inspeção das pernas e pés. Os pulsos devem ser avaliados e classificados quanto sua amplitude: 0 = ausente (pulso não palpável); 1 = diminuído (pulso pouco palpável); 2 = normal; 3 = aumentado. Atentar para presença de lesões de pele, agudas ou crônicas, principalmente em diabéticos (pé diabético). ^781^

 3) Recomenda-se utilizar protocolo atenuado de esforço: 

 – Com carga constante (sem incrementos de velocidade ou inclinação), com velocidade de 2 mph (3,2 km/h) e inclinação de 10% a 12%. Esse protocolo pode não permitir avaliação precisa de indivíduos com DAP e grave limitação funcional por dificuldade em manter o esforço nessa inclinação. 

 – Ou com incremento de carga de forma escalonada (p. ex., protocolo de Naughton) ou rampa (com velocidade ajustada para incrementos pequenos de carga, pequena ou nenhuma inclinação). Quando utilizado protocolo de rampa, recomenda-se descrever no laudo as velocidades e inclinações, inicial e final, bem como a carga em METs/min programada de modo a permitir reprodutibilidade do protocolo. 

 4) O paciente deve ser orientado que, caso necessite, poderá utilizar o apoio das mãos nos corrimões da esteira. Registrar no laudo se o paciente fez ou não uso do apoio das mãos. 

 5) O esforço deve ser interrompido quando o paciente apresentar claudicação de intensidade limitante e/ou dor máxima tolerada em membro inferior. Também poderá ser interrompido por outros sintomas CV (p. ex., dor precordial, dispneia etc.) e demais parâmetros que contraindiquem a continuidade do esforço. ^906^

 6) Registrar detalhadamente todos os sintomas ocorridos, quando, em qual parte (nádega, coxa, panturrilha ou outros) e momento de melhora. Atenção especial para o registro do momento de início da claudicação (claudicação inicial) e da claudicação que limita/impede a caminhada (claudicação absoluta). Sugere-se utilizar escala de percepção subjetiva de esforço (Borg ou Borg modificada) e escala de claudicação intermitente ( Figura 7 ). 

 7) Na recuperação, realizar novamente o exame vascular (ver item 2) e somente liberar o paciente após o reestabelecimento da condição basal. 

 8) É imprescindível registrar no laudo a distância máxima de caminhada livre de dor e/ou claudicação, a distância percorrida até a claudicação inicial e/ou absoluta, METs alcançados e capacidade funcional do paciente. 

 Dependendo do quadro do paciente e julgamento clínico, pode ser necessária a realização, simultânea/adicional ao TE, do exame de ITB pós-esforço, cujas indicações, modo de realização e interpretação constam na Seção 7.1 da Parte 2 desta diretriz. 

Particularidades do TE na DAP:

 – A Tabela 41 apresenta os principais diagnósticos diferenciais de dor e claudicação esforço-induzidas ao TE quando não provindas da DAP. 

**Tabela 41:** – Dor em membros inferiores e claudicação esforço-induzidas não relacionadas à DAP

Doença	Localização	Característica	Esforço	Recuperação	Particularidades
**Claudicação venosa**	Perna inteira, mais acentuada na panturrilha	Dor em aperto e súbita	Depois de caminhar	Desaparece lentamente	Alívio com elevação. História de: TVP iliofemoral; edema; sinais de estase venosa
**Síndrome compartimental crônica**	Musculatura da panturrilha	Dor em aperto e explosiva	Após esforço intenso	Desaparece muito lentamente	Alívio com descanso. Mais frequente em atletas com grande massa muscular
**Estenose espinhal**	Frequentemente nádegas bilaterais, perna posterior	Dor e fraqueza	Pode imitar claudicação	Alívio variável, podendo demorar para recuperar	Alívio com flexão da coluna lombar. Piora com o ortostatismo
**Hérnia de disco/compressão de raiz nervosa**	Irradia na extensão da perna	Dor lancinante aguda	Deflagrada ao sentar, ficar em pé ou caminhar	Frequentemente presente em repouso	Pode melhorar com mudança de posição. Frequentemente histórico de lombalgia
**Artrite do pé/tornozelo**	Tornozelo, pé, arco do pé	Dor profunda	Após esforço (independentemente de intensidade)	Alívio lento	Sintomas variáveis em repouso e/ou esforço-induzidos
* TVP: trombose venosa profunda. Adaptada de: Gerhard-Herman MD et al. ^*781*^*					

 – Estudos demonstraram que, em pacientes com DAP, as variáveis do TE são úteis para estratificação de risco de eventos cardíacos maiores e mortalidade cardiovascular. ^803^ ^,^ ^910^

 – A redução da capacidade funcional ao TE é forte preditor de mortalidade a longo prazo em pacientes com DAP e supera todos os fatores de risco clássicos (incluindo tabagismo e IC) na estratificação de risco. ^177^ ^,^ ^911^

 – Estudos em pacientes com DAP submetidos ao TE para prescrição e ajustes de programas de exercícios físicos demonstraram melhora da saúde cardiovascular (melhora do comportamento da PA, FC, recuperação da FC e capacidade funcional) e correlacionou-se com melhora no desempenho à caminhada. ^781^ ^,^ ^912^

8.4. Doença de Parkinson

 A doença de Parkinson (DPark) é o segundo distúrbio neurodegenerativo mais comum no mundo, afetando 0,4% das pessoas <40 anos e cerca de 1,6% das pessoas com ≥65 anos. É caracterizada por disfunções motoras clássicas (bradicinesia, rigidez e tremor de repouso), com redução da atividade parassimpática e simpática, em repouso e no esforço. ^913^ ^,^ ^914^

 As disfunções autonômicas e cardiovasculares são comuns na DPark, precedendo a disfunção motora em pelo menos uma década. A DPark geralmente evolui com fadiga, redução da capacidade funcional e baixa qualidade de vida. A presença de corpos de Lewy distribuídos no hipotálamo, centros simpático e parassimpático compromete a modulação autonômica, cronotropismo, inotropismo e resistência vascular periférica. ^915^ ^,^ ^916^

 Na DPark, a regulação anormal da PA pode se manifestar como: 

 – Hipotensão ortostática (HO) que afeta ≈50% dos pacientes. ^917^ ^,^ ^918^

 – Hipertensão em decúbito dorsal (hipertensão supina – HASup) é definida como PAS >140 mmHg e/ou PAD >90 mmHg quando medida após 5 minutos de repouso supino, tem prevalência de 79% e aumenta a incidência de lesões em órgãos-alvo (fator de risco para AVC e eventos CV maiores). ^915^ ^,^ ^919^

– Associação de HASup com HO é frequente (95%).

Particularidades do TE na DPark:

 1) Atentar para possibilidade de ocorrência de HASup, bem como de HO na fase pré-teste. 

 2) A individualização do protocolo deve ser cuidadosa. Sugere-se utilizar protocolo atenuado nos pacientes com maior limitação física. 

 3) Nos pacientes com HO sintomática e/ou história de síncope esforço-induzida, recomenda-se a realização do TE em bicicleta ergométrica visando reduzir o risco de queda. 

 4) Sugere-se o apoio das mãos nos corrimões da esteira ou guidom da bicicleta, inclusive para redução do tremor de membros superiores. 

 5) Na DPark inicial ou de leve intensidade, é possível que não haja limitação funcional e comprometimento hemodinâmico relevante. ^167^ ^,^ ^920^

 6) Registrar o comportamento da marcha durante o TE, inclusive se ocorreu ou não episódios de congelamento (quando a deambulação e outros movimentos voluntários podem cessar repentinamente). A marcha característica da DPark é tipo festinação, com diminuição dos passos largos, diminuição da cadência e velocidade de movimentos e distúrbios na amplitude do movimento. ^921^

 7) Pacientes com DPark e HO geralmente têm aumento reduzido da PA ao esforço, podendo apresentar hipotensão esforço-induzida. Esse comportamento da PA, associado à vasoconstrição reduzida em leitos vasculares periféricos, pode acentuar o desequilíbrio metabólico, hipoperfusão tecidual/encefálica, causando maior fadiga e menor tolerância ao esforço. ^920^ ^,^ ^922^

 8) Na DPark sem HO e com capacidade funcional reduzida, geralmente ocorre menor PASpico, menor FCpico e resposta cronotrópica deprimida. ^167^ ^,^ ^914^ ^,^ ^923^

 A prática de exercícios físicos se constitui em abordagem não farmacológica, preventiva e terapêutica, relevante em termos de custo-efetividade, retardando o desenvolvimento de DPark, controlando sintomas (motores e não motores) e reduzindo o risco de eventos CV. ^921^ ^,^ ^924^ ^,^ ^925^ O TE está indicado para avaliação da aptidão cardiorrespiratória, liberação/prescrição de exercícios físicos, inclusive no contexto da reabilitação cardiovascular. ^926^ ^,^ ^927^

8.5. Doenças Valvares

 O TE é relevante na avaliação dos pacientes com doença valvar (DV) contribuindo para a investigação de sintomas, ajustes terapêuticos, indicação de tratamento invasivo, liberação/prescrição de exercícios físicos, inclusive na reabilitação e prática esportiva. ^93^ ^,^ ^94^ ^,^ ^240^

 Pacientes com DV costumam reduzir as atividades físicas, de forma gradual e imperceptível (sedentarismo), dificultando a percepção de sintomas esforço-induzidos. Nesses pacientes, o TE tem papel fundamental para definição de *status* assintomático. ^17^ ^,^ ^928^

 Na DV leve, o TE geralmente é bem tolerado e seguro. Entretanto, em algumas situações específicas, pode apresentar maior risco de complicações e eventos adversos, sendo necessária a adoção de medidas preventivas específicas (p. ex., realização em nível hospitalar; utilização de protocolo atenuado etc.) ou até sua contraindicação. 

 As situações de contraindicação relativa e absoluta foram descritas na Parte 1, Seção 2.3 desta diretriz. Recomenda-se a realização do TE em ambiente hospitalar com suporte cardiológico nos pacientes assintomáticos com lesões valvares graves (estenóticas ou insuficientes), nos pacientes com lesões valvares múltiplas (moderadas/ graves) e com cardiopatia congênita associada. 

 Adequações na metodologia do TE na DV: ^13^ ^,^ ^112^ ^,^ ^293^

 – A anamnese pré-teste deve avaliar a etiologia, gravidade e evolução da DV, medicação em uso, presença de sintomas e identificação de contraindicações relativas e absolutas. 

 – Contraindicações absolutas ao TE: indicações claras para intervenção cirúrgica valvar, hipertensão não controlada, arritmias complexas ou doença sistêmica com incapacidade de se exercitar adequadamente. ^14^ ^,^ ^929^

 – No exame físico, além da ausculta cardíaca e pulmonar, realizar a pesquisa de frêmitos e palpação de pulsos periféricos. 

 – O protocolo deve considerar a capacidade funcional do paciente e evitar grandes incrementos carga de esforço (protocolo atenuado). ^930^

 – No TE seriado, deve-se, preferencialmente, manter o protocolo utilizado anteriormente, visando a comparação de sintomas, comportamento hemodinâmico e capacidade funcional. 

 – O TE deve ser sintoma-limitante, bem como buscar realizar recuperação ativa. 

 – São fundamentais a observação, a caracterização e a descrição pormenorizada de todos os sinais e sintomas ocorridos e, em especial, fadiga, dispneia, tontura, dor precordial, palidez e sudorese, por serem critérios de intervenções terapêuticas e de prognóstico. ^931^

 Abordaremos a seguir as particularidades referentes a doença valvar mitral e aórtica devido às suas incidências e disponibilidade de evidências científicas específicas quanto ao TE. 

#### 8.5.1. Estenose Aórtica

 A estenose valvar aórtica (EAo) tem como principais etiologias a doença reumática, a degenerativa (ou aterosclerótica) e a congênita. É uma das formas mais comuns de valvopatia adquirida e ≈3% da população brasileira >75 anos apresenta EAo grave. ^96^ ^,^ ^103^

 A tríade clássica de sintomas é composta de angina, síncope e dispneia. Após início dos sintomas, é necessária uma intervenção cirúrgica imediata devido à piora no prognóstico e uma sobrevida média de até 2 anos (se não associada a IC). A morte súbita cardíaca é a complicação mais temida, mas raramente observada em pacientes verdadeiramente assintomáticos. 

 O TE pode ser realizado com segurança em pacientes assintomáticos ou com sintomas leves. ^934^ É indicado para esclarecer sintomas duvidosos, avaliar a capacidade funcional, confirmar a ausência de sintomas ou diminuição na tolerância ao esforço. Quando normal, indica probabilidade muito baixa de sintomas e/ou complicações no seguimento de 6 a 12 meses. ^97^ ^,^ ^935^

 A incidência de um TE anormal dependerá da gravidade da EAo, variando de 25% a 65%. Metade dos pacientes com EAo grave assintomática apresentará TE anormal. ^108^ ^,^ ^936^ ^,^ ^937^

 As diretrizes nacionais e internacionais consideram os seguintes achados do TE para indicação de substituição cirúrgica da válvula aórtica: ^92^ ^-^ ^94^ ^,^ ^109^

 – Sintomas esforço-induzidos claramente relacionados à estenose aórtica. 

– Queda na pressão arterial intraesforço.

 – Diminuição da tolerância ao esforço/capacidade funcional. 

Particularidades do TE na EAo:

 – ECG de repouso na EAo grave geralmente apresenta HVE (em 85% dos casos), associada a alterações secundárias da repolarização ventricular. O ISTs (>1 mm) é observado em cerca de dois terços dos pacientes, mesmo com EAo leve/moderada. Outras alterações: sobrecarga atrial esquerda; bloqueio do ramo esquerdo; FA, principalmente em idosos hipertensos. ^112^ ^,^ ^115^

 – Cerca de um terço dos pacientes assintomáticos no basal desenvolve sintomas ao esforço, indicando alta probabilidade de desenvolvimento de sintomas em repouso e/ou complicações dentro de 12 meses. ^935^ ^,^ ^938^

 – Ocorrência de dispneia em altas cargas de esforço e rápida melhora na recuperação, bem como dispneia em portadores de DPOC (especialmente em idosos), podem ser achados inespecíficos. ^112^

 – Vertigem, pré-síncope e síncope aumentam o VPP para o desenvolvimento dos sintomas em repouso em acompanhamento por 1 ano. ^937^

 – O TE não é útil para a detecção de DAC na EAo (≈20% apresentam DAC obstrutiva assintomática sem ISTE). Metade dos pacientes apresenta ISTE (horizontal ou descendente, >2 mm), sendo marcador de alto risco geralmente não associado a DAC. ^934^ ^,^ ^938^

 – É frequente que os pacientes atinjam a FC submáxima prevista para a idade, mas sem aumento adequado do débito cardíaco (geralmente de apenas 50%). ^939^

 – Aumento precoce da FC (85% da FCmax prevista ou aumento ≥50% da FC nos primeiros 6 minutos), em pacientes com EA grave associou-se à necessidade de troca valvar (RR: 3,21; IC 95%: 1,70-6,08; p<0,001) e, na EA moderada, ao risco de morte por todas as causas (RR: 16,02; IC 95%: 1,83-140,02; p=0,012). ^374^ ^,^ ^938^

 – A resposta pressórica ao esforço é considerada anormal quando ocorrer queda intraesforço ou elevação da PAS <20 mmHg e está associada a maior ocorrência de sintomas. ^240^ ^,^ ^930^ ^,^ ^938^

 – A resposta hipertensiva (exagerada ao esforço) está relacionada a valores elevados de PAS em repouso, maior massa do VE e aumento da rigidez arterial. Entretanto, não se associa a maior incidência de sintomas ou capacidade funcional reduzida. ^940^ ^,^ ^941^

 – Arritmias ventriculares complexas esforço-induzidas são motivo de interrupção do TE, critério de anormalidade do exame e marcador de mau prognóstico. Arritmias ventriculares na recuperação têm correlação limitada com gravidade e prognóstico da EAo. ^939^ ^,^ ^942^

 – Baixa capacidade funcional na EAo grave assintomática associa-se a aumento de mortalidade. ^943^

#### 8.5.2. Regurgitação Aórtica

 A regurgitação aórtica (RAo) pode ser decorrente de uma anomalia primária, como a valva aórtica bicúspide (pacientes jovens) ou por degeneração (em idosos). Pode ser também secundária à cardiopatia reumática (principal causa) ou dilatação da raiz da aorta (hipertensão crônica, síndrome de Marfan etc.). No estudo de Framingham, a prevalência geral foi de 4,9% e de 0,5% de RAo moderada ou grave. ^104^ ^,^ ^944^

 A maioria dos pacientes permanece assintomática durante décadas. Nos assintomáticos com função sistólica de VE normal, a mortalidade é <0,2% ao ano. Com a função deprimida, a maioria desenvolve sintomas (taxa média >25% ao ano) e requer intervenção cirúrgica em 2 a 3 anos. Dispneia, angina ou IC são marcadores de mau prognóstico: mortalidade >10% ao ano no caso de angina e >20% nos casos de IC. Outros preditores de desfechos desfavoráveis são idade, volume sistólico final do VE e aptidão cardiorrespiratória. O início dos sintomas, mesmo que leves, é uma indicação para intervenção cirúrgica, independentemente da função de VE. ^102^ ^,^ ^105^ ^,^ ^945^

 O TE é realizado para esclarecimento de sintomas. A rápida progressão das dimensões ventriculares ou declínio na capacidade funcional em TE seriados é razão para se considerar a abordagem cirúrgica. ^97^ ^,^ ^946^

Particularidades do TE na RAo:

 – O ECG de repouso pode ser normal no início da doença ou mostrar hipertrofia do VE. Inicialmente, com a sobrecarga de volume do VE, ocorrem ondas Q proeminentes nas derivações DI, AVL e de V3 a V6. À medida que a RAo progride, ocorre diminuição das ondas Q com aumento progressivo da amplitude total dos complexos QRS. ^112^ ^,^ ^947^

 – No esforço, é comum a ocorrência de ISTE (>1,0 mm), geralmente não associado com DAC obstrutiva. ^948^

 – Arritmia ventricular esforço-induzida é relativamente comum e têm correlação significativa com o grau de hipertrofia e disfunção do VE. ^949^

 – É muito rara a ocorrência de dispneia em baixa carga de esforço e função sistólica normal. Em pacientes com sintomas esforço-induzidos, atentar para outros sinais de disfunção VE. ^950^

 – Assintomáticos com RAo moderada a grave podem realizar exercícios físicos de maior intensidade, e os com RAo grave, exercícios de intensidade moderada desde que o VE e a aorta não estejam com dilatação e apresentem FE>50%. ^951^ ^,^ ^952^

#### 8.5.3. Estenose Mitral

 A causa mundial mais comum da estenose mitral (EM) é a febre reumática. A EM isolada é duas vezes mais frequente em mulheres do que em homens. Outras causas de EM são raras e incluem anomalias congênitas, exposição prévia a radiação no tórax, mucopolissacaridose, mixoma de átrio esquerdo e calcificação anular mitral secundária ao envelhecimento. Os pacientes geralmente apresentam intolerância ao esforço e IC direita devido ao desenvolvimento de hipertensão pulmonar pós-capilar. ^92^ ^,^ ^93^ ^,^ ^240^ ^,^ ^953^

 O TE é útil para esclarecimento de sintomas duvidosos ou discordantes com a gravidade da EM, tendo revelado a presença de sintomas em até 46% de pacientes com EM moderada a grave antes considerados assintomáticos. O TE permite avaliar a fadiga e aptidão cardiorrespiratória na EM significativa (área valvar ≤1,5 cm ^2^ ). ^931^ ^,^ ^954^

Particularidades do TE na EM:

 – Deve ser realizado em uso de medicamentos (manter inclusive digoxina e betabloqueador). 

 – O ECG de repouso geralmente apresenta sobrecarga atrial esquerda, extrassistolia atrial ou fibrilação atrial (intermitente ou persistente). Na EM com HAP grave e/ou de longa duração, podem ser encontrados hipertrofia de ventrículo direito, desvio do eixo para direita e BRD. ^112^

 – No esforço, é comum a ocorrência de aumento exagerado da FC devido à redução do volume sistólico (principalmente na FA). Pode ocorrer ISTE geralmente sem associação com DAC obstrutiva. ^955^

 – Foi demonstrada ocorrência de arritmias ventriculares esforço-induzidas em 60% dos pacientes com EM (20% com arritmia complexa). A incidência e a complexidade não estiveram associadas à gravidade da estenose. ^956^

 – A dispneia tem valor prognóstico, sendo o sintoma mais frequente. A tolerância ao esforço tem boa correlação com a gravidade da lesão. Na EM grave, o aumento da pressão pulmonar com o esforço pode cursar com congestão pulmonar. ^106^ ^,^ ^957^

 – Dor precordial esforço-induzida geralmente está associada à inadequada elevação da PAS (resposta inotrópica deprimida), sem relação com DAC. ^106^ ^,^ ^240^

#### 8.5.4. Regurgitação Mitral

 A regurgitação mitral (RM) primária decorre de uma anormalidade estrutural da válvula (como na cardiopatia reumática), formas degenerativas (doença mixomatosa e deficiência fibroelástica) ou após uma endocardite. A RM secundária (funcional) geralmente ocorre devido à cardiomiopatia dilatada, isquêmica ou secundária a IAM. Afeta ≈24 milhões de pessoas em todo o mundo, com grande variação entre os países. ^958^ ^,^ ^959^

 Na RM, o TE é útil na avaliação de sintomas e determinação da aptidão cardiorrespiratória (principalmente se sintomas duvidosos) e, quando limitada, contribui para a indicação de intervenção cirúrgica. Os sintomas esforço-induzidos têm relação com a gravidade da doença. Nos assintomáticos com aptidão cardiorrespiratória preservada, permite adiar com segurança a correção valvar. ^98^ ^-^ ^100^

 A RM grave evolui com pressões elevadas no átrio esquerdo, HAP secundária, hipertrofia excêntrica com disfunção sistólica do VE. A disfunção do VE geralmente precede a RM moderada/grave com manifestações de dispneia e intolerância esforço-induzida. ^960^ ^,^ ^961^

 RM secundária à isquemia de músculo papilar geralmente está associada a DAC em CX ou CD. Na cardiomiopatia isquêmica ou dilatada, a remodelação do VE e a deformação da válvula mitral podem cursar com RM de pior prognóstico. ^962^ ^,^ ^963^

 Mesmo pacientes com sintomas leves podem descompensar em curto prazo. O início de sintomas indica que os mecanismos compensatórios do VE estão sobrecarregados. ^963^ ^,^ ^964^

Particularidades do TE na RM:

 – ECG de repouso na RM leve normalmente apresenta achatamento ou inversão da onda T nas derivações inferiores. Na moderada/grave, geralmente observa-se sobrecarga atrial esquerda. A SVE ocorre em ≈⅓ dos pacientes e a hipertrofia de ventrículo direito em ≈15%, sendo comum a presença de FA. ^965^

 – As alterações de repolarização esforço-induzidas não permitem a investigação de DAC principalmente se houver SVE e alterações do segmento ST no ECG basal. Mesmo com ECG basal normal, costuma ocorrer ISTE não associado a DAC, que não se relaciona à deterioração clínica/funcional. ^966^

 – Geralmente ocorre arritmia ventricular frequente e complexa (pareadas, TV não sustentada) no esforço e recuperação. ^967^

 – Atingir FCmax e/ou apresentar queda da FC no primeiro minuto da recuperação ≥29 bpm associaram-se a baixo risco de eventos cardíacos. ^966^

 – É comum observar resposta pressórica deprimida da PAS. Em casos mais graves, pode-se observar queda pressórica intraesforço devido à redução do débito cardíaco, indicando necessidade de interrupção imediata do esforço. ^931^

 – A aptidão cardiorrespiratória encontra-se reduzida em ≈20% dos pacientes assintomáticos com RM grave, estando associada com pior prognóstico. ^962^ ^,^ ^968^

#### 8.5.5. Prolapso da Válvula Mitral

 O prolapso da válvula mitral (PVM) é uma doença valvular, de predisposição genética, resultando em alterações mixomatosas dos folhetos da válvula mitral. Sua prevalência está em torno de 2% a 3%, sendo a evolução benigna na maioria dos casos. ^969^ ^,^ ^970^

 O PVM pode ser primário (ou “não sindrômico”) ou secundário (ou “sindrômico”) a distúrbios do tecido conjuntivo (síndrome de Marfan, síndrome de Loeys-Dietz, síndrome de Ehlers-Danlos, osteogênese imperfeita, pseudoxantoma elástico e síndrome de osteoartrite). Também pode ser observado na miocardiopatia hipertrófica. ^971^

 O PVM pode evoluir com progressiva regurgitação mitral por perda da aposição dos seus folhetos, sendo a principal causa de troca mitral cirúrgica por regurgitação em países desenvolvidos. A regurgitação mitral crônica frequentemente associa-se à hipertensão pulmonar com subsequente IC direita, aumento do risco de arritmias (incluindo FA), eventos tromboembólicos e endocardite infecciosa. ^95^ ^,^ ^969^ ^,^ ^971^

 Mulheres jovens com espessamento dos folhetos mitrais e/ou prolapso dos folhetos podem apresentar predisposição aumentada para arritmias complexas e morte súbita cardíaca arritmogênica (PVM arritmogênico), sendo essa a complicação mais temida. Geralmente o ECG basal apresenta ondas T negativas em parede inferior e EVs com padrão de BRD. ^970^ ^,^ ^972^

 PVM é um achado relativamente comum em atletas com evolução geralmente benigna. A presença de regurgitação mitral moderada/grave e arritmia ventricular são marcadores de atletas sob maior risco. ^973^ ^,^ ^974^

 Pacientes com PVM idiopático frequentemente queixam-se de palpitações ou taquicardias esforço-induzidas. Também podem apresentar dispneia, fadiga, tolerância reduzida aos esforços, pré-síncope, síncope e sequelas de AVC. 

 São marcadores de risco para morte súbita no PVM: sintomas pré-síncope/síncope; doença de Barlow (degeneração mixomatosa do tecido de colágeno); histórico familiar de morte súbita; regurgitação mitral moderada/grave; arritmias ventriculares. 

 O TE é útil no PVM para avaliação de sintomas, determinação da tolerância ao esforço, detecção de arritmias esforço-induzidos e liberação/prescrição exercícios físicos. 

Particularidades do TE no PVM:

 – A ausculta cardíaca basal pode ser normal ou evidenciar clique mesossistólico, sopro sistólico tardio ou mesotelessistólico (devido à insuficiência mitral). O sopro sistólico pode estar presente apenas na posição ortostática. ^975^

 – O ECG basal é normal na maioria dos pacientes. Podem ocorrer alterações inespecíficas das ondas T e segmento ST, especialmente nas derivações inferiores. Nos pacientes com regurgitação mitral crônica, pode ser observado padrão de SAE e SVE. ^976^

– Principais anormalidades durante o esforço:

 1) Tolerância ao esforço reduzida geralmente associada a padrão análogo à astenia neurocirculatória (caracterizada por queixa de palpitação, fraqueza, falta de ar, respiração difícil e outras reclamações subjetivas, mesmo em esforço físico leve). ^977^

 2) ISTE que mimetiza fortemente o padrão isquêmico da DAC obstrutiva, geralmente em pacientes com RM associada. O ISTE no PVM apresenta baixa sensibilidade e especificidade para DAC (resultado falso-positivo). ^978^ ^,^ ^979^

 3) As arritmias ventriculares esforço-induzidas com padrão de bloqueio de ramo direito ou complexas (EVs polimórficas, EVs pareadas e/ou TV não sustentada) são marcadores de risco na suspeita de PVM arritmogênico. ^974^ ^,^ ^980^

8.6. TE Pós-revascularização Miocárdica

 O TE no seguimento de pacientes após revascularização cirúrgica do miocárdio (CRVM) ou por intervenção coronária percutânea (ATCP) permite: ^6^ ^,^ ^13^ ^,^ ^27^ ^,^ ^134^

 – Esclarecer sintomas. ^45^ ^,^ ^981^

 – Determinar a aptidão cardiorrespiratória/tolerância aos esforços. 

– Avaliar a terapêutica farmacológica.

 – Estratificação de risco/prognóstico. ^982^

 – Avaliação da densidade e complexidade de arritmias. ^983^

 – Liberação para retorno a atividades laborais e perícia médica. 

 – Liberação e prescrição de exercícios físicos (inclusive reabilitação CV). ^17^ ^,^ ^29^

 – Avaliação de reestenose (na ATCP) ou estenose/oclusão de ponte (na CRVM) e/ou progressão da doença arterial coronariana. Nesses casos, o TE não deve ser indicado como forma de avaliação periódica/rotineira de pacientes assintomáticos sem indicações clínicas específicas. ^984^ ^-^ ^986^

 Pontos importantes a serem considerados previamente à realização do TE: ^6^ ^,^ ^13^ ^,^ ^293^

 – Persistência de sintomas mesmo com revascularização completa. 

– Se a revascularização foi parcial ou completa.

 – Ocorrência e/ou manutenção de disfunção ventricular/IC após a revascularização, particularmente após IAM. ^987^

– Necessidade de suspensão de medicamentos.

 – Nível de atividade física pré e pós-revascularização. 

 – Análise comparativa de achados hemodinâmicos e eletrocardiográficos em TE/TCPE pré-procedimento e/ou análise de seriada. 

#### 8.6.1. TE após Intervenção Coronária Percutânea

 De acordo com o tempo decorrido da ATCP, o TE apresenta indicações de reconhecido valor no acompanhamento clínico e evolução da DAC: ^6^ ^,^ ^13^ ^,^ ^988^

 – Entre 1-3 meses: investigação de novos sintomas; avaliação de carga isquêmica residual em ATCP incompleta; adequações na terapêutica medicamentosa; liberação/prescrição de exercícios (inclusive reabilitação); estratificação de risco; perícia médica e/ou avaliação pela medicina do trabalho. ^982^ ^,^ ^989^ ^,^ ^990^

 – 3-6 meses: investigação de manifestações isquêmicas devido a possível reestenose/oclusão de ponte; avaliação de pacientes com ATCP em ponte miocárdica; ajustes terapêuticos. ^50^ ^,^ ^991^ ^,^ ^992^

 – 6-24 meses: avaliação de sintomas; em pacientes com alto risco de eventos CV e progressão de DAC para otimização terapêutica (inclusive não farmacológica). ^50^ ^,^ ^984^ ^,^ ^993^

 – Após 24 meses: acompanhamento evolutivo da DAC e reestenoses, que deve ser seriada nos pacientes de alto risco. ^994^

Particularidades do TE após ATCP:

 – Deve ser limitado por sintomas, inclusive por ser característica fundamental na condução do paciente em programa de reabilitação (≥2 semanas após a alta hospitalar). ^29^

 – Ausência de angina nem sempre traduz revascularização completa. Angina esforço-induzida sugere isquemia residual e/ou reestenose. ^50^ ^,^ ^995^

 – DP <25.000 bpm.mmHg sugere mau resultado ou disfunção ventricular esquerda. A análise comparativa com os valores pré-procedimento é de fundamental importância. Fatores como o infarto intraprocedimento, progressão da DAC, desenvolvimento de colaterais, hipertensão arterial e medicamentos podem interferir no DP. ^996^

 – A normalização do segmento ST após a ATCP é o principal indicador de sucesso da revascularização. Na fase precoce após ATCP (1 a 6 meses), a recorrência de alterações do STs observadas em TE prévio não significa reestenose. A manutenção das alterações prévias, associada à diminuição do DP ou à reversão do TE para isquêmico, é sugestiva de mau prognóstico. Após 6 meses, na suspeita de DAC, um TE positivo pode refletir semioclusão/oclusão de *stent* , presença de lesões residuais, progressão/aparecimento de novas lesões ateroscleróticas e até áreas de miocárdio em adaptação pós-isquemia aguda. ^997^

 – Após 2 anos de ATCP, a interpretação de ISTE ainda pode ser limitada quando presente DAC multiarterial, revascularização incompleta, infarto prévio e alterações ECG preexistentes (SVE e BRE). Em geral, no TE de pacientes uniarteriais e multiarteriais, constatou-se sensibilidade de 46% e especificidade de 77% (lesões >50%) e sensibilidade de 50% e especificidade de 84% (lesões >70%). ^998^ ^,^ ^999^

 – Prolongamento da duração do QRS no pico do esforço tem se mostrado bom preditor de eventos coronários (em 23±9 meses). ^1000^

 – A maioria dos pacientes aumenta o tempo de tolerância ao esforço (TTE) após a intervenção, que nem sempre traduz revascularização completa ou suficiente. Em TE seriados, a redução do TTE costuma estar associada a reestenose e/ou progressão da DAC. ^996^ ^,^ ^1001^

####  8.6.2. TE após Cirurgia de Revascularização Miocárdica 

 O TE após CRMV permite a avaliação de sintomas, avaliação da aptidão cardiorrespiratória, ajustes terapêuticos e liberação/prescrição de atividades físicas (inclusive reabilitação CV). ^27^ ^,^ ^29^

 Existem duas fases para a indicação do TE após a CRVM: a precoce (<6 meses), com o objetivo de determinar os resultados imediatos da revascularização; e a tardia (>6 meses), para avaliação evolutiva do tratamento. Nos primeiros 5 anos após a CRVM, o TE não está indicado para avaliação seriada de pacientes assintomáticos, sem indicação específica. ^1002^ ^,^ ^1003^

 O TE após CRVM deve ser realizado somente após cerca de 3 meses, visando: ^115^ ^,^ ^1004^

 – Aguardar estabilização do esterno, fonte comum de dor/desconforto torácico e dificuldade respiratória (fator de confusão). 

 – Adaptações cardiocirculatórias e microcirculatórias como correção da taquicardia, volemia, hematócrito, excesso de catecolaminas e reserva coronária. 

 – Realização do TE antes desse prazo é segura, mas aumenta o risco de exames falsos-positivos e tem limitada aplicabilidade clínica. 

 No TE na CRVM completa e com sucesso: ^1005^

 – Pode ocorrer redução de ISTE e/ou normalização do STs. 

 – Em 30% dos pacientes, pode ocorrer ISTE não associado à obstrução/oclusão da ponte e/ou nova lesão coronariana. 

 – Enxertos ocluídos podem não causar ISTE compatível com isquemia. 

 – Na fase recente da CRVM (<6 meses), ISTE sugere trombose nas pontes. Na fase crônica, o ISTE associa-se à aterosclerose na ponte e/ou progressão da DAC. ^1006^

Nas oclusões de pontes, podem ocorrer:

 – Diminuição do tempo tolerância ao esforço, mesmo sem alterações eletrocardiográficas associadas. ^1007^

– Aumento do DP à custa da elevação da FC máxima.

 – DP <25.000 bpm.mmHg após CRVM é sugestivo de obstrução da ponte e/ou disfunção ventricular esquerda. 

Particularidade do TE no pós-CRVM:

 – A melhora dos sintomas de angina após CRVM decresce com o tempo, em decorrência de claudicação das pontes e/ou progressão da DAC. Cinco anos após CRVM completa, ≈30% dos pacientes retorna com quadro anginoso. ^1008^

 – Em acompanhamento de 435 pacientes pós-CRVM por TE seriado, na revascularização completa, houve melhora significativa da angina, TTE e ISTE no primeiro ano de evolução, com os resultados persistindo até o sexto ano. ^1009^

 – O TTE e o DP máximo foram mais elevados nos primeiros 3 anos de evolução de CRVM completa. ^1009^ ^,^ ^1010^

## Parte 3 – Teste Cardiopulmonar de Exercício

## 1. Introdução

 O teste cardiopulmonar de exercício (TCPE) é método de diagnóstico que proporciona análise conjunta das manifestações clínicas, hemodinâmicas, eletrocardiográficas, ventilatórias e concentrações de gases no ar expirado cujas indicações encontram-se na Seção 2.4. ^17^ ^,^ ^219^ ^,^ ^229^ ^,^ ^293^ ^,^ ^1011^

**Figura 18:**
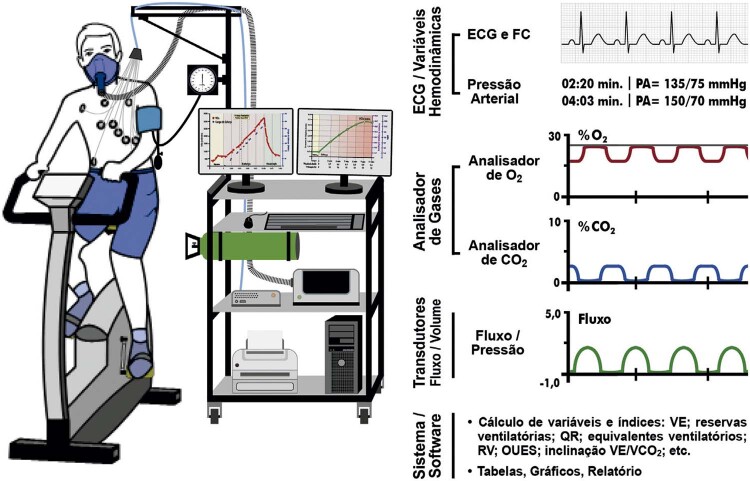
– Equipamentos no TCPE: transdutores de fluxo e volume de ar, analisadores de O 2 e CO 2 , ECG, estetoscópio/esfigmomanômetro e sistema/software específico.

 O TCPE incorpora em tempo real a mensuração do volume de ar ventilado (VE) pelos transdutores de fluxo e volume de ar (pneumotacógrafo, tubo de Pitot e turbina) e pelo analisador de gases (O _2_ e CO _2_ ), as frações expiradas de oxigênio (FEO _2_ ) e de dióxido de carbono (FECO _2_ ), permitindo a obtenção de múltiplas variáveis de interesse clínico ( Figura 18 ), sendo método de valor diagnóstico/prognóstico, excelência em termos de custo-efetividade e relevância na prática clínica e pesquisa. ^17^ ^,^ ^29^ ^,^ ^219^ ^,^ ^737^ ^,^ ^1012^ ^,^ ^1013^

## 2. Fisiologia do Exercício Aplicada ao TCPE

 Toda atividade física consome energia, requerendo a ressíntese ininterrupta da adenosina trifosfato (ATP), o que na musculatura esquelética ocorre por três vias metabólicas: 

 – Anaeróbio alático ou ATP-fosfocreatina (PCr): fornece energia imediatamente, porém por muito pouco tempo. Nas atividades muito intensas, o estoque celular de ATP-PCr se esgota em segundos, sendo esse o seu fator limitante. 

 – Anaeróbio lático ou glicólise anaeróbia: fornece energia a curto prazo, porém por pouco tempo. Nas atividades de grande intensidade com poucos minutos de duração, gera resíduo incremental de ácido lático que repercute em acidose metabólica limitante. 

 – Aeróbio ou oxidativo, exigindo consumo de O _2_ (VO _2_ ), fornece energia a longo prazo (fonte “ilimitada” de energia), mantendo esforço físico leve e moderado à custa da desintegração de macronutrientes. ^229^ ^,^ ^293^ ^,^ ^1011^

**Figura 19:**
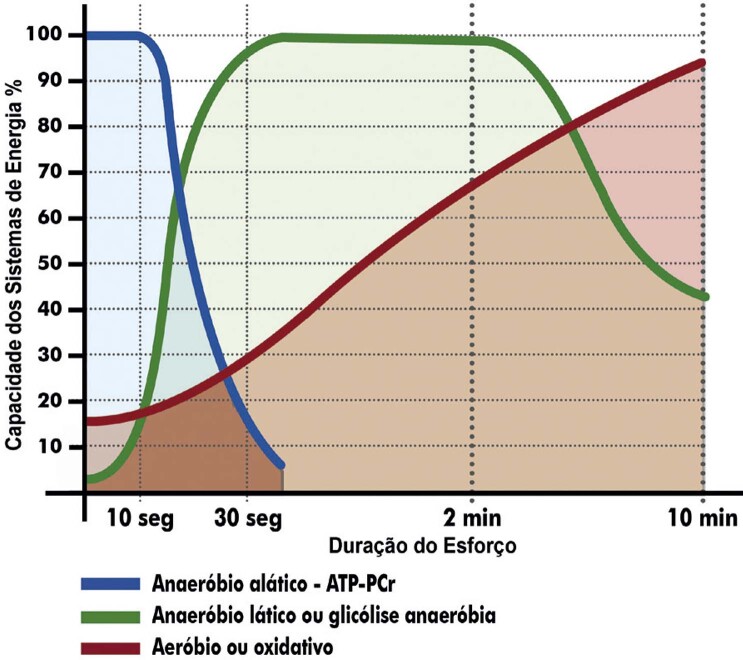
– Participação integrada e interdependente das vias metabólicas anaeróbicas e aeróbia no esforço físico.

 Essas três vias metabólicas funcionam integradas e interdependentes, e a recuperação das vias anaeróbicas depende do metabolismo aeróbico ( Figura 19 ). 

 Em relação ao TCPE, devemos considerar que, na transição do repouso para o esforço, há participação concomitante das 3 vias de metabolismo (aeróbico e anaeróbicos alático e lático). Somente após o período de ativação mitocondrial, a via aeróbica se torna predominante. 

**Figura 20:**
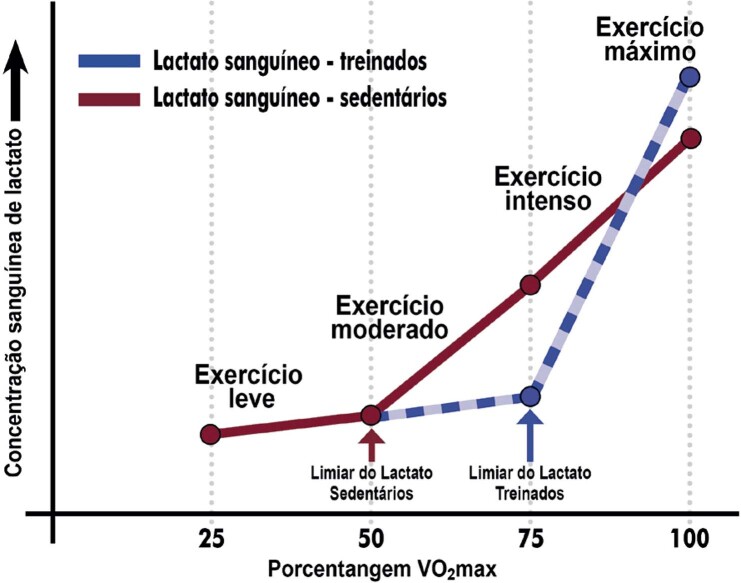
– Em sedentários e treinados, o limiar anaeróbio (LV1 – limiar de lactato) relacionado à intensidade do esforço físico (% do VO 2 máximo).

 A partir do primeiro limiar ventilatório (LV1), também denominado limiar anaeróbico ou limiar de lactato ( Figura 20 ), a via anaeróbica lática vai se tornando cada vez mais presente (atividades de moderada para elevada intensidade) com acidose metabólica compensada pela alcalose respiratória. A partir do segundo limiar ventilatório (LV2), também denominado ponto de compensação respiratória (PCR), ocorre acidose metabólica incrementalmente limitante devido à impossibilidade de compensação ventilatória/respiratória, culminando com exaustão física. ^219^ ^,^ ^229^ ^,^ ^293^ ^,^ ^1011^

 O limiar anaeróbio (LA) tem valor prognóstico em pacientes com IC, sendo relevante na avaliação do desempenho e prescrição de treinamento físico na reabilitação cardiovascular e no esporte. ^1014^ Com adaptação ao treinamento, o LA é deslocado para a direita, com maior tolerância ao esforço, aproximando-se do VO _2_ max. 

##  3. Ventilação Pulmonar, Gases no Ar Expirado e Variáveis Derivadas 

### 3.1. Ventilação Pulmonar

#### 3.1.1. Espirometria Basal

 A espirometria basal mede o volume e os fluxos derivados de manobras ventilatórias inspiratórias e expiratórias máximas. 

 Os parâmetros básicos para a interpretação apropriada dos testes de função pulmonar visando avaliar distúrbios ventilatórios (obstrutivo, restritivo e misto) são: capacidade vital; volume expiratório forçado no primeiro segundo (VEF1); relação VEF1/CVF; capacidade pulmonar total (CPT); e volume residual (VR), cujos valores de normalidade para a população brasileira branca (caucasiana) e negra são mostrados na Tabela 42 . ^1015^ ^-^ ^1017^

**Tabela 42:** – Valores de referência de parâmetros básicos da espirometria para a população brasileira adulta, branca e negra

	Raça Branca		Raça Negra	
		
**Variáveis**	**Homens**	**Mulheres**	**Homens**	**Mulheres**
CVF (L)	4,64±0,77	4,42±0,78	3,14±0,65	3,10±0,52
VEF1 (L)	3,77±0,67	2,56±0,57	3,55±0,69	2,55±0,48
VEF1/CVF (%)	81,0±5	81,0±5	80,3±5,4	82,0±5,4
FEF25-75 (L/s)	3,87±1,20	2,70±0,94	3,54±1,17	2,77±0,93
FEF50 (L/s)	4,82±1,44	3,40±1,14	4,39±1,36	3,54±1,06
FEF75 (L/s)	1,58±0,64	1,07±0,52	1,43±0,63	1,11±0,52
PFE (L/s)	11,1±1,75	7,14±1,28	9,77±2,07	6,73±1,28
* Valor ± desvio padrão; CVF: capacidade vital forçada; VEF1: volume expiratório forçado no primeiro segundo; FEF25-75: fluxo expiratório forçado entre 25% e 75%; FEF50: fluxo expiratório forçado de 50%; FEF75: fluxo expiratório; PFE: pico de fluxo expiratório. Adaptada de Pereira CAC et al. ^*1015*^ “Novos valores de referência para espirometria forçada em brasileiros adultos de raça branca.” e de Prata TA et al. ^*1016*^ “Valores de referência para espirometria forçada em adultos negros no Brasil. *				

Variáveis mais utilizadas na prática clínica:

 – Capacidade vital (CV): corresponde ao maior volume de ar mobilizado em uma expiração máxima, podendo ser obtida através de manobras forçadas (CVF) ou lentas (menos utilizadas). A CVF corresponde ao volume obtido em uma única inspiração máxima seguida de uma expiração também máxima. No esforço tanto nos sedentários quanto nos ativos, apesar dos grandes aumentos da VE, o VC somente excepcionalmente ultrapassa 60% da CVF. A CVF varia com idade, IMC e gênero, situando-se nos homens entre 4-5 L e nas mulheres entre 3-4 L. 

 – VEF1: volume de ar exalado no primeiro segundo durante a manobra de CVF. É uma das variáveis mais úteis clinicamente, auxiliando no diagnóstico de distúrbio ventilatório obstrutivo. 

 – Fluxo expiratório forçado intermediário (FEF25-75): representa o fluxo expiratório forçado médio obtido durante a manobra de CVF, na faixa intermediária entre 25% e 75% da CVF. 

 – Pico de fluxo expiratório (PFE): corresponde ao fluxo máximo de ar durante a manobra de CVF. 

 – Curva fluxo-volume: apresentação gráfica do fluxo e volume gerados durante a manobra de CVF, sendo importante para o diagnóstico de determinadas afecções respiratórias, permitindo a identificação visual de padrões obstrutivos, restritivos, amputações de fluxos inspiratórios ou expiratórios e avaliação das respostas ao broncodilatador. 

#### 3.1.2. Ergoespirometria

 A ventilação pulmonar (VE) em um minuto (L/min) corresponde ao volume total de ar que ventila o trato respiratório, incluindo o espaço morto (VD). É calculada pela fórmula: 


VE=VC×FR

VE: ventilação pulmonar (L/min)

FR: frequência respiratória (incursões respiratórias/min)

VC: volume corrente (L/min)

#### 3.1.3. Reserva Ventilatória

**Figura 21:**
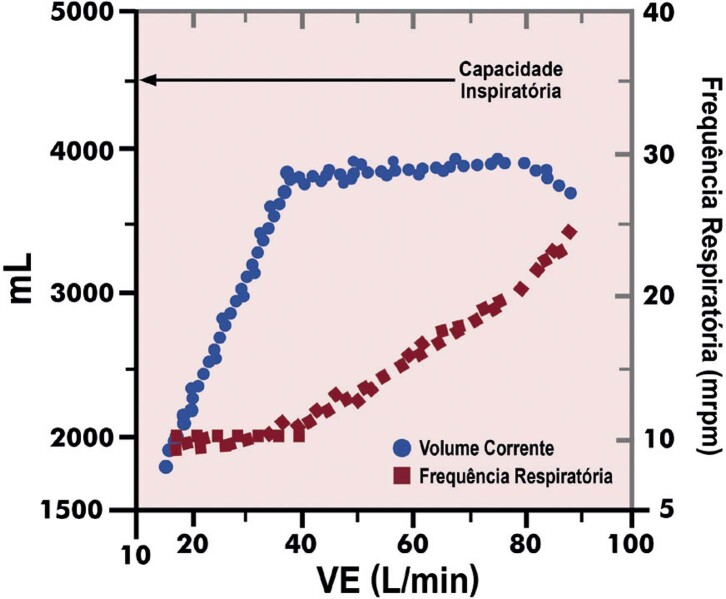
– Aumento da ventilação pulmonar (VE) – inicialmente dependente quase exclusivamente do volume corrente (VC) que logo atinge um platô, ficando em seguida dependente apenas do incremento da frequência respiratória (FR).

 Nos adultos jovens e saudáveis, em repouso, a FR costuma ser ≈12 incursões/min e o VC ≈0,5 L/min, resultando em VE = 6 L/min (0,5 L/min × 12 = 6 L/min). Durante esforço físico extenuante, a VE costuma aumentar 17-20 vezes, às custas de FR de 35-45/min. Excepcionalmente, a FR excede 50/min e o VC supera 2 L/min ( Figura 21 ). 

 A reserva ventilatória (RV) corresponde à relação entre a ventilação máxima de exercício (VEmax) e a ventilação voluntária máxima (VVM) em repouso (ambas em L/min; RV = [VVM-VEmax/VVM] x100), sendo o normal de 20% a 40% que corresponde em média a 3.000 mL nos homens e 2.100 mL nas mulheres. A VVM é avaliada por meio de uma respiração rápida e profunda por 15 segundos, extrapolada para o que seria obtido se a manobra persistisse por 1 minuto, oscilando entre 35 e 40 vezes o VEF1. A medida da RV contribui para o diagnóstico diferencial de dispneia. ^1011^ ^,^ ^1017^ ^,^ ^1018^

Equações de predição:


VVM=VEF1×(

$$RV=\frac{\text{ VVM-VEmax }}{\text{ VVM }}\times 100$$

VVM: ventilação voluntária máxima

VEF1: volume expiratório forçado em um segundo

RV: reserva ventilatória

VEmax: ventilação máxima de exercício

 O aumento do VC no esforço ocorre pela utilização adicional dos volumes de reserva inspiratória (VRInsp) e expiratória (VRExp), podendo atingir, respectivamente, 75% a 85% da CPT e 40% da CPT. Esses volumes são determinantes no estabelecimento da VEmax que, em condições normais, fica abaixo de 70% da VVM (resultando em RV entre 20% e 40%). Ressalta-se que em exercícios extenuantes, particularmente em atletas, pode ser utilizado um maior percentual da RV. ^219^ ^,^ ^229^ ^,^ ^293^ ^,^ ^1011^

## 3.2. Consumo de Oxigênio

**Figura 22:**
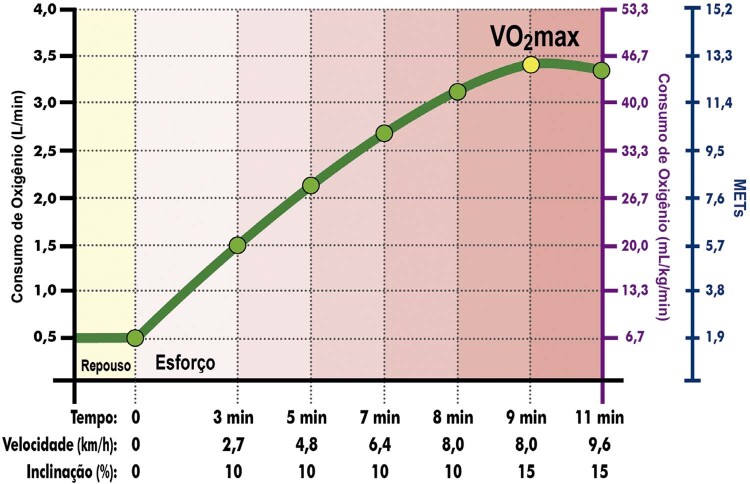
– Em TCPE máximo, consumo de oxigênio (VO 2 ) em valores absolutos (em L/min), relativos (em mL/kg/min) e em equivalentes metabólicos (METs). A característica da curva de VO 2 , com platô no pico de esforço indica que paciente atingiu o VO 2 máximo.

 O consumo de oxigênio (VO _2_ ) corresponde à diferença entre as concentrações de O _2_ do ar inspirado e do ar expirado, sendo a quantidade de O _2_ consumido na atividade metabólica ( Figura 22 ). Seus valores podem ser expressos de forma absoluta (L/min) ou, preferencialmente, em valores relativos ao peso corporal mais frequentemente expresso em mL/kg/min (também aceitável mL.kg ^-^ ^1^ .min ^-^ ^1^ ), podendo ser convertido em equivalente metabólico (1 MET = 3,5 mL/kg/min de VO _2_ ). 

**Figura 23:**
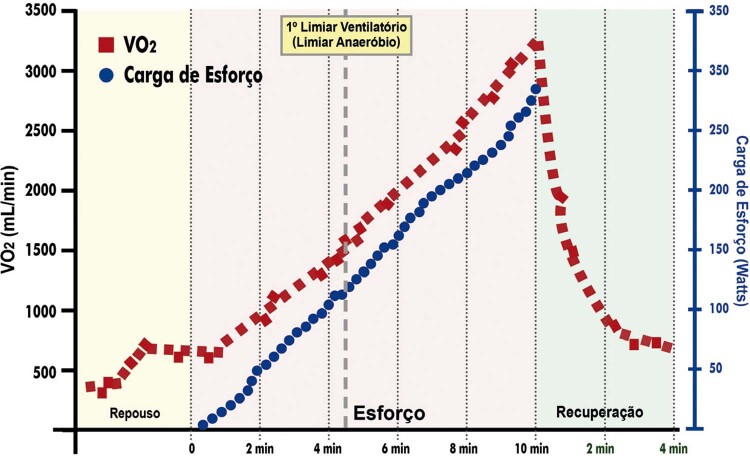
– TCPE, o consumo de oxigênio (VO 2 ) apresentado em valores absolutos* e a carga de trabalho em Watts. VO 2 pico: curva de VO 2 sem platô no pico de esforço indicando que o paciente não atingiu o VO 2 máximo. Figura ilustrativa: na prática, normalmente apresenta-se o valor de VO 2 em valores relativos ao peso corporal.

 O maior VO _2_ obtido no TCPE pode ser considerado: 

 – VO _2 _ máximo (VO _2_ max) quando, apesar do aumento da carga de esforço, se estabelece um platô de VO _2_ , sem elevação ou com apenas um discreto aumento (<50 mL/min ou 2,1 mL/kg/min) – vide Figura 22 . 

 – VO _2_ pico quando o maior valor obtido no final de um esforço exaustivo ocorre na ausência de um platô na curva de VO _2_ ( Figura 23 ). ^219^ ^,^ ^229^ ^,^ ^293^ ^,^ ^1011^

 O VO _2_ max é considerado o padrão-ouro na determinação da aptidão cardiorrespiratória (ACR). ^1012^

3.3. Produção de Gás Carbônico

 A produção de gás carbônico (VCO _2_ ), no TCPE determinada a partir da FECO _2_ , geralmente é expressa em L/min, sendo raramente utilizado isoladamente. Entretanto, as variáveis derivadas de sua medida, como o quociente respiratório (QR) e o equivalente ventilatório de CO _2 _ (VE/VCO _2_ ), têm grande utilidade clínica. ^219^ ^,^ ^229^ ^,^ ^293^ ^,^ ^1011^

## 3.4. Limiares Ventilatórios

 Os limiares ventilatórios são pontos de transição metabólica, observados nas curvas de VE, VO _2_ , VCO _2_ e nas suas variáveis derivadas. Podem ser expressos indiretamente em relação ao metabolismo (p. ex., VO _2_ em mL/min ou L/min ou em % do VO _2_ max) ou à demanda cardiovascular (p. ex., VO _2_ em mL/bpm) – vide Figuras 20 e 23. ^219^ ^,^ ^229^ ^,^ ^293^ ^,^ ^1011^

 A identificação dos limiares em pacientes com DCV (como IC e DAC) tem relevante valor prognóstico, contribui para ajustes na terapêutica farmacológica e otimiza a prescrição de exercícios na reabilitação cardiovascular e treinamento físico de indivíduos aparentemente saudáveis, particularmente atletas competitivos de atividades predominantemente aeróbicas. ^17^ ^,^ ^219^ ^,^ ^229^ ^,^ ^293^ ^,^ ^1011^ ^,^ ^1014^ ^,^ ^1016^

### 3.4.1. Primeiro Limiar Ventilatório

**Figura 24:**
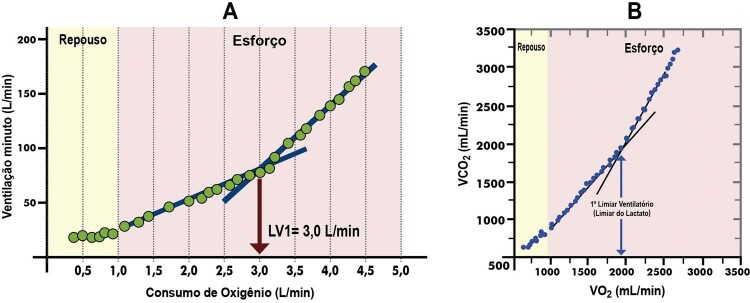
– A e B . Limiar Anaeróbio (LV1) determinado a partir das relações VE/VO 2 (A) e VCO 2 /VO 2 (B).

 O 1º limiar ventilatório (LV1), também denominado limiar anaeróbio lático (LA) ou limiar de lactato (LL), marca a aceleração da taxa de acúmulo sustentado de lactato na corrente sanguínea, identificando a transição para uma etapa de crescente e incremental participação do metabolismo anaeróbio lático, mas com acidose metabólica ainda compensada pela alcalose respiratória até atingir o 2º limiar ventilatório (LV2). A sua medida direta se faz por meio da dosagem do lactato sanguíneo (expresso em mmol/L ou mEq/L) e a indireta a partir das medidas do TCPE, podendo ser observado no primeiro ponto de dissociação das curvas VE/VO _2_ ( Figura 24A ) e VCO _2_ /VO _2_ ( Figura 24B ). ^219^ ^,^ ^229^ ^,^ ^293^ ^,^ ^1011^

### 3.4.2. Segundo Limiar Ventilatório

**Figura 25:**
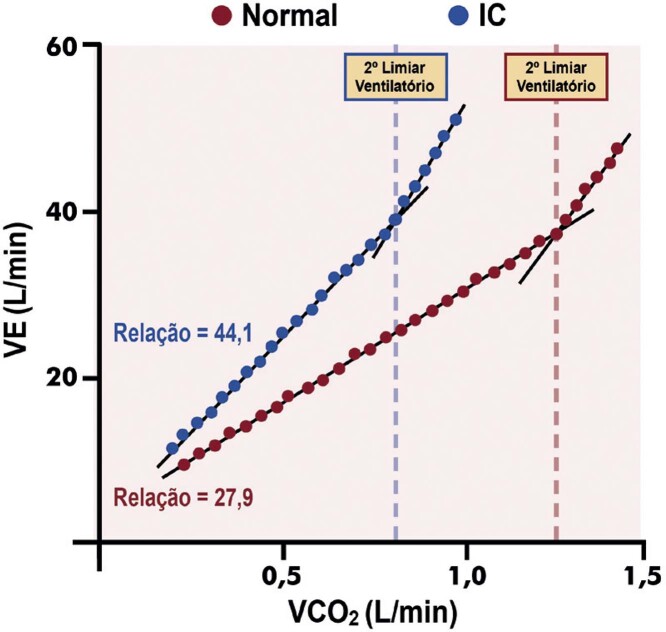
– Segundo limiar (LV2), a partir do equivalente ventilatório de CO 2 (VE/VCO 2 ) que se encontra anormal em indivíduo com insuficiência cardíaca (IC) e normal em saudável. A relação VE/VCO 2 é preditor de mortalidade e hospitalização na IC, indicando pior prognóstico se relação ≥34.

 O LV2, também denominado ponto de compensação respiratória (PCR) ou OBLA (do inglês, *onset of blood lactate accumulation* ), corresponde ao ponto em que ocorre uma segunda inflexão nas curvas de VE e CO _2_ em relação à curva de VO _2_ , reflexo da acidose metabólica descompensada. Corresponde à transição para o esforço de muito elevada até a máxima intensidade, com grande e incremental acidose metabólica descompensada, evoluindo rapidamente para exaustão física. A partir do LV2 ocorre também incremento desproporcional da VE em relação ao VCO _2, _ ou seja, dissociação das curvas da VE e da VCO _2_ , com aumento da relação VE/VCO _2_ . Nesse ponto, nos pacientes com IC, a relação VE/VCO _2_ ≥34 indica pior prognóstico ( Figura 25 ). ^219^ ^,^ ^229^ ^,^ ^293^ ^,^ ^1011^

## 3.5. Quociente Respiratório

 O quociente respiratório (QR) ou razão de trocas respiratórias (R) corresponde à razão entre VCO _2_ e o VO _2_ , permitindo identificar a intensidade do exercício e o macronutriente utilizado para gerar energia: 

 – Nas cargas iniciais do TCPE, encontra-se em torno de 0,72 (utilização quase exclusivamente de gordura). 

 – No LV1 costuma ser ≈0,82 (utilização ≈40% de lipídios e ≈60% de carboidratos). 

 – No LV2, está em torno de 1,00 (utilização quase exclusiva de carboidratos). 

 Em intensidades mais elevadas e próximo ao pico do esforço, o VCO _2_ supera o VO _2_ , tornando o QR incrementalmente superior a 1,00. O QR ≥1,10 é considerado sinal de quase exaustão ou exaustão, permitindo considerar o exame como sendo de esforço máximo. ^219^ ^,^ ^229^ ^,^ ^293^ ^,^ ^1011^ ^,^ ^1012^

##  3.6. Equivalentes Ventilatórios de Oxigênio e Gás Carbônico 

 Os equivalentes ventilatórios de O _2_ (VE/VO _2_ ) e de CO _2_ (VE/VCO _2_ ) indicam, respectivamente, a VE necessária para consumir 1 L/min de O _2_ e produzir/eliminar 1 L/min de CO _2_ . Durante o esforço progressivo, a razão VE/VO _2_ diminui até o LV1, a partir do qual progressivamente aumenta, com inflexões positivas nas curvas no LV1 e LV2. A razão VE/VCO _2_ diminui até o LV2, aumentando em seguida. 

**Figura 26:**
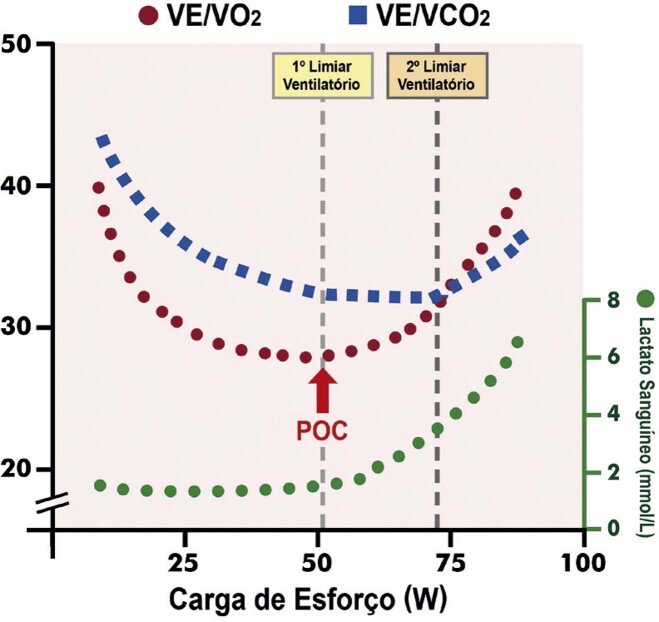
– Equivalentes ventilatórios de oxigênio (VE/VO 2 ) e de dióxido de carbono (VE/VCO 2 ) relacionados à carga de trabalho e curva de lactato sanguíneo, com visualização dos pontos correspondentes aos limiares ventilatórios (LV1 e LV2) e ponto ótimo cardiorrespiratório (POC).

 São parâmetros relevantes para avaliação da eficiência cardiorrespiratória, auxiliam na identificação dos limiares e contribuem para os diagnósticos, prognósticos, ajustes no tratamento farmacológico e prescrição de exercícios de várias situações clínicas, principalmente cardiopatia isquêmica e IC ( Figura 26 ). ^219^ ^,^ ^229^ ^,^ ^293^ ^,^ ^1011^

 A curva VE/VCO _2_ é considerada preditor de mortalidade e/ou de hospitalização, principalmente em pacientes com IC cardíaca e DPOC. Quanto maior o seu valor, pior o prognóstico ( Figura 25 ). 

##  3.7. Pressões Parciais Expiratórias do Oxigênio e Dióxido de Carbono 

 A pressão parcial expiratória final de oxigênio (PETO _2_ ) é obtida a partir da medida da FEO _2 _ no ar expirado pelo analisador de gases, sendo habitualmente referida em mmHg (baseada na Lei de Dalton em que a pressão total de uma mistura gasosa é igual à soma das pressões parciais de seus gases componentes). 

**Figura 27:**
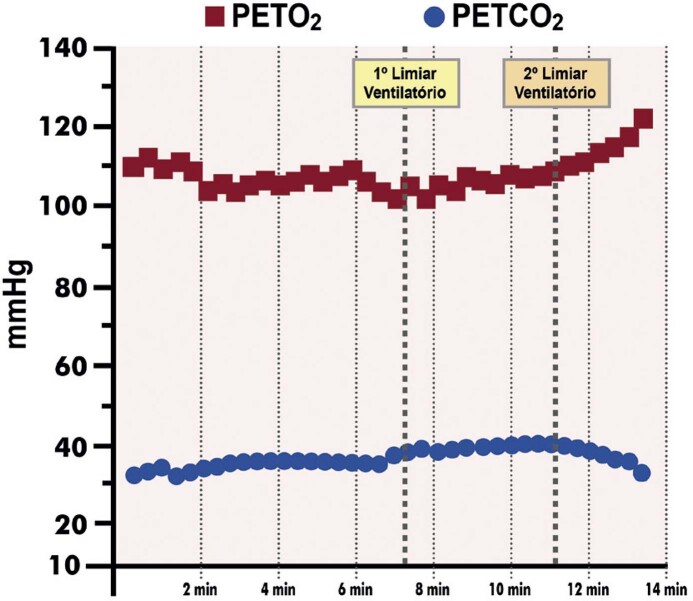
– Pressões parciais expiratórias do oxigênio (PETO 2 ) e dióxido de carbono (PETCO 2 ), com determinação dos limiares ventilatórios (LV1 e LV2).

 Normalmente, em repouso e no nível do mar, a PETO _2_ é de ≈100 mmHg. No início do esforço, apresenta diminuição transitória (aumento desproporcional de VO _2_ em relação à VE), para em seguida aumentar 10 a 30 mmHg até o esforço máximo ( Figura 27 ). 

 Foi demonstrado que a PETO _2_ , tanto em repouso quanto durante o esforço, correlacionou-se com 4 índices do TCPE comumente anormais em pacientes com disfunção do VE: VO _2_ pico; limiar anaeróbico (LV1); relação delta VO _2_ com delta carga de trabalho (ΔVO _2_ /ΔWR); inclinação aumentada da curva VE/VCO _2_ (marcador de incompatibilidade da ventilação com perfusão). 

 A PETO _2_ é maior nos pacientes com função cardiopulmonar prejudicada, sendo o LV1 o estado metabólico que apresenta melhores correlações. ^1019^

 A pressão parcial expiratória final de dióxido de carbono (PETCO _2_ ) é obtida a partir da medida da FECO _2_ , sendo habitualmente referida em mmHg. Reflete a pressão parcial de dióxido de carbono alveolar e arterial (PaCO _2_ ). Normalmente, em repouso e ao nível do mar, a pressão de CO _2 _ (PCO _2_ ) alveolar é de ≈40 mmHg, elevando-se 3-8 mmHg no esforço. Atinge o seu valor máximo no LV2 e, em seguida, diminui até o esforço máximo. A PETCO _2_ , assim como a PETO _2_ , correlaciona-se com: VO _2_ pico; LV1; ∆VO _2_ /∆WR; inclinação aumentada da VE/VCO _2_ ( Figura 27 ). ^1019^

 A PETCO _2_ medida no LV1 (momento de maior estabilidade metabólica) correlaciona-se com o débito cardíaco (DC) e em pacientes com IC crônica, refletindo a gravidade da doença. A sua determinação pode ser comprometida na hiperventilação aguda, no aumento do espaço morto (devido enfisema e outras doenças pulmonares) e na associação de FR elevada com ventilação superficial. ^1019^ ^,^ ^1020^

 No *shunt* direito-esquerdo esforço-induzido, ocorre aumento abrupto e sustentado da PETCO _2_ , com diminuição concomitante da PETO _2_ , coincidentes com aumento da VCO _2_ /VO _2_ e declínio da saturação. ^1021^ ^,^ ^1022^

## 3.8. Pulso de Oxigênio

**Figura 28:**
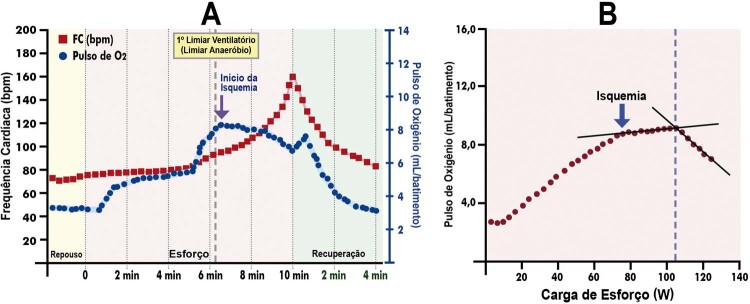
– A e B . Apresentação de pulsos de O 2 anormais devido à isquemia miocárdica: A = queda; B = platô.

 O pulso de oxigênio (PuO _2_ ) é obtido pela divisão do VO _2_ pela FC (VO _2_ /FC) em mL/kg/min/bpm, reflete a quantidade de O _2_ que é transportada a cada sístole cardíaca e tem relação direta com o volume sistólico, permitindo avaliar a função do VE. Na isquemia miocárdica, a disfunção do VE pode ser constatada pela resposta do PuO _2_ em platô e/ou queda ( Figura 28 A e B). ^219^ ^,^ ^229^ ^,^ ^1013^ ^,^ ^1023^

 No estudo ORBITA, dos parâmetros do TCPE, apenas o platô de PuO _2_ detectou objetivamente a gravidade da isquemia miocárdica diagnosticada no ecocardiograma sob estresse com dobutamina. ^1013^

##  3.9. Relação Delta VO 2 e Delta Carga de Trabalho (ΔVO 2 /ΔWR) 

 A relação ΔVO _2_ /ΔWR fornece uma quantificação fisiológica da taxa de trabalho, FC e VO _2_ em que uma isquemia miocárdica se desenvolve, permitindo seu diagnóstico, quantificação e eventual reversibilidade com o tratamento. É mais facilmente determinada em cicloergômetro com protocolo de rampa, sendo expressa em mL/min/W. ^219^ ^,^ ^229^ ^,^ ^293^ ^,^ ^1011^ ^,^ ^1024^

**Figura 29:**
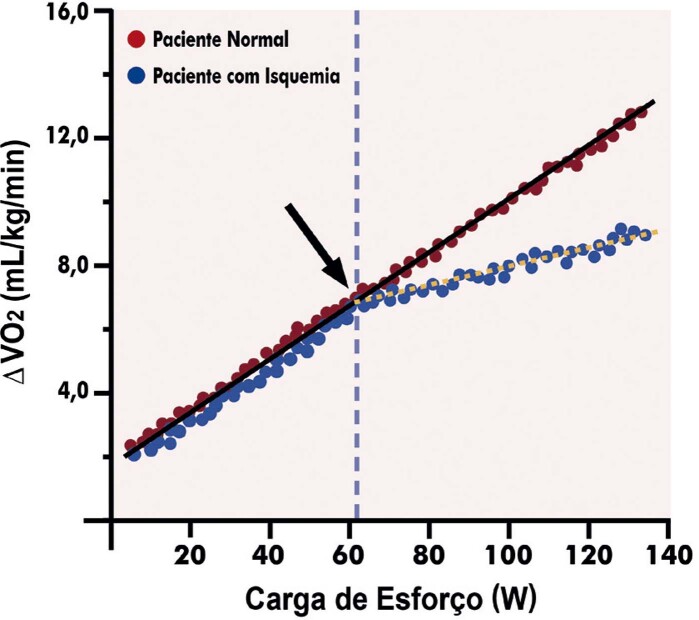
– Relação ∆VO 2 /∆WR característica da isquemia miocárdica.

 Em adultos saudáveis, a relação é linear do início ao pico do esforço, com valor ≈10 mL/min/W. ^1023^ ^,^ ^1024^ Na isquemia esforço-induzida, ocorre disfunção do VE com achatamento da curva ∆VO _2_ /∆WR a partir do limiar isquêmico ( Figura 29 ). ^1023^ ^,^ ^1024^

## 3.10. Ponto Ótimo Cardiorrespiratório

 O ponto ótimo cardiorrespiratório (POC) corresponde ao valor mínimo da curva VE/VO _2_ ( Figura 26 ), sendo uma variável submáxima de fácil utilização. Reflete a eficiência cardiorrespiratória, tem valor preditor de mortalidade cardiovascular e por todas as causas, isoladamente ou associada ao VO _2_ max. ^1025^ ^,^ ^1026^

##  3.11. Inclinação da Eficiência da Captação do Oxigênio (OUES) 

 A inclinação da eficiência da captação do oxigênio (do inglês, *oxygen uptake efficiency slope* [OUES]) é variável de grande reprodutibilidade, derivada inclusive de valores submáximos de VO _2_ . É uma relação não linear da resposta ventilatória ao esforço, gerada por regressão logarítmica entre o VO _2_ e a VE. De forma simples, pode-se dizer que o OUES corresponde ao aumento absoluto do VO _2_ associado a um aumento de 10 vezes da VE. ^1014^ ^,^ ^1027^ ^,^ ^1028^


$$\text{ Equação para obter OUES: }{VO}_{2}=a\log10VE+b$$

a = valor de referência de OUES calculado pelas fórmulas apresentadas no texto

b = valor da intercepção na curva

VE = ventilação pulmonar

 Em 2012, Sun et al. ^1028^ apresentaram fórmulas de previsão do OUES: 

 – Para homens: 1,178 – (idade × 0,032) + (0,023 × altura [cm]) + (0,008 × peso [kg]) 

 – Para mulheres: 0,61 – (idade × 0,032) + (0,023 × altura [cm]) + (0,008 × peso [kg]) 

**Figura 30:**
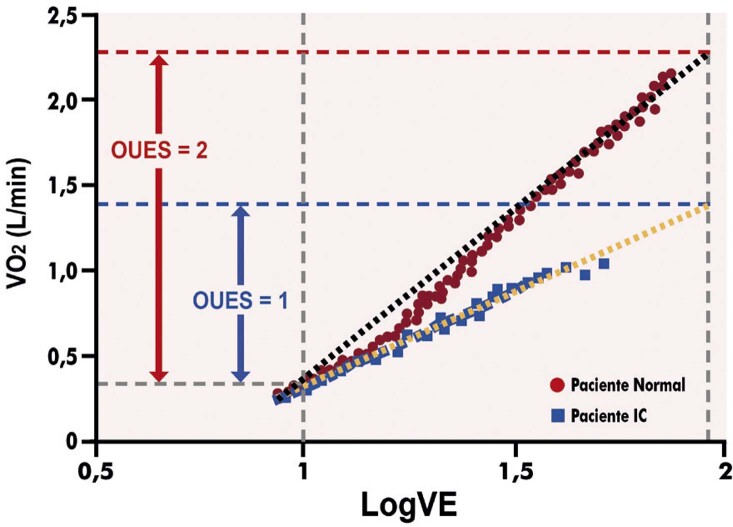
– Eficiência de captação de oxigênio (OUES) em indivíduos com boa eficiência (normal) e baixa eficiência (na insuficiência cardíaca).

 Quanto mais inclinada for a reta ajustada do VO _2_ , maior é o valor do OUES e, portanto, maior a eficiência no consumo de O _2_ . Pacientes com IC grave apresentam menor eficiência ventilatória com baixos valores do OUES ( Figura 30 ). ^1014^ ^,^ ^1027^ ^-^ ^1029^

 O OUES tem valor prognóstico na IC, inclusive isoladamente, quanto ao risco de eventos. ^1014^ Outros estudos também documentaram o valor prognóstico do OUES comparado a outras variáveis do TCPE. ^1027^ ^,^ ^1029^

## 3.12. Ventilação Oscilatória ao Esforço

 A ventilação oscilatória ao esforço (do inglês, *exercise oscilatory ventilation* [EOV]) é um fenômeno anormal, reprodutível, facilmente reconhecível no TCPE submáximo. É caracterizado como uma flutuação cíclica da VE (padrão ventilatório oscilatório >15% do valor médio de VE em repouso) e da cinética de gases expirados, com duração >60% do tempo de esforço. ^229^ ^,^ ^1030^ ^-^ ^1034^

**Figura 31:**
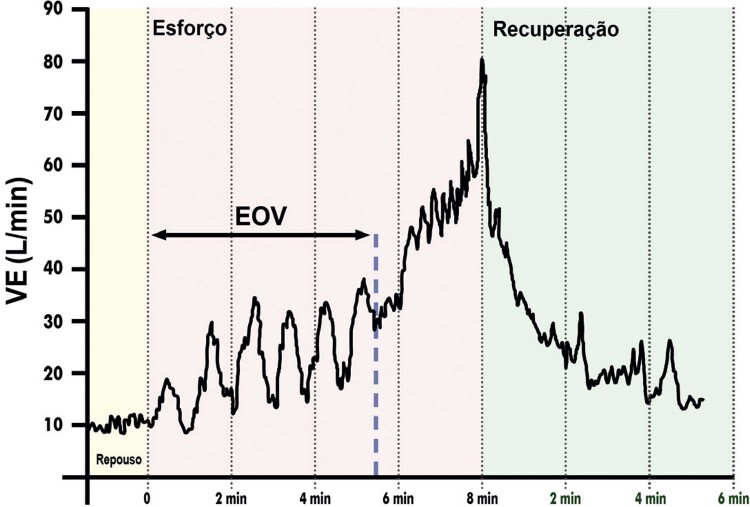
– Ilustração da ventilação oscilatória em TCPE de paciente com grave insuficiência cardíaca (ICFEr).

 A EOV é considerada um marcador de gravidade e pior prognóstico na IC, principalmente quando ocorre precocemente e o ciclo dura mais que 1 minuto ( Figura 31 ). ^229^ ^,^ ^1026^ ^,^ ^1035^

## 3.13. Tempo de Recuperação do Consumo de Oxigênio

**Figura 32:**
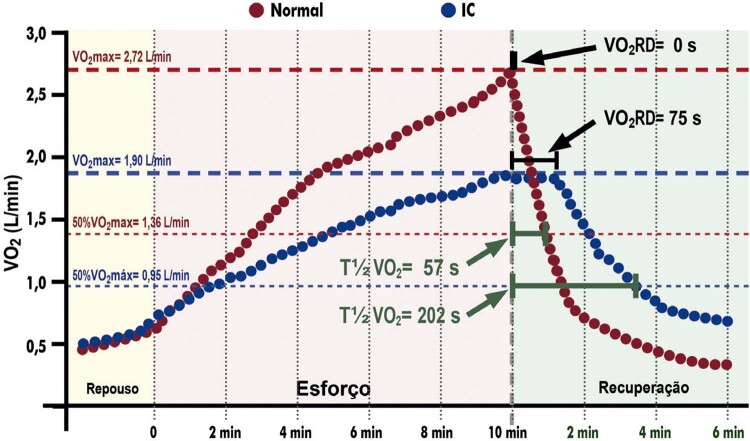
– Em indivíduo saudável e paciente com IC, as cinéticas de oxigênio no esforço e recuperação, com seus respectivos T½.

 O tempo de recuperação do consumo de oxigênio (do inglês, *recovery delay* [RD]), que corresponde à queda do VO _2_ na recuperação, tem padrão facilmente reconhecível, contribuindo para prognóstico, ajustes no tratamento farmacológico e prescrição de exercícios na reabilitação cardiovascular e no treinamento esportivo. O T½ corresponde ao tempo necessário para queda de 50% do VO _2_ pico, diminuindo com o treinamento físico bem conduzido, enquanto seu aumento associa-se a pior prognóstico nos pacientes com IC ( Figura 32 ). ^1011^ ^,^ ^1036^ ^-^ ^1038^

##  3.14. Potência Circulatória e Potência Ventilatória 

 A potência circulatória (PC), é o produto da pressão arterial sistólica pico (PASpico) pelo VO _2_ max ou pico. A potência ventilatória (PV) é o produto da PASpico dividida pelo VE/VCO _2_ . Ambas têm valor prognóstico na ICFEr, independentemente da potência circulatória. A avaliação conjunta melhora a estratificação de risco para desfechos maiores (morte, dispositivo de assistência ventricular, transplante cardíaco). ^1039^

## 3.15. Valores de Referência de Variáveis do TCPE

**Figura 33:**
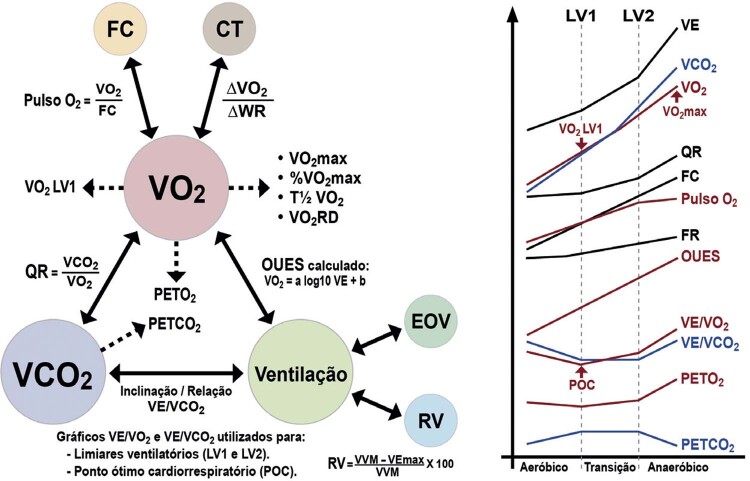
– Medidas e variáveis do TCPE e suas principais inter-relações. O sistema/software integra informações e disponibiliza múltiplas variáveis de interesse clínico a partir de medidas: ventilatórias (via transdutores de fluxo e volume do ar inspirado); metabólicas (via análise de gases no ar expirado – VE, FEO 2 e FECO 2 ); hemodinâmicas (ECG, FC, PA). Dessas medidas, são geradas múltiplas variáveis que podem ser analisadas por meio de gráficos. VO 2 : consumo de oxigênio; VCO 2 : produção de CO 2 ; VE: ventilação pulmonar; EOV: ventilação oscilatória ao esforço; RV: reserva ventilatória; VVM: ventilação voluntária máxima; LV1: 1 º limiar ventilatório; LV2: 2 º limiar ventilatório; PETO 2 : pressão parcial expiratória final de oxigênio; PETCO 2 : pressão parcial expiratória final de dióxido de carbono; VO 2 max: consumo máximo de oxigênio; %VO 2 max: porcentagem do consumo máximo de oxigênio; FC: frequência cardíaca; FR: frequência respiratória; CT: carga de trabalho em Watts; VO 2 RD: retardo da recuperação do VO 2 ; OUES: inclinação da eficiência da captação do oxigênio; T½: tempo necessário para queda de 50% do VO 2 pico na recuperação; ΔVO 2 /ΔWR: relação delta VO 2 e delta carga de trabalho; POC: ponto ótimo cardiorrespiratório; Pulso O 2 : pulso de oxigênio; QR: quociente respiratório.

 Os resultados obtidos em relação às diversas variáveis do TCPE ( Figura 33 ), sempre que possível, devem ser apresentados relacionando-os aos seus respectivos valores de referência (valores previstos), essencial para as devidas interpretações e conclusões. Sugere-se adotar os valores de referência apresentados nas Tabelas 43 e 44. 

## 4. Equipamentos e Metodologia

### 4.1. Ergômetros

 Os ergômetros mais utilizados no TCPE são a esteira ergométrica e a bicicleta ergométrica. 

 Na população em geral, a esteira tem algumas vantagens em relação à bicicleta, tais como: atividade física mais familiar, uso de maior massa muscular, trabalho contra a gravidade causando maior estresse do sistema cardiorrespiratório e, consequentemente, maior VO _2_ máximo/pico (≈5-15% maior). Em relação à bicicleta, uma limitação é a dificuldade/imprecisão na determinação da carga de trabalho em Watts. ^1040^

 A bicicleta ergométrica pode ser de frenagem mecânica ou eletromagnética, que é a preferencial por permitir incremento automático com pequenas mudanças na cadência de pedaladas (40-70 rpm). Apresenta menor risco de quedas e menos artefatos favorecendo os registros do ECG e verificação da PA. ^1040^

 Na avaliação de atletas de alto desempenho, o ideal é o uso do ergômetro que seja mais semelhante à atividade esportiva, ou seja, bicicleta ergométrica para os ciclistas e esteira para os corredores. Considerar adicionalmente a experiência da instituição, a familiaridade do atleta e os equipamentos disponíveis (p. ex., remo, tanque e esqui ergômetros). ^1011^ ^,^ ^1040^

### 4.2. Transdutores de Fluxo ou Volume de Ar

 São recomendados equipamentos homologados que permitam a plena utilização dos principais parâmetros propostos por esta diretriz e sociedades científicas a exemplo das Sociedades Brasileiras de Cardiologia e Pneumologia, a *European Respiratory Society* e a *American Thoracic Society* . 

Transdutores de fluxo ou volume de ar disponíveis:

 – Pneumotacógrafo: transdutor de fluxo que mede a diferença de pressão do ar ventilado através de membrana de baixa resistência. 

 – Tubo de Pitot: medidor de fluxo que determina a diferença de pressão entre orifícios orientados na direção do fluxo de ar. 

 – Turbina: transdutor de volume no qual uma turbina ultraleve com contador luminoso de rotações é posicionada no fluxo de ar ventilado. 

### 4.3. Analisadores de Gás

 Existem dois tipos principais de analisadores de gás: 

 1) Espectrômetro de massa, considerado o padrão-ouro, sendo capaz de medir todos os gases coletados. É de alto custo e limitado emprego em modelos comerciais. 

2) Analisadores individuais:

 – Analisador de CO _2_ : baseado na absorção das moléculas de CO _2_ por infravermelho. 

 – Analisadores de O _2_ : paramagnético, que utiliza o efeito das moléculas de O _2_ em um campo magnético; ou eletroquímico, no qual as reações entre O _2_ e um substrato em alta temperatura são medidas por sensor. 

### 4.4. Medições das Trocas Gasosas

 Consideramos o VO _2_ e o VCO _2_ como medida da diferença entre os volumes inspirado e expirado desses gases. Equação do VO _2_ : 


$${VO}_{2}=\frac{\left(VInsp\times {FIO}_{2}\right)-\left(VExp\times {FEO}_{2}\right)}{\text{ Tempo }}$$

VInsp: volume de ar inspirado é calculado usando a premissa que a fração expirada e inspirada de nitrogênio permanece constante; VExp: volume de ar expirado; Tempo: minutos em que a medida é realizada; FIO_2_: concentração de O_2_ no ar inspirado; FEO_2_: concentração de O_2_ no ar expirado.

Métodos para medições das trocas gasosas:

 – Câmara de mistura: sistema com válvula respiratória bidirecional e câmara de medição contínua das concentrações de O _2_ e CO _2_ . São mais acurados para protocolos de carga fixa. Em protocolos incrementais, sua acurácia é semelhante ao sistema respiração-a-respiração. 

 – Respiração-a-respiração: é o mais utilizado. As concentrações de O _2_ e CO _2_ são medidas próximas à boca (amostras 50 a 100/min). Para evitar erros de mensuração, requer correções da saturação de vapor, temperatura, pressão atmosférica e do tempo de atraso entre a amostragem e a chegada do gás ao analisador. 

###  4.5. Procedimentos de Calibração, Controle de Qualidade e Higienização 

 Os procedimentos de calibração e controle de qualidade devem seguir as recomendações do fabricante do dispositivo quanto ao modo, frequência e periodicidade: 

 – Calibrações de volume e gás (realizadas rotineiramente). 

 – Calibração volumétrica por meio de seringa de 3 litros (variação volumétrica de até 3% é considerada aceitável). 

 – Calibração gasosa envolve ar ambiente e cilindro com mistura de CO _2_ e O _2_ . Equipamentos respiração-a-respiração realizam calibração adicional quanto ao atraso da amostragem e a chegada do gás ao analisador. ^1041^

 Higienização é um processo rotineiro que, além dos aspectos metodológicos em relação ao TE (ver na Parte 2, Seção 1.1.3), deve considerar a higienização constante de mãos, superfícies e dos equipamentos do TCPE, em consonância com os protocolos institucionais e recomendações das autoridades sanitárias. ^1042^

 A calibração e o controle de qualidade visam a qualidade e reprodutibilidade dos exames. Entretanto, existem múltiplos fatores de interferência, tais como mudanças do estado clínico, motivação e curva de adaptação do paciente. 

### 4.6. Protocolos

Os protocolos validados podem ser divididos em:

 – Incrementais: escalonados ou em rampa (o mais recomendado para a prática clínica, tanto para cicloergômetro quanto para esteira). 

 – Sem incremento (carga fixa): empregados em atletas, em pneumopatas para avaliação de curvas fluxo-volume e hiperinsuflação dinâmica. ^1043^

 Os protocolos e critérios para interrupção do esforço são semelhantes aos do TE descritos nesta diretriz. 

###  4.7. *Software* para Análise dos Dados 

 O *software* para o TCPE deve: 

 – Integrar/relacionar as variáveis do TE aos dados ventilatórios (ergoespirometria) e do analisador de gases, com possibilidade de determinar as variáveis pertinentes ao TCPE. 

 – Possibilitar a visualização dos dados de modo numérico e gráfico. 

 – Possibilitar a marcação do limiar anaeróbio, do ponto de compensação respiratória e do esforço máximo. 

 – Permitir a realização de curvas fluxo-volume em repouso e durante o exame. 

 – Apresentar os resultados de forma ordenada e clara, incluindo, quando possível, os respectivos valores de referência. ^1044^

### 4.8. Recomendações Prévias aos Pacientes

 São as mesmas referentes ao TE apresentadas na Seção 1.1.6 da Parte 2 desta diretriz. É obrigatória a assinatura de termo de esclarecimento/consentimento específico para o TCPE. 

##  5. Realização do TCPE em Algumas Situações Específicas 

### 5.1. Insuficiência Cardíaca

 O TCPE se constitui em clássica indicação na seleção de pacientes com IC terminal para transplante cardíaco (classe I B): quando em uso de betabloqueadores, VO _2_ pico <12,0 mL/kg/min; nos intolerantes aos betabloqueadores, VO _2_ pico <14,0 mL/kg/min. ^1045^ ^,^ ^1046^

 Os limiares ventilatórios possibilitam a prescrição ideal de exercícios aeróbicos, particularmente na reabilitação dos pacientes com IC. ^17^ ^,^ ^29^

 Variáveis úteis do TCPE na estratificação de risco e ajustes terapêuticos nos pacientes com IC ( Tabela 45 ): 

**Tabela 45:** – Alterações das variáveis do TCPE em pacientes com IC 223,233,849,850,1034,1057-1059

Variável	Alteração	Utilidade	Interpretação
** VO _**2**_ pico **	↓	Avaliação geral do desempenho ao esforço. Marcador prognóstico de mortalidade	– Classificação de Weber – Indicação transplante cardíaco: uso de betabloqueadores <12,0; nos intolerantes betabloqueadores, <14,0 mL/kg/min
** VO _**2**_ LV1 **	↓	Avaliação geral/ ↓ débito cardíaco	– Avaliação da gravidade/prognóstico
** PuO _**2**_**	↓	Débito cardíaco/função do VE	– Relação direta com o volume sistólico. – PuO _2_ em platô e/ou queda = disfunção VE
** Inclinação VE/VCO _**2**_**	↑	Incompatibilidade ventilação-perfusão/aumento do tônus simpático	– Preditor de mortalidade e hospitalização – Quanto maior a inclinação, pior prognóstico
** Relação VE/VCO _**2**_**	↑	Marcador prognóstico/doença pulmonar concomitante	– Relação >34 é preditora independente de pior prognóstico
** ΔVO _**2**_ /ΔWR **	↓	↓ Débito cardíaco/ ↓ entrega O _2_	– Disfunção do VE: achatamento da curva ∆VO _2_ /∆WR. Marcador prognóstico
**EOV **	Presente	↓ Débito cardíaco/resposta quimiorreflexa alterada	– Marcador de gravidade/prognóstico, principalmente se precoce e ciclos >1 min
PETCO _**2**_ *	↓	↓ Débito cardíaco/ ↑ resposta quimiorreflexa	– Reflete gravidade da doença.
**POC**	↑	Variável em esforço submáximo. Reflete a eficiência cardiorrespiratória	– Preditor de mortalidade CV e por todas as causas isoladamente ou associado a outras variáveis – POC ≥36 apresenta maior mortalidade CV e transplante cardíaco urgente
**OUES**	↓	Eficiência ventilatória de consumo de O _2_	– Valor prognóstico e risco de eventos
**Relação VD/VT**	↓	Aumento do espaço morto com esforço	– Marcador de prognóstico. Associa-se à dispneia esforço-induzida
VO _*2*_ pico: consumo de oxigênio no pico do esforço; VO _*2*_ LV1 = consumo de oxigênio no 1º limiar ventilatório (anaeróbio); PuO _*2*_ : pulso de oxigênio; Inclinação VE/VCO _*2*_ = eficiência ventilatória (ventilação/produção de CO _*2*_ ); ΔVO _*2*_ /ΔWR: relação consumo oxigênio pela taxa de trabalho; EOV: ventilação oscilatória ao esforço (do inglês, exercise oscilatory ventilation); PETCO _*2*_ LV1: pressão parcial expiratória final de dióxido de carbono no 1 ***º*** limiar ventilatório; POC: ponto ótimo cardiorrespiratório; OUES: inclinação da eficiência da captação do oxigênio (OUES, oxygen uptake efficiency slope); Relação VD/VT: relação espaço morto/volume corrente; CV: cardiovascular. *Melhor avaliado no 1 ***º*** limiar ventilatório (LV1).			

 1) VO _2_ max/pico é excelente marcador prognóstico de mortalidade. ^1045^ ^-^ ^1048^

 2) Classificação de Weber baseada no VO _2_ max/pico e taxas de mortalidade crescentes: A (VO _2_ >20 mL/kg/min), B (16-20 mL/kg/min), C (10-15 mL/kg/min) ou D (<10 mL/kg/min). ^1049^

 3) Valor da relação VE/VCO _2_ >34 é preditor independente de pior prognóstico na IC. ^1047^ ^,^ ^1048^

 4) Classificação de risco pela VE/VCO _2_ (segundo Arena et al.) para eventos (mortalidade, transplante ou implante de dispositivo de assistência ventricular esquerda) por classe ventilatória (CVent): CVent-I: ≤29; CVent-II: 30,0-35,9; CVent-III: 36,0-44,9; CVent-IV: ≥45,0. A sobrevida livre de eventos para indivíduos em CVent-I, II, III e IV, respectivamente, 97,2%, 85,2%, 72,3% e 44,2% (p<0,001). ^1050^

 5) Escore TCPE: baseado em combinação de variáveis do exame com pontuação >15 associada a elevado risco de eventos: curva VE/VCO _2_ >34 = 7 pontos; queda da FC no 1º minuto da recuperação <6 bpm = 4 pontos; OUES <1,47 L/min = 3 pontos; VO _2_ pico ≤14 mL/kg/min = 2 pontos; PETCO _2_ em repouso <33 mmHg = 2 pontos. ^1014^ ^,^ ^1021^ ^,^ ^1022^ ^,^ ^1030^

 6) Outras variáveis prognósticas utilizadas: VO _2_ no LV1, OUES, pulso de oxigênio, EOV e POC. ^1014^ ^,^ ^1019^ ^,^ ^1020^ ^,^ ^1025^ ^,^ ^1026^ ^,^ ^1032^ ^,^ ^1033^ ^,^ ^1051^ ^-^ ^1056^

### 5.2. Doença Arterial Coronariana

 Na doença arterial coronariana estável, essencialmente, o TCPE possibilita: 

 – Diagnóstico, discriminação dos aspectos fisiopatológicos e determinação da gravidade da isquemia esforço- induzida. ^737^ ^,^ ^1012^ ^,^ ^1013^ ^,^ ^1023^ ^-^ ^1029^ ^,^ ^1035^ ^,^ ^1060^ ^-^ ^1068^ ^,^ ^1069^

 – Determinação da aptidão cardiorrespiratória (ACR) pela mensuração direta do VO _2_ pico/máximo (padrão-ouro). ^737^ ^,^ ^1012^ ^,^ ^1069^ ^,^ ^1070^

 – Contribuição na definição e eventuais ajustes das intervenções terapêuticas. ^1013^ ^,^ ^1062^ ^,^ ^1063^ ^,^ ^1065^ ^,^ ^1066^ ^,^ ^1068^

 – Prescrição individualizada e otimizada de exercícios aeróbicos na reabilitação CV. 

Variáveis de diagnóstico e prognóstico na DAC:

 – Pulso de oxigênio, curva VE/VCO _2_ , PETCO _2_ e PETO _2_ , ∆VO _2_ /∆WR, as quais permitem a detecção da isquemia miocárdica esforço-induzida, dando ao TCPE maior sensibilidade e especificidade em relação ao TE. ^1013^ ^,^ ^1023^ ^,^ ^1024^ ^,^ ^1027^ ^-^ ^1029^ ^,^ ^1035^ ^,^ ^1061^ ^-^ ^1068^ ^,^ ^1071^

 – Variáveis de prognóstico: aptidão cardiorrespiratória (VO _2_ max/pico); VO _2_ no limiar de angina; POC. ^737^ ^,^ ^1012^ ^,^ ^1025^ ^,^ ^1026^ ^,^ ^1069^ ^,^ ^1070^

 – Pode ocorrer disfunção de VE, persistente ou transitória, com incompatibilidade da ventilação/perfusão (V/Q) por redução do fluxo sanguíneo pulmonar com ventilação adequada. Na disfunção do VE, observa-se anormalidade da PETCO _2_ (no LV1), da PETO _2_ (no LV1 e no pico do esforço) e dos índices com as quais ambas se correlacionam: VO _2_ pico, LV1, ∆VO _2_ /∆WR e inclinação aumentada da VE/VCO _2_ . ^1019^ ^,^ ^1020^ ^,^ ^1027^ ^,^ ^1029^ ^,^ ^1035^ ^,^ ^1060^ ^,^ ^1061^

### 5.3. Miocardiopatia Hipertrófica

 No TCPE de pacientes com miocardiopatia hipertrófica (MCH), considerar que ( Tabela 46 ): 

**Tabela 46:** – Alterações das variáveis do TCPE em pacientes com MCH 1057,1074-1077

Variável	Alteração	Utilidade	Interpretação
** VO _**2**_ pico **	↓	Avaliação desempenho ao exercício. Marcador prognóstico de mortalidade	– VO _2_ pico <20 mL/kg/min ou <80% do previsto associou-se a pior prognóstico (transplante cardíaco e hospitalização para redução do septo) – VO _2_ pico <50% do previsto associou-se à mortalidade geral e CV
** VO _**2**_ LV1 **	↓	Avaliação geral/ ↓ débito cardíaco	– Mecanismos semelhantes aos envolvidos na redução do VO _2_ pico – Relação direta com volume sistólico
** PuO _**2**_**	↓	Débito cardíaco/fortemente relacionada ao volume sistólico	– Achatamento precoce em torno de 50% a 60% da carga máxima de esforço, devido à redução do volume sistólico – Quanto mais precoce o achatamento do PuO _2_ , maior a gravidade da MCH
** ΔVO _**2**_ /ΔWR **	↓	↓ Débito cardíaco/VO _2_	– Preservado ou ligeiramente reduzido na maioria dos pacientes – Redução da inclinação ou inclinação lenta na última parte do esforço sugerem comprometimento diastólico e/ou estágio final da doença – Mudança abrupta na linearidade ΔVO _2_ /ΔWR indica isquemia miocárdica associada
** Relação VE/VCO _**2**_**	↑	Prognóstico/mortalidade	– Relação >34 é preditora de mortalidade geral e transplante cardíaco
* VO _*2*_ pico: consumo de oxigênio no pico do esforço; VO _*2*_ LV1 = consumo de oxigênio no 1 ***º*** limiar ventilatório (anaeróbio); PuO _*2*_ : pulso de oxigênio; ΔVO _*2*_ /ΔWR: relação consumo oxigênio pela taxa de trabalho; Relação VE/VCO _*2*_ = eficiência ventilatória (relação ventilação/produção de CO _*2*_ ). *			

 – O VO _2_ máximo ou o VO _2_ pico (quando não se atinge o QR ≥1,10) têm valor prognóstico. ^17^ ^,^ ^29^ ^,^ ^1012^ ^,^ ^1072^ ^,^ ^1073^

 – É possível identificar os pacientes com obstrução da via de saída de VE esforço-induzida, particularmente pelo pulso de O _2_ , permitindo ajustes terapêuticos e da intensidade do exercício na reabilitação CV. ^219^ ^,^ ^1021^ ^,^ ^1072^ ^,^ ^1073^

5.4. Valvopatias

 O TCPE nas valvopatias, particularmente na estenose aórtica (EAo), contribui para avaliação da repercussão clínica-funcional e nas decisões de intervenções terapêuticas. ^939^ ^,^ ^1078^

Em relação à EAo:

 – O prognóstico piora com a presença de sintomas esforço-induzidos. 

 – TCPE é relevante para o aprimoramento diagnóstico e prognóstico, e diferenciação da limitação ao esforço, principalmente em sedentários, na intolerância ao esforço ou pacientes com múltiplas comorbidades. 

 – TCPE máximo deve ficar restrito aos pacientes assintomáticos ou questionavelmente sintomáticos, havendo contraindicação absoluta para os indiscutivelmente sintomáticos. ^1079^ ^,^ ^1080^

### 5.5. Pneumopatias

#### 5.5.1. Doença Pulmonar Obstrutiva Crônica

 A gravidade da doença pulmonar obstrutiva crônica (DPOC) pode ser estabelecida pelos sintomas e dados do teste de função pulmonar (espirometria). No entanto, a espirometria em repouso não permite determinar o grau de intolerância ao esforço. ^1081^

 A incapacidade de aumentar a ventilação para permitir níveis adequados de trocas gasosas (limitação ventilatória) é característica dos quadros pulmonares obstrutivos, mas pode também ocorrer nas doenças restritivas (doenças intersticiais e anormalidades da caixa torácica). A reserva ventilatória (RV) no pico do esforço <15%, principalmente quando o QR <1,00, permite o diagnóstico de limitação ventilatória. ^337^ ^,^ ^1081^ ^-^ ^1083^

 No DPOC, o VO _2_ pico é o melhor índice definidor da aptidão cardiorrespiratória (ACR), quando o paciente se exercitou até o seu limite. Adicionalmente, considerar a ocorrência de baixa ACR associada a alta demanda ventilatória e fadiga acentuada em membros inferiores. ^1081^

 A hiperinsuflação pulmonar dinâmica é um dos fatores que podem causar dispneia intolerável durante o exercício. Com o aumento do fluxo respiratório durante o exercício, pode haver aprisionamento aéreo com um aumento progressivo do volume residual e, consequentemente, redução da capacidade inspiratória (CI). Frequentemente, isso ocorre em conjunto com uma redução no volume corrente (VC), indicando que a mecânica ventilatória atingiu seu limite. No TCPE, a hiperinsuflação dinâmica pode ser melhor observada quando são realizadas análises periódicas da curva de fluxo-volume com medida da CI durante o exercício, principalmente quando há desproporção entre a intensidade dos sintomas e o grau de obstrução das vias aéreas. ^1081^ ^,^ ^1082^

 A hiperinsuflação pulmonar dinâmica (HPD), um dos mecanismos ventilatórios que pode causar dispneia intolerável ao esforço, decorre de aumento do fluxo respiratório, aprisionamento aéreo com aumento progressivo do VR, redução no volume corrente (VC), indicando consequente redução da capacidade inspiratória (CI). No TCPE, a HPD pode ser observada por meio de análises periódicas da curva de fluxo-volume com medida da CI, evidenciando desproporção entre a intensidade dos sintomas e o grau de obstrução das vias aéreas. ^1081^ ^,^ ^1082^

#### 5.5.2. Doença Vascular Pulmonar

 A hipertensão arterial pulmonar (HAP), definida por pressão média da artéria pulmonar (PAPmd) ≥25 mmHg, apresenta a dispneia ao esforço como seu sintoma mais precoce. ^1084^ A PAPmd em repouso frequentemente é normal nos estágios iniciais da doença vascular pulmonar (DVP), só alterando quando mais de 50% da circulação pulmonar estiver obstruída, causando um diagnóstico da tardio HAP. ^246^ ^,^ ^1085^ ^-^ ^1092^ A identificação da HAP pelo TCPE pode ser feita por método invasivo (padrão-ouro) ou não invasivo ( Tabela 47 ). 

**Tabela 47:** – Comportamento das principais variáveis do TCPE em pacientes com doenças vasculares pulmonares 246,1095-1098

Variável	Hipertensão arterial pulmonar	Hipertensão pulmonar tromboembólica crônica	Doença veno-oclusiva pulmonar
** VO _**2**_ pico **	↓	↓	↓
** VO _**2**_ LV1 **	↓	↓	↓ ↓
** PuO _**2**_**	↓	↓	↓
** Inclinação VE/VCO _**2**_**	↑	↑ ↑	↑ ↑
** ΔVO _**2**_ /ΔWR **	↓	↓	↓
** PETCO _**2**_**	↑	↑ ↑	↑ ↑
** SatO _**2**_**	↓*	↓ ↓	↓ ↓
**Relação VD/VT**	↑**	↑ ↑	↑ ↑
VO _*2*_ pico: consumo de oxigênio no pico do esforço; VO _*2*_ LV1 = consumo de oxigênio no 1 **º** limiar ventilatório (anaeróbio); PuO _*2*_ : pulso de oxigênio; Inclinação VE/VCO _*2*_ = eficiência ventilatória (ventilação/produção de CO _*2*_ ); ΔVO _*2*_ /ΔWR: relação consumo oxigênio pela taxa de trabalho; EOV: ventilação oscilatória ao esforço (do inglês, exercise oscilatory ventilation); PETCO _*2*_ : pressão parcial expiratória final de dióxido de carbono; POC: ponto ótimo cardiorrespiratório; OUES: inclinação da eficiência da captação do oxigênio (OUES, oxygen uptake efficiency slope); Relação VD/VT: relação espaço morto/volume corrente; SatO _*2*_ : saturação arterial de oxigênio. *Queda >3% sem aumento da PaCO _*2*_ (tensão arterial de dióxido de carbono). **Aumento >30% durante o esforço.			

 O método invasivo, disponível em poucas instituições no mundo, requer uso de cateter na artéria pulmonar para medida direta da PADmed. Seu valor de referência é foi determinado arbitrariamente (>30 mmHg). Esse método não é recomendado para uso rotineiro na detecção precoce de DVP. ^1092^ ^-^ ^1094^

 O método não invasivo é útil na avaliação de paciente com dispneia de etiologia indefinida e/ou suspeita de DVP, considerando que: 

 – As razões VE/VCO _2_ no LV1 e no pico do esforço são muito elevadas na HAP. ^1092^

 – Baixos valores de PETCO _2_ no repouso e no esforço, com aumento da PETO _2_ , foram associadas à HAP por incompatibilidade da relação V/Q (redução do fluxo sanguíneo pulmonar com ventilação adequada). 

 – VE/VCO _2_ >37 e PETCO _2_ <30 mmHg no POC podem indicar DVP, na ausência de hiperventilação aguda. 

 – Valores excepcionalmente baixos de PETCO _2_ (<20 mmHg) sugerem HAP como causa da dispneia ao esforço. ^1091^

 No TCPE utilizado na avaliação da gravidade da HAP estabelecida e na resposta terapêutica: 

 – Na HAP idiopática, o VO _2_ pico <10,4 mL/kg/min e a PASpico <120 mmHg são de pior prognóstico ( Tabela 48 ). ^246^

**Tabela 48:** – Avaliação do risco através das variáveis do TCPE em pacientes com HAP 246,1095,1097,1099

Variável	Comportamento com progressão da HAP	Baixo risco (<5%)	Intermediário (5-10%)	Alto risco (>10)
**Classe funcional**	↓	I e II	III	IV
** VO _**2**_ pico (mL/kg/min) **	↓	>15	11-15	<11
** %VO _**2**_ predito **	↓	>65	65-35	<35
** Relação VE/VCO _**2**_**	↑	<36	36-45	>45
* VO _*2*_ pico: consumo de oxigênio no pico do esforço; %VO _*2*_ predito = porcentagem alcançada do consumo de oxigênio predito; Relação VE/VCO _*2*_ = eficiência ventilatória (relação da ventilação/produção de CO _*2*_ ). *				

 – VO _2_ >15,0 mL/kg/min indica bom prognóstico. ^1086^ ^,^ ^1093^

 – VE/VCO _2_ no LV1 (≥54) e no esforço máximo (≥62) indicam pior prognóstico/menor sobrevida. ^1089^

 – Curva VE/VCO _2_ menos inclinada (valores mais elevados) é observada na HAP por tromboembolismo pulmonar (TEP) crônico. Valores elevados de VE/VCO _2_ em fases precoces do TEP não se associaram à classe funcional/gravidade. ^1088^

## 5.6. Diagnóstico Diferencial da Dispneia

 O TCPE é ferramenta importante no diagnóstico diferencial da dispneia esforço-induzida (DEI), particularmente nas de causa pulmonar e cardíaca. ^219^ ^,^ ^229^ ^,^ ^1018^ ^,^ ^1100^ ^,^ ^1101^ A baixa aptidão cardiorrespiratória, comum às prováveis etiologias da DEI, não é bom parâmetro isolado para o diagnóstico diferencial. ^219^ ^,^ ^229^ ^-^ ^231^ ^,^ ^1018^ ^,^ ^1100^ ^-^ ^1104^

 Parâmetros utilizados no diagnóstico diferencial da DEI ao esforço: ^219^ ^,^ ^229^ ^-^ ^231^ ^,^ ^1018^ ^,^ ^1100^ ^-^ ^1104^

 – Eficiência ventilatória avaliada por meio do VE/VO _2_ e VE/VCO _2_ , reserva ventilatória e relação espaço morto/volume corrente (VD/VT). 

– Análise da alça fluxo-volume.

 – Saturação arterial de oxigênio, que contribui para detecção dos distúrbios na difusão pulmonar. 

 Nas doenças pulmonares, a DEI está associada a equivalentes ventilatórios elevados, RV reduzida, aumento da relação VD/VT, PETCO _2_ reduzida e dessaturação arterial de oxigênio, observados em conjunto ou separadamente. ^219^ ^,^ ^229^ ^-^ ^231^ ^,^ ^1018^ ^,^ ^1100^ ^-^ ^1104^

 Em relação às limitações ventilatórias da DEI ( Tabela 49 ): ^219^ ^,^ ^229^ ^-^ ^231^ ^,^ ^1018^ ^,^ ^1100^ ^-^ ^1104^

**Tabela 49:** – Diagnóstico diferencial das principais causas de dispneia no TCPE 220,222,1018,1108

Variável	Cardíaca	Pulmonar	Vascular-pulmonar	Hiperventilação
**VO**_**2**_ **pico **	Reduzido	Reduzido	Reduzido	Normal
**LV1 **	Precoce	Normal	Precoce	Normal
**QR **	Normal	Reduzido	Normal/reduzido	Normal/reduzido
**Relação VE/VCO**_**2**_	Elevado	Elevado	Elevado	Elevado
**PuO**_**2**_	Reduzido/platô	Normal	Reduzido	Normal
**SatO**_**2**_	Normal	Queda	Queda	Normal
**ΔVO**_**2**_ **/ΔWR **	Reduzido	Normal	Reduzido	Normal
* VO _*2*_ pico: consumo de oxigênio no pico do esforço; LV1 = 1 **º** limiar ventilatório (anaeróbio); QR: coeficiente respiratório; Relação VE/VCO _*2*_ = eficiência ventilatória (relação ventilação/produção de CO _*2*_ ); PuO _*2*_ : pulso de oxigênio; SatO _*2*_ : saturação arterial de oxigênio por oxímetro; ΔVO _*2*_ /ΔWR: relação consumo oxigênio pela taxa de trabalho. *				

 – A espirometria precedendo o TCPE permite a detecção e caracterização dos distúrbios ventilatórios (obstrutivos, restritivos e mistos). 

– Frequente não detectação do LV1 (com QR <1,0).

 – Na interrupção do esforço decorrente da limitação ventilatória em geral observam-se valores baixos de VO _2_ e FCpico. 

 – Redução na saturação arterial de oxigênio >5% (oximetria de pulso) indica limitação pulmonar. 

 – As razões entre ΔVO _2_ /ΔWR e VO _2_ /FC (pulso de oxigênio) são normais, exceto quando associado a HAP com débito cardíaco comprometido. 

 – Na espirometria realizada em até 30 minutos após pico do esforço (pós-esforço), a redução do VEF1 ≥15% indica broncoespasmo esforço-induzido. 

 Diagnóstico diferencial da DEI por IC: ^219^ ^,^ ^229^ ^,^ ^1014^ ^,^ ^1018^ ^-^ ^1020^ ^,^ ^1028^ ^-^ ^1033^ ^,^ ^1035^ ^,^ ^1037^ ^-^ ^1039^ ^,^ ^1048^ ^-^ ^1056^ ^,^ ^1060^ ^,^ ^1061^ ^,^ ^1105^

 – VE/VCO _2_ elevada e VO _2_ máximo reduzido, correlacionados com a gravidade da IC. 

 – Reserva ventilatória preservada, frequentemente sem dessaturação arterial de oxigênio, indica causa cardíaca. Exceção para as cardiopatias congênitas complexas, que cursam com dessaturação. 

 – Relação VD/VT aumentada e EOV presente indicam pior prognóstico da IC. 

 Causas cardíacas e pulmonares podem se sobrepor gerando padrões mistos, refletindo efeitos secundários do distúrbio principal ou a associação das comorbidades. 

 Devido à COVID-19, existem pacientes cursando com dispneia e/ou intolerância ao esforço, inclusive na forma crônica da doença. Nesse contexto, devem ser cogitadas sequelas: fibrose pulmonar, tromboembolismo pulmonar, miocardite, disfunção diastólica e/ou sistólica dos ventrículos, miopatia. ^1106^ ^,^ ^1107^

## 5.7. Atletas e Exercitantes

 Os atletas compõem uma população heterogênea. Podem ser de modalidades de alta potência aeróbica (maratonista) ou de modalidade primariamente técnica e de pouca exigência física (tiro ao alvo). ^1106^

O TCPE visa principalmente a:

 – Determinação direta do VO _2_ , o padrão-ouro na avaliação da aptidão cardiorrespiratória. 

 – Determinação dos limiares ventilatórios (LV1 e LV2), possibilitando prescrição individualizada e otimizada do treinamento aeróbico. ^219^ ^,^ ^229^ ^,^ ^293^ ^,^ ^1011^

## 5.8. Reabilitação Cardiorrespiratória

 Na prescrição de exercícios de pacientes na reabilitação cardíaca, devem ser considerados principalmente: 

 – Determinação da aptidão cardiorrespiratória através do VO _2_ máximo. ^737^ ^,^ ^1012^ ^,^ ^1069^ ^,^ ^1070^

 – Os limiares ventilatórios (LV1 e LV2) para definição individualizada da zona de treinamento aeróbico. ^17^ ^,^ ^219^ ^,^ ^229^ ^,^ ^293^ ^,^ ^1011^

 – Os limiares de claudicação de membros inferiores (inicial e absoluta), determinados em esteira ergométrica. 

 – Na DAP, a utilização de bicicleta ergométrica permitirá melhor avaliação cardiopulmonar. 

 – O limiar de isquemia e suas repercussões clínicas (angina, arritmias, hipotensão) visando a ajustes finos da terapêutica e da intensidade do exercício. ^17^ ^,^ ^29^

 – Em pacientes com IAM prévio com disfunção do VE, a reabilitação aumenta a PETCO _2_ associada à melhora do débito cardíaco. ^1019^ ^,^ ^1020^

## 6. Interpretação e Elaboração do Laudo do TCPE

 A interpretação e a descrição de todas variáveis do TE devem ser incorporadas ao laudo do TCPE conforme constam nesta diretriz. 

Recomenda-se constar no laudo:

 – Descrição detalhada do comportamento das variáveis do TCPE, particularmente aquelas alteradas e/ou relacionadas ao motivo de solicitação do exame. 

 – Valores de referência das variáveis, se disponível com dados da população pertinente ao paciente (idade, sexo, raça, nível de atividade física diária etc.). 

 – Gráficos e tabelas das variáveis relevantes e suas respectivas interpretações, baseadas na indicação do exame e eventuais diagnósticos. 

 – Informações referentes à estratificação de risco/prognóstico. 

 Considerando que a prescrição de exercícios é de responsabilidade do médico assistente, não se recomendada a liberação automática dessa prescrição, baseada exclusivamente no TCPE. 

##  Parte 4 – Teste Ergométrico Associado aos Métodos de Imagem em Cardiologia 

##  1. Estresses Cardiovasculares Associado aos Métodos de Imagem em Cardiologia 

 Segundo a Atualização da Diretriz Brasileira de Cardiologia Nuclear, o princípio do estresse cardiovascular associado às imagens de perfusão miocárdica e ecocardiografia de estresse consiste em criar heterogeneidade de fluxo sanguíneo entre territórios vasculares irrigados por artérias coronárias normais e com lesões obstrutivas. ^1109^ ^,^ ^1110^

 O TE e as provas farmacológicas, na CPM e no EcoE, são utilizados para avaliação da heterogeneidade de fluxo regional, e as imagens resultantes mostram sensibilidade e especificidade semelhantes para a detecção de DAC. ^8^ ^,^ ^1111^ ^,^ ^1112^

### 1.1. Cintilografia Perfusional Miocárdica

**Figura 34:**
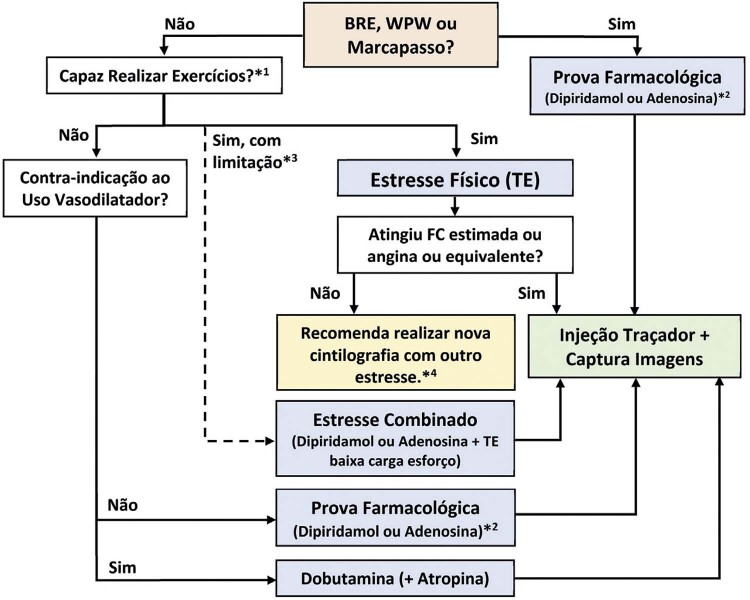
– Escolha da modalidade de estresse a ser aplicada na cintilografia perfusional miocárdica para diagnóstico de doença arterial coronariana. BRE: bloqueio de ramo esquerdo; TE: teste ergométrico; WPW: síndrome de Wolff-Parkinson-White; FC: frequência cardíaca; * 1 Capacidade funcional para realizar atividades físicas diárias estimadas >5 METs e habilidade para execução do esforço no ergômetro disponível; * 2 Outra opção: Regadenoson (ainda não disponível no Brasil); * 3 Possibilidade de realização a critério do médico solicitante. * 4 Por ser um exame diagnóstico, necessita realizar uma nova cintilografia sob estresse farmacológico (dipiridamol ou adenosina) ou estresse combinado, visando manter a acurácia do método.

 As duas modalidades de estresses cardiovasculares existentes (exercício físico ou vasodilatação farmacológica) mostram sensibilidade e especificidade semelhantes para a detecção de DAC pela análise das imagens de perfusão. ^9^ A Figura 34 apresenta organograma com ordenamento para a escolha da modalidade de estresse a ser aplicada. 

####  1.1.1. Metodologia do Estresse Físico – Teste Ergométrico 

 O estresse físico, através do TE ou TCPE, adiciona valor de diagnóstico e prognóstico aos métodos de imagem por abordar parâmetros clínicos, hemodinâmicos e eletrocardiográficos. As peculiaridades metodológicas referentes a cada um dos métodos de imagem abordadas a seguir deverão ser respeitadas e adotadas para obtenção dos melhores resultados. ^9^ ^,^ ^762^ ^,^ ^1111^

**1.1.1.1. Contraindicações à Realização do Estresse Físico na CPM**

 As contraindicações à realização do estresse físico são as mesmas consideradas em relação ao teste ergométrico descritas na Parte 1, Seção 2.3 desta diretriz. 

**1.1.1.2. Orientações para Marcação do Estresse Físico na CPM**

 O preparo para o procedimento do estresse físico envolve: 

 – Jejum de 3 horas, com refeição leve previamente ao exame. 

 – Cafeína (metil-xantinas), presente em bebidas, alimentos e medicamentos, deve ser evitada 12 horas antes do exame, similarmente ao estresse com vasodilatador, considerando que alguns exames podem ser convertidos para farmacológico ou híbridos ( Anexo 3 ). 

 – A descontinuação de medicações que podem interferir no TE/TCPE (em especial antiarrítmicos e antianginosos: betabloqueadores, bloqueadores de canais de cálcio e nitratos) deve seguir o preconizado na Seção 1.1.6 da Parte 2 desta diretriz, sendo delegada ao médico solicitante. ^1111^

 – Recomenda-se ao paciente trazer TE/TCPE realizados recentemente de modo a permitir melhor escolha do protocolo de esforço e comparação entre exames. 

 – Adotar as demais orientações para realização do TE descritas na Parte 2 desta diretriz. 

**1.1.1.3. Realização do Estresse Físico na CPM**

 Na CPM, é necessário acesso venoso prévio ao TE em um dos membros superiores para a injeção do radiofármaco. ^6^ A injeção deverá ser feita no pico do esforço, que poderá não corresponder à FC máxima prevista, pois deve-se levar em consideração a indicação e objetivo do exame (diagnóstico ou avaliação terapêutica) – GR-NE: IIb-C. ^1111^

 Após essa injeção, deve-se estimular a continuidade do esforço, na mesma carga, por mais um minuto, objetivando a melhor captação miocárdica do radiotraçador. Se for inviável manter a carga, recomenda-se tentar reduzir a velocidade e/ou inclinação do ergômetro antes de interrupção abrupta. ^9^

 Realizar a aquisição das imagens após o término da fase de estresse: 

 – Com a MIBI- ^99^ ^m^ Tc , entre 30 e 60 minutos (empregado na maioria dos serviços no Brasil). 

 – No caso do Tálio-201, iniciar no máximo entre 10 a 15 minutos, para evitar que o fenômeno da redistribuição seja significativo. As imagens iniciais ou de distribuição devem ser adquiridas após a injeção do radioisótopo (injeção única) e as tardias (redistribuição) após 3 a 4 horas. 

 A exposição à radiação pela equipe de realização do TE varia amplamente, dependendo da carga de trabalho e procedimentos de prevenção da instituição, mas a dose efetiva para os médicos executantes do TE geralmente está abaixo dos limites aceitáveis. Recomenda-se a leitura e a adoção de todos os procedimentos preconizados pela Comissão Nacional de Energia Nuclear quanto à CPM. 

**1.1.1.4. Interpretação do TE na CPM**

 Todos os marcadores de diagnóstico e prognóstico descritos nesta diretriz quanto ao TE são válidos na interpretação do TE associado à CPM e deverão constar no relatório final. O percentual da FC máxima prevista no momento de injeção do radiofármaco deverá constar do relatório para permitir análise e interpretação da CPM. 

## 1.1.2. Metodologia das Provas Farmacológicas

 As provas farmacológicas (PF) são realizadas com: vasodilatadores primários (adenosina e dipiridamol) que causam redistribuição de fluxo coronariano; vasodilatadores como a dobutamina e atropina, que provocam efeitos semelhantes aos observados no exercício com aumento do trabalho miocárdico. 

 As PF devem ser feitas em pacientes: com limitação física por patologias com comprometimento osteomuscular e neuromuscular impeditivos; em uso de fármacos que interferem no aumento do consumo miocárdico de oxigênio (MVO _2_ ) em exames de diagnóstico; com baixa capacidade funcional; IC compensada; BRE e presença de marca-passo artificial; contraindicações para realização do TE. 

**1.1.2.1. Fármacos que Promovem Vasodilatação**

**1.1.2.1.1. Dipiridamol**

 O dipiridamol atua por inibição da enzima adenosina-deaminase, que degrada a adenosina endógena, além de bloquear a recaptação da adenosina pela membrana celular, com consequente aumento da concentração extracelular e vasodilatação coronária e sistêmica. Sua meia-vida biológica é de aproximadamente 45 minutos. A dose preconizada para a realização da CPM é de 0,56 miligramas por quilo por minuto (mg.kg ^-^ ^1^ ), até o máximo de 60 mg diluídos em 50 mL de soro fisiológico, com administração intravenosa (IV) em 4 minutos, que pode ser realizada manualmente sem bomba de infusão. O radiofármaco é administrado pelo mesmo acesso venoso, 2 a 4 minutos após o término do dipiridamol (momento de hiperemia ou vasodilatação máxima). ^9^ ^,^ ^1109^ ^-^ ^1111^

 Os efeitos adversos dos vasodilatadores ocorrem em 50% dos pacientes com dipiridamol e em 80% com a adenosina. No dipiridamol as manifestações são revertidas com a administração de aminofilina intravenosa, na dose de 1 a 2 mg.kg ^-^ ^1^ até 240 mg, 2 minutos após a injeção do radiotraçador. A acurácia para a detecção de DAC é comparável entre os fármacos vasodilatores (sensibilidade e especificidade ≈80-90%) e o estresse físico no TE (sensibilidade e especificidade ≈85-90%). ^1113^ ^,^ ^1114^

**1.1.2.1.2. Adenosina**

 A adenosina induz vasodilatação coronária por ativação específica dos receptores A _2A_ da membrana celular, resultando em aumento do fluxo coronário em até 4 a 5 vezes. É utilizada na dose de 140 μg.kg ^-^ ^1^ .min ^-^ ^1^ , administrada obrigatoriamente em bomba de infusão contínua em 6 minutos. O radiofármaco é injetado no 3° minuto por outra via de acesso venoso, devido à meia-vida plasmática ultracurta (2 a 10 segundos), sendo a infusão continuada por mais 3 minutos. 

 Quando o paciente é muito sintomático ou com alterações isquêmicas no início da infusão da adenosina, pode-se optar por protocolo mais curto de 4 minutos de infusão que mostrou capacidade de detectar isquemia e DAC; o radiofármaco é injetado no 2º minuto e a infusão da adenosina continuada por mais 2 minutos. 

 Observação clínica e registros de ECG, da PA e FC devem ser contínuos durante e alguns minutos após a prova farmacológica com os vasodilatadores. 

Preparo para o procedimento:

– Jejum de 3 horas.

 – Evitar o consumo de qualquer produto (alimento ou bebida) contendo metilxantinas (inibidores competitivos dos receptores de adenosina), incluindo café, chás, chocolates, energéticos, refrigerantes ou outras bebidas contendo cafeína, medicamentos (analgésicos, antigripais, relaxantes musculares e anti-inflamatórios que contenham cafeína) nas 12 horas antes do exame. 

 – No caso de uso de adenosina, a suspensão deve ser por pelo menos 12 horas ^1111^ antes do exame e, no caso do dipiridamol, por pelo menos 24 horas. ^9^

 – Teofilina deve ser suspensa pelo menos 12 horas antes do exame. 

 – No uso da adenosina, são necessários dois acessos venosos periféricos: uma via para adenosina e outra para injeção do radiofármaco. No dipiridamol, é necessária apenas uma via de acesso. 

 – Bomba de infusão para administração da adenosina em dose constante e facultativa no dipiridamol. 

 – Aminofilina (dose de 1 a 2 mg.kg ^-1^ ou 72 mg a 240 mg) deve ficar prontamente disponível para reversão dos efeitos colaterais graves dos estresses com vasodilatador. No caso de efeitos adversos pelo dipiridamol, utilizar a aminofilina após 2 minutos da injeção do traçador. 

 Contraindicações: ^1110^ ^,^ ^1111^ ^,^ ^1115^

 1) Relativas: história de doença pulmonar reativa sem crises nos últimos 3 meses; doença do nó sinusal; bradicardia sinusal acentuada; doença carotídea grave bilateral. 

 2) Absolutas: broncoespasmo em atividade; estado de mal asmático; crises de hiper-reatividade recentes (<3 meses); bloqueio atrioventricular de 2º ou 3º graus sem marca-passo; hipotensão arterial sistólica (<90 mmHg); AVC ou AIT recentes (<2 meses); uso recente (<24 horas) de dipiridamol em pacientes que receberão adenosina. 

**1.1.2.2. Fármacos que Promovem a Elevação do Consumo de Oxigênio Miocárdico**

 Esses fármacos representam alternativa para os pacientes que não podem submeter-se ao TE ou provas farmacológicas com dipiridamol ou adenosina. O mais utilizado é a dobutamina, cuja ação é predominante nos receptores beta-1 (ß-1) adrenérgicos com estimulações inotrópica e cronotrópica, menor efeito sobre os receptores beta-2 (ß-2), com resposta de vasodilatação periférica dependente da dose infundida. Produz aumento do débito cardíaco, da FC e da PAS, levando ao aumento do MVO _2 _ e, consequentemente, à vasodilatação coronariana. 

 A administração intravenosa da dobutamina é feita em bomba de infusão com dose inicial de 5-10 μg.kg ^-1^ .min ^-1^ por 3 minutos, seguindo-se por doses incrementais de 20 μg.kg ^-1^ .min ^-1^ , 30 μg.kg ^-1^ .min ^-1^ até o máximo de 40 μg.kg ^-1^ .min ^-1^ . ^1116^ Nos pacientes que não alcançarem a FC submáxima e sem evidências de isquemia, pode-se associar atropina intravenosa na dose de 0,25 a 2 mg. ^1110^ Se necessário, adicionar manobras de esforço “ *handgrip* ” (p. ex., compressão manual de uma bola de tênis). O uso precoce de atropina após a fase inicial da dobutamina mostrou-se seguro, reduziu o tempo de infusão e as queixas do paciente durante o estresse, sem alterar a acurácia do exame. ^1117^

 O radiotraçador é injetado na FCpico, com infusão de dobutamina que deve ser continuada por mais 1 minuto. 

 Durante todo o exame, devem ser monitorados os sinais e sintomas clínicos e registrados ECG, PA e FC. Para a reversão dos efeitos adversos, betabloqueadores de ação curta (p. ex., metoprolol, esmolol) podem ser injetados via intravenosa após o primeiro minuto da injeção do radiotraçador. 

Preparo para o procedimento:

– Jejum de 3 horas.

 – Descontinuar o uso de betabloqueador por 48 a 72 horas antes da prova com dobutamina. 

 – Acesso venoso periférico para infusão da dobutamina e radiotraçador. 

 – Utilizar bomba de infusão para a administração da dobutamina. 

 – Metoprolol (dose de 5 mg) deve ficar prontamente disponível para reversão dos efeitos colaterais graves da dobutamina, sendo contraindicado em pacientes com história prévia de broncoespasmos graves e na DPOC. Nesses pacientes, preferir o uso do esmolol (em dose única entre 100 e 200 mg) por ser um betabloqueador cardiosseletivo. 

Contraindicações:

 1) Relativas: aneurismas de aorta abdominal (>5cm de diâmetro), presença de trombos em ventrículo esquerdo, fração de ejeção de VE <25% (aumento de risco de arritmias ventriculares). 

 2) Absolutas *:* arritmias cardíacas (fibrilação atrial ou taquicardia ventricular sustentada ou não), estenose aórtica grave, cardiomiopatia hipertrófica obstrutiva, hipotensão sistólica (<90 mmHg), hipertensão arterial sistêmica (>200 mmHg), angina instável ou IAM recente, aneurismas ou dissecção aórtica, insuficiência vascular cerebral sintomática, presença de CDI, alterações metabólicas do potássio. ^9^

### 1.1.3. Metodologia do Estresse Combinado

 É ideal para pacientes que tenham limitação de exercitar-se ou estão em uso de medicações que impedem o aumento da FC. O estresse combinado não é recomendado em pacientes com bloqueio de ramo esquerdo, WPW e marca-passo. 

 A associação dos vasodilatadores ao exercício com baixa carga de trabalho (p. ex., até o segundo estágio do protocolo de Bruce ou até ligeiro cansaço) tem evidenciado redução da atividade subdiafragmática (hepática) e melhora na qualidade das imagens. ^1118^ Também contribui para a diminuição da ocorrência e intensidade dos efeitos adversos do dipiridamol ou adenosina. 

 Na combinação de baixo nível de exercício com dipiridamol, em pacientes selecionados, pode ser empregado o primeiro estágio do protocolo de Bruce modificado (1,7 mph e 0% de inclinação) por 4 a 6 minutos, logo após a infusão do dipiridamol. O radiofármaco é então injetado durante o esforço, que deve continuar por mais 2 minutos para permitir adequada captação miocárdica, reduzindo os efeitos colaterais e melhorando a qualidade das imagens. Quanto ao preparo para o procedimento, observar as orientações prévias referentes ao estresse físico e com fármacos que promovem vasodilatação. 

### 1.1.4. Novos Fármacos

 O agonista seletivo do receptor A _2A _ que promove vasodilatação coronariana (regadenoson) tem evidenciado hiperemia coronariana adequada e menor intensidade de efeitos sistêmicos (dor torácica e bloqueios atrioventriculares). O regadenoson, ainda não está disponível no Brasil, tem meia-vida biológica curta (2 a 4 minutos na primeira fase), diminui o tempo do exame, minimiza e limita a duração dos efeitos adversos. Pode ser utilizado em pacientes com DPOC ou asma. ^1119^

 A dose intravenosa recomendada do regadenoson é de 0,4 mg (não requer ajuste em relação ao peso), em veia periférica, seguida de *flush* de 5 mL de solução fisiológica e, após 10 a 20 segundos, administrar o radiofármaco. ^1120^

## 1.2. Ecocardiografia sob Estresse

 As modalidades de estresse foram comparadas anteriormente nesta diretriz, inclusive quanto aos seus efeitos fisiológicos, resposta hemodinâmica e contraindicações ( Tabela 18 ). Na maioria dos pacientes adultos capazes de se exercitarem, o estresse físico (TE) é considerado a modalidade de escolha para avaliação de isquemia miocárdica. 

###  1.2.1. Metodologia 8,260,265,1121,1122 

 Inicialmente, deve-se realizar ecocardiograma basal (EcoB) em repouso, antes do início do estresse, incluindo: avaliação da estrutura cardíaca (tamanhos das câmaras e espessura das paredes); análise das valvas cardíacas e seus gradientes; função ventricular segmentar e global. 

 O EcoB também visa investigar possíveis causas para a sintomatologia do paciente (p. ex., dissecção aórtica) e identificar qualquer condição que possa tornar o estresse inseguro (p. ex., valvopatia grave em paciente sintomático). Nessas circunstâncias, deve-se avaliar criteriosamente a possibilidade de adiamento ou cancelamento do exame sob estresse. 

 A aquisição, a análise e a interpretação dos dados ecocardiográficos (basal e estresse) deverão seguir o preconizado pelas entidades internacionais ^260,265,1121^ e diretrizes da SBC: Posicionamento sobre Indicações da Ecocardiografia em Adultos; ^8^ Posicionamento sobre Indicações da Ecocardiografia em Cardiologia Fetal, Pediátrica e Cardiopatias Congênitas do Adulto; ^1121^ Normatização dos Equipamentos e Técnicas de Exame para Realização de Exames Ecocardiográficos. ^1122^

 As imagens obtidas em repouso serão comparadas com as obtidas durante e após o estresse. A obtenção de dados em vários ciclos cardíacos no pico de estresse aumenta a precisão da interpretação do exame. Recomenda-se a gravação contínua das imagens de repouso e estresse. 

 No estresse em esteira, as imagens obtidas em repouso e imediatamente após o exercício devem ser comparadas lado a lado (usando o formato de tela quádrupla). No estresse farmacológico, as imagens no pico de estresse devem ser comparadas com os estágios de repouso, dose baixa e pré-pico ou de recuperação precoce (também utilizando o formato de tela quádrupla). 

 As imagens ecocardiográficas são geralmente realizadas a partir das incidências paraesternal de eixo longo e curto, e apical de duas e quatro câmaras. Em alguns casos, podem ser utilizados os planos subcostal e apical de eixo longo. Outras incidências e manobras poderão ser necessárias de acordo com a patologia investigada. 

 A viabilidade é indicada pelo desenvolvimento de espessamento miocárdico ao estresse em segmentos acentuadamente hipocinéticos e acinéticos no repouso. Para a adequada determinação da viabilidade, é necessária observação cuidadosa do espessamento miocárdico desde níveis menores de estresse e evitar que ocorra um rápido aumento da FC com isquemia associada. 

 Alterações anormais da função diastólica podem ocorrer antes das anormalidades da motilidade sistólica da parede. Quando a detecção de isquemia é o objetivo primário do exame, recomenda-se registrar os parâmetros diastólicos próximo ao pico do esforço ou após a avaliação do movimento da parede. 

 A avaliação com Doppler em cores da regurgitação mitral na EcoB e durante o pico do esforço pode permitir a detecção de regurgitação mitral isquêmica. 

 O EcoE tem acurácia semelhante à da tomografia por emissão de pósitrons na detecção de disfunção reversível em pacientes com miocárdio hibernante. 

**1.2.1.1. Metodologia do Estresse Físico ^8,1123^**

 O estresse físico pode ser realizado empregando-se esteira ou cicloergômetro, de acordo com protocolos convencionais para o TE. Na esteira, o protocolo mais empregado é o de Bruce, com as imagens ecocardiográficas sendo adquiridas em repouso e imediatamente após o término do esforço. 

**Figura 35:**
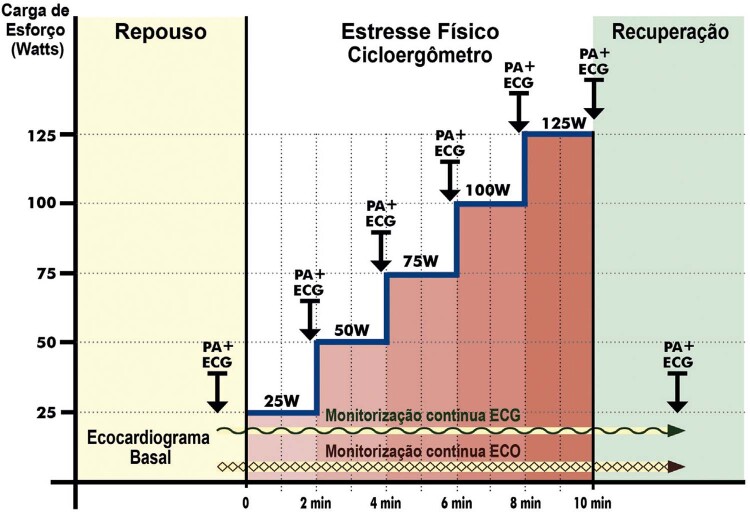
– Representação do ecocardiograma sob estresse físico em cicloergômetro. PA: medida da pressão arterial sistémica; ECG: registro do eletrocardiograma; ECO: ecocardiograma; min: minuto.

 No cicloergômetro o paciente pode manter-se em posição vertical (em bicicleta ergométrica) ou em posição supina (cicloergômetro de maca/maca ergométrica). No cicloergômetro de maca com rotação lateral, o paciente pedala em posição inclinada, permitindo maior adaptação do paciente e obtenção de imagens ecocardiográficas de melhor qualidade. O protocolo de esforço mais utilizado (protocolo de Astrand) inicia-se com a carga de 25W com aumento progressivo de 25W a cada 2 ou 3 minutos ( Figura 35 ). 

 A preparação da pele e o local de colocação dos eletrodos para monitorização eletrocardiográfica são os mesmos padronizados para realização do TE. As derivações poderão ser levemente deslocadas (para cima e para baixo), caso estejam interferindo nas janelas acústicas necessárias à realização do ecocardiograma. 

 Deve-se registrar um ECG de 12 derivações em repouso e a cada 2 minutos (ou estágio do esforço) ao longo do exame (esforço e recuperação). Uma derivação do ECG deverá ser exibida continuamente no monitor do ecocardiógrafo para fornecer referência sobre alterações do segmento ST e arritmias. 

 A pressão arterial deverá ser medida em repouso, a cada estágio do protocolo de esforço e na recuperação. 

 A análise e a interpretação dos dados quanto a FC, PA, escores (pré e pós-teste), registros eletrocardiográficos e aptidão cardiorrespiratória são similares às realizadas no TE. Recomenda-se a correlação desses dados aos achados ecocardiográficos. 

 Embora alguns serviços encerrem o esforço ao se atingir 85% da FC máxima prevista para a idade, recomenda-se a continuidade do esforço até o desenvolvimento de exaustão física (Escala de Borg ≥18) ou que surjam critérios absolutos para a interrupção. Atingir a FC máxima prevista aumenta a sensibilidade do exame e pode revelar anormalidades que ocorreriam apenas com alta carga de trabalho. ^8,260,1123^

**1.2.1.2. Metodologia do Estresse Farmacológico**

**1.2.1.2.1. Dobutamina ^260,1123^**

 O estresse farmacológico com dobutamina (ou dobutamina associada à atropina) é a modalidade alternativa mais utilizada para avaliação de isquemia miocárdica quando o paciente não consegue se exercitar. ^1124^

 A dobutamina geralmente é administrada em doses graduadas, começando em 5 μg.kg ^-1^ .min ^-1^ , com aumentos em intervalos de 3 minutos para 10, 20, 30 e 40 μg.kg ^-1^ .min ^-1^ . As doses de 20 ou 30 μg.kg ^-1^ .min ^-1^ de dobutamina geralmente permitem alcançar a FC alvo (85% da FC máxima prevista para a idade) mais rapidamente e com menos efeitos colaterais, principalmente se a FC não estiver aumentando conforme o esperado. 

 O uso de dobutamina em baixa dose inicial (2,5 μg.kg ^-1^ .min ^-1^ ) pode facilitar o reconhecimento da viabilidade miocárdica em segmentos anormais no EcoB. 

 Em pacientes betabloqueados e/ou nos quais não se atingiu a FC máxima prevista, ocorre redução da sensibilidade do EcoE na detecção de isquemia. Nesses casos, a atropina pode ser administrada visando atingir a FC, utilizando doses de 0,25 a 0,50 mg, em intervalos de 1 minuto, conforme necessário (dose total máxima de 2,0 mg). Recomenda-se utilizar a menor dosagem possível para evitar efeitos colaterais, incluindo toxicidade do sistema nervoso central. ^1125^

 Nos idosos, pacientes com pequeno porte corporal e naqueles que já estão próximos da FC máxima prevista, recomenda-se o uso de atropina em dose de 0,25 mg. Também é recomendada uma dose total <1,0 mg em pacientes neuropsiquiátricos, hepatopatas ou com índice de massa corporal inferior a 24 kg/m ^2^ . 

 Os desfechos para o término de estresse farmacológico incluem atingir a FC alvo, hipotensão, anormalidades da movimentação de parede do VE (novas ou agravadas), arritmias complexas, hipertensão grave e sintomas intoleráveis. 

**1.2.1.2.2. Vasodilatadores ^260,1123^**

 Testes de estresse com vasodilatadores (dipiridamol ou adenosina) induzem ao aumento do fluxo coronariano e são utilizados preferencialmente para avaliação de perfusão miocárdica. ^1126^ Também podem ser realizados para avaliação de isquemia e viabilidade miocárdica. A EcoE com vasodilatador tem menor sensibilidade para detecção de DAC do que quando realizada com estresse físico e/ou dobutamina-atropina. 

 Os agentes vasodilatadores são contraindicados em pacientes com obstrução reativa das vias aéreas, síndrome coronariana aguda instável ou complicada, arritmias cardíacas graves (TV e BAVT) ou hipotensão grave. 

 O dipiridamol geralmente é administrado em doses de até 0,84 mg/kg, durante 6 a 10 minutos. A administração de atropina ou realização de exercício de preensão manual no pico de infusão aumenta a sensibilidade do exame. ^1127^

 A adenosina em conjunto com ecocardiografia com contraste também pode ser empregada para avaliação de perfusão miocárdica. A taxa de infusão de adenosina é de 140 µg/kg/min, durante 4 a 6 minutos. A adenosina tem uma meia-vida mais curta e, portanto, tempo de ação mais curto que o dipiridamol. 

**1.2.1.3. Agentes de Realce Ultrassonográfico**

 Existem três agentes de realce ultrassonográfico com microbolhas comercialmente disponíveis no mundo ( *Sonovue* ^®^ , *Definity* ^®^ e *Optison* ^®^ ), utilizados para melhorar o delineamento da borda endocárdica, a qualidade das imagens e a detecção de DAC pelo EcoE. A administração (seja por *bolus* ou infusão contínua) visa opacificar toda a cavidade do VE, sem artefatos de redemoinho no ápice ou atenuação dos segmentos basais por sombra acústica. Podem ser, ainda, utilizados como marcadores de perfusão miocárdica. 

 O uso desses agentes em adultos é seguro, sendo muito rara a ocorrência de reações anafiláticas, mas não é recomendado em gestantes. Os protocolos específicos de administração encontram-se nas diretrizes da ASE de 2018. ^1128^

**Tabela 27:** – Duke database – Estimativa de risco para probabilidade pré-teste de DAC obstrutiva de acordo com classificação de angina 322

Idade (anos)	Sem angina		Angina atípica		Angina típica	
	Homens	Mulheres	Homens	Mulheres	Homens	Mulheres
35	3 a 35	1 a 19	8 a 59	2 a 39	30 a 88	10 a 78
45	9 a 47	2 a 22	21 a 70	5 a 43	51 a 92	20 a 79
55	23 a 59	4 a 21	45 a 79	10 a 47	80 a 95	38 a 82
65	49 a 69	9 a 29	71 a 86	20 a 51	93 a 97	56 a 84
* O intervalo apresenta probabilidade de DAC que varia de pacientes de baixo risco (sem diabetes mellitus, não tabagistas ou dislipidemia) até pacientes de alto risco. *						

**Tabela 43:** – Valores de referência do VO 2 pico, pulso de oxigênio e curva VE/VCO 2 no TCPE

Variáveis	Idade	Homens	Mulheres
** VO _**2**_ pico (mL/min) **	20-29	3,250-2,970	2,000-1,840
	30-39	2,950-2,690	1,820-1,660
	40-49	2,670-2,400	1,640-1,490
	50-59	2,380-2,130	1,470-1,320
	60-69	2,110-1,840	1,300-1,140
	70-80	1,820-1,570	1,120-0,940
**Pulso de oxigênio (mL/bpm)**	20-29	16,2-15,6	10,0-9,6
	30-39	15,5-14,9	9,6-9,2
	40-49	14,8-14,1	9,1-8,7
	50-59	14,0-13,2	8,6-8,2
	60-69	13,1-12,2	8,1-7,5
	70-80	12,1-11,1	7,4-6,7
** Relação VE/VCO _**2**_**	20-39	23,4-25,7	26,8-28,3
	40-59	25,8-28,1	28,4-29,9
	60-80	28,2-30,6	30,0-31,6
* Adaptada de Mezzani A. et al. ^*229*^ “Cardiopulmonary Exercise Testing: Basics of Methodology and Measurements”. *			

**Tabela 44:** – Valores de referência e interpretação das principais variáveis do TCPE

Variáveis	Normal	Alterado/comprometimento		
		Leve	Moderado	Grave
** %VO _**2**_ pico previsto **	≥100%	75-99%	50-74%	<50%
** %VO _**2**_ pico previsto no LV1 **	40-80%	<40%		
** Relação VE/VCO _**2**_**	<30	30-35,9	36-45	>45
**Pulso de oxigênio no esforço**	Aumento	Platô precoce ou diminuição*		
**Reserva respiratória**	≥30%	<30%**		
**EOV**	Ausente	Presente		
** VO _**2**_ LV1 **	≥40% VO _2_ pico previsto ou 40-60% VO _2_ pico medido	<40% VO _2_ pico previsto ou do VO _2_ pico medido		
** PETCO _**2**_ LV1 **	Aumento de 3 a 8 mmHg em relação ao valor de repouso	Aumento <3 ou >8 mmHg em relação ao valor de repouso		
EOV: ventilação oscilatória ao esforço; VCO _*2*_ : produção de CO _*2*_ ; VE: ventilação pulmonar; VO _*2*_ : consumo de oxigênio; LV1: 1 ***º*** limiar ventilatório. PETCO _*2*_ : pressão parcial expiratória final de dióxido de carbono. *Platô antes do VO _*2*_ máximo. ** Em caso de doença pulmonar. Adaptada de: Marcadet DM et al. ^*4*^ “French Society of Cardiology guidelines on exercise tests (part 1): Methods and interpretation” e de Mezzani A, et al. ^*229 *^ “Cardiopulmonary Exercise Testing: Basics of Methodology and Measurements”.				

## *Material suplementar

Para informação adicional, por favor, clique aqui


