# Supplementary material for: Diretriz Brasileira de Ergometria em População Adulta – 2024
Source: Arq Bras Cardiol. 2024 Feb 23;121(3):e20240110. [Article in Portuguese] doi: 10.36660/abc.20240110 (PMC11656589; doi:10.36660/abc.20240110)
Supplement: Supplementary file 1 [file 0066-782X-abc-121-3-e20240110-suppl01.pdf]

ANEXOS

Anexo 1 – Principais leis e resoluções pertinentes ao TE e TCPE

Aspectos legais	Referência
• O médico guardará sigilo a respeito das informações de que detenha conhecimento no desempenho de suas funções, com exceção dos casos previstos em lei<br>• É vedado:<br>– Delegar a outros profissionais atos ou atribuições exclusivas da profissão médica<br>– Deixar de assumir responsabilidade sobre procedimento médico que indicou ou do qual participou, mesmo quando vários médicos tenham assistido o paciente<br>– Acumpliciar-se com os que exercem ilegalmente a medicina ou com profissionais ou instituições médicas nas quais se pratiquem atos ilícitos<br>– Deixar de obter consentimento do paciente ou de seu representante legal após esclarecê-lo sobre o procedimento a ser realizado, salvo em caso de risco iminente de morte<br>– Deixar de garantir ao paciente o exercício do direito de decidir livremente sobre sua pessoa ou seu bem-estar, bem como exercer sua autoridade para limitá-lo<br>– Deixar de elaborar prontuário legível para cada paciente	Código de Ética Médica – Resolução Nº 2.217/2018 do CFM[1129]
• Determina que para a Área de Atuação em Ergometria, é necessário: ter formação de 1 ano; ter concluído Residência Médica em Cardiologia para realizar a formação; após formação, realizar concurso da AMB/Sociedade Brasileira de Cardiologia para obtenção do Título de Atuação; pré-requisito para o concurso, além da formação, ter o Título de Especialista em Cardiologia da AMB	Resolução Nº 2.221/2018 do CFM/Portaria CME Nº 1/2018[286]
• O TE deve ser individualizado e realizado, em todas as suas etapas, por médico habilitado e capacitado para atender a emergências cardiovasculares, tornando imprescindível, para tal, sua presença física na sala<br>• Por ser ato médico privativo, caracteriza-se como falta ética a delegação para outros profissionais da realização do TE<br>• As condições adequadas para a realização do TE estão previstas no Manual de Fiscalização do CFM	Resolução Nº 2.021/13 do CFM[287]
• Critérios norteadores da propaganda em medicina, conceituando os anúncios, a divulgação de assuntos médicos, o sensacionalismo, a autopromoção e as proibições referentes à matéria	Resolução Nº 2.336/2023 do CFM[1130]
• Garantir a privacidade e a confidencialidade dos dados e informações armazenadas digitalmente dos pacientes; organizar bancos de dados seguros e confiáveis; garantir a transmissão de dados e informações em segurança; fazer cópia de segurança na medida da possibilidade	Resolução Nº 1.821/2007 do CFM[282]
• Art. 186. Aquele que, por ação ou omissão voluntária, negligência ou imprudência, violar direito e causar dano a outrem, ainda que exclusivamente moral, comete ato ilícito	Código Civil Brasileiro – Lei 10.406 de 2002[1131]
• Capítulo III, Art. 6º - São direitos básicos do consumidor:<br>I – A proteção da vida, saúde e segurança contra os riscos provocados por práticas no fornecimento de produtos e serviços considerados perigosos ou nocivos<br>II – A educação e divulgação sobre o consumo adequado dos produtos e serviços, asseguradas a liberdade de escolha e a igualdade nas contratações<br>III – A informação adequada e clara sobre os diferentes produtos e serviços, com especificação correta de quantidade, características, composição, qualidade, tributos incidentes e preço, bem como sobre os riscos que apresentem	Código de Proteção ao Consumidor<br>Direitos Básicos do Consumidor - Lei Nº 8.078 de 11 de setembro de 1990[1132]

CFM: Conselho Federal de Medicina.

Anexo 2 – Fórmulas para cálculo do VO_2pico previsto[293,343-348]

Denominação	Equação para cálculo do VO_2pico previsto
**Fórmulas clássicas:**	
**Algoritmo de Wasserman**	**Homens (mL/min):**<br>1) Calcular peso normal (kg) = 0,79 × altura (cm) – 60,7<br>2) De acordo com avaliação da categoria do peso, utilizar uma das fórmulas:<br>– Se peso real (kg) = peso normal:<br>VO_2 = peso real × [50,72 – 0,372 × idade (anos)]<br>– Se peso real < peso normal (baixo peso):<br>VO_2 = [(peso normal + peso real)/2] × [50,72 – 0,372 × idade (anos)]<br>– Se peso real > peso normal (sobrepeso):<br>VO_2 = peso normal × [50,72 – 0,372 × idade (anos)] + [6 × (peso real – peso normal)]<br><br>**Mulheres (mL/min):**<br>1) Calcular peso normal = 0,65 × altura (cm) – 42,8<br>2) De acordo com avaliação da categoria do peso, utilizar uma das fórmulas:<br>– Se peso real (kg) = peso normal<br>VO_2 = (peso real + 43) × [22,78 – 0,17 × idade (anos)]<br>– Se peso real < peso normal (baixo peso)<br>VO_2 = [(peso normal + peso real + 86)/2] × [22,78 – 0,17 × idade (anos)]<br>– Se peso real > peso normal (sobrepeso)<br>VO_2 = (peso normal + 43) × [22,78 – 0,17 × idade (anos)] + [6 × (peso real – peso normal)]<br><br>**Se esteira** (mL/min)**: multiplicar resultado por 1,11**
**Equação de Jones**	**Cicloergômetro** (mL/min)<br>**Homens:** 4,2 – [0,032 × idade (anos)]<br>**Mulheres:** 2,6 – [0,014 × idade (anos)]<br><br>**Cicloergômetro** (mL/min)<br>[0,046 × altura (cm)] – [0,021 × idade (anos)] – [0,624 × sexo (homem = 0; mulher = 1)] – 4,31<br><br>**Esteira** (mL/kg/min)<br>**Homens:** [60 – (0,55 × idade)] × 1,11<br>**Mulheres:** [48 – (0,37 × idade)] × 1,11
**Veterans Affairs cohort**	**Homens** (MET): 18 – 0,15 × idade
**St. James Women Take Heart project**	**Mulheres** (MET): 14,7 – 0,13 × idade
**Novas Fórmulas:**	
**FRIEND registry (esteira)**	**79,9 – [0,39 × idade (anos)] – [13,7 × sexo (homem = 0; mulher = 1)] – [0,127 × peso (em libras)]**
**FRIEND registry (esteira ou cicloergômetro)**	45,2 – [0,35 × idade (anos)] – [10,9 × sexo (homem = 1; mulher = 2] – [0,15 × peso (libras)] + [0,68 × altura (em polegadas)] – [0,46 × modo de esforço (esteira = 1; cicloergômetro = 2)]
**Almeida et al. (esteira)**	53,478 + [-7,518 × sexo (homem = 1; mulher = 2)] + [-0,254 × idade (anos)] + [-0,430 × IMC] + [6,132 × atividade física (sedentário = 1; ativo = 2; atleta = 3)]
**Rossi Neto et al. (esteira)**	20,89706 + [11,19284 × sexo (homem = 1; mulher = 0)] – [0,20764 × idade (anos)] – [0,38435 × peso (kg)] + [28,14593 × altura (m)]
**Milani et al. (esteira)**	**Homem:** 37,09 + (0,4129 × idade) - (0,007798 × $idade^2$)<br>**Mulher:** 33,51 + (0,1242 × idade) - (0,004551 × $idade^2$)<br>**Idade em anos**

VO_2: consumo de oxigênio; IMC: índice de massa corporal; conversão: 1 kg = 2,2046 libras; 1 cm = 0,39370 polegadas.

Anexo 3 – Principais bebidas, alimentos e medicamentos que contêm cafeína

Cafés	Petiscos cafeinados
• Café	• Bolacha de chocolate
• Expresso	• Alguns tipos de batata chips
• Café Mocha	• Algumas balas e gomas
• Café Descafeinado	**Sorvetes**
**Chás - em geral**	• Starbucks ice cream café
• Chá preto	• Sorvetes de café
• Iced Tea	• Sorvete de café Häagen-Dazs
• Chá verde	**Chocolates e bebidas**
• Lemon Iced Tea garrafa	• Chocolate quente
• Lipton chá descafeinado (preto ou verde)	• Barra de chocolate
**Refrigerantes e Sucos**	• Barra de chocolate ao leite
• Pepsi	**Medicamentos**
• Coca-Cola, Coca Zero ou Diet Pepsi	• Tylenol DC
• Coca-Cola Plus	• Ormigrein
• Diet Coke	• Dipirona + cafeína
• Fanta, Sprite, 7-Up	• Neosaldina
• Guaraná	• Miorrelax
• Suco de acerola	• Miosan cafeína
**Energéticos – em geral**	• Dorflex
• Monster Energy	• Benegrip
• Red Bull	• Suplementos e Pílulas de cafeína
• Monster Energy	
• Fusion	
• TNT	

Nota: Os produtos e marcas listados são os mais frequentemente disponíveis no mercado. Os mesmos cuidados se aplicam a produtos similares brasileiros. Adaptado de: Henzlova MJ et al.,[1111] ASNC imaging guidelines for SPECT nuclear cardiology procedures: Stress, protocols, and tracers.
