# Supplementary material for: Diretriz Brasileira de Ergometria em População Adulta – 2024
Source: Arq Bras Cardiol. 2024 Feb 23;121(3):e20240110. [Article in Portuguese] doi: 10.36660/abc.20240110 (PMC11656589; doi:10.36660/abc.20240110)
Supplement: Supplementary file 2 [file 0066-782X-abc-121-3-e20240110-suppl01-en.pdf]

APPENDICES

Appendix 1 – Legal and regulatory framework applicable to ET and CPET in Brazil

Legal aspects	Reference
• The physician shall preserve the confidentiality of any information acquired in the performance of his or her duties, except when legally mandated otherwise<br>• The physician is forbidden from:<br>– Delegating to other providers acts or duties restricted to the medical profession<br>– Shirking responsibility for a medical procedure he or she indicated or in which he or she participated, even when the patient was assisted by several physicians<br>– Aiding and abetting those who practice medicine illegally or with medical professionals or facilities which engage in illicit activities<br>– Failing to obtain consent from the patient or his or her legal representative after explaining the procedure to be performed, except in case of imminent risk of death<br>– Failing to safeguard the patient's right to decide freely about his or her person or well-being, or utilizing his or her authority to violate this right<br>– Failing to keep a legible medical record for each patient	Brazilian Code of Medical Ethics (Código de Ética Médica), FMC Resolution No. 2217/2018[1129]
• Sets forth the requirements for Focused Practice Designation in Exercise Test: 1 year of training; having completed Medical Residency in Cardiology before such training; after training, take the Brazilian Medical Association/Brazilian Society of Cardiology board exam to obtain certification; as a prerequisite for sitting the aforementioned exam, in addition to training, holding a current Board Certification in Cardiology from the Brazilian Medical Association	FMC Resolution No. 2221/2018; Ordinance No. 1/2018[286]
• The ET must be individualized and carried out, at all stages, by a qualified physician who has been trained to respond to cardiovascular emergencies and must thus be physically present in the room at all times<br>• As ET is a medical procedure under the sole responsibility of the performing physician, delegating its performance to other providers is considered a violation of medical ethics<br>• The necessary and appropriate conditions for carrying out ET are listed in the FMC Inspection Manual	FMC Resolution No. 2021/13[287]
• Guiding criteria for advertising in medicine, conceptualizing advertisements, dissemination of medical matters, sensationalism, self-promotion, and prohibitions related thereto	FMC Resolution No. 2.336/2023[1130]
• Ensuring the privacy and confidentiality of patients' data and digitally stored information; organizing secure and reliable databases; ensuring the secure transmission of data and information; maintaining backup copies to the fullest possible extent	FMC Resolution No. 1821/2007[282]
• Art. 186. Anyone who, by willful action or inaction, negligence, or recklessness, violates a right and causes damage to others, even if exclusively moral, commits a wrongful act	Brazilian Civil Code – Law No. 10,406/2002[1131]
• Chapter III, Art. 6 – The following are basic consumer rights:<br>I – the protection of the consumer's life, health, and safety against any risks arising from any practices in the supply of products and services considered harmful or dangerous<br>II – education and information about the adequate consumption of products and services, ensuring freedom of choice and equality in transactions<br>III – adequate and clear information about different products and services, with correct specification of quantity, characteristics, composition, quality, price, and taxes, as well as the risks presented	Brazilian Consumer Protection Code.<br>Basic Consumer Rights – Law No. 8,078 of September 11, 1990[1132]

FMC: Brazilian Federal Medical Council.

Appendix 2 – Formulas for prediction of VO_2peak[293,343-348]

Name	Equation for VO_2peak prediction
**Classic formulas:**	
**Wasserman algorithm**	**Men Males (mL/min):**<br>1) Calculate normal weight (kg) = 0.79 × height (cm) – 60.7<br>2) According to weight classification, use one of the following equations:<br>– If actual weight (kg) = normal weight:<br>VO_2 = actual weight × [50.72 – 0.372 × age (years)]<br>– If actual weight < normal weight (underweight):<br>VO_2 = [(normal weight + actual weight)/2] × [50.72 – 0.372 × age (years)]<br>– If actual weight > normal weight (overweight):<br>VO_2 = normal weight × [50.72 – 0.372 × age (years)] + [6 × (actual weight – normal weight)]<br><br>**Women (mL/min):**<br>1) Calculate normal weight = 0.65 × height (cm) – 42.8<br>2) According to weight classification, use one of the following equations:<br>– If actual weight (kg) = normal weight<br>VO_2 = (actual weight + 43) × [22.78 – 0.17 × age (years)]<br>– If actual weight < normal weight (underweight)<br>VO_2 = [(normal weight + actual weight + 86)/2] × [22.78 – 0.17 × age (years)]<br>– If actual weight > normal weight (overweight)<br>VO_2 = (normal weight + 43) × [22.78 – 0.17 × age (years)] + [6 × (actual weight – normal weight)]<br><br>**Se esteira** (mL/min)**: multiplicar resultado por 1,11**
**Jones equation**	**Cycle ergometer** (L/min)<br>**Men:** 4.2 – [0.032 × age (years)]<br>**Women:** 2.6 – [0.014 × age (years)]<br><br>**Cycle ergometer** (L/min)<br>[0.046 × height (cm)] – [0.021 × age (years)] – [0.624 × sex (male = 0; female = 1)] – 4.31<br><br>**Treadmill** (mL/kg/min)<br>**Men:** [60 – (0.55 × age)] × 1.11<br>**Women:** [48 – (0.37 × age)] × 1.11
**Veterans Affairs cohort**	**Men** (MET): 18 – 0.15 × age
**St. James Women Take Heart project**	**Women** (MET): 14.7 – 0.13 × age
**New formulas:**	
**FRIEND registry (treadmill)**	**79.9 – [0.39 × age (years)] – [13.7 × sex (male = 0; female = 1)] – [0.127 × weight (lb)]**
**FRIEND registry (treadmill or cycle ergometer)**	45.2 – [0.35 × age (years)] – [10.9 × sex (male = 1; female = 2] – [0.15 × weight (lb)] + [0.68 × height (in)] – [0.46 × exercise mode (treadmill = 1; cycle ergometer = 2)]
**Almeida et al. (treadmill)**	53.478 + [-7.518 × sex (male = 1; female = 2)] + [-0.254 × age (years)] + [-0.430 × BMI] + [6.132 × level of physical activity (sedentary = 1; active = 2; athlete = 3)]
**Rossi Neto et al. (treadmill)**	20.89706 + [11.19284 × sex (male = 1; female = 0)] – [0.20764 × age (years)] – [0.38435 × weight (kg)] + [28.14593 × height (m)]
**Milani et al. (treadmill)**	**Men:** 37.09 + (0.4129 × age) – (0.007798 × age^2)<br>**Women:** 33.51 + (0.1242 × age) – (0.004551 × age^2)<br>**Age in years**

VO_2: oxygen consumption; BMI: body mass index; conversion formulas: 1 kg = 2.2046 pounds; 1 cm = 0.39370 inches.

Guidelines

Appendix 3 – Key caffeine-containing beverages, foods, and medications

**Coffees**
• Coffee
• Espresso
• Mocha
• Decaffeinated coffee
**Teas, general**
• Black tea
• Iced tea
• Green tea
• Lemon iced tea (bottled)
• Lipton Decaffeinated Tea (black or green)
**Soft drinks and juices**
• Pepsi
• Coca-Cola, Coke Zero, Diet Pepsi
• Coca-Cola Plus
• Diet Coke
• Fanta, Sprite, 7-Up
• Guaraná
• Acerola juice
**Energy drinks**
• Monster Energy
• Red Bull
• Monster Energy
• Fusion
• TNT
**Caffeinated snack foods**
• Chocolate cookies
• Some potato chips
• Some candies and gums
**Ice creams**
• Starbucks Frappuccino
• Coffee ice cream
• Häagen-Dazs coffee ice cream
**Cocoa and other beverages**
• Hot chocolate
• Candy bars
• Milk chocolate bars
**Drugs**
• Tylenol DC
• Ormigrein
• Metamizole/caffeine
• Neosaldina
• Miorrelax
• Miosan Caf
• Dorflex
• Benegrip
• Caffeinated supplements and caffeine pills

Note: Products and trademarks listed are the most common ones available on the market. The same precautions apply to similar Brazilian's products. Adapted from: Henzlova MJ et al.,[1111] ASNC imaging guidelines for SPECT nuclear cardiology procedures: Stress, protocols, and tracers.
