# Supplementary material for: Diretriz Brasileira de Ergometria em População Adulta – 2024
Source: Arq Bras Cardiol. 2024 Feb 23;121(3):e20240110. [Article in Portuguese] doi: 10.36660/abc.20240110 (PMC11656589; doi:10.36660/abc.20240110)
Supplement: Translation

# Translation: Brazilian Guideline for Exercise Test in the Adult Population – 2024

**DOI:** 10.36660/abc.20240110i

**Authors:** Tales de Carvalho, Odilon Gariglio Alvarenga de Freitas, William Azem Chalela, Carlos Alberto Cordeiro Hossri, Mauricio Milani, Susimeire Buglia, Dalton Bertolim Precoma, Andréa Maria Gomes Marinho Falcão, Luiz Eduardo Mastrocola, Iran Castro, Pedro Ferreira de Albuquerque, Ricardo Quental Coutinho, Fabio Sandoli de Brito, Josmar de Castro Alves, Salvador Manoel Serra, Mauro Augusto dos Santos, Clea Simone Sabino de Souza Colombo, Ricardo Stein, Artur Haddad Herdy, Anderson Donelli da Silveira, Claudia Lucia Barros de Castro, Miguel Morita Fernandes da Silva, Romeu Sergio Meneghello, Luiz Eduardo Fonteles Ritt, Felipe Lopes Malafaia, Leonardo Filipe Benedeti Marinucci, José Luiz Barros Pena, Antônio Eduardo Monteiro de Almeida, Marcelo Luiz Campos Vieira, Arnaldo Laffitte Stier

**Affiliations:** 1 Florianópolis SC Brasil Clínica de Prevenção e Reabilitação Cardiosport, Florianópolis , SC – Brasil; 2 Florianópolis SC Brasil Universidade do Estado de Santa Catarina, Florianópolis , SC – Brasil; 3 Belo Horizonte MG Brasil Minascor Centro Médico, Belo Horizonte , MG – Brasil; 4 São Paulo SP Brasil Instituto do Coração do Hospital das Clínicas da Faculdade de Medicina da Universidade de São Paulo (InCor-HCFMUSP), São Paulo , SP – Brasil; 5 São Paulo SP Brasil Hospital do Coração (HCOr), São Paulo , SP – Brasil; 6 São Paulo SP Brasil Instituto Dante Pazzanese de Cardiologia, São Paulo , SP – Brasil; 7 Brasília DF Brasil Universidade de Brasília (UnB), Brasília , DF , Brasil; 8 Hasselt Bélgica Hasselt University, Hasselt – Bélgica; 9 Hasselt Bélgica Jessa Ziekenhuis, Hasselt – Bélgica; 10 Campina Grande do Sul PR Brasil Sociedade Hospitalar Angelina Caron, Campina Grande do Sul , PR – Brasil; 11 Porto Alegre RS Brasil Instituto de Cardiologia do Rio Grande do Sul, Porto Alegre , RS – Brasil; 12 Maceió AL Brasil Hospital Arthur Ramos, Maceió , AL – Brasil; 13 Maceió AL Brasil Hospital do Coração Alagoano, Maceió , AL – Brasil; 14 Recife PE Brasil Universidade de Pernambuco (UPE), Recife , PE – Brasil; 15 São Paulo SP Brasil Hospital Sírio Libanês, São Paulo , SP – Brasil; 16 Natal RN Brasil Procardio Clínica Cardiológica, Natal , RN – Brasil; 17 Rio de Janeiro RJ Brasil Instituto Estadual de Cardiologia Aloysio de Castro (IECAC), Rio de Janeiro , RJ – Brasil; 18 Rio de Janeiro RJ Brasil Instituto Nacional de Cardiologia do Rio de Janeiro, Rio de Janeiro , RJ – Brasil; 19 Rio de Janeiro RJ Brasil Linkcare Saúde, Rio de Janeiro , RJ – Brasil; 20 Valinhos SP Brasil Faculdade São Leopoldo Mandic, Valinhos , SP – Brasil; 21 Porto Alegre RS Brasil Universidade Federal do Rio Grande do Sul (UFRGS), Porto Alegre , RS – Brasil; 22 Porto Alegre RS Brasil Hospital de Clínicas de Porto Alegre, Porto Alegre , RS – Brasil; 23 Porto Alegre RS Brasil Hospital Moinhos de Vento, Porto Alegre, RS Porto Alegre , RS – Brasil; 24 Rio de Janeiro RJ Brasil Universidade Federal do Rio de Janeiro (UFRJ), Rio de Janeiro , RJ – Brasil; 25 Rio de Janeiro RJ Brasil CLINIMEX – Clínica de Medicina de Exercício, Rio de Janeiro , RJ – Brasil; 26 Curitiba PR Brasil Universidade Federal do Paraná (UFPR), Curitiba , PR – Brasil; 27 Salvador BA Brasil Escola Bahiana de Medicina e Saúde Pública, Salvador , BA – Brasil; 28 Salvador BA Brasil Instituto D’Or de Pesquisa e Ensino, Salvador , BA – Brasil; 29 Salvador BA Brasil Hospital Cárdio Pulmonar, Salvador , BA – Brasil; 30 São Paulo SP Brasil Hospital Samaritano Paulista, São Paulo , SP – Brasil; 31 São Paulo SP Brasil UnitedHealth Group Brasil, São Paulo , SP – Brasil; 32 Belo Horizonte MG Brasil Faculdade Ciências Médicas de Minas Gerais, Belo Horizonte , MG – Brasil; 33 Belo Horizonte MG Brasil Hospital Felício Rocho, Belo Horizonte , MG – Brasil; 34 João Pessoa PB Brasil Cardiológica Métodos Gráficos, João Pessoa , PB – Brasil; 35 São Paulo SP Brasil Hospital Israelita Albert Einstein, São Paulo , SP – Brasil; 36 Curitiba PR Brasil Secretaria Municipal de Saúde Curitiba, Curitiba , PR – Brasil

Class of Recommendation

**Class I:** Conclusive evidence, or, failing that, general consensus that the procedure is safe and useful/effective.

**Class II:** Conflicting evidence and/or divergence of opinion about the procedure’s safety and usefulness/effectiveness.

**Class IIa:** Evidence/opinion is in favor of the procedure. Most approve of it.

**Class IIb:** Safety and usefulness/effectiveness less well established, with divergence of opinions.

**Class III:** Evidence and/or consensus that the procedure is not useful/effective and, in some cases, is harmful.

Level of Evidence

**Level A:** Data obtained from multiple large randomized studies, concordant and/or robust meta-analysis of randomized clinical studies.

**Level B:** Data derived from less robust meta-analyses, from a single randomized study or from non-randomized (observational) studies.

**Level C:** Data obtained from expert consensus.

**Table:** 

Brazilian Guideline for Exercise Test in the Adult Population – 2024	
The report below lists declarations of interest as reported to the SBC by the experts during the period of the developmentt of these statement, 2022/2023.	
**Expert**	**Type of relationship with industry**
Anderson Donelli da Silveira	Nothing to be declared
Andréa Maria Gomes Marinho Falcão	Nothing to be declared
Antonio Eduardo Monteiro de Almeida	Nothing to be declared
Arnaldo Laffitte Stier Junior	Financial declaration A - Economically relevant payments of any kind made to (i) you, (ii) your spouse/partner or any other person living with you, (iii) any legal person in which any of these is either a direct or indirect controlling owner, business partner, shareholder or participant; any payments received for lectures, lessons, training instruction, compensation, fees paid for participation in advisory boards, investigative boards or other committees, etc. From the brazilian or international pharmaceutical, orthosis, prosthesis, equipment and implants industry: - Libbs: continuing education, Ebatz, Vatis. Other relationships Any economically relevant equity interest in companies in the healthcare or education industry or in any companies competing with or supplying to SBC: - Quanta Diagnóstico Curitiba.
Artur Haddad Herdy	Nothing to be declared
Carlos Alberto Cordeiro Hossri	Nothing to be declared
Claudia Lucia Barros de Castro	Nothing to be declared
Clea Simone Sabino de Souza Colombo	Nothing to be declared
Dalton Bertolim Precoma	Financial declaration A - Economically relevant payments of any kind made to (i) you, (ii) your spouse/partner or any other person living with you, (iii) any legal person in which any of these is either a direct or indirect controlling owner, business partner, shareholder or participant; any payments received for lectures, lessons, training instruction, compensation, fees paid for participation in advisory boards, investigative boards or other committees, etc. From the brazilian or international pharmaceutical, orthosis, prosthesis, equipment and implants industry: - Novonordisk: Ozempic; Daiichi-Sankyo: Lixiana; Servier: Vastarel; Astrazeneca: Forxiga. B - Research funding under your direct/personal responsibility (directed to the department or institution) from the Brazilian or international pharmaceutical, orthosis, prosthesis, equipment and implants industry: - Bayer: anticoagulant; Janssen: anticoagulant; Novonordisk: cardiometabolism; Astrazeneca: heart failure, hypercalcemia, diastolic dysfunction; Daiichi-Sankyo: anticoagulant; Cardiol: COVID-19 e myocarditis; Servier: chronic coronary artery disease. Other relationships Funding of continuing medical education activities, including travel, accommodation and registration in conferences and courses, from the brazilian or international pharmaceutical, orthosis, prosthesis, equipment and implants industry: - Novonordisk: cardiometabolism; Daiichi-Sankyo: anticoagulant; Servier: chronic coronary artery disease; Torrent: dyslipidemia. Any economically relevant equity interest in companies in the healthcare or education industry or in any companies competing with or supplying to SBC: - Health Area: nuclear medicine.
Fabio Sandoli de Brito	Nothing to be declared
Felipe Lopes Malafaia	Nothing to be declared
Iran Castro	Nothing to be declared
José Luiz Barros Pena	Nothing to be declared
Josmar de Castro Alves	Nothing to be declared
Leonardo Filipe Benedeti Marinucci	Nothing to be declared
Luiz Eduardo Fonteles Ritt	Financial declaration A - Economically relevant payments of any kind made to (i) you, (ii) your spouse/partner or any other person living with you, (iii) any legal person in which any of these is either a direct or indirect controlling owner, business partner, shareholder or participant; any payments received for lectures, lessons, training instruction, compensation, fees paid for participation in advisory boards, investigative boards or other committees, etc. From the brazilian or international pharmaceutical, orthosis, prosthesis, equipment and implants industry: - Boeringher Lilly: Jardiance; Novonordis: pesquisador em estudos; Astrazeneca; Novartis; Bayer; Bristol; Pfizer. B - Research funding under your direct/personal responsibility (directed to the department or institution) from the Brazilian or international pharmaceutical, orthosis, prosthesis, equipment and implants industry: - MDI Medical. Other relationships Funding of continuing medical education activities, including travel, accommodation and registration in conferences and courses, from the brazilian or international pharmaceutical, orthosis, prosthesis, equipment and implants industry: - Novo Nordisk: Ozempic.
Luiz Eduardo Mastrocola	Nothing to be declared
Marcelo Luiz Campos Vieira	Nothing to be declared
Mauricio Milani	Nothing to be declared
Mauro Augusto dos Santos	Nothing to be declared
Miguel Morita Fernandes da Silva	Financial declaration A - Economically relevant payments of any kind made to (i) you, (ii) your spouse/partner or any other person living with you, (iii) any legal person in which any of these is either a direct or indirect controlling owner, business partner, shareholder or participant; any payments received for lectures, lessons, training instruction, compensation, fees paid for participation in advisory boards, investigative boards or other committees, etc. From the brazilian or international pharmaceutical, orthosis, prosthesis, equipment and implants industry: - Novartis: Entresto; Bayer: Firialta; Astrazeneca: Forxiga; Boehringer: Jardiance. B - Research funding under your direct/personal responsibility (directed to the department or institution) from the Brazilian or international pharmaceutical, orthosis, prosthesis, equipment and implants industry: - Novartis; Bayer.
Odilon Gariglio Alvarenga de Freitas	Nothing to be declared
Pedro Ferreira de Albuquerque	Financial declaration A - Economically relevant payments of any kind made to (i) you, (ii) your spouse/partner or any other person living with you, (iii) any legal person in which any of these is either a direct or indirect controlling owner, business partner, shareholder or participant; any payments received for lectures, lessons, training instruction, compensation, fees paid for participation in advisory boards, investigative boards or other committees, etc. From the brazilian or international pharmaceutical, orthosis, prosthesis, equipment and implants industry: - MMedicine Cursos: Ergometry class. Other relationships Employment relationship with the brazilian or international pharmaceutical, orthosis, prosthesis, equipment and implants industry, as well as any employment relationship with health insurance companies or medical audit companies (including part-time jobs) in the year to which your declaration refers: - Cooperated Partner at Unimed Maceió Alagoas.
Ricardo Quental Coutinho	Nothing to be declared
Ricardo Stein	Other relationships Funding of continuing medical education activities, including travel, accommodation and registration in conferences and courses, from the brazilian or international pharmaceutical, orthosis, prosthesis, equipment and implants industry: - Life Genomics
Salvador Manoel Serra	Nothing to be declared
Susimeire Buglia	Nothing to be declared
Tales de Carvalho	Nothing to be declared
William Azem Chalela	Nothing to be declared

## Table of Contents

Part 1 – Indications, Legal Aspects, and Training in Exercise Test 08

1. Introduction 08

2. Indications and Contraindications for ET, CPET, and Cardiac Stress Imaging 08

2.1. General Indications for ET 08

2.2. Specific Clinical Indications for ET 09

**2.2.1. Indications for ET in Coronary Artery Disease** 09

**2.2.2. Indications for ET in Asymptomatic Patients** 09

**2.2.3. Indications for ET in Athletes** 09

**2.2.4. Indications for ET in Hypertension** 09

**2.2.5. Indications for ET in Valvular Heart Disease** 09

**2.2.6. Indications for ET in Heart Failure and Cardiomyopathies** 11

**2.2.7. Indications for ET in the Context of Arrhythmias and Conduction Disorders** 11

**2.2.8. Indications for ET in Other Clinical Conditions** 11

2.3. Relative and Absolute Contraindications 11

**2.3.1. Relative Contraindications to ET/CPET** 12

**2.3.2. Absolute Contraindications to ET/CPET** 12

2.4. Indications for CPET 12

**2.4.1. General Indications for CPET** 12

**2.4.2. Specific Clinical Indications for CPET** 12

2.5. Indications for Cardiac Stress Imaging 12

**2.5.1. Myocardial Perfusion Imaging** 12

**2.5.2. Indications for Stress Echocardiography** 13

3. Legal Aspects and Essential Conditions for Performing ET, CPET, and Cardiac Stress Imaging 15

3.1. Legal Aspects Involved in the Practice of ET and CPET 15

3.2. Essential Conditions for Performing ET and CPET 15

3.3. Informed Consent Forms for ET/CPET 18

3.4. Informed Consent Forms for Cardiac Stress Imaging 18

4. Aspects Related to Training in Exercise Test as an Area of Focused Practice 18

Part 2 – The Exercise Test 21

1. ET methodology 21

1.1. Core Conditions to Perform ET 22

**1.1.1. Team** 22

**1.1.2. Physical Infrastructure** 23

**1.1.3. Equipment** 23

**1.1.4. Emergency Supplies** 23

**1.1.5. Emergency Drugs** 23

**1.1.6. Orientations for Patients when Scheduling ET** 23

1.2. Basic ET Procedure 24

**1.2.1. Pre-test Phase** 24

**1.2.2. Initial Assessment** 24

**1.2.3. Targeted and Specific Physical Examination** 24

**1.2.4. Electrocardiographic Monitoring and Recording System** 24

**1.2.4.1. Three-lead Systems** 25

**1.2.4.2. Twelve-lead Systems** 25

**1.2.4.3. Placements Including 13 or More Leads** 27

**1.2.4.4. Skin Preparation for ECG Monitoring** 27

**1.2.4.5. ECG Recording** 27

**1.2.5. Hemodynamic Monitoring** 27

**1.2.5.1. Heart Rate Monitoring** 27

**1.2.5.2. Blood Pressure Monitoring** 27

**1.2.6. Monitoring for Signs and Symptoms** 28

**1.2.7. Prevention of ET Complications** 28

1.3. Ergometers 28

**1.3.1. Cycle Ergometer** 28

**1.3.2. Treadmill** 28

**1.3.3. Upper-Body Ergometer** 28

**1.3.4. Other Ergometers** 28

1.4. Choice of Protocol 29

**1.4.1. Cycle Ergometer Protocols** 29

**1.4.2. Treadmill Protocols** 29

**1.4.2.1. Step Protocols** 29

**1.4.2.1.1. Bruce Protocol** 29

**1.4.2.1.2. Modified Bruce Protocol** 29

**1.4.2.1.3. Ellestad Protocol** 29

**1.4.2.1.4. Naughton Protocol** 30

**1.4.2.2. Ramp Protocol** 30

**1.4.3. Upper-Body Ergometer Protocol** 30

**1.4.4. Test Cessation/Termination** 30

2. Test Accuracy, Pretest Probability, and Pretest Scores 31

2.1. Pretest Probability of CAD 31

2.2. Sensitivity, Specificity, and Predictive Value 31

2.3. Pretest CVD Scores and Risk Factors 32

3. Clinical and Hemodynamic Responses to Exercise in the Adult Population 32

3.1. Clinical Responses 32

**3.1.1. Exercise Tolerance** 32

**3.1.2. Cardiorespiratory Fitness/Functional Classification** 33

**3.1.3. Symptoms** 34

**3.1.4. Inspection and Auscultation** 34

3.2. Hemodynamic Responses 35

**3.2.1. Heart Rate** 35

**3.2.1.1. Resting Heart Rate** 35

**3.2.1.2. Chronotropic Response** 36

**3.2.2. Blood Pressure Response** 36

**3.2.3. Double Product** 39

4. ECG Responses 41

4.1. P Wave 41

**4.1.1. Normal Responses** 42

**4.1.2. Abnormal Responses** 42

4.2. PR Interval/Segment 43

**4.2.1. Normal Responses** 43

**4.2.2. Abnormal Responses** 43

4.3. Q Wave 43

**4.3.1. Normal Responses** 43

**4.3.2. Abnormal Responses** 43

4.4. R Wave 43

**4.4.1. Normal Responses** 43

**4.4.2. Abnormal Responses** 43

4.5. S Wave 43

**4.5.1. Normal Responses** 44

**4.5.2. Abnormal Responses** 44

4.6. QRS Duration 44

**4.6.1. Normal Responses** 44

**4.6.2. Abnormal Responses** 44

4.7. High-frequency Assessment of QRS Fragmentation 44

**4.7.1. Normal Responses** 44

**4.7.2. Abnormal Responses** 44

4.8. T Wave 45

**4.8.1. Normal Responses** 45

**4.8.2. Abnormal Responses** 45

4.9. U Wave 45

**4.9.1. Normal Responses** 45

**4.9.2. Abnormal Responses** 45

4.10. Early Repolarization 46

4.11. ST-segment Elevation 46

4.12. J Point and Upsloping Depression 47

4.13. Slow Upsloping, Horizontal, and Downsloping ST-segment Depression 47

**4.13.1. ST Hump Sign** 48

4.14. Normalization of ST-segment Changes 49

4.15. ST/HR Slope, ST/HR Index, ST/HR Loop, and ST/HR Hysteresis 49

**4.15.1. ST/HR Slope** 49

**4.15.2. ST/HR Index** 49

**4.15.3. ST/HR Loop** 49

**4.15.4. ST/HR Hysteresis** 50

4.16. QT Interval, QTc, QT Hysteresis, and QT Dispersion 50

**4.16.1. Abnormal Responses** 50

4.17. Disorders of Atrioventricular Conduction, Intraventricular Conduction, and Impulse Formation 51

**4.17.1. Atrioventricular Conduction Disorders** 51

**4.17.1.1. First-degree Atrioventricular (AV) Block** 51

**4.17.1.2. Type I Second-degree AV Block (Mobitz I)** 52

**4.17.1.3. Type II Second-degree AV Block (Mobitz II)** 52

**4.17.1.4. Type 2:1 AV Block/Advanced or High-grade AV Block/Third-degree AV Block or Complete AV Block** 52

**4.17.2. Intraventricular Conduction Disorders** 52

**4.17.2.1. Left Bundle Branch Block** 52

**4.17.2.1.1. Pre-existing Left Bundle Branch Block** 53

**4.17.2.1.2. Exercise-induced Left Bundle Branch Block** 53

**4.17.2.2. Fascicular Blocks** 53

**4.17.2.3. Right Bundle Branch Block** 54

**4.17.2.3.1. Pre-existing Right Bundle Branch Block** 54

**4.17.2.3.2. Exercise-induced Right Bundle Branch Block** 54

**4.17.3. Disorders of Impulse Formation** 54

**4.17.3.1. Ventricular Arrhythmias** 55

**4.17.3.2. Supraventricular Arrhythmias** 56

**4.17.3.3. Atrial Fibrillation/Atrial Flutter** 57

**4.17.3.4. Bradyarrhythmias/Chronic Chronotropic Incompetence** 58

**4.17.3.5. Inappropriate Sinus Tachycardia** 58

4.18. Indirect Metabolic Assessment 58

**4.18.1. VO**_**2**_ **/METs** 58

**4.18.2. Functional Aerobic Impairment (FAI)** 59

**4.18.3. Myocardial Aerobic Impairment (MAI)** 59

4.19. Post-test Risk Scores and ET Prognostic Variables 60

**4.19.1. Duke Score** 60

**4.19.2. Athens Score/QRS Score** 60

**4.19.3. Raxwal and Morise Score** 61

5. Test Cessation Criteria 61

6. ET Reporting 61

6.1. General Information 61

6.2. Observed, Measured, and Recorded Data 62

6.3. Descriptive Report 63

6.4. Conclusion 63

6.5. ECG Recording 64

7. Other Diagnostic Tests Performed Simultaneously with or in Addition to ET 64

7.1. Ankle-brachial Index 64

**7.1.1. Method of ABI Measurement** 64

**7.1.1.1. Resting ABI** 64

**7.1.1.2. Post-exercise ABI** 65

**7.1.2. Patient Preparation and Measurement Technique** 65

7.2. Noninvasive Oximetry 66

**7.2.1. Equipment** 66

**7.2.2. Procedures for Noninvasive Oximetry** 67

**7.2.3. Interpretation of Findings** 67

7.3. Biomarkers and Other Laboratory Tests 67

**7.3.1. Blood Lactate** 68

**7.3.2. Arterial Blood Gas Analysis** 68

8. Particular Aspects of ET Performance and Interpretation in Specific Clinical Conditions 70

8.1. Dextrocardia/Situs Inversus 70

8.2. Chagas Disease/Chagas Cardiomyopathy 71

8.3. Peripheral Artery Disease 72

8.4. Parkinson’s Disease 73

8.5. Valvular Heart Disease 74

**8.5.1. Aortic Stenosis** 74

**8.5.2. Aortic Regurgitation** 75

**8.5.3. Mitral Stenosis** 75

**8.5.4. Mitral Regurgitation** 76

**8.5.5. Mitral Valve Prolapse** 76

8.6. ET after Myocardial Revascularization 77

**8.6.1. ET after Percutaneous Coronary Intervention** 77

**8.6.2. ET after Coronary Artery Bypass Grafting** 78

Part 3 – Cardiopulmonary Exercise Test 78

1. Introduction 78

2. Exercise Physiology Applied to CPET 78

3. Pulmonary Ventilation, Expired Gas Analysis, and Derived Variables 79

3.1. Pulmonary Ventilation 79

**3.1.1. Baseline Spirometry** 79

**3.1.2. Ergospirometry** 80

**3.1.3. Ventilatory Reserve** 80

3.2. Oxygen Consumption 81

3.3. Carbon Dioxide Production 81

3.4. Ventilatory Thresholds 81

**3.4.1. First Ventilatory Threshold** 81

**3.4.2. Second Ventilatory Threshold** 81

3.5. Respiratory Quotient 82

3.6. Ventilatory Equivalents of Oxygen and Carbon Dioxide 82

3.7. End-tidal Oxygen and Carbon Dioxide Pressures 83

3.8. Oxygen Pulse 84

3.9. Delta VO_2_to Delta Work Rate (ΔVO_2_/ΔWR) Ratio 84

3.10. Cardiorespiratory Optimal Point 84

3.11. Oxygen Uptake Efficiency Slope (OUES) 84

3.12. Exercise Oscillatory Ventilation 85

3.13. Oxygen Uptake Recovery Delay 85

3.14. Circulatory Power and Ventilatory Power 85

3.15. Reference Ranges for CPET Variables 85

4. Equipment and Methods 86

4.1. Ergometers 86

4.2. Airflow or Volume Transducers 86

4.3. Gas Analyzers 88

4.4. Measurements of Gas Exchange 88

4.5. Calibration, Quality Control, and Sanitization 88

4.6. Protocols 88

4.7. Data Analysis Software 88

4.8. Guidelines for Patients when Scheduling CPET 88

5. CPET in Selected Specific Situations 89

5.1. Heart Failure 89

5.2. Coronary Artery Disease 89

5.3. Hypertrophic Cardiomyopathy 90

5.4. Valvular Heart Disease 90

5.5. Lung Diseases 90

**5.5.1. Chronic Obstructive Pulmonary Disease** 90

**5.5.2. Pulmonary Vascular Disease** 91

5.6. Differential Diagnosis of Dyspnea 91

5.7. Athletes and Exercise Enthusiasts 92

5.8. Cardiorespiratory Rehabilitation 92

6. Interpretation and Preparation of the CPET Report 92

Part 4 – Exercise Test Combined with Cardiac Imaging Methods 93

1. Cardiovascular Stress Combined with Cardiac Imaging Methods 93

1.1. Myocardial Perfusion Imaging 93

**1.1.1. Physical Stress Methods (Exercise Test)** 93

**1.1.1.1. Contraindications to Physical Stress in MPI** 93

**1.1.1.2. Guidelines for Patients When Scheduing MPI with Physical Stress** 93

**1.1.1.3. Methodological Aspects of Physical Stress in MPI** 93

**1.1.1.4. Interpretation of ET Results in MPI** 93

**1.1.2. Methodological Aspects of Pharmacologic Stress in MPI** 94

**1.1.2.1. Vasodilators** 94

**1.1.2.1.1. Dipyridamole** 94

**1.1.2.1.2. Adenosine** 94

**1.1.2.2. Drugs that Increase Myocardial Oxygen Consumption** 95

**1.1.3. Hybrid Stress Methods** 96

**1.1.4. Novel Drugs** 96

1.2. Stress Echocardiography 96

**1.2.1. Methodology** 96

**1.2.1.1. Exercise Stress Methods** 96

**1.2.1.2. Pharmacologic Stress Methods** 97

**1.2.1.2.1. Dobutamine** 97

**1.2.1.2.2. Vasodilators** 97

**1.2.1.3. Ultrasound Enhancing Agents** 98

References 98

## Part 1 – Indications, Legal Aspects, and Training in Exercise Test

## 1. Introduction

Exercise test (ET) is a diagnostic modality, widely used in routine clinical cardiology practice, in which a person is subjected to a planned, individualized degree of physical effort with the purpose of evaluating their clinical, hemodynamic, autonomic, electrocardiographic, indirect metabolic and, occasionally, enzymatic responses to physical exertion.^1 , 2^When ET also included the assessment of ventilatory parameters and analysis of exhaled gases, it is called cardiopulmonary exercise test (CPET or CPX).^3^The term *cardiac stress test* encompasses both ET and CPET.

Overall, ET and CPET:

– Contribute to the diagnosis and prognosis of cardiovascular diseases, provide guidance to inform the selection of therapeutic interventions, assist in the adoption of preventive measures and support sports practice, are used in forensic medical opinions and in the medical examination of disability claimants, and provide vital information for the follow-up of patients over time.^1 , 3 - 5^

– Provide high reproducibility, well-established cost-benefit and cost-effectiveness, and are available in all regions of Brazil.^1 , 6^

– Are recognized and legally registered as an Area of Focused Practice by the Joint Commission on Medical Specialties.^7^

– Are of great importance as a cardiovascular stressor for stress perfusion imaging methods in cardiology, especially with a view to the diagnosis and prognosis of ischemic cardiovascular disease.^8 , 9^

This guideline consolidates and updates, in a single document, all information and recommendations present in previous SBC guidelines on TE and CPET, addressing new aspects not considered in previous documents, with particular emphasis on important new information related to testing in the adult population and necessary adaptations to TE/CPET practice in the setting of acute respiratory syndromes.^1 , 2^This Guideline will be a relevant source of reference for the general cardiologist and, in particular, for physicians training and working in the area of focused practice of exercise test.

## 2. Indications and Contraindications for ET, CPET, and Cardiac Stress Imaging

### 2.1. General Indications for ET

ET is widely available in Brazil, at an affordable cost and with recognized utility in clinical practice.^10 , 11^It is an important diagnostic tool for risk stratification and prognostication in patients with known or suspected heart disease. It allows assessment of the impact of cardiovascular diseases and of the effectiveness of implemented therapies.

General indications and overall objectives for ET include:^1 , 6 , 12 - 18^

1) Assessment of exercise-induced symptoms.

2) Determination of functional capacity.

3) Assessment of blood-pressure behavior.

4) Assessment of heart-rate behavior.

5) Detection of myocardial ischemia.

6) Recognition of cardiac arrhythmias and elucidation of their type, density, and complexity.

7) Assessment of the response to exertion in patients with channelopathies.

8) Diagnosis and prognosis of certain cardiovascular diseases.

9) Indication assessment of therapeutic interventions.

10) Assessment of the results of therapeutic interventions.

11) Preoperative assessment.

12) Assessment of cardiorespiratory fitness and physical conditioning.

13) Provide information to support the prescription of physical exercise, including in cardiopulmonary rehabilitation.

14) Provide information to support pre-employment health screenings, periodic check-ups, and disability and worker’s compensation claims.

ET can be performed for the aforementioned purposes in any clinical situations and conditions, as long as its relative and absolute contraindications are followed.

2.2. Specific Clinical Indications for ET

ET has had its effectiveness studied and tested in a range of specific clinical situations. This has allowed determination of the strength of recommendation and level of evidence for these specific indications, which will be presented in detail below.^6 , 12 - 14 , 17 , 19^

#### 2.2.1. Indications for ET in Coronary Artery Disease

Coronary artery disease remains a leading cause of morbidity and mortality, with an estimated prevalence of angina of 12% to 14% in men and 10% to 12% in women aged 65 to 84. In Brazil, approximately 30% of all deaths are due to cardiovascular causes.^20^

ET is indicated in the investigation of chest pain of probable cardiac origin due to its relevance, widespread availability, and cost-effectiveness, and has been endorsed as the optimal choice in this setting by the Choosing Wisely Initiative.^21^

The prevalence of asymptomatic CAD and silent ischemia varies widely depending on the population studied. Asymptomatic diabetics have a relative risk (RR) of 2.0 for CAD, and the prevalence of a positive ET is approximately 23% in these patients.^22 , 23^

The diagnosis of silent myocardial ischemia allows interventions aimed at reducing the risk of future events, including death.^24^

ET is recommended for risk stratification of patients with stable CAD, prognostication, assessment of the effectiveness of interventions, and investigation of changes in clinical manifestations.^25 - 27^

Even with a non-ischemic ET, patients with suspected CAD may benefit from ET-enhanced risk stratification, through prognostic variables such as exertional symptoms, functional capacity, blood-pressure and chronotropic response, autonomic function, and musculoskeletal response.^28^

In patients with CAD, ET is essential for the initial exercise prescription and to inform subsequent adjustments to the cardiovascular rehabilitation program (Table 1).^17 , 29 , 30^

#### 2.2.2. Indications for ET in Asymptomatic Patients

ET plays a relevant role in the evaluation of asymptomatic patients, as its variables (HR, blood pressure, ECG, etc.) provide information on prognosis and risk of future abnormalities.^57 , 58^

Cardiorespiratory fitness (functional capacity), as determined by ET, is considered a key marker of health and can be used to define therapeutic and preventive goals. In asymptomatic patients with comorbidities, ET assists in exercise prescription to promote health and well-being. ET is feasible and safe even in patients with advanced age and significant comorbidities.^59 , 60^

It also improves risk stratification of asymptomatic individuals in terms of physical fitness to carry out their duties at work without putting themselves or others at undue risk (Table 2).^61^

#### 2.2.3. Indications for ET in Athletes

Physical activity (PA) is defined as any bodily movement produced by the musculoskeletal system. Physical exercise, or physical training, is a program of structured, repetitive physical activity, with the aim of recovering, maintaining, or improving one or more components of physical fitness (cardiorespiratory, morphologic, muscular, metabolic, or motor). An athlete is defined an individual of any age, whether amateur or professional, who regularly engages in physical exercise with an emphasis on performance and may participate in competitive sports.^13 , 34^

TE provides important data for cardiologists, sports medicine and preventive medicine specialists regarding the health of elite athletes, Olympic athletes, professional athletes, competitive athletes, members of athletic federations and/or sports clubs, masters athletes, and recreational (pleasure and leisure) athletes. It is used in preparticipation physical evaluation and allows detection of latent pulmonary and cardiovascular diseases (i.e. exercise-induced asthma, hypertension, ischemia, arrhythmias, etc.), monitoring of response to interventions, and prognostic evaluation (Table 3).^13 , 17 , 34 , 68^

#### 2.2.4. Indications for ET in Hypertension

The systolic blood pressure (SBP) response during ET is considered a risk marker for development of hypertension, death from cardiovascular disease, and stroke.^71 - 73^Recent data suggest that the BP response to submaximal-intensity exercise has greater clinical and prognostic significance than the BP achieved during maximal-intensity exercise. Physical performance at the time of ET influences the interpretation of the BP response to exercise. Both hypotension and an exaggerated BP response serve as prognostic markers and indicators of the need to investigate underlying CVD (Table 4).^74 , 75^

Research has found that, in healthy male athletes undergoing ET, the workload-indexed BP response was superior to peak SBP as a predictor of mortality, and was particularly useful for preparticipation screening. A hypertensive response to ET was associated with development of hypertension in young athletes.^76^

#### 2.2.5. Indications for ET in Valvular Heart Disease

In valvular heart disease, ET should be routinely performed to elucidate questionable symptoms, evaluate indicators that can inform the decision to intervene, clear patients for exercise and inform the exercise prescription (Table 5).^92 - 94^ET is useful to unmask so-called “pseudo-asymptomatic” patients and allows serial follow-up of truly asymptomatic patients.^94^In symptomatic patients or those with exercise-induced symptoms, intervention, whether surgical or transcatheter, is indicated outright.^94^

Clearing a patient with valvular heart disease requires assessment of symptoms, functional capacity, and characteristics of the valve lesion and its impact on cardiac function. Asymptomatic individuals with lesions of up to moderate severity can engage in high intensity exercise if the ET reveals good functional capacity and absence of myocardial ischemia, hemodynamic derangements, or arrhythmias.^95^

#### 2.2.6. Indications for ET in Heart Failure and Cardiomyopathies

In heart failure (HF) and in cardiomyopathies, ET is used to elucidate symptoms, assess exercise tolerance/functional class, support prognostic assessment, and inform adjustment of therapy and prescription of exercise programs (Table 6).^29 , 116^

Exercise intolerance is a typical manifestation of HF, and functional class and HR response to ET are important prognostic variables.^117 , 118^

#### 2.2.7. Indications for ET in the Context of Arrhythmias and Conduction Disorders

Exercise-induced arrhythmias are often caused by cardiovascular conditions that can be evaluated by ET. These may be fully asymptomatic, or may present with symptoms ranging from palpitations to syncope. ET allows investigation of these symptoms, diagnosis and quantification (density) of arrhythmias, and stratification of the risk of sudden cardiac death (SCD). It also plays a relevant role in investigating the causes, impact, and therapeutic options for atrioventricular and intraventricular conduction disorders (Table 7).^63 , 128 - 132^

#### 2.2.8. Indications for ET in Other Clinical Conditions

Table 8 describes other clinical conditions in which ET is recommended, whether for functional assessment, to inform exercise prescription, or for adjustment and optimization of therapy.

## 2.3. Relative and Absolute Contraindications

ET is generally well-tolerated and safe when properly indicated and performed. However, some specific clinical situations may increase the risk of complications requiring immediate medical intervention (Table 9). The risk of sudden cardiac death is around 1 in every 10,000 exercise tests.^6 , 184 , 185^

### 2.3.1. Relative Contraindications to ET/CPET

These are high-risk clinical scenarios in which preventive and, potentially, therapeutic measures must be adopted before ET/CPET can be performed (Table 10). Such measures include carrying out the ET exclusively in a hospital setting; using modified protocols and a reduced target load; strict observation of emergent symptoms; more frequent BP measurements; and presence of trained personnel and equipment for reprogramming the patient’s pacemaker, ICD or other implantable devices as needed.

### 2.3.2. Absolute Contraindications to ET/CPET

Chart 1 lists absolute contraindications to the performance of ET/CPET.^1 , 6 , 12 - 17^

## 2.4. Indications for CPET

### 2.4.1. General Indications for CPET

The general indications for CPET are the same as for ET, but it is particularly indicated when there is a need to assess ventilatory and metabolic variables as well (Chart 2).

### 2.4.2. Specific Clinical Indications for CPET

Clinical situations for which there is sufficient scientific to determine a class of recommendation for CPET are listed in Table 11.

## 2.5. Indications for Cardiac Stress Imaging

### 2.5.1. Myocardial Perfusion Imaging

Myocardial perfusion imaging (MPI) is indicated in different clinical presentations of ischemic heart disease and contributes to defining its severity.^9^Other indications include evaluation of potential for revascularization in patients with viable myocardium and as part of preoperative assessment in specific situations (Table 12).^26 , 31 , 128 , 251 - 253^

Symptomatic patients at intermediate risk for ischemic heart disease benefit most from MPI for diagnostic and prognostic purposes. It should be performed, preferably, under stress, as long as the patient has a functional capacity above 5 METs and the ability to perform the necessary effort on whichever ergometer is available.

Patients with left bundle branch block (LBBB), Wolff-Parkinson-White (WPW) syndrome, or a pacemaker should undergo MPI with pharmacologic stress (dipyridamole or adenosine) instead.^9^

Among various indications for MPI, it is particularly useful in patients with low functional capacity or conditions that would prevent interpretation of whether an ischemic response to ET/CPET is present. MPI is also superior for CAD risk stratification in patients with a high pretest probability of CAD. MPI should be performed under pharmacologic stress in individuals with intermediate pre-test probability of CAD and an uninterpretable resting ECG or in those incapable or intolerant of physical exertion (Table 13).^9^

Following the recommendations of the Brazilian Guideline on Nuclear Cardiology, we adopted the following international score: MCI is appropriate for indications with a score of 7 to 9; possibly appropriate for indications with a score of 4 to 6; and rarely appropriate for those indications with a score of 1 to 3.^9^

Asymptomatic patients with no history of ischemic heart disease and a normal ET/CPET generally do not benefit from MPI. Asymptomatic patients with an abnormal ET may benefit from MPI, especially those at intermediate or high risk (Table 14).^9 , 254^

In patients who are asymptomatic after percutaneous coronary intervention (PCI) and/or CABG, MPI has a favorable cost-benefit ratio in follow-ups longer than 2 and 5 years, respectively. Patients who are symptomatic (angina or anginal equivalents) after PCI and/or CABG are considered to benefit from MPI at any time (Table 15).^9 , 31 , 251 , 252 , 254^

Patients with established CAD and worsening symptoms (or equivalent manifestations) can benefit from MPI at any time, with the main objective of quantifying ischemic burden (extent and intensity of defects) and informing medical management (Class of recommendation-Level of evidence: I-C).^252^

In patients with acute chest pain with suspected ACS, normal ECG (no ischemic changes or necrosis) or uninterpretable ECG (LBBB, WPW, or pacemaker rhythm), and normal cardiac biomarkers, resting MPI presents has a high negative predictive value (NPV), thus allowing patients to be discharged from the emergency department (Table 16).^1 , 9 , 33 , 254 - 258^

Assessment of myocardial viability through MPI aids in selection of patients with severe left ventricular dysfunction who are eligible for myocardial revascularization (Table 17).^1 , 9 , 31 , 128 , 258 , 259^

Other indications for MPI, such as investigation of heart failure, arrhythmias, syncope, patients with high calcium score (≥400), patients with diabetes, chronic renal failure, or a family history of ischemic heart disease, and preoperative risk assessment before noncardiac surgery and vascular surgery, are covered in the Update of the Brazilian Guideline on Nuclear Cardiology.^9 , 253^

### 2.5.2. Indications for Stress Echocardiography

Stress echocardiography (EcoE) is a noninvasive imaging method used for diagnosis, risk stratification and prognosis, and assessment of myocardial viability in coronary artery disease (CAD), valvular heart disease, and cardiomyopathies.^260^

In investigation of ischemia, it provides good accuracy in moderate-to-high-risk patients, with slightly higher specificity compared to other noninvasive imaging methods, such as MPI.^260 - 262^However, it should not be considered a substitute for ET; instead, it is indicated in patients with limitations or contraindications to ET.^262^

The applicable stress testing modalities are exercise (ESE; performed on a treadmill, conventional bicycle/cycle ergometer, or in-bed/supine cycle ergometer) or pharmacologic, either with dobutamine (DSE; atropine can be added to increase sensitivity) or with a vasodilator (adenosine or dipyridamole; rarely performed). Both ESE and DSE have similar diagnostic performance for investigation of ischemia. However, ESE (Class of recommendation-Level of evidence: I-A). allows better interpretation of functional impact, assessment of cardiorespiratory fitness and ventricular dysfunction, and decision-making regarding prognosis and treatment in ischemic heart disease, valvular heart disease, and cardiomyopathies (Table 18).^8 , 260 , 263^

Stress echocardiography can be recommended for risk stratification of patients with acute coronary syndrome in chest pain units (Table 19) and as part of the workup for stable CAD (Table 20). The main indications of stress echocardiography in other, nonischemic CVDs are listed in Table 21.

## 3. Legal Aspects and Essential Conditions for Performing ET, CPET, and Cardiac Stress Imaging

### 3.1. Legal Aspects Involved in the Practice of ET and CPET

ET and CPET are widely accessible, reproducible, noninvasive methods with a low risk of complications in unselected populations.^6 , 10 , 11 , 277^As their performance in Brazil is strictly limited to physicians, they are governed by the Code of Medical Ethics and, therefore, the physician must be aware of their possible ethical and legal implications, duly addressed in the Code of Medical Ethics of the Federal Medical Council ( *Conselho Federal de Medicina* ), Brazilian Civil Code, Consumer Protection Code, and other applicable laws (Appendix 1).

### 3.2. Essential Conditions for Performing ET and CPET

Given the specificities of the various stress testing methods and the established legal framework, the following are essential conditions for ET/CPET:

1) Exercise test and cardiopulmonary exercise test are medical procedures under the exclusive responsibility of a qualified physician, who must be physically present at all stages of the procedure. ET and CPET cannot be performed under any circumstance by means of any modality of telemedicine or remote reporting, nor may more than one ET/CPET be conducted simultaneously by the same physician – even in person. This restriction is due to the need to carry out procedures and diagnoses throughout the examination which are the sole and exclusive attribution of physicians, as well as respond to any complications or emergencies.

2) The qualified physician performing the examination must be registered with the Medical Council and must be fit to practice medicine. The Department of Exercise Test, Exercise, Nuclear Cardiology and Cardiovascular Rehabilitation of the Brazilian Society of Cardiology (SBC/DERC) recommends that the physician be board-certified in Cardiology by the Brazilian Medical Association and hold a focused practice designation in exercise test, both duly registered with the Federal Medical Council (FMC).

3) ET and CPET should only be performed upon formal medical request.

4) Written informed consent from the patient or his/her legal guardian (for patients under 18 years of age) is mandatory.

5) If the patient is underage or otherwise legally incapable, it is recommended that their legal guardian or proxy remain in the examination room.

6) The exercise test service or department must have all the recommended equipment available, as well as all of the supplies (including equipment and drugs) necessary for emergency response, as stated in this Guideline.^278 - 280^

7) The physician responsible for the test must obtain a summary history, perform a targeted physical examination, and perform a pre-test ECG. Current medications, comorbidities, and risk factors must be recorded.

8) Relative and absolute contraindications to ET/CPET must be assessed.

9) When choosing the ET/CPET protocol, the clinical conditions of the patient, the referring physician’s request, the availability of ergometers, and the experience of the performing physician must all be considered.

10) After the test, the patient may only be cleared or discharged once clinically and hemodynamically stable.

11) If serious or life-threatening adverse events arise during the test, the physician responsible for the test will provide the necessary support until the attending physician and/or emergency medical services are able to effectively take over or transfer to the emergency department can be completed. If the event is fatal, the physician responsible for the test is advised to notify the Medical Council and request an opinion from its Ethics Committee.

12) After the test, the patient should be instructed to return to the requesting physician for further management. If the patient or his/her legal guardian or proxy inquires as to the result of the test, the physician performing the test must provide any relevant information.

13) Compensation for the test should include a fair physician’s fee and cover all operating costs.

14) The physician performing the test must follow all recommendations of public health authorities and medical societies regarding any ongoing endemics, epidemics, and pandemics, as well as the applicable rules and regulations of the patient safety system.^281^

15) ET and/or CPET involves obtaining and processing sensitive patient data, and exercise test services must therefore respect the Brazilian General Data Protection Law and other relevant legislation and FMC ordinances.^282 - 284^

### 3.3. Informed Consent Forms for ET/CPET

Informed consent forms (ICFs) for ET/CPET and the consenting process itself must follow the guidelines of the Brazilian Code of Medical Ethics and FMC Recommendation Nº 1/2016.^285^

### 3.4. Informed Consent Forms for Cardiac Stress Imaging

ICFs for exercise stress echocardiography, myocardial perfusion imaging, and positron emission tomography must also follow the determinations of the departments and specialty societies and associations involved, in compliance with the guidelines of the Code of Medical Ethics and FMC Recommendation Nº 1/2016.^285^

## 4. Aspects Related to Training in Exercise Test as an Area of Focused Practice

Exercise test is recognized as an area of focused practice by the Joint Commission on Medical Specialties (CME) of the FMC, Brazilian Medical Association, and Brazilian National Medical Residency Commission (CNRM).^7^Specific training in exercise test as an area of focused practice seeks to provide cardiologists with an opportunity for professional improvement and, consequently, improve the quality of cardiologic diagnostic services and care for patients undergoing ET and CPET.

Such training must follow the legal determinations established by the aforementioned medical organizations and the following SBC/DERC recommendations:

1) Must take place at an educational facility with an active, formally constituted exercise test service, registered with all relevant public authorities, with regular and up-to-date paperwork (including Department of Health clearance). The educational facility may be subject to registration, assessment, and accreditation by SBC/DERC.

2) Routine performance of ET is considered a bare-minimum requirement for the establishment of an educational program providing ET/CPET training. In order to comply with the practical aspects of the program, the facility must also routinely perform CPET and both methods (ET/CPET) in combination with imaging modalities (cardiac stress imaging). The training facility may establish an official agreement with another institution to carry out practical training in CPET and/or cardiac stress imaging.

3) The manner of selection of participants for the training program shall be at the discretion of the facility, and may comprise an interview and/or theoretical test and/or practical test. Facilities are advised to publicize their selection process through public announcements listing the prerequisites, manner of registration, selection criteria, and timetable, and publicize the result of the selection process as well. The facility must ensure a fair, equitable, and transparent process.

4) As a mandatory prerequisite for training in exercise test, candidates must have completed a medical residency in Cardiology or be board-certified in Cardiology and registered as such at the Brazilian Medical Association/FMC.

5) The aim of any training program in exercise test must be for qualified cardiologists to acquire the necessary experience to be responsible for the performance, interpretation, and organization of ET/CPET services. Programs shall be theoretical and practical, with a duration of 12 months (1 year) and a minimum workload of 960 hours (48 weeks of 20 hours of training per week + 30 vacation days).

6) The theoretical portion shall correspond to a minimum of 10% and a maximum of 20% of the total program workload, and shall be dedicated exclusively to theoretical activities: classes, seminars, scientific meetings, conferences, discussion of scholarly articles (journal club), clinical/cardiological grand rounds, and meetings focusing on interpretation of invasive and noninvasive diagnostic tests.

7) The theoretical syllabus must include, at a minimum, all topics and subjects covered in this Guideline. Additional training in basic research techniques, diagnostics, essentials of scientific methodology, basic statistics, ethics, and how to communicate with patients is advised.

8) The practical portion of the program must be under the direct, on-site supervision of a *preceptor* , who must be board-certified in Cardiology and hold a focused practice designation in exercise test. All programs must have a minimum ratio of one preceptor to two participants or fewer.

9) Practical training shall be divided into: initial training under the direct supervision of the preceptor, corresponding to at least 25% of the total workload of the practical portion of the program; and training under indirect supervision, once the candidate has passed the direct supervision period, corresponding to at least another 55% of the total workload. The recommended distribution of tests during the practical portion of the training program is as follows: 70% ET (at least), 15% CPET, and 15% cardiac stress imaging.

10) All of the aforementioned prerequisites appy to established cardiology training programs which include an additional year of training in exercise test accredited by the Brazilian Ministry of Education (MEC). Program activities must comprise a total workload of 2,880 hours, distributed as follows: 10% to 20% (288 h to 576 h) theoretical activities and 80% to 90% (2,304 to 2,592 h) practical activities. For the practical portion of the program, it is recommended that part of the ET and CPET tests be performed in combination with other imaging methods, and part within the context of evaluation for cardiovascular rehabilitation programs and sports cardiology.

11) Periodic training in emergency care is also recommended, to ensure optimal management of patients who experience complications during testing. This training should corresponding to completion of an Advanced Cardiovascular Life Support (ACLS) or Brazilian Society of Cardiology *Treinamento de Emergências Cardiovasculares Avançado* (TECA-A) course.

12) The educational facility may provide training and/or education in other cardiological and non-cardiological diagnostic methods independently or simultaneously with training in exercise test. However, if this is the case, there can be no interference in the exercise test training program, nor will these activities be computed toward the theoretical and/or practical training workload.

13) The training facility must carry out an evaluation of the participants, using its own criteria, during and/or at the end of the training program. It´s recommended to maintain transparency in the evaluations, previously defining the objective criteria that will be required, and including a self-evaluation with an attitude scale. When candidates fail the program, it is suggested that the training facility provide additional training options to remedy any pending issues, followed by a reassessment. The training facility must provide an official certificate to all approved candidates, as well as a declaration of compliance with all requirements listed herein.

14) SBC recommends that all candidates who have successfully completed training take the AMB/Brazilian Society of Cardiology board exam to obtain focused practice designation in Exercise Training, and subsequently register said designation with the FMC.^286^

15) After completion of the training program, periodic participation in scientific events/refresher programs in ET and TCPE, at the national and/or international level, are highly recommended for revalidation and continuous improvement of the qualification acquired during training.

## Part 2 – The Exercise Test

## 1. ET Methodology

Performing an ET necessarily requires compliance with a series of methodological conditions, both to maintain patient safety and to obtain valid and reproducible results.

### 1.1. Core Conditions to Perform ET

#### 1.1.1. Team

ET must be performed by a qualified, experienced physician, who must be present in the examination room, performing a single test at a time, and who must issue the corresponding report after the test. This physician may be assisted by other health care providers (registered nurse, nurse technician, or nursing assistant), all of whom must have been specifically trained to assist in ET/CPET and participate in any emergency response.^286 , 287^

The facility and/or the physician should properly guide and train any other providers potentially involved in the ET regarding the scheduling of the test, cleaning of equipment, cleaning of the examination room, and patient transport.

#### 1.1.2. Physical Infrastructure

ET must be performed in a planned, well-lit and well-ventilated environment, large enough to accommodate all ET equipment (including an examination table or stretcher/gurney, patient chair, and a crash cart) while also allowing circulation of at least three people (at least 7 m^2^), at a controlled ambient temperature of 18-22°C and a relative humidity of at least 40%.^288 - 290^

#### 1.1.3. Equipment

Recommended essential equipment: ergometer; exercise test system (including display for ECG monitoring); printer (or print server access); calibrated sphygmomanometer; stethoscope; wall-mounted thermometer; fingertip pulse oximeter; armchairs for patient and physician; examination table or stretcher/gurney; crash cart (if there is only one examination room); oxygen cylinder (next to crash cart) or wall-mounted oxygen port in each examination room; portable suction device (next to crash cart) or wall-mounted suction in each examination room; waste receptacles (for common and hospital waste).^4 , 13^

The following steps are recommended before each test:^4 , 13^

– Wipe the ECG patient cable with 70% alcohol.

– Clean and disinfect the treadmill support bar, cycle ergometer saddle, stethoscope, etc. Use appropriate cleaning supplies according to routine institutional protocol.

– Preferably use disposable materials, and dispose of them properly in an appropriate container.

#### 1.1.4. Emergency Supplies

A crash cart stocked with basic and advanced life support supplies must be available on site wherever ET and/or CPET are performed. This guideline recommends that ET/CPET facilities adopt the standardized crash cart composition given in the Brazilian Society of Cardiology Guideline for Cardiopulmonary Resuscitation and Emergency Cardiovascular Care (see Box 17.2: Standardization of the emergency trolley in the intensive care unit and emergency department).^279^

#### 1.1.5. Emergency Drugs

This guideline recommends that ET/CPET facilities stock the standardized formulary of basic and advanced life support drugs given in the Brazilian Society of Cardiology Guideline for Cardiopulmonary Resuscitation and Emergency Cardiovascular Care (see Box 17.2: Standardization of the emergency trolley in the intensive care unit and emergency department).^279^

#### 1.1.6. Orientations for Patients when Scheduling ET

Patients should be instructed to:^4 , 13^

– Avoid smoking for 3 hours before the test.^291^

– Refrain from strenuous or unusual physical exertion on the day of the test and on the day before.

– Avoid fasting or eating to excess before the test; have a light meal 2 hours before. Refrain from drinking any alcoholic beverages and/or caffeine-rich beverages (including energy drinks) on the day of the test and on the day before.

– Wear shorts or comfortable pants and shoes with rubber soles and no heel (preferably sneakers/tennis shoes). Women should be advised to wear a regular or sports bra.

– Bring the ET request or order form.

– Bring the reports of any previous ETs.

– Whether to withhold or continue any medication remains at the discretion of the patient’s attending physician.

When the purpose of the ET is diagnosis of CAD, some drugs must be discontinued as they may interfere with the result (Table 22). No drugs should be withheld if the purpose of the ET is to inform exercise prescription or assess treatment response. Discontinuation should be done sparingly and weighing the risks of clinical decompensation against the benefits of any additional information to be gained from the test. Some drugs may influence (positively or negatively) the duration of exercise, the exertional load achieved, ischemia and angina thresholds, the onset of anginal pain, ST-segment depression and time to ST-segment normalization, the HR and BP responses, etc.^6 , 13^

## 1.2. Basic ET Procedure 292

### 1.2.1. Pre-test Phase

The physician responsible for the ET must ascertain whether the patient has complied with the pre-test recommendations and answer any questions regarding the test and the consent process. If the patient refuses to sign the ICF, the test cannot proceed.

### 1.2.2. Initial Assessment

The physician performing the ET is advised to evaluate the referring physician’s request, the reason for ET, the patient’s current medications and symptoms, and obtain a history and targeted physical examination of the cardiovascular and respiratory systems, with a view to identifying possible relative and absolute contraindications for ET (Table 23). The patient’s daily activities should also be reviewed, in order to identify possible limitations and inform an appropriate choice of protocol and ergometer.^6 , 13 , 293^

Pre-test hyperventilation is not recommended, as it can cause chest discomfort, bronchospasm, and ECG changes that might interfere with the accuracy of the ET.^294^

### 1.2.3. Targeted and Specific Physical Examination

The physical examination should be carried out in a targeted manner, directed by the history and review of systems. Heart and lung auscultation and measurement of resting BP and HR are mandatory.

The importance of auscultation in the evaluation of valvular heart disease, HF, lung diseasea, investigation of dyspnea, and in post-COVID patients cannot be overstated. In these patients, measurement of oxygen saturation using a fingertip pulse oximeter is recommended, since inadequate saturation (SpO_2_≤92% on room air) is a contraindication to ET. Partial desaturation (SpO_2_>92% and <95% on room air) requires special precautions, including pulse oximetry monitoring throughout the ET (see section Tests Performed Simultaneously and In Addition to ET).^122 , 226 , 228^

### 1.2.4. Electrocardiographic Monitoring and Recording System

Continuous ECG monitoring and recording are mandatory at all stages of the ET: rest, stress, and recovery. Hypoallergenic long-term monitoring electrodes with extra tacky (diaphoretic) adhesive are recommended.

Ideally, a computerized stress testing system, including software that allows continuous ECG monitoring, data collection, recording, and interpretation, should be used. This system should undergo preventive maintenance in accordance with current legislation and receive routinely scheduled updates.^13 , 115^

Performing a conventional 12-lead ECG before the ET/CPET is also advised. Conventional ECG is a supplemental noninvasive test for assessment of the patient’s cardiac condition, which may help uncover potential contraindications to ET/CPET. The conventional ECG differs from the ET/CPET recordings in that it includes peripheral electrodes on the extremities, is performed with the patient in supine position, and uses specific signal filters for resting ECG. A conventional 12-lead ECG is considered a medical procedure, and as such is covered in the Hierarchical Brazilian Classification of Medical Procedures (code 4.01.01.01-0).^295^

**1.2.4.1. Three-lead Systems**

Usually comprise a combination of two bipolar leads (CM5 = mandatory; aVF or modified lead II [lead MII]) and one unipolar lead (usually V2). Their use in ET is no longer recommended, given the established superiority of systems with additional leads.^296^

**1.2.4.2. Twelve-lead Systems**

The classic Mason-Likar 12-lead system, its modified version (without substitition of the CM5 lead), or a 13-lead placement (Mason-Likar + CM5) are recommended for ET.^6 , 297 , 298^

Electrode positioning to obtain the classic Mason-Likar lead system placement is shown in Figure 1:

1) The right arm electrode is placed near the root of the right shoulder, in line with the second right intercostal space.

2) The left arm electrode is placed near the root of the left shoulder, in line with the second left intercostal space.

3) The right leg electrode is placed on the highest portion of the right iliac crest (preferably just below the umbilicus, on the right midclavicular line). This electrode is important as a reference for electrical impedance and technical quality of ECG tracings, and its position does not directly interfere with Einthoven’s triangle.

4) The left leg electrode is placed on the highest portion of the left iliac crest (preferably just below the umbilicus, on the left midclavicular line).

5) Precordial leads are positioned at points V1 to V6 of the standard ECG placement:

– V1: in the 4th intercostal space, on the right parasternal line.

– V2: in the 4th intercostal space, on the left parasternal line.

– V3: between electrodes V2 and V4.

– V4: in the 5th intercostal space, on the left midclavicular line.

– V5: in the 5th intercostal space, between V4 and V6, on the anterior axillary line.

– V6: in the 5th intercostal space, on the midaxillary line.

Note: Electrodes V4, V5, and V6 must be placed at the same level along a horizontal line that does not necessarily follow the intercostal space.

Electrode positioning to obtain the modified 12-lead placement with CM5 (Figure 1):

1) The right arm electrode is placed adjacent to the sternal notch (manubrium).

2) The left arm electrode is positioned as for lead V5 on the classic ECG placement (5th intercostal space, anterior axillary line).

3) The right leg electrode is placed just below the right costal margin, on the right midclavicular line (or on the highest portion of the right iliac crest).

4) The left leg electrode is placed just below the left costal margin, on the left midclavicular line (or on the highest portion of the left iliac crest).

5) Precordial leads:

– V1, V2, V3, V4 and V6 as for the standard ECG placement.

– V5: shifted to the left side, placed immediately before the classic V6 lead.

**1.2.4.3. Placements Including 13 or More Leads**

A 13-lead system in which the CM5 bipolar lead is added to the classic Mason-Likar 12-lead system (Figure 2) is the most widely used system in Brazilian stress-testing equipment. The 13th lead is obtained by placing an additional electrode adjacent to the sternal notch (manubrium).^152 , 299^

CM5 is considered the single most sensitive lead for detection of myocardial ischemia, although this does come with a slight decrease in specificity. It monitors the anterolateral region of the left ventricle.^152^

The 16-lead system, which adds right precordial leads V1R, V2R, and V3R, has not become established in clinical practice, although studies have shown a significant improvement in ET sensitivity and specificity when it is used, especially for detection of lesions in the right and circumflex coronary arteries.^134^

**1.2.4.4. Skin Preparation for ECG Monitoring**

Skin preparation is essential to ensure a high-quality ECG tracing. The recommended method is to clean the skin where electrodes will be placed with alcohol wipes (70% to 99% concentration). In older adults and children, greater care should be taken to prevent abrasions due to increased skin sensitivity and propensity for injury. In men with excess body hair in areas where electrodes will be placed, shaving with a disposable blade is recommended.^299^

**1.2.4.5. ECG Recording**

ECG recordings must be obtained immediately after BP and HR measurements at rest (in supine and orthostatic position); at the end of each stage of exercise; at the peak of effort; and during the recovery phase (at 1, 2, 4, and 6 minutes of recovery as the bare minimum, or until normalization of any emergent ECG changes). Additional recordings should be obtained in the event of abnormal rhythms, atrioventricular and intraventricular block, or ST-segment deviation.

### 1.2.5. Hemodynamic Monitoring

**1.2.5.1. Heart Rate Monitoring**

HR behavior reflects the autonomic system response to exertion, providing diagnostic and prognostic information.^6^In ET, it is conceptualized as follows:

– Maximal heart rate (HRmax): that reached at the point of exhaustion.

– Peak heart rate (HRpeak): the highest HR observed at peak exertion, even if the subject has not reached the point of exhaustion.

The HRmax that an individual will reach during ET can be estimated through regression equations, adjusted for age and/or sex. There is no consensus on the best HRmax estimation equation. Those most widely used are:

HRmax prediction equations for both sexes:

**HRmax = 220 – age** (Karvonen et al., 1957)^300^

**HRmax = 208 – (0.7 × age)** (Tanaka et al., 2001)^301^

Sex-specific HRmax prediction equations:


$$\text{HRmax}=192-(0.7\times \text{age})(\text{Calvert et. al,}1977\text{- para mulheres}{)}^{302}$$

$$\text{HRmax}=201-(0.6\times \text{age})(\text{Calvert et. al.,}1977\text{- para homens}{)}^{302}$$

HRmax is influenced by individual conditions, the type of ergometer, emotional state, metabolic state, physical capacity, medication/drug use, and implantable devices, among multiple other factors (i.e. ambient temperature, relative humidity, etc.).^301 , 303 , 304^

Continuous monitoring of HR throughout the ET is recommended, as well as continuous recording of HR alongside the ECG tracing.

**1.2.5.2. Blood Pressure Monitoring**

BP measurement should be performed by a duly trained, experienced provider. It may be:^13 , 134^

– Manual, performed with an aneroid sphygmomanometer.

– Semiautomated, performed with a monitoring device not synchronized with the ECG signal.

– Automated, performed with an auscultatory blood pressure device synchronized with the ECG signal.

Automated measurement is limited at high rates of exertion, due to increased body movement and instability of the upper extremities.

All forms of measurement must use a Velcro® cuff of the appropriate size for the subject’s arm circumference, protected against excess sweat with a paper towel or mesh tubing. The stethoscope or sensor (if an automated device is used) must be placed over the brachial artery.

It is good practice to measure BP in both arms before the test and obtain all subsequent measurements (both during the test and in recovery) in the extremity with the highest BP level (usually the left arm). In case of pre-test hypertension, repeated, accurate measurements should be obtained in both arms.^215^

Arm BP measurement is contraindicated in case of arteriovenous fistula, history of lymph node dissection, thrombosis, lymphedema, and/or radial artery harvesting.^134 , 305^

BP should be measured (at the very least): before exercise; at the end of each stage of a step incremental protocol or every 2 minutes with a ramp incremental protocol; when the patient’s exertion reaches 5 METs (see Section 3.2.2. Blood Pressure Response); at the peak of effort; and during recovery (at 1, 2, 4, and 6 minutes). Measurements should be continued for as long as is necessary during the recovery period. BP should be reassessed whenever there are any discrepancies or a measurement is deemed unreliable or otherwise questionable.

### 1.2.6. Monitoring for Signs and Symptoms

Any signs and symptoms presented at rest, during exercise, and during recovery must be objectively monitored and described in the ET report, including the reason for cessation in the event of a symptom-limited test. All symptoms that are directly related to the reason for requesting the ET should be recorded in detail.

Subjects should be monitored particularly closely for chest pain (precordial or retrosternal), with or without dyspnea, which may indicate exertional angina.^306 , 307^

Dyspnea on exertion may be related to heart disease, lung disease, and exercise-induced asthma.^225 , 307 - 309^On general physical examination, skin color (pallor, cyanosis), diaphoresis, and any distinctive breathing and gait patterns should be noted and recorded.^175 , 310^

### 1.2.7. Prevention of ET Complications

The following are recommended as preventive measures:^13 , 152^

– Respect the criteria for performing ET in a hospital setting with the necessary backup services.

– Select the ergometer and protocol most suitable for each patient.

– When necessary, allow the patient to hold onto the treadmill handrail; this increases patient safety and improves the quality of the ECG tracing.

– Observe the patient’s behavior and posture on the ergometer.

– Respect the patient’s reported tolerance limits and the established criteria for test cessation.

– Have the necessary material at hand to manage potential emergencies and complications.

## 1.3. Ergometers

Ergometers used for ET/CPET are designed specifically for this purpose and must be registered with the Brazilian Health Surveillance Agency. The main types of ergometer are the cycle ergometer (stationary bicycle), treadmill, and upper-body ergometer (arm machine or arm cycle), among others. The ergometers most commonly used in Brazil are the treadmill and cycle ergometer.

The choice of ergometer should take into account the indication for ET (i.e. prefer a cycle ergometer for patients with syncope and suspected catecholaminergic ventricular tachycardia); the patient’s habitual physical activity; whether testing will be one-off or serial (ideally, the latter should always be performed on the same ergometer); device availability; and the patient’s physical limitations.^4 , 6^

### 1.3.1. Cycle Ergometer

The cycle ergometer should be preferred for use by cyclists; patients with neurological, visual, or balance issues; and when ET is performed for investigation of syncope (for fall prevention purposes). It facilitates BP measurement and cardiopulmonary auscultation during exertion.

On a cycle ergometer or stationary bicycle, the effort load is increased by means of a mechanical or electromagnetic brake. It’s considered ideal to maintain pedaling speed at 60 rpm for an adequate estimate of VO_2_by formula, but considering individual limitations (example: age, morbidities, etc.) a variation between 40 and 70 rpm is accepted.

Key limitations: difficulty in coordination and maintaining constant speed in older adults and subjects not used to cycling; in the latter, higher SBP values and lower HR and VO_2_values (5% to 25% lower) are found compared to treadmill testing.^1 , 292^

### 1.3.2. Treadmill

Treadmills are more suitable for the general population, as adaptation to the ergometer is easier. They also allow higher HR and VO_2_to be reached compared to a cycle ergometer. The work load on a treadmill can be increased by increasing the grade (incline), alone or in addition to an increase in speed.^1^

Key limitations: greater difficulty in measuring BP at high speeds; may trigger vertigo syndromes; unsuitable for patients with escalaphobia (a fear of escalators or treadmills); less suitable when the indication for ET involves a risk of falls (i.e. investigation of syncope).^134^

### 1.3.3. Upper-Body Ergometer

Upper-body or arm ergometers are recommended for subject who are unable to exercise with the lower limbs, as well as for those who practice sports predominantly involving the upper body.^311^BP measurement should be performed on the thigh (or on one arm, while the other remains active). The total muscle mass involved in exercise is smaller. When compared to ET on a treadmill, the VO_2_and SBP values achieved are generally lower.^312^

### 1.3.4. Other Ergometers

Other options are available, such as wheelchair-adapted treadmills; row ergometers; and adapted swimming pools and water tanks, among others. Each ergometer requires its own individualized protocol, as well as distinct, specific formulas for VO_2_estimation. Possible variations in hemodynamic response related to the choice of ergometer must also be considered.^134^

## 1.4. Choice of Protocol

Exercise test protocols can be divided according to the mode of effort exerted:

1) Incremental (gradual increase in load):

– Step (scaloned, graded or stepped): load is increased in stages (in a stepwise manner, as the name implies) at predetermined time points (i.e. every 1 minute or at longer intervals). Step protocols generally involve a substantial load increment at the end of each stage.

– Ramp: small, frequent load increments (tending to a linear increase) at very short time intervals (seconds, not minutes).

2) Fixed-load: there is no increase in load at any point during the test. When performed on a treadmill, speed and grade (incline) are simply kept constant. Fixed-load protocols are reserved for specific clinical settings (i.e. in determining initial and absolute claudication distances, or the post-exercise ankle-brachial index).^1 , 4^

The choice of protocol should be individualized, taking into account the indication for ET and the patient’s level of physical conditioning and possible physical limitations, aiming at an ideal exercise time of 10 minutes (ranging from 8 to 12 minutes).^1 , 4^

### 1.4.1. Cycle Ergometer Protocols

Several ET protocols are available for cycle ergometers and major ones are listed in Table 24. The workload performed on a cycle ergometer is generally expressed in watts (W).

The most widely used protocol is Balke’s. Maximal oxygen consumption (VO_2_max) is estimated with the formula: VO_2_max = (12 × load [W]) + 300/body mass (kg).

Ramp protocols are more commonly used in athletes and for CPET. VO_2_max may also be calculated rather than estimated, most commonly with the ACMS formula^313^:


$${VO}_{2}\max(mL/kg/min)=10.8\times W+7/\text{weight}(kg)$$

In this equation, VO^2^max relative to weight is estimated as:


$${VO}_{2}\max(mL/kg/min)=(W\times 11.4+260+\text{weight}\times 3.5)/\text{weight}$$

### 1.4.2. Treadmill Protocols

**1.4.2.1. Step Protocols**

**1.4.2.1.1. Bruce Protocol^4 , 13 , 134^**

The Bruce protocol is most widely used step protocol. It is recommended for the general adult population, and is also suitable for older adults without physical limitations and at least some degree of physical conditioning. VO_2_max can be estimated with the following formulas:^13 , 134 , 293^


$$\text{Men:}{VO}_{2}\max=(2.9\times \text{time in minutes})+8.33$$

$$\text{Women:}{VO}_{2}\max=(2.74\times \text{time in minutes})+8.03$$

The Bruce protocol includes abrupt increments in workload and, in sedentary individuals, this can cause loss of balance and difficult adaptation. In athletes, conversely, the load increments are generally too small, making the test very long.

**1.4.2.1.2. Modified Bruce Protocol^13 , 134^**

Several modifications aim to make the Bruce protocol more suitable for adults and elderly subjects with reduced physical capacity. The best-known adaptation is that suggested by Shefield (Table 25), in which the first two stages involve a reduced load and, from the third stage onward (which corresponds to the first stage of the unmodified Bruce protocol), the original protocol is followed, with large load increments at the end of each stage.

**1.4.2.1.3. Ellestad Protocol^13 , 134^**

This protocol employs marked increases in speed with a fixed grade up until the fourth stage of the test. It is preferably reserved for young subjects, physically active adults, and older adults who are habitual runners.

The key limitation of this protocol is that it begins at fairly high speeds, making adaptation difficult for subjects who are not used to running and hindering BP measurement somewhat.

**1.4.2.1.4. Naughton Protocol^13 , 134 , 293^**

This protocol involves small load increments (equivalent to 1 MET per stage). It is preferred for use in sedentary subjects, older adults, individuals with physical limitations/low physical capacity, and in compensated heart failure, recent myocardial infarction (MI), and peripheral artery disease of the lower limbs.

This protocol should not be used in active patients, as it prolongs the test unnecessarily and makes it difficult to reach maximal effort.

**1.4.2.2. Ramp Protocol^4 , 6 , 13^**

Ramp protocols can be fully individualized in terms of speed, grade (initial and final), and duration. They allow better assessment of cardiorespiratory fitness (aerobic capacity).

Clinicians are advised to use a scale of daily physical activity or a questionnaire that estimates maximal functional capacity (such as the Duke activity scale or the Veterans Specific Activity Questionnaire [VSAQ]) to define the upper limit of physical exertion for a maximal test. If this limit is underestimated or overestimated, the target load can be adjusted during the test itself in order to maintain the target duration of 8 to 12 minutes.^13 , 134 , 314^

The load increment rate depends on the programmed initial and final speed and grade. In sedentary individuals or those with physical limitations, the ET should ideally start at a low speed (1.6 to 2.7 km/h) and grade (0% to 5%). In active individuals, the suggested starting speed is 2.7 to 4.0 km/h (grade 0% to 5%).^13 , 315 , 316^

Foster’s formulas are recommended for calculation of VO_2_:^317^


$${VO}_{2}=0.694\times ACSM{VO}_{2}+3.33$$

(when holding onto handrails)


$${VO}_{2}=0.869\times ACSM{VO}_{2}-0.07$$

(when not holding onto handrails)

### 1.4.3. Upper-Body Ergometer Protocol

The load increment should usually be half of that employed in lower-body protocols. Balady et al. developed a protocol with an initial load of 10W, adding 10W every 2 minutes, and maintaining a constant speed of 75 to 80 rpm.^318^

Mitropoulos et al. developed a protocol with an initial load of 30W for men and 20W for women, a linear ramp increase (10 W/min for men and 6 W/min for women), and a rate of 70 rpm.^319^

### 1.4.4. Test Cessation/Termination

Temporary cessation of effort during ET/CPET can be done by slowing down or even stopping the ergometer briefly, in exceptional situations: as necessary to confirm BP levels; to adjust or replace electrodes; for cardiopulmonary auscultation; in case of vertigo (with a view to attempted resumption of exercise); etc.

After the exercise phase, an active recovery period of at least 1 to 3 minutes is recommended:

– On a cycle ergometer, gradually unload the internal resistance.

– On a treadmill, initially perform a slow, gradual deceleration and declination, followed by simple walking at a speed of 1.5 mph (2.4 km/h) and 2.5% grade.^13 , 152^

Regardless of the ergometer and protocol, after complete cessation of effort, the patient must remain seated until he or she returns to near-baseline condition (or for at least 6 minutes). If symptoms arise during the test and/or the patient is unable to complete active recovery, quickly stop the ergometer and place the patient in the supine position.

## 2. Test Accuracy, Pretest Probability, and Pretest Scores

The diagnostic accuracy of ET/CPET varies according to the investigated condition and its prevalence, the clinical characteristics of the patient, age, and sex. Based on this information, one can select which patients will benefit most from ET/CPET for diagnostic purposes, avoiding unnecessary investigations and interventions.^277^

### 2.1. Pretest Probability of CAD

The probability of having CAD varies with age, sex, symptom profile, risk factors, and lifestyle. Use of one of several risk estimation tables based on classification of precordial pain by sex and age (Tables 26 to 28) is recommended for definition of pretest probability. Notably, the frequency of CAD increases with age.

ET is more useful for diagnosing CAD in patients with an intermediate pretest probability (defined as 10% to 90% in Table 26, or 25% to 75% in Table 28), as this is the population in which findings will have the greatest impact on the clinical decision.^6 , 320^

### 2.2. Sensitivity, Specificity, and Predictive Value

The performance of diagnostic tests, including ET, can be evaluated by calculation of sensitivity, specificity, positive and negative predictive value, and accuracy (Figure 3).

For the investigation of CAD, most studies have reported a sensitivity of ET between 61% and 73% (mean, 69%) and a specificity between 69% and 81% (mean, 75%). This variability is associated with differences in methodology and among studied populations.^6 , 323^

The positive predictive value of ET is always higher in men, across all age groups, due to their higher prevalence of CAD.^324^

Women have a lower prevalence of CAD in general and, therefore, a greater prevalence of false-positives tests than men.^325^In women, age has a greater influence on PPV: it is much lower in the young (age 35 to 50 years = 36%) compared to older women (age >65 years = 68%).^326^

In a cohort study comparing ET in both sexes, among women, the use of HRmax, duration of exercise, and ST-depression recovery time provided significant increases in PPV (from 47.8 to 61.5%) and NPV (88%).^327^

### 2.3. Pretest CVD Scores and Risk Factors

The administration of cardiovascular disease risk scores before ET aims to provide an individualized approach, predict possible complications (especially in intermediate and high-risk patients), and, consequently, allow a more contextualized analysis of ET findings. This assessment is recommended in adults and the elderly (typical age group: 40-75 years), and should be based on one of the following scores: European Society of Cardiology Systematic Coronary Risk Evaluation (SCORE2), American College of Cardiology/American Heart Association Atherosclerotic Cardiovascular Disease (ASCVD) algorithms, or Framingham Risk Score.^328 - 330^

In young adults (<40 years), evaluation and recording of classic risk factors – diabetes, smoking, dyslipidemia, stress, sedentary lifestyle, obesity, hypertension, and family history – are suggested.^331^

## 3. Clinical and Hemodynamic Responses to Exercise in the Adult Population

### 3.1. Clinical Responses

#### 3.1.1. Exercise Tolerance

Exercise tolerance is recognized as the current best marker of life expectancy, regardless of age, sex, ethnicity, and comorbidities. During ET, it can be quantified objectively by the power generated in watts, by the duration of exercise, or by the metabolic equivalents of task (METs).^332^

Exercise tolerance can be quantified subjectively using the Borg or modified Borg Rating of Perceived Exertion scales (Figure 4), to measure the level of intensity of physical activity, tiredness, severity of dyspnea on exertion, and lower-limb fatigue.^333 , 334^

Regardless of the presence of CVD, low exercise tolerance is associated with higher mortality rates and increased incidence of HF and CAD.^332^

Exercise intolerance (EI) is defined as impaired ability to perform physical activities due to the presence of symptoms such as dyspnea and/or fatigue.^335^

In both HFrEF and heart failure with preserved ejection fraction (HFpEF), EF is associated with worse quality of life, more frequent hospitalizations, and increased all-cause mortality.^116 , 336^In chronic diastolic heart failure, one of the main mechanisms of EI is chronotropic incompetence, which can be assessed by ET.^337^

In type 2 diabetes mellitus (T2DM), EI is also associated with chronotropic incompetence, both of which are associated with an increased risk of CVD and premature death.^338^

Figure 5 illustrates the main mechanisms and factors that contribute to the emergence and progression of EI.

ET and CPET are essential methods for diagnosing EI and monitoring physical training programs, aiming to document improvements in exercise tolerance and cardiopulmonary performance and consequent reductions in morbidity and mortality.^339 , 340^Physical training seems to be one of the only potentially effective and viable interventions for improving EI.^338 , 340^

#### 3.1.2. Cardiorespiratory Fitness/Functional Classification

Cardiorespiratory fitness (CRF)/functional classification by ET involves stratification of physical performance based on oxygen consumption, or uptake (VO_2_).

Maximal oxygen consumption (VO_2_max) expresses the highest amount of oxygen extracted from inspired air during dynamic exercise involving a large muscle mass. In submaximal ET, the highest VO_2_value obtained is more properly called the VO_2_peak.^13 , 134 , 293^

ET is considered maximal when:^4^

– Signs or symptoms of physical exhaustion develop.

– The patient is unable to continue.

– A Borg rating ≥18 is achieved.

– HR does not increase further even with increasing exercise intensity.

– The predicted HRmax is reached (always taking the previous items into account) or exceeded (≥110%).

In conventional ET, the VO_2_is estimated through formulas (indirect measurement), while in CPET, it is measured directly. Indirect measurement tends to overestimate VO_2_values due to the limitations of the original studies that generated the formulas.^341 , 342^

Several formulas (“classic” and “novel”) are available for calculating predicted VO_2_peak, based on the type of ergometer, sex of the patient, protocol, whether the handrail was used for support, etc. ( Annex 2 ).^293 , 343 - 348^

VO_2_is most often expressed in mL/kg/min (mL.kg^- 1^.min^- 1^is also acceptable) It can also be expressed in MET and each MET corresponds to a VO_2_of 3.5 mL/kg/min.^6^


$$MET=\frac{{VO}_{2}mL/kg/min}{3.5mL/kg/min}$$

In the general population, VO_2_measured on a cycle ergometer is usually lower than on a treadmill. VO_2_max shows a progressive decline (on average, 8% to 10% per decade) after the age of 30. At age 60, the mean VO_2_max in men is approximately two-thirds that measured at age 20. When compared to men, women tend to have a lower VO_2_max due to lower hemoglobin, blood volume, stroke volume, and muscle mass.^13 , 134^

The New York Heart Association (NYHA) functional classification is used for diagnostic and prognostic purposes, contributes to therapeutic decision-making and exercise prescription, and is associated with VO_2_/METs achieved in ET (Figure 6).^6^

#### 3.1.3. Symptoms

Symptoms during ET must be described in detail and correlated with clinical signs, hemodynamic response, and ECG findings. Any symptoms that lead to test cessation during the exercise phase must be described alongside the respective rating of perceived exertion (Borg or modified Borg) and the achieved performance (work load/METs).

The use of scales to quantify any symptoms of angina, dyspnea, and intermittent claudication (especially during CPET) is suggested. These scales must be visible to the patient and clearly explained to them before starting the test (Figure 7).^13 , 292 , 349 , 350^

Chest pain should be described in terms of character, location, irradiation, aggravating/alleviating factors, duration, other concomitant symptoms, and the timing of pain onset and resolution during the ET. Clinicians are also advised to describe whether the pain was test-limiting; the initial HR, SBP, and double product; and respective ECG findings.

The occurrence of typical angina (i.e. progressing with increasing exertion) is by itself considered consistent with an ischemic response.^6^

In a study of 10,870 patients with symptom-limited ETs, typical angina pectoris was associated with an increased risk of mortality (RR: 2.7; 95% CI: 1.4-5.1; p<0.002) compared to non-anginal chest pain.^351^

The occurrence of dyspnea during ET is associated with a significant increase in all-cause mortality, but it is not associated with higher rates of ischemia compared to chest pain.^352^

In suspected PAD and evaluation of intermittent claudication, use of an intermittent claudication pain scale to quantify pain and its severity is recommended. Ideally, ET should continue until pain becomes intolerable and/or the patient can no longer walk (absolute claudication).^353^

The intermittent claudication scale ranges from 0 to 4 (see Figure 7) and can be used to quantify the progression of claudication during ET. The respective work load, duration of exercise, and the time point of pain resolution should be recorded.^175^

All other test-emergent symptoms (i.e. vertigo, dizziness, presyncope, headache) must be recorded, as well as the timing of occurrence, severity, duration, BP and HR at the time of onset, and any other information deemed useful to allow appropriate interpretation.

#### 3.1.4. Inspection and Auscultation

Sweating and facial flushing are to be expected during intense physical exercise. Onset of skin pallor, accompanied or followed by diaphoresis, cyanosis, or tachypnea, denotes a pathological condition. Occurrence of any of these at low work loads and/or in combination with hemodynamic instability or ECG changes (ST deflection and complex arrhythmias) denotes even greater severity.^1^

Auscultation should be performed and compared before and after exercise in order to correlate it with any observed clinical signs. The presence of wheezing, rhonchi, or crackles before exercise may contraindicate the performance of ET; after exercise, they may indicate exercise-induced asthma or ventricular dysfunction.^354 , 355^

On auscultation of new murmurs and/or worsening of pre-existing murmurs during exertion or recovery, their relationship with the cardiac cycle, location, quality or tone, intensity, and presence of ejection clicks should be described and recorded.^356 , 357^

Exertion usually increases the intensity of regurgitant murmurs originating from the left heart chambers (mitral and aortic regurgitation).^92 , 94^

Appearance of S3 on exertion in men over 40 years of age, in the presence of dyspnea or excess fatigue, may be associated with ventricular dysfunction.^358^In adults and the elderly, the appearance of S3 soon after cessation of exercise is often associated with ventricular dysfunction, previous MI, and left bundle branch block.^359^

Adults and young athletes usually have S3 on baseline auscultation. If it appears during exercise in these populations, it is considered a physiological adaptation, and does not correlate with structural heart disease.^360^

## 3.2. Hemodynamic Responses

The following concepts are important to understanding the hemodynamic response to increasing exertion during ET:^13 , 134 , 293^

– Dromotropic reserve: increased speed of cardiac electrical impulse conduction.

– Inotropic reserve: capacity to increase ventricular function (efficiency of ventricular filling and emptying); evaluated by the behavior of SBP.

– Peripheral arterial resistance: capacity of peripheral vessels to adapt (vasodilate/vasoconstrict); evaluated by the behavior of DBP.

– Coronary reserve: capacity of the coronary network to adapt to increased blood flow in response to increased myocardial metabolic activity.

– Cardiac reserve: capacity of the heart to increase its output to compensate for the greater metabolic demand of the exercising skeletal musculature. It is influenced by all of the above parameters.

### 3.2.1. Heart Rate

Assessment of HR behavior allows evaluation of chronotropic response, chronotropic reserve, double product, and autonomic regulation, making it an important diagnostic and prognostic parameter.

**3.2.1.1. Resting Heart Rate**

The normal resting HR (RHR) range is 50 to 99 bpm, as measured by ECG at rest (sitting or supine). In the context of ET, RHH is a valuable parameter for the interpretation of HR response, autonomic regulation, and prognosis.^361 , 362^

It is predictive of CAD, HF, AF, stroke and is associated with an increased risk of cardiovascular events, cardiac death, and death from all causes.^363 - 365^

Research has shown that, during ET, an RHR ≥80 bpm in the general population and ≥75 bpm in diabetics with stable CAD is associated with increased all-cause mortality.^366^

In a cohort of 56,634 individuals (49% women) without known CAD or AF, RHR ≥90 bpm was associated with a significant increase in all-cause mortality; in men, this was independent of physical fitness.^367^

**3.2.1.2. Chronotropic Response**

The normal chronotropic response to exercise consists of an increase in HR due to a decrease in vagal tone, followed by an increase in sympathetic tone and consequent adaptations of systemic vascular blood flow.^368^

The increase in HR follows the increase in work loads and usually presents a linear correlation with VO_2_alone between 50 and 90% of VO_2_max. In healthy adults, HR normally increases at a rate of ≈10 bpm per MET.^134^

Vagal withdrawal is responsible for the initial increase of 10 to 30 beats per minute (bpm), with further increments usually mediated by sympathetic activity. HR is responsible for most of the increase in cardiac output during exertion, particularly at higher loads (Figure 8).^134 , 299^

HR recovery (HRrec) during the first 30 to 60 seconds after exertion involves reactivation of the parasympathetic system and progressive inhibition of sympathetic activity. HRrec can also be influenced by the degree of venous return and the atrial baroreceptor response. Delayed HRrec is an important marker of autonomic dysfunction and risk of mortality (CV and all-cause) and major adverse cardiovascular events (MACE).^369^

The estimated maximum heart rate should not be used as the sole criterion for ET cessation or to assess the effectiveness of ET. There is generally a wide margin of error (wide dispersion of the mean) in formulas used to estimate this parameter, ranging from ±10 to 15 bpm. Women tend to have a lower estimated maximum heart rate.^301 , 370^

Evaluation of chronotropic response through the estimated maximum heart rate is limited in patients on medications that interfere with autonomic modulation (antiarrhythmics and beta-blockers), in AF and atrial flutter, and in patients with rate-responsive pacemakers, ICDs, congenital AV block, etc. Major factors that interfere with the HRmax achieved during ET are listed in Table 29.

Table 30 lists variables which measure the HR response to ET, while Table 31 gives their definitions, criteria, and interpretations.

Particular Features of Chronotropic Response:

– In a cohort of 458 men (age 56±8.5; mean follow-up: 6 years) with CAD, an increase in HR during the first minute of the exercise phase of ET ≥12 bpm was strongly associated with cardiac death (RR: 15.6; 95% CI: 2.0-118.7; p<0.001) and nonfatal MI (RR: 5.0; 95% CI: 2.7-9.1; p<0.0001).^379^

– In a follow-up study of 306 patients with asymptomatic aortic stenosis (age 65±12 years, 33% women; mean follow-up duration 25 months), among severe cases, rapid early rise in HR (defined as reaching 85% HRmax or increase ≥ 50% HR at 3.5 METs) was associated with need for valve replacement (RR: 3.32; 95% CI: 2.03-5.45; p<0.001).^374^

### 3.2.2. Blood Pressure Response

Assessment of the blood pressure response (BPR) to exercise is an important diagnostic, prognostic, and cardiovascular risk stratification tool. BPR essentially depends on cardiac output, peripheral vascular resistance, and medication use.^6 , 380^

In healthy adults, the increase in systolic blood pressure (SBP) is expected to be proportional to the increase in dynamic exercise load (corresponding to the inotropic response). As work load is maintained, the SBP stabilizes within 2 to 3 minutes.^380 , 381^The average increase in SBP is usually 10 mmHg/MET. In individuals who reach an exercise load >10 METs, the SBP increase is 6.2 mmHg/MET.^6 , 382^After cessation of exertion, SBP tends to decrease gradually due to the vagal response and rapid reduction in cardiac output. It generally returns to resting levels within the first 6 minutes of recovery, and may remain below pre-exercise levels for several hours (Figure 9).^383^

During exertion, diastolic blood pressure (DBP) remains unchanged or may show minor fluctuations (±10 mmHg) due to the drop in peripheral arterial resistance. Occasionally, in healthy individuals, DBP sounds can be heard down to 0 mmHg during exercise without any pathologic significance. In such cases, use of phase IV Korotkoff sounds (abrupt muffling of sounds, which then become softer) to define DBP is recommended. At the onset of recovery, DBP may increase slightly or remain unchanged. It generally returns to resting levels within the first 6 minutes of recovery.^384^

ET should not be performed in patients with a resting SBP ≥180 and/or DBP >110 mmHg. When measuring with an aneroid sphygmomanometer, round values to the nearest 5 mmHg. When using automated BP devices, consider the actual absolute values.^4 , 6 , 13^

A hypertensive/exaggerated SBP response to exercise is defined as a maximum value ≥210 mmHg for men and ≥190 mmHg for women, regardless of protocol and/or ergometer.^85 , 385 - 387^In subjects who are normotensive at baseline, such an SBP response is associated with increased risk of future hypertension and cardiovascular events (Table 32).^388 , 389^

Peak SBP on exertion becomes gradually higher values with advancing age. It is generally lower in women than in men, a difference that tends to disappear in older adults. Young women may present a plateau SBP response or even a slight drop in SBP at peak exercise, with no specific clinical significance.^6 , 87 , 390^

The DBP response is considered abnormal when there is a ≥15 mmHg increase and/or it exceeds >90 mmHg, considering normal pre-ET DBP values at rest.^391^There is strong scientific evidence from Brazilian and international studies validating these criteria for normal SBP and DBP response to ET for the diagnosis of hypertension, assessment of treatment efficacy, association with CVD, and risk stratification.^391 - 393^

A hypertensive response to exercise (HRE) is more common in older adults and in individuals with hypertension, even when BP is well controlled at rest.^394^In hypertensive patients, HRE is associated with a higher risk of future HF, LV hypertrophy, endothelial dysfunction, diastolic dysfunction, and cardiovascular events.^392 , 395 , 396^

In the absence of systemic arterial hypertension or other cardiovascular diseases, HRE is not a benign phenomenon; its main causes are endothelial dysfunction and stiffness of the major arteries. HRE is usually associated with functional and structural LV abnormalities, especially when accompanied by a rise in central BP.^72 , 305 , 387 , 397^

Paradoxically, studies in patients with suspected or established CAD have shown that the occurrence of HRE was associated with less severe coronary lesions and lower mortality compared to those who had a normal BP response.^398 , 399^

It is suggested that BP measurement be performed upon reaching a work load of 5 METs. SBP ≥150 mmHg is a discriminatory threshold for hypertension, alongside systolic hypertension on 24-hour ABPM and LV hypertrophy on echocardiography.^400 , 401^

In a prospective cohort of 6,578 asymptomatic participants from the Lipid Research Clinics Prevalence Study (mean age 46 years; 45% women; mean follow-up duration, 20 years), among individuals who were normotensive or prehypertensive at rest, BP >180/90 mmHg during the second stage of the Bruce protocol was associated with risk of CVD mortality (SBP RR: 1.96; 95% CI: 1.40 to 2.74; p<0.001/DBP RR: 1.48; 95% CI:1.06 to 2.06; p=0.02).^75^

Pre-ET hypertension with a normal BP response to exercise is common in anxious patients, and is generally not associated with future development of hypertension.^215 , 402^

SBP >250 mmHg and DBP ≥120 mmHg in normotensive individuals, or DBP ≥140 mmHg in hypertensive individuals, are considered criteria for test cessation.^4 , 6 , 13^

Any occurrence of hypotension/drop in BP during exercise, a rare event (incidence <2% to 6%), requires immediate intervention (cessation of exercise and placement of patient in the supine position) for safety reasons, as it portends acute risk of a MACE. The most common causes are: severe multivessel CAD with LV dysfunction; cardiomyopathies; LV outflow tract obstruction; increased vagal tone; hypovolemia; and arrhythmias. Its PPV is higher in men than in women (Figure 10).^403^

Hypotension and syncope during recovery may occur due to a variety of causes, ranging from physical exhaustion in an apparently healthy individuals to abnormal, pathologic responses, such as LV outflow tract obstruction (i.e. in AS and obstructive hypertrophic cardiomyopathy); dysautonomia, and negative vagal baroreflex regulation (see Figure 10).^74 , 386 , 404 , 405^

Table 32 presents definitions of key terms, normal values and ranges, interpretation criteria, and implications regarding the BP response during ET.

### 3.2.3. Double Product

The double product (DP), or rate pressure product, expresses myocardial oxygen consumption (linear relationship with myocardial oxygen uptake and coronary blood flow). It is calculated by multiplying the HR by the SBP at any time during ET.^6 , 134 , 423^


DP=HR×SBP(bpm.mmHg)

DP is an important parameter in the assessment of anginal thresholds, ECG changes (ST-segment deviation and arrhythmias), cardiovascular efficiency, progression of cardiorespiratory fitness, and response to pharmacologic and interventional therapy. It has prognostic value independently of the presence of CAD and, therefore, serial measurement is recommended. A maximum DP (usually obtained at peak exertion during ET) <25,000 bpm.mmHg is associated with worse prognosis.^115 , 134 , 423 , 424^

Factors that limit DP assessment include beta-blocker and antiarrhythmic therapy; uncontrolled hypertension; and atrial fibrillation and flutter with uncontrolled ventricular rate. It should not be calculated during tachyarrhythmias.^134 , 293 , 299^

## 4. ECG Responses 371

For proper analysis, description, and interpretation of ECG responses to ET, the following factors should be taken into account:

– Check proper electrode placement and attachment to minimize errors and artifacts.^425 , 426^

– Follow the standard ECG reporting guidance of the Brazilian Society of Cardiology Guidelines on the Analysis and Issuance of Electrocardiographic Reports – 2022.^295^

– Use automated measurement systems for intervals, durations, and amplitudes of ECG waves and segments.^427^

– Consider the effects of any ECG filters applied (high, medium, low) for baseline stabilization and reduction of muscle and electrical artifacts. Use high-pass filters with a cutoff of at least 150 Hz in adults and adolescents. Filters with lower frequencies may interfere with capture of pacemaker spikes.^295 , 428 , 429^

– Review any automated measurements to rule out errors due to possible interference, artifacts, or abnormalities in the underlying tracing.^427 , 430^

– Provide a detailed, contextualized description in the ECG record.

### 4.1. P Wave

#### 4.1.1. Normal Responses

In adults, P waves on a resting ECG – in the presence of sinus rhythm – are positive in leads D1, D2, and aVF, with a mean electrical axis vector of 60° (range, 0° and 90°), maximum amplitude of 250 mV (2.5 mm), and duration ≤110 ms.^295^

During exercise, the following are normally observed (Figure 11):^431^

– Gradual, linear increase in P wave amplitude as HR increases (in inferior leads in the frontal plane). This increase is, on average, 100 mV (1 mm).

– Preservation of the axis vector of the P wave.

– No change or, rarely, a minimal increase (≤20 ms) in duration.

Normal P wave response to recovery:

– In the first minute of recovery, there may be an additional increase in P wave amplitude (exceeding than seen during exertion), even as the HR declines. A progressive reduction in amplitude occurs thereafter, returning to baseline after the 6th minute.^432^

– P wave duration remains unchanged or, rarely, increases minimally (≤20 ms) until the 3rd minute.^433^

#### 4.1.2. Abnormal Responses

The main abnormal P wave responses to exercise are:^431 , 434 , 435^

1) No change in P wave amplitude from baseline, especially regarding the negative component (change <0.25 mm). This increases sensitivity (69%) and specificity (78%) for the diagnosis of CAD.

2) Increased P wave duration. Is associated with left atrial pressure overload during exercise-induced ischemia.

3) Change in P wave morphology with increase in the amplitude of the terminal negative component in lead V1, at 50% of the maximum exercise time, is the single ECG change most predictive of CAD verified by myocardial perfusion imaging.

4) Dispersion of P wave duration combined with ST-segment depression increased ET sensitivity to 79% and PPV to 91%.^436^

## 4.2. PR Interval/Segment

### 4.2.1. Normal Responses

On a normal resting ECG, the PR segment (PRs) begins at the end of the P wave, ends with the beginning of the QRS complex, and is isoelectric. It serves as a bridge between atrial activation and ventricular activation, as well as atrial recovery, which is usually of very low amplitude and difficult to detect, as it occurs within the QRS. When sufficient amplification is used or in the presence of first-degree AV block, the atrial repolarization wave (Ta wave) can be visualized. The interval from beginning of the P wave to the end of the Ta wave is equivalent to the atrial QT interval.

The PR interval (PRi; start of P wave to start of QRS complex) normally measures 120 to 200 ms and is best determined in lead II.

The PRs serves as a baseline demarcation point for the assessment of changes in the ST-segment and wave amplitude. The line joining the PQ junctions (end of the PR segment and beginning of the QRS complex) is defined as the “baseline”, considering at least four successive complexes, at the same horizontal level and without artifacts. Computerized ECG analysis systems use automated algorithms which consider the end of the PRs as the isoelectric baseline.^1 , 295^

During exertion, the PRs shortens and slopes slightly downwards in the inferior leads, while the PRi shortens to an extent directly proportional to the increase in HR.^437^

During the initial phase of recovery, the PRi and PRs are dependent on the degree of physical fitness of the patient. In sedentary men, the mean PRi is ≈110 ms; in athletes, ≈280 ms.^438^

### 4.2.2. Abnormal Responses

The main abnormal PRs responses during ET are:

– Even in normal individuals, a greater downward slope of the PRs may occur, attributable to exuberant atrial repolarization (negative Ta wave). If it persists in the initial period of ventricular repolarization, it will cause depression of the J point and ST-segment (upsloping; false-positive if in the inferior leads).^152 , 439^

– Exercise-induced atrial infarction: PRs depression >0.5 mm, usually with ST-segment elevation (denoting associated ventricular infarction) and atrial arrhythmias.^295 , 440^

– Pathologic PRi prolongation will be addressed in the section on atrioventricular blocks.

## 4.3. Q Wave

### 4.3.1. Normal Responses

On resting ECG, Q waves are considered normal when their duration is ≤30 ms and their amplitude is <0.2 mV (up to 0.4 mV in adolescents), which corresponds to <25% of the subsequent R wave. In lead III, Q wave duration can exceed 40 ms, but rarely reaches 50 ms. The presence of a Q wave in lead V1 is always pathological.^295 , 371^

During exercise, the Q wave normally increases significantly in amplitude, particularly in CM5 configuration and in the lateral leads.^441^

### 4.3.2. Abnormal Responses

On resting ECG, the Q wave is considered abnormal when, in the absence of bundle branch block and/or a preexcitation syndrome, its duration is ≥40 ms and/or amplitude is greater than one-third of the adjacent R wave (in two or more leads from the same ventricular wall).

The main abnormal Q wave responses during ET are:

– Reduction in amplitude/disappearance of the Q wave during exercise or recovery, which may indicate septal ischemia and which, when associated with ST-segment depression, increases the PPV for CAD.^441 , 442^

– Increased Q wave amplitude in the presence of ST-segment depression reduces the PPV, increasing the possibility of false positives.^443^

– Exercise-induced increase in Q wave duration (10±13 ms) in patients with single-vessel CAD and recent MI is associated with ischemia on thallium perfusion scan.^444^

– Transient Q waves can be seen in hypoglycemia, hyperkalemia, and asthma.

## 4.4. R Wave

### 4.4.1. Normal Responses

On resting ECG, the duration of R waves will depend on the lead and duration/pattern of the QRS complex. R-wave amplitude is variable; it increases progressively in the precordial leads and is generally <27 mm in V5 and V6.^445^

During exercise, the following phenomena usually occur:

– Reduction (2.6±1.1 mm) in average R-wave amplitude as maximal exertion is approached.^446^A marked reduction is observed in the lateral leads (V5 and V6) at maximal exercise and in the first minute of recovery.

– As R waves decrease in amplitude, an increase in S waves is observed.^115^

### 4.4.2. Abnormal Responses

On resting ECG, patients with anterior wall MI exhibit reduced R-wave amplitude or absent R waves altogether.^447 , 448^

The main abnormal R wave responses during ET are:

– Increase in R-wave amplitude resulting from changes in LV dimensions and myocardial ischemia.^449^The presence and extent of reversible ischemia in patients with CAD correlates directly with increased amplitude of the R wave and ST-segment depression.^450 , 451^

– R-vave amplitude increases significantly in the precordial leads during episodes of transmural ischemia.^452^

– Low-amplitude R waves (<10 mm) may occur with ST-segment depression of limited magnitude in relation to actual ischemia, thus interfering with the accuracy of ET for diagnosis of CAD.^453 , 454^

## 4.5. S Wave

### 4.5.1. Normal Responses

On resting ECG, the S wave is commonly observed in leads I, II, III, aVF, V1, and V2 (where its amplitude is greater than that of the R wave); it is usually absent in V5 and V6. Under normal conditions, the S wave amplitude is <0.3 mV (30 mm).^445^

During ET, in the absence of intraventricular conduction disorders, the S wave usually:

– Tends to increase amplitude in inferolateral leads (particularly in aVF and V5; maximum 0.3-0.4 mV) with concomitant reduction of the R wave.^115 , 455^

– In the first minute of recovery, the S wave amplitude remains unchanged or is slightly reduced. It returns to pretest range at 3 to 5 minutes of supine recovery.^455^

– The mean S wave duration shows a progressive reduction with increasing effort, regardless of intraventricular conduction disorders.^456^

### 4.5.2. Abnormal Responses

The major abnormal S wave responses to exercise are:

– Decreased S waves amplitude in patients with CAD are associated with subendocardial ischemia, even in the absence of ST-segment changes, increasing ET sensitivity.^457^

– In patients without intraventricular conduction disorders, unchanged S wave duration is usually associated with significant right coronary artery (RCA) or circumflex artery obstruction.^456 , 458^

– A significant increase in duration (≈12.5±6 ms) is observed in patients with critical left anterior descending artery (LAD) obstruction, left anterior fascicular block, and right bundle branch block.^456^

## 4.6. QRS Duration

### 4.6.1. Normal Responses

The duration of QRS complexes generally decreases proportionally to the increase in HR during exercise (decrease ≈3.0 to 4.9 ms). Exceptionally, in healthy patients, there is no such change in duration.^459^

The variance in QRS complex duration (ΔQRSd) is a parameter that corresponds to the difference in QRS duration measured in aVF and V5 immediately upon cessation of effort (onset of recovery) as compared to the resting condition.^459^The normal ΔQRSd is ≤3 ms.^460^

ΔQRSd = QRS duration at onset of recovery – QRS duration at rest*

*Measured in leads aVF and V5, expressed in milliseconds.

### 4.6.2. Abnormal Responses

Significance of ΔQRSd changes:

– A ΔQRSd >3 ms is considered positive for ischemia.^460^In patients with CAD, this parameter is directly and significantly associated with the number of arteries with significant obstruction (4.8 ms = one artery, 7.8 ms = two arteries, 13.3 ms = three arteries; p<0.001) and regional wall motion abnormalities on radionuclide ventriculography (6.7 ms = one LV region, 13.5 ms = two LV regions, 21 ms = three regions; p<0.0001).^461^

– A ΔQRSd >3 ms improved the diagnostic accuracy of ET for CAD, regardless of ST-segment changes, compared to myocardial perfusion scan (sensitivity 93%, specificity 71%, PPV 86%).^459^

– A ΔQRSd >3 ms in women is more sensitive and specific than ST-T changes for the detection of ischemia.^462 , 463^A previous study showed that abnormal ΔQRSd in women increased sensitivity (91%), specificity (89%), and PPV (88%) of exercise test; in younger women (age 27-50 years), sensitivity was 80% and specificity 83%.^462^

– A ΔQRSd ≥15 ms in patients with ischemic heart disease predicted occurrence of severe ventricular arrhythmias (ventricular tachycardia or fibrillation) with a PPV of 73%. After surgical revascularization, these patients no longer had an abnormal ΔQRSd and arrhythmias were suppressed.^464^

## 4.7. High-frequency Assessment of QRS Fragmentation

### 4.7.1. Normal Responses

High-frequency assessment of QRS fragmentation, or high-frequency QRS (HF-QRS), is a special ECG filtering technique (usually between 150 and 250 Hz) that allows analysis of the high-frequency components of QRS complexes.^465^

The technique involves combining QRS complexes from the same lead (from a full 12-lead recording) or complexes from four leads (V3, V4, V5, and V6) to form a “precordial average complex”. The normalized root mean square (NRMS) and/or normalized peak amplitude (NAMP) of these complexes are then calculated. These parameters are generally evaluated on the resting ECG, at peak exertion, and during recovery.^466^

In healthy people, the HF-QRS is considered normal when the average NRMS values are >1 μV in all three stages of the ET (rest, peak exertion, and recovery) and an increase in NRMS is observed during and immediately after exercise (compared to the resting ECG).^466 , 467^

### 4.7.2. Abnormal Responses

The HF-QRS is considered abnormal (positive for ischemia), regardless of changes in the ST-segment, when: there is an absolute reduction ≥1μV or a relative reduction ≥50% between the maximum and minimum NRMS values in ≥3 leads in patients who reached maximum exertion. In submaximal tests (≤85% of the predicted maximum HR), the relative reduction must be adjusted linearly, between 40% and 50%, according to the ratio between the actual maximum HR achieved and the predicted HR. HF-QRS has greater sensitivity, specificity, and PPV than ST-segment changes.^468 - 470^

Abnormal HF-QRS is usually found in post-MI patients with myocardial scarring, ischemia, or conduction delay due to non-homogeneous ventricular activation. It is a predictor of mortality and cardiac events in patients with CAD.^467 , 468^

Abnormal HF-QRS demonstrated incremental diagnostic value when associated with ST-segment changes (PPV 80.4% *vs.* 74.9%; p<0.0001). Joint analysis of HF-QRS and ST-segment changes identified 92.3% of individuals with significant ischemia.^471^

Analysis of both HF-QRS and ST-segment depression demonstrated a 99% NPV for severe ischemic disease. On multivariate regression, abnormal HF-QRS was an independent risk factor for MACE at 2 years (RR: 2.8; 95% CI: 1.7-4.4; p<0.001).^472^

## 4.8. T Wave

### 4.8.1. Normal Responses

On the resting ECG, the T wave is rounded, asymmetrical, with a slower initial upsloping portion and a faster downsloping final portion, usually with the same polarity as the QRS and positive in almost all leads (always negative in aVR). Its duration ranges from 100 to 300 ms, and its amplitude is a maximum of 5 mm in peripheral leads and <15 mm in precordial leads, corresponding to approximately 10% to 30% of the total amplitude of the QRS complex that precedes it.^473 , 474^

In the initial phase of exercise, there is a general decrease in T wave amplitude followed by an increase at higher exercise loads (returning to baseline), with a further increase in the initial phase of recovery.^455^

### 4.8.2. Abnormal Responses

When interpreting T wave changes, pre-existing diseases (especially hypertension, valvular heart disease, and CKD), risk factors and the pretest probability of CAD should be considered.

On resting ECG, the main T wave changes that may be associated with ischemia are:

– Positive, symmetric, pointed T waves followed by a U wave (positive or negative).^475^

– Negative, symmetric, pointed T waves in leads with a predominantly positive QRS (except III, aVR, and V1).^115^

– Biphasic pattern in the anterior thoracic leads (V1 to V3), usually associated with myocardial ischemia in unstable angina.

– Flat (usually nonspecific; may be associated with myocardial ischemia).

Abnormal T wave responses to exertion:

– Increase in amplitude (>2.5 mV), symmetric, in leads V2 to V4: in patients with chest pain, is associated with severe ischemia.^115^

– T wave pseudonormalization, corresponding to the presence of an inverted T wave ≥1 mm in any lead at rest which becomes positive at peak exertion; is generally associated with reversible, fixed defects on myocardial perfusion scintigraphy.^476^

– T wave pseudonormalization associated with U wave inversion in anterior thoracic leads is highly indicative of critical LAD stenosis.^477^

– T wave pseudonormalization in leads related to previous MI at low work loads has shown to be a sensitive, specific indicator for the presence of residual myocardial viability. Sensitivity and accuracy were higher for prior MI.^478 , 479^

– In submaximal ET, performed in the early phase of stabilized unstable angina or post-MI without Q waves, T wave pseudonormalization (regardless of the occurrence of ST-segment depression) was predictive of survival at 6-month follow-up.^480^

– T wave pseudonormalization in populations with a low prevalence of CAD is a nondiagnostic finding.^152^

## 4.9. U Wave

### 4.9.1. Normal Responses

On resting ECG, the U wave is a low-amplitude, low-frequency deflection that follows the T wave (usually with the same polarity), with an amplitude proportional to that of the T wave (≈5% to 25%; mean: 0.33 mm). It is best observed in leads V2 and V3 (≤2 mm), has a mean duration of 221±73 ms (<50% of the preceding T wave), and is most commonly identified at HRs <95 bpm. It is seen in up to 50% of normal individuals. During exercise, duration, amplitude, and axis generally remain unchanged, but U waves become difficult to identify at HR >120 bpm due to encroachment of the T and P waves.^481 - 483^

### 4.9.2. Abnormal Responses

A negative U wave on resting ECG is considered abnormal and is often associated with mitral and/or aortic regurgitation, hypertension, and ischemic heart disease.^484 , 485^Increased amplitude may be associated with hypokalemia and medications (digitalis, amiodarone, and quinidine).^483 , 484 , 486^

The main abnormal U wave responses during ET are:

– At baseline (positive), increased exertional amplitude (≥0.5 mV) in precordial leads is associated with inferoposterior myocardial ischemia, and is a marker of significant circumflex coronary artery (CX) or RCA obstruction.^487 , 488^

– Appearance of a positive U wave on exertion and/or during the first 3 minutes of recovery is often associated with CX or RCA obstruction.^489^

– Appearance of a negative U wave with amplitude ≥0.5 mV, persisting for at least 1 minute during and/or after exercise, is associated with severe LAD disease.^487 , 490^

– Appearance of a negative U wave is a marker of a well-developed collateral circulation in patients with severe CAD or stable angina.^487^In precordial leads, it is a marker of viable myocardium after MI.^491^

– Transient exercise-induced U wave inversion with amplitude ≥0.5 mV in anterior wall leads (V2 to V5), is associated with episodes of acute ischemia and severe LAD disease.^492 , 493^

## 4.10. Early Repolarization

Early repolarization pattern (IRP) is seen in 1% to 13% of the general population.^494 , 495^In middle-aged individuals, it has been associated with increased risk of sudden cardiac death (SCD).^496 , 497^

The criteria for diagnosis of early repolarization on resting ECG are:^498 , 499^

1) QRS complex duration <120 ms.

2) End-QRS notch or slur on the downstroke of a prominent R wave. If there is notch, it must be completely above the baseline. The point of J-wave onset (Jo) must also be above the baseline (Figure 12).

3) The peak of the J-point notch (Jp) must be ≥0.1 mV in two or more contiguous leads of a 12-lead ECG, except V1 through V3.^500^

ST-segment elevation should be measured 100 ms after the Jt point (termination of the J-point notch). In addition to the magnitude of elevation, the pattern should be described:

– “Early repolarization with upsloping ST-segment”, when the ST-segment is ascending (inclined upwards) and followed by a vertical T wave.

– “Early repolarization with horizontal or descending ST-segment”, when the ST-segment is horizontal or descending (inclined downwards).

Behavior and significance of ERP in ET:

– Common in young individuals. In this setting, usually reduces progressively with increasing exertion, and may disappear altogether at moderate loads. ERP with rapidly upsloping ST-segment elevation in the anterolateral leads has been reported in athletes.^501^

– Persistent ERP on exertion has been observed in symptomatic patients (aborted sudden cardiac death, sustained ventricular arrhythmia, and/or unexplained syncope).^502^

– During recovery, ERP resumes slowly, progressively, and, in approximately 30% of patients, occurs at the 5th minute.^503 , 504^

High-risk markers verifiable on ET in patients with ERP:^55 , 494^

– Exercise-induced polymorphic VT.

– Persistent horizontal and/or descending ST elevation during exercise in inferior or inferolateral leads is associated with risk of idiopathic VF and a threefold increase in risk of sudden arrhythmic death.^500^

## 4.11. ST-segment Elevation

Exercise-induced ST-segment elevation is defined as an ST-segment elevation ≥1.0 mm (≥0.10 mV) within 60 ms of the J-point, occurring in two or more leads, regardless of the presence of a Q wave (Figure 12). There may be reciprocal ST-segment depression (“mirror image”).^505 , 506^

Exercise-induced ST-segment elevation is generally associated with severe myocardial ischemia (usually transmural), coronary artery spasm, Prinzmetal angina, left ventricular aneurysm, peri-infarction ischemia, and LV wall motion abnormalities.^506 , 507^

The leads in which exercise-induced ST-segment elevation occur correlate with anatomic-vascular segments of the LV.^506 , 508 , 509^Use of Myers’ topographic description of ischemic manifestations is advised:^295^

– Anteroseptal wall: leads V1, V2, V3.

– Anterior wall: leads V1, V2, V3, and V4.

– Localized anterior wall: leads V3, V4, or V3-V5.

– Anterolateral wall: leads V4 to V5, V6, I, and aVL.

– Extensive anterior wall: V1 to V6, I, and aVL.

– Lateral wall: leads V5 and V6.

– High lateral wall: I and aVL.

– Inferior wall: II, III, and aVF.

The terms “posterior wall” and “dorsal” should no longer be used due to current evidence that leads V7 through V9 refer to the lateral wall.

Thirty percent of patients with a past anterior wall MI and 15% of those with prior inferior wall MI exhibit exercise-induced ST-segment elevation in the leads involved, and its significance varies depending on additional ET findings.^510 , 511^The finding of exercise-induced ST-segment elevation in leads with abnormal Q waves may represent residual ischemia of the peri-infarct area (myocardial viability), ventricular dyskinesia, or akinetic LV wall motion.^511 , 512^However, it does not allow quantification of viable tissue, justifying additional investigation by imaging methods to ascertain whether therapeutic intervention is indicated.^512 , 513^

Particular features of exercise-induced ST-segment elevation:

– Exercise-induced ST-segment elevation ≥0.2 mV (2 mm) in leads without Q waves mandates test cessation.

– Exercise-induced ST-segment elevation is more commonly associated with severe proximal obstruction than with coronary spasm in unobstructed arteries.^514 , 515^

– Exercise-induced ST-segment elevation, with or without concomitant depression, was predictive of the presence, extent, and location of myocardial ischemia assessed by myocardial perfusion scintigraphy. Exercise-induced ST-segment elevation in lead V1 with ST-segment depression in aVR and V4-V6 was associated with left main coronary artery (LMCA) or proximal LAD stenosis.^509^

– Exercise-induced ST-segment elevation in lead aVR showed 100% sensitivity to detect LMCA stenosis (specificity 33.5%) and 94.3% sensitivity for LAD stenosis (specificity 26.6%). The combination of exercise-induced ST-segment elevation in both aVR and V1 reduced sensitivity (74.4% and 65.9%) but increased specificity (68.5% and 64.4%).^508 , 516^

– Exercise-induced ST-segment elevation in lead aVR was associated with MACE in 33% of patients at 2-year follow-up.^517^

– In patients who have undergone successful single-vessel PCI without residual ischemia for treatment of MI, exercise-induced ST-segment elevation may be associated with impaired coronary microcirculation and lower myocardial viability.^518^

## 4.12. J Point and Upsloping Depression

The J point (where the end of the QRS joins the onset of the ST-segment) is usually depressed on exertion in the lateral wall leads, gradually returning to its pre-exercise tracing during recovery. J-point depression is more common in older adults and is generally not associated with CAD.^519^

Upsloping ST depression – J-point depression followed by rapidly ascending ST depression without Y-point depression (measured at 60 or 80 ms from the J-point) – is seen in 10% to 20% of normal subjects. It is not considered a diagnostic criterion for CAD (Figure 13).^519^

## 4.13. Slow Upsloping, Horizontal, and Downsloping ST-segment Depression

Exercise-induced ST-segment depression is the most frequent ECG manifestation of myocardial (usually subendocardial) ischemia. The diagnostic accuracy of exercise-induced ST-segment depression will depend on age, sex, clinical characteristics, pre-existing cardiovascular diseases, prevalence of CAD, intensity of exertion, and HR achieved.^277 , 453 , 520 , 521^

The following ST-segment changes during exercise and/or recovery are considered abnormal and suggestive of exercise-induced ischemia (Figure 13):

1) Horizontal (rectified) or downsloping (descending) depression ≥1 mm (measured at the Y point). Depending on the HR at the time of measurement, the Y point will be 60 or 80 ms from the J point (i.e. generally, in children/young people 60 ms and in adults/elderly 80 ms).^6 , 522 , 523^

2) Depression with slow upsloping morphology (apply pre-test CVD risk scores – see Section 2.3):

– ≥1.5 mm in individuals at moderate or high risk of CAD;

– ≥2 mm in individuals at low risk of CAD (measured at the Y point within 80 ms of the J point).^524 - 527^

Slow upsloping ST-segment depression during exercise has lower sensitivity, specificity, low PPV and, accordingly, yields more false-positive results compared to horizontal or downsloping depression.

Particular aspects regarding quantification and interpretation of exercise-induced ST-segment depression:

– Depression <1.0 mm does not meet criteria for myocardial ischemia (nonischemic).

– When there is ST-segment depression at rest, with the patient in the standing position, consider only any additional ST depression occurring during exercise.

– The presence of ST-segment depression ≥1.0 mm at rest weakens the association of any additional ST depression with obstructive CAD.^528^However, in patients under investigation for chest pain, the presence of ST depression at rest did not interfere with the diagnostic accuracy and sensitivity of ET.^529^

– Factors that influence the magnitude of exercise-induced ST-segment depression and CAD severity: pretest probability; exercise capacity; timing of onset; work load at onset; duration and number of leads with depression; timing of normalization during recovery. The lower the work load and DP at which ST depression appears, the worse the prognosis and the greater the probability of multivessel CAD.^522 , 523 , 530 , 531^

– In patients with early repolarization at rest, if exercise-induced ST-segment depression occurs, consider only those changes below baseline.

– In the presence of right bundle branch block, ignore secondary ST depression in leads V1, V2, and V3 regarding ischemia; analysis and interpretation of ST depression in other leads should follow the conventional pattern described in this section.

– Exercise-induced ST-segment depression with concomitant exertional angina or anginal equivalent increases the sensitivity of the ET; it is associated with severe CAD and worse prognosis.^532^

– Exercise-induced ST-segment depression ≥3 mm (0.3 mV) in addition to any resting depression in the presence of suspected or known CAD mandates test cessation.^6 , 209^

– Downsloping ST-segment depression is usually associated with more severe ischemia when compared to horizontal ST-segment depression.^533 , 534^

– Early normalization of abnormal ST-segment depression within the first minute of recovery has been associated with lower ischemic burden and higher probability of a “false-positive” ET for CAD.^535 , 536^However, persistence of ST-segment depression for >3 minutes after cessation of effort was associated with severe CAD. Recurrence of exercise-induced ST-segment depression after early normalization during recovery also indicates severe CAD.^4 , 299^

– Exercise-induced ST-segment depression observed exclusively during recovery has the same diagnostic and prognostic accuracy as that observed during exertion.^537 - 539^However, its occurrence exclusively in late recovery (after the 3rd minute) increases the probability of a false-positive test for CAD.^538^

Regarding ST-segment depression and risk stratification:

– Exercise-induced ST-segment depression in asymptomatic individuals of both sexes is generally associated with a greater probability of future coronary events (angina, MI, or cardiac death).^184 , 540 , 541^

– In a 19-year follow-up, exercise-induced ST-segment depression in patients with low physical capacity (<8 METs) yielded a relative risk of 4.8 (95% CI: 2.9-7.9; p=0.013) for sudden cardiac death.^541^

– In a 3.4-year follow-up of patients with resting ST-segment depression ≥1.0 mm, only additional exercise-induced ST-segment depression ≥2.0 mm had significant prognostic value for MI and sudden cardiac death.^542^

– In a prospective cohort of 11,605 patients (52.9% male, mean follow-up 6.7 years), the occurrence of exercise-induced ST-segment depression (horizontal or downsloping) was associated with a relative risk of 3.9 (95% CI: 2.7-5.7) of ACS in 1 year in those without typical angina, and a relative risk of 20.8 (95% CI: 13.9-31.3) in those with typical angina.^543^

– In a 5-year prospective follow-up of 366 peri/postmenopausal women (age 54.4±5.5 years) with low-to-intermediate Framingham Risk Scores, horizontal/downsloping exercise-induced ST-segment depression ≥1 mm was an independent risk factor for CV events (RR: 10.3; 95% CI: 1.9-61.4; p=0.007).^544^

Table 33 lists the main situations and conditions that interfere with assessment of ST-segment changes during ET regarding diagnosis of myocardial ischemia and CAD.

ET is considered nondiagnostic for CAD when 85% of the predicted HRmax is not achieved in the absence of ST-segment changes and/or angina (or anginal equivalent).^4 , 6^

**4.13.1. ST Hump Sign**

The “ST hump sign” (STHS), also known as convex ST-segment depression, is attributed to exaggerated atrial repolarization waves with upsloping ST depression followed by a hump-like wave after the J point.^545 , 546^In asymptomatic individuals with no known heart disease, it has been associated with resting hypertension and hypertensive response to exercise. It is probably a “false-positive” finding for obstructive CAD, with a good prognosis.^547^

Particular features of STHS:

– Has been associated with LV diastolic dysfunction.^546^

– Has been considered a risk factor for sudden cardiac death in hypertrophic cardiomyopathy (5.3 years of follow-up).^548^

– In a cohort of 81 patients with hypertrophic cardiomyopathy (mean age 42 years; 30% women; follow-up 5.3 years), was found in 52% of patients, with a CV mortality rate of 19%.^548^

– In a cohort of 237 nonconsecutive patients (59% men, mean age 41 years), of which 130 had a STHS, it showed a strong correlation with LV diastolic dysfunction (in 88% of patients).^546^

Additional studies are needed to better define the clinical repercussions of STHS.

## 4.14. Normalization of ST-segment Changes

Repolarization changes present at rest (T wave inversion and ST-segment depression) may, during exertion, exhibit progressive reduction and even normalization (“pseudonormalization of the ST-segment”) during anginal episodes and in chronic CAD.^549 , 550^This may be related to vectors directed oppositely to areas of ischemia canceling out. It is an uncommon finding and should only be considered significant when associated with pain or anginal equivalent.^549 , 551^

## 4.15. ST/HR Slope, ST/HR Index, ST/HR Loop, and ST/HR Hysteresis

The magnitude of ST-segment depression is associated with increases in exertion load, HR, and myocardial oxygen demand, especially in the presence of CAD. The correlation of ST-segment depression with HR behavior improves the accuracy of ET (PPV) for CAD diagnosis and risk stratification.^423 , 552^

### 4.15.1. ST/HR Slope

The ST/HR slope is calculated using linear regression, by correlating the magnitude of ST-segment depressions (most frequently within 60 ms of the J point) in each lead individually (including CM5; excluding aVR, aVL, and V1), with the respective HR at each stage of the ET.^553^Practical use of the ST/HR slope requires a ramp protocol with small increments, which is essential for adequate regression.^554^

An ST/HR slope >2.4 µV/bpm is considered abnormal; values >6 µV/bpm are suggestive of severe CAD (three-vessel CAD or LMCA involvement).^553^

Particular features of the ST/HR slope:

– Has shown 78% sensitivity, 93% specificity, and 89% accuracy in identifying three-vessel CAD or LMCA lesions.^555^

– At peak exertion, in patients with suspected CAD, an ST/HR slope ≥6.0 µV/bpm identified three-vessel CAD with 78% sensitivity, 97% specificity, a PPV of 93%, and 90% accuracy.^556^

### 4.15.2. ST/HR Index

The ST/HR index represents the ratio of ST-segment depression (ΔST) to change in HR (ΔHR) in the standing or sitting position. It does not require logistic regression or a smooth ramp protocol.^557^The ST/HR index is deemed abnormal if >1.6 µV/bpm.^553 , 557 , 558^


$$ST/HR\text{index}(\mu V/bpm)=\frac{\begin{array}{c} \Delta ST=\text{(greatest ST-segment depression on exertion}-\\ \text{resting ST-segment depression in millivolts}) \end{array}}{\Delta HR=(\text{maximum HR}-\text{resting HR)}}\times 100$$

ΔST: change in ST-segment depression from rest; ΔHR: change in HR

Note: 1 mm of voltage corresponds to 0.1 mV (millivolt) and 100 μV (microvolts)

Particular features of the ST/HR index:

– A cutoff value of 4.7 µV/bpm (±4.7) was associated with severe ischemia on myocardial perfusion scintigraphy (score ≥11; p<0.0001), with a sensitivity of 77% and specificity of 82%.^559^

– Its use has been shown to improve prediction of coronary events in asymptomatic high-risk men and in asymptomatic low-risk men and women.^557^

### 4.15.3. ST/HR Loop

The ST/HR loop is a continuous graph reflecting the behavior of ST-segment deflection (elevation or depression) in relation to the change in HR in lead V2 during exercise and at each minute of recovery. According to the direction of rotation of the ST/HR loop, patients can be divided into: clockwise rotation (with ST-segment deflection changing more quickly during recovery) or counterclockwise rotation (with more prolonged deflection during recovery).^560^

Particular features of the ST/HR loop:

– In post-MI patients, counterclockwise rotation of the ST/HR loop showed 88% sensitivity, 73% specificity, and 77% accuracy for myocardial ischemia in the peri-infarct area.^561^

– A sampling frequency <2 samples/min may impair performance in diagnosing CAD.^562^

### 4.15.4. ST/HR Hysteresis

ST/HR hysteresis measures the difference (in area) of ST depression between the exercise and recovery phases in relation to the corresponding HR. ST-segment amplitudes are measured in microvolts, 60 ms from the J point.^563 , 564^ST/HR hysteresis may provide greater diagnostic and prognostic accuracy compared to isolated analysis of ST deviation or the ST/HR index.^563 , 565 , 566^

Particular features of ST/HR hysteresis:

– Has shown 89% accuracy in detecting CAD.^563^

– A cutoff value of -15 µV provided the best diagnostic discrimination of CAD. In this study, an ST/HR slope of 2.4 µV/bpm and an ST/HR index of 1.6 µV/bpm were also helpful in the diagnosis of CAD.^567^

## 4.16. QT Interval, QTc, QT Hysteresis, and QT Dispersion

The QT interval (QTi) represents the ventricular electrical activity. It is directly related to HR (an increase in HR leads to shortening of the QTi) and is influenced by neurohumoral changes, including exertion.^568 , 569^

Due to the variation of QTi with HR, correction of QTi for HR (QTc) is recommended:


$$QTc=\frac{QTi}{\sqrt{RR}}$$

*QT measured in milliseconds and distance between RR in seconds.

On baseline ECG, QTc is usually corrected by Bazett’s formula for HR values between 60 and 90 bpm (Figure 14). When HR <60 bpm or >90 bpm, other formulas (such as the Fridericia, Framingham, and Hodges formulas) should be used instead. A technical limitation is the difficulty of accurately measuring the QTi in all phases of exercise, especially at higher HR values.^6 , 570 , 571^

On baseline ECG, the QTi is considered prolonged if >500 ms. QTc is normal up to ≤450 ms for men and ≤470 ms for women.^295 , 371 , 572^

Normally, the QTi begins to shorten at the onset of exertion. An increase in HR to 160 bpm shortens the QTi by 25% to 40%.^573^However, in some individuals (usually women), paradoxical QTi prolongation may occur in the first few minutes of exercise.

QTc increases at the onset of exertion, followed by a progressive decrease as HR becomes elevated. During recovery, as the HR decreases, the QTc returns to its baseline pattern.^574 , 575^

QTi dispersion (dQTi) is the difference between the longest and shortest QTi measured on all 12 ECG leads during a given ET phase (exercise or recovery).

QTc hysteresis (hQTc) – more appropriately, QT/RR hysteresis – is usually estimated using one of the following methods (Figure 14):^576 , 577^

– The hQTc variance (∆hQTc) is estimated by the difference (in ms) between the QTi measured in a predetermined RR (generally 600 ms) during exercise and recovery.^578^

– Area of the hQTc loops (AhQTc), estimated by quantifying the difference in area (in ms) of the QT/RR loop adjusted separately for HR increase and decrease during exercise and recovery.^579^

Healthy women exhibit greater QTc variation during exercise and recovery than men, resulting in a higher HQTc.^580^

### 4.16.1. Abnormal Responses

– Absence of QTc reduction at peak exertion has been associated with exercise-induced ischemia. However, it cannot be used as the sole criterion for this diagnosis.^581^

– Patients with stable CAD and a normal ET (without inducible ischemia) have a higher risk of arrhythmia when their QTc at peak exertion increased significantly compared to baseline (from 381 ms to 447 ms; p<0.001).^582^

– In patients with ischemic ST-segment changes, QTc (by Bazett’s formula; OR: 1.051) and dQTi (OR: 1.117) measured in the second minute of recovery were independent predictors of critical CAD. On recovery, a QTc ≥404 ms or dQTi ≥37 ms increased sensitivity to 90%.^583^

– Exercise-induced QTc prolongation (QTc >440 ms by Bazett’s formula) can discriminate post-MI patients at high risk of sudden cardiac death.^584^

– QTc interval hysteresis (AhQTc method) ≥375 during exercise and recovery was an independent predictor of myocardial ischemia (OR: 1.61; 95% CI: 1.22-2.12; p=0.0008).^585^

– A QTc interval hysteresis (AhQTc method) of 11 ms showed 77.9% sensitivity, 85.2% specificity, a PPV of 87%, NPV of 75.4%, and accuracy superior to that of the Duke score for CAD detection.^586^

– In a study of 273 patients with no past MI (age 56± 9 years, both sexes), dQTi immediately after exercise ≥60 ms (OR: 2.60; p<0.01) was a significant predictor of CAD, regardless of sex or the presence of ST-segment depression.^587^

– QTc measurements at peak exertion and recovery (3-4 minutes) contribute to the identification of LQTS1, and are recommended for survivors of sudden cardiac death.^70 , 153 , 588 , 589^

– Patients with LQTS1 showed a maximal increase in QTc at peak exercise (SD: ±21 to ±90 ms). QTc prolongation >30 ms in the third minute of recovery was associated with 75% positivity for LQTS1 on genetic testing.^140^

– Patients with LQTS1 showed progressive or persistent QTc prolongation with increasing HR on exertion. In LQTL2, there was maximal prolongation of the QTc at submaximal HR (50% of the predicted HRmax), with the QTc at peak exertion significantly lower than in LQTL1 (335±45 ms *vs* . 366±33 ms; p=0.01).^590^

– The isolated occurrence of QTc >480 ms in the 4th minute of recovery adds 1 point to the long QT syndrome risk score (Schwartz score).^591^A screening algorithm combining QTc at rest and at 4 minutes of recovery showed a sensitivity of 94% and specificity of 90% to detect LQTS.^592^

It is recommended that QTi and QTc values always be checked on the baseline ECG. If within normal limits, they do not need to be recorded in the ET report. If abnormal or if ET is being performed to investigate the behavior of the QT interval or in sudden cardiac death survivors, the report must include the baseline ECG QTc values, the formula used for correction, the longest QTc value observed on exertion, the QTc on peak exertion, and the behavior of the QTc during recovery (recording the QTc at the 4th minute of recovery is mandatory).

## 4.17. Disorders of Atrioventricular Conduction, Intraventricular Conduction, and Impulse Formation

### 4.17.1. Atrioventricular Conduction Disorders

**4.17.1.1. First-degree Atrioventricular (AV) Block**

Defined as PR interval (PRi) prolongation >200 ms (at HRs between 50 and 90 bpm) on baseline ECG in adults. Prevalence varies with age: in healthy young adults (20 to 30 years old), it is 0.65% to 2%; in the general population, ≈4%; in those over 60, between 3% and 5%. It is usually asymptomatic and, in medium and long-term follow-up, has been associated with a slightly increased risk of CAD, HF, and AF.^593 - 596^In high CV risk patients, it is strongly associated with ischemic stroke, MI, and CV death.^593 , 595 , 597^

In most cases, the PRi prolongation observed on the baseline ECG normalizes with exercise due to autonomic modulation. In healthy individuals, the PRi can reach up to 100 ms as HR increases.^295 , 598 , 599^

First-degree AV block (exercise-induced or persistent) is defined as a measured PRi greater than predicted for HR. PRi-for-HR prediction equations:


$$\text{- Average PRi for HR from}90\text{to}140bpm=(-0.287\times HR)+182.9^{600}$$

$$\text{- Average PRi for HR from}90\text{to}140bpm=(-0.287\times HR)+182.9^{600}$$

Patients with severe first-degree AV block (PRi ≥300 ms) on baseline ECG may develop a clinical picture similar to that of pacemaker syndrome during ET. Such patients are more likely to become symptomatic on light or moderate exertion due to failure of the PRi to adapt to exercise. PRi does not decrease appropriately as HR increases, causing excessive approximation of atrial systole to the preceding ventricular systole. Some of these symptomatic patients, particularly those with normal LV function, may benefit from pacemaker implantation with dual-chamber pacing (Class of Recommendation: IIa; Level of Evidence: B).^601 , 602^

First-degree AV block can occur at the end of exercise or in recovery, particularly in occult AV node disease. It may be associated with medication use (digitalis, beta-blockers, some calcium channel blockers, etc.) or conditions that prolong AV conduction time (myocarditis, Chagas disease, etc.).^439^

The Finnish Cardiovascular Study (FINCAVAS) of 1,979 patients undergoing ET (mean follow-up 47 months) demonstrated that first-degree AV block in the 2nd minute of recovery was associated with risk of CV mortality (continuous RR: 1.29, p=0.006; dichotomous RR: 2.41, p=0.045).^603^

**4.17.1.2. Type I Second-degree AV Block (Mobitz I)**

Characterized by gradual slowing of AV conduction (Wenckebach phenomenon) with progressive lengthening of the PRi until AV conduction is blocked. The block frequency can be variable, and repetition of the cycle may occur.^604^

The presence of Mobitz I AV block on the baseline ECG in asymptomatic healthy individuals does not contraindicate ET, and normalization of AV conduction usually occurs. In asymptomatic patients with heart disease, the benefit of ET should be carefully weighed.^605 - 607^

Persistence or onset of Mobitz I AV block during exertion are considered test cessation criteria when presenting with: symptoms of low cardiac output or angina; increased number of blocked beats; or HR reduction with progression of exercise.^6 , 608 - 610^

Mobitz I AV block with QRS complexes lasting ≥120 msec is associated with infranodal AV block in 30% to 40% of patients, having the same prognostic significance as seen in type II second-degree AV block, as both indicate severe disease of the His-Purkinje system.^131^

**4.17.1.3. Type II Second-degree AV Block (Mobitz II)**

In second-degree type II AV block (Mobitz II), sudden failure of AV conduction occurs, with the block located at or below the level of the His-Purkinje system.^611^

The presence of Mobitz II AV block on the baseline ECG is a contraindication for ET, because it is associated with severe disease in the cardiac conduction system and other heart diseases.^606^

Exercise-induced Mobitz II AV block is a test cessation criterion because it interferes with maintenance of cardiac output.^612 , 613^It is usually associated with CAD or aortic valve stenosis, and may progress to complete AV block.^614 , 615^

**4.17.1.4. Type 2:1 AV Block/Advanced or High-grade AV Block/Third-degree AV Block or Complete AV Block**

In 2:1 AV block, for every two beats of atrial origin, one is conducted and depolarizes the ventricle, while the other is blocked, with maintenance of constant PP intervals (considering that blocked premature atrial contractions have been ruled out). Exercise-induced 2:1 AV block is unusual and is associated with a drop in cardiac output, possibly leading to dyspnea and syncope. Most patients have a history of symptoms, and ET may be indicated for further investigation and to distinguish nodal from infranodal block. Type 2:1 AV block may be preceded by a run of Mobitz I or II block. Type 2:1 AV block triggered by increased HR (including exertion) is usually associated with disease of the His-Purkinje system.^612 , 616 - 618^

In advanced or high-grade AV block, there is AV conduction in fewer than half of the atrial beats, yielding a ratio of 3:1, 4:1, or higher. The presence of AV conduction is noted by the constant PR interval with each beat that generates a QRS complex. Most exercise-induced high-grade AV blocks are intra- or infra-Hisian.^619 , 620^

In third-degree AV block, also known as complete AV block, there is complete dissociation between atrial and ventricular electrical activity, resulting in blocked P waves that do not depolarize the ventricles. A subsidiary pacemaker below the level of the block takes over ventricular rhythm. The frequency of the atrial rhythm is usually higher than that of the escape rhythm. Complete heart block of supra-Hisian origin presents with QRS complex of the ventricular escape rhythm similar to those of the baseline ECG, while in those of infra-Hisian origin, the escape QRS complexes are wide.^621^

Acquired complete heart block is a contraindication to ET because increased sympathetic activity without a corresponding effective increase in HR can result in complex ventricular arrhythmias and serious complications.

Exercise-induced complete heart block is uncommon, and may be associated with transient ischemia or severe degenerative disease of the conduction system. If it occurs, test cessation is mandatory.^207 , 208 , 622^

Congenital AV block has a prevalence of 1 per 15,000 to 20,000 live births (60% are women).^623^ET can be used to help document symptoms, assess increased ventricular escape response, ascertain whether ectopy is present, and assess the hemodynamic repercussions of the block. Complete heart block located within the His-Purkinje system carries a worse prognosis. Occurrence of exercise-induced ventricular ectopy is associated with an increased risk of sudden death.^624^

ET can be performed in individuals with congenital AV block if there are no comorbidities (congenital or otherwise) that would jeopardize the safety of ET.

Particular aspects of ET in congenital complete heart block:

– Many patients may exhibit normal functional capacity. VO_2_max and HRmax prediction equations should not be used. Exercise-induced ectopy is frequent (50% to 70% of patients).^625 , 626^

– The natural history of congenital complete heart block consists of a progressive decline in ventricular rates throughout life. On resting ECG, between the ages of 6 and 10 years, the average HR is 50 bpm; between 16 and 20 years, 45 bpm; and over age 40 years, 38 bpm. Among adults with congenital complete heart block, 8% had sudden cardiac death as the first symptom. Fatigue, dyspnea, dizziness, and exercise-induced ventricular ectopy accounted for 26.5% of pacemaker placements.^627^

### 4.17.2. Intraventricular Conduction Disorders

Intraventricular conduction disorders (blocks) may precede the ET or may develop or disappear with exertion, with various clinical repercussions and associations with heart diseases.

ECG criteria for diagnosing intraventricular conduction disorders are found in the Brazilian Society of Cardiology Guidelines on the Analysis and Issuance of Electrocardiographic Reports – 2022.^295^

**4.17.2.1. Left Bundle Branch Block**

Left bundle branch block (LBBB) is rare in patients aged <50 years and almost never occurs in those aged <35 years, suggesting that it is an acquired disorder secondary to CV disease. Population studies have shown that the prevalence increases steadily after 50 years of age (<1%), reaching 6% at age 80.^295 , 628 , 629^

**4.17.2.1.1. Pre-existing Left Bundle Branch Block**

LBBB on baseline ECG poses a challenge for the analysis of ST-segment depression, which is generally not associated with myocardial ischemia in this setting, thus reducing the specificity and accuracy of ET.^628 , 630 - 632^In normal, healthy individuals with LBBB, ST-segment depression during exercise can reach up to 10 mm. ET can still be performed to investigate symptoms, as analysis of the other test variables is not impaired.^12 , 628^

In the presence of LBBB, use of the Sgarbossa diagnostic score is recommended in order to identify possible acute adverse events during ET:^633 , 634^

– ST-segment elevation ≥1 mm concordant with QRS (in lead with predominantly positive QRS): score = 5.

– ST depressions ≥1 mm in V1, V2 or V3: score = 3.

– ST elevation ≥5 mm discordant with QRS (in a lead with predominantly negative QRS): score = 2.

Interpretation of the Sgarbossa score in ET:

– ≥3 points = acute myocardial infarction (AMI) in presence of LBBB.

– <3 and >0 points = cannot rule out AMI. In high-risk patients or those with symptoms, perform additional evaluation.

– 0 points = expected behavior of LBBB during ET.

To diagnose AMI in the presence of LBBB as a complication during ET, one can also use the modified Sgarbossa score, Barcelona algorithm, or Smith criteria.^635 - 638^

Particular aspects of ET in patients with pre-existing LBBB:

– Causes major hemodynamic effects, including asynchronous myocardial activation, impaired systolic and diastolic function, and reduced ejection fraction.^639 - 641^

– In dilated cardiomyopathy with LBBB, the VO_2_peak is significantly lower than predicted VO_2_max (≈33% lower; p<0.001). LBBB and QRS duration are predictors of poor exercise tolerance.^642^

– Disappearance of the LBBB during exercise is exceedingly rare situation, usually associated with temporary (lasting for days or months) or transient (lasting only seconds or hours) bundle branch block. It is considered a fortuitous observation and its true relation to exercise is uncertain. Such blocks may be associated with underlying heart disease or conditions such as hypertensive disease, rheumatic fever, pulmonary embolism, hyperkalemia, and thyrotoxicosis.^643 , 644^

**4.17.2.1.2. Exercise-induced Left Bundle Branch Block**

Exercise-induced LBBB (EI-LBBB) occurs in approximately 0.4-0.5% of patients undergoing ET.^202 , 203 , 645^Its mechanism remains uncertain, and may be associated with valvular heart disease, cardiomyopathy, congenital heart disease, primary defects of the conduction system, CAD, or even in patients with no detectable disease. A longitudinal study showed that CAD and HF were the most prevalent causes.^203^

Transient EI-LBBB can cause reversible left ventricular dyssynchrony and secondary changes in filling pattern.^646 , 647^In addition, patients with EI-LBBB are at increased risk of developing permanent LBBB, ventricular dysfunction, and, rarely, complete heart block requiring pacemaker implantation.^203^

The main clinical forms of EI-LBBB are:

1) Asymptomatic, both at onset and at resolution.

2) Abrupt onset of chest pain (usually localized and nonradiating) of varying severity (ranging from discomfort to severe pain), simultaneous with onset, and concomitant improvement upon disappearance of LBBB, with a normal ECG before and after LBBB. A minority of patients may experience pain relief even before the LBBB disappears.

3) Typical chest pain before the onset of LBBB, which does not improve with resolution of LBBB, is usually associated with CAD (especially if ST-segment depression precedes LBBB).^643 , 648^

Painful LBBB syndrome consists of chest pain associated with EI-LBBB (as described in item 2, above) in a patient with normal ventricular function, with no other possible causes, and with an S/T wave amplitude ratio <1.8 (precordial and inferior wall leads). ET plays an essential role in its diagnosis.^648 - 652^

Particular aspects of EI-LBBB and associated abnormalities:

– In intermittent LBBB or after disappearance of EI-LBBB, deep, symmetric T wave inversions (in V1 to V4) commonly occur in normally conducted beats. They are a consequence of the LBBB itself, constituting an electrical phenomenon secondary to abnormal activation, and should not be interpreted as myocardial ischemia.^653 - 657^

– During the run of LBBB, the accuracy of ST-segment changes to detect myocardial ischemia is impaired.^631^

– Occurrence of ST-segment depression preceding the onset of LBBB is a valuable finding and its interpretation is not impaired; if it meets criteria for myocardial ischemia, it is usually associated with CAD.^648^

– Case reports and series have shown that the onset of LBBB at HR ≤125 bpm correlates strongly with the presence of obstructive CAD.^649 , 658^When EI-LBBB occurred at HR >125/min, normal coronary angiography and a better prognosis were generally observed.^202 , 659^

– A cohort of 25 patients with EI-LBBB (among 16,500 ETs) identified a PPV of 72% for CAD and, when occurring at HR <120 bpm, it was associated with proximal LAD stenosis.^660^

– Painful LBBB syndrome and EI-LBBB at HR >125 bpm are generally not associated with obstructive CAD.^648 - 652^

– In a study of 9,318 patients (mean follow-up 6.9 years), only 20 patients had EI-LBBB. Those with CAD (60%) had a worse prognosis (increased risk of death and MI). The risk of developing permanent LBBB and complete heart block was similar in patients with and without CAD.^648^

**4.17.2.2. Fascicular Blocks**

Fascicular blocks, or hemiblocks, on baseline ECG do not preclude the analysis of exercise-induced changes in ventricular repolarization in terms of ischemia; however, they can decrease the accuracy of ET.^661 , 662^

Exercise-induced left fascicular blocks are rare. The most accepted mechanism for its occurrence is ischemia-induced slow conduction in fibers of the left bundle and its hemifascicles, or in the myocardial fibers of Purkinje.^663^

Particular aspects of exercise-induced left fascicular blocks:

– They correlate strongly not only with the presence of CAD, but with extension and severity.^661 , 664 - 667^

– Exercise-induced left posterior fascicular block (LPFB) has been associated with RCA or multivessel CAD.^668^

– Exercise-induced left anterior (LAFB) and left septal (LSFB) fascicular blocks are associated with LMCA or LAD disease.^669^

– Several case reports have documented the reversibility of exercise-induced LAFB after interventional treatment of coronary obstruction.^664 , 665 , 670^

**4.17.2.3. Right Bundle Branch Block**

**4.17.2.3.1. Pre-existing Right Bundle Branch Block**

Right bundle branch block (RBBB) occurs in 0.2% to 3% of the general population. Prevalence increases with age, and is higher in men (≈14.3% in men >80 years).^671^

Isolated RBBB is generally benign, except in certain heart diseases (i.e. cardiomyopathy, CAD, or HF), in which it is associated with increased CV mortality. RBBB is common in apparently healthy individuals.^672^The main differential diagnosis of the ECG with RBBB is Brugada syndrome.^673 - 675^

RBBB on the baseline ECG invalidates the interpretation of ST changes on exertion, but only in leads V1 to V3. It is usually present at rest and increases with exertion, with no association with CAD. Quantification and interpretation of ST-segment depression in the other leads allows diagnosis of exercise-induced ischemia.^645 , 676 , 677^

Particular aspects of pre-existing RBBB:

– A study of 3,609 patients undergoing ET identified RBBB in 163 (4.5%), of whom 133 were followed up (36% went on to develop MI). ET had a sensitivity of 27%, specificity of 87%, and accuracy of 62% for CAD. During follow-up, an annual mortality rate of 10% was observed.^678^

– In a study of 23,026 patients without a diagnosis of CVD, 220 (0.96%) with RBBB had a higher all-cause mortality rate (RR: 1.5; 95% CI: 1.1-2.0; p=0.0058) and CV mortality rate (RR: 1.7; 95% CI: 1.1-2.8; p=0.0178). Patients with RBBB had lower exercise tolerance, slow HR recovery, and a higher prevalence of dyspnea.^673^

– In a cohort of 7,073 adults undergoing ET plus myocardial perfusion scintigraphy (mean follow-up 6.7 years), mortality was higher among those 190 patients with RBBB than in those with no such block (24% *vs.* 11%, respectively; RR: 1.5; 95% CI: 1.1-2.1; p=0.007), even after adjustment for exercise capacity, nuclear perfusion defects, and other risk factors. Incomplete RBBB has not been associated with mortality.^679^

– There are few case reports in the literature of pre-existing RBBB disappearing during exercise; the mechanism involved is unclear.^680 - 682^

**4.17.2.3.2. Exercise-induced Right Bundle Branch Block**

Exercise-induced RBBB (EI-RBBB) occurs in approximately 0.25% of patients undergoing ET, thus being less common than EI-LBBB. It is usually associated with CAD.^152^

Particular aspects of EI-RBBB:

– Analysis of ischemic changes during a run of EI-RBBB is similar to that described above for patients with pre-existing RBBB.

– Two cohorts evaluating patients with EI-RBBB identified a high prevalence of CAD in these patients, whether single or multivessel.^645 , 660^

– In a cohort of 8,047 patients (mean follow-up 8.8 years), those 23 patients with EI-RBBB had a higher prevalence of CAD and HF and a higher risk of death.^204^

– In a cohort of 3,974 men (mean age 57.5 years; mean follow-up 5.9 years), 1.9% had EI-RBBB, which was associated with an increased risk of all-cause mortality (p<0.001).^306^

### 4.17.3. Disorders of Impulse Formation

Development of abnormal heart rhythms during ET is common in patients with and without CVD. Arrhythmias are often isolated, transient, episodic, and asymptomatic, and generally pose no risk of CV events. They usually exhibit great spontaneous and circadian variability, which hinders reproducibility.^299^

When abnormal heart rhythms occur, continuous ECG recording is suggested for diagnosis, quantification, and documentation of arrhythmias.

At rest, the main mechanisms involved in arrhythmogenesis are reentry, enhanced automaticity/triggered activity, and late/delayed afterdepolarizations. Other factors include electrolyte and pH abnormalities, hypoxia, hemodynamic factors (pre and afterload, LV wall distention, etc.), autonomic modulation, circulating catecholamines, drug interactions, and myocardial ischemia. Exercise can be a trigger due to the simultaneous withdrawal of vagal activity, increased sympathetic activity, changes in cardiac automaticity, and increased myocardial oxygen consumption. During recovery, there is a sudden resumption of vagal tone and other hemodynamic changes that can also precipitate arrhythmias (Figure 15).^63 , 133 , 134 , 142^

During the pretest interview, clinicians are advised to investigate factors that might precipitate or aggravate arrhythmias, such as physical exertion, excess intake of caffeine and alcohol, smoking, recreational drug use, and hyperthyroidism. If abnormal heart rhythms occur during ET, correlation with pretest data and possible exercise-related triggers (i.e. ischemia) is advised.^63 , 133 , 134 , 683^

**4.17.3.1. Ventricular Arrhythmias**

ET is useful for investigating symptoms suggestive of arrhythmia, establishing the diagnosis, and assessing arrhythmia behavior (potentiation and suppression) and prognosis in selected patients.^684^

Premature ventricular contractions (PVCs) present as ectopic ventricular beats, premature in relation to the previous RR interval, usually with a post-extrasystolic pause. If there is no change in duration of the RR interval, the PVC is known as an *interpolated* PVC.^63 , 188 , 371^PVCs are a common finding in clinical practice, increasing in frequency with age and CV disease. The prevalence in the apparently healthy population ranges from 1% to 4% at rest and 5% to 34% during ET. In patients with cardiomyopathy, the incidence of PVCs during ET can reach ≈90%.^684 , 685^

PVCs can be classified according to:

– Morphology: monomorphic or polymorphic (more than one morphology).

– Relationship with the sinus rhythm and with other PVCs:

• Isolated: a single ventricular ectopic beat.

• Paired (pairs, doublet or couplet): two PVCs, with the same or different morphology, with a fixed or variable coupling interval.

• Triplet: three ventricular ectopic beats in a row. Considered equivalent to NSVT.

• Bigeminy: PVCs alternate with normal sinus rhythm at a 1:1 ratio, i.e. every other beat is a PVC. Occurs repetitively for short or prolonged runs.

• Trigeminy: PVCs alternate with normal sinus rhythm at a 1:2 ratio, i.e. every third beat is a PVC.

• Quadrigeminy: PVCs alternate with normal sinus rhythm at a 1:3 ratio, i.e. every fourth beat is a PVC.

– Frequency: The Lown-Wolf grading sstem allows quantification of PVCs, and is thus useful for defining severity. Classes I and II require Holter nonitoring:

0: Absence of PVCs.

I: <30 PVCs/hour.

II: ≥ 30 PVCs/hour.

III: Polymorphic PVCs.

IVa: Ventricular doublets.

IVb: Ventricular tachycardia (3 or more consecutive PVCs).

V: short coupling interval (R-on-T phenomenon).

In the general population, PVCs are defined as frequent when at least one PVC is observed on a baseline 12-lead ECG or ≥30 PVCs/h (Lown-Wolf class II) are observed on Holter monitoring. Frequent PVCs are associated with increased CV risk and mortality.^63 , 191^

Classification according to morphology, QRS duration, and relationship with the sinus rhythm and with other PVCs can be done both on baseline ECG and during the ET.

Ventricular tachycardia (VT) corresponds to at least three successive ventricular beats with HR >100 bpm. It is classified according to:

– Morphology: Monomorphic VT (uniform morphology) or polymorphic VT (three or more morphologies).

– Duration: nonsustained (NSVT) if <30 seconds or sustained if ≥30 seconds.

– Symptoms and hemodynamic repercussions: absent or present.

If wide-QRS tachycardia is present (QRS >120 ms), algorithms (Brugada or Vereckei) may be necessary for the differential diagnosis of supraventricular tachycardia versus aberrant ventricular conduction.^686^

*Torsades de pointes* -type VT presents with wide, polymorphic QRS complexes, “twisting” around the baseline, preceded by long/short cycles, with a long QTc interval (congenital or secondary to drugs and electrolyte disturbances). It is usually self-limiting.

Bidirectional VT is VT with a RBBB (or, rarely, LBBB) morphology associated with alternating blockade of the anterior and posterior divisions (hemifascicles) of the left bundle branch. The “bidirectional” appearance is so named because a ventricular beat with a positive QRS complex is followed by another with a negative QRS morphology, and so on successively. It is usually associated with severe advanced cardiomyopathy, digitalis toxicity, and familial catecholaminergic polymorphic ventricular tachycardia.

Exercise-induced ventricular arrhythmias (EIVA) are defined as any PVCs or ventricular tachycardia emerging during exercise and recovery. When frequent, it is a marker of poor prognosis.^687^

Main definitions of frequent EIVA:

1) Occurrence of ≥7 PVCs/minute, ventricular bigeminy or trigeminy, doublets or triplets, ventricular tachycardia, ventricular flutter, *torsades de pointes* , or ventricular fibrillation.^192^

2) When, during any 30-second period of the ET, ventricular arrhythmias account for more than 10% of beats, or VT (defined as ≥3 consecutive PVCs) occurs.^688 , 689^

When EIVA occurs, the ET report should include its manner of presentation and whether it was frequent (according to the above definitions).

Symptoms associated with EIVA generally consist of a “missed beat” or “rapid heartbeat” sensation (palpitations), chest discomfort, fatigue, and dizziness. Existing ejection murmurs may be exacerbated due to increased stroke volume and contractile force after compensatory pauses. In VTs, symptoms tend to be more frequent and intense. With sustained VT, hemodynamic repercussions are common, often associated with complaints of chest discomfort or pressure, typical chest pain, dyspnea, palpitations, diaphoresis, dizziness, nausea, presyncope, and syncope.^690^

Particular aspects of ventricular arrhythmias during ET:

– Frequent PVCs at any phase of the ET is associated with a higher risk of death from all causes and CV death, especially when PVCs occur during recovery, and is even higher if they occur during both exercise and recovery.^191 - 194 , 196 , 689 , 691^

– Markers of poor prognosis: polymorphic PVCs; PVCs with LV outflow tract morphology; PVCs of increasing density on exertion; PVCs with short coupling interval.^684 , 692^

– In a study with 302 patients (mean age 54 years, 152 men), 22% had frequent EIVAs associated with a higher occurrence of perfusion abnormalities and ST-segment depression, especially among men (67% *vs.* 38%; p<0.05).^693^

– In the apparently healthy population (including athletes), isolated PVCs at rest tend to decrease with exercise, and generally carry a good prognosis.^694 - 696^

– In athletes with EIVT (≥3 consecutive beats at ≥120 bpm, sustained or nonsustained), further investigation is recommended for diagnostic elucidation and risk stratification.^136 , 694 , 695^

– PVCs and EIVAs in familial arrhythmic conditions (i.e. long QT syndrome) and catecholamine-sensitive conditions (i.e. right ventricular outflow tract VT).^63 , 130 , 188^

**4.17.3.2. Supraventricular Arrhythmias**

Supraventricular arrhythmias are relatively common, often repetitive, occasionally persistent, and rarely fatal. The main precipitating factors are age (more common in older adults), sex (more common in females), and comorbidities (i.e. hypertension, valvular heart disease, and cardiomyopathies).^697 - 700^

An isolated supraventricular extrasystole (SVES) is a premature atrial contraction (PAC) or premature junctional contraction (PJC) that is followed by ventricular depolarization, with morphology and duration similar to those of the preceding sinus beats. Atrial tachycardia (AT) is an atrial rhythm originating in a region other than the sinus node, characterized by the presence of a P wave distinct from the normal sinus P wave, with an atrial rate >100 bpm. Variable AV conduction is common. Multifocal atrial tachycardia (MFAT) has the same characteristics as multifocal atrial rhythm, with an atrial rate >100 bpm.

Typical atrioventricular nodal reentry tachycardia (AVNRT) uses the AV node as a fundamental part of its circuit through the mechanism of nodal reentry. The atrial activation wave is generally within the QRS complex and is not observable. In cases of AVNRT with wide QRS, tachycardias of ventricular origin must be included in the differential diagnosis. In atypical AVNRT, the direction of activation is reversed (retrograde atrial conduction), with an RP interval longer than the PR interval.

Orthodromic atrioventricular reentry tachycardia (OAVRT) uses the normal conduction system in the antegrade direction and an accessory pathway in the retrograde direction. The QRS complex is narrow and the P wave is retrograde, with different morphologies possible (depending on the location of the accessory pathway) and an RP interval >80 ms.

Supraventricular tachycardias can be classified on the basis of the RP interval:

– Short RP’ (usually up to 120-140 ms): associated with typical AVNRT and anomalous bundle branch reentry tachycardia.

– Long RP’: associated with atypical AVNRT, OAVRT, and Coumel’s (or permanent junctional reentrant) tachycardia (in which reentry is caused by retrograde conduction through an accessory pathway).

Supraventricular arrhythmias may present with a wide QRS complex in the following situations:

– Aberrant conduction, in which a supraventricular stimulus (normal or extrasystolic) meets resistance to regional propagation in the conduction system, generating a QRS with bundle branch block morphology.

– Supraventricular tachycardia with aberrant conduction is a generic umbrella term for short- or long-RP’ supraventricular tachycardias.

Supraventricular arrhythmias (SVES, atrial fibrillation, atrial flutter, PSVT) are commonly exercise-induced. They occur in up to 10% of ETs of apparently healthy patients and in up to 25% of ETs of those with known or suspected CAD.^189 , 197^

Isolated PACs are a common finding during ET, with an incidence of 4% to 18%. They are usually asymptomatic, even when exercise-induced. PACs do not correlate with myocardial ischemia or risk of CV/MI mortality.^189 , 197 , 198^

Isolated PACs, sinus arrhythmias with runs of sinus bradycardia, and wandering atrial pacemaker rhythm are relatively common at the onset of exertion and recovery, both in apparently healthy patients and in those with heart disease. PACs on baseline ECG tend to become progressively less frequent with exertion, and are generally benign.^6 , 142 , 699^However, older adults with frequent PACs during exercise (>5/stage) are at increased risk of AF/flutter (RR: 15.23; 95% CI: 4.59-50.56; p<0.001).^199^

OAVRT is more common in middle-aged and elderly patients, while in adolescents, a similar prevalence of OAVRT and AVNRT is observed. Both are rare during ET, begin and end suddenly, cause HR of 150 to 250 bpm, and usually require adenosine for termination.^129 , 190^

The incidence of manifest pre-excitation or WPW pattern in the general population is 0.1% to 0.3%, and exercise-induced PSVT (EI-PSVT) is exceedingly rare.^129 , 701 , 702^

The incidence of EI-PSVT ranges from 3.4% to 15% in patients with paroxysmal supraventricular arrhythmias.^185 , 197^It is more common in elderly men, is generally asymptomatic, nonsustained, occurs near peak exertion, and is not associated with exercise-induced ischemia or CV mortality.^189^However, it is usually associated with palpitations, chest discomfort, malaise, dizziness, presyncope, and dyspnea. More rarely, sudden exercise intolerance, hypotension, frank syncope, signs of HF, and even shock may occur.^129 , 703 - 705^

Particular aspects of supraventricular arrhythmias during ET:

– Individuals with EI-PSVT (i.e. a run of 3 or more consecutive heartbeats) had a higher risk of AF at a mean follow-up of 5.7 years (RR: 7.6; p<0.001).^189^

– During episodes of PSVT, ST-segment depression may occur and is generally not associated with myocardial ischemia.^706 , 707^

– Exercise-induced supraventricular arrhythmias are most commonly associated with advanced age, COPD, recent alcohol intake, or excess caffeine intake.^6^

**4.17.3.3. Atrial Fibrillation/Atrial Flutter**

Atrial fibrillation (AF) is characterized by disorganized atrial electrical activity (“f” waves) with an atrial rate between 450 and 700 bpm and a variable ventricular response.^371^At rest, consider rhythm and rate as follows:

– Slow ventricular response when HR ≤50 bpm.

– Strict (or adequate) rate control when HR is 51 to 89 bpm.

– Lenient (or inadequate) rate control when HR is 90 to 110 bpm.

– Rapid ventricular response when HR >110 bpm.^295^

AF classification criteria:^119^

– Paroxysmal: a single episode of AF that spontaneously converts to normal sinus rhythm within 7 days.

– Persistent: patient requires electrical or chemical cardioversion to restore sinus rhythm.

– Permanent or chronic: present for >6 months or when the patient and physician decide to no longer attempt to restore sinus rhythm.

Atrial flutter is characterized by organized atrial electrical activity (“F” waves), and is divided into:^133 , 371^

– Type I (common or typical), with counterclockwise activation, an atrial rate between 240 and 340 bpm, and “F” waves with a sawtooth appearance (negative in the lower leads and positive in V1).

– Type II (atypical or unusual), with clockwise activation, an atrial rate between 340 and 430 bpm, varying degrees of AV block, and wide positive “F” waves in the inferior leads.

In AF and flutter, HR should be determined from a 6-second ECG tracing. Generally, exertion does not cause an increase in the frequency of atrial waves, and the increase in ventricular rate depends on AV conduction. In AF (persistent and chronic) and chronic atrial flutter, ET is useful in assessing symptoms and HR response, optimizing therapy, and informing exercise prescription/rehabilitation.^708 - 712^

Preferably, patients should continue to take any medications to control rhythm and/or ventricular rate and anticoagulants; these should not be withheld for ET.^133^

In patients with untreated permanent AF, the HR during an ET usually ranges from 90 to 170 bpm. AF with HR <60 bpm on resting ECG may be associated with AV node disease, sinus node dysfunction, or medications that affect HR (beta-blockers and antiarrhythmics).^133^

Patients with AF can safely be exercised to symptom-limited maximal exertion in the absence of other formal indications for test cessation. Drug control of HR is considered adequate when the chronotropic response is similar to that of patients in normal sinus rhythm.^708 - 711^Marked increases in HR, reaching or exceeding submaximal HR as early as the first stage of exercise, as well as a HRpeak >110% of the predicted maximum HR (for age), require optimization of pharmacotherapy, as they are predictors of HF and reduce physical ability.^159 , 713 - 716^In patients with HF and permanent AF, ΔHR has been shown to be associated with exercise performance, morbidity, and mortality.^158 , 717 , 718^

In the presence of AF, assessment of ischemia by ET is impaired, as any changes in the ST-segment may be due to the arrhythmia itself (low PPV). However, absence of exercise-induced ST-segment depression confers a high NPV for ischemia.^713 , 719^

Permanent AF, regardless of the underlying disease (HF, hypertension, ischemic heart disease, cardiomyopathy, or valvular heart disease), is associated with a lower VO_2_max than predicted for age.^715 , 717^

Conversion to sinus rhythm has been shown to reduce HRpeak (≈40 bpm) and improve cardiorespiratory fitness (≈15%).^720^

In chronic atrial flutter, there is usually a change in the AV conduction pattern – with a reduction in the degree of blockade and an increase in HR – during exercise. Flutter-associated symptoms (fatigue, dyspnea, malaise) are all related to elevated HR. Although atrial flutter with 1:1 AV conduction is rare, recognizing it is essential, as it can precipitate rapid hemodynamic instability and syncope. At the onset of recovery, a transient type I second-degree AV block is usually seen, followed by a return to baseline ECG pattern. Ischemia cannot be assessed if atrial flutter is present.^721 - 723^

Exercise-induced AF and flutter are rare, each occurring in <1% of the population. During ET, there may be a switch from exercise-induced AF to exercise-induced atrial flutter or vice versa, as one arrhythmia can trigger the other.^724^Both can occur in apparently healthy individuals, in rheumatic heart disease, hyperthyroidism, Wolff-Parkinson-White syndrome, and cardiomyopathy. Both tend to cause hemodynamic repercussions when there is a rapid ventricular response to exercise. In exercise-induced AF, assessment of ischemia is hampered by the low PPV, and in exercise-induced flutter, it is altogether impossible.^201 , 725^

**4.17.3.4. Bradyarrhythmias/Chronic Chronotropic Incompetence**

A heterogeneous group of individuals have inappropriately low resting HR, and most are asymptomatic and unaware of this abnormality. Resting bradycardia is common in high-performance athletes, in whom it is associated with increased vagal tone. It may also be secondary to medications (antiarrhythmics and beta-blockers), intrinsic sinus node disease (generally due to ischemia), degenerative changes, atrial cardiomyopathy, and sinus node dysfunction.^131 , 726 , 727^

In this setting, the ET allows analysis of the chronotropic response to sympathetic stimuli, correlating any potential symptoms with bradycardia, which may play a decisive role in diagnosis and therapy (including the decision to implant a permanent pacemaker).^131 , 368^

In patients with nonpathologic sinus bradycardia undergoing ET, a normal chronotropic response is observed, which characterizes these individuals as “vagotonic”. However, in patients with sinus bradycardia and a depressed chronotropic response, sinus node dysfunction (SND) is usually seen. Some patients with SND may achieve an appropriate HRpeak during exertion, but may exhibit very slow HR acceleration in the early stages of the protocol or rapid HR deceleration in the early recovery stage. SND can trigger symptoms of CHF and angina on exertion.^133^

In patients with nonpathologic sinus bradycardia undergoing ET, a normal chronotropic response is observed, which characterizes these individuals as “vagotonic”.

Patients with sinus node dysfunction (SND) undergoing ET may present with: sinus bradycardia at rest, with a depressed chronotropic response to exertion; rarely, very slow HR acceleration in the initial stages of the protocol, potentially even reaching the predicted HRmax; and very rarely, isolated rapid HR deceleration at the beginning of recovery despite absence of good physical conditioning. SND can trigger symptoms of CHF and angina on exertion.^133^

Some patients with exercise-induced sinus bradycardia may also experience exercise-induced syncope, with a profound drop in BP due to the Bezold-Jarisch reflex.^205 , 206^

**4.17.3.5. Inappropriate Sinus Tachycardia**

Inappropriate sinus tachycardia syndrome (IST) is a chronic disorder characterized by:^133 , 728^

– Increased HR (sinus rhythm) disproportionate to physiologic demand: resting daytime HR >100 bpm; mean HR on 24-hour Holter >90 bpm; exaggerated HR response to minimal physical exertion or emotional stress.

– Absence of other causes that would explain sinus tachycardia.

– On resting ECG, during sinus tachycardia, the P wave has an axis and morphology similar to those of regular sinus rhythm.

– Symptoms associated with tachycardia, such as palpitations, fatigue, dyspnea, exercise intolerance, and anxiety.

Most patients with IST are women aged ≈38±12 years. In a middle-aged population, the prevalence of IST (symptomatic or asymptomatic) is up to 1.2%. However, in post-COVID and chronic (long) COVID, this prevalence can reach up to 20% (age 40.1±10 years, 85% women, 83% mild COVID-19), despite absence of structural heart disease, a pro-inflammatory state, myocyte injury, or hypoxia.^729 , 730^

The natural course and prognosis of IST are generally benign, and tachycardia-induced cardiomyopathy rarely occurs. An association with comorbid psychiatric conditions is not uncommon. The main differential diagnosis is with postural orthostatic tachycardia syndrome (POTS), a disorder of the autonomic nervous system in which an increase in HR >30 bpm or HR >120 bpm is observed in the first 10 minutes when standing (hence, orthostatic).^731 , 732^

ET is useful in the evaluation of IST, as it usually shows an early, excessive increase in HR in response to minimal exertion (HR >130 bpm within the first 90 seconds of exercise with the Bruce protocol) and/or rapid achievement of HRmax. This HR response is different from that found in sedentary individuals, as there are associated symptoms.^133 , 733 , 734^

## 4.18. Indirect Metabolic Assessment

### 4.18.1. VO 2 /METs

Estimation of aerobic capacity should preferably be described using the amount of effort performed in METs or the respective estimated VO_2_max. The actual value achieved must be the one reported, as well as the percentage of the predicted value. It should not be expressed in number of minutes of exercise or stage of exercise achieved, as these terms hinder clinical interpretation and may vary greatly between protocols.^735 , 736^

Calculation of VO_2_and its conversion to METs, predicted values for age and sex, and their prognostic significance were described elsewhere in this Guideline.

ET is indicated to determine exercise tolerance in apparently healthy individuals with CVD (ischemic heart disease, HF, cardiomyopathies, valvular heart diseases, arrhythmias, congenital heart diseases, PAD, etc.) and comorbidities (i.e. diabetes and COPD).

In adult patients with CVD, failure to achieve the following is considered indicative of exercise intolerance/poor physical performance:

– Work load of 5 METs (estimated aerobic capacity).^737^

– Aerobic capacity of 15.0 mmL/kg/min (measured directly by CPET).

Particular features of VO_2_/METs:

In adults, achieving <5 METs (in women) or <7 METs (in men) during an exercise test was considered a high-risk marker; this is no longer true. Each individual should be evaluated individually, in relation to age, physical conditioning, and comorbidities.^735 , 738^

– Several studies have shown that patients who achieve ≥10 METs on ET, especially older adults, have low rates of adverse cardiac events and mortality, regardless of exercise-induced ischemia.^257 , 739 - 742^

– For every 1 MET increase in work load, there was an 18% reduction in cardiac events in men aged ≥65 years and a 14% reduction in those aged <65 years.^742^

### 4.18.2. Functional Aerobic Impairment (FAI)

Functional aerobic impairment (FAI) is the percent difference between the VO_2_maximum actually achieved (estimated or measured) and the predicted VO_2_(for age, sex, and activity level), calculated using regression equations.

The FAI is a measure that expresses percent deficit, i.e. the percentage of impaired functional aerobic capacity:^743^


$$FAI(\%)=\frac{{VO}_{2}\text{max predicted}-{VO}_{2}\text{peak (estimated or measured)}}{{VO}_{2}\text{max predicted}}\times 100$$

Legend (age in years):

– VO_2_peak: estimated by ET or measured by CPET.

– Calculation of VO2max predicted (mL/kg/min):

1) For sedentary subjects

• Women: 42.3 – (0.356 × age)

• Men: 57.8 – (0.445 × age)

2) For active subjects

• Women: 42.9 – (0.312 × age)

• Men: 69.7 – (0.612 × age)

FAI values can be interpreted as follows (Table 34):

– >26%: the higher the FAI value, the greater the impairment of the patient’s functional capacity.

– 0% to 26%: there is no significant impairment considering the patient’s age and sex.

– Negative values: the patient exceeded the predicted VO_2_. This is commonly observed in apparently healthy and active subjects, especially athletes.

Its main indications are:

– In serial ET, to quantify the progression of aerobic capacity (worsening or improving).

– In athletes, to quantify the progression of improvement in functional capacity.

– To estimate cardiorespiratory fitness for age. FAI with a positive value denotes that the patient has a capacity below that expected for age, and vice versa.^745^

– Objective evolution of cardiorespiratory fitness for physical training and rehabilitation programs.^29 , 746 , 747^

### 4.18.3. Myocardial Aerobic Impairment (MAI)

Myocardial aerobic impairment (MAI), also known as left ventricular impairment (LVI), is a measure that expresses the percentage of compromised left ventricular myocardial capacity to respond to the demands of physical activity, based on HR and SBP responses to exercise. The MAI calculation formula is:^748^


$$\text{MAI}(\%)=\frac{\text{Maximum DP predicted}-\text{maximum DP achieved}}{\text{Maximum DP predicted}}\times 100$$

Legend:

– Maximum DP (double product) achieved = HRpeak × SBPmax.

– Maximum predicted DP in sedentary patients:

• Women: [354 – (0.48 × age)] × 100

• Men: [438 – (1.59 × age)] × 100

– Predicted DP for active men:

Maximum predicted DP = [364 – (0.58 × age)] × 100

Note: age in years. MAI interpretation is impaired when there is a drop in intra-exercise HR and/or hypotension/drop in intra-exercise SBP (see Tables 31 and 32).

Particular features of the MAI:

– Used in serial ET for longitudinal follow-up after interventions in patients with CAD and valvular heart disease.^749 , 750^

– A cohort of 104 patients undergoing PCI and 38 status post successful CABG who underwent ET (pre- and post-intervention; 2-year follow-up) found significant improvement in the MAI in both groups (PCI: from 20.2±17.8% to 9.9±15.8%; CABR: from 31.9±21.7% to 9.9±19.3%).^751^

4.19. Post-test Risk Scores and ET Prognostic Variables

Application of post-test scores reduces interpretation biases, improves diagnostic accuracy, allows estimation of prognosis, provides cost-effective strategies for managing CVD, and assists non-specialist physicians in the interpretation of ET results.^752 - 755^

Scores are developed, validated, and applied in specific populations. The choice of score must be based on the characteristics of each patient, and the clinician is advised to record the rationale for score selection in the ET report.

### 4.19.1. Duke Score

The Duke score is among those most widely used for the diagnostic evaluation of severe CAD and the prediction of morbidity and mortality.^756 , 757^It is indicated for symptomatic patients of both sexes with suspected CAD, aged between 45 and 75 years.^758 , 759^Application of the Duke score has significant limitations in asymptomatic, low-risk patients; after CABG; and after recent MI.^760 , 761^

Calculation of the Duke score requires only 3 ET variables: magnitude of the ST-segment deviation, exercise tolerance time, and whether exertional angina occurred. The equation is:^762^


Duke score=exercise time−(5×ST deviation)−(4×angina)

Legend:

1) Exercise time: duration of exercise in minutes (Bruce protocol). If another protocol is used, convert the achieved METs to the corresponding Bruce protocol value to determine the time in minutes.

2) ST deviation: depression or elevation of the ST-segment, measured in millimeters.

3) Angina: zero, if no angina; 1 point, if angina occurred during exertion; 2 points if the angina was test-limiting.

The final Duke score ranges from ≥ +15 points to ≤ -25 points, allowing risk classification:

– High: score ≤ -11 points, indicates a 5-year survival of ≈67% and annual mortality ≥5%.

– Intermediate: ranges from -10 to +4 points, indicates a 5-year survival of ≈90%.

– Low: ≥ +5 points, indicates a 5-year survival of ≈97% and annual mortality ≤1%.^756 , 757^

Particular features of the Duke score:

– In the original score development study, carried out in men, 74% of those classified as high-risk had three-vessel occlusive CAD or an LMCA lesion on coronary angiography.^762^

– A study of 976 women and 2,249 men who underwent ET and coronary angiography found that women and men differed in terms of Duke score (1.6 *vs* . -0.3; p<0.0001), prevalence of CAD (32% *vs.* 72%; p<0.001), and 2-year mortality (1.9% *vs.* 4.9%; p<0.0001). The Duke score performed better to rule out CAD in women, especially in those classified as low risk.^763^

– In a prospective study with 603 patients followed for 2 years, patients with normal MPI and a low-to-intermediate-risk Duke score had fewer nonfatal MIs, whereas high-risk patients had a higher risk of MI and CV death.^764^

– 6,251 patients from the GISSI-2 study underwent ET 1 month after MI and were stratified by the Duke score. At 6-month follow-up, mortality rates by Duke risk groups were: low risk = 0.6%; intermediate risk = 1.8% (RR: 2.50; 95% CI:1.47-12.59; p=0.0001); and high risk = 3.4% (RR: 5.13; 95% CI: 3.61-15.55; p=0.0001).^765^

### 4.19.2. Athens Score/QRS Score

The Athens QRS score is a post-test score for the diagnostic evaluation of multivessel CAD that can be used in both sexes. It should not be used in patients with bundle branch blocks (right or left), left ventricular hypertrophy, ventricular pre-excitation, or ventricular bigeminy.^766 - 768^

Exercise-induced changes in the amplitude of Q, R, and S waves are useful for the diagnosis of CAD.^769^In 1990, these were compiled into a score dubbed the “Athens QRS score”, now also simply called the QRS score.^770^

It is calculated by averaging the amplitude of the Q, R, and S waves in three consecutive QRS complexes in leads aVF and V5, at rest and immediately after exercise. QS complexes should be treated as either a Q wave or an S wave. The QRS score is calculated by the formula:^767 , 769 , 770^


QRS score (in millimeters)=(ΔR−ΔQ−ΔS)aVF+(ΔR−ΔQ−ΔS)V5

ΔR = mean R at rest – mean R at peak exertion.

ΔQ = mean Q at rest – mean Q at peak exertion.

ΔS = mean S at rest – mean S at peak exertion.

In patients without CAD, a QRS score >5 mm is considered normal.

In ET, the QRS score is considered abnormal in the following situations:

– A QRS score ≤ +5 mm predicts the presence of obstructive CAD, regardless of ST-segment changes, with sensitivity ranging from 75% to 86% and specificity from 73% to 79%. The QRS score is associated with CAD severity; the lower the score, the greater the probability of significant multivessel coronary stenosis.^770 , 771^

– In women, a QRS score <5 mm in the presence of ischemic ECG response to exercise increased sensitivity from 59% to 80%, specificity from 40% to 94%, accuracy from 50% to 87%, and reduced false positives from 60% to 6%.^772^

– A QRS score < +5 mm has greater diagnostic capacity for restenosis when compared to ST-segment depression in patients 6 months after single-vessel PCI (sensitivity = 80%, specificity = 89%, PPV = 77%) and in ischemia 1 year after CABG (sensitivity = 75%, specificity = 86%, PPV = 62%).^766 , 773^

– A QRS score ≤ -3 mm predicts multivessel coronary stenosis 1 month after MI.^774^

– A QRS score < -4 mm in patients with CAD was an independent predictor of cardiac mortality (RR: 11.7; 95% CI: 2.5-55.4; p=0.002).^768^

### 4.19.3. Raxwal and Morise Score

The Raxwal and Morise score is a post-ET score to assess the probability of CAD in patients of both sexes, symptomatic or asymptomatic. Its calculation involves a sum of points, as shown in Table 35.^775 , 776^

CAD probability classification according to Raxwal and Morise scores:

– Low probability: 0 to 39 points.

– Intermediate probability: 40 to 60 points.

– High probability: >60 points.

A study with 4,640 patients (mean age 50 years, 53% males) with no known CAD undergoing ET to assess chest pain found that the Raxwal and Morise scores and the Duke score adequately stratified patients into low, intermediate, and high risk categories (p<0.00001). In this study, the Raxwal and Morise scores had the best prognostic value for all-cause mortality.^776^

## 5. Test Cessation Criteria

Overall, test cessation should be driven by symptoms, physical examination findings, cardiovascular, respiratory, and ECG variables, failure of ECG monitoring, and any other conditions considered to pose a risk of serious intercurrent events (Table 36).

## 6. ET Reporting

Immediately after the end of the recovery phase, the physician performing the ET shall analyze and interpret all pre-ET data, symptoms, normal and abnormal physical examination findings, ECG variables, measurements and recordings obtained, intercurrent events, scores, and information of prognostic relevance. Based on this information, the ET report must be written, containing the minimum requirements listed in this section and divided as follows:

1) Description of general ET data.

2) Observed, measured, and recorded data.

3) Descriptive report of the ET.

4) Conclusions.

5) ECG recordings.

### 6.1. General Information

The ET report should begin with a general description of the patient, their health condition, indications for ET, and the ergometer/protocol selected:

1) Patient identification: name, sex, weight, height, BMI, medical record number in the ET system (number or code assigned to the ET).

2) Patient’s health condition: current medications (note whether the requesting physician has complied with the instructions to withhold medication); risk factors for CVD; pre-test risk score.

3) Indication for the ET and/or corresponding ICD code.

4) Ergometer, exercise protocol, and ECG recording system (12 or 13 leads, electrode position). In step protocols, a detailed description of all variables should be provided: duration (minimum, average, and maximum); load (initial and final); and/or speed (initial and final) and grade (initial and final).

5) Additional optional notes or remarks considered relevant, such as results of previous ETs, pacemaker/ICD model if present, etc.

### 6.2. Observed, Measured, and Recorded Data

After the general data section, the report must present the data obtained at all phases of the ET (rest, exercise, and recovery):

1) Tables containing SBP and DBP measurements (with respective HRs and associated symptoms) and ECG records (with HR, METs, BP, and DP at the time of recording).

2) The use of graphs to evaluate BP (systolic and diastolic), HR, and ST-segment behavior during the test is recommended.

3) Table containing information about any ET-emergent symptoms and their timing of onset and improvement (noting the BP, HR, and DP at the time if possible).

4) Data obtained on: HR at rest; predicted HRmax; actual achieved HRmax or HRpeak; FAI; double product; score (numeric or interpretative) on the perceived exertion scale (Borg or modified Borg); VO_2_/METs (predicted, achieved, and percentage); functional classification.

5) Use of a post-test risk score is recommended. A caption or legend for interpretation of the score result should be included.

6) Any other measurements, data, tables, and graphs deemed relevant: behavior of ECG variables such as QTi, QTc, QRS score, etc.; scales used to quantify angina, dyspnea, and intermittent claudication; pacemaker and/or ICD behavior.

7) Additional tests that can add diagnostic and prognostic value to the ET: oximetry; ankle-brachial index; blood biomarkers.

### 6.3. Descriptive Report

A systematic, descriptive and interpretive report of the ET should be written, briefly presenting the behavior of the data and variables obtained during all phases of the test. The information necessary for proper description and interpretation of an ET is contained in this Guideline.

The descriptive report must contain:

1) Relevant data from the patient’s pre-test interview (targeted history and review of systems) and physical examination (general and specific).

2) A brief note on how well the patient adapted to the ergometer and protocol.

3) The timing of and reason(s) for test cessation.

4) A brief description of clinical responses (symptoms, signs, physical examination findings, exercise tolerance) and their possible interpretations. If an adverse event occurs, a detailed report thereof must be provided.

5) Description of the hemodynamic responses (HR, BP, DP, and other relevant parameters) and interpretation of results.

6) Description of ECG responses. When abnormal, include their respective interpretations:

– Waves and intervals: P wave, PR interval, Q wave, R wave, S wave, QRS complex, T wave, U wave, QT interval, QTc.

– ST-segment: depression, elevation, pseudonormalization, or absence of changes. Describe timing of onset, the greatest magnitude reached, if there were associated symptoms, timing of normalization, and whether criteria for ischemia were met.

– Normal atrioventricular conduction or AV conduction disorder (pre-existing or exercise-induced).

– Intraventricular conduction or IV conduction disorders (pre-existing or exercise-induced).

– Arrhythmias: timing of occurrence, any associated symptoms, hemodynamic repercussions, behavior (changes in density and complexity at all phases of the ET) and timing of resolution (if resolved).

7) Interpretive description of indirect metabolic assessment: VO_2_/METs, FAI, functional classification, etc.

8) Any comments deemed pertinent regarding pre-test risk score data. If using a post-test risk score (optional) and ET prognostic variables (if applicable), include any relevant comments regarding those as well.

9) Any noteworthy results obtained regarding the following conditions:

– Patient’s specific clinical picture (pre-existing diseases, physical and psychological limitations).

– Effect and interference of any current medications.

– Limitations to/interference with interpretation of the ET variables.

Including exercise prescriptions in the ET report, based on the physical performance achieved during the test, is discouraged. Exercise prescription is the sole responsibility of the referring physician.

Suggestions:

– Include a glossary of terms and abbreviations found in the ET report, to facilitate understanding.

– When relevant, include interpretation of and comparison with any previous ETs.

– Use an exercise test system/software that facilitates preparation of the descriptive report by presenting in an orderly manner all information that might be included and using pre-programmed language to standardize reports, while still allowing editing and individualized descriptions.

– Include a supplemental report on any additional tests performed at the time of the ET (i.e. laboratory tests, biomarker measurements).

### 6.4. Conclusion

The conclusion must present, in a concise and clear manner, information related to the indication for performing the test and any abnormal parameters relevant to diagnosis and prognosis. These include:

1) Clinical response.

2) BP response, based on the concepts presented in Table 32.

3) Chronotropic response, based on the concepts presented in Table 31.

4) Arrhythmias.

5) ECG response, including ventricular repolarization.

6) Functional classification.

7) Limitations to/interference with interpretation of the ET variables.

Use of the following expressions is not recommended: “positive test”, “negative test”, or “inconclusive test”. These expressions are vague, generally restricted to a single variable (ST-segment), and disregard the nuanced interpretation of multiple parameters/variables needed for adequate diagnosis and prognosis.

### 6.5. ECG Recording

The ET report needs to contain ECG recordings that demonstrate the ECG progression across all phases (rest, exercise, and recovery) and correspond to the pertinent information in the descriptive report. It is suggested that recordings be obtained in 12 or 13 leads and that continuous rhythm recording (generally in lead II) be performed for documentation of arrhythmias. Each ECG record must describe the ET phase, the velocity and amplitude of the tracing, the HR and, if available, the measured BP.

Recording of automatically calculated complexes is optional. Avoid in case of artifacts, large oscillation of the isoelectric baseline, and ventricular arrhythmias, all of which interfere with automated calculation.

One suggestion is to incorporate into the report a table containing any automated measurements of waves, intervals, and ECG segments, as long as they are relevant and consistent with the ET diagnosis.

## 7. Other Diagnostic Tests Performed Simultaneously with or in Addition to ET

### 7.1. Ankle-brachial Index

Calculation of the ankle-brachial index (ABI) is a noninvasive test for the diagnosis and follow-up of peripheral artery disease (PAD) of the lower extremities. ABI is a strong marker of atherosclerosis, functional impairment, cardiovascular risk, and mortality.^778 , 779^It can identify patients at risk of complications in the lower extremities, and can thus inform optimized preventive management of these patients.^780^

ABI may be performed:

– At rest, during a specialized clinical examination. It is recommended in patients with clinical suspicion or physical examination findings consistent with PAD (Class of Recommendation: I; Level of Evidence: B), i.e. lower-limb pain on exertion; intermittent claudication; loss of pulses and/or onset of leg bruits; and failure of wound healing on the lower extremities. It is also recommended in patients at risk of PAD: those with CAD or atherosclerotic obstruction of arteries elsewhere in the body (i.e. carotid; subclavian); chronic kidney disease; HF; asymptomatic men and women aged >65 years, or <65 years if at high risk for CVD, or >50 years if with family history of PAD.^780 - 785^

– Post-exertion, as part of a treadmill test (post-exercise ABI), it is useful for establishing the diagnosis of PAD in symptomatic patients when the ABI at rest is normal or borderline abnormal. ABI allows objective quantification of functional limitations attributable to symptoms, as well as improved risk stratification. Indications are listed in Table 37.^780 , 781 , 786 - 788^

#### 7.1.1. Method of ABI Measurement

When performed as part of a treadmill ET, ABI measurement comprises two phases: at rest and post-exercise.

**7.1.1.1. Resting ABI**

The resting ABI is measured with the patient in the supine position with the head and heels supported, at a comfortable ambient temperature (19-22°C), after 5 to 10 minutes of rest. Systolic blood pressure measurements are performed in all four limbs using continuous-wave Doppler ultrasound (5-10 MHz):^780 , 781 , 791^

– The cuff size should be suitable for the circumference and width of the limbs (i.e. cover at least 40% of the limb circumference)

– The cuff should be placed just above the ankle (avoiding areas with wounds), with its lower edge 2 cm above the uppermost point of the medial malleolus

– Ultrasound transmission gel should be applied over the Doppler transducer, which is then placed over the wrist area, at an angle of 45° to 60°. The transducer is moved until the clearest sound of arterial flow is detected. The cuff is then progressively inflated to 20 mmHg above the pressure level at which the sound disappears. It should be deflated slowly to detect the reappearance of the sound, and the corresponding SBP recorded.

– Measure and record SBP in the brachial arteries of both arms and in the posterior and anterior tibial arteries of both ankles (or dorsalis pedis of the same limb if the pulse is absent in one of the tibial arteries).^796^

– To calculate the ABI, use the highest pressure found among the measurements of the arteries of each leg.

The ABI for each leg is calculated by dividing the highest ankle SBP by the arm SBP on the same side.^780^ABI values must be recorded to two decimal places.^781^


$$\text{Resting ABI}=\frac{\text{Highest SBP measured in one of the ankle arteries}}{\text{Highest SBP measured in the brachial arteries}}$$

ABI: ankle-brachial index; SBP: systolic blood pressure (measured by Doppler ultrasound). Numerator: highest systolic blood pressure measured in one of the arteries (posterior and anterior tibial, or dorsalis pedis of the same limb if the pulse is absent in one of the tibial arteries).

Denominator: highest brachial artery pressure measured in both arms.

Note: record which ankle artery had the highest SBP, as the measurement for calculation of post-exercise ABI should be performed on the same artery.

Automatic blood pressure measurement devices are, in most cases, invalid for measuring ankle SBP, and generally overestimate BP values when low.

The ABI measured at rest must be interpreted according to the purpose of the test:

– For diagnosis of PAD, interpret each leg separately (one resting ABI per leg).

– For CV risk stratification, consider the lowest ABI of the two legs.

Resting ABI may be classified as:^780 , 781^

– Abnormal (low): ≤0.90. In symptomatic patients, this is a diagnostic criterion for PAD with >50% stenosis and is associated with an increased risk of coronary events, CV mortality, and all-cause mortality at 10 years.^781 , 797^

– Borderline low: 0.91-0.99. PAD cannot be ruled out. If there are symptoms suspicious for exercise-induced ischemia of the lower limbs, post-exercise ABI measurement is advised.^791^

– Normal: 1.00-1.40.

– High: >1.40. Represents arterial stiffening (intima-media calcification), leading to incompressibility of the arteries. This is more prevalent in older adults (especially those with diabetes or CKD) and has a lower sensitivity for the diagnosis of PAD, but is associated with a higher risk of CV events and mortality.^778^

Resting ABI performs reasonably well for diagnosis of PAD, with a sensitivity of 61% to 73% and a specificity of 83% to 96%.^790 , 798^Sensitivity is lower in patients with advanced diabetes and/or CKD, due to the frequent occurrence of arterial calcification in these populations.^785 , 791^

**7.1.1.2. Post-exercise ABI**

In healthy patients, treadmill exercise leads to a progressive increase in SBP in the central circulation and upper limbs. However, due to physiologic vasodilation of the lower-limb muscles, there is a decrease in SBP at the ankle level and, consequently, a slight decrease in the overall post-exercise ABI (on average, 5% of the resting ABI value). In patients with PAD, a >20% reduction occurs.^791 , 799^

At 1 to 2 minutes of recovery, the lower-limb SBP increases rapidly, returning to pre-exercise levels. Return to at least 90% of the resting ABI value in the first 3 minutes of recovery rules out PAD with 94% specificity.^791 , 799^

In occlusive PAD (typically of proximal vessels), ankle pressure decreases more markedly with exertion, and the time taken to return to resting ABI values is prolonged, proportionally to the severity of PAD.^791^

#### 7.1.2. Patient Preparation and Measurement Technique

In addition to the general instructions for ET, it is recommended that patients:

1) Wear clothes that allow easy exposure of the arms and legs for cuff placement and BP measurement.

2) Refrain from wearing shorts or pants that are tight-fitting at the legs or made of compression fabrics, such as elastane (Spandex®/Lycra®): fitness shorts, training shorts, “run-dry” or “quick-dry” shorts, etc.

A baseline SBP measurement is obtained in the arms and ankles at rest.

ET adaptations:^797 , 800^

1) The ET must necessarily be performed on a treadmill.

2) A fixed-load protocol must be used, with a walking speed of 3.2 km/h (2 mph), 10% to 12.5% grade, and maximum duration of 5 minutes (so as not to impair assessment of ABI recovery time).^801^For patients with significant limitations, the speed and/or grade may be reduced.

3) The test should be terminated early if the patient complains of lower-limb pain and/or absolute claudication (preventing further exercise).

4) There must be no active recovery.

5) All clinical, hemodynamic, and ECG parameters, cardiorespiratory fitness, and scores must be evaluated as recommended elsewhere in this Guideline.

6) All symptoms must be recorded. Use of a scale of perceived exertion (Borg or modified Borg) and an intermittent claudication scale is recommended.

7) After test cessation, recovery should be passive, with the patient in the supine position. SBP should be immediately measured in both arms and both ankles, on the sides that read the highest SBP for calculation of the resting ABI. SBP measurements and ABI calculation should be repeated bilaterally at least 3 times and/or until lower-limb SBP returns to the baseline value. All SBP values obtained should be recorded, alongside the respective measurement times and any associated symptoms. Post-exercise ABI is calculated according to the following equation:


$$\text{Post-exercise}ABI=\frac{\text{SBP measured in the same artery as the resting}ABI}{\text{Highest SBP measured in the brachial arteries}}$$

ABI: ankle-brachial index; SBP: systolic blood pressure (measured by Doppler ultrasound).

Note: ankle artery with the highest SBP used to calculate resting ABI. Measure in both ankles and calculate post-exercise ABI for the two limbs. Denominator: highest brachial artery pressure measured in both arms.

Calculation of the percent change in ABI in each limb (individually) can be calculated through the following equation:


$$\text{Percent change in}ABI(\%)=\frac{\text{Post-exercise ABI}-\text{Resting ABI}}{\text{Resting}ABI}\times 100$$

The criteria for diagnosing PAD have not yet been fully standardized. It is recommended that the following be considered “abnormal” test results consistent with a diagnosis of PAD:^780 , 781 , 791 , 799^

– Post-exercise ABI <0.90 (70% to 88% sensitivity and 72% accuracy for PAD)^788 , 791 , 795 , 802^or

– A >20% drop in post-exercise ABI values compared to resting values for the same limb (67% sensitivity for risk stratification of mortality and CV events)^800 , 802 , 803^or

– A drop >30 mmHg in ankle SBP in relation to the baseline SBP value.

One limitation of post-exercise ABI is when the resting ABI is abnormally high due to an incompressible ankle artery (SBP >250 mmHg), which makes it impossible to adequately detect PAD by the post-exercise measurement.

Particular features of post-exercise ABI:

– One study demonstrated that, in individuals with normal resting ABI, the addition of post-exercise ABI identified 25% more lesions with significant stenoses (>75%).^802^

– A study of 619 patients (mean age 64.2 years; 64% men) with suspected PAD, normal resting ABI, and post-exercise ABI <0.90 showed a sensitivity of 81.7%, specificity of 94.7%, PPV of 84.8%, and NPV of 94.4%. A percent change in ABI >20% showed 70.4% sensitivity, 83.4% specificity, 47.2% PPV, and 93% NPV.^787^

## 7.2. Noninvasive Oximetry

Noninvasive oximetry (pulse oximetry) is a technique that aims to continuously monitor tissue oxygenation. It contributes to the detection of hypoxia and hemodynamic instability, both at rest and during exertion, which could go unnoticed on clinical evaluation alone. Noninvasive oximetry is considered a medical procedure, and as such is covered in the Hierarchical Brazilian Classification of Medical Procedures (code 4.14.01.51-4).^804^

A conventional pulse oximeter continuously, indirectly, and transcutaneously monitors the oxygen saturation of hemoglobin in arterial blood (SpO_2_) through spectrophotometric methods. The determination of SpO_2_is based on the average of a series of measurements compared to an internal calibration curve (SpO_2_values <70% are unreliable).^805 , 806^

Multi-wavelength pulse oximeters allow measurement of carbon monoxide (CO) bound to hemoglobin (Co-oximetry) and determination of carboxyhemoglobin (CO_2_Hb) and methemoglobin (MetHb) concentrations, better defining potential causes of hypoxia/hypoxemia.^807^

The oximeter also records the plethysmographic waveform, which corresponds to the pulsatile signals associated with each heartbeat. The plethysmographic waveform plays an important role in assessing signal quality and interpreting saturation data (Figure 16):^808^

– When normal, the waveform shows a clear dicrotic notch synchronized with each heartbeat.

– In low-perfusion conditions, the waveform is typically sinusoidal, with low amplitude and no notches (but still synchronized with the heartbeat).

– The presence of arrhythmias can make the waves appear irregular and, in case of PVCs, the waveform may exhibit reduced amplitude.

– Artifacts (noise or movement) lead to an irregularly shaped wave, with multiple notches and no clear relationship with the heartbeat.

The plethysmographic waveform bears a resemblance to invasive blood pressure tracings, but is not directly analogous to either blood pressure or cardiac output.

Pulse oximetry can be performed during all phases of ET (rest, exercise, and recovery), as is already standard in CPET. It is suitable for pediatric and adult patients alike.

The addition of oximetry increases the safety of ET and allows a more accurate assessment of symptoms of dyspnea and fatigue in specific populations. The indication to perform pulse oximetry in association with ET does not modify its original indications and degrees of recommendation.^809^

General indications for adding pulse oximetry to ET include: patients complaining of dyspnea on exertion; lung diseases; cardiomyopathies and other heart diseases; HF; valvular heart disease; and post-COVID condition.^228 , 810^Key specific indications are listed in Table 38.

### 7.2.1. Equipment

The pulse oximeter should ideally be capable of measuring light absorption at various wavelengths, determining both O_2_saturation and CO-oximetry, and recording the FR and plethysmographic waveform. Prefer devices that have been calibrated and validated to obtain measurements during physical exertion. The oximeter can be a standalone tabletop model or integrated into the ET system/cart. It should preferably be capable of communicating with the computer/cart software, allowing export of the data necessary for inclusion in the ET report.^804^

Oximetry sensors must be appropriately sized for the patient (pediatric vs. adult), and the fixation method must be appropriate for the selected body site (i.e. fingertipe, earlobe). The sensor cable must be flexible, sized appropriately to allow patient movement, and capable of withstanding strain.^814^It is recommended that SpO_2_behavior, FR, and the plethysmographic waveform be monitored continuously throughout the ET (whether on the computer/cart or directly on the oximeter display). The portable fingertip oximeters so widely used in clinical practice are not recommended for ET/CPET.

### 7.2.2. Procedures for Noninvasive Oximetry 804

– Explain and guide the patient through the oximetry procedure.

– Make sure that the area where the sensor will be placed is clean.

– When using an earlobe sensor, remove all ear jewelry that could interfere with proper sensor placement and fit.

– When using a fingertip sensor (preferably on the index finger), remove any nail polish and hand jewelry. Do not place the sensor on the limb where BP is to be monitored.

– Make sure that the sensor is properly attached (neither too loose nor too tight).

– Turn on the oximeter and wait for its self-calibration and initial measurement.

– Wait for the oximeter to detect pulse and FR and calculate the oxygen saturation. If artifacts or signal fluctuations occur, reposition the sensor and/or switch to another body site (i.e. finger to earlobe).

During the exercise phase of the ET:^815^

– When using a fingertip sensor, discourage the patient from grasping the treadmill/ergometer handrail too tightly, as this introduces noise.

– If frequent artifacts (noise and/or movement) are recorded, adjust the sensor and, if necessary, reposition it.

– If markedly altered values are obtained on sequential measurements (i.e. not caused by artifacts), examine for symptoms, measure RF, and perform pulmonary auscultation to correlate with the oximetry findings.^816^

### 7.2.3. Interpretation of Findings

FR should be interpreted according to the patient’s age group and work load achieved. The plethysmographic waveform should be interpreted during monitoring as described earlier in this section.

Regarding baseline SpO_2_evaluation (at rest, on ambient air), consider the following classification: ≥97% – normal lung function; 96-91% – mild to moderate abnormal lung function; ≤90% – hypoxemia/hypoxia.

Baseline SpO_2_values <94% are usually found in smokers and in patients with chronic lung disease, chronic heart disease, or HF. In the event of hypoxia, assess the patient’s symptoms and clinical condition and determine whether test cessation would be warranted.^219^

In asymptomatic, apparently healthy adults, SpO_2_fluctuations between 1% and 4% are to hbe expected during exertion. This fluctuation is expected to disappear in the first few minutes of recovery.

A ≥5% drop in SpO_2_has been associated with baseline conditions (male sex, older age, smoking), in addition to lower HRmax and VO_2_.^816^

In both pediatric and adult patients, severe hypotension, low cardiac output, vasoconstriction, and hypothermia reduce the pulsatile volume of blood in the extremities, causing desaturation.^817 - 819^

In the pediatric population, progressive desaturation (<90%) or a 10-point drop in saturation at rest with concomitant symptoms mandates test cessation.^820^

In some situations and conditions, pulse oximetry may yield unreliable readings (Table 39).^817^

## 7.3. Biomarkers and Other Laboratory Tests

Performing laboratory tests and measuring biomarkers in addition to ET/CPET has specific indications. When these will be done concomitantly, it is recommended that the procedure be performed at a hospital-level facility. Measurement of blood lactate and arterial blood gases are the most frequently performed laboratory tests.

In patients with suspected stable CAD, ultra-high sensitivity cardiac troponin T (u-hscTnT) has demonstrated clinical utility due to its predictive value both alone as well as when performed in addition to ET for the diagnosis of CAD.^821 - 824^A u-hscTnT value >6.0 ng/L at rest was predictive of CAD (RR: 2.55; 95% CI: 1.40-4.65; p=0.002) and, when elevated during ET, increased the accuracy to 0.71, demonstrating its value as a marker of MACE risk.^822 , 825^

Figure 17 illustrates other potential biomarkers that have been studied in the last decade. The body of scientific evidence on the utility, reproducibility, and applicability of biomarkers continues to grow, as do prospects for their incorporation into clinical practice.^821 , 826 - 830^

Laboratory tests may be performed:

– Outside of the context of ET, often in combination or as part of routine screening/testing.

– During the pretest interview, to assess the patient’s baseline status.

– During exercise, which requires placement of a venous and/or arterial line during the pretest period and adequate setup for sample collection. The purpose is to assess the acute effect of exercise on the biomarkers of interest.

– During recovery, to evaluate the late effects of exercise or confirm return to baseline condition.

– During two or more ET phases (pretest and exercise; pretest and recovery; exercise and recovery; at all three phases).

Some of these biomarkers are widely used in clinical practice; others still lack scientific evidence to support their clinical use.

### 7.3.1. Blood Lactate

Measurement of serum lactate levels is one of the best available indicators to assess cell metabolism, both in patients with comorbidities and in athletes. When added to ET, serum lactate measurement can optimize the assessment of conditions such as CAD, COPD, chronic renal failure, and multiple sclerosis. It also contributes to optimal exercise prescription, particularly in the context of competitive sports and rehabilitation (Table 40).^831 , 832^

In patients with comorbidities, despite the complexity of the biochemical pathways related to blood lactate kinetics, serum lactate levels have proven to be a better prognostic predictor than variables derived from tissue oxygenation and oxygen consumption. In these patients, part of the increase in serum lactate may be associated with hypoxemia.^833^

The normal resting blood lactate concentration is <2 mmol/L (18 mg/dL). Patients who complete an ET with progressive, incremental effort load until voluntary exhaustion exhibit significantly increased serum lactate (≈8-10 mmol/L). Exercise is considered maximal when the serum lactate concentration in adults is >9 mmol/L (healthy individuals) or >5 mmol/L (with comorbidities).^834 , 835^However, it is during recovery (between 3 and 8 minutes) that the highest lactate levels are observed (≈15-25 mmol/L).^836^

In sports medicine, serum lactate measurement (which may be combined with CPET) has been most often indicated for serial performance assessment, adjustment of training loads, and determination of lactate threshold. Suggestions:

– Perform protocol on a cycle ergometer with an initial load of 40 W and load increments of 40 W/4 min.^835^

– If possible, use a protocol and ergometer seeking to simulate the sport practiced by the subject (this includes allowing exercise to continue for longer periods than usual).^837 - 839^

– Percentile tables for serum lactate level according to the exercise intensity achieved and/or sport practiced by the patient require validation in the Brazilian population.

In competitive athletes, additional serum lactate analyses can be carried out, i.e. behavior of the serum lactate curve, recovery pattern, maximum lactate concentration in steady state, and minimum lactate test, among others.^840 , 841^

### 7.3.2. Arterial Blood Gas Analysis

Arterial blood gas analysis (ABG) is an invasive diagnostic test that measures blood pH, partial pressures of carbon dioxide (PaCO_2_or pCO_2_) and oxygen (PaO_2_), bicarbonate ion (HCO_3_) and oxyhemoglobin saturation. Its key purpose is evaluation of acid-base balance.

Baseline (resting) ABG allows assessment of the adequacy of ventilation, acid-base balance, and oxygenation; evaluation of the patient’s response to therapy; diagnostic investigation; and monitoring of the severity and progression of cardiorespiratory and metabolic diseases. General indications for ABG in ET/CPET include:^842 , 843^

– More accurate assessment of dyspnea.

– Differential diagnosis of hypoxemia.

– Conditions in which the addition of ABG will aid in the diagnosis and increase the safety and accuracy of assessment. Examples: advanced lung diseases (COPD and emphysema); in combination with resting spirometry.

– In high-performance athletes (especially masters athletes).

The indication to perform ABG in addition to ET/CPET does not modify its original indications and degrees of recommendation (Table 40).

Blood samples for ABG can be obtained through:^844^

– An arterial line in an upper limb (generally in the radial artery) which is not being used for BP measurement. The sampling technique should follow standard procedures for ABG collection, followed by a heparin flush in order to allow further collection from the same line if necessary.

– Arterialized earlobe blood is not recommended, as it correlates poorly with PaO_2_and PaCO_2_.^845 , 846^

The following ABG parameters can be used as criteria to ascertain whether an ET/CPET was maximal:^847 , 848^

– pH: <7.25.

– Base excess: <9 mmol/L (healthy individuals), <5 mmol/L (with comorbidities).^134 , 849^

Arterial blood gas analysis is necessary to calculate the dead space to tidal volume ratio (VD/DT) corrected for mechanical dead space during CPET. This correction produces quantitatively and qualitatively different results, which can have a major impact on the interpretation of V/Q mismatch.^850 - 852^

Particular aspects of ABG when performed in addition to ET/CPET:

– In HF, there may be a progressive increase in PaCO_2_with exercise, due to poorly ventilated lung area. In severe HF, an increase in VD/DT and PaCO_2_in the absence of hypoxemia it associated with massive V/Q mismatch.^850 , 853^

– In HFpEF, V/Q mismatch and worsening of gas exchange efficiency may occur, a reflected by an increase in VD/DT.^850 , 851^

– In smokers, a high VD/DT ratio (with compensatory increases in minute ventilation) is associated with a greater degree of dyspnea and exercise intolerance.^854^

## 8. Particular Aspects of ET Performance and Interpretation in Specific Clinical Conditions

### 8.1. Dextrocardia/Situs Inversus

Dextrocardia (DxC) is a congenital anomaly in which the heart is positioned in the right side of the chest, with mirror-image inversion of its chambers ( *situs inversus* ). The prevalence of DxC ranges from 1 in 6,000 to 1 in 35,000 births. When there is associated transposition of the abdominal organs, it is known as *situs inversus totalis* .^865 , 866^

It is generally asymptomatic, but may be associated with congenital heart malformations. The most common are atrioventricular discordance with right ventricular outflow tract obstruction in *situs solitus* and double-outlet right ventricle with outflow tract obstruction in *situs inversus* .^867^

DxC should not be confused with dextroposition of the heart, a situation in which there is no inversion of the cardiac chambers and the heart is simply shifted into the right side of the chest (due to, i.e. pneumonectomy or a large left pleural effusion). Dextroposition does not require any modification to the usual ECG lead placement for ET.^868^

DxC is usually diagnosed in childhood, and is usually reported by the patient during the pre-ET interview. Its recognition on physical examination (auscultation of heart sounds and apex in the right side of the chest) and/or resting ECG helps prevent potential diagnostic errors.

DxC should be suspected if, a negative P wave, QRS and T wave are seen in lead I on resting ECG. In such an event, the clinician should rule out an inadvertent transposition of the upper limb electrodes and verify the progression of R-wave amplitude in the precordial leads (in DxC, the R waves do not increase in amplitude from V1 to V6).^869^

In patients known to have DxC, the following measures are recommended:

1) Document the resting ECG with the electrodes in the classic positions, considering that:

1.1) In *situs inversus* , the following phenomena are observed:^870 , 871^

– Inverted P wave in leads I and aVL and a positive P wave in aVR due to the mirrored (left) location of the sinus node.

– Inverted ventricular activation with negative QRS complex and negative T wave in lead I. The right precordial leads mirror the left precordial leads of a normal heart.

– Q waves are present in the right precordial leads, due to right-left septal depolarization secondary to mirror inversion of the heart.

1.2) In *situs solitus* , the following are observed:^870 , 872^

– Normal progression of atrial depolarization, regardless of cardiac location (right or left side of the chest).

– Counterclockwise ventricular depolarization, usually with Q waves in lead I, aVL, and left precordial leads (due to appropriate septal depolarization).

1.3) In *situs ambiguus* and right atrial isomerism (heterotaxy), P waves can have different origins, as they represent the activity of bilateral atrial pacemakers. However, the P wave axis may still be normal if the right sinus node is the dominant pacemaker. Due to the absence of a functional sinus node, patients with left atrial isomerism have an ectopic atrial pacemaker with a generally abnormal P wave. With age, this ectopic pacemaker undergoes progressive deceleration, and most patients require permanent pacemaker implantation.

2) In ET, ECG monitoring must be adjusted for each type of DxC:

2.1) In *situs inversus* , both the limb electrodes and the precordial leads must be reversed:

– In the position of V2 (4th intercostal space, on the left parasternal line), place electrode V1 (V1R).

– In the position of V1 (4th intercostal space, on the right parasternal line), place electrode V2 (V2R).

– V3R: between electrodes V2R and V4R.

– V4R: in the 5th right intercostal space, on the right midclavicular line.

– V5R: in the 5th right intercostal space, on the anterior axillary line.

– V6R: in the 5th right intercostal space, on the midaxillary line.

– The arm and leg electrodes are to be reversed (right to left and vice versa).

– In 13-lead systems, lead CM5 (manubrium) is placed as usual.

– It is mandatory that the clinician note in the report the inversion of the electrodes and add the letter “R” or “D” (for *right* or *dexter* ) after each lead.

2.2) In *situs solitus* with DxC, the precordial lead electrodes must be inverted (V1 to V6 placed over the right side of the chest, as in *situs inversus* ), while the limb electrodes should remain unchanged.

2.3) In *situs ambiguus* , no change in the conventional ECG electrode placement is recommended.^872^

ET performed with the electrodes in the positions recommended above allows appropriate evaluation of all ECG variables of interest and their respective interpretations.

The prevalence of arrhythmias in patients with DxC is significantly higher than in the general population (RR: 2.60; 95% CI: 1.67-4.06; p<0.001), with the most common being atrial fibrillation or flutter (RR: 3.06; 95% CI: 1.02-9.18; p=0.046).^873^

Close attention should be paid to the possibility of cardiac malformations associated with DxC and their possible interference with the hemodynamic, functional, and electrocardiographic responses to ET.^870 , 874 , 875^

### 8.2. Chagas Disease/Chagas Cardiomyopathy

Chagas disease (ChD) remains a serious public health problem worldwide. Appropriate measures are required for its diagnosis, treatment, and follow-up. In Latin America, an estimated ≈6 million people are infected, of whom 30% to 40% may have the cardiac form of the disease, with a mortality rate of ≈24.5 per 1,000 patients/year.^876 , 877^

ChD has a broad spectrum of clinical presentations: the indeterminate, clinically inapparent form; Chagas cardiomyopathy (ChC), with cardiac involvement, ECG changes, and positive serology for *Trypanosoma cruzi* ; and dilated ChC, with HF and ventricular dysfunction. Cardiac arrhythmias (arrhythmic syndrome) may be the sole manifestation of ChC, often occurring in combination with HF and/or thromboembolic events.^878 , 879^

ET is useful in all forms of ChD, not least due to the frequent presence of comorbidities. General indications for ET in ChD:^879 , 880^

– Assessment of functional capacity, chronotropic response, and BP behavior.^881 - 883^

– Assessment of symptoms, including chest pain.^884^

– Assessment of comorbidities (i.e. hypertension, CAD, etc.).

– Detection and evaluation of arrhythmias (suspected or known) and atrioventricular conduction disorders.^885^

– Risk stratification for sudden death (including as part of the Rassi score criteria).^878 , 886 , 887^

– Serial evaluation, including as part of a rehabilitation program, to inform exercise prescription.^888^

– Disability/worker’s compensation and/or work capacity assessment.^889^

Particular aspects of ET in ChD:

– On resting ECG, no single electrocardiographic finding is pathognomonic; multiple changes are common, including those resulting from comorbidities.^890^

– Resting ECG changes potentially associated with poor prognosis: pathologic Q waves (mimicking prior MI), low-voltage QRS complexes, and primary T wave changes.^886 , 891^

– Functional capacity is relatively preserved in the indeterminate form and in chronic ChC, even in the presence of ECG changes and depressed ventricular function.^892 , 893^Functional impairment is a predictor of decline in cardiovascular function, including in the indeterminate form and in the early stages of ChC. In dilated ChC, VO_2_peak is an independent predictor of death.^883 , 885 , 894^

– The exercise protocol should be adapted to the patient’s functional class, which includes the use of attenuated protocols as necessary.

– Decreased chronotropic response and/or chronotropic incompetence are considered manifestations of autonomic dysfunction secondary to ChD. This dysautonomia is generally associated with the density and lethality of ventricular arrhythmias.^895 , 896^

– Patients with indeterminate ChD and isolated Chagas gastrointestinal disease may still have chronotropic incompetence or exercise-induced ventricular arrhythmias.^881 , 897^

– Exercise-induced ST-segment changes may be secondary to LV apical aneurysm, especially in ChD chest pain syndrome. However, these abnormalities may also be inherent to ChD (acute or chronic).^898^

– Occurrence of precordial pain with ischemic ST changes on an interpretable ECG has a PPV of 100% for obstructive CAD.^884^

– Baseline ECG changes associated with intraventricular conduction disorders generally make it impossible to properly assess ischemia in the ST-segment.^884^

– Changes in heart rhythm on baseline ECG are predictors of severity/functional class in ChC, while exercise-induced changes correlate with CV mortality.^899 , 900^

– NSVT, especially in patients with LV dysfunction, is a predictor of sudden cardiac death.^877 , 901 , 902^

– Exercise-induced ventricular arrhythmia or increased ventricular arrhythmia density as compared to baseline ECG (>10%) are associated with risk of CV death.^877 , 903^

– Sudden death in ChC, usually precipitated by exertion, can be caused by ventricular tachycardia or fibrillation, asystole, or complete AV block.^904^

– For exercise prescription and serial evaluation in the context of a rehabilitation program, ET should be performed without discontinuing or withholding any usual medications. This is especially important for patients on negative chronotropic agents, such as beta-blockers, digitalis, or antiarrhythmics, to mimic the condition they will be in during physical training sessions. It is suggested that monitoring of oxygen saturation and cardiometabolic variables (i.e. CPET) be performed as well, to better quantify possible limitations.^905^

– For exercise prescription and serial evaluation in the context of a rehabilitation program, ET should be performed without discontinuing or withholding any usual medications (including negative chronotropic agents), to mimic the patient’s condition during physical training sessions.^888^

### 8.3. Peripheral Artery Disease

Peripheral artery disease (PAD) is characterized by atherosclerotic lesions of the arteries of the lower limbs that cause reduced blood flow, intermittent claudication (IC), and exercise-induced ischemic muscle pain, relieved by rest (usually within 10 minutes).^175 , 780^Atypical manifestations such as cramps and exercise limitation may occur without any symptoms clearly associated with the lower limbs.^906^It is estimated that, worldwide, PAD affects more than 200 million people, and its presentation and natural history are influenced by sex, age, and ethnicity. PAD frequently occurs in smokers and patients with diabetes.^907^

IC occurs in 7.5% to 33% of these patients, progressively undermining walking ability, functional capacity, and quality of life. ABI at rest is the main test for diagnosing and establishing the severity of PAD, regardless of symptoms.^780 , 908^

Patients with a suspected or confirmed PAD are at increased risk of obstructive arterial disease elsewhere in the body, especially in the subclavian, coronary, and renal arteries. Patients with both PAD and CAD experience a worse natural history of PAD and increased CV and all-cause mortality.^175 , 176 , 909^

ET is generally indicated in PAD for assessment of claudication/lower-limb pain, quantification of ischemia is present, evaluation of functional capacity, risk stratification, exercise prescription, and optimization of therapy.^175 - 177 , 781 , 801^

Particular aspects of ET in PAD:

1) Follow pretest orientations closely, especially regarding smoking cessation and avoiding physical exertion before the test.

2) In the pre-ET phase, a vascular examination should be carried out, including palpation of lower-extremity pulses, auscultation for femoral murmurs, and inspection of the legs and feet. Pulses should be evaluated and classified according to amplitude: 0 = absent (no palpable pulse); 1 = decreased (barely palpable pulse); 2 = normal; 3 = increased. Particular attention should be paid to the presence of acute or chronic cutaneous lesions, especially in patients with diabetes (diabetic foot).^781^

3) An attenuated protocol is recommended:

– Fixed load (no speed or grade increments), at a of 2 mph (3.2 km/h) and 10% to 12% incline. This protocol may not allow accurate assessment of individuals with PAD and severe functional limitation, due to difficulty in maintaining effort at such a marked incline.

– Or, an incremental protocol, either step (i.e. Naughton) or ramp (with speed adjusted to ensure a small incremental load and little or no incline). When using a ramp protocol, the ET report should include the initial and final speeds and grades, as well as the planned load in METs/min, to allow later reproducibility of the protocol.

4) The patient should be instructed to hold onto the treadmill handrail if necessary. Whether the patient does so should be recorded in the report.

5) The test should cease when the patient developed exercise-limiting claudication and/or maximal tolerated pain in the lower limbs. The test may also be terminated due to other CV symptoms (i.e. chest pain, dyspnea, etc.) or any other parameters that would contraindicate continuation of exercise.^906^

6) Any symptoms should be recorded, as well as the timing of onset, affected body part (buttock, thigh, calf, other), and timing of improvement. Special attention should be paid to recording the time points of onset of any claudication (initial claudication) and of claudication that limits/prevents further walking (absolute claudication). Use of a scale of perceived exertion (Borg or modified Borg) and an intermittent claudication scale (see Figure 7) is recommended.

7) During recovery, the vascular examination should be repeated (see item 2), and the patient cleared or discharged only after returning to baseline condition.

8) The report must include the maximum pain- and/or claudication-free walking distance, the distance walked until onset of initial and absolute claudication, METs achieved, and the patient’s functional capacity.

Depending on the patient’s condition and the clinician’s judgment, a post-exercise ABI test, simultaneously or in addition to the ET, may be deemed necessary. The indications, methods, and interpretation of this test are described in Part 2, Section 7.1 of this Guideline.

Particular aspects of ET in PAD:

– Table 41 lists the main differential diagnoses of exertional lower-limb pain and claudication unrelated to PAD.

– Studies have shown that, in patients with PAD, ET variables are useful for risk stratification of MACE and CV mortality.^803 , 910^

– Decreased functional capacity on ET is a strong predictor of long-term mortality in patients with PAD, surpassing all classical risk factors (including smoking and HF) for risk stratification purposes.^177 , 911^

– Studies in patients with PAD who underwent ET for exercise prescription or optimization of physical exercise programs showed improvement in cardiovascular health (BP response, HR, HR recovery, and functional capacity) which correlated with improvement in walking performance.^781 , 912^

### 8.4. Parkinson’s Disease

Parkinson’s disease (PD) is the second most common neurodegenerative disorder worldwide, affecting 0.4% of people aged <40 years and about 1.6% of those aged ≥65. It is characterized by classic motor dysfunctions (bradykinesia, rigidity, rest tremor) and reduced parasympathetic and sympathetic activity, both at rest and during exertion.^913 , 914^

Autonomic and cardiovascular dysfunctions are common in PD and precede motor dysfunction by at least a decade. As PD progresses, patients usually experience fatigue, reduced functional capacity, and increasingly poor quality of life. The presence of Lewy bodies in the hypothalamus and sympathetic and parasympathetic centers impairs autonomic modulation, chronotropism, inotropism, and peripheral vascular resistance.^915 , 916^

In PD, abnormal BP regulation can manifest as:

– Orthostatic hypotension (OH), affecting ≈50% of patients.^917 , 918^

– Supine hypertension (SH), defined by an SBP >140 mmHg and/or DBP >90 mmHg when measured after 5 minutes of rest in the supine position; it affects 79% of patients and increases the incidence of target-organ damage (risk factor for stroke and MACE).^915 , 919^

– Presence of both SH and OH is common (95%).

Particular aspects of ET in PD:

1) Particular attention is needed to the possibility of SH and OH in the pretest phase.

2) The protocol must be carefully individualized. Use of attenuated protocols is suggested for patients with greater physical limitation.

3) In patients with symptomatic OH and/or a history of exertional syncope, ET is best performed on a cycle ergometer to reduce the risk of falls.

4) The patient should be encouraged to hold onto the treadmill handrail or cycle ergometer handlebars, not least to reduce tremor.

5) In early or mild PD, there may be no significant functional limitation or hemodynamic impairment.^167 , 920^

6) Gait behavior during ET should be recorded, including whether any freezing episodes (when walking and other voluntary movements may suddenly cease) occurred. The characteristic gait of PD involves festination, with small, shuffling steps, a decrease in cadence rate and walking speed, and abnormal range of motion.^921^

7) Patients with PD and OH generally have a reduced increase in BP in response to exercise, and may have exercise-induced hypotension. This BP response, which is associated with reduced vasoconstriction in the peripheral vasculature, can accentuate metabolic derangements and tissue/cerebral hypoperfusion, causing greater fatigue and lower exercise tolerance.^920 , 922^

8) In PD without OH but with reduced functional capacity, lower SBP peak, lower HRpeak, and depressed chronotropic response are generally present.^167 , 914 , 923^

Physical exercise constitutes a relevant nonpharmacologic, preventive and therapeutic approach to delay the development of PD, control PD symptoms (motor and otherwise) once established, and reduce the risk of CV events.^921 , 924 , 925^In these patients, ET is indicated for assessment of cardiorespiratory fitness and medical clearance/exercise prescription, including in the context of cardiovascular rehabilitation.^926 , 927^

### 8.5. Valvular Heart Disease

ET plays a relevant role in the evaluation of patients with valvular heart disease (VHD), contributing to the investigation of symptoms, optimization of therapy, indication of invasive treatment, and medical clearance for physical activity/exercise prescription (including rehabilitation and sports).^93 , 94 , 240^

Patients with VHD tend to gradually and imperceptibly reduce their level of physical activity (sedentary lifestyle), making exercise-induced symptoms difficult to notice. In these patients, ET plays a fundamental role in confirming truly asymptomatic status.^17 , 928^

In mild VHD, ET is generally well tolerated and safe. In some specific scenarios, however, the risk of complications and adverse events may be increased, thus requiring adoption of specific preventive measures (i.e. performance in hospital or a hospital-level facility, use of an attenuated protocol) or even contraindicating ET altogether.

Relative and absolute contraindications have been described in Part 1, Section 2.3 of this Guideline. ET in a hospital setting with cardiology support available is recommended in asymptomatic patients with severe valve involvement (stenosis or regurgitation), in patients with multiple-valve disease (moderate/severe), and in those with congenital heart disease.

Adaptations to ET methodology for patients with VHD:^13 , 112 , 293^

– The pre-test history and review of systems should focus on the etiology, severity, and course of VHD, current medications, presence of symptoms, and potential relative and absolute contraindications.

– Absolute contraindications to ET: clear indication for valve surgery, uncontrolled hypertension, complex arrhythmias, or systemic disease with exercise intolerance.^14 , 929^

– The physical examination, in addition to cardiac and pulmonary auscultation, should include a search for thrills and palpation of peripheral pulses.

– The ET protocol should consider the patient’s functional capacity and avoid large increments in work load (attenuated protocol).^930^

– In serial exercise test, the protocol last used should preferably be repeated, to allow comparison of symptoms, hemodynamic response, and functional capacity.

– The ET must be symptom-limited, and recovery should preferable be active.

– Close observation, characterization, and detailed description of any ET-emergent signs and symptoms is mandatory, with particular emphasis on fatigue, dyspnea, dizziness, chest pain, pallor, and diaphoresis, all of which are criteria for therapeutic intervention and prognostic markers.^931^

The following subsections will address particular aspects related to mitral and aortic valve disease, due to their widespread incidence and the availability of specific scientific evidence regarding ET in these settings.

#### 8.5.1. Aortic Stenosis

The main etiologies of aortic stenosis (AS) are rheumatic, degenerative (or atherosclerotic), and congenital heart disease. It is one of the most common forms of acquired valvular heart disease; ≈3% of the Brazilian population aged >75 has severe AS.^96 , 103^

The classic symptom triad is angina, syncope, and dyspnea. Immediate surgical intervention is mandatory after symptom onset, due to worsening prognosis and a median survival of up to 2 years if untreated (if not associated with HF). Sudden cardiac death is the most fearsome complication, but rarely occurs in truly asymptomatic patients.

ET can be performed safely in asymptomatic or mildly symptomatic patients with AS.^934^It is indicated to elucidate questionable symptoms, assess functional capacity, confirm absence of symptoms, and confirm decreased exercise tolerance. A normal ET indicates a very low probability of symptom onset and/or complications at 6 to 12 month follow-up.^97^

The incidence of abnormal TE depends on the severity of AS, ranging from 25% to 65%. Half of patients with asymptomatic severe AS will have an abnormal ET.^108 , 936 , 937^

Brazilian and international guidelines consider the following ET findings as indications for surgical aortic valve replacement:^92 - 94 , 109^

– Exercise-induced symptoms clearly attributable to aortic stenosis.

– Drop in blood pressure during exercise.

– Reduced exercise tolerance/functional capacity.

Particular aspects of ET in AS:

– The resting ECG in severe AS is usually (in 85% of cases) consistent with LVH, associated with secondary changes in ventricular repolarization. ST depression (>1 mm) is seen in approximately two-thirds of patients, even those with mild/moderate AS. Other changes include left atrial overload; left bundle branch block; and AF, especially in hypertensive older adults.^112 , 115^

– Approximately one-third of patients asymptomatic at baseline develop symptoms on exertion, which indicates a high probability of developing symptoms at rest and/or complications within 12 months of ET.^935 , 938^

– Occurrence of dyspnea at high work loads with rapid improvement during recovery, as well as dyspnea in patients with comorbid COPD (especially older adults), may be nonspecific findings.^112^

– Vertigo, presyncope, and syncope increase the PPV for development of symptoms at rest at 1-year follow-up.^937^

– In patients with AS, ET has no utility for detection of CAD (≈20% have asymptomatic obstructive CAD without ST depression). Half of patients exhibit exercise-induced ST-segment depression (horizontal or downsloping, >2 mm), which a high-risk marker but generally not associated with CAD.^934 , 938^

– It is common for patients to reach the submaximal HR predicted for age, but without an appropriate increase in cardiac output (usually only 50%).^939^

– Early increase in HR (85% of predicted HRmax or ≥50% increase in HR in the first 6 minutes) in patients with severe AS is associated with need for valve replacement (RR: 3.21; 95% CI: 1.70-6.08; p<0.001) and, in moderate AS, all-cause mortality (RR: 16.02; 95% CI: 1.83-140.02; p=0.012).^374 , 938^

– The BP response to ET is considered abnormal when there is a drop during exercise or an increase in SBP <20 mmHg; either is associated with greater incidence of symptoms.^240 , 930 , 938^

– A hypertensive response to exercise is associated with elevated SBP at rest, greater LV mass, and increased arterial stiffness. However, it is not associated with a higher incidence of symptoms or reduced functional capacity.^940 , 941^

– Exercise-induced complex ventricular arrhythmias mandate ET cessation, are considered a positive ET, and are a marker of poor prognosis. Ventricular arrhythmias during recovery have limited correlation with the severity and prognosis of AS.^939 , 942^

– Low functional capacity in asymptomatic severe AS is associated with increased mortality.^943^

#### 8.5.2. Aortic Regurgitation

Aortic regurgitation (AR) may result from a primary anomaly, such as a bicuspid aortic valve (in young patients), or from degeneration (in older adults). It may also occur secondary to rheumatic heart disease (the leading cause) or aortic root dilatation (in chronic hypertension, Marfan syndrome, etc.). In the Framingham Heart Study, the overall prevalence was 4.9%, with 0.5% of participants having moderate or severe AR.^104 , 944^

Most patients remain asymptomatic for decades. In asymptomatic patients with normal LV systolic function, mortality is <0.2% per year. With depressed systolic function, most develop symptoms (mean rate >25% per year) and require surgical intervention within 2 to 3 years. Dyspnea, angina, or HF are markers of poor prognosis: mortality is >10% per year in patients with angina and >20% in those with HF. Other predictors of adverse outcomes are age, LV end-systolic volume, and cardiorespiratory fitness. Onset of symptoms, even if mild, is sufficient to indicate surgical intervention, regardless of LV function.^102 , 105 , 945^

ET is performed to elucidate questionable symptoms. Rapid progression of ventricular enlargement or decline in functional capacity over the course of serial ETs is also a reason to consider surgical intervention.^97 , 946^

Particular aspects of ET in AR:

– Resting ECG may be normal early in the disease or show evidence of LV hypertrophy. Initially, with LV volume overload, prominent Q waves develop in leads I, aVL, and V3 to V6. As AR progresses, there is a decrease in Q waves and a progressive increase in the overall amplitude of the QRS complexes.^112 , 947^

– Exercise-induced ST-segment depression (>1.0 mm) is common, and generally is not associated with obstructive CAD.^948^

– Exercise-induced ventricular arrhythmias are also relatively common and correlate significantly with the degree of LV hypertrophy and dysfunction.^949^

– Dyspnea at low work loads in patients with normal systolic function is exceedingly rare. In patients with exercise-induced symptoms, other signs of LV dysfunction should be sought carefully.^950^

– Asymptomatic individuals with moderate to severe AR can engage in high-intensity physical exercise, and even those with severe AR can perform moderate-intensity exercise, provided that the LV and aorta are not enlarged and EF is >50%.^951 , 952^

#### 8.5.3. Mitral Stenosis

The most common cause of mitral stenosis (MS) worldwide is rheumatic fever. Isolated MS is twice as common in women as in men. Other causes of MS are rare and include congenital anomalies, radiation exposure to the chest, mucopolysaccharidosis, left atrial myxoma, and mitral annular calcification secondary to aging. Patients often present with exercise intolerance and right HF due to the development of post-capillary pulmonary hypertension.^92 , 93 , 240 , 953^

ET is useful for elucidating questionable symptoms or manifestations discordant with the severity of MS; it discloses symptoms in up to 46% of patients with moderate-to-severe MS previously considered asymptomatic. It also allows assessment of fatigue and cardiorespiratory fitness in significant MS (valve area ≤1.5 cm^2^).^931 , 954^

Particular aspects of ET in MS:

– Must be performed with patient on all current medications (including digoxin and beta-blockers).

– Resting ECG usually shows left atrial enlargement, atrial extrasystoles, or atrial fibrillation (intermittent or persistent). In MS with severe and/or long-term PAH, right ventricular hypertrophy, right axis deviation, and RBBB may be found.^112^

– An exaggerated HR response to exercise is common, due to the reduction in stroke volume (especially if AF is present). Exercise-induced ST-segment depression may occur, usually without any association with obstructive CAD.^955^

– Exercise-induced ventricular arrhythmias have been demonstrated to occur in 60% of patients with MS (complex arrhythmias in 20%). Incidence and complexity were not associated with stenosis severity.^956^

– Dyspnea is the most frequent symptom and has prognostic value. Exercise tolerance correlates well with lesion severity. In severe MS, the increase in pulmonary pressure with exertion can lead to pulmonary congestion.^106 , 957^

– In these patients, exercise-induced chest pain is usually associated with inadequate SBP elevation (blunted inotropic response) and is unrelated to CAD.^106 , 240^

#### 8.5.4. Mitral Regurgitation

Primary mitral regurgitation (MR) may occur as the result of a structural abnormality of the mitral valve (as in rheumatic heart disease), valve degeneration (i.e. in myxomatous disease and fibroelastic deficiency), or after endocarditis. Secondary (functional) MR is usually due to dilated cardiomyopathy, ischemic heart disease, or MI. It affects ≈24 million people worldwide, with wide variation across countries.^958 , 959^

In MR, ET is useful for assessment of symptoms and cardiorespiratory fitness (especially if symptoms are questionable) and, when symptom-limited, helps indicate surgical intervention. Exercise-induced symptoms are associated with disease severity. In asymptomatic patients with preserved cardiorespiratory fitness, valve repair can be postponed safely.^98 - 100^

Severe MR leads to high left atrial pressures, secondary PAH, and eccentric LV hypertrophy with systolic dysfunction. LV dysfunction usually precedes moderate-to-severe MR, with exercise-induced dyspnea and exercise intolerance.^960 , 961^

MR secondary to papillary muscle ischemia is usually associated with circumflex or right coronary artery disease. In ischemic or dilated cardiomyopathy, LV remodeling and mitral valve deformation may lead to MR, which carries a worse prognosis in this setting.^962 , 963^

Even patients with mild symptoms can decompensate quickly. Onset of symptoms indicates that LV compensatory mechanisms are overloaded.^963 , 964^

Particular aspects of ET in MR:

– Resting ECG in mild MR usually shows T wave flattening or inversion in the inferior leads. In moderate/severe MR, left atrial overload is usually observed. LV hypertrophy occurs in ≈⅓ of patients and right ventricular hypertrophy in ≈15%; AF is common.^965^

– Exercise-induced repolarization changes preclude use of ET for investigation of CAD, especially if LV hypertrophy and ST-segment changes in the baseline ECG are present. Even with a normal baseline ECG, exercise-induced ST-segment depression usually occurs; this is not associated with CAD nor related to clinical/functional deterioration.^966^

– Frequent, complex ventricular arrhythmias (couplets, non-sustained VT) usually occurs during exercise and recovery.^967^

– Reaching HRmax and/or experiencing a ≥29 bpm drop in HR in the first minute of recovery are associated with a low risk of cardiac events.^966^

– A depressed SBP response to exercise is common. In more severe cases, BP may drop during exercise due to a reduction in cardiac output; this mandates immediate test cessation.^931^

– Cardiorespiratory fitness is reduced in ≈20% of asymptomatic patients with severe MR, and portends a worse prognosis.^962 , 968^

#### 8.5.5. Mitral Valve Prolapse

Mitral valve prolapse (MVP) is a valvular heart disease with genetic predisposition resulting in myxomatous changes in the mitral valve leaflets. Its prevalence is around 2% to 3%, and the natural history is benign in most cases.^969 , 970^

MVP can be primary (“nonsyndromic”) or secondary (“syndromic”) to connective tissue disorders (Marfan syndrome, Loeys-Dietz syndrome, Ehlers-Danlos syndrome, osteogenesis imperfecta, pseudoxanthoma elasticum, and osteoarthritis). It can also be seen in hypertrophic cardiomyopathy.^971^

MVP can lead to progressive mitral regurgitation due to loss of leaflet apposition, and is accordingly the main cause of surgical mitral valve replacement due to mitral regurgitation in developed countries. Chronic mitral regurgitation is often associated with pulmonary hypertension with subsequent right HF, increased risk of arrhythmias (including AF), thromboembolic events, and infective endocarditis.^95 , 969 , 971^

Young women with mitral leaflet thickening and/or prolapse may have an increased predisposition to complex arrhythmias and arrhythmogenic sudden cardiac death (arrhythmogenic MVP), which is the most feared complication. The baseline ECG usually shows negative T waves in the inferior wall and a RBBB pattern.^970 , 972^

MVP is a relatively common finding in athletes, in whom it follows a generally benign course. The presence of moderate/severe mitral regurgitation and ventricular arrhythmia are markers of athletes at higher risk.^973 , 974^

Patients with idiopathic MVP often complain of palpitations or exertional tachycardia. They may also present with dyspnea, fatigue, reduced exercise tolerance, presyncope, syncope, and sequelae of stroke.

Risk markers for sudden death in MVP are: presyncope/syncope; Barlow’s disease (myxomatous degeneration of collagen tissues); family history of sudden death; moderate/severe mitral regurgitation; and ventricular arrhythmias.

ET is useful in MVP for assessment of symptoms, determination of exercise tolerance, detection of exertional arrhythmias, and medical clearance for physical activity/exercise prescription.

Particular aspects of ET in MVP:

– Baseline cardiac auscultation may be normal or reveal s mid-systolic click, late systolic or mid-systolic murmur (due to mitral regurgitation). Systolic murmur may be present only when the patient is standing.^975^

– Baseline ECG is normal in most patients. Nonspecific T wave and ST-segment changes may occur, especially in the inferior leads. In patients with chronic mitral regurgitation, patterns consistent with LA and LV hypertrophy may be observed.^976^

– Major abnormalities seen during exertion:

1) Reduced exercise tolerance, usually with a pattern analogous to neurocirculatory asthenia (characterized by palpitations, weakness, shortness of breath, labored breathing, and other subjective complaints, even with light physical exertion).^977^

2) Exercise-induced ST-segment depression that strongly mimics the ischemic pattern of obstructive CAD, usually in patients with associated MR. In MVP, exercise-induced ST-segment depression has low sensitivity and specificity for CAD (false-positive ET).^978 , 979^

3) Exercise-induced ventricular arrhythmias, with right bundle branch block pattern or complex arrhythmias (polymorphic PVCs, ventricular couplets, and/or non-sustained VT) are risk markers for suspected arrhythmogenic MVP.^974 , 980^

## 8.6. ET after Myocardial Revascularization

ET in the follow-up of patients who have undergone myocardial revascularization, whether through coronary artery bypass grafting (CABG) surgery or percutaneous coronary intervention (PCI), allows:^6 , 13 , 27 , 134^

– Elucidation of symptoms.^45 , 981^

– Determination of cardiorespiratory fitness/exercise tolerance.

– Assessment of response to pharmacotherapy.

– Risk stratification/prognosis.^982^

– Assessment of arrhythmia density and complexity.^983^

– Medical clearance for return to work and disability/work capacity assessment.

– Medical clearance for exercise prescription (including CV rehabilitation).^17 , 29^

– Assessment of restenosis (post-PCI), graft stenosis/occlusion (post-CABG), and/or progression of CAD. In these cases, ET is not indicated for periodic or routine evaluation of asymptomatic patients unless other specific clinical indications are present.^984 - 986^

Important points to be considered prior to performing ET in this setting:^6 , 13 , 293^

– Whether symptoms persist despite complete revascularization.

– Whether revascularization was partial or complete.

– Whether ventricular dysfunction/HF persisted and/or developed after revascularization, particularly after MI.^987^

– Need to withhold or discontinue medication.

– Patient’s level of physical activity before and after revascularization.

– Serial comparative analysis of hemodynamic and ECG findings in pre-CABG or pre-PCI ET/CPET, if available.

### 8.6.1. ET after Percutaneous Coronary Intervention

Depending on the time elapsed since PCI, ET has several indications with recognized value in follow-up of clinical outcome and CAD progression:^6 , 13 , 988^

– At 1-3 months post-PCI: investigation of new symptoms; assessment of residual ischemic load after incomplete PCI; optimization of drug therapy; medical clearance/exercise prescription (including cardiac rehabilitation); risk stratification; and disability/worker’s compensation and/or work capacity assessment.^982 , 989 , 990^

– At 3-6 months post-PCI: investigation of ischemic manifestations due to possible restenosis/reocclusion; evaluation of patients who have undergone PCI for myocardial bridging; optimization of therapy.^50 , 991 , 992^

– At 6-24 months post-PCI: assessment of symptoms; in patients at high risk of further CV events and progression of CAD, for optimization of therapy (including nonpharmacologic interventions).^50 , 984 , 993^

– >24 months post-PCI: follow-up of CAD progression and restenosis, which should be performed serially in high-risk patients.^994^

Particular aspects of ET after PCI:

– ET should always be symptom-limited, not least because it plays a fundamental role in managing patients during cardiac rehabilitation program (≥2 weeks after hospital discharge).^29^

– Absence of angina does not always mean complete revascularization. Exertional angina suggests residual ischemia and/or restenosis.^50 , 995^

– DP <25,000 bpm.mmHg suggests poor outcome or left ventricular dysfunction. Comparison with findings on pre-PCI ET, if available, is essential. Factors such as intraprocedural MI, progression of CAD, development of collateral circulation, hypertension, and current medications can all interfere with DP.^996^

– ST-segment normalization after PCI is the main indicator of successful revascularization. In the early post-PCI stage (1 to 6 months), the recurrence of ST-segment changes observed in pre-PCI ET does not mean restenosis. However, persistence of pre-PCI changes combined with a decrease in DP (or reversal of ET to ischemic findings after normalization) is suggestive of poor prognosis. At >6 months post-PCI, if CAD is suspected, a positive ET may reflect partial or complete stent occlusion, presence of residual lesions, progression of atherosclerosis or development of new atherosclerotic lesions, and even areas of myocardium showing post-acute ischemic adaptation.^997^

– At >2 years post-PCI, interpretation of exercise-induced ST-segment depression may still be limited if there is multivessel CAD, incomplete revascularization, history of MI, or pre-existing ECG changes (LVH and LBBB). On ET of patients with single-vessel and multivessel disease, a sensitivity of 46% and specificity of 77% (for >50% occlusion) and sensitivity of 50% and specificity of 84% (for >70% occlusion) were observed.^998 , 999^

– Prolongation of the QRS complex at peak exertion has shown to be a good predictor of coronary events (at 23±9 months).^1000^

– Most patients increase their exercise tolerance time (ETT) after PCI, which does not always translate to complete or sufficient revascularization. Reduction of ETT over serial exercise tests is usually associated with restenosis and/or CAD progression.^996 , 1001^

### 8.6.2. ET after Coronary Artery Bypass Grafting

ET after CABG allows assessment of symptoms and cardiorespiratory fitness, optimization of therapy, and medical clearance/prescription of physical activities (including CV rehabilitation).^27 , 29^

There are two stages at which ET may be indicated after CABG: early (<6 months), to assess the immediate results of revascularization; and late (>6 months), to assess the progression of response to treatment. In the first 5 years after CABG, ET is not indicated for serial evaluation of asymptomatic patients unless another specific indication exists.^1002 , 1003^

Early ET after CABG should not be performed before approximately 3 months. The aim is to:^115 , 1004^

– Ensure that sternal fixation – a common source of chest pain/discomfort and difficulty breathing, and, thus, a confounding factor – is stable.

– Cardiocirculatory and microcirculatory adaptations such as correction of tachycardia, blood volume, hematocrit, excess catecholamines, and coronary reserve are well established.

– Earlier ET is safe, but has limited clinical applicability due to the risk of false-positive results.

In ET performed after complete, successful CABG:^1005^

– Reduction and/or even normalization of exercise-induced ST-segment depression may occur.

– In up to 30% of patients, exercise-induced ST-segment depression may occur without graft obstruction/occlusion and/or new coronary lesion.

– Occluded grafts may not cause exercise-induced ST-segment depression consistent with ischemia.

– In the early post-CABG phase (<6 months), exercise-induced ST-segment depression suggests graft thrombosis. In the chronic post-CABG phase, it is associated with atherosclerosis and/or progression of CAD.^1006^

In graft occlusions, the following may occur:

– Decreased exercise tolerance time, even without associated ECG changes.^1007^

– Increased DP due to elevated HRmax.

– DP <25,000 bpm.mmHg after CABG is suggestive of graft obstruction and/or left ventricular dysfunction.

Particular aspects of post-CABG ET:

– The improvement of anginal symptoms after CABG decreases over time, due to loss of graft patency and/or CAD progression. Five years after CABG with complete revascularization, angina has returned in ≈30% of patients.^1008^

– In a study of serial ET follow-up of 435 post-CABG with complete revascularization, there was significant improvement in angina, ETT, and exercise-induced ST-segment depression in the first year of follow-up, with improvements persisting into the sixth year.^1009^

– ETT and maximal DP were highest in the first 3 years of follow-up.^1009 , 1010^

## Part 3 – Cardiopulmonary Exercise Test

### 1. Introduction

Cardiopulmonary exercise test (CPET) is a diagnostic method that allows simultaneous analysis of clinical, hemodynamic, electrocardiographic, and ventilatory parameters and gas concentrations in expired air. The indications for CPET are given in Part 1, Section 2.4.^17 , 219 , 229 , 293 , 1011^

CPET incorporates real-time measurement of the volume of expired air (VE) by air flow and volume sensors (pneumotachograph, Pitot tube, turbine flowmeter) and the fractions of expired oxygen (FEO_2_) and carbon dioxide (FECO_2_) by a gas analyzer, allowing collection of multiple variables of clinical interest (Figure 18). CPET has diagnostic and prognostic value, excellent cost-effectiveness, and is relevant both in clinical practice and in research settings.^17 , 29 , 219 , 737 , 1012 , 1013^

## 2. Exercise Physiology Applied to CPET

All physical activity consumes energy. This requires uninterrupted resynthesis of adenosine triphosphate (ATP), which in skeletal muscle occurs through three metabolic pathways:

– Anaerobic alactic or ATP-creatine phosphate (CP) system: provides energy immediately, but for a very short time. During extremely intense activity, cellular stores of ATP-CP are depleted within seconds, which is the limiting factor.

– Lactic anaerobic system or anaerobic glycolysis: provides short-term energy, for a short time. In high-intensity activities lasting few minutes, it generates incremental amounts of waste lactic acid, which leads to limiting metabolic acidosis.

– Aerobic or oxidative system: requires O_2_consumption (VO_2_). Provides an “unlimited” long-term source of energy, allowing sustained mild to moderate physical exertion at the expense of macronutrient metabolism.^229 , 293 , 1011^

These three metabolic pathways function in an integrated, interdependent manner, and the recovery of anaerobic pathways is dependent on aerobic metabolism (Figure 19).

When performing CPET, one must take into account that, during the transition from rest to exertion, all three metabolic pathways (aerobic, alactic anaerobic, and lactic anaerobic) participate concomitantly. Only after the mitochondrial activation period does the aerobic pathway become predominant.

From the first ventilatory threshold (VT1), also known as the anaerobic or lactate threshold (Figure 20), the lactic anaerobic pathway becomes increasingly active (moderate- to high-intensity exercise), with metabolic acidosis compensated by respiratory alkalosis. From the second ventilatory threshold (VT2) onward, also known as the respiratory compensation point (RCP), incrementally limiting metabolic acidosis occurs due to the impossibility of ventilatory/respiratory compensation, culminating in physical exhaustion.^219 , 229 , 293 , 1011^

The anaerobic threshold (AT) has prognostic value in patients with HF, and is relevant for performance assessment and exercise prescription in cardiovascular rehabilitation and sports.^1014^As exercise tolerance increases with adaptation to training, the AT shifts to the right, approaching the VO_2_max **.**

## 3. Pulmonary Ventilation, Expired Gas Analysis, and Derived Variables

### 3.1. Pulmonary Ventilation

#### 3.1.1. Baseline Spirometry

Baseline spirometry measures the volume and flows derived from maximal inspiratory and expiratory maneuvers.

The minimal parameters for the appropriate interpretation of lung function tests when performed to assess ventilatory disorders (obstructive, restrictive, and mixed) are: vital capacity (VC); forced expiratory volume in one second (FEV1); FEV1/FVC ratio; total lung capacity (TLC); and residual volume (RV). Normal values of these parameters for white and black Brazilian populations are shown in Table 42.^1015 - 1017^

The variables most widely used in clinical practice are:

– Vital capacity (VC): corresponds to the largest volume of air mobilized during maximal expiration, which can be obtained through forced maneuvers (forced vital capacity, FVC) or slow maneuvers (slow vital capacity, SVC; far less common). The FVC corresponds to the volume obtained in a single maximum inspiration followed by maximal forced expiration. During exertion, both in sedentary and active individuals, despite large increases in VE, VT only exceptionally exceeds 60% of FVC. FVC varies with age, BMI, and sex (4-5 L in men and 3-4 L in women).

– FEV1: volume of air exhaled in the first second during an FVC maneuver. It is one of the most clinically useful variables, aiding in the diagnosis of obstructive ventilatory disorders.

– Forced mid-expiratory flow (FEF25-75): represents the mean forced expiratory flow obtained during an FVC maneuver, at 25% to 75% of FVC.

– Peak expiratory flow (PEF) rate: corresponds to maximal air flow during an FVC maneuver.

– Flow-volume loop: graphic representation of the flow and volume generated during an FVC maneuver. This plays an important role in the diagnosis of certain respiratory conditions, as it allows visual identification of obstructive and restrictive patterns, cutoff of inspiratory or expiratory flows, and assessment of responses to bronchodilator administration.

#### 3.1.2. Ergospirometry

Minute ventilation (VE), measured in L/min, corresponds to the total volume of air that ventilates the respiratory tract, including dead space (VD). It is calculated as:


VE=VT×FR

VE: minute ventilation (L/min)

FR: respiratory frequency (breaths/min)

VT: tidal volume (L/min)

#### 3.1.3. Ventilatory Reserve

In healthy young adults at rest, the FR is usually ≈12 breaths/min and the VT ≈0.5 L/min, resulting in a VE = 6 L/min (0.5 L/min × 12 = 6 L/min). During strenuous physical exertion, the LV usually increases 17 to 20-fold, and the FR to 35-45 breaths/min. Exceptionally, the FR may exceed 50 breaths/min for a VT >2 L/min (Figure 21).

Ventilatory reserve (VR) reflects the relationship between maximal exercise ventilation (VEmax) and maximal voluntary ventilation (MVV) at rest (both in L/min; VR = [MVV–VEmax/MVV]x100). Its normal range is 20% to 40%, which corresponds on average to 3,000 mL in men and 2,100 mL in women. MVV is assessed by breathing in an out as deeply and quickly as possible for 15 seconds; the result is extrapolated to what would be obtained if the maneuver persisted for 1 minute. The normal range is 35 to 40 times the FEV1. VR measurement contributes to the differential diagnosis of dyspnea.^1011 , 1017 , 1018^

Prediction equations:


MVV=FEV1×(normal range is35to40)

$$VR=\frac{\text{MVV-VEmax}}{\text{MMV}}\times 100$$

MMV: maximal voluntary ventilation

FEV1: forced expiratory volume in one second

VR: ventilatory reserve

VEmax: maximal exercise ventilation

The increase in V_T_during exertion occurs due to the additional use of inspiratory (VRInsp) and expiratory (VRExp) reserve volumes, which can reach, respectively, 75% to 85% of TLC and 40% of TLC. These volumes are determinants of VEmax, which, under normal conditions, is <70% of MVV (resulting in a VR between 20% and 40%). It bears stressing that during strenuous exercise, particularly by athletes, a higher percentage of VR can be mobilized.^219 , 229 , 293 , 1011^

## 3.2. Oxygen Consumption

Oxygen consumption (VO_2_) corresponds to the difference between the O_2_concentration in inspired air and the concentration in expired air. It reflects the amount of O_2_consumed in metabolic activity (Figure 22). VO_2_can be expressed in absolute form (L/min) or, preferably, relative to body weight most often expressed in mL/kg/min (mL.kg^- 1^.min^- 1^is also acceptable). These values can be converted into metabolic equivalents of task (1 MET = 3.5 mL/kg/min VO_2_).

The highest VO_2_value obtained in CPET can be defined as VO_2_max or VO_2_peak:

– VO_2_max when, despite an increase in the exercise load, a VO_2_plateau is reached, with no further increase or only a slight increase (<50 mL/min or 2.1 mL/kg/min) (Figure 22).

– VO_2_peak when the highest value achieved at the end of an exhaustive effort occurs in the absence of a plateau in VO_2_(Figure 23).^219 , 229 , 293 , 1011^

VO_2_max is considered the gold standard for determination of cardiorespiratory fitness (CRF).^1012^

3.3. Carbon Dioxide Production

Carbon dioxide production (VCO_2_), which in CPET is derived from FECO_2_, is usually expressed in L/min, and is rarely used alone. However, the variables derived from its measurement, such as the respiratory quotient (RQ) and the ventilatory equivalent of CO_2_(VE/VCO_2_), have great clinical utility.^219 , 229 , 293 , 1011^

3.4. Ventilatory Thresholds

The ventilatory thresholds are metabolic transition points observed on VE, VO_2_, VCO_2_curves and their derived variables. VTs can be expressed indirectly, in relation to metabolism (i.e. VO_2_in mL/min or L/min or in % of VO_2_max) or to cardiovascular demand (i.e. VO_2_in mL/bpm) – see Figures 20 and 23.^219 , 229 , 293 , 1011^

The identification of ventilatory thresholds in patients with CVD (such as HF and CAD) has prognostic value, contributes to optimization of pharmacologic therapy, and optimizes the exercise prescription both for cardiovascular rehabilitation and for physical training of apparently healthy individuals, particularly competitive athletes engaged in predominantly aerobic activities.^17 , 219 , 229 , 293 , 1011 , 1014 , 1016^

### 3.4.1. First Ventilatory Threshold

The first ventilatory threshold (VT1), also known as the anaerobic threshold (AT) or lactate threshold (LT), reflects the acceleration of the rate of sustained lactate buildup in the bloodstream, identifying the transition to a stage of increasing and incremental participation of anaerobic lactic metabolism, but at which metabolic acidosis is still compensated by respiratory alkalosis (until the second ventilatory threshold, VT2, is reached). Its direct measurement is performed by measuring blood lactate (in mmol/L or mEq/L), while indirect measurement is based on CPET parameters; VT1 can be observed at the point when the VE/VO_2_(Figure 24A) and VCO_2_/VO_2_curves begin to dissociate (Figure 24B).^219 , 229 , 293 , 1011^

### 3.4.2. Second Ventilatory Threshold

The second ventilatory threshold (VT2), also known as the respiratory compensation point (RCP) or onset of blood lactate accumulation (OBLA), is the point at which decompensation of metabolic acidosis occurs. It is represented by a second inflection in the VE and CO_2_curves in relation to the VO_2_curve. It corresponds to the transition from very high intensity exercise to maximal exercise, with severe, incremental decompensation of metabolic acidosis, rapidly progressing to physical exhaustion. From VT2 onward, there is also a disproportionate increase in VE in relation to VCO_2_, i.e. dissociation of the VE and VCO_2_curves, with an increase in the VE/VCO_2_slope. At this point, in patients with HF, a VE/VCO_2_slope ≥34 is indicative of poor prognosis (Figure 25).^219 , 229 , 293 , 1011^

## 3.5. Respiratory Quotient

The respiratory quotient (RQ) or respiratory exchange ratio (RER) is the ratio between VCO_2_and VO_2_. It allows identification of exercise intensity and of which macronutrient is being consumed to generate energy:

– During initial CPET loads, it is approximately 0.72 (almost exclusively fat).

– At VT1, it is usually ≈0.82 (≈40% fat, ≈60% carbohydrate).

– At VT2, it is approximately 1.00 (almost exclusively carbohydrates).

At higher intensities and close to peak exertion, the VCO_2_surpasses the VO_2_, making the RQ incrementally >1.00. A RQ ≥1.10 is considered a sign of near exhaustion or exhaustion, allowing the test to be considered maximal.^219 , 229 , 293 , 1011 , 1012^

## 3.6. Ventilatory Equivalents of Oxygen and Carbon Dioxide

The ventilatory equivalents of O_2_(VE/VO_2_) and of CO_2_(VE/VCO_2_) indicate, respectively, the VE required to consume 1 L/min of O_2_and produce/dispose of 1 L/min of CO_2_. During progressive exertion, the VE/VO_2_ratio decreases up to VT1, at which point it progressively increases, with positive inflections at VT1 and VT2. The VE/VCO_2_slope decreases up to VT2, then increases thereafter.

These parameters can be used to assess cardiorespiratory efficiency, help identify thresholds, and contribute to diagnosis, prognosis, optimization of therapy, and exercise prescription in various clinical scenarios, especially ischemic heart disease and HF (Figure 26).^219 , 229 , 293 , 1011^

The VE/VCO_2_curve is considered a predictor of mortality and/or hospitalization, especially in patients with HF and COPD. The higher the ratio, the worse the prognosis (see Figure 25).

## 3.7. End-tidal Oxygen and Carbon Dioxide Pressures

The end-tidal partial pressure of oxygen (PETO_2_) is obtained from the measurement of FEO_2_in expired air by the gas analyzer. It is usually measured in mmHg (based on Dalton’s law, which states that the total pressure of a gaseous mixture is equal to the sum of the partial pressures of its component gases).

At rest and at sea level, normal PETO_2_is ≈100 mmHg. It decreases transiently at the start of exercise (due to a disproportionate increase in VO_2_in relation to VE), and then increases 10 to 30 mmHg until maximum exertion (Figure 27).

PETO_2_, both at rest and during exertion, has been shown to correlate with 4 CPET parameters that are usually abnormal in patients with LV dysfunction: VO_2_peak; anaerobic threshold (VT1); delta VO_2_to delta work rate (ΔVO_2_/ΔWR) ratio; and increased slope of the VE/VCO_2_curve (a marker of ventilation/perfusion mismatch).

PETO_2_is higher in patients with impaired cardiopulmonary function, with VT1 being the metabolic state that presents the best correlation.^1019^

The end-expiratory partial pressure of carbon dioxide (PETCO_2_) is derived from measurement of FECO_2_, and is usually reported in mmHg. It reflects the alveolar and arterial partial pressure of carbon dioxide (PaCO_2_). Normally, at rest and at sea level, the alveolar CO_2_pressure (PCO_2_) is ≈40 mmHg, rising 3-8 mmHg on exertion. It reaches its peak at VT2 and then decreases until maximum exertion. Like PETO_2_, PETCO_2_correlates with VO_2_peak, VT1, ∆VO_2_/∆WR, and increased slope of the VE/VCO_2_curve (see Figure 27).^1019^

PETCO_2_measured at VT1 (the time point of greatest metabolic stability) correlates with cardiac output (CO) and, in patients with chronic HF, reflects disease severity. Its measurement may be compromised by acute hyperventilation, increased dead space (due to emphysema and other lung diseases), and in rapid shallow breathing.^1019 , 1020^

In exercise-induced right-to-left shunting, there is an abrupt, sustained increase in PETCO_2_with a concomitant decrease in PETO_2_, coinciding with an increase in VCO_2_/VO_2_and oxygen desaturation.^1021 , 1022^

## 3.8. Oxygen Pulse

The oxygen pulse (OP or O_2_pulse) is obtained by dividing the VO_2_by the heart rate (VO_2_/HR) and is given in mL/kg/min/bpm. It reflects the amount of O_2_transported with each cardiac systole and is directly related to stroke volume, allowing assessment of LV function. In myocardial ischemia, LV dysfunction can be detected by a plateau (early flattening) or decreasing (downsloping) OP response (Figure 28 A and B).^219 , 229 , 1013 , 1023^

In the ORBITA study, among the CPET parameters tested, only OP plateau objectively detected the severity of myocardial ischemia as diagnosed on dobutamine stress echocardiography.^1013^

## 3.9. Delta VO 2 to Delta Work Rate (ΔVO 2 /ΔWR) Ratio

The ΔVO_2_/ΔWR ratio provides a physiological measure of the work rate, HR, and VO_2_at which myocardial ischemia develops, allowing its diagnosis, quantification, and, if possible, reversal with treatment. It is most easily determined on a cycle ergometer with a ramp protocol, and is expressed in mL/min/W.^219 , 229 , 293 , 1011 , 1024^

In healthy adults, the ratio is linear from the onset of exercise to peak exertion, and its value is ≈10 mL/min/W.^1023 , 1024^In exercise-induced ischemia, as LV dysfunction occurs, the ∆VO_2_/∆WR curve flattens from the ischemic threshold onward (Figure 29).^1023 , 1024^

## 3.10. Cardiorespiratory Optimal Point

The cardiorespiratory optimal point (COP) corresponds to the nadir of the VE/VO_2_curve (Figure 26). It is an easy-to-use submaximal variable. It reflects cardiorespiratory efficiency and has predictive value for cardiovascular and all-cause mortality, alone or combined with VO_2_max.^1025 , 1026^

## 3.11. Oxygen Uptake Efficiency Slope (OUES)

The oxygen uptake efficiency slope (OUES) is a highly reproducible variable, even when derived from submaximal VO_2_values. It reflects a nonlinear relationship of the ventilatory response to exertion, generated by a logarithmic regression between VO_2_and VE. In simple terms, the OUES can be said to represent the absolute increase in VO_2_corresponding to a 10-fold increase in VE.^1014 , 1027 , 1028^


$$\text{To obtain the OUES:}{VO}_{2}=a\log10VE+b$$

where a = reference value of OUES (calculated by the formulas presented in the text)

b = intercept on the curve

VE = minute ventilation

In 2012, Sun et al.^1028^presented a series of formulas to predict the OUES:

– For men: 1.178 – (age × 0.032) + (0.023 × height [cm]) + (0.008 × weight [kg])

– For women: 0.61 – (age × 0.032) + (0.023 × height [cm]) + (0.008 × weight [kg])

The steeper the adjusted VO_2_slope, the greater the OUES and, therefore, the greater the efficiency of O_2_uptake. Patients with severe HF have lower ventilatory efficiency, with low OUES values (Figure 30).^1014 , 1027 - 1029^

OUES has prognostic value as a marker of event risk in HF, even as a standalone variable.^1014^Other studies have also documented the prognostic value of OUES compared to other CPET variables.^1027 , 1029^

## 3.12. Exercise Oscillatory Ventilation

Exercise oscillatory ventilation (EOV) is an abnormal, reproducible phenomenon, easily recognizable on submaximal CPET. It is characterized by cyclic fluctuation of VE (anoscillatory ventilatory pattern >15% of mean resting VE) and expired gas kinetics that persists >60% of exercise duration.^229 , 1030 - 1034^

EOV is considered a marker of severity and worse prognosis in HF, especially when it occurs early and the cycle lasts >1 minute (Figure 31).^229 , 1026 , 1035^

## 3.13. Oxygen Uptake Recovery Delay

Oxygen uptake recovery delay (VO_2_RD) is an easily recognizable parameter which can contribute to prognosis, optimization of pharmacotherapy, and exercise prescription for cardiovascular rehabilitation and sports training. T½ corresponds to the time required for VO_2_to decrease 50% from VO_2_peak. It is shorter in well-trained individuals, while its increasea is associated with worse prognosis in patients with HF (Figure 32).^1011 , 1036 - 1038^

## 3.14. Circulatory Power and Ventilatory Power

Circulatory power (CP) is the product of peak systolic blood pressure (SBP peak) times VO_2_max or VO_2_peak. Ventilatory power (VP) is the product of SBPpeak divided by VE/VCO_2_. Both have prognostic value in HFrEF, regardless of circulatory power. Combined assessment of both improves risk stratification for major outcomes (death, ventricular assist device, heart transplant).^1039^

## 3.15. Reference Ranges for CPET Variables

Whenever possible, measurements obtained during CPET (Figure 33) should be presented relative to their respective reference (predicted) values or ranges; this is essential for proper interpretation and conclusions. Suggested reference values are given in Tables 43 and 44.

## 4. Equipment and Methods

### 4.1. Ergometers

The ergometers most commonly used in CPET are the treadmill and the bicycle or cycle ergometer.

In the general population, the treadmill has some advantages over the cycle ergometer, such as greater familiarity with the activity (walking vs. cycling), use of a larger muscle mass, and work against gravity, which causes greater stress on the cardiorespiratory system and, consequently, a higher VO_2_max/VO_2_peak (≈5-15% greater). One limitation of cycle ergometers is the difficulty or inaccuracy in determining the workload in watts.^1040^

Cycle ergometers can be mechanically or electromagnetically braked, with the latter being preferred as they allow automatic increments of workload with small changes in cycling cadence (40-70 rpm). Advantages of cycle ergometers over treadmills include a lower risk of falls and fewer artifacts, facilitating ECG recording and BP measurement.^1040^

When assessing high-performance athletes, one would ideally use the ergometer closest to their habitual sporting activity, i.e. a cycle ergometer for cyclists and a treadmill for runners. One should also consider the facility’s experience, the athlete’s familiarity, and whether specialty ergometers (i.e. row, tank, and ski ergometers) are available.^1011 , 1040^

### 4.2. Airflow or Volume Transducers

Only certified equipment that allows full assessment of the key parameters proposed in this Guideline and by scientific societies such as the Brazilian Societies of Cardiology and Pulmonology, the European Respiratory Society, and the American Thoracic Society should be used.

Airflow or volume transducers available:

– Pneumotachograph: flow transducer that measures the pressure differential of ventilated air through a low-resistance membrane.

– Pitot tube: flowmeter that measures the pressure differential between ports oriented in the direction of air flow.

– Turbine flowmeter: volume transducer in which an ultralight turbine coupled to an optoelectronic revolution counter is placed in the path of the ventilatory flow.

### 4.3. Gas Analyzers

Two main types of gas analyzers are available:

1) Mass spectrometer: considered the gold standard, is able to analyze all collected gases. Its high cost restricts widespread use, especially in commercial systems.

2) Single gas analyzers:

– CO_2_analyzer: based on the absorption of CO_2_molecules in the infrared range.

– O_2_analyzers: may be paramagnetic, which take advantage of the effect of O_2_molecules in a magnetic field, or electrochemical, in which reactions between O_2_and a suitable substrate at high temperatures are measured by a sensor.

### 4.4. Measurements of Gas Exchange

VO_2_and VCO_2_are defined as the difference between the inspired and expired volumes of these gases. VO_2_equation:


$${VO}_{2}=\frac{\left(VInsp\times {FIO}_{2}\right)-\left(VExp\times {FEO}_{2}\right)}{\text{Time}}$$

VInsp: inspired air volume, calculated on the premise that the expired and inspired fraction of nitrogen remains constant;

VExp: expired air volume; Time: time (in minutes) over which the measurement is performed; FIO_2_: concentration of O2 in inspired air; FEO_2_: concentration of O_2_in expired air.

Methods for measuring gas exchange:

– Mixing chamber: these systems contain a two-way breathing valve and chamber for continuous measurement of O_2_and CO_2_concentrations. They are more accurate when using fixed-load protocols. For incremental protocols, their accuracy is similar to that of breath-by-breath systems.

– Breath-by-breath: the most widely used method. O_2_and CO_2_concentrations are measured near the mouthpiece (50 to 100 samples/min). To avoid measurement errors, breath-by-breath systems require corrections for steam saturation, temperature, atmospheric pressure, and the delay between sampling and actual arrival of the gas at the analyzer.

### 4.5. Calibration, Quality Control, and Sanitization

Calibration and quality control procedures must follow the device manufacturer’s recommendations regarding method, frequency, and regularity.

– Volumetric and gas calibrations should be performed routinely.

– Volumetric calibration: using a 3-liter syringe (volume variations of up to 3% are considered acceptable).

– Gas calibration: performed with ambient air and a compressed mixture of CO_2_and O_2_. Breath-by-breath systems require additional calibration for sampling delay and gas arrival at the analyzer.^1041^

Sanitization must be a routine process. In addition to the methodological aspects of ET (see in Part 2, Section 1.1.3), it must provide for constant sanitizing of providers’ hands, surfaces, and CPET equipment, in line with institutional protocols and relevant recommendations from health authorities.^1042^

Calibration and quality control aim to ensure the quality and reproducibility of test results. However, multiple factors can interfere with this, including changes in clinical status, patient motivation, and patient adaptation curve.

### 4.6. Protocols

Validated protocols can be divided into two categories:

– Incremental: step or ramp (the latter is most recommended for clinical practice, whether with a cycle ergometer or treadmill).

– Fixed-load: used in athletes and in patients with known lung disease, for evaluation of flow-volume loops and dynamic hyperinflation.^1043^

The protocols and criteria for cessation of exercise are the same as for ET, described elsewhere in this Guideline.

### 4.7. Data Analysis Software

CPET software should:

– Integrate/correlate ET variables with ventilatory (ergospirometry) data and gas analyzer output, allowing determination of variables relevant to CPET.

– Allow numeric and graphic visualization of data.

– Allow clear demarcation of the anaerobic threshold, the respiratory compensation point, and maximal exertion.

– Allow flow-volume loops to be plotted at rest and during exercise.

– Present the results in an orderly and clear manner, including, whenever possible, the respective reference ranges or values.^1044^

### 4.8. Guidelines for Patients When Scheduling CPET

The same recommendations apply as for ET (listed in Part 2, Section 1.1.6 of this Guideline). However, a CPET-specific informed consent form is mandatory.

## 5. CPET in Selected Specific Situations

### 5.1. Heart Failure

A classic indication for CPET is the selection of patients with end-stage HF for heart transplantation (class IB evidence): when on beta-blockers, VO_2_peak <12.0 mL/kg/min; in those intolerant to beta-blockers, VO_2_peak <mL/kg/min.^1045 , 1046^

Determination of ventilatory thresholds allows optimal prescription of aerobic exercise, particularly in the rehabilitation of patients with HF.^17 , 29^

CPET variables useful for risk stratification and optimization of therapy in patients with HF (Table 45):

1) VO_2_max/peak is an excellent prognostic marker of mortality.^1045 - 1048^

2) Weber’s classification based on VO_2_max/peak and increasing mortality rates: A (VO_2_>20 mL/kg/min), B (16-20 mL/kg/min), C (10-15 mL/kg/min), or D (<10 mL/kg/min).^1049^

3) VE/VCO_2_slope >34 is an independent predictor of worse prognosis in HF.^1047 , 1048^

4) Arena et al. risk classification by VE/VCO_2_for events (mortality, transplantation, or left ventricular assist device implantation) by ventilatory class (VClass): VClass-I: ≤29; VClass-II: 30.0-35.9; VClass-III: 36.0-44.9; VClass-IV: ≥45.0. Event-free survival for subjects in VClass-I, II, III, and IV, respectively: 97.2%, 85.2%, 72.3%, and 44.2% (p<0.001).^1050^

5) CPET score: based on a combination of test variables with a score >15 associated with a high risk of events: VE/VCO_2_curve >34 = 7 points; HR drop <6 bpm in the 1st minute of recovery = 4 points; OUES <1.47 L/min = 3 points; VO_2_peak ≤14 mL/kg/min = 2 points; resting PETCO_2_<33 mmHg = 2 points.^1014 , 1021 , 1022 , 1030^

6) Other prognostic variables: VO_2_VT1, OUES, oxygen pulse, EOV, and COP.^1014 , 1019 , 1020 , 1025 , 1026 , 1032 , 1033 , 1051 - 1056^

### 5.2. Coronary Artery Disease

In stable coronary artery disease, CPET essentially enables:

– Diagnosis, discrimination of pathophysiological aspects, and determination of the severity of exercise-induced ischemia.^737 , 1012 , 1013 , 1023 - 1029 , 1035 , 1060 - 1068 , 1069^

– Determination of cardiorespiratory fitness (CRF) by direct measurement of VO_2_peak/VO_2_max (the gold standard).^737 , 1012 , 1069 , 1070^

– Contributes to the selection and possible optimization of therapeutic interventions.^1013 , 1062 , 1063 , 1065 , 1066 , 1068^

– Individualized, optimized prescription of aerobic exercise for CV rehabilitation.

CPET variables with diagnostic utility and prognostic significance in CAD:

– Oxygen pulse, VE/VCO_2_curve, PETCO_2_and PETO_2_, ∆VO_2_/∆WR all allow detection of exercise-induced myocardial ischemia, conferring greater sensitivity and specificity to CPET compared to ET.^1013 , 1023 , 1024 , 1027 - 1029 , 1035 , 1061 - 1068 , 1071^

– Prognostic variables: cardiorespiratory fitness (VO_2_max/VO_2_peak); VO_2_at anginal threshold; COP.^737 , 1012 , 1025 , 1026 , 1069 , 1070^

– Persistent or transient LV dysfunction may occur, with ventilation/perfusion (V/Q) mismatch due to reduced pulmonary blood flow with adequate ventilation. In LV dysfunction, PETCO_2_(at VT1), PETO_2_(at VT1 and at peak exertion), and their indices and ratios – VO_2_peak, VT1, ∆VO_2_/∆WR, and increased VE/VCO_2_slope – are abnormal.^1019 , 1020 , 1027 , 1029 , 1035 , 1060 , 1061^

### 5.3. Hypertrophic Cardiomyopathy

When performing CPET in patients with hypertrophic cardiomyopathy (HCM), bear in mind that (Table 46):

– VO_2_max or VO_2_peak (when RQ ≥1.10 is not reached) have prognostic value.^17 , 29 , 1012 , 1072 , 1073^

– CPET can identify patients with exercise-induced LV outflow tract obstruction, particularly by the oxygen pulse, allowing optimization of therapy and adjustment of exercise intensity for CV rehabilitation.^219 , 1021 , 1072 , 1073^

### 5.4. Valvular Heart Disease

In valvular heart disease, particularly in aortic stenosis (AS), CPET contributes to evaluation of clinical and functional impact and helps inform treatment decisions.^939 , 1078^

Specifically in AS:

– Prognosis worsens with the presence of stress-induced symptoms.

– CPET is relevant for improving diagnosis and prognosis and differential diagnosis of limited exercise capacity, especially in sedentary individuals, those with exercise intolerance, or patients with multiple comorbidities.

– Maximal CPET should be restricted to asymptomatic or questionably symptomatic patients; it is absolutely contraindicated in indisputably symptomatic patients.^1079 , 1080^

### 5.5. Lung Diseases

#### 5.5.1. Chronic Obstructive Pulmonary Disease

The severity of chronic obstructive pulmonary disease (COPD) can be established by symptoms and lung function test (spirometry) data. However, resting spirometry does not allow determination of the severity of exercise intolerance.^1081^

An inability to increase ventilation sufficiently to allow adequate levels of gas exchange (ventilatory limitation or constraint) is characteristic of obstructive pulmonary conditions, but it can also occur in restrictive diseases (interstitial and chest-wall diseases). A ventilatory reserve (VR) <15% at peak exertion, especially when the RQ <1.00, establishes the diagnosis of ventilatory limitation.^337 , 1081 - 1083^

In COPD, VO_2_peak is the best marker of cardiorespiratory fitness (CRF), i.e. of when the patient has exercised to their limit. The possibility low ACR associated with high ventilatory demand and severe lower-limb fatigue should also be considered.^1081^

Dynamic hyperinflation is one of the factors that can cause intolerable dyspnea during exercise. As respiratory flow increases during exercise, air trapping may occur, with a progressive increase in residual volume and, consequently, a reduction in inspiratory capacity (IC). This often occurs in conjunction with a reduction in tidal volume (V_T_), indicating that ventilatory mechanics have reached their limits. In CPET, dynamic hyperinflation can be better observed when periodic analyses of the flow-volume curve are performed alongside IC measurement during exercise, especially when there is a discrepancy between symptom severity and the degree of airway obstruction.^1081 , 1082^

Dynamic hyperinflation, one of the ventilatory mechanisms that can cause intolerable dyspnea on exertion, results from increased respiratory flow and air trapping with progressive increase in VR and reduction in V_T_, with a consequent reduction in IC. On CPET, dynamic hyperinflation can be observed through periodic analyses of the flow-volume curve alongside IC measurement, showing a discrepancy between symptom intensity and the degree of airway obstruction.^1081 , 1082^

#### 5.5.2. Pulmonary Vascular Disease

Pulmonary arterial hypertension (PAH), defined as a mean pulmonary arterial pressure (mPAP) ≥25 mmHg, has dyspnea on exertion as its earliest symptom.^1084^Resting mPAP is often normal in the early stages of pulmonary vascular disease (PVD), becoming abnormal when more than 50% of the pulmonary circulation is obstructed, leading to delayed diagnosis of PAH.^246 , 1085 - 1092^Identification of PAH by CPET can be performed using an invasive method (the gold standard) or a noninvasive method (Table 47).

The invasive method, available only in a select few institutions worldwide, requires placement of a pulmonary artery catheter for direct measurement of mPAP. Its reference value is determined arbitrarily as >30 mmHg. This method is not recommended for routine use in the early detection of PVD.^1092 - 1094^

The noninvasive method is useful in the evaluation of patients with dyspnea of unclear etiology and/or suspected PVD, considering that:

– The VE/VCO_2_slope at VT1 and at peak exertion are very high in PAH.^1092^

– Low PETCO_2_values at rest and during exercise, with an increase in PETO_2_, have been associated with PAH due to V/Q ratio mismatch (reduced pulmonary blood flow despite adequate ventilation).

– VE/VCO_2_>37 and PETCO_2_<30 mmHg at COP may indicate PVD, in the absence of acute hyperventilation.

– Exceptionally low PETCO_2_values (<20 mmHg) suggest PAH as a cause of exertional dyspnea.^1091^

When CPET is used to assess severity and response to therapy in established PAH:

– In idiopathic PAH, VO_2_peak <10.4 mL/kg/min and SBP peak <120 mmHg portend a worse prognosis (Table 48).^246^

– VO_2_>15.0 mL/kg/min indicates a good prognosis.^1086 , 1093^

– VE/VCO_2_≥54 at VT1 and ≥62 at maximum exertion indicate worse prognosis/shorter survival.^1089^

– A flatter VE/VCO_2_curve (higher values) is observed in PAH caused by chronic pulmonary thromboembolism (PTE). In the early stages of PTE, VE/VCO_2_values are not associated with functional class or severity.^1088^

## 5.6. Differential Diagnosis of Dyspnea

CPET is an important tool in the differential diagnosis of exertional dyspnea or exercise-induced dyspnea (EID), particularly of pulmonary and cardiac causes.^219 , 229 , 1018 , 1100 , 1101^Poor cardiorespiratory fitness, common to the probable etiologies of EID, is not a good isolated parameter for differential diagnosis.^219 , 229 - 231 , 1018 , 1100 - 1104^

Parameters used in the differential diagnosis of EID:^219 , 229 - 231 , 1018 , 1100 - 1104^

– Ventilatory efficiency assessed through VE/VO_2_and VE/VCO_2_, ventilatory reserve, and dead space/tidal volume ratio (VD/DT).

– Analysis of the flow-volume loop.

– Arterial oxygen saturation, which contributes to the detection of derangements in lung diffusion.

In lung diseases, EID is associated with high ventilatory equivalents, reduced VR, increased VD/DT ratio, reduced PETCO_2_, and oxygen desaturation, observed concomitantly or separately.^219 , 229 - 231 , 1018 , 1100 - 1104^

Regarding the ventilatory limitations of EID (Table 49):^219 , 229 - 231 , 1018 , 1100 - 1104^

– Pre-CPET spirometry allows detection and characterization of ventilatory disorders (obstructive, restrictive, and mixed).

– VT1 is often not detected (with RQ <1.0).

– Upon cessation of effort due to ventilatory limitation, low VO_2_and HRpeak are generally observed.

– A decrease in arterial oxygen saturation >5% (measured by pulse oximetry) indicates limitation of pulmonary etiology.

– The ΔVO_2_/ΔWR and VO_2_/HR (oxygen pulse) ratios are normal, except when there is comorbid PAH with cardiac output involvement.

– On post-exercise spirometry performed within 30 minutes after peak exertion, a reduction in FEV1 ≥15% is indicative of exercise-induced bronchospasm.

Differential diagnosis of EID secondary to HF:^219 , 229 , 1014 , 1018 - 1020 , 1028 - 1033 , 1035 , 1037 - 1039 , 1048 - 1056 , 1060 , 1061 , 1105^

– Elevated VE/VCO_2_and reduced VO_2_max correlate with the severity of HF.

– Preserved ventilatory reserve, often without arterial oxygen desaturation, is indicative of EID of cardiac etiology. Complex congenital heart diseases are an exception, as desaturation is common in this setting.

– Increased VD/VT ratio and presence of exercise oscillatory ventilation (EOV) are markers of worse prognosis in HF.

Cardiac and pulmonary causes may overlap, generating mixed patterns, reflecting secondary effects of the underlying disorder or of comorbid disorders.

Some patients develop dyspnea and/or exercise intolerance as sequelae of COVID-19, including in the chronic form of the disease (“long COVID”). In a post-COVID-19 context, other sequelae should also be investigated: pulmonary fibrosis, pulmonary thromboembolism, myocarditis, ventricular diastolic and/or systolic dysfunction, and myopathy.^1106 , 1107^

## 5.7. Athletes and Exercise Enthusiasts

Athletes are a heterogeneous population. Their activities range from high-workload aerobics (i.e. marathon runners) to primarily technical sports involving little physical demand (i.e. target shooting).^1106^

CPET is primarily aimed at:

– Direct determination of VO_2_, which is the gold standard for assessing cardiorespiratory fitness.

– Determination of ventilatory thresholds (VT1 and VT2), enabling individualized, optimized prescription of aerobic training.^219 , 229 , 293 , 1011^

## 5.8. Cardiorespiratory Rehabilitation

When prescribing exercise for patients undergoing cardiac rehabilitation, the following key aspects should be taken into account:

– Cardiorespiratory fitness should be determined through VO_2_max.^737 , 1012 , 1069 , 1070^

– Ventilatory thresholds (VT1 and VT2) should be ascertained for individualized definition of the optimal aerobic training zone.^17 , 219 , 229 , 293 , 1011^

– Claudication distances (initial and absolute), determined on a treadmill ergometer.

– In PAD, use of a cycle ergometer will allow better assessment of cardiopulmonary fitness.

– Ischemic threshold and its clinical repercussions (angina, arrhythmias, hypotension), to allow fine-tuning of therapy and exercise intensity.^17 , 29^

– In patients with a history of MI leading to LV dysfunction, rehabilitation increases PETCO_2_, which is associated with improved cardiac output.^1019 , 1020^

## 6. Interpretation and Preparation of the CPET Report

The interpretation and description of all ET variables must be incorporated into the CPET report, as set out in this Guideline.

Clinicians are advised to include the following in their reports:

– Detailed description of the behavior of CPET variables, particularly those found to be abnormal and/or pertinent to the reason for requesting a CPET.

– Reference ranges for the variables of interest, preferably with data from the population to which the patient belongs if available (age, sex, ethnicity, level of daily physical activity, etc.).

– Graphs and tables of the variables of interest and their respective interpretations, based on the indication for CPET and possible diagnoses.

– Information regarding risk stratification/prognosis.

Considering that exercise prescription is the responsibility of the attending (i.e. referring) physician, automatic clearance for such prescription based exclusively on CPET findings is not recommended.

## Part 4 – Exercise Test Combined with Cardiac Imaging Methods

### 1. Cardiovascular Stress Combined with Cardiac Imaging Methods

According to the Update of the Brazilian Guideline on Nuclear Cardiology, the basic principle of combining cardiovascular stress with myocardial perfusion images and echocardiography consists of creating heterogeneity in blood flow between vascular territories irrigated by normal coronary arteries and those irrigated by arteries with significant obstructive lesions.^1109 , 1110^

ET and pharmacologic stress, combined with MPI and echocardiography, are used to assess heterogeneity in regional blood flow, and the resulting images show similar sensitivity and specificity for detection of CAD.^8 , 1111 , 1112^

### 1.1. Myocardial Perfusion Imaging

The two existing cardiovascular stress modalities (physical exercise and pharmacological vasodilation) have shown similar sensitivity and specificity for the detection of CAD by analysis of perfusion images.^9^Figure 34 presents an algorithm to guide selection of the stress method to be employed.

#### 1.1.1. Physical Stress Methods (Exercise Test)

Physical stress, through ET or CPET, adds diagnostic and prognostic value to imaging methods by addressing clinical, hemodynamic, and electrocardiographic parameters. To obtain the best results, the methodological specificities of each imaging modality, discussed below, must be considered and adopted.^9 , 762 , 1111^

**1.1.1.1. Contraindications to Physical Stress in MPI**

The contraindications to physical stress in this setting are the same as for conventional exercise test, described in Part 1, Section 2.3 of this Guideline.

**1.1.1.2. Guidelines for Patients when Scheduing MPI with Physical Stress**

Preparation for the procedure involves:

– 3-hour fast with a light meal before the test.

– Methylxanthine (caffeine)-containing foods, beverages, and drugs should be withheld for at least 12 hours before the test, as some tests may need to be converted to pharmacologic or hybrid stress and would thus require vasodilator administration (Appendix 3).

– Discontinuation of drugs that may interfere with ET/CPET (especially antiarrhythmics and antianginal drugs, i.e. beta-blockers, calcium channel blockers, and nitrates) should follow the recommendations in Part 2, Section 1.1.6 of this Guideline, and is delegated to the requesting/referring physician.^1111^

– Patients are advised to bring the reports of any recently performed ET/CPET, to inform better selection of the effort protocol and allow comparison between tests.

– The other orientations for ET described in Part 2 of this Guideline all apply.

**1.1.1.3. Methodological Aspects of Physical Stress in MPI**

Before ET, peripheral venous access in one of the upper extremities must be obtained for injection of the radiopharmaceutical.^6^This injection should be performed at peak exercise, which may not correspond to the predicted HRmax; the indication and purpose of the test (diagnosis or assessment of response to therapy) must be taken into account (Class of recommendation - Level of evidence: IIb-C).^1111^

Following injection of the radiopharmaceutical, the patient should be encouraged to continue exercise at the same load for one additional minute, to improve myocardial uptake of the radiotracer. If the same load cannot be maintained, the clinician should attempt to reduce the speed and/or grade of the ergometer instead of abrupt cessation.^9^

Images should be acquired after the end of the exercise phase:

– When using MIBI-^99M^Tc (used in most services in Brazil), 30 to 60 minutes after cessation of stress.

– When using Thallium-201, acquisition should start no later than 10 to 15 minutes after cessation of stress, to prevent significant redistribution. Initial or distribution images should be acquired after the radioisotope injection (single injection), and late (redistribution) images, 3 to 4 hours later.

Radiation exposure of staff performing ET varies widely depending on the facility’s volume and preventive measures in place, but the effective dose received by physicians performing ET generally remains below acceptable limits. Clinicians are advised to familiarize themselves with and adopt all procedures recommended by the Brazilian National Nuclear Energy Commission for MPI.

**1.1.1.4. Interpretation of ET Results in MPI**

All diagnostic and prognostic markers described in this Guideline regarding conventional ET apply to the interpretation of combined ET/MPI, and must be included in the final report. The percent predicted HRmax at the time of injection of the radiopharmaceutical must be included in the report to allow analysis and interpretation of MPI.

#### 1.1.2. Methodological Aspects of Pharmacologic Stress in MPI

Pharmacologic stress tests may be performed with: primary vasodilators (adenosine and dipyridamole), which cause redistribution of coronary flow; or drugs that promote increased myocardial oxygen consumption, such as dobutamine and atropine, which cause effects similar to those observed in dynamic exercise with increased myocardial workload.

Pharmacologic stress tests are preferred: in patients with physical limitations to exercise due to musculoskeletal or neuromuscular conditions; in those patients on drugs that interfere with myocardial oxygen consumption (MVO_2_), when ET is performed for diagnostic purposes; those with low functional capacity; in compensated HF; in LBBB, when an artificial pacemaker is present; and in patients with other contraindications to physical exercise.

**1.1.2.1. Vasodilators**

**1.1.2.1.1. Dipyridamole**

Dipyridamole acts by inhibiting the enzyme adenosine deaminase, which degrades endogenous adenosine, in addition to blocking adenosine reuptake by the cell membrane, with consequent increases in extracellular adenosine concentration leading to coronary and systemic vasodilation. Its biological half-life is approximately 45 minutes. The recommended dose for MPI is 0.56 milligrams per kilogram (mg.kg^- 1^) per minute, up to a maximum dose of 60 mg, administered intravenously over the course of 4 minutes, diluted in 50 mL of saline solution. Dipyridamole can be injected manually, without an infusion pump. The radiopharmaceutical is administered through the same intravenous (IV) line, 2 to 4 minutes after completion of the dipyridamole infusion (hyperemia or maximum vasodilation).^9 , 1109 - 1111^

Adverse effects of vasodilators occur in 50% of patients receiving dipyridamole and in 80% of those receiving adenosine. With dipyridamole, these manifestations are reversed upon administration of intravenous aminophylline, at a dose of 1 to 2 mg.kg^- 1^up to 240 mg, 2 minutes after radiotracer injection. Vasodilators (sensitivity and specificity ≈80-90%) and physical stress (sensitivity and specificity ≈85-90%) have comparable accuracy for the detection of CAD.^1113 , 1114^

**1.1.2.1.2. Adenosine**

Adenosine induces coronary vasodilation by specific activation of A_2A_receptors in the cell membrane, resulting in a 4- to 5-fold increase in coronary flow. The usual dose is 140 μg/kg/min, given via continuous infusion over 6 minutes. Use of an infusion pump is mandatory. During the third minute of infusion, the radiopharmaceutical is injected via another peripheral venous line (due to the ultra-short plasma half-life of 2 to 10 seconds), and the adenosine infusion is continued for a further 3 minutes.

When patients are markedly symptomatic or show ischemic changes at the start of the adenosine infusion, a shorter 4-minute infusion protocol can be used instead, which has been shown to be able to detect ischemia and CAD. In this protocol, the radiopharmaceutical is injected during the second minute and the adenosine infusion continued for a further 2 minutes.

Clinical status and ECG, BP, and HR must be monitored continuously during pharmacologic stress testing with vasodilators, and for a few minutes afterwards.

Preparation for the procedure:

**–** 3-hour fast.

**–** Avoid intake of any food, beverage, or other product containing methylxanthines (competitive adenosine receptor inhibitors), including coffee, tea, chocolate, energy drinks, soft drinks or other caffeinated beverages, caffeine-containing medications (analgesics, cold and flu medicines, muscle relaxants, nonsteroidal anti-inflammatory drugs) for 12 hours before the test.

– The aforementioned foods, beverages, and medications must be avoided for at least 12 hours^1111^before the test if adenosine will be used and for at least 24 hours if dipyridamole will be used.^9^

**–** Theophylline should be discontinued at least 12 hours before the test.

**–** When using adenosine, two IVs must be placed: one for adenosine infusion and another for injection of the radiopharmaceutical. When using dipyridamole, only one IV line is required.

**–** An infusion pump is mandatory for administration of adenosine and optional for administration of dipyridamole.

**–** Aminophylline (1 to 2 mg.kg^- 1^, i.e. a 72 to 240 mg dose) should be readily available to reverse any serious side effects of vasodilator stress. In case of dipyridamole-induced adverse effects, aminophylline should be administered 2 minutes after the radiotracer injection.

Contraindications:^1110 , 1111 , 1115^

1) Relative: history of reactive airway disease with no exacerbations or episodes for at least 3 months; sinus node dysfunction; marked sinus bradycardia; severe bilateral carotid artery disease.

2) Absolute: active bronchospasm; status asthmaticus; recent (<3 months) episodes of reactive airway disease; second- or third-degree AV block (unless an artificial pacemaker is in place); systolic hypotension (<90 mmHg); recent (<2 months) stroke or TIA; recent (<24 hours) use of dipyridamole, if the test is to be performed with adenosine.

**1.1.2.2. Drugs that Increase Myocardial Oxygen Consumption**

These drugs are an alternative for patients who cannot undergo ET or pharmacologic stress with dipyridamole or adenosine. The most commonly used agent is dobutamine, which acts predominantly on beta-1 (ß-1) adrenergic receptors, with inotropic and chronotropic stimulation, and to a lesser extent on beta-2 (ß-2) receptors, with a peripheral vasodilation response dependent on the infused dose. It increases cardiac output, HR, and SBP, leading to an increase in MVO_2_and, consequently, to coronary vasodilation.

Dobutamine is administered via intravenous infusion pump at an initial dose of 5-10 μg/kg/min for 3 minutes, followed by incremental doses of 20 μg/kg/min and 30 μg/kg/min up to a maximum of 40 μg/kg/min.^1116^In patients who do not reach submaximal HR and have no evidence of ischemia, intravenous atropine can be added at a dose of 0.25 to 2 mg.^1110^If necessary, handgrip maneuvers (i.e. squeezing a tennis ball) can be added as well. Early use of atropine after the first phase of dobutamine infusion has proven to be safe and reduce infusion time and patient complaints during stress, without altering the diagnostic accuracy of the test.^1117^

The radiotracer is injected at HRpeak, and dobutamine infusion should be continued for 1 minute thereafter.

Throughout the test, clinical signs and symptoms should be monitored and ECG, BP, and HR recorded continuously. For reversal of adverse effects, short-acting beta-blockers (i.e. metoprolol or esmolol) can be injected intravenously after the first minute of radiotracer injection.

Preparation for the procedure:

**–** 3-hour fast.

– Withhold any beta-blockers for 48 to 72 hours before dobutamine stress.

– Peripheral venous access for infusion of dobutamine and radiotracer.

– Dobutamine should only be administered via infusion pump.

– Metoprolol (5 mg) should be readily available to reverse any serious side effects of dobutamine. However, it is contraindicated in patients with history of severe bronchospasm and in those with COPD. In these patients, esmolol (single dose, 100 and 200 mg), a cardioselective beta-blocker, is preferred.

Contraindications:

1) Relative: abdominal aortic aneurysm (>5cm diameter), presence of thrombi in the left ventricle, LVEF <25% (increased risk of ventricular arrhythmias).

2) Absolute *:* cardiac arrhythmias (sustained or paroxysmal atrial fibrillation or ventricular tachycardia), severe aortic stenosis, hypertrophic obstructive cardiomyopathy, systolic hypotension (<90 mmHg) or hypertension (>200 mmHg), unstable angina or recent MI, aortic aneurysm or dissection, symptomatic cerebrovascular insufficiency, presence of ICD, alterations in potassium metabolism.^9^

#### 1.1.3. Hybrid Stress Methods

These are ideal for patients who have limited exercise capacity or are on medications that prevent the HR response to exercise. Hybrid stress is not recommended in patients with LBBB, WPW syndrome, or an artificial pacemaker.

The combination of vasodilators with low-workload exercise (i.e. up to the second stage of the Bruce protocol or slight fatigue) has been shown to reduce subdiaphragmatic (hepatic) activity and improve image quality.^1118^It also helps reduce the occurrence and severity of adverse effects seen with dipyridamole and adenosine.

In in selected patients, a hybrid stress protocol can consist of applying the first stage of the modified Bruce protocol (1.7 mph, 0% grade) for 4 to 6 minutes after dipyridamole infusion. The radiopharmaceutical is then injected during exercise, which must continue for another 2 minutes to allow adequate myocardial uptake, reducing side effects and improving image quality. Regarding preparation for the procedure, the same guidance applies as for exercise- and vasodilator-based protocols.

#### 1.1.4. Novel Drugs

The selective A_2A_receptor agonist regadenoson, which promotes coronary vasodilation, has been shown to induce adequate coronary hyperemia with a lower severity of systemic effects (such as chest pain and AV blockade). Regadenoson, which is not yet available in Brazil, has a short biological half-life (2 to 4 minutes), which shortens test time and minimizes and limits the duration of adverse effects. It can be used in patients with COPD or asthma.^1119^

The recommended dose of regadenoson is 0.4 mg IV (no adjustment for weight required), injected into a peripheral vein followed by a 5-mL flush of saline solution; the radiopharmaceutical should be administered 10 to 20 seconds later.^1120^

## 1.2. Stress Echocardiography

The different stress modalities used for stress echocardiography have been compared elsewhere in this Guideline, including their physiological effects, hemodynamic responses, and contraindications (see Table 18). In most adult patients capable of exercising, exercise stress echocardiography (ESE) is considered the modality of choice for assessment of myocardial ischemia.

### 1.2.1. Methodology 8,260,265,1121,1122

First, a baseline echocardiogram should be obtained at rest to assess cardiac structure (chamber sizes and wall thickness), heart valves and their gradients, regional wall motion, and global ventricular function.

Baseline echocardiography also aims to investigate potential causes for the patient’s symptoms (i.e. aortic dissection) and to identify any condition that might make stress unsafe (i.e. severe valve disease in a symptomatic patient). Under these circumstances, the possibility of postponing or canceling the stress test altogether should be carefully evaluated.

The acquisition, analysis, and interpretation of echocardiographic data (baseline and stress) should follow the recommendations of international organizations^260 , 265 , 1121^and relevant Brazilian Society of Cardiology position statements and guidelines: Position Statement on Indications of Echocardiography in Adults;^8^Position Statement on Indications for Echocardiography in Fetal and Pediatric Cardiology and Congenital Heart Disease of the Adult;^1121^and Standardization of Examination Equipment and Techniques for Performing Echocardiographic Examinations ( *Normatização dos Equipamentos e Técnicas de Exame para Realização de Exames Ecocardiográficos* ).^1122^

Images obtained at rest will be compared with those obtained during and after stress. Obtaining data across multiple cardiac cycles at peak stress increases the accuracy of interpretation. Continuous recording of resting and stress images is recommended.

In treadmill tests, images obtained at rest and immediately after exercise should be compared side by side (in quad screen format). In pharmacologic stress tests, images obtained at peak stress should be compared with resting, low-dose, and pre-peak or early-recovery images (also using the quad screen format).

Echocardiographic images are generally obtained from parasternal long-axis, short-axis, and apical two–and four-chamber views. In some cases, the subcostal and apical long-axis views may be used. Other views and maneuvers may be necessary on a case by case basis.

Development of wall thickening under stress in segments which are markedly hypokinetic or akinetic at rest is indicative of myocardial viability. For proper determination of viability, myocardial thickening must be observed carefully at lower levels of stress, to avoid a rapid increase in HR and associated ischemia.

Abnormal changes in diastolic function may precede abnormalities in systolic wall motion. When detection of ischemia is the primary objective of a stress test, the clinician is advised to record diastolic parameters close to peak effort or after assessment of regional wall motion.

Color Doppler assessment of mitral regurgitation on baseline echocardiography and during peak exertion may allow detection of ischemic mitral regurgitation.

Stress echocardiography is as accurate as positron emission tomography in detecting reversible dysfunction in patients with hibernating myocardium.

**1.2.1.1. Exercise Stress Methods^8 , 1123^**

Just as conventional ET protocols, exercise stress echocardiography can be performed on a treadmill or cycle ergometer. The most widely used treadmill-based method is the Bruce protocol, with echocardiographic images being acquired at rest and immediately upon completion of exercise.

If using a cycle ergometer, the patient can remain vertical (bicycle or conventional cycle ergometer) or supine (supine cycle ergometer or in-bed cycle ergometer). On a supine cycle ergometer with lateral rotation, the patient pedals in an inclined position, allowing better adaptation and yielding higher-quality echocardiographic images. The most widely used cycle ergometer protocol, the Åstrand test, starts with a load of 25W that is increased progressively in 25W increments every 2 or 3 minutes (Figure 35).

Skin preparation and electrode placement for ECG monitoring are as those standardized for ET. The leads may be displaced slightly up and down in case they interfere with the acoustic windows needed to perform echocardiography.

A 12-lead ECG should be recorded at rest and every 2 minutes (or at every stage) throughout the test (during exertion and recovery). One ECG lead should be continuously displayed on the echocardiograph monitor to provide a reference of ST-segment changes and arrhythmias.

BP should be measured at rest, at each stage of the exercise protocol, and during recovery.

Analysis and interpretation of HR, BP, scores (pre- and post-test), ECG recordings, and cardiorespiratory fitness parameters are similar to those for ET. Correlation of these data with echocardiographic findings is recommended.

Although some facilities terminate the test when 85% of the predicted HRmax for age is reached, all are advised to continue the test until physical exhaustion (Borg scale ≥18) or until absolute criteria for test cessation arise. Reaching the predicted HRmax increases the sensitivity of the test and may reveal abnormalities that would only occur at a high enough workload.^8 , 260 , 1123^

**1.2.1.2. Pharmacologic Stress Methods**

**1.2.1.2.1. Dobutamine^260 , 1123^**

Pharmacologic stress with dobutamine (or dobutamine plus atropine) is the most widely used alternative modality for assessing myocardial ischemia when a patient is unable to exercise.^1124^

Dobutamine is usually administered in graded doses, starting at 5 μg/kg/min, with increments at 3-minute intervals to 10, 20, 30, and 40 μg.kg^- 1^.min^- 1^. Dobutamine doses of 20 or 30 μg.kg^- 1^.min^- 1^generally allow reaching target HR (85% of maximum predicted HR for age) more quickly and with fewer side effects, especially if HR is not increasing as expected.

Infusion of dobutamine at a low initial dose (2.5 μg.kg^- 1^.min^- 1^) may facilitate recognition of myocardial viability in echocardiographically abnormal segments.

In beta-blocked patients and/or those in whom the predicted HRmax is not reached, the sensitivity of DSE for detection of ischemia is reduced. In such cases, atropine can be administered to achieve the target HR (0.25 to 0.50 mg, at 1-minute intervals, as needed; maximum cumulative dose 2.0 mg). To avoid side effects, including central nervous system toxicity, the lowest possible dosage should be used.^1125^

In older adults, patients with a small body habitus, and in those who are already close to the predicted HRmax, the use of atropine in a dose of 0.25 mg is recommended. A total dose <1.0 mg is also recommended in patients with neuropsychiatric conditions, patients with liver disease, or those with a BMI <24 kg/m^2^.

Pharmacologic stress should be terminated upon achieving target HR, hypotension, LV wall motion abnormalities (new or worsening), complex arrhythmias, severe hypertension, and intolerable symptoms, among other endpoints.

**1.2.1.2.2. Vasodilators^260 , 1123^**

Pharmacologic stress testing with vasodilators (dipyridamole or adenosine) induces an increase in coronary flow, and should preferably be used to assess myocardial perfusion.^1126^It can also be performed to assess ischemia and myocardial viability. Pharmacologic stress echocardiography with vasodilators has a lower sensitivity for CAD detection than ESE or DSE.

Vasodilating agents are contraindicated in patients with reactive airway obstruction, unstable or complicated acute coronary syndrome, severe cardiac arrhythmias (VT and AV block), or severe hypotension.

Dipyridamole is usually given in doses of up to 0.84 mg.kg^- 1^over 6 to 10 minutes. Administering atropine or performing a handgrip exercise at peak infusion increases the sensitivity of the test.^1127^

Adenosine in conjunction with contrast-enhanced echocardiography can also be used to assess myocardial perfusion. The adenosine infusion rate is 140 μg.kg^- 1^.min^- 1^over 4 to 6 minutes. Adenosine has a shorter half-life and, therefore, a shorter duration of action than dipyridamole.

**1.2.1.3. Ultrasound Enhancing Agents**

Three microbubble ultrasound enhancing agents (UEAs) are commercially available worldwide: *Sonovue* ^®^, *Definity* ^®^, and *Optison* ^®^. UEAs are used to improve endocardial border delineation, image quality, and CAD detection by stress echocardiography. Management (whether by bolus or continuous infusion) aims to opacify the entire LV cavity, without swirling artifacts in the apex or attenuation of basal segments due to acoustic shadowing. UEAs can also be used as myocardial perfusion markers.

Use of these agents in nonpregnant adults is safe; anaphylactic reactions may occur, but are very rare. UEA administration is not recommended in pregnancy. Specific administration protocols are given in the 2018 ASE guidelines.^1128^

**Table 1:** – Indications for ET in symptomatic and asymptomatic coronary artery disease

Indication	Class of recommendation	Level of evidence
Patients having an intermediate pretest probability of CAD, including those with right bundle branch block or less than 1 mm of resting ST-segment depression^14,31^	I	A
Differential diagnosis of chest pain in low-risk, clinically and hemodynamically stable (for at least 9 to 12 hours) patients with no ECG signs of ischemia or ventricular dysfunction and normal cardiac markers, in the chest pain unit^32,33^	I	A
Exercise prescription and serial longitudinal assessment within a rehabilitation program^29,30^	I	A
Assessment of atypical symptoms and resting ECG abnormalities (interpretable) to clear patients for high-intensity physical activity^17,34^	I	A
Risk stratification and therapeutic definition in acute coronary syndromes, after at least 72 hours of complete clinical and hemodynamic stability^33,35^	I	B
After uncomplicated MI, before hospital discharge, for risk stratification and optimization of therapy^36,37^	I	B
Prognostic assessment in stable CAD*^38,39^	I	B
Investigation of CAD in symptomatic diabetic patients with interpretable ECG^40-42^	I	B
Suspected vasospastic angina^43,44^	IIa	B
Risk stratification and selection of therapy in patients at high risk of CAD^14,45^	IIa	B
Assessment of asymptomatic patients with three or more classic risk factors^46,47^	IIa	B
Therapeutic decision-making in intermediate coronary lesions detected on coronary angiography^14,26^	IIa	B
Assessment of pharmacotherapeutic efficacy in CAD^27,48^	IIa	B
Investigation of changes in ventricular repolarization (provided that <1 mm depression) on resting ECG^6,14^	IIa	B
Assessment of patients still symptomatic after CABG or percutaneous coronary intervention^49,50^	IIa	B
Assessment of asymptomatic patients after myocardial revascularization (CABG or percutaneous coronary intervention) for risk stratification, optimization of therapy, clearance for physical exertion/exercise prescription, including rehabilitation^14,49^	IIa	B
Preoperative assessment of patients at intermediate or high risk of complications**^51,52^	IIa	C
Investigation of CAD in patients with ECG criteria for left ventricular hypertrophy with ST-segment depression <1 mm^53,54^	IIb	B
Functional assessment when another method has been used to evaluate coronary anatomy^6,14^	IIb	B
Disability/worker’s compensation and/or work capacity assessment^55,56^	IIb	B
Cardiovascular risk stratification in persons with low probability of CAD^24^	IIb	C
Asymptomatic patients with LMCA lesion or known equivalent, for longitudinal follow-up and therapeutic optimization/decision-making^6,14^	IIb	C
Acute coronary syndromes not yet clinically or hemodynamically stable or with persistent ECG changes or abnormal cardiac markers^14,33^	III	B
CAD screening in patients with LBBB, WPW syndrome, PM rhythm, ST-segment depression ≥1 mm on resting ECG, and digitalis therapy^6,14^	III	B
Presence of LMCA lesion or known symptomatic equivalent^6,14^	III	B
ECG: electrocardiogram; MI: myocardial infarction; CAD: coronary artery disease; HTN: hypertension; PM: pacemaker; LBBB: left bundle branch block; LMCA: left main coronary artery; WPW: Wolff-Parkinson-White. *Prognostic/longitudinal assessment of CAD may be required annually, depending on clinical condition. **For a classification of the intrinsic risk of cardiac complications of noncardiac surgeries, refer to the 3rd Guideline for Perioperative Cardiovascular Evaluation of the Brazilian Society of Cardiology.^*51,52*^		

**Table 2:** – Indications for ET in asymptomatic patients

Indication	Class of recommendation	Level of evidence
Assessment of individuals with a family history of early CAD (in women <65 years and in men <55 years) – perform at least one ET by age 40^45,62^	I	B
Screening of individuals with a history of sudden death in first-degree relatives^55,63^	IIa	B
Assessment of sedentary diabetics for the diagnosis of moderate or severe exertion-induced symptoms and/or exercise prescription^41,64,65^	IIa	B
Individuals classified as high risk by the Framingham score^1,62^	IIa	B
Individuals with high-risk occupations and/or responsible for the lives of others, such as aircraft pilots, professional drivers, military personnel, law enforcement officers, firefighters, etc.^14,66^	IIa	B
Preparticipation physical evaluation of individuals ≥60 years old before engagement in leisure activities and recreational sports^17,34^	IIa	C
Preoperative assessment of patients with a family history of early CAD scheduled to undergo moderate and major noncardiac surgery^52,67^	IIa	C
May be considered for preparticipation physical evaluation of individuals aged 35-59 before engagement in leisure activities and recreational sports^17,34^	IIb	B
Preparticipation physical evaluation of individuals aged <35, without cardiovascular risk factors, before starting a light or moderate physical activity program^34^	III	C
*CAD: coronary artery disease; ET: exercise test.*		

**Table 3:** – Indications for ET in athletes

Indication	Class of recommendation	Level of evidence
In individuals ≥60 years old before starting any high-intensity activity, sports, and before participating in sports competitions^13,17,69^	I	B
Screening of individuals with a history of sudden death in first-degree relatives^13,17, 70^	I	B
Preparticipation physical assessment of individuals aged ≥35 years at high risk (clinical score), before high-intensity exercise and sports competitions^13,17, 34^	IIa	A
May be considered for preparticipation physical evaluation of individuals aged 35-59 before engagement in high-intensity exercise and sports competitions^13,17,34^	IIa	B
Assessment of individuals with a family history of early CAD (in women <65 years and in men <55 years) – perform at least one ET by age 35^13,17,34^	IIa	B
Diagnosis of exercise-induced signs and symptoms, risk stratification, and prognosis in athletes with diabetes^17,40,41,64^	IIa	B
Before adjusting the physical training load of athletes	III	C
Athletes with symptomatic overtraining syndrome	III	C
*CAD: coronary artery disease; ET: exercise test.*		

**Table 4:** – Indications for ET in hypertension

Indication	Class of recommendation	Level of evidence
Investigation of CAD in symptomatic hypertensive patients with normal ECG^27,77,78^	I	B
Assessment of BP response in patients with the metabolic syndrome or diabetes^79,80^	IIa	B
For assessment of cardiorespiratory fitness, risk stratification, and clearance for sports practice in hypertensive patients^71,76,81,82^	IIa	B
To inform adjustment of antihypertensive therapy^83-85^	IIa	B
Assessment of BP response in patients under investigation for hypertension^86,87^	IIa	B
Assessment of BP response in hypertensive patients with CAD, for risk stratification, therapeutic optimization, and clearance for physical exercise^78,88^	IIa	B
Assessment of hypertensive older adults before starting a physical activity program^29,34,89^	IIa	C
Suspected exercise-induced hypotension in patients with treated hypertension^90,91^	IIa	B
Assessment of BP response in individuals with a family history of HTN^71^	IIb	B
*CAD: coronary artery disease; HTN: hypertension; BP: blood pressure; ECG: electrocardiogram.*		

**Table 5:** – Indications for ET in valvular heart disease

Indication	Class of recommendation	Level of evidence
In mild and moderate valvular heart disease, to confirm absence of symptoms, elucidate unclear symptoms, assess functional capacity, and inform exercise prescription^93,94,96,97^	I	B
In mitral regurgitation, to elucidate unclear symptoms, assess functional capacity, indicate intervention, and determine prognosis^98-100^	IIa	A
In AS, to elucidate unclear symptoms, indicate intervention, and determine prognosis^93,94,101,102^	IIa	A
In asymptomatic patients with moderate and severe AS, to evaluate markers of poor prognosis and indicate intervention^93,94,96,101,103^	IIa	A
During follow-up of AR, to elucidate unclear symptoms, assess functional capacity, and determine prognosis^104,105^	IIa	B
In asymptomatic MS, MS with atypical symptoms, or symptoms discordant with the degree of stenosis^14,106,107^	IIa	B
During follow-up of asymptomatic severe AS, at least every 6 months, for early detection of symptoms, functional assessment, and indication of intervention^93,108,109^	IIa	B
For preconception family planning in severe, asymptomatic AS with normal LVEF^110,111^	IIa	B
After any valvular intervention to elucidate symptoms, assess functional capacity, determine prognosis, and inform exercise prescription (including cardiovascular rehabilitation)^93,112^	IIa	B
To determine surgical risk and functional capacity before noncardiac surgery^52,67,113^	IIb	B
In asymptomatic aortic and mitral stenosis or regurgitation, to determine functional capacity and inform exercise prescription^17,29,114^	IIb	B
Investigation of CAD in patients with severe valvular heart disease^115^	III	B
In severe, symptomatic AS or MS^93^	III	C
*AS: aortic stenosis; AR: aortic regurgitation; CAD: coronary artery disease; LVEF: left ventricular ejection fraction.*		

**Table 6:** – Indications for ET in heart failure and cardiomyopathies

Indication	Class of recommendation	Level of evidence
In compensated HF and cardiomyopathies, for exercise prescription and optimization (including cardiovascular rehabilitation programs)*^6,13,17,29,119^	IIa	B
In hypertrophic cardiomyopathy and compensated HF, using a modified protocol, to elucidate symptoms, assess functional capacity and prognostic markers (symptoms, ventricular arrhythmia, BP response)^17,29,120,121^	IIa	B
In hypertrophic cardiomyopathy, serially, to inform adjustment of exercise programs and participation in recreational sports^6,13,17,29,121^	IIa	B
In asymptomatic patients recovered from myocarditis, 3 to 6 months after the acute episode, to clear patients for exercise and inform the exercise prescription^122,123^	IIa	B
After heart transplantation*, for exercise prescription (including cardiovascular rehabilitation) and optimization^13,29,124,125^	IIa	C
In hypertrophic cardiomyopathy or compensated HF, serially, to assess BP response and response to therapeutic interventions^14,18,119,121^	IIa	C
Periodic reassessment after myocarditis, within the first 2 years after the acute episode, to identify silent disease progression and for risk stratification^115,123,126,127^	IIa	C
Selection for heart transplantation (based on estimated, not measured, VO_2_values)**^6,115^	III	B
Myocarditis, acute pericarditis, or decompensated HF^6,115^	III	C
Diagnosis of heart failure^6,115^	III	C
HF: heart failure; VO_*2*_: oxygen consumption. *If CPET is unavailable. **The variables obtained by CPET are essential for ascertaining whether heart transplantation is indicated, as they allow a more accurate detection of the mechanisms underlying exercise intolerance.		

**Table 7:** – Indications for ET in the context of arrhythmias and conduction disorders

Indication	Class of recommendation	Level of evidence
Palpitations, syncope, pre-syncope, syncope equivalents, undefined malaise, or pallor associated with physical exertion and/or recovery^6,14,133,134^	I	B
Asymptomatic arrhythmia detected during physical or other examination, for assessment of response to exertion and prognostic determination^14,39,133,135,136^	I	B
In congenital heart block, for assessment of ventricular response and indication of pacemaker placement^131,132,137,138^	I	B
In patients with catecholaminergic polymorphic ventricular tachycardia, for evaluation of pharmacotherapy and indication of implantable cardioverter-defibrillator placement^132,138,139^	I	B
In sinus node dysfunction, to assess the chronotropic response*^14,133,134^	I	B
In long QT syndrome (symptomatic and asymptomatic), for diagnostic confirmation, risk stratification, assessment of arrhythmogenic potential, and therapy^140,141^	I	B
Diagnostic suspicion of catecholaminergic polymorphic ventricular tachycardia^115,132,138,139^	I	C
In congenital heart block, for assessment of atrial response and, consequently, pacemaker selection^131,132,134,138^	I	C
Assessment of efficacy of pharmacological therapy and/or ablation^131-134,142^	IIa	B
Assessment of whether pacemaker placement is indicated^14,131,132,138,143^	IIa	B
In clinically stable survivors of cardiac arrest, to clear patients for exercise and inform the exercise prescription (recreational and/or cardiovascular rehabilitation)^13,144-146^	IIa	B
In Brugada syndrome (symptomatic and asymptomatic), for diagnostic confirmation, risk stratification, assessment of arrhythmogenic potential, and therapy**^147,148^	IIa	B
Suspected chronotropic incompetence^6,14,149-151^	IIa	B
For evaluation of heart rate behavior in patients with rate-responsive pacemakers^131,132,134,137,138,152^	IIa	B
To adjust programming of pacemakers, cardiac resynchronization therapy devices, and/or implantable cardioverter- defibrillators^131,132,134,137,138,152^	IIa	C
Screening of family members of patients with long QT syndrome^13,140,153,154^	IIa	B
In patients with known, controlled arrhythmia, to clear patients for exercise and inform the exercise prescription (recreational and/or cardiovascular rehabilitation)^6,13-14,133,134^	IIa	C
For annual assessment of asymptomatic patients with arrhythmogenic cardiomyopathy who engage in physical exercise^17,155-157^	IIa	C
In persistent (chronic) atrial fibrillation, for assessment of response to therapy, ventricular rate control, risk stratification, and clearance for exercise (including rehabilitation)^119,158,159^	IIa	C
Assessment of accessory pathway behavior (pre-excitation) and arrhythmogenic potential^6,13,14,133,134^	IIb	B
In arrhythmogenic right ventricular dysplasia, for risk stratification and clearance for physical exercise^14,134,135^	IIb	B
In patients with an implantable cardioverter/defibrillator, for assessment of function, prognosis, therapeutic efficacy, and clearance for physical exercise^131,132,160,161^	IIb	B
Screening of family members of patients with Brugada syndrome**^13,134,147,162^	IIb	C
In patients with a fixed-rate pacemaker^115,133^	III	B
Acquired third-degree (complete) AV block with slow ventricular rate response^115,133^	III	B
Uncontrolled arrhythmia, symptomatic arrhythmia, or arrhythmia with hemodynamic instability^115,133^	III	C
AV: atrioventricular. *Third-degree sinoatrial block is an absolute contraindication to ET. **Using high precordial leads and passive recovery.		

**Table 8:** – Indications for ET in other recognized clinical conditions

Indication	Class of recommendation	Level of evidence
Asymptomatic patients with anomalous origin of coronary artery, for risk stratification, definition of therapeutic approach, and medical clearance for physical exercise/sports^13,17,34^	IIa	B
3 months after surgical correction of anomalous origin of coronary artery, if asymptomatic, for medical clearance for physical exercise/sports^13,17,29,163^	IIa	C
Myocardial bridge, for risk stratification, therapeutic decision-making, and medical clearance for physical exercise^13,17,29,163,164^	IIa	C
In asymptomatic left ventricular noncompaction cardiomyopathy with LVEF ≥40%, with a low-to-moderate-intensity exercise protocol^17,127,165^	IIa	C
In Parkinson’s disease, for assessment of exercise tolerance and medical clearance/exercise prescription^19,166,167^	IIa	B
In sickle cell anemia, for assessment of functional capacity, risk stratification, and medical clearance/exercise prescription^168-170^	IIa	C
In patients undergoing cancer treatment, for medical clearance/exercise prescription (including rehabilitation)^171,172^	IIa	C
Risk assessment and prognosis after adverse effects of cancer treatment^173,174^	IIb	C
In peripheral artery disease, to assess claudication, quantify ischemia, stratify risk, and therapeutic decisions^175,176^	I	B
In peripheral artery disease, to assess functional capacity and prescribe and adapt a physical exercise program^176,177^	I	B
In patients receiving renal replacement therapy and renal transplant recipients, for exercise prescription (including cardiovascular rehabilitation) and optimization^178,179^	IIb	C
In asymptomatic aortic or other aneurysm, not meeting criteria for intervention, to inform adjustment of therapy (i.e. optimization of antihypertensive treatment) and medical clearance/exercise prescription (including rehabilitation)^180^	IIa	C
In clinically stable survivors of stroke or transient ischemic attack, to inform adjustment of therapy (i.e. optimization of antihypertensive treatment) and medical clearance/exercise prescription^181^	IIb	C
In clinically stable (functional class I and II) adults with congenital heart disease, to inform exercise prescription and optimization of exercise program^17,182,183^	IIb	B
*LVEF: left ventricular ejection fraction.*		

**Table 9:** – Main events and complications arising during ET

Event	Frequency	Notes
**Sudden cardiac death**	1 in every 10,000 exams	Dependent on the clinical condition and comorbidities.^6,184,185^
**Exercise-induced ventricular tachycardia**	0.05-2.3%	Increased risk of occurrence in those with a history of ventricular arrhythmias. Increased risk of CVD and all-cause mortality.^135,186,187^Common in suspected catecholaminergic paroxysmal VT, right ventricular outflow tract tachycardia, and fascicular LV tachycardia.^63,130,188^
**Paroxysmal supraventricular tachycardia**	3.4-15%	Increased risk of developing AF.^189^PSVT resulting from reentrant pathways usually requires drug therapy.^129,142,190^
**Exercise-induced premature ventricular contractions**	2-20%	When frequent, carry an increased risk of mortality (all-cause and CVD) and cardiovascular events.^191-195^More common in patients with CAD: 7% to 20%.^196^
**Exercise-induced atrial ectopy**	4-25%	Found in up to 10% of apparently healthy individuals and in up to 25% of those with CAD. Not associated with cardiac mortality or MI.^189,197,198^In older adults, however, is associated with increased risk of AF/atrial flutter.^199,200^
**Exercise-induced atrial fibrillation/atrial flutter**	<1%	These usually cause hemodynamic repercussions if there is rapid ventricular response.^197,201^
**Intermittent left bundle branch block**	0.4-0.5%	CAD and HF are the most prevalent causes. Increased risk of all-cause mortality and cardiovascular events.^202,203^
**Intermittent right bundle branch block**	0.25%	Usually associated with CAD.^202-204^
**Exercise-induced bradyarrhythmia/third-degree AV block**	<0.1%	In sinus node dysfunction, symptoms of HF and angina may occur.^133^In exercise-induced sinus bradycardia, syncope may occur due to the Bezold-Jarisch reflex.^205,206^Exercise-induced third-degree AV block may be associated with transient ischemia or severe degenerative disease of the conduction system.^207,208^
**Acute coronary syndrome**	0.1-0.5%	Requires immediate cessation of exertion.^6,209,210^
*CVD: cardiovascular disease; AV: atrioventricular; CAD: coronary artery disease; HF: heart failure; VT: ventricular tachycardia; MI: myocardial infarction; AF: atrial fibrillation.*		

**Table 10:** – Relative contraindications to ET/CPET and special measures **1,6,12-17**

Hospital setting + Special measures	Special measures
Acute chest pain: perform exclusively in hospital, ideally in a chest pain unit, strictly following protocol	Nonobstructive hypertrophic cardiomyopathy
Severe asymptomatic valve stenosis*	Single-chamber, ventricular, non-rate-response pacemaker (VVI pacing mode)
Severe valve regurgitation*	Advanced (NYHA class III) compensated heart failure
Uncomplicated MI (after day 5 and if clinically stable)	Recent (<2 months) stroke or transient ischemic attack^9^
Unstable angina (once stable for at least 72 hours)*	Aortic or other aneurysm not meeting criteria for intervention
Asymptomatic known left main coronary artery disease or equivalent*	Asymptomatic AF or flutter detected during pre-test evaluation in a patient claiming to be unaware of the arrhythmia**
Suspected complex tachyarrhythmia and/or bradyarrhythmia, long QT syndrome, and Brugada syndrome	Persistent or chronic AF or chronic atrial flutter with elevated resting HR**
Syncope of probable arrhythmogenic etiology or suspected exercise-induced high-grade or third-degree (complete) AV block	Pregnancy***
Dialytic renal failure	
Implantable cardioverter/defibrillator (ICD)	
Acyanotic complex congenital heart defects	
Severe or symptomatic pulmonary hypertension*	
Obstructive hypertrophic cardiomyopathy with resting gradient indicating severe disease*	
Severe anemia (hemoglobin <8.0 g/dL)**^211,212^	
AF: atrial fibrillation; AV: atrioventricular; HR: heart rate. *Risk/benefit ratio of CPET must be carefully evaluated. **Risk/benefit ratio of CPET must be carefully evaluated and will likely result in a decision to postpone or cancel the test. ***With a submaximal protocol, in specific situations in pregnancy (i.e. valvular and congenital heart diseases), after any absolute clinical and obstetric contraindications have been ruled out. Not recommended as routine practice.^*213,214*^	

**Table 11:** – Specific indications for CPET

Indication	Class of recommendation	Level of evidence
Exercise intolerance and differential diagnosis of dyspnea^14,224,225^	I	A
Investigation of dyspnea, chronic fatigue, and/or exercise intolerance in post-acute respiratory syndrome (including COVID-19)^226-228^	I	B
Assessment of cardiorespiratory fitness and medical clearance/exercise prescription (including rehabilitation) in post-acute respiratory syndrome (including COVID-19)^13,226,227^	I	B
Assessment of exercise-induced bronchospasm (combined with pre and post-exercise spirometry)^219,229-231^	IIa	B
In stable HF, for assessment of cardiorespiratory fitness, risk stratification, adjustment of therapy, and medical clearance/exercise prescription (including rehabilitation)^14,232,233^	I	A
In HF, to inform indication of ventricular support device placement or heart transplantation^3,14,224,232,234,235^	I	B
In CAD, for assessment of cardiorespiratory fitness, risk stratification, adjustment of therapy, and medical clearance/exercise prescription (including rehabilitation)^14,29,236^	I	B
In suspected CAD, for diagnostic investigation, risk stratification, and to inform treatment decision^237,238^	IIa	B
In asymptomatic severe aortic stenosis, to guide treatment decision^14,93,239,240^	IIa	B
In stable valvular heart disease, for assessment of cardiorespiratory fitness, adjustment of therapy, and medical clearance/exercise prescription (including rehabilitation)^13,17,29,163^	IIa	B
Valvular heart disease with discrepancy between clinical picture and echocardiographic findings (except in aortic stenosis)^14,92-94,240^	IIa	C
In adults with CHD, for assessment of symptoms, treatment decisions, risk stratification, and medical clearance/exercise prescription (including rehabilitation)^17,30,241,242^	I	B
Preparticipation physical evaluation of athletes with CHD^17,241-243^	IIa	B
After CABG or valve repair or replacement in competitive athletes, for risk stratification and medical clearance to return to sport^17,34^	IIa	B
In hypertrophic cardiomyopathy, for assessment of cardiorespiratory fitness, risk stratification, and medical clearance/exercise prescription (including rehabilitation)^14,121,244,245^	IIa	B
In pulmonary hypertension, for diagnosis and serial evaluation (at 6 to 12-month intervals)^14,246^	I	B
In pulmonary hypertension, for investigation of worsening symptoms and risk stratification^14,246^	IIa	B
In symptomatic patients post-acute (>3 months) pulmonary embolism with ventilation/perfusion (V/Q) mismatch, for diagnosis and follow-up of pulmonary hypertension^247^	I	B
In patients undergoing cancer treatment, for risk stratification and medical clearance/exercise prescription (including rehabilitation)^248,249^	I	B
Preoperative assessment for noncardiac surgery in patients with low functional capacity (<4 METs) and/or high cardiovascular risk^14,250^	IIa	B
*CAD: coronary artery disease; CHD: congenital heart disease; HF: heart failure.*		

**Table 12:** – Choice of cardiovascular stressor for myocardial perfusion imaging **26,31,128,251-253**

Indication	Class of recommendation	Level of evidence
Physical stress (ET), as long as there are no limitations or contraindications	I	A
Pharmacologic stress (dipyridamole or adenosine) in cases of LBBB, WPW syndrome, and artificial pacemaker	I	A
Pharmacologic stress (dipyridamole, adenosine, dobutamine) whenever physical stress (ET) is contraindicated	I	A
Pharmacologic stress (dipyridamole, adenosine, dobutamine) when there are limitations to physical stress (ET)	IIa	A
Combined protocol: low-workload physical stress after pharmacologic stress with dipyridamole or adenosine	IIa	A
*LBBB: left bundle branch block; ET: exercise test; WPW: Wolff-Parkinson-White.*		

**Table 13:** – Indication criteria for myocardial perfusion imaging in symptomatic patients **9**

Indication	Class of recommendation	Level of evidence	Score
High pretest probability of CAD, regardless of interpretable resting ECG and ability to exercise*	I	A	8
Intermediate pretest probability of CAD, with uninterpretable resting ECG or inability to exercise*	I	A	9
Intermediate pretest probability of CAD, with interpretable resting ECG and ability to exercise*	IIa	B	7
Low pretest probability of CAD, with uninterpretable resting ECG or inability to exercise*	IIa	B	7
Low pretest probability of CAD, with interpretable resting ECG and ability to exercise*	III	C	3
CAD: coronary artery disease; ECG: 12-lead electrocardiogram; ACS: acute coronary syndrome. *“Ability to exercise” defined as functional capacity to perform daily activities with an estimated metabolic expenditure of >5 METs and ability to use whichever ergometer is available.			

**Table 14:** – Indication criteria for myocardial perfusion imaging in asymptomatic patients and/or those having undergone prior cardiac testing **9,254**

Asymptomatic patients – detection of CAD/risk stratification	Class of recommendation	Level of evidence	Score
Low risk (ATP III criteria)	III	A	1
Intermediate risk (ATP III criteria) – uninterpretable ECG	IIa	B	5
Intermediate risk (ATP III criteria) – interpretable ECG	IIb	C	3
High risk (ATP III criteria)	I	A	7
High risk and calcium score (Agatston) between 100 and 400	IIa	B	7
Calcium score (Agatston) >400	IIa	B	7
High-risk Duke score (<-11)	I	A	8
Intermediate-risk Duke score (between -11 and +5)	IIa	B	7
Low-risk Duke score (>+5)	III	B	2
*Agatston: score that defines the presence and quantity of calcium in the coronary arteries, characterizing atherosclerosis; ATP III: Third Report of the National Cholesterol Education Program Expert Panel on Detection, Evaluation, and Treatment of High Blood Cholesterol in Adults; CAD: coronary artery disease.*			

**Table 15:** – Indication criteria for myocardial perfusion imaging in patients who have undergone revascularization procedures (CABG or PCI) **9,31,251,252,254**

Previous percutaneous or surgical revascularization	Class of recommendation	Level of evidence	Score
Symptomatic at any time	I	B	8
Asymptomatic, CABG ≥5 years prior	IIa	B	7
Asymptomatic, CABG <5 years prior	IIb	B	5
Asymptomatic, percutaneous revascularization ≥2 years prior	IIa	B	6
Asymptomatic, percutaneous revascularization <2 years prior	III	C	3
*PCI: percutaneous coronary intervention; CABG: coronary artery bypass graft surgery.*			

**Table 16:** – Indication criteria for myocardial perfusion imaging in patients with acute chest pain or post-acute coronary syndrome **1,9,33,254-258**

Acute chest pain (resting image only)	Class of recommendation	Level of evidence	Score
Possible ACS – normal or uninterpretable ECG*; low-risk TIMI score; borderline, minimally elevated, or negative cardiac markers	IIa	A	8
Possible ACS – normal or uninterpretable ECG*; high-risk TIMI score; borderline, minimally elevated, or negative cardiac markers	IIa	A	7/8
Possible ACS – normal or uninterpretable ECG*; negative initial cardiac markers. Recent (up to 2 hours) or evolving chest pain	IIa	B	7
**Post-ACS assessment (infarction with or without ST-segment elevation)**	**Class of recommendation**	**Level of evidence**	**Score**
Stable, post-STEMI patients, for assessment of ischemia/myocardial viability; cardiac catheterization not performed	IIa	B	8
Stable, post-NSTEMI patients, for assessment of ischemia/myocardial viability; cardiac catheterization not performed	IIa	B	9
*LBBB: left bundle branch block; CAD: coronary artery disease; ECG: 12-lead electrocardiogram; STEMI: ST-elevation myocardial infarction; NSTEMI: non-ST-elevation myocardial infarction; ACS: acute coronary syndrome. Normal ECG: no ischemic or necrotic changes. Uninterpretable ECG: BBB, pacemaker rhythm, WPW syndrome, significant left ventricular hypertrophy.*			

**Table 17:** – Indication criteria for myocardial perfusion imaging for assessment of myocardial viability **1,9,31,128,258,259**

Assessment of myocardial viability	Class of recommendation	Level of evidence	Score
Marked left ventricular dysfunction, eligible for myocardial revascularization	I	A	9

**Table 18:** – Advantages, disadvantages, and contraindications of the different stress testing modalities **8,260,264**

	Supine cycle ergometer	Conventional cycle ergometer	Treadmill	Dobutamine
Increases myocardial oxygen demand	Yes	Yes	Yes	Yes
Allows assessment during the stress phase	Yes	Yes	No	Yes
Allows imaging at peak stress	Yes	Yes	No*	Yes
Provides adequate assessment of CVD severity	Yes	Yes	Yes	Yes
Allows diagnostic evaluation of ischemia	Yes	Yes	Yes	Yes
Allows assessment of cardiorespiratory fitness	Yes	Yes	Yes – best	No
Allows assessment of functional impact	Yes	Yes	Yes	No
Risk of complications	Very low	Low	Low	Low
Usefulness to define prognosis	Yes	Yes	Yes	Limited
Availability	Moderate	Low	High	High
Contraindications	1) Unstable or complicated acute coronary syndrome** 2) Serious cardiac arrhythmias (VT, complete AV block)** 3) Moderate-to-severe hypertension (Resting SBP >180 mmHg)** 4) Echo abnormalities that might make stress unsafe** 5) Absolute contraindications to ET (see Box 1)			Same (1 through 4) and 5) Significant LV outflow tract obstruction
CVD: cardiovascular diseases; VT: ventricular tachycardia; AV: atrioventricular; SBP: systolic blood pressure; LV: left ventricle. Echo: baseline echocardiogram. *Image acquisition is done immediately (as soon as possible) after exertion. **Contraindications to both physical exercise and dobutamine administration.				

**Table 19:** – Indications for stress echocardiography in acute coronary syndrome (exclusively in chest pain units or inpatient hospital settings) **8,260,261,265**

Indication	Class of recommendation	Level of evidence
Patients with clinically controlled, low-risk unstable angina* before deciding on an invasive strategy	IIa	A
To assess the functional significance of moderate coronary obstruction on angiography, as long as the result would change management	IIa	C
Risk stratification after uncomplicated myocardial infarction	IIa	A
Investigation of patients with suspected microvascular disease** to establish if there are echocardiographic wall motion abnormalities simultaneous to angina and ECG changes	IIa	C
Speckle tracking-derived strain and strain rate parameters as an adjunct to the wall motion score index for diagnosis and/or prognosis of acute coronary syndromes^266^	IIa	B
High-risk unstable angina or in the acute stage of myocardial infarction	III	C
*No recurrence of angina, no signs of heart failure, no changes in baseline/serial ECG, and normal troponin. **Typical anginal pain with abnormal ECG or functional test but normal coronary angiography.		

**Table 20:** – Indications for stress echocardiography in patients with suspected or known coronary artery disease **8,260,265**

Indication	Class of recommendation	Level of evidence
In patients with low or intermediate pretest probability of CAD unable to undergo ET and/or with uninterpretable ECG	I	B
Further investigation after nondiagnostic ET	I	B
Further investigation after finding of calcium score (Agatston) >400 on coronary CT^254^	I	B
Further investigation after finding of intermediate lesions on coronary angiography	I	B
Assessment of myocardial viability in patients with ventricular dysfunction eligible for revascularization	I	B
Preoperative assessment for noncardiac surgery in patients at intermediate and high risk according to risk scores*	I	B
Preoperative assessment of intermediate-risk noncardiac surgery in patients with one or more risk factors and/or low functional class (<4 METs)	IIa	B
Patients symptomatic after cardiac revascularization	IIa	B
Patients asymptomatic after incomplete revascularization	IIa	C
Microbubble contrast-enhanced echocardiography: as an adjunct to stress modalities in the investigation of ischemia and assessment of myocardial viability	IIa	B
Preoperative assessment of intermediate-risk noncardiac surgery in patients with functional class ≥4 METs	III	B
Initial or routine replacement of ET in patients capable of physical exertion conditions with an interpretable ECG	III	C
MET: metabolic equivalent of task; CAD: chronic coronary artery disease; ET: exercise test; LVEF: left ventricular ejection fraction; ECG: electrocardiogram; FC: functional class. *Risk scores associated with cardiovascular outcomes: Lee’s Revised Cardiac Risk Index (RCRI); American College of Physicians (ACP) Index; Multicenter Study of Perioperative Evaluation for Noncardiac Surgeries in Brazil (EMAPO) method.^*52,267-269*^		

**Table 21:** – Indications for stress echocardiography in patients with nonischemic CVD

Indication	Class of recommendation	Level of evidence
**Mitral stenosis:** in cases of discrepancy between symptoms and valve area/gradient (mitral area >1.5 cm^2^)^8,93,265,270^	I	C
**Mitral stenosis:** in asymptomatic patients with mitral area <1 cm^2 8,93,265,270^	IIa	C
**Mitral stenosis:** in asymptomatic patients with mitral area 1-1.5 cm^2^planning pregnancy or scheduled to undergo major surgery^8,93,265,270^	IIb	C
**Mitral regurgitation:** in cases of discrepancy between symptoms and severity of valvular disease^8,93,265,270^	IIa	B
**Mitral regurgitation:** in severe asymptomatic cases, to assess exercise tolerance and hemodynamic compromise^8,93,265,270^	IIa	B
**Mitral insufficiency:** to assess left ventricular reserve^8,93,265,270^	IIb	B
**Aortic stenosis:** in moderate or severe (stages B and C1), asymptomatic cases, to assess exercise-induced symptoms, BP or pulmonary arterial pressure responses, gradient behavior, and left ventricular function^8,93,265^	IIa	B
**Aortic stenosis:** in asymptomatic or mild/questionable symptoms, with low flow/gradient and preserved LVEF, to differentiate true stenosis from pseudostenosis^8,93,265^	IIb	B
**Aortic stenosis:** exercise or dobutamine stress echocardiography in severe symptomatic AS^8,93,265^	III	C
**Aortic regurgitation:** in severe, asymptomatic cases (or with questionable symptoms), to assess exercise-induced symptoms and functional capacity^8,93,265^	IIa	B
**Aortic regurgitation:** in moderate cases, to elucidate symptoms and rule out other causes^8,93,265^	IIa	B
**Aortic regurgitation:** exercise or dobutamine stress echocardiography, to quantify AR in cases of discrepancy between symptoms and lesion severity^8,93,265^	III	C
**Prosthetic aortic or mitral valve:** assessment of symptoms, confirmation of hemodynamically significant stenosis and/or patient-prosthesis mismatch, when the transprosthetic gradient at rest is mild to moderate (aortic position, 20-40 mmHg; mitral position, 5-10 mmHg)^265,271^	IIa	B
**Hypertrophic cardiomyopathy:** in symptomatic patients with an intraventricular gradient (at rest or Valsalva-provoked) <50 mmHg, to assess the degree of dynamic obstruction and mitral regurgitation during exertion^8,121,272^	I	B
**Hypertrophic cardiomyopathy:** in asymptomatic patients without dynamic obstruction at rest, when a relevant LVOT gradient is detected; for guidance on lifestyle changes, occupational changes, and to inform treatment decisions^8,121,272^	IIb	C
**Heart failure:** for etiological diagnosis of dyspnea, to guide and monitor response to treatment, clinical deterioration, risk stratification and assessment of contractile reserve^265,273,274^	IIa	B
**In athletes:** on clinical suspicion or symptoms (dizziness or syncope) of dynamic obstruction with development of an intraventricular systolic pressure gradient^265,275,276^	IIb	B
*AR: aortic regurgitation; LVEF: left ventricular ejection fraction; LVOT: left ventricular outflow tract.*		

**Table 22:** – Recommended drug washout period before ET for diagnosis of CAD **4,6,13,134**

Medication	Washout period
**Amiodarone**	30 days
Beta-blockers*	4 days (cardioselective), 7 days (other)
**Calcium channel blockers**	4 days
**Other antiarrhythmics**	3-5 days
**Digoxin**	7 days
**ACE inhibitors:**	
• Captopril, enalapril	1 day
• Other	3 days
**ARBs**	3 days
Loop diuretics**	3 days
**Nitrates**	1 day
**Trimetazidine**	2 days
**Methyldopa/clonidine**	1 day
**Minoxidil**	2 days
ACE: angiotensin converting enzyme; ARB: angiotensin receptor blocker. *Beta-blockers and antihypertensive drugs should be withdrawn gradually to avoid rebound hypertension. **Consider discontinuation judiciously in case of heart failure.	

**Table 23:** – Recommendations regarding targeted history and physical examination **13,115,293**

History	Physical examination
Current symptoms/history of present illness (if any)	General condition (anemia, syndromic facies, pallor)
Family history and risk factors	Heart rate/blood pressure
Past medical history	Heart and lung auscultation
Current medications	Pulse oximetry*
Exercise tolerance	Peripheral pulses and ankle-brachial index**
Inquire if the patient has undergone ET before If so, whether any abnormality was identified	Further examination targeted at any symptoms***
Pre-test clinical score	
*Additional exam to the ET. Recommended in CHF, valvular heart disease, cardiomyopathies and post-COVID patients. **Additional exam to the ET in case of peripheral artery disease and claudication. ***Examples: carotid artery auscultation in older adults with suspected syncope, BP measurement in all four limbs in cases of coarctation of the aorta, etc.	

**Table 24:** – Cycle ergometer protocols

Protocol	Indications	Initial load	Load increase
Balke	Young adults	25W to 50W*	25W/2 minutes
Åstrand	Adults	25W	25W/3 minutes
Jones	Sedentary subjects, older adults	25W	15W/1 minute
Mellorowicz	Well-conditioned subjects or athletes	50W	50W/2 minutes
Ramp	All populations; ideal for athletes**	10W to 50W***	5 to 50W/1 min. Subdivide increment into equal amounts and increase at regular intervals (<60 seconds)****
*In young, healthy individuals, the recommended starting load is 50W; in subjects with physical limitations, zero load; in all others, 25W. **Adjustable to the subject’s expected physical performance and activities of daily living. ***In athletes, the recommended starting load is at least 50W; in subjects with physical limitations, 10W; in all others, 25W. ****Example: ramp protocol with increment of 15 W/minute = increase load by 5W every 20 seconds.			

**Table 25:** – Most common graded exercise protocols for treadmill ET and their characteristics **6,13,134,293**

	Bruce				Modified Bruce/Sheffield				Ellestad				Naugthon			
Stage	Min	mph/km/h	% grade	METs	Min	mph/km/h	% grade	METs	Min	mph/km/h	% grade	METs	Min	mph/km/h	% grade	METs
01	3	1.7/2.7	10	4.6	3	1.7/2.7	0	1.7	3	1.7/2.7	10	4.6	2	1.0/1.6	0	1.5
02	6	2.5/4.0	12	7.1	6	1.7/2.7	5	2.9	5	3.0/4.8	10	7.4	4	2.0/3.2	0	2.0
03	9	3.4/5.5	14	9.6	9	1.7/2.7	10	4.1	7	4.0/6.4	10	9.6	6	2.0/3.2	3.5	3.0
04	12	4.2/6.7	16	12.0	12	2.5/4.0	12	6.7	10	5.0/8.0	10	12.0	8	2.0/3.2	7.0	4.0
05	15	5.0/8.0	18	14.5	15	3.4/5.5	14	10.0	12	6.0/9.7	15	14.5	10	2.0/3.2	10.5	5.0
06	18	5.5/8.8	20	16.8	18	4.2/6.7	16	13.5	14	7.0/9.6	15	17.0	12	2.0/3.2	14.0	6.0
07	21	6.0/9.7	22	19.3	21	5.0/8.0	18	17.5	16	8.0/11.2	15	19.0	14	2.0/3.2	17.5	7.0
08	24	6.5/10.5	24	22.4	24	5.5/8.8	20	20.0	18	9.0/12.8	15	21.5	16	2.0/3.2	21.0	8.0
*Min: minute; mph: miles per hour; km/h: kilometers per hour; MET: metabolic equivalent of task.*																

**Table 26:** – Diamond-Forrester/CASS risk estimation score for pretest probability of obstructive CAD according to angina classification **321**

Age (years)	No angina		Atypical angina		Typical angina	
	Men	Women	Men	Women	Men	Women
30 to 39	4	2	34	12	76	26
40 to 49	13	3	51	22	87	55
50 to 59	20	7	65	31	93	73
60 to 69	27	14	72	51	94	86

**Table 27:** – Duke database risk estimation score for pretest probability of obstructive CAD according to angina classification **322**

Age (years)	No angina		Atypical angina		Typical angina	
	Men	Women	Men	Women	Men	Women
35	3 to 35	1 to 19	8 to 59	2 to 39	30 to 88	10 to 78
45	9 to 47	2 to 22	21 to 70	5 to 43	51 to 92	20 to 79
55	23 to 59	4 to 21	45 to 79	10 to 47	80 to 95	38 to 82
65	49 to 69	9 to 29	71 to 86	20 to 51	93 to 97	56 to 84
*The range shows the probability of CAD from low-risk patients (no diabetes, smoking, or dyslipidemia) to high-risk patients.*						

**Table 28:** – European database risk estimation score for pretest probability of obstructive CAD in symptomatic patients **45**

Age (years)	No angina		Atypical angina		Typical angina	
	Men	Women	Men	Women	Men	Women
30 to 39	18	5	29	10	59	28
40 to 49	25	8	38	14	69	37
50 to 59	34	12	49	20	77	47
60 to 69	44	17	59	28	84	58
70 to 79	54	24	69	37	89	68
>80	65	32	78	47	93	76
*Estimated probability of CAD for patients aged 35, 45, 55, 65, 75, and 85 years.*						

**Table 29:** – Factors that affect HRmax in response to dynamic exercise

Factors that affect HRmax	

Age	Cardiovascular disease
Gender	Drugs
Body weight	Pacemaker/ICD
Prolonged rest	Arrhythmias/atrial fibrillation/flutter
Type of exercise	Anemia
Intensity of exertion achieved	Hypo– and hyperthyroidism
*ICD: implantable cardioverter/defibrillator.*	

**Table 30:** – Variables measuring the HR response to ET

Index	Calculation	Normal range
**Resting HR^**371**^**	Measured on resting ECG (seated or supine)	50 to 99 bpm
**Estimated HRmax for age^**300-302**^**	– HRmax prediction equations for both sexes:** HRmax = 220 – age HRmax = 208 – (0.7 × age) – Sex-specific HRmax prediction equations:** HRmax for women = 192 – (0.7 × age) HRmax for men = 201 – (0.6 × age)	≥85% of estimated HRmax
**Measured chronotropic reserve^**372,373**^**	HRR = maximum HR reached – RHR	Serial measurement
**Predicted chronotropic reserve for age**	HRR for age = estimated HRmax for age – RHR	Serial measurement
**Chronotropic index (%)**	CI = (HRmax – RHR) × 100 (estimated HRmax – RHR)	≥80%
HR in 1st minute of active recovery*	HRmax – HRrec in 1st minute	>12 bpm
**HR in 1st minute of passive recovery (supine)**	HRmax – HRrec in 1st minute	>18 bpm
**HR in 2nd minute of passive recovery (seated)**	HRmax – HRrec in 2nd minute	≥22 bpm
HR: heart rate; HRmax: maximum heart rate; CI: chronotropic index; HRR: chronotropic reserve; RHR: resting heart rate. *Active recovery (treadmill): walking at 1.5 mph (2.4 km/h) and 2.5% grade. **Age in years.		

**Table 31:** – Definitions, criteria, and interpretation of the HR response to ET

Term	Criteria*	Interpretation
**HR behavior on baseline ECG**		
**Normal HR behavior**	HR ranging from 50 to 99 bpm on resting ECG (seated or supine)	Adult in sinus rhythm.
**Sinus bradycardia at rest**	HR <50 bpm on resting ECG (seated or supine)	Common in athletes and asymptomatic young adults with increased vagal tone. If secondary to beta-blockers or antiarrhythmics, mention in report. In patients not on negative inotropic medications, evaluate the possibility of sinus node dysfunction or other secondary causes. Rule out second-degree and high-grade AV block.
**Sinus tachycardia at rest**	HR ≥100 bpm on resting ECG (seated or supine)	Usually found in obese patients, those with severe anxiety, hyperthyroidism, anemia, or after excess caffeine or alcohol intake.
**HR behavior on exertion**		
**Normal chronotropic response**	≥85% of estimated HRmax reached between 8 and 12 minutes of exercise	When in sinus rhythm.
**Accelerated chronotropic response**	1) Rapid, early rise in HR, disproportionate to the workload, reaching ≥85% of the predicted HRmax or a ≥50% increase in resting HR at 3.5 METs, or 2) ≥17 bpm increase in the first minute of exercise in normal subjects, or 3) ≥15 bpm increase in the first minute of exercise in patients with CAD.	Usually found in sedentary subjects, patients with severe anxiety, neurovegetative dystonia, hyperthyroidism, conditions that reduce vascular volume or peripheral resistance, anemia, metabolic derangements, ET performed soon after myocardial infarction and/or CABG, etc.^374^
**HR drop during exercise**	HR decrease (>10 bpm) as exercise progresses	Although rare, correlates strongly with ischemic heart disease.^152^
**Chronotropic reserve**	This parameter should be evaluated by serial ET. The greater the reserve, the better the functional status, vagal tone/autonomic modulation, and cardiovascular health.	Reduction in HRR is a risk factor for CV mortality.^375^Each 1 bpm increment in HRR reduces the incidence of SCD by 1-2% and the incidence of type 2 diabetes by 2-3%.^373,376^
**Impaired chronotropic response or chronotropic incompetence**	1) HR achieved on exertion <2 standard deviations from predicted HRmax, or 2) Failure to achieve 85% of predicted HR for age, or 3) Chronotropic index <0.80	Associated with decreased vagal activity and increased risk of cardiovascular and all-cause mortality.^149,151,377,378^
**HR plateau during exercise**	HR unchanged even as exercise progresses	In women and children, has no clinical significance. May occur in patients with CAD.^134,152^
**HR response during recovery**		
**Normal HR response to recovery**	1) HR >12 bpm in 1st minute of active recovery, or 2) HR >18 bpm in 1st minute of passive recovery (supine), 3) HR ≥22 bpm in 2nd minute of passive recovery (seated)	When in sinus rhythm.
**Slow HR recovery post-exercise**	1) HR decrease ≤12 bpm in 1st minute of active recovery, or 2) HR decrease ≤18 bpm in 1st minute of passive recovery (supine), or 3) HR decrease ≤21 bpm in 2nd minute of passive recovery (seated)	Abnormal slow HR recovery after exercise is associated with increased cardiovascular and all-cause mortality.^38^
**Sudden, sharp drop in HR during recovery**	Abrupt drop in HR at any point during recovery.	Common finding in physically fit individuals, including athletes, as long as it is asymptomatic.^13^
HR: heart rate; HRmax: maximal heart rate; SCD: sudden cardiac death; HRR: chronotropic reserve; CAD: coronary artery disease; CV: cardiovascular; AV: atrioventricular. *Describe the use of medications that may affect HR response.		

**Table 32:** – Blood pressure response to ET in adults

Term	Criteria	Interpretation
**Normal BP response to exercise and recovery**	1) Normal resting BP: SBP <140 mmHg and DBP <90 mmHg; and 2) During exercise: SBP <210 mmHg for men and <190 mmHg for women; DBP unchanged or fluctuating up to ±10 mmHg; and 3) Normal BP during recovery: gradual drop in SBP to resting value or just below it. DBP may rise slightly at the beginning of recovery or remain unchanged; by 6 minutes, it tends to return to resting values.	Normotension at rest, during exercise and recovery. In the absence of other ET abnormalities, good prognosis and low risk.^6,215^
**Pretest hypertension with normal BP response to exercise**	1) High resting BP: SBP ≥140 mmHg and/or DBP ≥90 mmHg; and 2) During exercise: SBP <210 mmHg for men and <190 mmHg for women; DBP unchanged or fluctuating up to ±10 mmHg; and 3) Recovery: normal.	Usually due to anxiety; not associated with future development of hypertension.^6,215^
**Hypertensive response to exercise**	1) Resting BP may be normal or high (SBP ≥140 mmHg and/or DBP ≥90 mmHg); and 2) During exercise: SBP ≥210 mmHg for men and ≥190 mmHg for women; DBP elevation ≥15 mmHg or DBP >90 mmHg (men and women)**^85,387^Note: Describe SBP and DBP response separately.	Represents perpetuation/worsening of hypertension on exertion. In patients who are normotensive at rest, HRE is associated with risk of future hypertension.^71,387-389,406^Systolic HRE is associated with an increased risk of LVH, MI, AF, stroke, and CV death.^74,405,407,408^Diastolic HRE is associated with increased risk of CAD and of hypertension.^73,396^
**Hypotension/drop in BP during exercise**	1) Drop in SBP below resting value without a concomitant drop in DBP (usually associated with ischemia)^409^2) Drop in SBP and DBP below resting values (usually not associated with ischemia, but rather with LV inotropic deficit; i.e. valvular heart disease)^409^3) Initial rise in SBP followed by a drop in SBP ≥20 mmHg***^115,386,410^	Exercise-induced hypotension is a marker of adverse events during ET and poor prognosis, and is thus useful for defining intervention.^403,410^Systolic hypotension is associated with left ventricular dysfunction and reduced cardiac output and is a marker of severe heart disease.^405,409^Approximately one-third of adult HCM patients have on exercise systolic hypotension, caused by an inadequate drop in systemic vascular resistance and low cardiac output reserve. Such hypotension is defined as a drop in SBP >20 mmHg.^410^
Depressed BP response*^4^	1) Systolic pressure reserve (difference between maximal exercise SBP and resting SBP) <35 mmHg in the absence of a marked drop in DBP; or 2) Maximum increase in SBP <140 mmHg; or 3) Plateau SBP response (BP unchanged over 2 or more stages in step protocol or for more than 3 consecutive minutes in ramp protocol) with systolic pressure reserve <35 mmHg^411^	Often associated with severe CAD and worse prognosis.^403^Associated with increased risk of cardiovascular events and all-cause mortality.^412^In HCM, consider depressed pressure response as a failure to increase SBP by at least 20 mmHg from rest to exercise peak.^410^
**Normal recovery BP response**	1) Progressive reduction in SBP. At the onset of recovery, DBP may increase slightly or remain unchanged. SBP and DBP tend to return to resting values by the 6th minute of recovery 2) Ratio of SBP in the 3rd minute of recovery / peak SBP ≤ 0.9 3) BP in the 5th minute of recovery: SBP <160 mmHg and DBP <90 mmHg 4) Absence of hypotension during recovery	In the absence of other ET abnormalities, good prognosis and low risk.^383,384,413,414^
**Paradoxal (increase) BP response in recovery**	Ratio of SBP in the 3rd minute of recovery to SBP in the 1st minute of recovery ≥1^415-417^	Predictive of CAD, MI, stroke, and CV mortality.^387,418,419^
Slow systolic BP response in recovery*^5^	Regardless of BP behavior at rest and during exercise: – SBP in the 3rd minute of recovery/peak SBP >0.9^420^– BP in the 5th minute of recovery: SBP ≥160 mmHg and DBP ≥90 mmHg^421^	Correlates well with future hypertension and CAD^420^
Hypotension during recovery*^5^	Lightheadedness, dizziness, nausea, presyncope, or syncope during recovery, in the presence of: – SBP drop during recovery >50% of SBPmax during exercise; or – SBP <90 mmHg during recovery^422^	Post-exercise hypotension usually occurs in apparently healthy individuals. Although associated with an increased incidence of arrhythmias, it has no association with CV morbidity or mortality, and is most common in young individuals who exercise to exhaustion.^422^
**Diastolic hypertensive response in recovery**	DBP in the 5th minute of recovery ≥90 mmHg.	Predictive of future hypertension, CAD, and stroke^73,421^
BP: blood pressure; ET: exercise test; SBP: systolic blood pressure; DBP: diastolic blood pressure; LVH: left ventricular hypertrophy; LV: left ventricle; HRE: hypertensive response to exercise; CAD: coronary artery disease; AF: atrial fibrillation; CV: cardiovascular; HF: heart failure; MI: myocardial infarction; SBPmax: SBP measured at maximum effort; SBP peak: SBP at peak of effort even not associated with physical exhaustion (maximum effort). *Describe whether the ET response occurred during use of drugs with an antihypertensive effect. **The average increase in SBP is usually 10 mmHg/MET. In individuals who reach a work load >10 METs, the expected SBP increase is 6.2 mmHg/MET. ***Occasionally, individuals without clinically significant cardiac disease will experience exercise-induced hypotension due to dehydration, an inadequate dose of antihypertensive therapy, or prolonged strenuous exercise. If asymptomatic, confirm the drop in BP in at least one more measurement. *^4^In athletes, children and adolescents, and women in the follicular phase, fixed SBP despite progressive exertion is not necessarily pathologic.^*5^For active or passive recovery.		

**Table 33:** – Conditions that interfere with the interpretation of repolarization changes during ET for diagnosis of CAD **4,6**

Invalidate interpretation altogether	Interpretation possible, but with less accuracy
Wolff-Parkinson-White syndrome	Exaggerated atrial repolarization wave*
Variant pre-excitation syndromes	Mitral valve prolapse
Left bundle branch block	Cardiomyopathy, valvular heart disease, pericarditis
Ventricular stimulation by artificial pacemaker	Metabolic disorders, hypokalemia
ST-segment depression ≥1 mm on resting ECG	Anti-ischemic, antiarrhythmic, and beta-blocker therapy
Digitalis therapy	LV hypertrophy on resting ECG
Unsatisfactory technical quality of ECG recording	
*Negative Ta wave which, in the initial period of ventricular repolarization, may cause J-point and ST-segment depression (upsloping, false positive).	

**Table 34:** – Classification of functional capacity based on the FAI **744**

Classification	% of FAI
Surpassed the VO_2_predicted (ideal)	Negative*
No significant impairment	0-26%
Mild impairment	27-40%
Moderate impairment	41-54%
Marked impairment	55-68%
Extreme impairment	>68%
*The higher the negative value, the better the subject’s cardiorespiratory fitness.	

**Table 35:** – Raxwal and Morise score

Variable	Men		Women	
	Data	Score	Data	Score
**HRmax**	<100 bpm	30	<100 bpm	20
	100 to 129 bpm	24	100 to 129 bpm	16
	130 to 159 bpm	18	130 to 159 bpm	12
	160 to 189 bpm	12	160 to 189 bpm	08
	190 to 220 bpm	06	190 to 220 bpm	04
**ST depression**	1-2 mm	15	1-2 mm	06
	>2 mm	25	>2 mm	10
**Exertional angina**	Present	03	Present	09
	Limiting	05	Limiting	15
**Age**	>55 years	20	>65 years	25
	40-55 years	12	50-65 years	15
**Pre-ET angina**	Definite/typical	05	Definite/typical	10
	Likely/atypical	03	Likely/atypical	06
	Noncardiac	01	Noncardiac	02
**Diabetes**	Present	05	Present	10
**Dyslipidemia**	Present	05	Not assessed	--
**Smoking**	Not assessed	--	Present	10
**Estrogenic state**	Not applicable	--	Positive	- 05
			Negative	+ 05
*Estrogenic state: negative = postmenopausal or oophorectomized women without hormone replacement therapy; positive = premenopausal women or postmenopausal/oophorectomized women on hormone replacement therapy.*				

**Table 36:** – Test cessation criteria **6,209,210,762,777**

Parameter	Criteria
**Symptoms**	– Physical exhaustion (Borg scale ≥18) – Lower-limb muscle pain and fatigue – Lower-limb claudication (limiting), ataxia – Persistent (limiting) vertigo, nausea, presyncope, syncope – Increasing chest discomfort or chest pain with increasing work load (limiting), typical angina (moderate to severe) – Early dyspnea disproportionate to the intensity of exertion
**Physical examination/cardiovascular and respiratory variables**	– Pallor (skin and mucous membranes), diaphoresis (profuse, disproportionate sweating), poor peripheral perfusion – Tachypnea (disproportionate to exertion), bronchospasm, bilateral basal crackles; increased crackles in the elderly* – New onset of heart murmur and/or S3 or S4 – Initial rise in SBP followed by a drop in SBP ≥20 mmHg. If asymptomatic, confirm the drop in BP in at least one more measurement – Marked elevation of SBP >250 mmHg – Elevation of DBP ≥120 mmHg in normotensive individuals or ≥140 mmHg in hypertensive patients – Normal fingertip pulse oximetry at baseline followed by desaturation (SpO_2_≤92%)
**ECG findings**	– ST-segment changes: depression ≥0.3 mV (3.0 mm) in addition to resting values; elevation ≥0.2 mV (2.0 mm) in leads without evidence of previous infarction – Nonsustained supraventricular tachyarrhythmia, symptomatic or with hemodynamic repercussions – Sustained supraventricular tachyarrhythmia, asymptomatic or without hemodynamic repercussions: individualize the number of beats and repetitions, considering the indication for ET and underlying diseases – Sustained supraventricular tachyarrhythmia (≥30 seconds) – Exercise-induced atrial fibrillation or flutter – Nonsustained ventricular tachycardia (≥3 beats/<30 seconds)** or any episode of polymorphic NSVT – Sustained ventricular tachycardia (≥30 seconds) – Ventricular fibrillation – Exercise-induced 2nd and 3rd degree AV block – Exercise-induced left bundle branch block (symptomatic or with hemodynamic repercussions)*** – New bundle branch block which cannot be distinguished from ventricular tachycardia – Patients with ICDs (terminate test at 10 bpm below the defibrillator firing threshold) – Persistent drop in HR with increasing load
**Others**	– At the patient’s request, regardless of the occurrence of any abnormal findings – Failure or malfunction of the ECG monitoring/recording system – Failure to adapt to and/or coordinate with the chosen ergometer
SBP: systolic blood pressure; DBP: diastolic blood pressure; AV: atrioventricular; ICD: implantable cardioverter/defibrillator; HR: heart rate; NSVT: nonsustained ventricular tachycardia. *In asymptomatic older adults, the presence of pulmonary crackles on baseline physical examination is common; age is the only independent predictor. **In case of a single episode of NSVT, individualize the number of beats for test cessation, taking into account the indication for ET, underlying condition, clinical findings during ET, and setting where the ET is being performed (hospital or other). ***If asymptomatic or without hemodynamic repercussion, individualize the decision to continue or terminate at the discretion of the performing physician.	

**Table 37:** – Indications for post-exercise ABI measurement as part of a treadmill test

Indication	Class of recommendation	Level of evidence
Patients with exertional lower-extremity symptoms* and normal or borderline ABI at rest (>0.90 and ≤1.40), for diagnosis and risk stratification^781,788-791^	I	B
Patients with PAD and abnormal ABI at rest (≤0.90), for assessment of functional status and prognostic stratification^781,787,792,793^	IIa	B
Asymptomatic diabetic patients with abnormal ABI at rest (≤0.90), for assessment of functional status and prognostic stratification^785^	IIb	B
Combined with transcutaneous oxygen pressure measurement during exercise, to investigate lower-limb arterial stenosis^794,795^	IIb	B
*Non-articular symptoms.		

**Table 38:** – Indications for performing pulse oximetry during ET **219,811**

Indication	Class of recommendation	Level of evidence
Children and adolescents with congenital heart disease (corrected and uncorrected), cardiomyopathies, heart failure, valvular heart disease^812,813^	I	B
Pediatric and adult patients undergoing investigation of a complaint or differential diagnosis of dyspnea*	IIa	B
Adult patients with asymptomatic valvular heart disease or cardiomyopathy	IIb	B
Pediatric and adult patients after a respiratory infection with potential capacity to compromise lung function (i.e. post-COVID condition)^228^	IIb	B
Pediatric and adult patients after cardiac surgery (i.e. valve replacement, CABG)	IIb	B
Adult patients with heart failure or congenital heart disease^814^	IIb	B
*When there is no formal indication for CPET or CPET is not available. CABG: coronary artery bypass grafting.		

**Table 39:** – Causes of unreliable SpO **2** measurements and underlying mechanisms **817-819**

1. Causes of intermittent oscillation or inability to measure SpO_**2**_
• Poor peripheral perfusion (i.e. hypovolemia, vasoconstriction, etc.)
• Tremor (Parkinson’s disease) and edema
**2. Causes of falsely normal or elevated SpO_**2**_**
• Carbon monoxide poisoning
• Vaso-occlusive crisis of sickle cell anemia
**3. Causes of falsely low SpO_**2**_**
• Venous pulsations (arteriovenous fistula)
• Excess movement
• Hereditary forms of abnormal hemoglobin
• Severe anemia (with concomitant hypoxemia)
• Nail polish
**4. Causes of falsely low or high SpO_**2**_**
• Methemoglobinemia
• Sulfhemoglobinemia
• Sepsis and septic shock
**5. Causes of falsely low CO_**2**_Hb as measured on CO-oximetry**
• Severe hyperbilirubinemia
• Fetal hemoglobin (HbF)

**Table 40:** – Indications for lactate measurement and arterial blood gas analysis in combination with ET/CPET

Indication	Class of recommendation	Level of evidence
**Lactate measurement**		
Serial measurement in competitive athletes engaged in predominantly aerobic activities, for adjustment of training load and intensity^840,841,855-857^	IIa	B
Medical clearance/exercise prescription and/or adjustment in the rehabilitation setting^831,832,855,858^	IIb	A
Investigation of overtraining syndrome^859,860^	IIb	B
Evaluation of cellular metabolism in patients with comorbidities (i.e. CAD, COPD, chronic renal failure, multiple sclerosis) and risk stratification*^831,832,855,858^	IIb	B
**Arterial blood gases**		
Investigation of dyspnea/hypoxemia and suspected desaturation^861,862^	IIb	B
Suspected ventilation/perfusion mismatch (i.e. heart failure, COPD)*^850,851,853,863^	IIb	B
In COPD, to determine the physiologic dead space, dynamic hyperinflation, and their interrelationships, for diagnostic and prognostic purposes*^854,864^	IIb	B
HF: heart failure; COPD: chronic obstructive pulmonary disease; CAD: coronary artery disease. *Usually in combination with CPET.		

**Table 41:** – Causes of lower extremity pain and exertional claudication unrelated to PAD

Condition	Location	Characteristic	During exercise	During recovery	Particular features
**Venous claudication**	Whole leg, more pronounced in the calf	Sharp, sudden pain	After walking	Resolves slowly	Relieved by elevation History of iliofemoral DVT; edema; signs of venous stasis
**Chronic compartment syndrome**	Calf muscles	Crushing, explosive pain	After intense exertion	Resolves very slowly	Relieved by rest Most common in athletes with large muscle mass
**Spinal stenosis**	Often bilateral buttocks, posterior leg	Pain and weakness	May mimic claudication	Variable relief, may take time to resolve	Relieved by flexion of the lumbar spine Worse when standing
**Disc herniation/ nerve root compression**	Radiates down leg	Sharp, acute pain	Triggered by sitting, standing, or walking	Often present at rest	May improve by changing position Frequent history of low back pain
**Foot/ankle arthritis**	Ankle, foot, arch of foot	Deep-seated pain	Onset with exertion (regardless of intensity)	Slow relief	Variable symptoms at rest and/or with exertion
*DVT: deep vein thrombosis. Adapted from: Gerhard-Herman MD et al.^*781*^*					

**Table 42:** – Reference values of core spirometry parameters for the adult Brazilian population, stratified by ethnicity

	White		Black	
Variable	Men	Women	Men	Women
FVC (L)	4.64±0.77	4.42±0.78	3.14±0.65	3.10±0.52
FEV1 (L)	3.77±0.67	2.56±0.57	3.55±0.69	2.55±0.48
FEV1/FVC (%)	81.0±5	81.0±5	80.3±5.4	82.0±5.4
FEF25-75 (L/s)	3.87±1.20	2.70±0.94	3.54±1.17	2.77±0.93
FEF50 (L/s)	4.82±1.44	3.40±1.14	4.39±1.36	3.54±1.06
FEF75 (L/s)	1.58±0.64	1.07±0.52	1.43±0.63	1.11±0.52
PEF (L/s)	11.1±1.75	7.14±1.28	9.77±2.07	6.73±1.28
*All values given as mean ± standard deviation. FVC: forced vital capacity; FEV1: forced expiratory volume in one second; FEF25-75: forced expiratory flow between 25% and 75% of FVC; FEF50: forced expiratory flow at 50% of FVC; FEF75: forced expiratory flow at 75% of FVC; PEF: peak expiratory flow rate. Adapted from Pereira CAC et al.^*1015*^“Novos valores de referência para espirometria forçada em brasileiros adultos de raça branca.” and Prata TA et al.^*1016*^“Valores de referência para espirometria forçada em adultos negros no Brasil.”*				

**Table 43:** – Reference values for the CPET variables VO **2** peak, oxygen pulse, and VE/VCO **2** curve

Variable	Age	Men	Women
**VO_**2**_peak (mL/min)**	20-29	3.250-2.970	2.000-1.840
	30-39	2.950-2.690	1.820-1.660
	40-49	2.670-2.400	1.640-1.490
	50-59	2.380-2.130	1.470-1.320
	60-69	2.110-1.840	1.300-1.140
	70-80	1.820-1.570	1.120-0.940
**Oxygen pulse (mL/bpm)**	20-29	16.2-15.6	10.0-9.6
	30-39	15.5-14.9	9.6-9.2
	40-49	14.8-14.1	9.1-8.7
	50-59	14.0-13.2	8.6-8.2
	60-69	13.1-12.2	8.1-7.5
	70-80	12.1-11.1	7.4-6.7
**VE/VCO_**2**_slope**	20-39	23.4-25.7	26.8-28.3
	40-59	25.8-28.1	28.4-29.9
	60-80	28.2-30.6	30.0-31.6
*Adapted from Mezzani A. et al.^*229*^“Cardiopulmonary Exercise Testing: Basics of Methodology and Measurements”.*			

**Table 44:** – Reference values and interpretation of key CPET variables

Variable	Normal	Abnormal		
		Mild	Moderate	Severe
**% predicted VO_**2**_peak**	≥100%	75-99%	50-74%	<50%
**% predicted VO_**2**_peak at VT1**	40-80%	<40%		
**VE/VCO_**2**_slope**	<30	30-35.9	36-45	>45
**Oxygen pulse during exercise**	Increase	Early flattening or decrease*		
**Respiratory reserve**	≥30%	<30%**		
**EOV**	Absent	Present		
**VO_**2**_VT1**	≥40% predicted VO_2_peak or 40-60% actual VO_2_peak	<40% predicted VO_2_peak or actual VO_2_peak		
**PETCO_**2**_VT1**	3 to 8 mmHg increase from resting value	<3 or >8 mmHg increase from resting value		
EOV: exercise oscillatory ventilation; VCO_*2*_: CO_*2*_production; VE: minute ventilation; VO_*2*_: oxygen consumption; VT1: first ventilatory threshold. PETCO_*2*_: end-tidal partial pressure of carbon dioxide. *Plateau before VO_*2*_max. **In case of lung disease. Adapted from: Marcadet DM et al.^*4*^“French Society of Cardiology guidelines on exercise tests (part 1): Methods and interpretation” and Mezzani A, et al.^*229*^“Cardiopulmonary Exercise Testing: Basics of Methodology and Measurements”.^*229*^				

**Table 45:** – Changes in CPET variables commonly seen in in patients with HF **223,233,849,850,1034,1057-1059**

Variable	Change	Usefulness	Interpretation
**VO_**2**_peak**	↓	General assessment of performance on exertion. Prognostic significance; marker of mortality	1) Weber’s classification 2) Heart transplant indicated if <12.0 mL/kg/min (on beta-blockers) or <14.0 mL/kg/min (if intolerant to beta-blockers)
**VO_**2**_VT1**	↓	General assessment/ ↓ cardiac output	Assessment of severity/prognosis
**OP**	↓	Cardiac output/LV function	– Direct relationship with stroke volume. – Plateauing and/or descending OP = LV dysfunction
**VE/VCO_**2**_slope**	↑	Ventilation-perfusion mismatch/increased sympathetic tone	– Predictor of mortality and hospitalization – The greater the slope, the worse the prognosis
**VE/VCO_**2**_slope**	↑	Prognostic marker/indicative of comorbid lung disease	A ratio >34 is an independent predictor of worse prognosis
**ΔVO_**2**_/ΔWR**	↓	↓ Cardiac output/ ↓ O_2_delivery	LV dysfunction: flattening of ∆VO_2_/∆WR curve. Prognostic marker.
**EOV**	Present	↓ Cardiac output/abnormal chemoreflex response	Marker of severity/prognosis, especially if early and cycles last >1 min
PETCO_2_*	↓	↓ Cardiac output/ ↑ chemoreflex response	Reflects disease severity.
**COP**	↑	Variable calculated at submaximal exertion. Reflects cardiorespiratory efficiency	– Predictor of CV and all-cause mortality, alone or combined with other variables – COP ≥36 is associated with increased CV mortality and indicates urgent heart transplantation
**OUES**	↓	Ventilatory efficiency of O_2_consumption	Prognostic significance; indicates risk of events
**VD/DT Ratio**	↓	Increased dead space with exertion	Prognostic marker. Associated with exertional dyspnea
VO_*2*_peak: oxygen consumption at peak exertion; VO_*2*_VT1: oxygen consumption at the first (anaerobic) ventilatory threshold; OP: oxygen pulse; VE/VCO_*2*_slope: ventilatory efficiency (ventilation/CO_*2*_production ratio); ΔVO_*2*_/ΔWR: oxygen consumption to work rate ratio; EOV: exercise oscillatory ventilation; PETCO_*2*_VT1: end-expiratory partial pressure of carbon dioxide at the first ventilatory threshold; COP: cardiorespiratory optimal point; OUES: oxygen uptake efficiency slope; VD/DT ratio: dead space/tidal volume ratio; CV: cardiovascular. *Best assessed at first ventilatory threshold (VT1).			

**Table 46:** – Changes in CPET variables commonly seen in patients with HCM **1057,1074-1077**

Variable	Change	Usefulness	Interpretation
**VO_**2**_peak**	↓	Assessment of exercise performance. Prognostic significance; marker of mortality.	– VO_2_peak <20 mL/kg/min or <80% of predicted has been associated with worse prognosis (heart transplantation and hospitalization for septal reduction) – VO_2_peak <50% of predicted has been associated with all-cause and CV mortality
**VO_**2**_VT1**	↓	General assessment/ ↓ cardiac output	– Mechanisms similar to those involved in the reduction of VO_2_peak – Direct relationship with stroke volume
**OP**	↓	Cardiac output/ strongly related to stroke volume	– Early flattening at around 50% to 60% of maximal exertion load due to the reduction in stroke volume – The earlier the flattening of OP occurs, the greater the severity of HCM
**ΔVO_**2**_/ΔWR**	↓	↓ Cardiac output/VO_2_	– Preserved or slightly reduced in most patients – A reduced slope or slow decline during the last stage of exertion suggests diastolic compromise and/or end-stage disease – Abrupt loss of ΔVO_2_/ΔWR linear relationship indicates comorbid myocardial ischemia
**VE/VCO_**2**_slope**	↑	Prognostic value/marker of mortality	– A ratio >34 is predictive of all-cause mortality and heart transplantation
*VO_*2*_peak: oxygen consumption at peak exertion; VO_*2*_VT1: oxygen consumption at the first (anaerobic) ventilatory threshold; OP: oxygen pulse; ΔVO_*2*_/ΔWR: oxygen consumption to work rate ratio; VE/VCO_*2*_slope: ventilatory efficiency (ventilation/CO_*2*_production ratio).*			

**Table 47:** – Behavior of key CPET variables in patients with pulmonary vascular diseases **246,1095-1098**

Variable	Pulmonary arterial hypertension	Chronic thromboembolic pulmonary hypertension	Pulmonary veno-occlusive disease
**VO_**2**_peak**	↓	↓	↓
**VO_**2**_VT1**	↓	↓	↓ ↓
**OP**	↓	↓	↓
**VE/VCO_**2**_slope**	↑	↑ ↑	↑ ↑
**ΔVO_**2**_/ΔWR**	↓	↓	↓
**PETCO_**2**_**	↑	↑ ↑	↑ ↑
**SaO_**2**_**	↓*	↓ ↓	↓ ↓
**VD/DT Ratio**	↑**	↑ ↑	↑ ↑
VO_*2*_peak: oxygen consumption at peak exertion; VO_*2*_VT1: oxygen consumption in the first (anaerobic) ventilatory threshold; OP: oxygen pulse; VE/VCO_*2*_slope: ventilatory efficiency (ventilation/CO_*2*_production ratio); ΔVO_*2*_/ΔWR: oxygen consumption to work rate ratio; EOV: exercise oscillatory ventilation; PETCO_*2*_: end-expiratory partial pressure of carbon dioxide; COP: cardiorespiratory optimal point; OUES: oxygen uptake efficiency slope; VD/DT ratio: dead space/tidal volume ratio; SaO_*2*_: arterial oxygen saturation. *Drop >3% without increase in PaCO_*2*_(partial pressure of carbon dioxide in arterial blood). **Increase >30% during exertion.			

**Table 48:** – Use of CPET variables for risk assessment in patients with PAH **246,1095,1097,1099**

Variable	Behavior regarding PAH progression	Low risk (<5%)	Intermediate risk (5-10%)	High risk (>10%)
**Functional class**	↓	I and II	III	IV
**VO_**2**_peak (mL/kg/min)**	↓	>15	11-15	<11
**% predicted VO_**2**_**	↓	>65	65-35	<35
**VE/VCO_**2**_slope**	↑	<36	36-45	>45
*VO_*2*_peak: oxygen consumption at peak exertion; % predicted VO_*2*_= percentage actually achieved of predicted oxygen consumption; VE/VCO_*2*_slope = ventilatory efficiency (ventilation/CO_*2*_production ratio).*				

**Table 49:** – Differential diagnosis of the main causes of dyspnea during CPET **220,222,1018,1108**

Variable	Cardiac	Pulmonary	Vascular/pulmonary	Hyperventilation
**VO**_**2**_ **peak**	Decreased	Decreased	Decreased	Normal
**VT1**	Early	Normal	Early	Normal
**RQ**	Normal	Decreased	Normal/reduced	Normal/reduced
**VE/VCO**_**2**_ **slope**	Increased	Increased	Increased	Increased
**OP**	Reduced/plateau	Normal	Decreased	Normal
**SaO**_**2**_	Normal	Desaturation	Desaturation	Normal
**ΔVO**_**2**_ **/ΔWR**	Decreased	Normal	Decreased	Normal
*VO_*2*_peak: oxygen consumption at peak exertion; VT1 = first (anaerobic) ventilatory threshold; RQ: respiratory quotient; VE/VCO_*2*_slope = ventilatory efficiency (ventilation/CO_*2*_production ratio); OP: oxygen pulse; SaO_*2*_: arterial oxygen saturation measured by pulse oximetry; ΔVO_*2*_/ΔWR: oxygen consumption to work rate ratio.*				

**Figure 1:** – Electrode placement in the classic Mason-Likar lead system and its modified arrangement including lead CM5. RA: right arm; LA: left arm; RL: right leg; LL: left leg.

**Figure 2:** – Electrode placement for the 13-lead system (Mason-Likar + CM5). RA: right arm; LA: left arm; RL: right leg; LL: left leg; CM5: additional bipolar lead (extra electrode).

**Figure 3:** – Definitions of sensitivity, specificity, predictive values, and accuracy. TP: true positive – when the test is positive and the patient actually has the disease; FP: false positive – when the test is positive but a patient does not have the disease; TN: true negative – when the test is negative and the patient does not have the disease; FN: false negative – when the test is negative but the patient actually has the disease.

**Figure 4:** – Borg and Modified Borg Rating of Perceived Exertion scales.

**Figure 5:** – Mechanisms and factors associated with the emergence and progression of exercise intolerance and the role of ET. CAD: coronary artery disease; HF: heart failure; AF: atrial fibrillation; PAD: peripheral artery disease; ET: exercise test; HR: heart rate; ↓ METs: low cardiorespiratory fitness; ↑ Baseline HR: elevated baseline heart rate; ↓ HR Reserve: reduction of chronotropic reserve; ↓ HRrec: abnormal, slow, post-exercise recovery of HR.

**Figure 6:** – VO 2 /MET values in relation to functional class and clinical status. 6,232 VO 2 max: maximum oxygen consumption; ET: exercise test; MET: metabolic equivalent of task. *New York Heart Association. **Depending on age and level of physical activity.

**Figure 7:** – Scales for quantification of angina, dyspnea, and intermittent claudication.

**Figure 8:** – Heart rate response and autonomic adjustments during ET in adults. HRmax: maximal heart rate; Act.: activity; min: minute.

**Figura 9:** – Systolic and diastolic blood pressure response to ET in adult men and women. SBP: systolic blood pressure; DBP: diastolic blood pressure; min: minute.

**Figure 10:** – Main blood pressure responses to ET and their repercussions. HTN: hypertension; CBF: cerebral blood flow; Hypertensive Resp.: hypertensive response; Orthostatic pos.: orthostatic position on the treadmill; BP: blood pressure, *Pretest normotension with a hypertensive response to exercise indicates risk of future hypertension. * 2 Associated with decreased adrenergic activity and histamine-mediated vasodilation. * 3 Associated with hyperthermia on exertion and pre-existing dehydration (including secondary to diuretics).

**Figure 11:** – Representation of the main responses of the P, Q, R, S, T, and U waves, PR interval, PR and ST-segments, and J point during the exertion stage of an ET. A. Normal response to exercise: P wave duration reduces and amplitude increases (peaked P wave); PRi shortens with increasing heart rate (HR); PR segment remains unchanged (at baseline); Q wave increases in amplitude; R wave presents a reduction in amplitude concomitantly with the increase of the S wave; J point maintains the same baseline level or exhibits is a slight depression; ST-segment remains at baseline or may exhibit rapid upsloping depression; T wave amplitude and morphology remain unchanged, with a rapid initial component and a slow final component (may exhibit a small decrease in amplitude and reduction in duration); U wave tends to remain positive at the onset of exercise and disappear as the HR increases. B. Abnormal response: P wave exhibits increased duration and reduced amplitude; PRi remains unchanged despite increasing HR; PR segment depression >0.5 mm; Q wave amplitude unchanged; R wave unchanged despite progressive exertion and S wave reduction; J-point depression with horizontal ST-segment depression (Y-point ≥1.0 mm); reduction in amplitude of the T wave (tends to be symmetrical); negative U wave. C. Abnormal response: Increased P wave duration, with notched morphology; prolongation of PRi as the HR increases; Q wave disappears; increasing R-wave amplitude with concomitant reduction of the S wave; J-point depression with downsloping ST-segment depression (Y-point ≥1.0 mm); peaked and symmetric T wave with increased amplitude.

**Figure 12:** – ST-segment elevation patterns, including early repolarization. ST-seg.: ST-segment; ms: milliseconds. *Exercise-induced ST-segment elevation (≥1.0 mm measured at 60 ms after the J-point). **In the early repolarization pattern, ST-segment elevation should be measured 100 ms after the Jt point, and the pattern of elevation (upsloping, horizontal, or descending) should also be assessed.

**Figure 13:** – ST-segment behavior and types of ST depression. ST-seg.: ST-segment. *Upsloping, horizontal, or downsloping: depending on the HR at the time of measurement, the Y point will be 60 or 80 ms from the J point. **Slow upsloping: measured at point Y within 80 ms of the J point.

**Figure 14:** – Example of evaluation of the QT interval, QTc, and QTc hysteresis calculation.

**Figure 15:** – Main mechanisms and factors involved in arrhythmogenesis during ET. *Remain as potential mechanisms throughout all phases of the ET. ↑ = increase. O 2 : oxygen.

**Figure 16:** – Oximeter plethysmographic waveform behavior. Normal (A), in low perfusion (B), and in the presence of artifacts (movement [C] and noise [D]).

**Figure 17:** – Biomarkers with potential for adjunctive use in ET/CPET. Resp.: response; S.: system; NS: nervous system; ANP: atrial natriuretic peptide; BNP: B-type natriuretic peptide; NT-proBNP: inactive N-terminal fragment of B-type natriuretic peptide; GDF-15: growth differentiation factor-15; SST2: interleukin-1 receptor protein; u-hscTnI: ultra-high sensitivity cardiac troponin-I; CK-Mb: creatine kinase-MB fraction; TRAIL: tumor necrosis factor-related apoptosis-inducing ligand; MR-proADM: midregional pro-adrenomedullin; LDL: low-density lipoprotein; proET-1: endothelin 1 precursor peptides; NGAL: neutrophil gelatinase associated lipocalin; NAG: N-acetyl-beta-D-glucosaminidase; FGF23: fibroblast growth factor 23; CRP: C-reactive protein; TNF-α: tumor necrosis factor alpha; Lp-PLA2: lipoprotein-associated phospholipase A2 (platelet activating factor); APO-1: apolipoprotein A-1; TIMP-1: tissue inhibitor of metalloproteinase 1; MMP: matrix metalloproteinases; IL-6: interleukin-6.

**Figure 18:** – CPET equipment: air flow and volume sensors, O 2 and CO 2 analyzers, ECG, stethoscope/sphygmomanometer, and specific hardware/software.

**Figure 19:** – Integrated, interdependent participation of anaerobic and aerobic metabolic pathways in physical exertion.

**Figure 20:** – Anaerobic threshold (VT1 or lactate threshold) as a function of exercise intensity (% of VO 2 max) in sedentary and trained individuals.

**Figure 21:** – Increase in VE is initially dependent almost exclusively on VT, which soon plateaus; then becomes dependent solely on the increase in FR.

**Figure 22:** – Oxygen consumption (VO 2 ) in absolute values (L/min), relative values (mL/kg/min), and metabolic equivalents of task (METs) during a maximal CPET. A characteristic VO 2 curve plateauing at peak exertion indicates that the patient has reached VO 2 max.

**Figure 23:** – Oxygen consumption (VO 2 ) in absolute values* and workload in watts (W) during CPET. A VO 2 curve with no plateau at peak exertion indicates that the patient has not reached VO 2 max. (This image is purely illustrative: in practice, VO 2 is usually presented in values relative to body weight.)

**Figure 24:** – Anaerobic threshold (VT1) as determined by the VE/VO 2 (A) and VCO 2 /VO 2 (B) ratios.

**Figure 25:** – Second ventilatory threshold (VT2), derived from the ventilatory equivalent of CO 2 (VE/VCO 2 ), which is abnormal in heart failure (HF) and normal in healthy individuals. The VE/VCO 2 slope is a predictor of mortality and hospitalization in HF, indicating worse prognosis if ≥34.

**Figure 26:** – Ventilatory equivalents of oxygen (VE/VO 2 ) and carbon dioxide (VE/VCO 2 ) and their relationships to workload and blood lactate curve. Also shown are the ventilatory thresholds (VT1 and VT2) and cardiorespiratory optimal point (COP).

**Figure 27:** – End-tidal partial pressures of oxygen (PETO 2 ) and carbon dioxide (PETCO 2 ), with determination of ventilatory thresholds (VT1 and VT2).

**Figure 28:** – Abnormal oxygen pulse morphology due to myocardial ischemia. A = decreasing; B = flattening.

**Figure 29:** – ∆VO 2 /∆WR curve characteristic of myocardial ischemia.

**Figure 30:** – Oxygen uptake efficiency slope (OUES) in subjects with good (healthy) and low heart failure efficiency.

**Figure 31:** – Illustration of exercise oscillatory ventilation in the CPET of a patient with severe heart failure with reduced ejection fraction (HFrEF).

**Figure 32:** – Oxygen kinetics during exercise and recovery, with their respective T½ values, in a healthy individual and a patient with HFrEF.

**Figure 33:** – CPET measurements and variables and key inter-relationships. The system/software integrates information and returns multiple variables of clinical interest based on ventilatory (via flow transducers and inspired air volume), metabolic (via expired gas analysis: LV, FEO 2 , FECO 2 ), and hemodynamic (ECG, HR, BP) measurements. From these measurements, multiple variables are derived, which can then be analyzed graphically. VO 2 : oxygen consumption; VCO 2 : CO 2 production; VE: minute ventilation; EOV: exercise oscillatory ventilation; VR: ventilatory reserve; MVV: maximal voluntary ventilation; VT1: first ventilatory threshold; VT2: second ventilatory threshold; PETO 2 : end-tidal partial pressure of oxygen; PETCO 2 : end-tidal partial pressure of carbon dioxide; VO 2 max: maximum oxygen consumption; %VO 2 max:percentage of maximum oxygen consumption; HR: heart rate; RF: respiratory frequency; WL: work load in watts; VO2RD: oxygen uptake recovery delay; OUES: oxygen uptake efficiency slope; T½: time required for a 50% drop from VO 2 peak in recovery; ΔVO 2 /ΔWR: delta VO 2 to delta work load ratio; COP: cardiorespiratory optimal point; OP: oxygen pulse; RQ: respiratory quotient.

**Figure 34:** – Choice of stress modality to be combined with myocardial perfusion scintigraphy for the diagnosis of coronary artery disease. LBBB: left bundle branch block; ET: exercise test; WPW: Wolff-Parkinson-White; HR: heart rate; MPS: myocardial perfusion scintigraphy. * 1 Functional capacity to perform estimated daily physical activities >5 METs and ability to perform the necessary effort on whichever ergometer is available; * 2 Alternative: regadenoson (not yet available in Brazil); * 3 May be performed at the discretion of the requesting/referring physician. * 4 As this is a diagnostic test, a new myocardial perfusion scan under pharmacologic stress (dipyridamole or adenosine) or hybrid stress must be performed to ensure accuracy.

**Figure 35:** – Representation of an exercise stress echocardiogram performed on a bicycle/cycle ergometer. BP: blood pressure; ECG: electrocardiogram; ECHO: echocardiogram; min: minute.

**Chart 1:** – Absolute contraindications to ET and CPET

Absolute contraindications to ET and CPET
– Acute pulmonary embolism or infarction
– Acute febrile or serious illness
– Mental or physical disability that precludes proper adherence to the exercise protocol
– Substance intoxication
– Hydroelectrolytic disorders and metabolic disturbances
– Atrioventricular block deemed to increase risk of events/complications*
– Persistent resting systolic blood pressure ≥180 mmHg or diastolic blood pressure >110 mmHg**
– Hypertensive crisis (urgency or emergency)**
– Uncontrolled hyperthyroidism
– Recent retinal detachment (during recovery phase***)
– Decompensated cyanotic congenital heart disease
– Acute myocardial infarction (<5 days or complicated)
– Unstable angina
– Uncontrolled cardiac arrhythmias
– Symptomatic severe aortic stenosis
– Decompensated heart failure
– Acute myocarditis or pericarditis
– Acute aortic dissection
– Aortic or other aneurysm meeting criteria for intervention
– Decompensated lung disease
– Decompensated diabetes mellitus****
*Considered at high risk of events/complications: type II second-degree AV block; 2:1 AV block; high-grade block; third-degree (complete) AV block (unless congenital). **Hypertensive episode: acute elevation of systolic blood pressure (BP) ≥180 mmHg and/or diastolic BP ≥120 mmHg, which may or may not result in target organ damage. Divided into hypertensive urgency (BP elevation with no target organ damage and no risk of imminent death; allows BP reduction within 24 to 48 hours) and hypertensive emergency (BP elevation with acute or progressive target organ damage and immediate risk of death; requires rapid, gradual BP reduction within minutes to hours via intravenous medication).^215^***Medical clearance for physical activity, especially at moderate/high intensity, requires evaluation and approval by an ophthalmologist.^216,217^****Patients with type 2 diabetes who have performed self-monitoring of blood glucose immediately before the test or on the day of the test: discontinue ET if blood glucose >300 mg/dL (16.7 mmol/L). Patients with type 1 diabetes who have performed self-monitoring of blood glucose: discontinue ET if blood glucose >350 mg/dL; if 251-350 mg/dL, ketone testing is suggested; if moderate-to-large amounts of ketones are detected, discontinue ET.^*64,218*^

**Chart 2:** – General indications for CPET **3,5,219-223**

General indications for CPET
**1) Diseases and conditions in which adding direct determination of ventilatory parameters and exhaled breath gas analysis would contribute to diagnostic assessment, risk stratification, and definition of preventive and therapeutic management**
**2) Causal determination of factors limiting cardiorespiratory performance and underlying pathophysiological mechanisms**
**3) Differential diagnosis of dyspnea (exercise-induced asthma, HF, COPD, etc.)**
**4) Diagnosis, prognosis, and adjustment of therapy in various cardiovascular diseases (CAD, CHD, HF, etc.)**
**5) Selection of candidates for heart transplantation**
**6) Diagnosis, prognosis, and adjustment of therapy in various lung diseases (COPD, asthma, emphysema, interstitial lung disease, etc.)**
**7) Assessment of response to therapy in pulmonary hypertension and cystic fibrosis**
**8) Other scenarios:**
– Preoperative assessment for noncardiac surgery in patients with lung disease
– Assessment after lung, heart, or heart-lung transplantation
– Selection of sport modalities for competitive athletes
– Serial testing for adjustment of training intensity load in competitive athletes of predominantly aerobic activities
– Disability/work capacity/occupational medicine assessment
– Assessment and exercise prescription for cardiovascular, pulmonary, and metabolic rehabilitation
*COPD: chronic obstructive pulmonary disease; CHD: congenital heart disease; CAD: coronary artery disease; HF: heart failure.*

## *Supplemental Materials

For additional information, please click here.
